# Epigenome-wide association study of physical activity and physiological parameters in discordant monozygotic twins

**DOI:** 10.1038/s41598-022-24642-3

**Published:** 2022-11-23

**Authors:** Glen E. Duncan, Ally Avery, Jennifer L. M. Thorson, Eric E. Nilsson, Daniel Beck, Michael K. Skinner

**Affiliations:** 1 Department of Nutrition and Exercise Physiology, Elson S. Floyd College of Medicine, Washington State University, Spokane, WA 99202 USA; 2 Center for Reproductive Biology, School of Biological Sciences, Washington State University, Pullman, WA 99164-4236 USA

**Keywords:** DNA methylation, Obesity

## Abstract

An epigenome-wide association study (EWAS) was performed on buccal cells from monozygotic-twins (MZ) reared together as children, but who live apart as adults. Cohorts of twin pairs were used to investigate associations between neighborhood walkability and objectively measured physical activity (PA) levels. Due to dramatic cellular epigenetic sex differences, male and female MZ twin pairs were analyzed separately to identify differential DNA methylation regions (DMRs). A priori comparisons were made on MZ twin pairs discordant on body mass index (BMI), PA levels, and neighborhood walkability. In addition to direct comparative analysis to identify specific DMRs, a weighted genome coexpression network analysis (WGCNA) was performed to identify DNA methylation sites associated with the physiological traits of interest. The pairs discordant in PA levels had epigenetic alterations that correlated with reduced metabolic parameters (i.e., BMI and waist circumference). The DNA methylation sites are associated with over fifty genes previously found to be specific to vigorous PA, metabolic risk factors, and sex. Combined observations demonstrate that behavioral factors, such as physical activity, appear to promote systemic epigenetic alterations that impact metabolic risk factors. The epigenetic DNA methylation sites and associated genes identified provide insight into PA impacts on metabolic parameters and the etiology of obesity.

## Introduction

Regular physical activity (PA) and proper nutrition are the foundations of chronic disease prevention and treatment efforts. However, we have failed to motivate adoption and maintenance of these critical health behaviors at the population level. Strategies to increase levels of PA in the population are a critical public health goal^1–4^. The role of the “built” environment in supporting health behaviors has received increased attention over the last decade because of the failure of individual-level approaches (i.e., behavior change) to impact this population-level problem (i.e., obesity and associated metabolic syndrome). “Healthy” or “walkable” built environments provide opportunities for PA and better choices for food. These environments potentially lead to lower obesity and associated diseases^5^. Detrimental built environments include sprawling suburbs where automobiles are the only transportation option and fast-food restaurants and strip malls are numerous. This “obesogenic” environment minimizes energy expenditure and maximizes energy intake to promote obesity. While it is known that exposure to an obesogenic built environment negatively influences PA and eating behaviors, it is not clear whether exposure to an obesogenic built environment can affect on a molecular level gene activity^6^. These “molecular signatures” of obesity can be investigated through epigenetics.

Our previous work documents “quasi-causal” associations between the built environment and health behaviors in a community-based sample of identical adult twins (monozygotic, MZ) who were reared together as children, but now reside apart^7,8^. This unique sample group permits us to address environmental self-selection by accounting for genetic and shared environmental factors that would otherwise introduce selection biases confounding statistical associations. Because MZ twins have the same genetic background and are matched on numerous childhood exposures, they are an ideal sample to study epigenetics and environmental influences on health behaviors and associated health outcomes.

Genetic mutation and gene expression correlations in with twin studies have demonstrated that most genome-wide association studies (GWAS) have identified genes with a very low frequency correlation often in less than 1% of the patient population examined^9^. Although twin studies help reduce the variation of the GWAS analysis, negligible correlations or associations with genetic mutations have been observed^10^. Therefore, neither PA nor metabolic parameters (e.g., BMI and waist circumference) have been shown to have a high frequency association with genetic mutations^11^. Gene expression analysis has been more useful to correlate PA and metabolic parameters, which supports the hypothesis that gene expression is involved^12^. However, the molecular control of this gene expression and how PA may impact metabolism is unclear. Observations support a lack of genetic DNA sequence alterations in PA and metabolic pathways.

An additional molecular mechanism not involving DNA sequence alterations is epigenetics. Limited associations between PA and metabolic disease have been made using a systems epigenome-wide approach. Since the primary molecular control of gene expression involves epigenetics, environmental factors such as PA may alter epigenetic regulation of gene expression to promote the physiology observed^13^. Classic genetics and genetic mutations do not have the capacity to be environmentally responsive and promote gene expression alterations and physiologies without the inclusion of epigenetics in the process. Therefore, the current study takes a novel systems approach to examine in a genome-wide manner the impacts of PA and metabolic syndrome (e.g., obesity) measures in twin studies with controlled genetics. Epigenetics is defined as “molecular factors and processes around DNA that regulate genome activity, independent of DNA sequence, and are mitotically stable”^14^. Epigenetic factors include DNA methylation, histone modifications, chromatin structure and non-coding RNAs^14^. Epigenetics in part evolved to provide a molecular mechanism to be responsive to the environment and impact biology. Examples of environmental and behavioral factors that can regulate epigenetics to impact physiology include environmental toxicants, nutrition, and stress^14,15^. The current study extends these observations to examine the impact of PA^16^ on epigenetics and allow associations with metabolic parameters (e.g., BMI and waist circumference)^17,18^ measures using identical twins (monozygotic, MZ) to control for genetic background variation.

The initial analysis used is a standard paired comparison between two groups to identify differential DNA methylated regions (DMRs), as previously described^15^. This is ideal when the specific groups to compare are known, but not as useful with unknown potential correlations. Therefore, an additional analysis was performed involving weighted genome coexpression network analysis (WGCNA)^19^. The ability to use a WGCNA protocol was established for genetic and physiological parameter correlations^20^ with the use of primarily gene expression data^21^. The potential of WGCNA to be used for an epigenetic analysis has also been established^22,23^. The current study uses WGCNA with epigenetic DNA methylation data to correlate PA and measures of metabolic parameters (i.e., BMI and waist circumference). The epigenome gene associations are then used to correlate with PA and metabolic parameters in MZ twin samples. Therefore, positive impacts of PA to reduce measures of obesity can provide insights into the role of epigenetics and physical activity on metabolic measures.

## Results

Descriptive parameters for selected characteristics of the study participants are presented in Supplemental Table S1. The participants were on average 50.2 ± 12.6 years, 74.3% female, and most participants identified as Non-Hispanic White (94.3%). The majority of the participants were married (70.7%). Most participants had a bachelor’s degree or higher (62.6%), and more than half reported an annual income above $100,000 (55.0%). Measured physical activity (minutes of moderate to vigorous physical activity, MVPA) was higher in men (187.8 ± 167.4) compared to women (120.3 ± 131.4).

Within-pair discordance measures were calculated for objective physical activity (PA), walkability, waist size, and body mass index (BMI, kg/m^2^). Descriptive statistics for discordant pairs are presented in Supplemental Table S2. Physical activity discordance was defined as one twin having at least 150 min of moderate to vigorous PA per week, and their co-twin having less than 150 min. A higher percentage of male pairs (43.8%) were discordant for PA compared to female pairs (36.7%). Neighborhood walkability discordance was determined by one twin living in a car dependent or somewhat walkable neighborhood, and their co-twin living in a very walkable or walker’s paradise neighborhood, which involved 27.8% of male pairs and 30.8% of female pairs as discordant for neighborhood walkability. BMI discordance was defined as a difference of ≥ 5 kg/m^2^ within the twin pair, which involved 11.1% of male pairs and 13.5% of female pairs. Discordant pairs for these parameters were used for the epigenetic analysis.

Buccal cells were used as a marker cell for systemic impacts on the individuals. Buccal cell cheek swabs were obtained under the Washington State University Institutional Review Board (IRB) (#16419). Participants provided written informed consent prior to sample collection. The buccal cell collection procedure is outlined in the Methods section. The twins were sent kits with a swab brush and following collection the swab brush was sent back to the Washington State Twin Registry (WSTR) laboratory for storage at − 80 °C. At the conclusion of data collection for the full study, collected samples were sent to the Skinner laboratory at the WSU Pullman campus for processing and storage at − 80 °C. Discordant for PA, walkability, and BMI were identified and used for the study. Male and female groups were separated for the analysis (Supplemental Tables S1 and S2).

**Figure 1:**
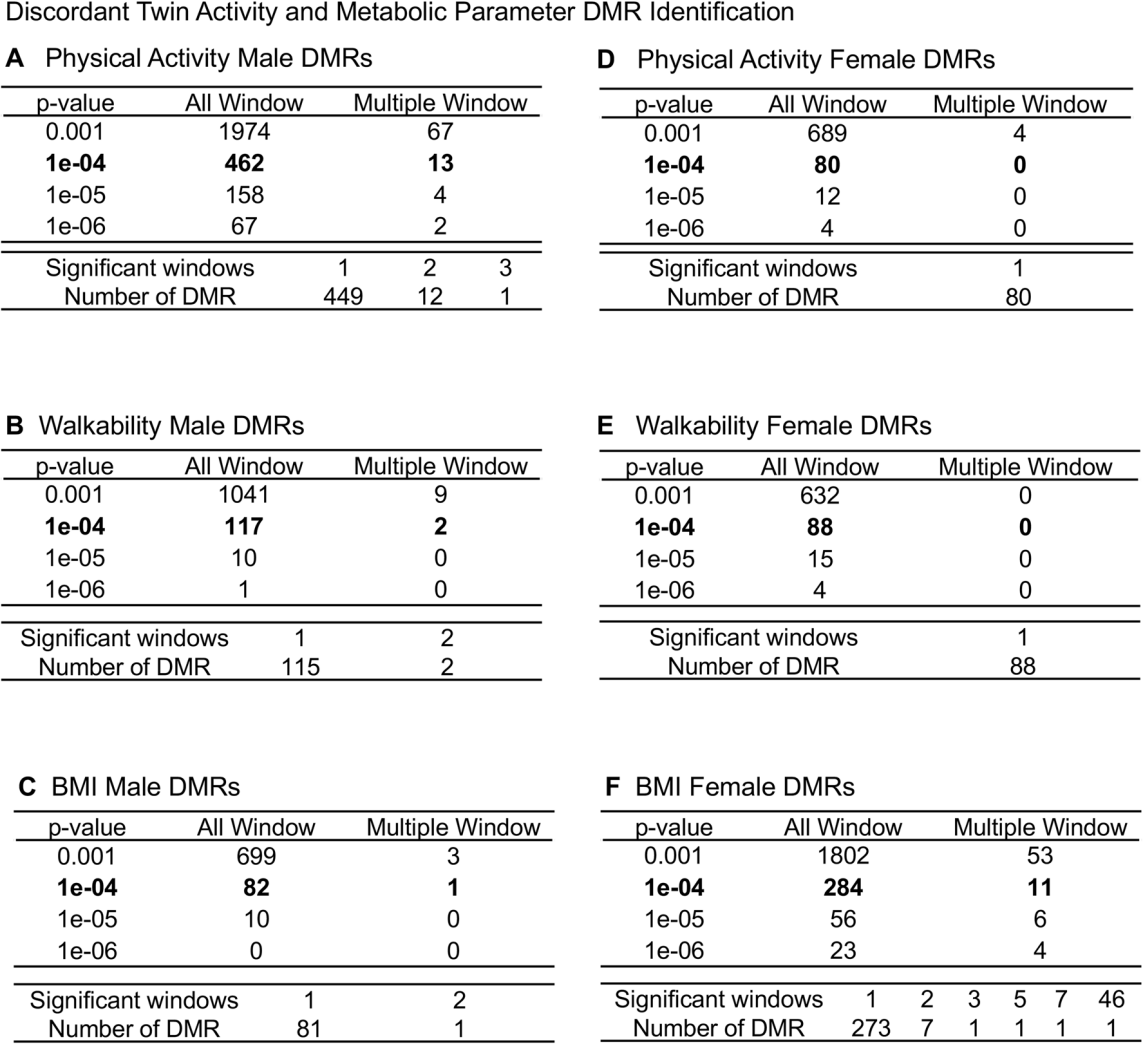
Discordant twin activity and metabolic parameter DMR identification. The number of DMRs found using different *p* value cutoff thresholds. The All-Window column shows all DMRs. The Multiple Window column shows the number of DMRs containing at least two nearby significant windows (1 kb each). The number of DMRs with the number of significant windows (1 kb per window) at a *p* value threshold of *p* < 1e−04 for DMR is bolded. (**A**) Activity male DMRs; (**B**) Walkability male DMRs; (**C**) BMI male DMRs; (**D**) Activity female DMRs; (**E**) Walkability female DMRs; and (**F**) BMI female DMRs.

**Figure 2:**
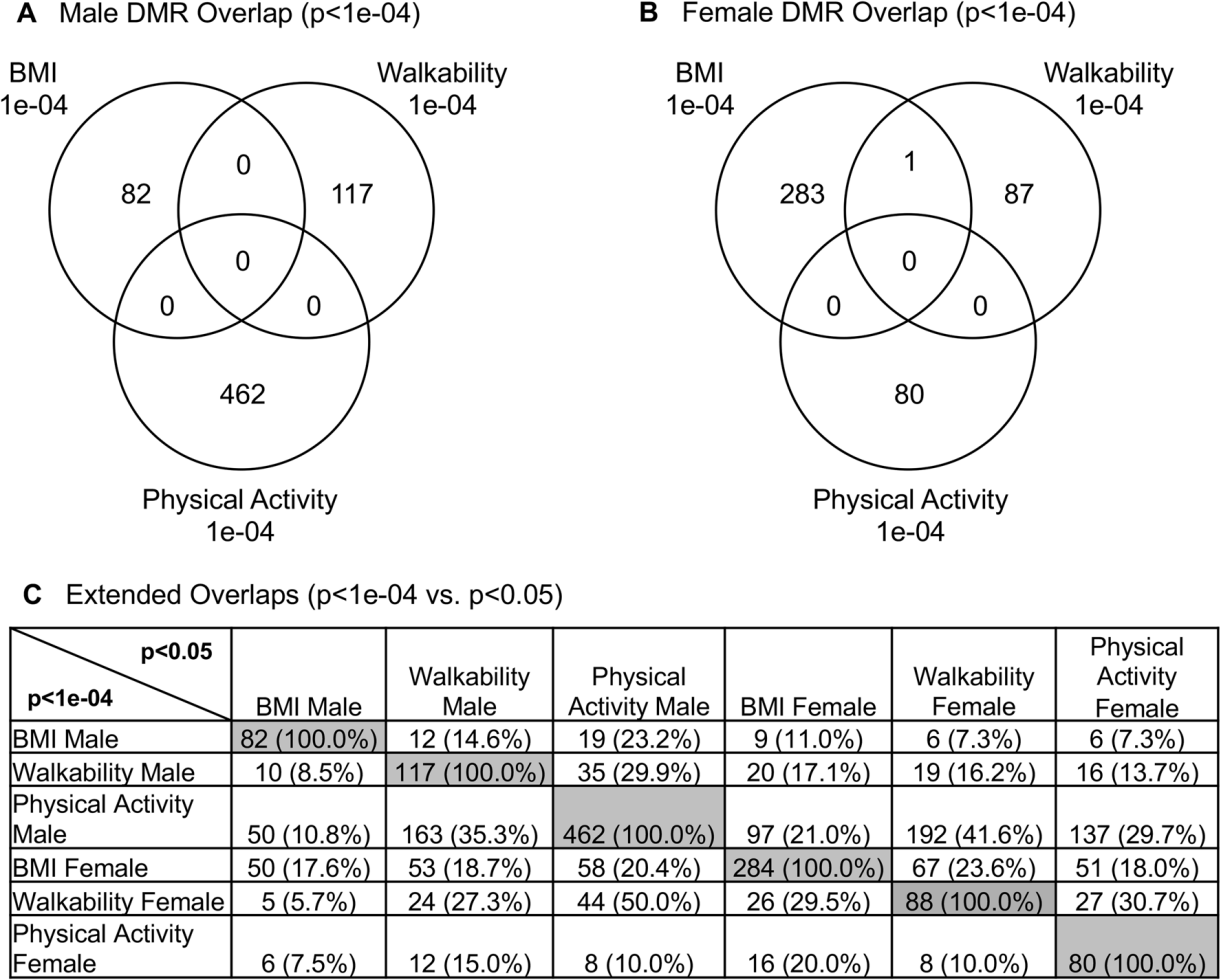
Discordant DMR overlaps. (**A**) Male DMR *p* < 1e−04 Venn diagram overlap. (**B**) Female DMR *p* < 1e−04 Venn diagram overlap. (**C**) Extended overlaps with *p* < 1e−04 and *p* < 0.05 comparisons. DMR number and percent (%) overlap presented within the rows.

A male and female separation of groups was made due to sex specific differences in epigenetics observed in previous studies^24,25^. Each individual’s buccal cell epigenetic analysis was obtained such that optimal comparisons of parameters could be assessed. The buccal cells were used as a marker cell for alterations in epigenetics for each individual. Similar analyses have been performed for disease specific comparisons, such as female susceptibility for arthritis^24–28^. The DNA was isolated from the buccal cells and used in a methylated DNA immunoprecipitation (MeDIP) protocol followed by next generation sequencing (MeDIP-Seq), as described in the Methods^24^. The analysis and comparisons between the discordant twins for PA, walkability, and BMI were made for each sex (Fig. 1). A variety of edgeR *p* values were used, and the differential DNA methylation regions (DMRs) identified for PA (lower 66 min weekly versus higher 266 min weekly, Supplemental Table S2) in the discordant twin comparison identified 462 DMRs for males and 80 DMRs for females at *p* < 1e−04 (Fig. 1A,D). Walkability (lower 25.7 versus higher 82.0, Supplemental Table S2) in the discordant twin comparison identified 117 DMRs for males and 88 DMRs for females at *p* < 1e−04 (Fig. 1B,E). BMI (lower 28.4 versus higher 35.2 kg/m^2^, Supplemental Table S2) in the discordant twin comparison identified 82 DMRs for males and 284 DMRs for females at *p* < 1e−04 (Fig. 1C,F). False discovery rate (FDR) analysis demonstrated with the male PA DMRs an FDR < 0.1, female BMI DMRs an FDR < 0.1 (15%), and with the other DMR comparisons being primarily FDR > 0.1. The overlap of the DMRs for the three measures was found to be negligible at *p* < 1e−4 between the comparison for either male or female (Fig. 2A,B). An extended overlap with the *p* < 1e−4 DMR overlap with the other comparison at *p* < 0.05 demonstrated a 5.7–50% overlap (Fig. 2C). The male and female DMR overlaps demonstrated similar percentage overlaps. This extended overlap identified highest overlap at a reduced threshold comparison for PA versus walkability.

**Figure 3:**
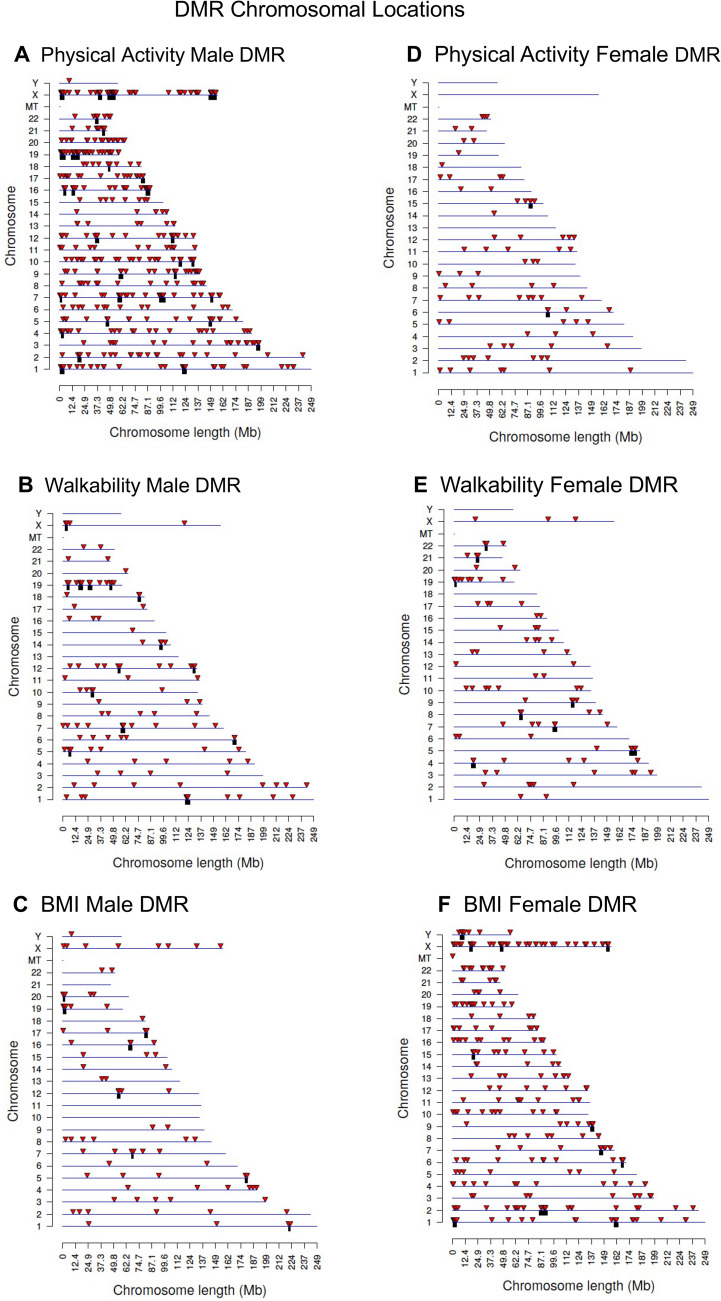
DMR chromosomal locations. The DMR locations on the individual chromosomes is represented with an arrowhead and a cluster of DMRs with a black box. All DMRs containing at least one significant window at a *p* value threshold of *p* < 1e−04 for DMR are shown. (**A**) Activity male DMR; (**B**) Walkability male DMR; (**C**) BMI male DMR; (**D**) Activity female DMR; (**E**) Walkability female DMR; and (**F**) BMI female DMR. The chromosome number versus size (megabase) is presented.

**Figure 4:**
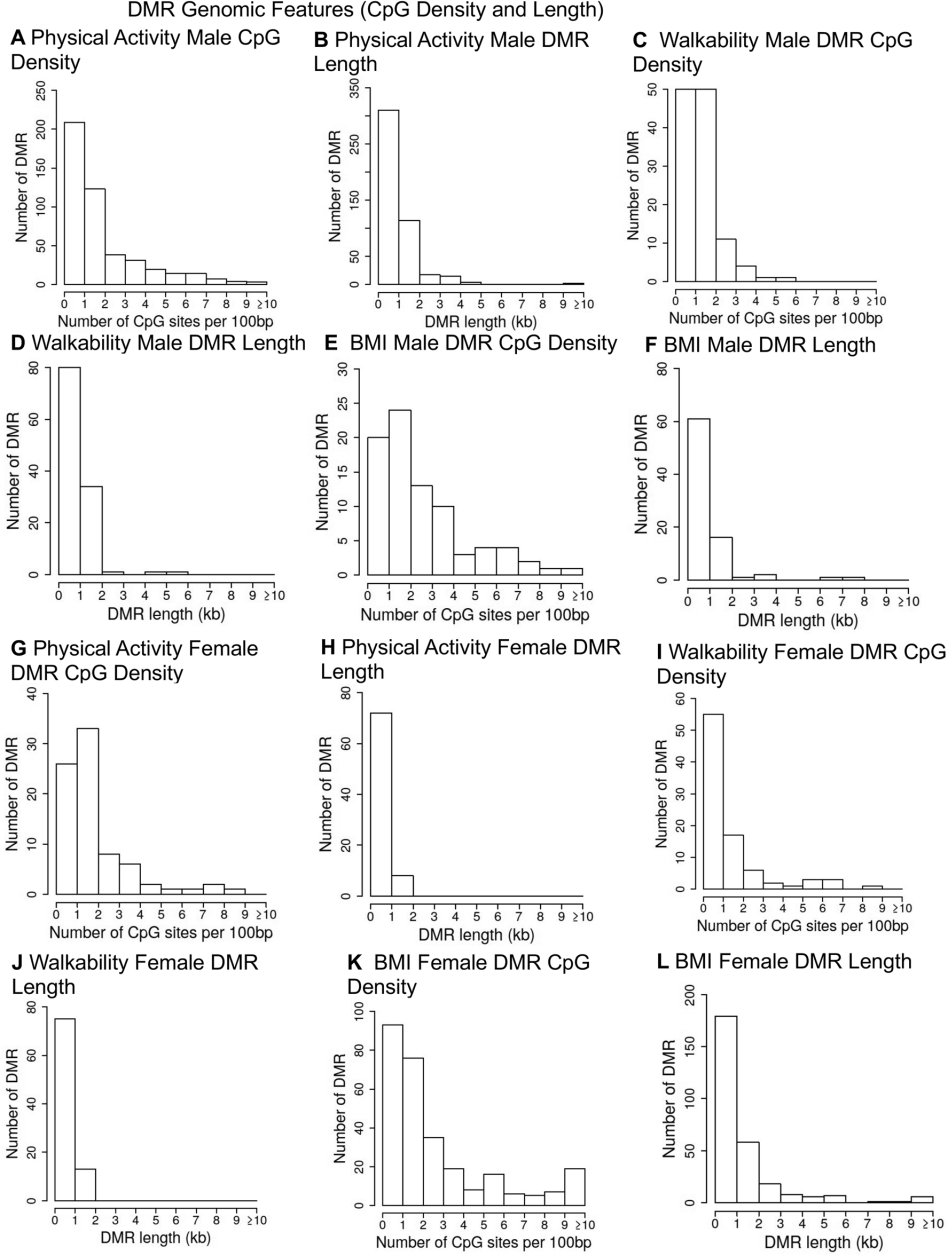
DMR genomic features CpG density and length. (**A**) Activity male CpG density; (**B**) Activity male DMR length; (**C**) Walkability male CpG density; (**D**) Walkability male DMR length; (**E**) BMI male CpG density; (**F**) BMI male DMR length; (**G**) Activity female CpG density; (**H**) Activity female DMR length; (**I**) Walkability female CpG density; (**J**) Walkability female DMR length; (**K**) BMI female CpG density; and (**L**) BMI female DMR length.

**Figure 5:**
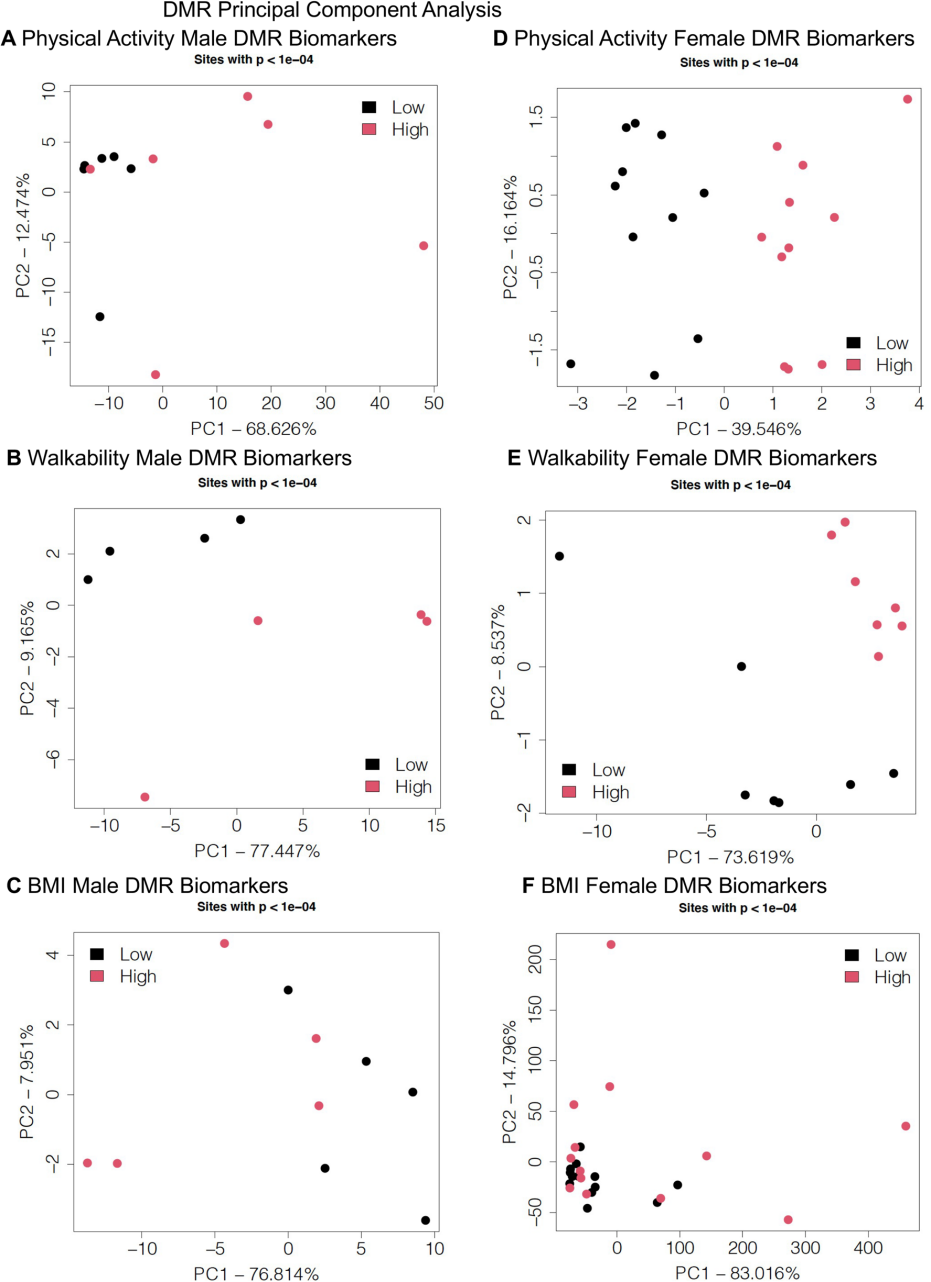
DMR principal component analysis. (**A**) Activity male DMR biomarkers; (**B**) Walkability male DMR biomarkers; (**C**) BMI male DMR biomarkers; (**D**) Activity female DMR biomarkers; (**E**) Walkability female DMR biomarkers; and (**F**) BMI female DMR biomarkers.

The genomic features of the DMRs were then investigated. The chromosomal locations demonstrated that the DMRs were throughout the genome (red arrowheads) and clusters of DMRs were also identified (black boxes), but no over-represented sites were observed (Fig. 3). The DMR CpG density was predominantly 1 or 2 CpG/100 bp for PA and walkability (Fig. 4), but the BMI DMRs did have some with higher density (Fig. 4E,K). The DMRs were predominantly 1 kb in length, with some > 2 kb (Fig. 4 and Supplemental Tables S3–S8). A principal component analysis (PCA) for DMR specific genomic features identified good separation for principal components 1 and 2 for PA and walkability (Fig. 5A–E). For the discordant twin BMI DMRs, some overlap was observed between the low and high BMI groups (Fig. 5C,F). Lists of all the genomic features for each DMR are presented in Supplemental Table S3 for PA males, Supplemental Table S4 for PA females, Supplemental Table S5 for walkability males, Supplemental Table S6 for walkability females, Supplemental Table S7 for BMI males, and Supplemental Table S8 for BMI females. The chromosomal location, CpG density, length, increase or decrease in DNA methylation (log fold change), and gene annotations and categories are listed in Supplemental Tables S3–S8.

An alternate approach to assess the epigenetic correlations within the discordant twin sets for PA, walkability and BMI used the weighted genome coexpression network analysis (WGCNA). This approach assesses the entire epigenome for variations that correlate with all parameters assessed to provide correlating coefficients and associated *p* values^19,20^. In this approach no specific comparisons are assessed, but all data for individuals are included to identify the complex epigenetic networks involved. Twin pair information is also ignored, since WGCNA does not allow such sample pairs to be considered, so all samples are considered independent. This analysis identifies clusters of data that are put in modules that are then correlated with all parameters with correlation coefficients and statistics. In this manner, groups of epigenetic sites within the genome can be identified that associate with the parameters of interest. The male and female traits and correlations within the methylation data for twins are presented in Supplemental Figs. S1 and S2. The PA sample set associated with moderate to vigorous PA (MVPA) is one of the highly connected parameters for both male and female twins. The walkability scores are also presented, as well as BMI discordance and waist circumference, in Supplemental Figs. S1 and S2.

**Figure 6:**
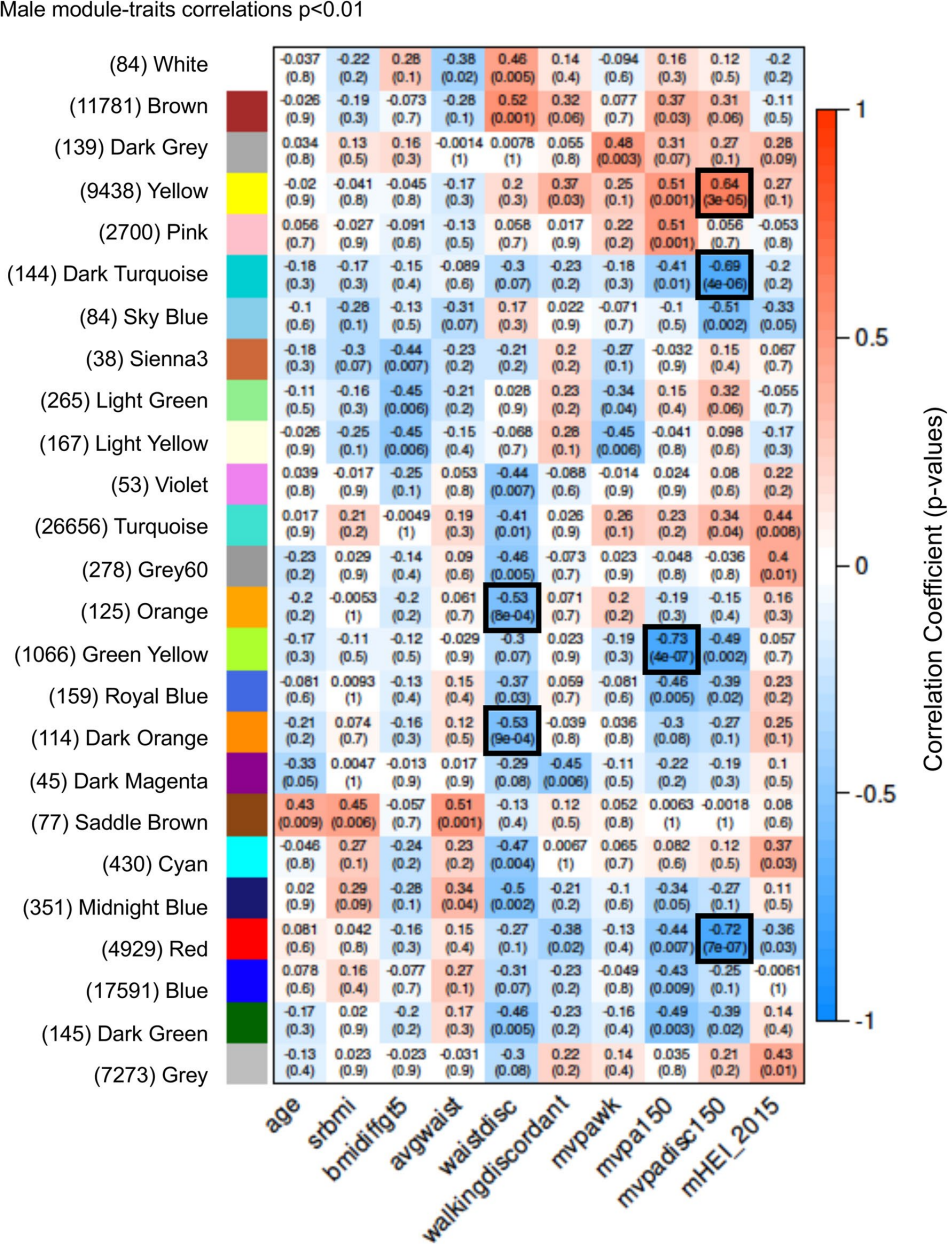
Male module-traits correlations *p* < 0.001. Rows and columns removed if no correlation met threshold.

**Figure 7:**
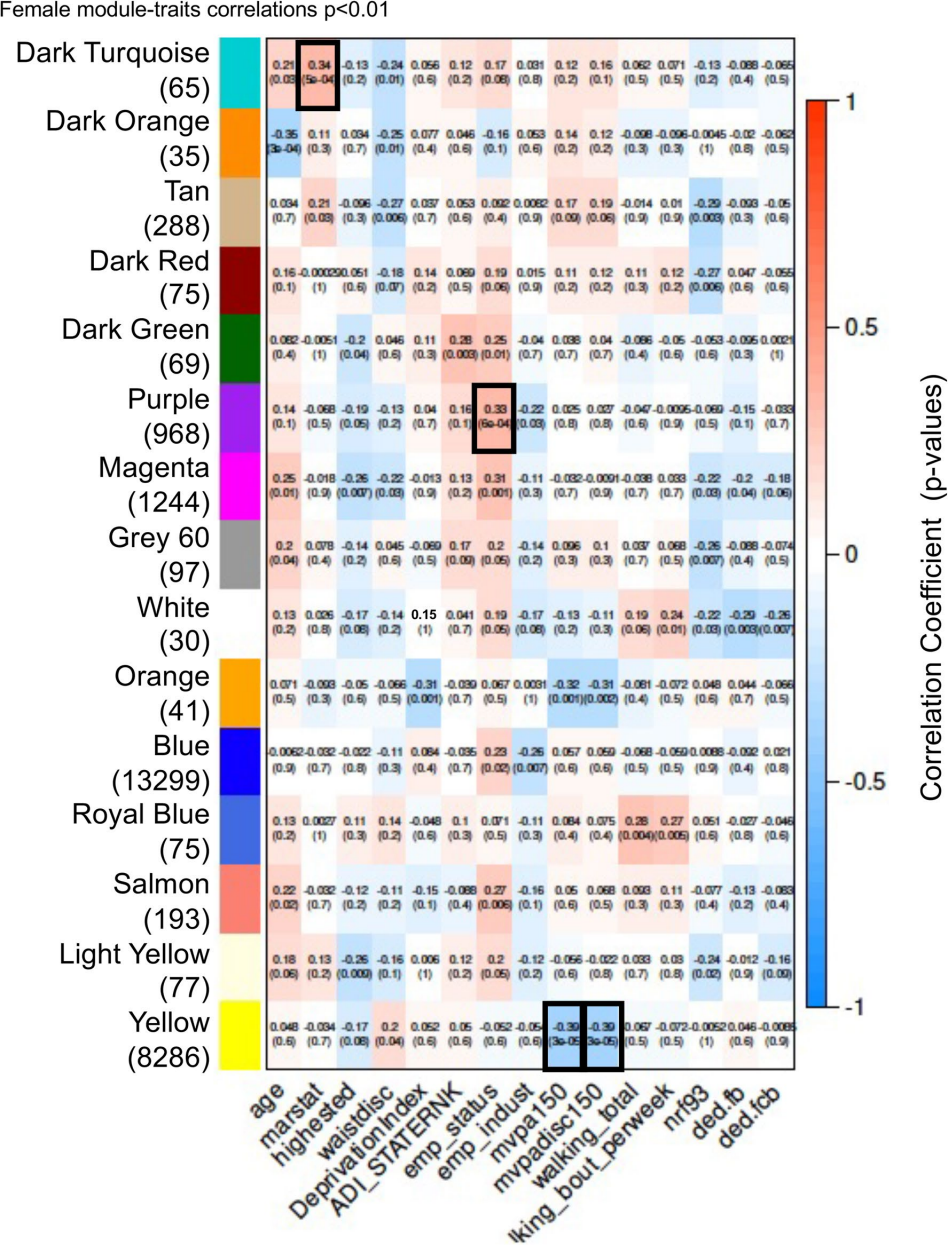
Female module-traits correlations *p* < 0.001. Rows and columns removed if no correlation met threshold.

The trait connectivity information was then used to generate module-trait relationships which provide correlation coefficients and *p* values for all DNA methylation site data (Supplemental Fig. S3 for males and Supplemental Fig. S4 for females). The data was next assessed with a presentation of module-trait relationships with a correlation *p* < 0.001 as shown for males in Fig. 6 and females in Fig. 7. The modules and number of DNA methylation epigenetic sites are presented for all traits with the correlation coefficients and *p* values listed.

A summary of the module-trait correlations with *p* < 0.001 is presented in Table 1 for both males and females for the PA set, walkability, and waist circumference (waist) parameters indicated. The female set also had module-trait correlations with *p* < 0.001 for marital status (marstat) and employment (EMP) traits (Fig. 7 and Table 1). These traits are not directly relevant or correlated to PA or metabolic traits, so are not considered further. The module epigenetic sites for DNA methylation are presented for those modules with correlated traits (*p* < 0.001), Supplemental Table S9 for male modules and Supplemental Table S10 for female modules. The gene associations were assessed when an epigenetic DNA methylation site was within 10 kb of a gene, so as to include the distal and proximal promoter region. Only the epigenetic sites with gene associations are presented in Supplemental Tables S9 and S10 for each module.

**Table 1:** (A) Male (M) and (B) female (F) summary module-trait correlations *p* < 0.001.

Module	Waistdisc-9	MVPA	MVPA disc	
**(A)**				
Yellow			M	
Dark turquoise			M	
Orange	M			
Green yellow		M		
Dark orange	M			
Red			M	

Module	Marstat	EMP Status	MVPA	MVPA disc
**(B)**				
Dark turquoise	F			
Purple		F		
Yellow			F	F

**Figure 8:**
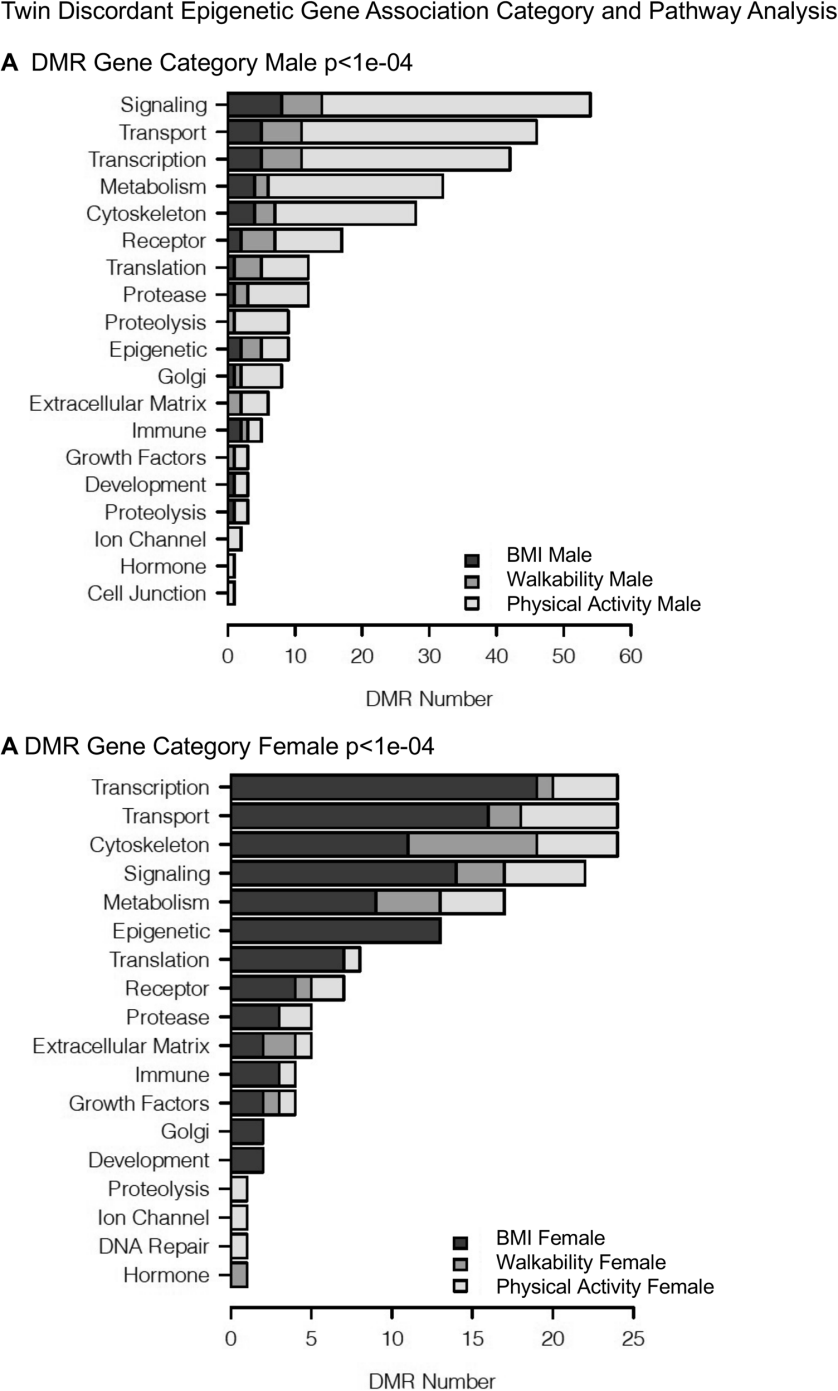
DMR gene categories. (**A**) Male (**B**) Female, with inset legends for the distinct DMR sets.

**Figure 9:**
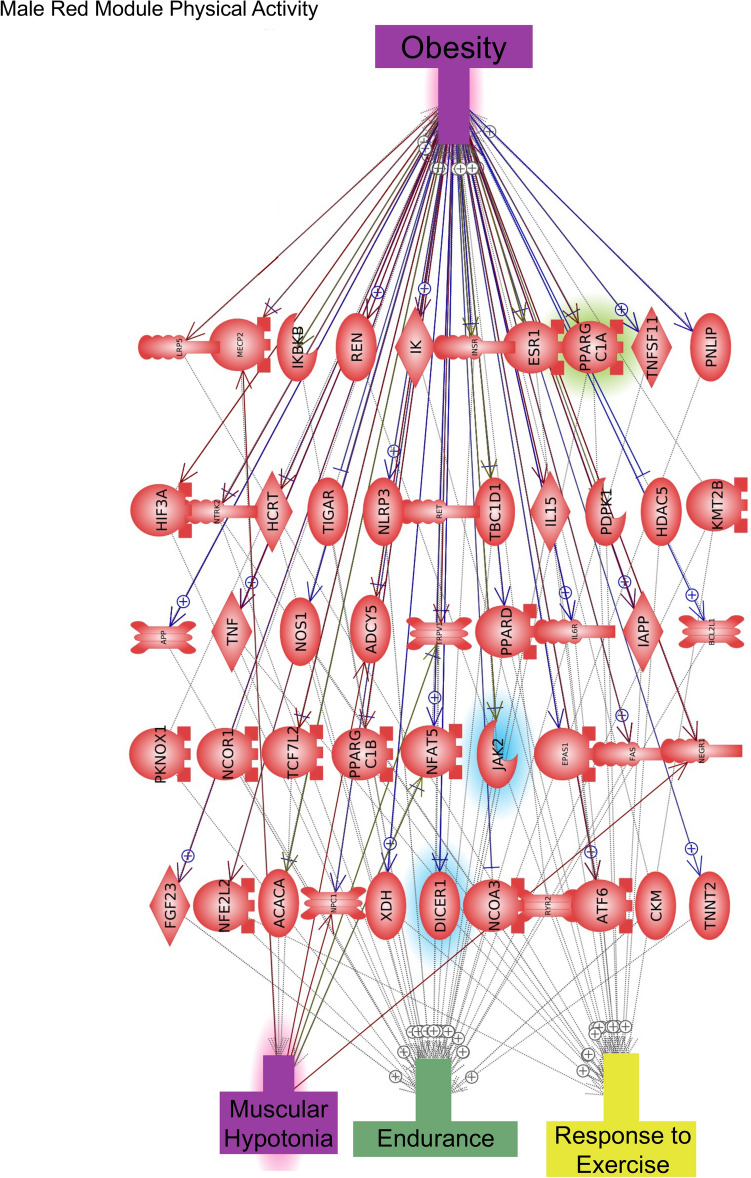
Male Red module-trait physical activity. Twin epigenetic site gene association network analysis. Male red module physical activity. Highlights indicate blue = activity, green = walkability, yellow = BMI, and red = significant over-representation. Gene links in common for obesity and physical activity parameter.

**Figure 10:**
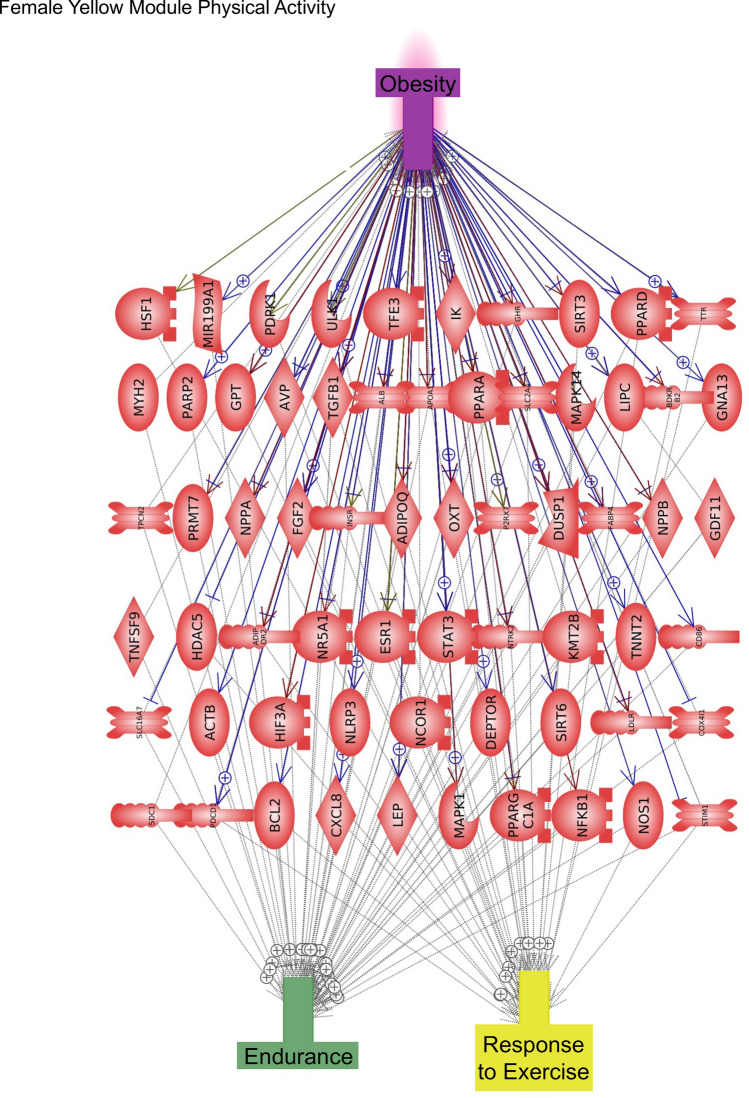
Female Yellow module-trait physical activity. Twin epigenetic site gene association network analysis. Female yellow module physical activity. Gene links in common for obesity and physical activity parameter.

The DMR sites identified with the PA, walkability, and metabolic parameters (BMI) were identified and associated genes presented in Supplemental Tables S3–S8. The WGCNA used DNA methylation sites and patterns in a genome-wide analysis to identify correlations with traits. The module-trait associations were correlated and statistically identified for each of the major trait correlation coefficients and *p* values involving PA, walkability, and metabolic parameters such as BMI and waist circumference. This was correlated to selected modules and the DNA methylation data that associated with genes identified, (Supplemental Tables S9 and S10 for males and females separately). The epigenetic gene associations for both data sets were correlated with genes known to be linked to PA and obesity measures in the discordant MZ twins using Pathway Studio software (v 12.5 Elsevier, Inc.)^29^. The DMR associated gene categories related to PA, walkability, and BMI demonstrated that signaling, transport, transcription, and metabolism were all predominant, as expected, since these are the larger gene activity families in the genome, (Fig. 8). The DMR associated gene pathways for each of the traits did not show strong overlap between the traits (Supplemental Fig. S5). The WGCNA epigenetic site analysis identified module-trait relationships with PA, walkability, and BMI (Table 1). The correlated module-trait epigenetic sites identified were associated with genes as shown in Supplemental Tables S9 and S10. Gene network analyses were performed with the DMR and module-trait associated genes. The male gene networks for physical activity and obesity measures demonstrated, for the basic DMR analysis, fewer associated genes and smaller networks (Supplemental Figs. S6 and S7) compared to those of the WGCNA module-trait analysis. The male module-trait associated gene networks involved a larger number of genes with common associations to physical activity and obesity (Fig. 9 and Supplemental Fig. S6). The female gene networks for PA and obesity measures also demonstrated a small DMR associated gene network (Supplemental Fig. S7). A large gene network with common PA and obesity gene associations involved the female yellow module (Fig. 10). The male yellow module-trait network also had a large number of associated genes in common between PA and obesity (Supplemental Fig. S6). The smaller module-trait WGCNA associated network genes in common with PA and obesity are also presented in Supplemental Fig. S6. Observations indicate the DMR approach did provide some similar genes in the module-trait network that are indicated with color highlights, however, the majority of associated genes with PA and obesity were identified with the WGCNA. Finding genes that have previously been shown to be associated with both PA and obesity helps validate the approach used for both the DMR and WGCNA analyses. As expected, the male and female DMRs, WGCNA modules and gene networks were primarily distinct, due to the sex differences in the epigenome.

## Discussion

Epigenetics has a critical role in the regulation of gene expression and maintenance of genome biology^30^. In contrast to genetics, environmental exposures can directly regulate epigenetic processes to subsequently impact gene expression, cell and developmental biology, and the physiology of the organism^31^. These exposures can include nutrition, stress, and toxicants to impact epigenetic processes that in turn impact physiology and associated pathologies^15^. Physical activity (PA) is a common health behavior that is influenced by a number of factors, including environmental exposures, and that in turn impacts biology and disease susceptibility^32^. The current study was designed to minimize genetic variation using monozygotic (MZ) twins to identify DMRs among pairs who are discordant in PA, walkability (an environmental exposure related to PA), and BMI and related metabolic parameters (e.g., waist size). The direct epigenetic associations with PA and metabolic parameters were assessed.

All cell types of an organism have the same DNA sequence, but develop cell specificity through cell specific epigenomes^33^. Therefore, mixed cell populations work well for genetic analysis, but are not useful for epigenetic analysis. This is why many mixed cell (i.e., blood) analyses have been difficult to interpret^34^. One of the easiest purified cell populations to collect from humans are buccal swab cheek cells. This can be used as a marker cell for exposures and disease susceptibility. Recent studies have demonstrated buccal cells have epigenetic biomarkers for diseases such as arthritis or preterm birth^24,26^. Since ancestral and early life exposures can impact the majority of different cell types development and epigenetics, the current study used buccal cells as marker cells to identify physical activity exposure effects on epigenetics and make associations to metabolic parameters (e.g., BMI).

The initial analysis involved a comparison between MZ twins with discordant PA, where one twin had at least 150 min per week of moderate to vigorous PA while their co-twin had less than 150 min. This threshold is based on guidelines for activity levels in the U.S. population^1^. Using a direct comparison for DMRs, an epigenetic signature for PA was identified. When this was correlated with obesity measures such as body mass index (BMI) and waist size, an epigenetic DMR signature was also observed. Therefore, PA promoted epigenetic DMR alterations that correlated with a change of metabolic parameters within the sample population. Specific epigenetic DMR signatures were obtained for PA, walkability, and BMI. This analysis identified individuals with a specific discordance and supports the concept that PA and associated metabolic measures have an epigenetic component that allows a mechanistic understanding of the phenomenon.

An alternate approach examined the epigenetics on a genome-wide level for all individuals to identify epigenetic alterations that correlated to the PA, walkability, and metabolic measures. A weighted genome coexpression network analysis (WGCNA) allows such a genome-wide correlation^19,20^. Although primarily used for gene expression analysis, the epigenome can also be assessed to then correlate with genomic actions, gene associations, and physiological parameters^22,23^. Similar observations were made in WGCNA module-trait analyses that PA, walkability, and metabolic parameters (e.g., BMI) did have epigenetic alterations that correlated. The modules identified with statistically significant correlations contained DNA methylation alterations and associated genes that were identified.

The DNA methylation WGCNA module and DMR associated genes were identified and compared. The DNA methylation alterations had associated genes that have previously been shown to be involved in PA and obesity parameters (Figs. 9 and 10). Although a comparison demonstrated male and female epigenetic changes and associated genes were distinct, similar gene pathways and networks were involved and common PA and obesity associated genes were observed for both male and female networks. The current study demonstrates physical activity (exercise response and duration) through epigenetic (DNA methylation) alterations can impact associated gene expression events that correlate with altered obesity measures (Figs. 9 and 10 and Supplemental Figs. S6–S7). This provides a molecular link between PA with the physiology and disease parameters observed in this MZ twin study. The WGCNA analysis also identified other clinical parameters within the twin population investigated. One male parameter that was found to have a correlation was deprivation index, Supplemental Figs. S1 and S2. The females had correlations with marital status (Marstat) and employment (EMP status) that are not directly relevant to the PA and metabolic measures. Future studies will need to further investigate these correlated parameters.

Observations from the current study clearly identified that physical activity (PA) impacts the epigenetics of discordant MZ twins. An increase in PA is correlated with a decrease in metabolic measures such as BMI and waist circumference. Although this is expected, the current study provides direct molecular insights into how this correlation exists. Genetic sequence mutation associations with PA and metabolic disease have not provided high frequency events (i.e., generally less than 1% of study population) that could explain the observations. No previous GWAS correlations could be identified that were similar to the EWAS observations. This is in part due to GWAS being focused on gene bodies while EWAS is not. Since environmental epigenetics is a high frequency event that is more consistent among individuals, the alterations in DNA methylation that in turn impact gene expression known to be involved in the parameters assessed does reveal how this is correlated on a molecular level. Further research on this topic and correlations with more specific molecular and physiological parameters will help elucidate how environmental epigenetics can mediate on a molecular level how PA reduces pathologies associated with obesity and associated metabolic measures.

## Methods

### Twin clinical sample collection and information

Participants for this study were MZ twins recruited from the community-based Washington State Twin Registry (WSTR). Recruitment procedures and details about the WSTR have been described^35^. Participants in the current study previously participated in a study of objective measures of physical activity (PA) and locations among individuals residing in the Puget Sound area around Seattle between 2012 and 2015. Follow-up data collection was conducted in 2018 and 2019, 72 pairs completed follow-up collection out of the 144 pairs who completed the baseline study^6,36^. Once participants were enrolled in the study, the study coordinator sent all study materials to the participant for remote data collection. Participants wore a Qstarz BT-Q1000XT (Qstarz International Co. Ltd, Taipei, Taiwan) GPS data logger and Actigraph GT3X+ accelerometer (Actigraph Inc. Pensacola, FL) attached to a belt worn around the waist for one week. They also completed questionnaires and provided a buccal sample, with 70 complete pairs provided buccal samples. The buccal cell collection procedure involved 30 s brushing of each cheek before putting in enclosed vial for shipment by mail and then stored at − 80 °C. Materials were sent to the Skinner lab at WSU Pullman after sample and data collection was completed. Buccal swab brushes were stored at − 80 °C until use. The study protocol was approved by the Washington State University Institutional Review Board (#16419), and informed written consent was obtained from all participants prior to receiving the study materials. All methods were carried out in accordance with relevant guidelines and regulations.

### DNA preparation

Frozen human buccal samples were thawed for analysis. Genomic DNA from buccal samples was prepared as follows: The buccal brush was suspended in 750 μl of cell lysis solution and 3.5 µl of Proteinase K (20 mg/ml). This suspension was incubated at 55 °C for 3 h, then vortexed and centrifuged briefly. The lysis solution was then transferred to a new 1.5 µl microcentrifuge tube. The microcentrifuge tube with the buccal brush was centrifuged again to retain any remaining solution which was combined with the transferred lysis solution. The buccal brush was discarded and 300 µl of protein precipitation solution (Promega, A795A, Madison, WI) was added to the lysis solution. The sample was incubated on ice for 15 min, then centrifuged at 4 °C for 30 min. The supernatant was transferred to a fresh 2 ml microcentrifuge tube and 1000 µl ice cold isopropanol was added along with 2 µl glycoblue. This suspension was mixed thoroughly and incubated at − 20 °C overnight. The suspension was then centrifuged at 4 °C for 20 min, the supernatant was discarded, and the pellet was washed with 75% ethanol, then air-dried and resuspended in 100 μl H2O. DNA concentration was measured using the Nanodrop (Thermo Fisher, Waltham, MA).

### Methylated DNA Immunoprecipitation (MeDIP)

Methylated DNA Immunoprecipitation (MeDIP) with genomic DNA was performed as follows: individual DNA samples (2–4 μg of total DNA) were diluted to 130 μl with 1 × Tris–EDTA (TE, 10 mM Tris, 1 mM EDTA) and sonicated with the Covaris M220 using the 300 bp setting. Fragment size was verified on a 2% E-gel agarose gel. The sonicated DNA was transferred from the Covaris tube to a 1.7 ml microfuge tube, and the volume was measured. The sonicated DNA was then diluted with TE buffer (10 mM Tris HCl, pH7.5; 1 mM EDTA) to 400 μl, heat-denatured for 10 min at 95 °C, then immediately cooled on ice for 10 min. Then 100 μl of 5X IP buffer and 5 μg of antibody (monoclonal mouse anti 5-methyl cytidine; Diagenode #C15200006) were added to the denatured sonicated DNA. The DNA-antibody mixture was incubated overnight on a rotator at 4 °C. The following day magnetic beads (Dynabeads M-280 Sheep anti-Mouse IgG; 11201D) were pre-washed as follows: The beads were resuspended in the vial, then the appropriate volume (50 μl per sample) was transferred to a microfuge tube. The same volume of Washing Buffer (at least 1 mL 1XPBS with 0.1% BSA and 2 mM EDTA) was added and the bead sample was resuspended. The tube was then placed into a magnetic rack for 1–2 min and the supernatant was discarded. The tube was removed from the magnetic rack and the beads were washed once. The washed beads were resuspended in the same volume of 1xIP buffer (50 mM sodium phosphate ph7.0, 700 mM NaCl, 0.25% TritonX-100) as the initial volume of beads. 50 μl of beads were added to the 500 μl of DNA-antibody mixture from the overnight incubation, then incubated for 2 h on a rotator at 4 C. After the incubation, the bead-antibody-DNA complex was washed three times with 1X IP buffer as follows: The tube was placed into a magnetic rack for 1–2 min and the supernatant was discarded, then the magnetic bead antibody pellet was washed with 1xIP buffer 3 times. The washed bead antibody DNA pellet was then resuspended in 250 μl digestion buffer with 3.5 μl Proteinase K (20 mg/ml). The sample was incubated for 2–3 h on a rotator at 55 °C, then 250 μl of buffered Phenol–Chloroform–Isoamylalcohol solution was added to the sample, and the tube was vortexed for 30 s and then centrifuged at 14,000 rpm for 5 min at room temperature. The aqueous supernatant was carefully removed and transferred to a fresh microfuge tube. Then 250 μl chloroform were added to the supernatant from the previous step, vortexed for 30 s and centrifuged at 14,000 rpm for 5 min at room temperature. The aqueous supernatant was removed and transferred to a fresh microfuge tube. To the supernatant 2 μl of glycoblue (20 mg/ml), 20 μl of 5 M NaCl and 500 μl ethanol were added and mixed well, then precipitated in -20 C freezer for 1 h to overnight. The precipitate was centrifuged at 14,000 rpm for 20 min at 4 C and the supernatant was removed, while not disturbing the pellet. The pellet was washed with 500 μl cold 70% ethanol in − 20 °C freezer for 15 min then centrifuged again at 14,000 rpm for 5 min at 4 °C and the supernatant was discarded. The tube was spun again briefly to collect residual ethanol to the bottom of the tube and as much liquid as possible was removed with gel loading tip. The pellet was air-dried at RT until it looked dry (about 5 min) then resuspended in 20 μl TE. DNA concentration was measured in Qubit (Life Technologies) with ssDNA kit (Molecular Probes Q10212).

### MeDIP-Seq analysis

The MeDIP DNA samples (50 ng of each) were used to create libraries for next generation sequencing (NGS) using the NEBNext Ultra RNA Library Prep Kit for Illumina (San Diego, CA) starting at step 1.4 of the manufacturer’s protocol to generate double stranded DNA. After this step the manufacturer’s protocol was followed. Each sample received a separate index primer. NGS was performed at WSU Spokane Genomics Core using the Illumina HiSeq 2500 with a PE50 application, with a read size of approximately 50 bp and approximately 10–35 million reads per sample, and 6–10 sample libraries each were run in one lane.

### Molecular bioinformatics and statistics

Basic read quality was verified using information produced by the FastQC program^37^. Reads were filtered and trimmed to remove low quality base pairs using Trimmomatic^38^. The reads for each sample were mapped to the GRCh38 human genome using Bowtie2^39^ with default parameter options. The mapped read files were then converted to sorted BAM files using SAMtools^40^. Samples with an overall mapping less than 70% were removed from the DMR analysis along the corresponding twin samples. To identify DMR, the reference genome was broken into 1000 bp windows. The MEDIPS R package^41^ was used to calculate differential coverage between control and exposure sample groups. The edgeR *p* value^42^ was used to determine the relative difference between the two groups for each genomic window. Windows with an edgeR *p* value less than 10^–4^ were considered DMRs. The DMR edges were extended until no genomic window with an edgeR *p* value less than 0.1 remained within 1000 bp of the DMR. False discovery rate (FDR) analysis demonstrated with the male PA 255 DMRs with an FDR < 0.1 (55%), 39 female BMI DMRs an FDR < 0.1 (14%), and with the other DMR comparisons being primarily FDR > 0.1. CpG density and other information was then calculated for the DMR based on the reference genome. DMR were annotated using the NCBI provided annotations. The genes that overlapped with DMR were then input into the KEGG pathway search^43,44^ to identify associated pathways. The DMR associated genes were then sorted into functional groups by reducing Panther^45^ protein classifications into more general categories. All MeDIP-Seq genomic data obtained in the current study have been deposited in the NCBI public GEO database (GEO #: GSE216387).

### Weighted genome coexpression network analysis (WGCNA)

The weighted genome coexpression network analysis (WGCNA)^46^ was performed using the WGCNA R package^47^. All samples were considered independent for the WGCNA analyses, so twin pair correlations were not considered and were ignored. Therefore, the independent twin epigenetic information was considered in the correlations and statistics observed. All MeDIP-Seq genomic windows were ranked by the mean RPKM read depth across all samples. The top 100,000 sites were chosen for inclusion in the analysis. The size of this subset was chosen to allow for a reasonable read depth to be considered and to limit computational time (< 1wk) requirements. WGCNA is a parameter rich analysis and only limited exploration of parameter variations was performed. Modules were calculated using the *blockwiseModules* function with the following parameters: maxBlockSize = 15,000, power = 6 (female), 9 (male), TOMType = “unsigned”, minModuleSize = 30, reassignThreshold = 0, and mergeCutHeight = 0.25. The Pearson correlation was calculated for each development stage and module. The *p* value for each correlation was calculated using the *corPvalueStudent* function. Sites within each module were annotated using the same methods as the DMRs.

### Ethics

The study protocol was approved by the Washington State University Institutional Review Board (#16419), and informed written consent was obtained from all participants prior to receiving the study materials. All methods were carried out in accordance with relevant guidelines and regulations.

## Supplementary Information

Supplementary Information.

## Data Availability

All molecular data have been deposited into the public database at NCBI https://www.ncbi.nlm.nih.gov/geo/ (GEO # GSE216387), and R code computational tools are available at GitHub (https://github.com/skinnerlab/MeDIP-seq) and www.skinner.wsu.edu.

## Abbreviations

EWAS: Epigenome-wide association study
MZ: Monozygotic twins
PA: Physical activity
DMRs: DNA methylation regions
BMI: Body mass index
WGCNA: Weighted genome complex network analysis
BE: “Built” environment
GWAS: Genome-wide association studies
MeDIP: Methylated DNA immunoprecipitation
MeDIP-Seq: Next generation sequencing
PCA: Principal component analysis
MVPA: Minutes of moderate to vigorous physical activity
WSTR: Washington State Twin Registry
