# Supplementary material for: Epigenome-wide association study of physical activity and physiological parameters in discordant monozygotic twins
Source: Sci Rep. 2022 Nov 23;12:20166. doi: 10.1038/s41598-022-24642-3 (PMC9691628; doi:10.1038/s41598-022-24642-3)
Supplement: Supplementary file 1 — Supplementary Information. [file 41598_2022_24642_MOESM1_ESM.pdf]

Supplemental Figure and Table Titles and Legends

Supplemental Figure S1. Male dendrogram and trait heatmap. This figure shows how the samples cluster by read depth and the traits associated with each sample. It can be used to identify outlier samples and to determine if there are any large scale associations between sample clusters and sample traits.

Supplemental Figure S2. Female dendrogram and trait heatmap. This figure shows how the samples cluster by read depth and the traits associated with each sample. It can be used to identify outlier samples and to determine if there are any large scale associations between sample clusters and sample traits.

Supplemental Figure S3. Male module-trait relationships. This figure shows how the identified modules correlate with sample traits.

Supplemental Figure S4. Female module-trait relationships. This figure shows how the identified modules correlate with sample traits.

Supplemental Figure S5. Kegg pathway gene associations. The number in brackets indicate number associated DMR genes.

Supplemental Figure S6. Module gene associations. (A) Male yellow module activity. Legend: Highlights indicate blue = activity, green = walkability, yellow = BMI, and red = significant over-representation. **(B)** Male green-yellow module activity. No DMR genes in common. **(C)** Male orange module BMI. No DMR genes in common.

Supplemental Figure S7. DMR associated gene networks. (A) Twin activity male DMRs. **(B)** Twin walkability male DMRs. **(C)** Twin BMI female DMRs.

Supplemental Table S1. Monozygotic twin identification and parameters. (A) Male twins and **(B)** Female twins.

Supplemental Table S2. Discordant twin pairs information for each parameter.

Supplemental Table S3. Activity male DMR. DMR name, chromosome number, start nucleotide site, length (bp), number sig window, p-value, maximum log fold change (LFC), CpG number and density, gene annotation, and gene category.

Supplemental Table S4. Activity female DMR. DMR name, chromosome number, start nucleotide site, length (bp), number sig window, p-value, maximum log fold change (LFC), CpG number and density, gene annotation, and gene category.

Supplemental Table S5. Walkability male DMR. DMR name, chromosome number, start nucleotide site, length (bp), number sig window, p-value, maximum log fold change (LFC), CpG number and density, gene annotation, and gene category.

Supplemental Table S6. Walkability female DMR. DMR name, chromosome number, start nucleotide site, length (bp), number sig window, p-value, maximum log fold change (LFC), CpG number and density, gene annotation, and gene category.

Supplemental Table S7. BMI male DMR. DMR name, chromosome number, start nucleotide site, length (bp), number sig window, p-value, maximum log fold change (LFC), CpG number and density, gene annotation, and gene category.

Supplemental Table S8. BMI female DMR. DMR name, chromosome number, start nucleotide site, length (bp), number sig window, p-value, maximum log fold change (LFC), CpG number and density, gene annotation, and gene category.

Supplemental Table S9. Male module lists with genes WGCNA.

Supplemental Table S10. Female yellow module lists with genes WGCNA.

Supplemental Figure S1

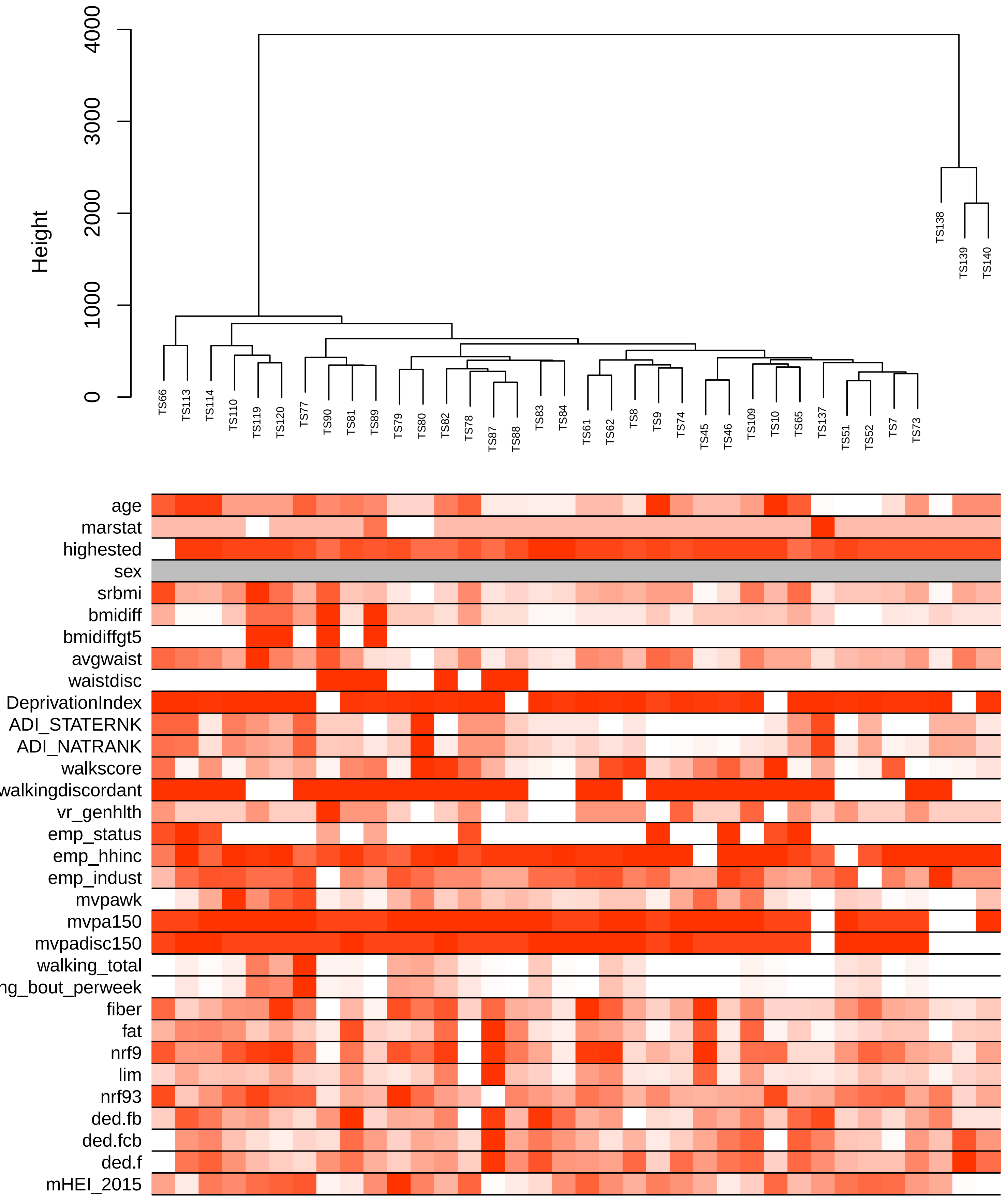

Supplemental Figure S2

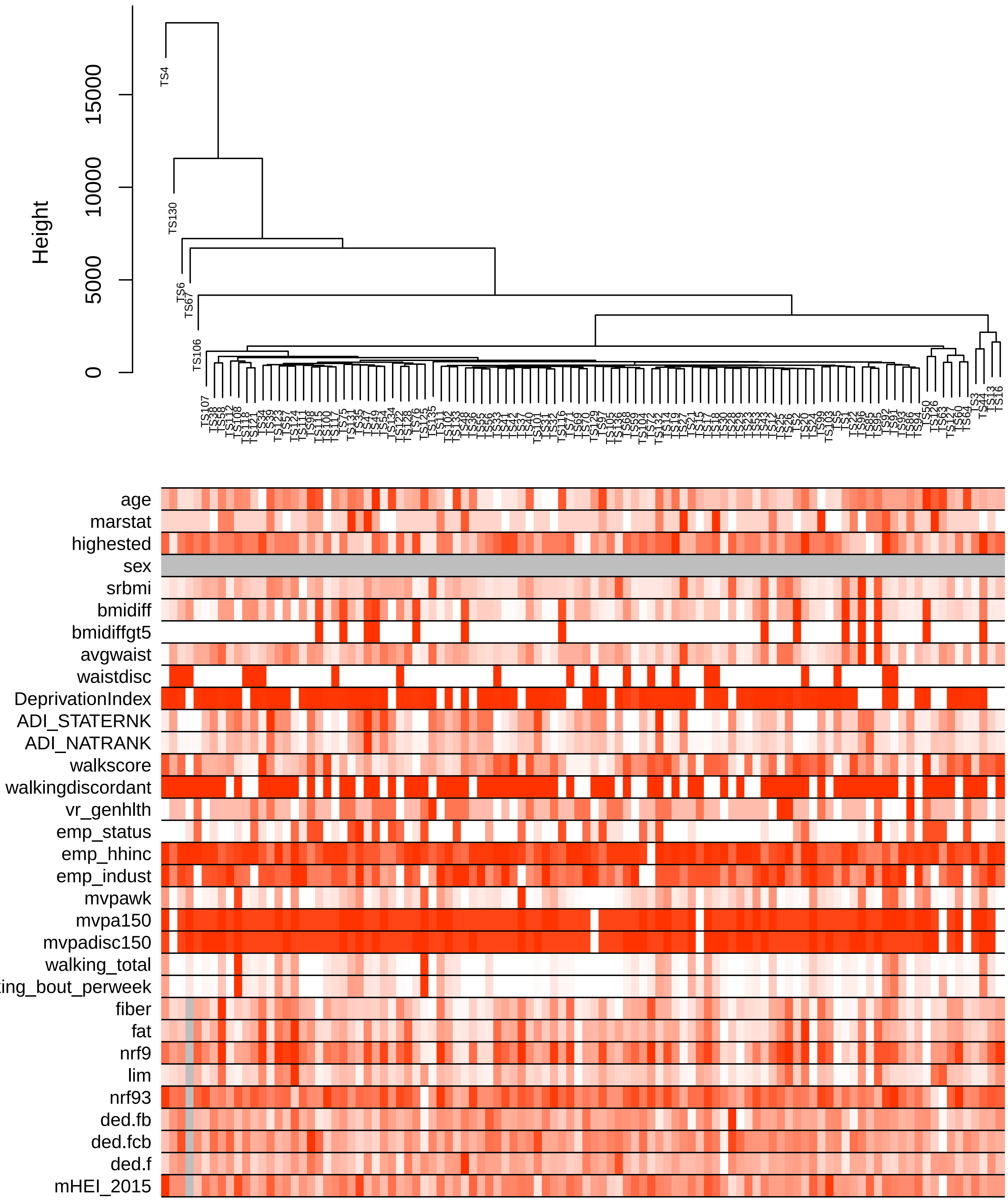

Supplemental Figure S3

Male Module-Trait Relationships

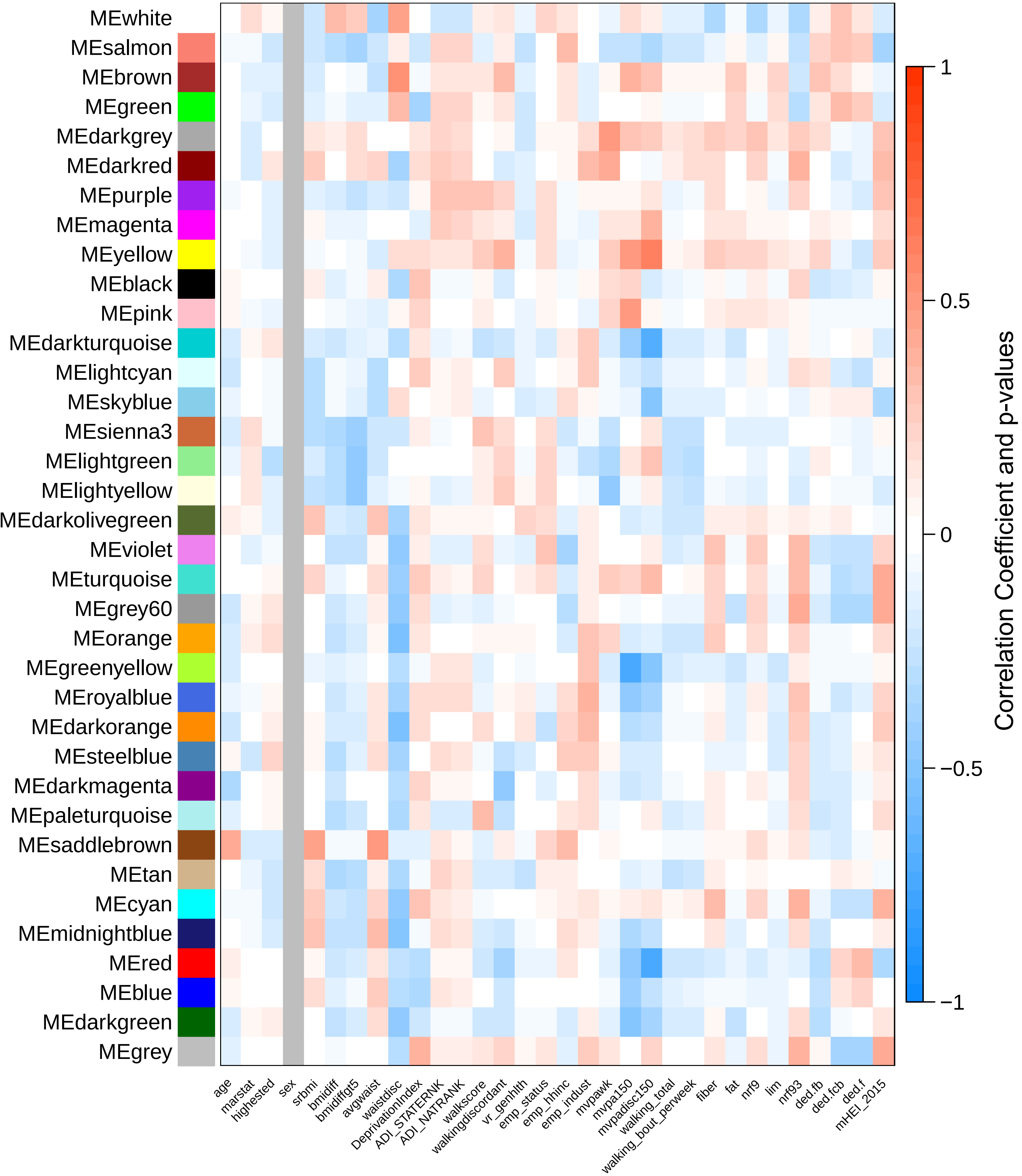

Supplemental Figure S4

Female Module-Trait Relationships

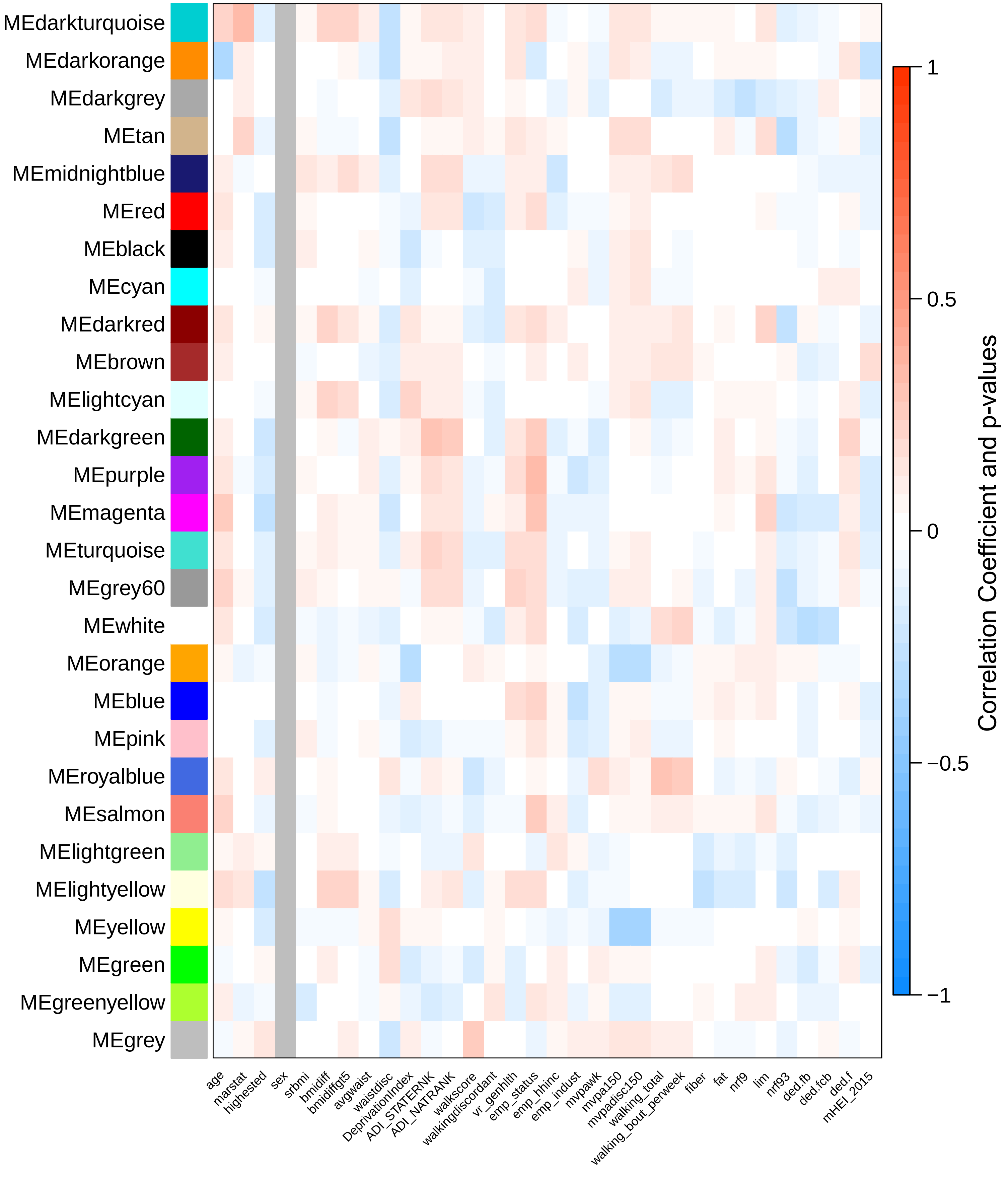

Supplemental Figure S5

Kegg Gene Pathway Associations

(A) Physical Activity Male
hsa01100 Metabolic pathways - (human) (27)
hsa04151 PI3K-Akt signaling pathway - (human) (13)
hsa04014 Ras signaling pathway - (human) (12)
hsa05200 Pathways in cancer - (human) (12)
hsa04010 MAPK signaling pathway - (human) (12)

(B) Walkability Male
hsa05206 MicroRNAs in cancer - (human) (5)
hsa05205 Proteoglycans in cancer - (human) (4)
hsa01100 Metabolic pathways - (human) (4)
hsa05022 Pathways of neurodegeneration - multiple diseases - (human) (4)

(C) BMI Male
hsa05022 Pathways of neurodegeneration - multiple diseases - (human) (6)
hsa05010 Alzheimer disease - (human) (5)
hsa05206 MicroRNAs in cancer - (human) (3)
hsa04390 Hippo signaling pathway - (human) (3)
hsa04740 Olfactory transduction - (human) (3)

(D) Physical Activity Female
hsa01100 Metabolic pathways - (human) (6)
hsa05206 MicroRNAs in cancer - (human) (4)

(E) Walkability Female
hsa05163 Human cytomegalovirus infection - (human) (3)
hsa04151 PI3K-Akt signaling pathway - (human) (3)
hsa05165 Human papillomavirus infection - (human) (3)

(F) BMI Female
hsa01100 Metabolic pathways - (human) (9)
hsa05200 Pathways in cancer - (human) (7)
hsa04010 MAPK signaling pathway - (human) (6)
hsa04390 Hippo signaling pathway - (human) (6)
hsa04014 Ras signaling pathway - (human) (6)
hsa05417 Lipid and atherosclerosis - (human) (5)

Supplemental Figure S6

(A) Male Yellow Module Physical Activity

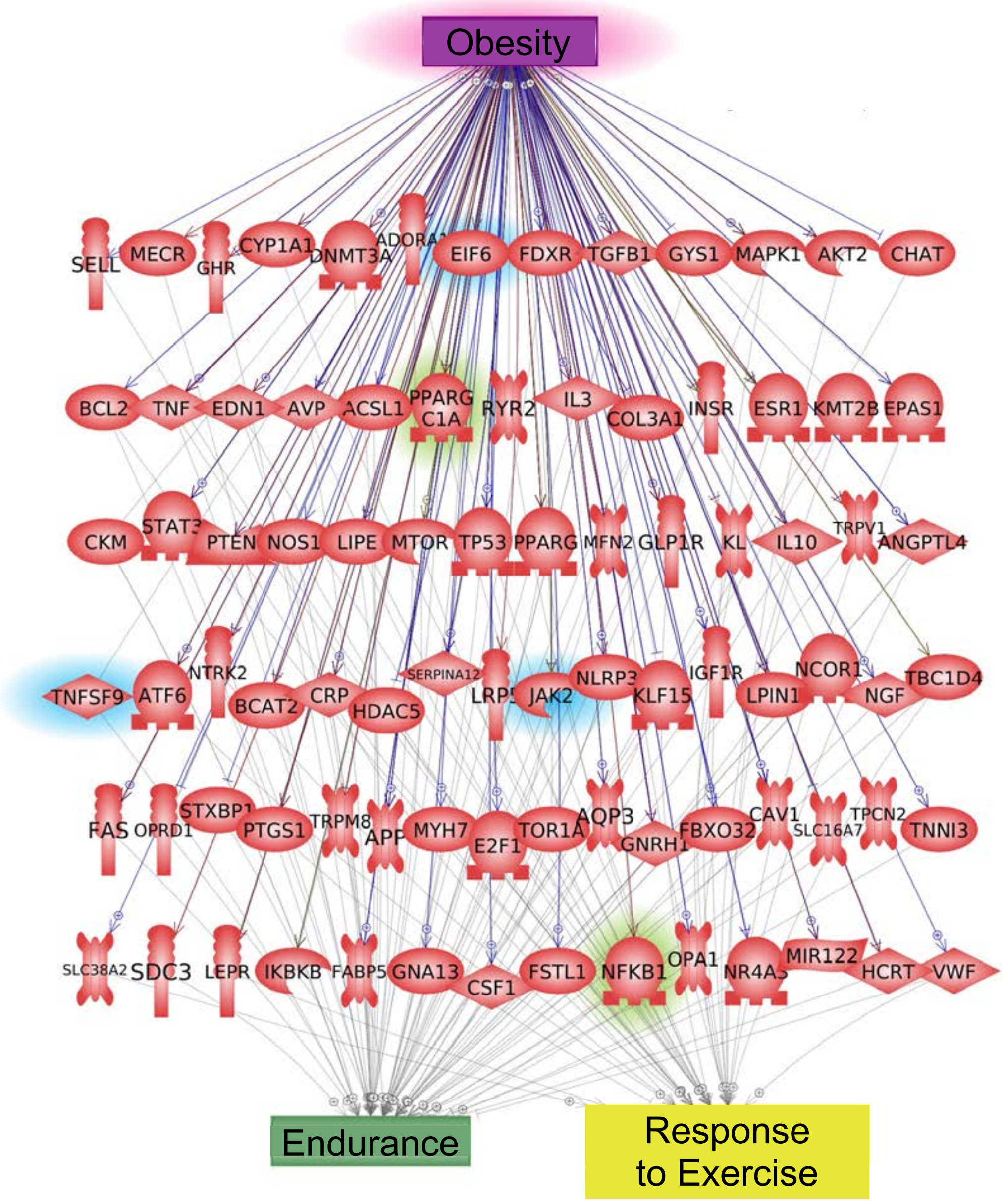

(B) Male Green-Yellow Module Physical Activity

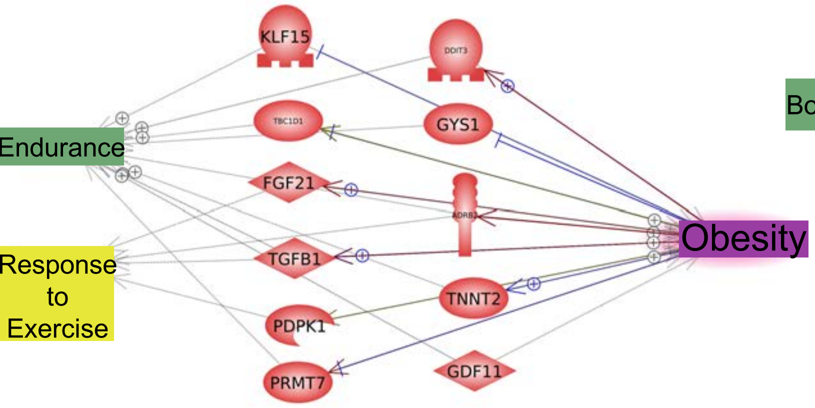

(B) Male Orange Module BMI

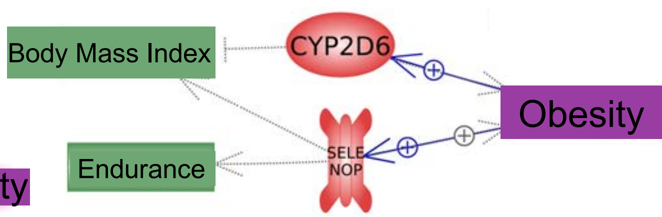

Supplemental Figure S7

(A) Twin Physical Activity Male DMR

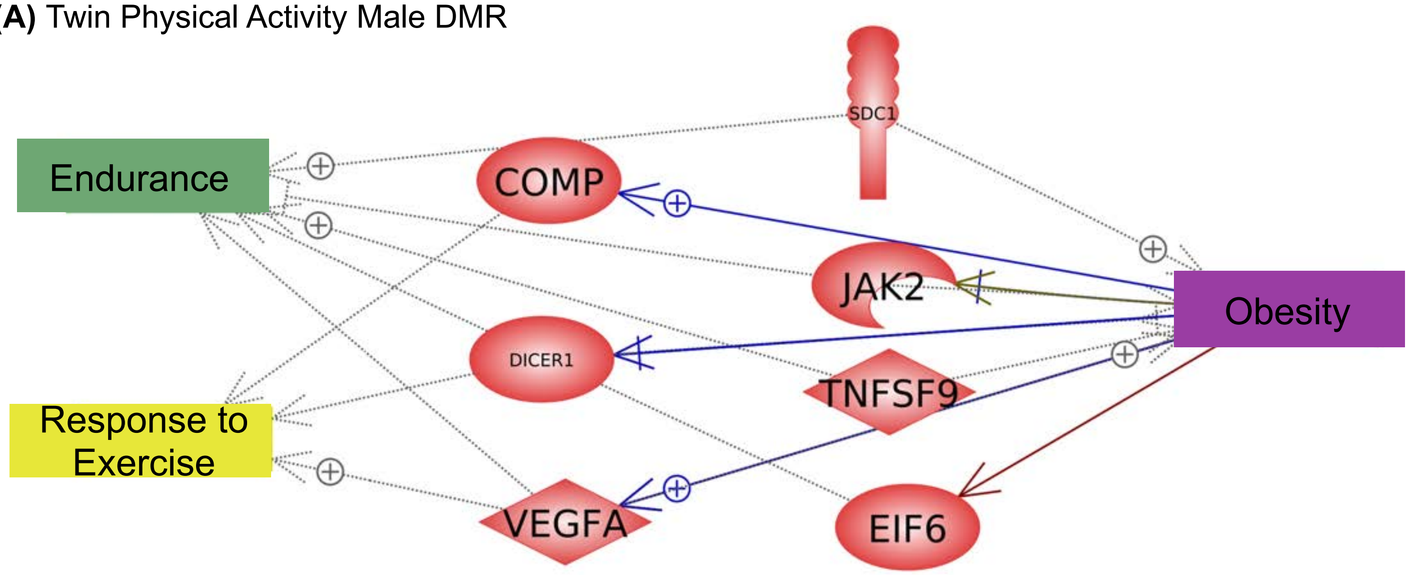

(B) Twin Walkability Male DMR

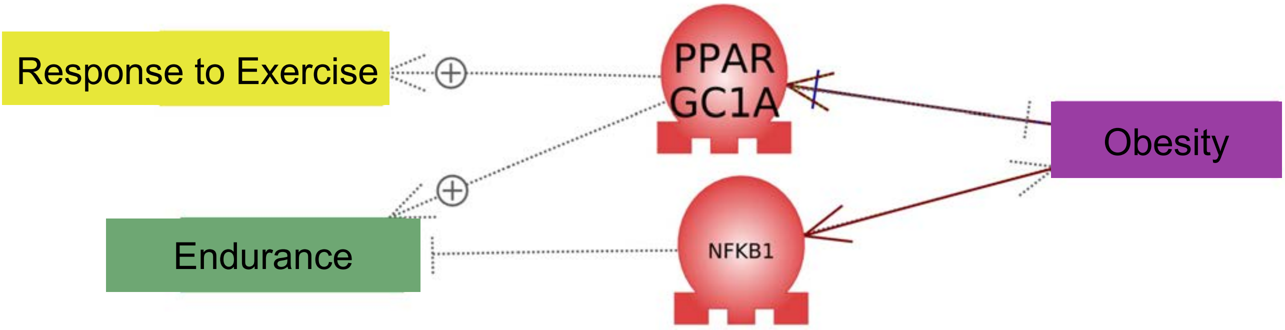

(C) Twin BMI Female DMR

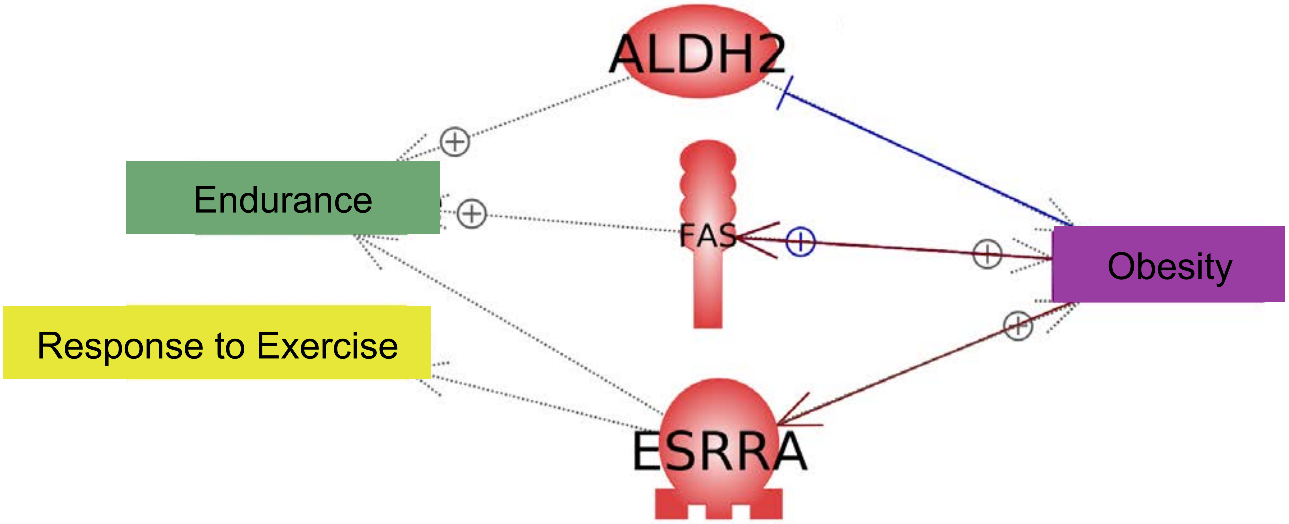

Supplemental Table S1

Monozygotic Twin Parameters

	Total	Male	Female
N (individuals)	140	36	104
Age (years)	50.2 (12.6)	51.2 (13.2)	49.9 (12.4)
Race (%)			
Non-Hispanic White	94.3	94.4	94.2
Marital Status (%)			
Single	14.3	8.3	16.3
Married/Living with Partner	70.7	86.1	65.4
Divorced/Separated	9.3	2.8	11.5
Widowed	5.7	2.8	6.7
Education (%)			
High school	9.4	0	12.5
Some college	12.9	14.3	12.5
Associate’s or Technical	15.1	8.6	17.3
Bachelor’s	33.8	34.3	33.7
Masters or higher	28.8	42.9	24.0
Household income (%)			
<50	11.4	8.3	12.5
50-90	33.6	25.0	36.5
100+	55.0	66.7	51.0
BMI (Mean ±SD)	26.7 (5.8)	27.4 (4.2)	26.4 (6.3)
Within-pair BMI difference >=5 kg/m2 (%yes)	12.9	11.1	13.5
Walkscore (Mean ±SD)	42 (29)	37.6 (30.4)	43.7 (28.6)
Within-pair “very walkable” discordant (%yes)	30.0	27.8	30.8
Weekly minutes MVPA (Mean ±SD)	137.7 (144.0)	187.8 (167.4)	120.3 (131.4)
Within-pair 150 minutes of activity/week discordant (%yes)	38.5	43.8	36.7
Waist circumference, cm (Mean ±SD)	92.6 (29.3)	100.8 (13.4)	89.7 (32.6)
Within-pair waist circumference discordant (male >=102 & <102, female >=88 & <88) (%yes)	24.3	33.3	21.2

Supplemental Table S2
Discordant Twin Pairs Characteristics

Physical Activity (PA) Discordant

	Female		Male	
Pair N	18		7	
Age	51.6 (11.8)		48.4 (14.3)	
Non-Hispanic White	94.4		100	
	Lower PA	Higher PA	Lower PA	Higher PA
BMI (Mean ±SD)	26.1 (6.2)	26.0 (7.1)	26.3 (1.4)	26.5 (2.1)
Walkscore (Mean ±SD)	45.6 (26.0)	46.3 (29.2)	25.4 (28.6)	49.6 (37.1)
Weekly minutes MVPA (Mean ±SD)	60.3 (36.9)	294.7 (128.1)	80.7 (44.4)	192.7 (47.1)
Waist circumference cm (Mean ±SD)	86.7 (17.3)	100.5 (72.7)	101.6 (10.0)	100.1 (10.7)

Walkscore (WS) Discordant

	Female		Male	
Pair N	16		5	
Age	54.8 (13.8)		53.7 (13.8)	
Non-Hispanic White	93.8		80.0	
	Lower WS	Higher WS	Lower WS	Higher WS
BMI (Mean ±SD)	27.6 (5.8)	25.8 (4.4)	25.3 (3.1)	24.7 (3.0)
Walkscore (Mean ±SD)	23.1 (21.5)	80.7 (7.2)	33.8 (20.7)	86.2 (9.3)
Weekly minutes MVPA (Mean ±SD)	75.6 (91.7)	102.5 (126.1)	191.6 (167.9)	211.0 (103.9)
Waist circumference cm (Mean ±SD)	90.8 (11.9)	87.8 (11.2)	100.6 (15.3)	92.9 (12.2)

BMI discordant

	Female		Male	
Pair N	7		2	
Age	58.1 (12.5)		54.5 (3.0)	
Non-Hispanic White	85.7		100	
	Lower BMI	Higher BMI	Lower BMI	Higher BMI
BMI (Mean ±SD)	28.0 (7.9)	34.8 (7.8)	29.8 (4.5)	36.7 (2.9)
Walkscore (Mean ±SD)	53.9 (36.9)	34.4 (13.8)	44.5 (21.9)	23.5 (23.3)
Weekly minutes MVPA (Mean ±SD)	121.0 (151.9)	110.4 (128.9)	267.3 (323.5)	198.5 (211.4)
Waist circumference cm (Mean ±SD)	93.9 (20.8)	140.5 (109.4)	98.7 (17.5)	128.3 (8.1)

Waist circumference discordant

	Female		Male	
Pair N	11		6	
Age	51.2 13.2		61.1 8.0	
Non-Hispanic White	100		100	
	Smaller waist	Larger waist	Smaller waist	Larger waist
BMI (Mean ±SD)	23.9 (3.0)	26.9 (4.5)	28.0 (3.2)	31.1 (4.1)
Walkscore (Mean ±SD)	49.4 (35.1)	24.5 (23.6)	45.7 (40.9)	33.2 (25.3)
Weekly minutes MVPA (Mean ±SD)	200.8 (147.0)	134.5 (125.4)	198.0 (225.3)	123.8 (166.9)
Waist circumference cm (Mean ±SD)	80.1 (7.3)	94.6 (8.0)	97.2 (6.5)	115.0 (6.5)

Supplemental Table S3
DMR Table Activity Male 1e-04

DMR Name	Chr	Start	Length	# Sig Win	minP	maxLFC	CpG #	CpG Density	Gene Annotation	Gene Category
DMR1:888001	1	888001	1000	1	1.72E-05	1.4148402	2	0.2		
DMR1:1315001	1	1315001	1000	1	3.14E-05	0.9061241	38	3.8	ACAP3;PUSL1;INTS11;MIR6727;CPTP	Metabolism;Translation;Transport
DMR1:2436001	1	2436001	1000	1	3.55E-06	1.3522612	3	0.3	PLCH2	Metabolism
DMR1:2901001	1	2901001	1000	1	8.37E-05	0.9697271	29	2.9		
DMR1:3799001	1	3799001	1000	1	9.59E-05	1.2128465	13	1.3	LRRC47	Translation
DMR1:9768001	1	9768001	1000	1	3.62E-05	0.7862399	28	2.8	CLSTN1	Transport
DMR1:17191001	1	17191001	1000	1	1.37E-05	1.4129277	21	2.1	LINC02783	
DMR1:22599001	1	22599001	2000	1	1.71E-07	1.5092074	48	2.4	EPHA8	Receptor
DMR1:30791001	1	30791001	2000	1	4.27E-06	1.3996625	18	0.9	LOC105378621	
DMR1:37429001	1	37429001	1000	1	1.00E-06	1.3248934	10	1		
DMR1:43349001	1	43349001	1000	1	1.86E-06	1.4081137	61	6.1	MPL;LOC105378687;CDC20	Receptor;Proteolysis
DMR1:43817001	1	43817001	1000	1	4.78E-05	1.120806	1	0.1	ST3GAL3	Transport
DMR1:58799001	1	58799001	1000	1	7.24E-06	1.4174903	3	0.3	LINC01135;LOC101926907	
DMR1:58861001	1	58861001	1000	1	8.48E-06	1.5805774	1	0.1	LINC01135;LOC107984961	
DMR1:100065001	1	100065001	1000	1	5.08E-05	1.0951083	2	0.2	MFSD14A	
DMR1:104172001	1	104172001	1000	1	8.31E-05	0.9294398	33	3.3		
DMR1:121743001	1	121743001	1000	1	4.98E-05	-1.1244488	19	1.9		
DMR1:122534001	1	122534001	3000	1	4.72E-05	-1.1073784	49	1.633333		
DMR1:123145001	1	123145001	4000	1	6.86E-05	-1.3453912	70	1.75		
DMR1:123606001	1	123606001	1000	1	2.94E-05	-1.3575218	19	1.9		
DMR1:123694001	1	123694001	1000	1	4.29E-05	-1.6203234	18	1.8		
DMR1:124406001	1	124406001	4000	1	4.63E-05	-1.4348315	73	1.825		
DMR1:152196001	1	152196001	2000	1	4.89E-06	0.9803767	81	4.05	LOC112268240	
DMR1:153513001	1	153513001	1000	1	2.98E-05	1.2596747	13	1.3		
DMR1:159945001	1	159945001	1000	1	8.90E-05	1.1000175	59	5.9	IGSF9;SLAMF9	Cytoskeleton;Immune
DMR1:162367001	1	162367001	1000	1	4.58E-05	1.0118082	55	5.5	NOS1AP;SPATA46	Cytoskeleton
DMR1:182295001	1	182295001	1000	1	7.84E-05	1.1247362	13	1.3	LINC01344	
DMR1:184861001	1	184861001	1000	1	3.95E-06	1.7869374	2	0.2	NIBAN1	
DMR1:219759001	1	219759001	1000	1	2.31E-05	1.1206057	2	0.2	LOC105372926;RNA5SP76	
DMR1:225903001	1	225903001	1000	1	5.71E-05	-1.2751545	11	1.1		
DMR1:231227001	1	231227001	2000	1	9.99E-05	-1.3703203	33	1.65	TRIM67;C1orf131	Proteolysis
DMR1:231575001	1	231575001	1000	1	6.09E-05	-1.2684277	4	0.4	TSNAX-DISC1;TSNAX	
DMR2:2501001	2	2501001	1000	1	1.12E-05	1.2889612	10	1	LOC105373389	
DMR2:17946001	2	17946001	1000	1	8.63E-07	1.2091714	4	0.4		
DMR2:19416001	2	19416001	1000	1	4.18E-05	1.2238267	5	0.5	LOC101928196	
DMR2:19690001	2	19690001	1000	1	1.15E-06	1.5613455	3	0.3		
DMR2:20204001	2	20204001	1000	1	4.67E-07	1.7344981	14	1.4	SDC1	Receptor
DMR2:28286001	2	28286001	1000	1	2.06E-05	1.1310978	18	1.8	BABAM2	
DMR2:43806001	2	43806001	1000	1	6.67E-05	-1.4537123	12	1.2	DYNC2LI1;ABCG5	Cytoskeleton;Transport
DMR2:50446001	2	50446001	1000	1	5.11E-05	1.4292399	2	0.2	NRXN1	
DMR2:54944001	2	54944001	2000	1	1.01E-05	1.8226845	13	0.65	EML6	
DMR2:70139001	2	70139001	1000	1	9.33E-05	0.925834	27	2.7	C2orf42	
DMR2:70693001	2	70693001	1000	1	6.57E-05	1.3732875	13	1.3	ADD2	Cytoskeleton
DMR2:77836001	2	77836001	2000	1	3.30E-05	1.5707086	8	0.4	LOC101927967	
DMR2:79850001	2	79850001	1000	1	4.79E-05	1.6681455	2	0.2	CTNNA2	Cytoskeleton
DMR2:87066001	2	87066001	1000	1	4.76E-06	1.2561412	7	0.7	ANAPC1P2	
DMR2:95682001	2	95682001	1000	1	6.64E-05	1.7658774	37	3.7		
DMR2:96894001	2	96894001	1000	1	6.87E-05	0.8404312	9	0.9	FAM178B	
DMR2:119073001	2	119073001	1000	1	6.15E-05	1.1127371	6	0.6		
DMR2:121610001	2	121610001	3000	2	1.38E-08	1.4667252	7	0.233333	CLASP1	Cytoskeleton
DMR2:133588001	2	133588001	1000	1	1.37E-05	1.6473019	4	0.4	NCKAP5;RN7SKP93	
DMR2:145819001	2	145819001	2000	1	4.02E-05	1.1965845	5	0.25	METAP2P1	
DMR2:173737001	2	173737001	2000	1	4.15E-05	1.0223698	9	0.45		
DMR2:205881001	2	205881001	1000	1	2.11E-07	1.8663983	1	0.1		
DMR2:240640001	2	240640001	2000	1	8.73E-06	0.8637349	75	3.75	GPR35	Signaling
DMR3:23688001	3	23688001	1000	1	5.68E-05	-1.3604513	13	1.3		
DMR3:50153001	3	50153001	2000	1	7.89E-05	1.4967272	64	3.2	SEMA3F-AS1;SEMA3F	Signaling
DMR3:52809001	3	52809001	1000	1	1.03E-05	1.107886	5	0.5	ITIH3;ITIH4	Protease; Proteolysis
DMR3:55025001	3	55025001	2000	1	5.97E-05	-1.3699426	23	1.15	CACNA2D3	Transport
DMR3:92631001	3	92631001	1000	1	6.56E-05	-1.100498	18	1.8		
DMR3:103792001	3	103792001	1000	1	1.47E-05	2.0032078	3	0.3		
DMR3:132017001	3	132017001	1000	1	6.07E-05	0.9335571	12	1.2	CPNE4	

DMR3:148237001	3	148237001	1000	1	7.50E-07	1.7186568	4	0.4		
DMR3:165476001	3	165476001	1000	1	6.71E-05	0.9372948	4	0.4	LINC01322	
DMR3:168845001	3	168845001	2000	1	2.99E-05	1.437314	9	0.45		
DMR3:169472001	3	169472001	1000	1	5.66E-05	1.1637621	1	0.1	MECOM;MECOM-AS1	Transcription
DMR3:176751001	3	176751001	2000	1	2.25E-06	1.0651029	4	0.2		
DMR3:184753001	3	184753001	2000	1	8.79E-06	1.1168959	2	0.1	LINC02069	
DMR3:185664001	3	185664001	2000	1	4.18E-05	0.8634446	20	1	IGF2BP2	Metabolism
DMR3:190831001	3	190831001	1000	1	4.42E-06	1.3425366	14	1.4	LOC100131685	
DMR3:192893001	3	192893001	1000	1	2.36E-05	1.2779679	17	1.7	MB21D2	
DMR3:196335001	3	196335001	1000	1	9.54E-05	0.854425	16	1.6	TM4SF19-DYNLT2B;TM4SF19-AS1;TM4SF19;RNU6-910P	
DMR3:196811001	3	196811001	1000	1	7.77E-06	1.2134919	6	0.6	PAK2	Signaling
DMR3:196948001	3	196948001	1000	1	9.29E-05	0.9323677	34	3.4	NCBP2;NCBP2-AS1;NCBP2AS2;PIGZ	Metabolism;Golgi
DMR4:893001	4	893001	1000	1	4.01E-05	0.912486	47	4.7	GAK	Transport
DMR4:1757001	4	1757001	1000	1	8.16E-05	1.242344	27	2.7		
DMR4:2948001	4	2948001	1000	1	8.45E-05	0.951692	21	2.1	NOP14-AS1;NOP14	Metabolism
DMR4:3617001	4	3617001	1000	1	4.21E-05	1.0030016	5	0.5		
DMR4:7351001	4	7351001	1000	1	7.38E-05	0.7673716	15	1.5	SORCS2	Transport
DMR4:8211001	4	8211001	1000	1	4.82E-05	0.8822293	17	1.7	SH3TC1	
DMR4:10843001	4	10843001	1000	1	5.49E-05	-1.4706574	13	1.3		
DMR4:18611001	4	18611001	2000	1	2.41E-09	1.7603441	5	0.25	LOC105374510	
DMR4:50938001	4	50938001	2000	1	3.38E-05	-1.4749488	31	1.55		
DMR4:54271001	4	54271001	1000	1	6.81E-05	1.2276445	2	0.2	PDGFRA	Receptor
DMR4:58991001	4	58991001	2000	1	7.52E-05	1.1042011	5	0.25	LOC105377246;LINC02429;LINC02619	
DMR4:71967001	4	71967001	1000	1	9.27E-05	-1.4368142	15	1.5		
DMR4:73788001	4	73788001	1000	1	2.56E-05	1.2430171	4	0.4		
DMR4:88719001	4	88719001	1000	1	4.51E-05	1.247866	4	0.4	HERC3;FAM13A-AS1;FAM13A	Proteolysis
DMR4:95191001	4	95191001	2000	1	2.06E-07	1.0936704	11	0.55	UNC5C	Receptor
DMR4:108868001	4	108868001	1000	1	5.10E-05	1.0217608	1	0.1	COL25A1	Extracellular Matrix
DMR4:137827001	4	137827001	2000	1	5.01E-07	1.4423657	18	0.9		
DMR4:140689001	4	140689001	1000	1	6.62E-05	1.1549118	7	0.7	TBC1D9	Signaling
DMR4:145417001	4	145417001	1000	1	8.81E-05	1.0486849	4	0.4		
DMR4:151731001	4	151731001	2000	1	9.79E-05	1.012617	79	3.95	GATB	Metabolism
DMR4:182831001	4	182831001	4000	1	8.39E-06	1.1808547	18	0.45		
DMR4:185743001	4	185743001	1000	1	2.37E-05	1.2910707	8	0.8	SORBS2	
DMR4:188151001	4	188151001	4000	2	2.58E-07	1.125732	9	0.225	TRIML1;LINC02434	Proteolysis
DMR5:3276001	5	3276001	3000	1	1.26E-08	1.1043358	36	1.2		
DMR5:5687001	5	5687001	1000	1	2.46E-05	-1.4771223	7	0.7	LOC105374634	
DMR5:6571001	5	6571001	2000	1	5.56E-06	0.9074854	40	2	LINC01018	
DMR5:26569001	5	26569001	2000	1	3.32E-06	1.407312	47	2.35		
DMR5:46926001	5	46926001	1000	1	1.37E-05	-1.0154739	12	1.2		
DMR5:47325001	5	47325001	2000	1	3.06E-05	-1.1360208	34	1.7		
DMR5:47629001	5	47629001	1000	1	6.52E-05	-1.1271792	17	1.7		
DMR5:68325001	5	68325001	1000	1	4.09E-05	1.4170889	2	0.2		
DMR5:68808001	5	68808001	1000	1	2.67E-05	1.4480184	1	0.1	LOC105379014	
DMR5:86239001	5	86239001	1000	1	7.56E-05	-1.1299105	14	1.4		
DMR5:115249001	5	115249001	1000	1	4.05E-05	1.664675	6	0.6	PGGT1B	Metabolism
DMR5:128811001	5	128811001	1000	1	6.69E-05	1.5548846	2	0.2	LOC105379168	
DMR5:148788001	5	148788001	1000	1	7.96E-05	1.4433404	11	1.1		
DMR5:148931001	5	148931001	1000	1	5.51E-05	1.2099086	3	0.3		
DMR5:149366001	5	149366001	1000	1	7.38E-05	1.2175148	14	1.4	GRPEL2-AS1;PCYOX1L;IL17B	Metabolism
DMR5:151537001	5	151537001	2000	1	1.97E-05	1.472744	18	0.9	SLC36A1;FAT2	Transport;Cytoskeleton
DMR5:158309001	5	158309001	2000	1	1.47E-05	1.2422994	8	0.4	LOC105377680;LINC02227	
DMR5:158851001	5	158851001	1000	1	2.60E-05	1.1569157	5	0.5	EBF1	Transcription
DMR5:174558001	5	174558001	1000	1	8.04E-07	1.1707713	2	0.2	LOC105377739;SUMO2P6	
DMR6:3316001	6	3316001	1000	1	7.80E-06	-1.6678217	20	2	PSMG4;SLC22A23	Transcription;Transport
DMR6:12921001	6	12921001	1000	1	1.11E-09	2.4594946	2	0.2	PHACTR1	Signaling
DMR6:18565001	6	18565001	2000	1	1.97E-05	0.9895397	5	0.25	MIR548A1HG;MIR548A1	
DMR6:22364001	6	22364001	1000	1	6.01E-05	1.5576452	13	1.3	LOC105374971	
DMR6:37057001	6	37057001	1000	1	3.59E-05	1.1899689	13	1.3		
DMR6:43787001	6	43787001	1000	1	2.73E-05	1.2890329	11	1.1	POLR1C;VEGFA;LOC105375070	Transcription;Growth Factors
DMR6:46525001	6	46525001	1000	1	3.44E-05	1.728231	4	0.4	LOC101926898	
DMR6:59818001	6	59818001	1000	1	6.78E-05	-0.8416338	13	1.3		
DMR6:81261001	6	81261001	1000	1	6.87E-07	1.5789558	1	0.1		
DMR6:87137001	6	87137001	1000	1	6.40E-06	1.7360455	2	0.2		
DMR6:132600001	6	132600001	2000	2	8.23E-06	1.254214	4	0.2	TAAR5;TAAR4P;TAAR3P	Signaling

DMR6:161453001	6	161453001	1000	1	5.54E-06	1.7041196	12	1.2	PRKN	Proteolysis
DMR6:166392001	6	166392001	2000	1	3.04E-05	-1.3903571	80	4	MPC1	
DMR7:76001	7	76001	1000	1	6.52E-05	1.0112031	20	2	LOC105375113;LOC101929756	
DMR7:1059001	7	1059001	1000	1	1.38E-05	1.0783672	54	5.4	C7orf50;GPR146;LOC107986755	Signaling
DMR7:1526001	7	1526001	1000	1	1.02E-08	1.7168917	10	1	MAFK;LOC100128653	Transcription
DMR7:5548001	7	5548001	3000	1	8.66E-06	1.1905098	53	1.766667	LOC100288712	
DMR7:15184001	7	15184001	2000	1	1.06E-05	1.0454558	10	0.5	AGMO	
DMR7:41522001	7	41522001	1000	1	8.93E-05	1.1687098	5	0.5		
DMR7:44174001	7	44174001	1000	1	2.25E-08	1.639888	3	0.3	GCK;LOC105375257	Signaling
DMR7:58497001	7	58497001	1000	1	7.09E-05	-1.0719644	14	1.4		
DMR7:59303001	7	59303001	3000	1	8.46E-06	-1.5576883	50	1.666667		
DMR7:59487001	7	59487001	1000	1	7.06E-05	-1.4540946	16	1.6		
DMR7:60093001	7	60093001	1000	1	1.83E-05	-1.2673791	16	1.6		
DMR7:60754001	7	60754001	1000	1	8.38E-05	-1.3059035	16	1.6		
DMR7:65113001	7	65113001	1000	1	2.15E-05	0.9201454	4	0.4		
DMR7:66865001	7	66865001	1000	1	6.93E-05	1.1673591	1	0.1	GTF2IP23	
DMR7:72106001	7	72106001	1000	1	3.83E-05	0.8188375	13	1.3	CALN1;ABCF2P2	
DMR7:84817001	7	84817001	1000	1	3.76E-05	1.5506787	4	0.4		
DMR7:98623001	7	98623001	1000	1	3.59E-05	-1.5577671	14	1.4	NPTX2	
DMR7:99142001	7	99142001	2000	1	3.99E-05	0.9452965	122	6.1	SMURF1;KPNA7	Proteolysis;Transport
DMR7:100487001	7	100487001	2000	1	4.70E-05	0.7802468	70	3.5	TSC22D4;NYAP1	Cytoskeleton
DMR7:100589001	7	100589001	2000	1	5.80E-05	1.1474959	35	1.75	LRCH4;FBXO24;PCOLCE-AS1	
DMR7:101463001	7	101463001	1000	1	8.43E-05	1.0140566	2	0.2	COL26A1	
DMR7:102430001	7	102430001	1000	1	7.25E-06	1.2407335	14	1.4	LOC100630923;PRKRIP1;LOC105375433;ORAI2	Transport
DMR7:102512001	7	102512001	1000	1	3.42E-05	1.1083053	22	2.2	RASA4B	Signaling
DMR7:102611001	7	102611001	1000	1	4.46E-06	1.2883471	31	3.1	RASA4	Signaling
DMR7:113240001	7	113240001	1000	1	3.61E-05	1.1886464	3	0.3		
DMR7:132561001	7	132561001	4000	3	6.07E-06	1.2458933	16	0.4	PLXNA4	
DMR7:139865001	7	139865001	1000	1	1.76E-05	1.1462989	6	0.6	TBXAS1	Metabolism
DMR7:149772001	7	149772001	1000	1	1.25E-08	1.868421	65	6.5	ZNF467;SSPOP	Extracellular Matrix
DMR7:149829001	7	149829001	2000	1	4.93E-09	1.2719414	9	0.45	SSPOP;ZNF862	Extracellular Matrix
DMR7:151056001	7	151056001	2000	1	6.86E-05	1.4672439	71	3.55	ABCB8;ASIC3;CDK5;SLC4A2	Transport;Transport;Signaling;Transport
DMR7:157776001	7	157776001	2000	1	2.38E-05	0.8996703	23	1.15	PTPRN2	Signaling
DMR7:158717001	7	158717001	1000	1	9.61E-08	1.3719449	0	0	RPL21P76	
DMR8:2191001	8	2191001	1000	1	7.21E-06	1.2035495	2	0.2	LOC105377781;LOC105377782	
DMR8:19450001	8	19450001	1000	1	1.23E-06	1.767291	7	0.7	CSGALNACT1	Golgi
DMR8:21043001	8	21043001	1000	1	3.77E-05	0.934924	5	0.5		
DMR8:23523001	8	23523001	1000	1	4.83E-05	1.3389185	30	3	SLC25A37	
DMR8:27918001	8	27918001	2000	1	4.88E-05	1.1836801	10	0.5	SCARA5;LOC105379342	Protease
DMR8:36387001	8	36387001	2000	1	9.91E-06	1.7338244	10	0.5		
DMR8:37997001	8	37997001	1000	1	8.70E-07	1.2553246	19	1.9		
DMR8:57399001	8	57399001	1000	1	4.28E-05	1.0370096	1	0.1	RPL30P10	
DMR8:89867001	8	89867001	1000	1	3.83E-05	1.107166	24	2.4		
DMR8:105533001	8	105533001	2000	1	1.05E-05	1.1858105	6	0.3	ZFPM2;LOC105375696	Transcription
DMR8:116175001	8	116175001	2000	1	3.06E-06	1.0495164	8	0.4	LINC00536	
DMR8:132059001	8	132059001	1000	1	1.70E-05	1.5470577	4	0.4	OC90;HHLA1	Metabolism
DMR8:134243001	8	134243001	1000	1	7.23E-06	1.1118962	1	0.1		
DMR8:139769001	8	139769001	1000	1	8.97E-05	-1.2597127	13	1.3	TRAPPC9	
DMR8:142191001	8	142191001	3000	1	5.33E-05	1.323755	49	1.633333	LOC105375792;LINC00051	
DMR9:5111001	9	5111001	1000	1	8.76E-05	0.8229697	68	6.8	JAK2;INSL6;MTCO3P11;MTND3P14;MTND4LP6;MTND4P14;MTND5P14;TCF3P1;LOC107987044;IGHEP2	Hormone
DMR9:7476001	9	7476001	1000	1	3.77E-05	-1.4665724	9	0.9	RPL4P5	
DMR9:15126001	9	15126001	1000	1	2.45E-05	0.9901397	12	1.2		
DMR9:37188001	9	37188001	1000	1	3.77E-05	1.4997753	4	0.4	ZCCHC7;LOC105376033	
DMR9:37378001	9	37378001	1000	1	7.55E-05	1.0444094	28	2.8	LOC105376035;LINC01627	
DMR9:60577001	9	60577001	12000	1	6.89E-05	-1.0131413	164	1.366667		
DMR9:60623001	9	60623001	15000	2	7.82E-06	-0.8652991	190	1.266667		
DMR9:60663001	9	60663001	2000	1	1.39E-05	-1.1044879	23	1.15		
DMR9:60686001	9	60686001	2000	1	3.85E-05	-0.8443258	24	1.2		
DMR9:65420001	9	65420001	1000	1	9.05E-06	1.0131284	7	0.7		
DMR9:88534001	9	88534001	1000	1	7.56E-05	1.1370347	84	8.4	LOC105376134;NXNL2	Metabolism
DMR9:93818001	9	93818001	2000	1	5.35E-05	1.0441094	23	1.15	MIR4291	
DMR9:107638001	9	107638001	1000	1	5.81E-06	2.0720533	32	3.2	LOC105376205	
DMR9:113687001	9	113687001	1000	1	1.59E-09	2.0773776	29	2.9	LOC105376223	

DMR9:114339001	9	114339001	1000	1	6.24E-05	1.2741197	23	2.3	ORM2;AKNA	
DMR9:115144001	9	115144001	1000	1	2.64E-07	1.4889875	4	0.4	LOC101928748;DELEC1	
DMR9:124829001	9	124829001	1000	1	2.07E-05	1.1402883	10	1		
DMR9:127409001	9	127409001	1000	1	3.16E-05	0.9287786	12	1.2	LOC112268056;SLC2A8	
DMR9:130307001	9	130307001	1000	1	8.60E-05	0.9110093	23	2.3	HMCN2	Receptor
DMR9:134024001	9	134024001	2000	1	5.52E-05	1.4170413	93	4.65	BRD3OS;BRD3	
DMR9:134444001	9	134444001	1000	1	3.02E-05	1.5381812	6	0.6	RXRA	Transcription
DMR9:135990001	9	135990001	1000	1	2.64E-05	1.1009243	38	3.8		
DMR9:136865001	9	136865001	1000	1	5.21E-05	1.2072651	22	2.2	MAMDC4;EDF1	
DMR9:138203001	9	138203001	1000	1	9.47E-06	0.8726769	19	1.9	LOC101928932	
DMR10:6226001	10	6226001	1000	1	6.61E-05	0.7909026	43	4.3	PFKFB3	Metabolism
DMR10:11795001	10	11795001	1000	1	5.38E-05	0.7920126	26	2.6	LOC105376415	
DMR10:17337001	10	17337001	1000	1	3.47E-05	-1.5378838	15	1.5	ST8SIA6	Transport
DMR10:17519001	10	17519001	1000	1	1.91E-05	1.6423527	8	0.8		
DMR10:21089001	10	21089001	2000	1	4.38E-05	1.0966487	21	1.05	NEBL	
DMR10:23286001	10	23286001	1000	1	7.60E-06	1.5532843	2	0.2	C10orf67	
DMR10:34462001	10	34462001	2000	1	6.99E-07	1.3978148	10	0.5	PARD3	
DMR10:36247001	10	36247001	1000	1	5.29E-06	1.4934948	3	0.3		
DMR10:40658001	10	40658001	1000	1	8.07E-05	-1.0569374	16	1.6		
DMR10:41405001	10	41405001	4000	1	8.09E-05	-1.3511011	63	1.575		
DMR10:55538001	10	55538001	1000	1	1.28E-05	1.1411058	5	0.5	PCDH15	Cytoskeleton
DMR10:58481001	10	58481001	1000	1	8.43E-05	1.5563304	4	0.4	LOC105378316	
DMR10:69273001	10	69273001	1000	1	6.63E-05	-1.2586319	14	1.4	HKDC1;HK1	Signaling
DMR10:72492001	10	72492001	1000	1	4.11E-05	0.93983	19	1.9	MICU1;SNX19P4	Signaling
DMR10:78613001	10	78613001	1000	1	1.98E-07	1.6888653	7	0.7		
DMR10:89142001	10	89142001	1000	1	1.53E-07	1.7212315	3	0.3	LOC100289238	
DMR10:94141001	10	94141001	1000	1	4.30E-06	1.7438312	3	0.3	PLCE1;HDAC1P1	Metabolism
DMR10:97251001	10	97251001	1000	1	1.30E-08	1.4015158	1	0.1	ARHGAP19-SLIT1;ARHGAP19	Signaling
DMR10:97713001	10	97713001	1000	1	5.61E-05	1.5271906	80	8	MARVELD1	Transport
DMR10:112455001	10	112455001	1000	1	8.90E-09	2.2867127	5	0.5	ZDHHC6;VTI1A	Transcription
DMR10:119063001	10	119063001	1000	1	4.00E-05	1.4549664	17	1.7	EIF3A;SNORA19	Translation
DMR10:119291001	10	119291001	2000	2	1.34E-07	1.6757251	13	0.65	GRK5	Signaling
DMR10:119953001	10	119953001	1000	1	6.96E-05	1.3663523	2	0.2	SEC23IP;MIR4682	Metabolism
DMR10:129564001	10	129564001	1000	1	1.54E-06	1.3068098	8	0.8	MGMT	
DMR10:131346001	10	131346001	2000	1	5.57E-05	1.0150971	16	0.8		
DMR10:132545001	10	132545001	2000	1	3.32E-05	1.0053216	76	3.8	LOC107984282;INPP5A	Signaling
DMR10:132603001	10	132603001	3000	1	8.95E-05	0.9369152	56	1.866667	INPP5A	Signaling
DMR11:316001	11	316001	1000	1	8.42E-05	1.5773832	75	7.5	IFITM2;IFITM1;IFITM3;LOC105376504	
DMR11:391001	11	391001	2000	1	7.71E-07	1.9980067	91	4.55	B4GALNT4;PKP3	Golgi;Cytoskeleton
DMR11:2379001	11	2379001	1000	1	1.73E-05	1.1695365	17	1.7	CD81-AS1;LOC105376520;CD81	
DMR11:15854001	11	15854001	1000	1	6.79E-07	1.6338496	0	0	LOC107984370	
DMR11:30092001	11	30092001	1000	1	2.13E-07	1.6857781	2	0.2	ARL14EP-DT	
DMR11:34405001	11	34405001	1000	1	1.43E-05	0.942802	7	0.7		
DMR11:77490001	11	77490001	1000	1	3.32E-05	0.8618087	56	5.6	PAK1	Signaling
DMR11:87755001	11	87755001	1000	1	3.89E-05	1.6270115	1	0.1	LOC107984361	
DMR11:106123001	11	106123001	1000	1	9.74E-05	-1.4650655	6	0.6	LINC02719	
DMR11:109841001	11	109841001	1000	1	1.37E-05	1.0367894	3	0.3		
DMR11:112196001	11	112196001	1000	1	5.63E-07	2.2208805	2	0.2	BCO2	Metabolism
DMR11:113195001	11	113195001	1000	1	8.13E-05	-1.2648362	12	1.2	NCAM1	
DMR11:133706001	11	133706001	2000	1	6.90E-08	1.3354959	9	0.45		
DMR12:1661001	12	1661001	2000	1	1.36E-07	1.5406629	142	7.1	MIR3649	
DMR12:2572001	12	2572001	1000	1	1.27E-05	0.9725348	2	0.2	CACNA1C	Transport
DMR12:6375001	12	6375001	1000	1	4.42E-06	1.6985806	19	1.9	SCNN1A;LOC105369626;LTBR	Transport;Receptor
DMR12:26565001	12	26565001	2000	1	2.37E-05	1.0325794	4	0.2	ITPR2	Ion Channel
DMR12:34995001	12	34995001	1000	1	7.53E-06	-1.2152939	14	1.4		
DMR12:36939001	12	36939001	1000	1	2.23E-05	-1.3287738	14	1.4		
DMR12:37007001	12	37007001	2000	1	4.37E-05	-1.0327526	26	1.3		
DMR12:37092001	12	37092001	1000	1	2.77E-05	-1.0359176	14	1.4		
DMR12:52213001	12	52213001	1000	1	2.58E-05	1.0638258	17	1.7	LINC02874;LINC00592	
DMR12:65133001	12	65133001	2000	1	1.14E-05	1.487696	5	0.25	APOOP3	
DMR12:97988001	12	97988001	2000	1	2.47E-05	1.0926717	7	0.35	MIR4303	
DMR12:102973001	12	102973001	1000	1	4.89E-05	0.9778434	2	0.2		
DMR12:110820001	12	110820001	1000	1	2.74E-06	1.1321712	8	0.8		
DMR12:111257001	12	111257001	1000	1	9.74E-08	1.3767985	6	0.6	CUX2	Development
DMR12:112574001	12	112574001	1000	1	5.40E-05	0.833677	3	0.3	RPH3A	
DMR12:113045001	12	113045001	1000	1	2.08E-05	1.2491646	2	0.2	RPS15AP32	

DMR12:116737001	12	116737001	1000	1	9.98E-06	1.6376953	51	5.1	SPRING1;RNFT2	
DMR12:117026001	12	117026001	2000	1	8.43E-05	1.0276012	57	2.85	FBXW8	
DMR12:124540001	12	124540001	2000	1	7.04E-05	0.9636778	7	0.35	NCOR2	Epigenetic
DMR12:132304001	12	132304001	4000	1	6.89E-05	0.7633562	205	5.125	GALNT9	Golgi
DMR13:17246001	13	17246001	1000	1	6.65E-05	-1.2252314	16	1.6		
DMR13:17620001	13	17620001	4000	1	5.85E-05	-1.2230957	74	1.85		
DMR13:27925001	13	27925001	1000	1	1.86E-05	1.3663179	15	1.5	PLUT;PDX1	
DMR13:76828001	13	76828001	1000	1	2.50E-10	2.3113146	3	0.3	LOC107984587	
DMR13:80420001	13	80420001	1000	1	1.00E-06	1.4091993	1	0.1		
DMR13:105992001	13	105992001	1000	1	8.29E-05	1.1662834	5	0.5		
DMR13:112802001	13	112802001	4000	1	1.26E-05	1.4727086	57	1.425	ATP11A	Transport
DMR13:112881001	13	112881001	3000	1	4.54E-05	0.8276708	129	4.3	ATP11A	Transport
DMR14:16834001	14	16834001	1000	1	7.57E-06	-1.5653951	17	1.7		
DMR14:76539001	14	76539001	1000	1	1.03E-05	1.6295984	5	0.5		
DMR14:95137001	14	95137001	1000	1	8.45E-07	1.5362567	4	0.4	DICER1;MIR3173	
DMR14:95374001	14	95374001	2000	1	7.84E-05	1.2003347	11	0.55	LOC107984710	
DMR14:101782001	14	101782001	2000	1	2.06E-05	1.2576505	13	0.65	PPP2R5C	Signaling
DMR14:105597001	14	105597001	1000	1	6.55E-06	0.9459991	44	4.4	IGH;IGHA2;IGHE	Immune
DMR14:105678001	14	105678001	1000	1	7.05E-06	0.9277178	40	4	IGH;LOC112268138;IGHGP;ELK2AP	
DMR15:19788001	15	19788001	1000	1	4.84E-05	-1.3739536	10	1		
DMR15:40343001	15	40343001	1000	1	2.65E-06	1.6931502	16	1.6	CCDC9B;PHGR1	
DMR15:45666001	15	45666001	1000	1	1.17E-05	1.7415007	1	0.1	SQOR	Metabolism
DMR15:52119001	15	52119001	1000	1	1.57E-05	0.9348399	9	0.9	BCL2L10;GNB5	Signaling
DMR15:64702001	15	64702001	1000	1	1.69E-05	1.2478246	19	1.9	OAZ2	Signaling
DMR15:78763001	15	78763001	1000	1	9.29E-05	0.8989674	38	3.8	LOC646938;LOC112268143;ADAMTS7	Protease
DMR15:83950001	15	83950001	1000	1	6.42E-05	-1.6113924	5	0.5	ADAMTSL3;LOC105370935	Protease
DMR15:86701001	15	86701001	1000	1	7.17E-05	1.2293147	1	0.1	AGBL1	Protease
DMR16:4182001	16	4182001	1000	1	2.69E-05	0.9390926	44	4.4	LOC105371062;SRL	Transport
DMR16:5422001	16	5422001	1000	1	3.56E-05	0.9721442	7	0.7	RBFOX1	Translation
DMR16:5616001	16	5616001	1000	1	4.66E-07	1.4084118	23	2.3	RBFOX1;LINC01570	Translation
DMR16:13126001	16	13126001	2000	1	7.52E-06	1.1062377	9	0.45	SHISA9	
DMR16:13264001	16	13264001	2000	1	3.22E-06	1.0416797	14	0.7	SHISA9;LOC107984137	
DMR16:13854001	16	13854001	1000	1	8.47E-05	1.1572485	7	0.7		
DMR16:15850001	16	15850001	1000	1	8.98E-05	1.2614393	14	1.4	MYH11	
DMR16:23981001	16	23981001	2000	2	1.04E-08	1.3101244	14	0.7	PRKCB	Signaling
DMR16:49477001	16	49477001	1000	1	9.70E-06	1.1548284	2	0.2	LINC02179;ZNF423	Transcription
DMR16:54684001	16	54684001	2000	1	7.36E-10	2.2725152	16	0.8		
DMR16:63455001	16	63455001	1000	1	7.52E-05	1.0255496	4	0.4	LOC105371308	
DMR16:64561001	16	64561001	1000	1	5.10E-05	1.492595	4	0.4		
DMR16:66087001	16	66087001	1000	1	5.32E-05	1.2163323	1	0.1		
DMR16:80724001	16	80724001	1000	1	1.13E-05	1.1976218	1	0.1	CDYL2	
DMR16:84618001	16	84618001	2000	1	3.96E-07	1.8446711	65	3.25	COTL1	Cytoskeleton
DMR16:85716001	16	85716001	1000	1	6.17E-05	1.3634919	13	1.3	C16orf74	
DMR16:86603001	16	86603001	1000	1	9.15E-05	0.8938171	8	0.8		
DMR16:87491001	16	87491001	1000	1	2.05E-05	1.0170997	75	7.5	ZCCHC14;ZCCHC14-DT	
DMR16:88207001	16	88207001	1000	1	5.45E-05	0.7562426	66	6.6		
DMR16:89192001	16	89192001	2000	1	7.06E-06	0.9603445	133	6.65	CDH15;SLC22A31;ZNF778	Cytoskeleton;Transport;Transcription
DMR17:242001	17	242001	2000	1	8.20E-07	1.1179605	1	0.05	RPH3AL	
DMR17:344001	17	344001	1000	1	1.91E-05	1.4596077	6	0.6	RPH3AL;LOC105371425	
DMR17:3890001	17	3890001	1000	1	3.49E-05	1.3915156	28	2.8	CAMKK1;P2RX1	Signaling;Ion Channel
DMR17:6560001	17	6560001	3000	1	8.88E-07	1.2840112	23	0.766667	PITPNM3	Metabolism
DMR17:7549001	17	7549001	1000	1	3.56E-05	1.0496973	57	5.7	TNFSF12-TNFSF13;TNFSF12;TNFSF13	
DMR17:8828001	17	8828001	1000	1	8.87E-05	0.9097404	46	4.6	PIK3R6	Signaling
DMR17:18410001	17	18410001	2000	1	1.37E-07	1.8941065	60	3	TBC1D3P4;LINC02076;YWHAEP2	
DMR17:21004001	17	21004001	1000	1	6.14E-05	1.41517	27	2.7	USP22	Protease
DMR17:40474001	17	40474001	1000	1	9.44E-06	1.3326506	19	1.9	LOC107985064;TNS4	Cytoskeleton
DMR17:57131001	17	57131001	2000	1	5.98E-06	0.9468673	39	1.95	AKAP1	Cytoskeleton
DMR17:63993001	17	63993001	1000	1	7.99E-06	1.1948396	4	0.4	PRR29-AS1;PRR29;ICAM2	
DMR17:66968001	17	66968001	1000	1	2.93E-05	1.0915067	8	0.8	CACNG4	Transport
DMR17:69765001	17	69765001	1000	1	1.84E-06	1.1883059	4	0.4	LINC01483	
DMR17:76130001	17	76130001	2000	1	1.62E-05	0.9365612	48	2.4	LOC101928447;FOXJ1;RNF157-AS1	
DMR17:76700001	17	76700001	2000	1	8.81E-06	1.4214091	60	3	MXRA7;RNY4P36	
DMR17:79004001	17	79004001	2000	1	6.52E-06	1.0097815	15	0.75	CANT1	Signaling
DMR17:79332001	17	79332001	2000	1	4.00E-05	1.1079058	21	1.05	RBFOX3	Translation
DMR17:82187001	17	82187001	1000	1	3.31E-05	0.771438	34	3.4	CCDC57	
DMR17:82359001	17	82359001	3000	1	4.08E-08	1.3934834	10	0.333333	TEX19;UTS2R	Signaling

DMR17:82424001	17	82424001	2000	1	3.27E-07	1.1102652	37	1.85	OGFOD3;HEXD;HEXD-IT1	Golgi
DMR18:23410001	18	23410001	2000	1	7.37E-05	1.1217911	30	1.5	TMEM241	Transport
DMR18:26593001	18	26593001	1000	1	3.46E-06	1.1777359	4	0.4	KCTD1;MIR8057;CIAPIN1P	Cytoskeleton
DMR18:32782001	18	32782001	1000	1	6.41E-07	1.6290664	1	0.1	KLHL14;LOC105372055;RPS27P28	
DMR18:38565001	18	38565001	1000	1	6.56E-06	1.3703862	2	0.2	LOC112268212	
DMR18:47982001	18	47982001	1000	1	4.45E-05	1.608221	6	0.6		
DMR18:48107001	18	48107001	1000	1	7.03E-06	1.3447277	12	1.2	ZBTB7C	
DMR18:49848001	18	49848001	1000	1	3.64E-05	1.4243071	8	0.8	SNHG22;MYO5B	Cytoskeleton
DMR18:56270001	18	56270001	1000	1	4.75E-06	1.5585645	1	0.1		
DMR18:69894001	18	69894001	1000	1	3.82E-05	1.697905	2	0.2	CD226	
DMR18:78228001	18	78228001	2000	1	3.23E-05	1.1290342	39	1.95		
DMR19:1115001	19	1115001	3000	1	1.03E-05	1.6181513	122	4.066667	GPX4;SBNO2	Metabolism
DMR19:1148001	19	1148001	2000	1	5.29E-06	0.8679053	69	3.45	SBNO2	
DMR19:1247001	19	1247001	1000	1	4.34E-05	1.3824779	37	3.7	CBARP;ATP5F1D;LOC102723811;MIDN	Transport;Metabolism
DMR19:1282001	19	1282001	1000	1	5.64E-05	0.8572212	9	0.9	CIRBP;FAM174C;EFNA2	Signaling
DMR19:1669001	19	1669001	1000	1	1.75E-05	0.9531915	14	1.4		
DMR19:1746001	19	1746001	1000	1	1.56E-07	2.1590041	22	2.2	ONECUT3	Development
DMR19:2056001	19	2056001	1000	1	1.61E-06	1.9439661	24	2.4	MKNK2	Signaling
DMR19:2639001	19	2639001	2000	1	2.33E-05	1.2116071	35	1.75	GNG7;MIR7850	Signaling
DMR19:3387001	19	3387001	1000	1	4.91E-05	1.2829325	34	3.4	NFIC	Transcription
DMR19:3577001	19	3577001	2000	1	1.36E-07	1.0507825	160	8	HMG20B;GIPC3	Cytoskeleton
DMR19:4143001	19	4143001	3000	1	2.87E-06	1.4259085	47	1.566667	CREB3L3	
DMR19:6533001	19	6533001	2000	1	7.16E-05	1.1033026	52	2.6	TNFSF9	
DMR19:9792001	19	9792001	1000	1	8.98E-05	1.3520995	82	8.2	ZNF846;LOC100505555	Transcription
DMR19:12998001	19	12998001	1000	1	2.00E-05	1.3297049	36	3.6	NFIX	Transcription
DMR19:13025001	19	13025001	1000	1	5.88E-06	1.9141494	46	4.6	NFIX	Transcription
DMR19:13208001	19	13208001	1000	1	4.94E-05	0.8643444	54	5.4	LOC107985288;CACNA1A	Transport
DMR19:13241001	19	13241001	1000	1	1.83E-05	0.8976953	27	2.7	CACNA1A	Transport
DMR19:14476001	19	14476001	1000	1	3.44E-05	0.787332	26	2.6	PKN1;PTGER1;GIPC1	Signaling;Signaling;Cytoskeleton
DMR19:16777001	19	16777001	1000	1	3.35E-08	1.354203	0	0	NWD1	
DMR19:16948001	19	16948001	2000	1	3.96E-05	1.270589	21	1.05	CPAMD8	Protease; Proteolysis
DMR19:17830001	19	17830001	2000	2	2.00E-05	1.2782043	106	5.3	INSL3;JAK3	
DMR19:18790001	19	18790001	2000	1	3.10E-05	1.8029381	140	7	CRTC1;COMP;LOC107985321	Transcription
DMR19:19239001	19	19239001	2000	1	9.55E-05	1.0017175	21	1.05	NCAN;RNU6-1028P	Extracellular Matrix
DMR19:23465001	19	23465001	5000	1	1.56E-05	0.8928964	24	0.48	LOC105372334	
DMR19:23878001	19	23878001	5000	1	3.27E-06	0.9266857	25	0.5	RPSAP58	
DMR19:25644001	19	25644001	1000	1	3.18E-06	-1.4745921	20	2		
DMR19:26019001	19	26019001	1000	1	2.94E-05	-1.5199676	17	1.7		
DMR19:28282001	19	28282001	4000	2	6.63E-05	0.9877658	14	0.35		
DMR19:29511001	19	29511001	4000	1	1.52E-06	1.1785604	27	0.675	VSTM2B-DT	
DMR19:32392001	19	32392001	1000	1	7.42E-05	1.0913827	7	0.7	ZNF507;DPY19L3-DT	Transcription
DMR19:38648001	19	38648001	1000	1	4.25E-05	0.9838342	23	2.3	ACTN4	
DMR19:41913001	19	41913001	3000	1	7.88E-06	0.9210792	124	4.133333	ARHGEF1;ERFL	Transcription;Transcription
DMR19:41921001	19	41921001	1000	1	3.29E-05	1.4281148	36	3.6	ERFL;LOC100505585	Transcription
DMR19:44684001	19	44684001	1000	1	7.02E-07	1.3822445	12	1.2	CEACAM16-AS1;CEACAM19;LOC107985306	
DMR19:48398001	19	48398001	1000	1	1.86E-05	1.1196754	116	11.6	KDELR1;LOC107984140;GRIN2D	Transport;Receptor
DMR19:49338001	19	49338001	3000	1	2.01E-11	2.3194165	150	5	SLC6A16;CD37;TEAD2	Transport;Transcription
DMR19:50978001	19	50978001	4000	1	5.47E-09	1.3583036	46	1.15	KLK6;LOC105372442;KLK7	Protease
DMR19:51691001	19	51691001	1000	1	3.90E-05	1.0363269	22	2.2	SPACA6P-AS;SPACA6;MIR99B;MIRLET7E;MIR125A	
DMR19:57963001	19	57963001	1000	1	2.76E-05	-1.0468709	24	2.4	RPL19P19;C19orf18	
DMR19:58326001	19	58326001	1000	1	3.55E-05	1.293289	22	2.2	LOC105372480;LOC100887072;ZSCAN22;MIR6806	Transcription
DMR20:1818001	20	1818001	1000	1	3.69E-05	0.7412836	8	0.8	LOC107984104;LOC105372501	
DMR20:1829001	20	1829001	1000	1	7.71E-05	1.434196	14	1.4	LOC107984104;LOC105372501	
DMR20:6657001	20	6657001	1000	1	2.83E-05	1.6515301	6	0.6		
DMR20:11275001	20	11275001	3000	1	6.19E-05	0.8240951	20	0.666667	LOC339593	
DMR20:11864001	20	11864001	1000	1	8.72E-05	-1.2055948	5	0.5	LINC00687	
DMR20:26936001	20	26936001	2000	1	5.15E-05	-1.0897064	32	1.6		
DMR20:27599001	20	27599001	1000	1	7.20E-06	-1.1734587	10	1		
DMR20:34873001	20	34873001	1000	1	1.49E-06	1.4982938	22	2.2	GGT7;ACSS2	Protease;Metabolism
DMR20:40959001	20	40959001	1000	1	1.33E-05	1.1368291	10	1		
DMR20:42958001	20	42958001	1000	1	8.40E-06	1.5991858	4	0.4	PTPRT;LOC105372623;PTPRT-AS1	Signaling
DMR20:47844001	20	47844001	1000	1	2.36E-07	1.9061608	3	0.3	LOC105372636	
DMR20:48920001	20	48920001	2000	1	3.26E-05	1.1043051	89	4.45	ARFGEF2	Transcription

DMR20:52610001	20	52610001	2000	1	3.84E-05	1.2488861	6	0.3	LOC105372666;TRI-AAT12-1;LOC105372665;LINC01524	
DMR20:53072001	20	53072001	2000	1	8.64E-05	0.9088598	10	0.5	TSHZ2	Transcription
DMR20:56710001	20	56710001	2000	1	9.61E-05	0.7884777	28	1.4	PTMAP6	
DMR20:57928001	20	57928001	1000	1	1.01E-06	1.5853523	2	0.2		
DMR20:62451001	20	62451001	2000	1	8.04E-05	1.0187947	32	1.6	LOC105372710	
DMR20:64157001	20	64157001	2000	1	3.40E-06	1.1110763	29	1.45	MYT1	Transcription
DMR21:12394001	21	12394001	1000	1	8.81E-07	-1.4639017	13	1.3		
DMR21:28225001	21	28225001	1000	1	6.82E-05	1.4650888	2	0.2	LINC01695	
DMR21:36496001	21	36496001	2000	1	3.09E-06	0.9656082	25	1.25	LOC105369301;CLDN14;PSMD4P1	Cell Junction
DMR21:37712001	21	37712001	1000	1	1.23E-07	1.5511273	4	0.4	KCNJ6;LOC107985507;KCNJ6-AS1	Transport
DMR21:39020001	21	39020001	3000	1	4.76E-05	1.2965504	67	2.233333	LOC102724740;LOC105372803;LOC107985484	
DMR21:42586001	21	42586001	5000	1	7.95E-05	0.884804	329	6.58	SLC37A1;LINC01671	Transport
DMR21:43280001	21	43280001	1000	1	5.36E-05	1.015324	4	0.4		
DMR21:44395001	21	44395001	2000	1	6.56E-06	1.1796079	15	0.75	TRPM2	Transport
DMR22:15038001	22	15038001	4000	1	6.01E-05	-1.1724117	72	1.8		
DMR22:33561001	22	33561001	1000	1	4.37E-05	1.0341267	18	1.8	LARGE1	Golgi
DMR22:36126001	22	36126001	1000	1	1.12E-06	1.2509965	1	0.1	LOC102723507	
DMR22:37374001	22	37374001	2000	1	3.13E-05	0.8482812	128	6.4	ELFN2	Receptor
DMR22:37418001	22	37418001	1000	1	3.39E-05	1.2432818	22	2.2	ELFN2	Receptor
DMR22:44575001	22	44575001	1000	1	5.79E-05	1.3461762	12	1.2	KRT18P23;LINC00207	
DMR22:48528001	22	48528001	2000	1	7.52E-05	1.2019284	31	1.55	TAFA5;LOC284933	Growth Factors
DMR22:50083001	22	50083001	1000	1	8.49E-05	1.4765776	37	3.7	MLC1;MOV10L1	
DMRX:1168001	X	1168001	2000	1	2.71E-05	1.1792464	32	1.6	LOC652608	
DMRX:1514001	X	1514001	1000	1	3.33E-07	1.3415139	5	0.5	P2RY8	Signaling
DMRX:1588001	X	1588001	1000	1	6.95E-07	1.5194337	4	0.4	AKAP17A;ASMT	Epigenetic
DMRX:2213001	X	2213001	2000	2	2.38E-05	1.1099269	14	0.7	DHRSX	Metabolism
DMRX:2610001	X	2610001	2000	1	1.47E-06	1.2071753	29	1.45	LOC101928092;MIR6089;CD99P1;LINC00102	
DMRX:3328001	X	3328001	2000	1	7.54E-05	0.854447	17	0.85	MXRA5	
DMRX:5532001	X	5532001	1000	1	4.02E-07	1.691798	3	0.3		
DMRX:9273001	X	9273001	2000	1	7.21E-05	1.1180245	20	1		
DMRX:17245001	X	17245001	1000	1	3.79E-05	0.9968243	51	5.1		
DMRX:17718001	X	17718001	2000	1	1.51E-05	1.2610537	11	0.55	NHS	
DMRX:30553001	X	30553001	1000	1	5.79E-05	0.9987854	25	2.5	TASL	
DMRX:35548001	X	35548001	1000	1	2.26E-05	1.435874	9	0.9		
DMRX:38286001	X	38286001	1000	1	4.43E-06	1.3049445	2	0.2	RPGR	Proteolysis
DMRX:39398001	X	39398001	1000	1	2.44E-05	0.8831156	12	1.2	LINC01282;LINC01283	
DMRX:39908001	X	39908001	1000	1	1.39E-06	1.6874698	22	2.2		
DMRX:40103001	X	40103001	2000	1	8.22E-05	1.1263814	101	5.05	BCOR;LOC107985687	
DMRX:47219001	X	47219001	2000	1	6.50E-05	1.6727217	66	3.3	UBA1;LOC105373194;CDK16	Proteolysis;Signaling
DMRX:48802001	X	48802001	1000	1	6.96E-07	2.1163783	48	4.8	GATA1;HDAC6	Transcription
DMRX:49175001	X	49175001	2000	2	6.85E-06	1.7439005	61	3.05	MAGIX;PLP2;PRICKLE3	Transport;Cytoskeleton
DMRX:49269001	X	49269001	1000	1	4.82E-06	1.9610628	82	8.2	FOXP3;FLICR;PPP1R3F	Signaling
DMRX:51223001	X	51223001	1000	1	3.42E-05	2.0137265	10	1		
DMRX:51893001	X	51893001	2000	1	1.57E-09	2.5295357	93	4.65	MAGED1	Cytoskeleton
DMRX:52067001	X	52067001	2000	1	5.28E-06	1.9561905	93	4.65	LOC105377208;MAGED4B;SNORA11E	Cytoskeleton
DMRX:52185001	X	52185001	1000	1	2.06E-07	2.3838441	48	4.8	MAGED4;SNORA11D;LOC105377209	
DMRX:52199001	X	52199001	2000	1	7.21E-06	1.2306588	30	1.5	MAGED4;SNORA11D;LOC105377209	
DMRX:53080001	X	53080001	4000	1	9.11E-05	1.5706782	154	3.85	GPR173;TSPYL2	Signaling;Epigenetic
DMRX:53279001	X	53279001	2000	1	7.35E-05	1.4895308	31	1.55	IQSEC2	Transcription
DMRX:53721001	X	53721001	1000	1	3.02E-05	0.902591	59	5.9		
DMRX:55000001	X	55000001	2000	2	7.12E-06	1.4299467	64	3.2	PFKFB1;APEX2;ALAS2	Metabolism;Metabolism
DMRX:55747001	X	55747001	1000	1	2.18E-06	1.5421844	13	1.3	RRAGB	Signaling
DMRX:68990001	X	68990001	2000	1	8.89E-05	1.3137463	18	0.9		
DMRX:71096001	X	71096001	1000	1	5.13E-05	1.7745689	53	5.3	FOXO4;CXorf65	
DMRX:74421001	X	74421001	1000	1	7.53E-05	1.9406193	63	6.3	LOC105373252;SLC16A2	Transport
DMRX:75210001	X	75210001	2000	1	9.45E-05	0.8256465	15	0.75	UPRT;BUD31P2;RNU6-562P	Signaling
DMRX:107776001	X	107776001	1000	1	1.02E-06	1.5533902	54	5.4	TSC22D3;NCBP2L	Metabolism
DMRX:118232001	X	118232001	1000	1	1.75E-05	1.761427	8	0.8		
DMRX:118495001	X	118495001	1000	1	4.50E-05	2.0451926	78	7.8	DOCK11	Transcription
DMRX:120251001	X	120251001	1000	1	1.08E-07	3.088409	62	6.2	NKAPP1;ZBTB33;TMEM255A	Transcription
DMRX:123732001	X	123732001	1000	1	2.09E-06	2.9328897	65	6.5	THOC2	Transcription
DMRX:129931001	X	129931001	4000	1	9.98E-07	2.4525139	242	6.05	UTP14A;LOC105373335	Metabolism
DMRX:129980001	X	129980001	5000	2	3.62E-06	2.3839175	406	8.12	BCORL1	

DMRX:134796001	X	134796001	1000	1	6.86E-08	1.9931638	32	3.2	PABIR2;PABIR3	
DMRX:135985001	X	135985001	1000	1	4.97E-05	2.0486637	73	7.3	SLC9A6	Transport
DMRX:137431001	X	137431001	2000	1	3.41E-05	1.3731296	3	0.15	RAC1P4	
DMRX:149504001	X	149504001	2000	1	4.32E-05	1.3215942	76	3.8	IDS;IDS2	
DMRX:149528001	X	149528001	1000	1	8.97E-07	1.9546019	4	0.4	IDS2;IDSP1;LINC00893	
DMRX:149539001	X	149539001	2000	1	1.38E-05	1.5730679	97	4.85	LINC00893;EOLA1;HSFX3	Transcription
DMRX:149938001	X	149938001	3000	1	4.64E-05	1.6330928	182	6.066667	EOLA2;HSFX4;LINC00894	Transcription
DMRX:149944001	X	149944001	1000	1	6.31E-05	0.9747079	14	1.4	EOLA2;LINC00894	
DMRX:150824001	X	150824001	1000	1	4.98E-07	1.7032543	0	0	CD99L2;PPIAP91	
DMRX:153255001	X	153255001	1000	1	2.86E-05	1.6279182	10	1		
DMRX:153495001	X	153495001	1000	1	3.30E-05	2.040127	39	3.9	HAUS7;ECMXP;BGN	
DMRX:154389001	X	154389001	1000	1	4.28E-05	2.6932271	96	9.6	EMD;RPL10	Translation
DMRX:154437001	X	154437001	1000	1	9.69E-05	2.352696	95	9.5	CH17-340M24.3;ATP6AP1;GDI1;FAM50A	Metabolism;Signaling
DMRY:9353001	Y	9353001	1000	1	4.62E-05	-1.2639768	9	0.9	FAM197Y8;TSPY8	Epigenetic

Supplemental Table S4
DMR Table Activity Female 1e-04

DMR Name	Chr	Start	Length	# Sig Win	minP	maxLFC	CpG #	CpG Density	Gene Annotation	Gene Category
DMR1:1171001	1	1171001	1000	1	2.68E-05	-0.8516337	38	3.8	MIR200B;MIR200A;MIR429;TTLL10-AS1;TTLL10	
DMR1:11367001	1	11367001	1000	1	5.31E-05	-0.9215992	15	1.5	LOC105376739;LOC105376740	
DMR1:30601001	1	30601001	1000	1	5.98E-05	-0.9954342	11	1.1		
DMR1:60907001	1	60907001	1000	1	1.75E-05	0.9861331	12	1.2	LOC105378764	
DMR1:63904001	1	63904001	1000	1	7.77E-05	0.7867719	9	0.9	ROR1	Receptor
DMR1:108489001	1	108489001	1000	1	7.64E-05	-0.6536165	78	7.8		
DMR1:187703001	1	187703001	1000	1	9.32E-05	-0.7766313	38	3.8	SLC4A1APP2	
DMR2:24302001	2	24302001	1000	1	6.09E-05	0.8028772	23	2.3	ITSN2	Transport
DMR2:29259001	2	29259001	1000	1	3.05E-05	1.1408279	10	1	ALK	Receptor
DMR2:33909001	2	33909001	1000	1	8.88E-05	0.6773225	6	0.6	LINC01317	
DMR2:48326001	2	48326001	1000	1	3.65E-05	0.858934	5	0.5	FOXN2	
DMR2:92655001	2	92655001	1000	1	9.16E-06	1.119608	12	1.2		
DMR2:100741001	2	100741001	1000	1	8.08E-05	-1.0204756	8	0.8	LOC105375310;LOC105373508	
DMR2:106336001	2	106336001	1000	1	2.05E-05	-0.8264929	8	0.8	ILRUNP1	
DMR3:50326001	3	50326001	1000	1	8.21E-05	0.7595643	21	2.1	HYAL2;TUSC2;RASSF1	Metabolism;Cytoskeleton
DMR3:63927001	3	63927001	1000	1	6.57E-05	-0.9453321	10	1	ATXN7	
DMR3:71390001	3	71390001	1000	1	4.28E-05	-1.0011886	12	1.2	FOXP1	
DMR3:110316001	3	110316001	1000	1	9.05E-05	-0.9772592	3	0.3		
DMR3:165196001	3	165196001	1000	1	3.73E-05	-1.1086429	58	5.8	SLITRK3;LINC01322	
DMR4:86979001	4	86979001	1000	1	7.90E-05	0.9989322	16	1.6	AFF1	Transcription
DMR4:114438001	4	114438001	1000	1	4.47E-05	0.9449089	6	0.6		
DMR4:150910001	4	150910001	1000	1	4.83E-05	1.0727728	9	0.9	LRBA	
DMR5:474001	5	474001	1000	1	4.30E-05	-0.946201	79	7.9	EXOC3;SLC9A3;PP7080;SLC9A3-AS1	Transport;Transport
DMR5:10307001	5	10307001	2000	1	4.01E-05	-1.152241	79	3.95	CMBL;LOC105374651	
DMR5:122149001	5	122149001	1000	1	9.52E-05	-1.0420674	12	1.2	AS1;ZNF474;LOC100505841	Transcription
DMR5:134779001	5	134779001	1000	1	8.42E-07	1.0379501	18	1.8	DDX46	
DMR5:146154001	5	146154001	1000	1	6.23E-05	0.8274374	17	1.7	LARS1	Translation
DMR6:106667001	6	106667001	1000	1	1.30E-07	1.3501911	9	0.9	QRSL1	Metabolism
DMR6:107134001	6	107134001	1000	1	8.47E-05	-0.8432768	14	1.4	RNU6-1299P	
DMR6:125029001	6	125029001	1000	1	2.33E-05	0.9835007	6	0.6	RNF217	Proteolysis
DMR6:166981001	6	166981001	1000	1	2.36E-05	1.0392199	9	0.9	LOC105378120	
DMR7:1404001	7	1404001	1000	1	7.50E-05	-0.8176941	26	2.6		
DMR7:29580001	7	29580001	2000	1	7.57E-06	-0.7583464	53	2.65		
DMR7:39295001	7	39295001	1000	1	4.34E-06	-1.0886774	11	1.1	POU6F2	
DMR7:78978001	7	78978001	1000	1	4.60E-05	0.9840403	8	0.8	MAGI2	
DMR7:88039001	7	88039001	1000	1	9.57E-05	0.7040459	16	1.6	ADAM22	Protease
DMR7:92228001	7	92228001	1000	1	6.55E-06	0.9196559	10	1	KRIT1	Cytoskeleton
DMR7:100523001	7	100523001	2000	1	6.35E-05	-0.7210724	127	6.35	LOC107986829;RN7SL416P	
DMR7:140015001	7	140015001	1000	1	6.01E-05	-0.7520128	14	1.4	TBXAS1;PARP12	Metabolism
DMR8:6453001	8	6453001	1000	1	9.67E-06	0.9459981	12	1.2	MCPH1	DNA Repair
DMR8:32755001	8	32755001	2000	1	3.17E-06	-0.8357478	26	1.3	NRG1	Growth Factors
DMR8:91129001	8	91129001	2000	1	6.05E-05	-1.0858967	15	0.75	LRRC69	Cytoskeleton
DMR8:112547001	8	112547001	1000	1	7.26E-05	0.8579381	8	0.8	CSMD3;RPL30P16	
DMR9:196001	9	196001	1000	1	8.18E-05	0.9819944	13	1.3	LOC105375943	
DMR9:20594001	9	20594001	1000	1	8.17E-05	-1.0774078	12	1.2	MLLT3;LOC107987052	Transcription
DMR9:38526001	9	38526001	1000	1	1.82E-05	1.0877828	42	4.2	FAM220BP	
DMR10:84496001	10	84496001	1000	1	7.19E-05	0.9123825	16	1.6	CCSER2	
DMR10:92445001	10	92445001	1000	1	9.93E-07	1.2894892	15	1.5	IDE	Protease
DMR10:96777001	10	96777001	1000	1	1.47E-05	-0.8233216	29	2.9		
DMR11:25008001	11	25008001	1000	1	4.84E-05	-1.0428433	12	1.2	LUZP2	
DMR11:46419001	11	46419001	1000	1	4.84E-05	-1.1181666	8	0.8	AMBRA1;RPS10P19	
DMR11:67822001	11	67822001	1000	1	9.58E-05	-0.9121288	18	1.8	ENPP7P7	
DMR11:117868001	11	117868001	1000	1	9.24E-05	-0.6827925	22	2.2	FXYD6-FXYD2;FXYD6	Transport
DMR11:129113001	11	129113001	1000	1	3.37E-05	-1.1049256	5	0.5	ARHGAP32	Signaling
DMR12:54583001	12	54583001	1000	1	7.00E-06	-0.9314141	12	1.2	PDE1B;PPP1R1A	Signaling;Signaling
DMR12:80202001	12	80202001	1000	1	8.34E-06	1.1176368	10	1	OTOGL	Extracellular Matrix
DMR12:121533001	12	121533001	2000	1	2.95E-05	-0.776559	43	2.15	KDM2B	

DMR12:127772001	12	127772001	1000	1	7.37E-05	-1.0497356	9	0.9		
DMR12:132618001	12	132618001	2000	1	8.56E-05	-0.9223151	175	8.75	LRCOL1;P2RX2;POLE	Signaling;Ion Channel;Transcription
DMR14:54262001	14	54262001	1000	1	1.85E-05	-1.0192604	11	1.1		
DMR15:77028001	15	77028001	1000	1	1.32E-05	-1.001416	50	5	PSTPIP1	Cytoskeleton
DMR15:84970001	15	84970001	1000	1	9.65E-07	-1.146135	11	1.1	SLC28A1;PDE8A	Transport;Signaling
DMR15:89765001	15	89765001	1000	1	9.93E-05	-0.9056335	8	0.8		
DMR15:89906001	15	89906001	1000	1	4.80E-05	-0.8704713	11	1.1	ARPIN-AP3S2;ARPIN;RNU7-111P	
DMR15:93722001	15	93722001	1000	1	1.70E-05	-0.8591376	4	0.4	LOC107983974	
DMR16:21638001	16	21638001	1000	1	1.38E-05	1.100014	8	0.8	METTL9;IGSF6;RNU6-1005P;RNU6-196P	Immune
DMR16:51288001	16	51288001	1000	1	6.07E-05	0.7560215	13	1.3	UNGP1	
DMR17:1561001	17	1561001	1000	1	8.69E-05	-0.6910111	35	3.5	PITPNA;SLC43A2	Transport
DMR17:10749001	17	10749001	1000	1	9.43E-05	-1.0001069	13	1.3	TMEM220-AS1	
DMR17:60113001	17	60113001	1000	1	3.26E-05	-0.9667747	18	1.8		
DMR17:62666001	17	62666001	2000	1	4.71E-05	-0.8304495	74	3.7	MRC2	
DMR18:3244001	18	3244001	1000	1	9.33E-05	0.8767558	16	1.6	LOC101927044;MYL12A	Cytoskeleton
DMR19:19546001	19	19546001	1000	1	1.34E-05	-0.799124	32	3.2	YJEFN3;CILP2	Metabolism
DMR20:24585001	20	24585001	1000	1	9.63E-05	-1.0285147	18	1.8	SYNDIG1	
DMR20:34344001	20	34344001	1000	1	1.86E-05	-0.8143655	21	2.1		
DMR21:16293001	21	16293001	1000	1	2.59E-05	-1.0576813	7	0.7	MIR99AHG;SNORD74B	
DMR21:32143001	21	32143001	1000	1	1.00E-05	0.9745884	20	2		
DMR22:42668001	22	42668001	1000	1	9.32E-05	0.7232808	13	1.3		
DMR22:45029001	22	45029001	1000	1	9.64E-05	-1.0379535	16	1.6		
DMR22:47310001	22	47310001	1000	1	9.76E-05	-1.0196725	7	0.7		

Supplemental Table S5
DMR Table Walkability Male 1e-04

DMR Name	Chr	Start	Length	# Sig Win	minP	maxLFC	CpG #	CpG Density	Gene Annotation	Gene Category
DMR1:3394001	1	3394001	1000	1	2.90E-05	-1.8307962	58	5.8	PRDM16	Transcription
DMR1:18385001	1	18385001	1000	1	1.49E-06	1.2341086	11	1.1	IGSF21	
DMR1:21893001	1	21893001	2000	1	6.47E-05	1.0379201	29	1.45	HSPG2	Extracellular Matrix
DMR1:122349001	1	122349001	1000	1	9.60E-05	-1.7447288	18	1.8		
DMR1:123164001	1	123164001	2000	1	1.96E-05	-1.8755319	37	1.85		
DMR1:123867001	1	123867001	1000	1	4.24E-06	-2.149883	22	2.2		
DMR1:123926001	1	123926001	2000	1	2.56E-05	-1.5613253	31	1.55		
DMR1:163409001	1	163409001	1000	1	3.82E-05	-1.394613	13	1.3		
DMR1:175326001	1	175326001	2000	1	3.99E-07	1.3898472	21	1.05	TNR;LOC105371623	Signaling
DMR1:208697001	1	208697001	1000	1	5.23E-05	-1.6059514	14	1.4	LOC105372892;RPS26P13	
DMR1:228068001	1	228068001	6000	2	4.90E-05	0.9260582	96	1.6	WNT3A;LINC02809;ARF1	Signaling;Signaling
DMR2:10977001	2	10977001	1000	1	4.09E-05	-1.8826268	19	1.9		
DMR2:30969001	2	30969001	2000	1	9.39E-05	-1.505153	27	1.35	GALNT14	Golgi
DMR2:70434001	2	70434001	2000	2	1.06E-05	1.2775377	1	0.05	TRUND-NNN9-1	
DMR2:116375001	2	116375001	1000	1	5.00E-05	1.327538	6	0.6	LOC105373576	
DMR2:198277001	2	198277001	1000	1	3.17E-05	1.4713954	7	0.7		
DMR2:211924001	2	211924001	1000	1	3.93E-05	-1.8418039	7	0.7	ERBB4	Receptor
DMR2:221566001	2	221566001	1000	1	1.92E-06	1.8886068	3	0.3	EPHA4	Receptor
DMR2:242024001	2	242024001	2000	1	2.78E-05	0.9801476	37	1.85	LINC01237	
DMR3:34256001	3	34256001	1000	1	7.85E-06	1.1924847	7	0.7	LINC01811	
DMR3:57142001	3	57142001	1000	1	7.12E-05	-1.65827	15	1.5	IL17RD;RPL19P2;LOC105377101	Receptor
DMR3:86779001	3	86779001	1000	1	9.19E-05	1.4406472	8	0.8		
DMR3:163203001	3	163203001	1000	1	1.16E-05	1.9265837	7	0.7	LINC01192	
DMR4:24432001	4	24432001	1000	1	8.74E-05	1.0926065	10	1	PPARGC1A	Transcription
DMR4:71074001	4	71074001	1000	1	4.23E-05	1.5474741	6	0.6	SLC4A4	Transport
DMR4:102496001	4	102496001	1000	1	7.73E-05	1.8585978	12	1.2	LOC105377621;NFKB1	Transcription
DMR4:165424001	4	165424001	1000	1	5.56E-05	-1.6360043	22	2.2	CPE	Protease
DMR4:183514001	4	183514001	1000	1	1.98E-05	1.0337852	18	1.8	LOC389247;ING2	Epigenetic
DMR5:1418001	5	1418001	2000	1	7.68E-05	0.8863687	12	0.6	SLC6A3	Transport
DMR5:5774001	5	5774001	2000	1	1.72E-05	1.5666236	8	0.4	LOC105374636	
DMR5:7741001	5	7741001	1000	1	4.06E-05	1.0067048	3	0.3	ADCY2	
DMR5:27724001	5	27724001	1000	1	6.82E-05	-1.4633521	16	1.6		
DMR5:37497001	5	37497001	1000	1	9.02E-05	0.9030269	9	0.9	WDR70;KCTD9P5	
DMR5:140558001	5	140558001	2000	1	6.70E-05	1.2350758	21	1.05	ANKHD1-EIF4EBP3;EIF4EBP3;SRA1;APBB3;MIR6831;SLC35A4	Translation;Transport
DMR5:174631001	5	174631001	1000	1	4.69E-05	-1.3826291	17	1.7	LOC105377740;HIGD1AP3	
DMR6:16679001	6	16679001	1000	1	9.52E-05	0.9272367	3	0.3	ATXN1	
DMR6:29819001	6	29819001	1000	1	8.55E-05	1.0151381	14	1.4	MICG;HCG4P8;HLA-G	Immune
DMR6:44711001	6	44711001	1000	1	7.84E-05	0.8839075	7	0.7		
DMR6:57791001	6	57791001	2000	1	1.04E-05	1.4144673	28	1.4	LOC105375103;TRI-AAT1-1	
DMR6:62794001	6	62794001	1000	1	5.93E-05	0.9532836	7	0.7		
DMR6:170249001	6	170249001	2000	1	7.51E-05	-1.4207238	62	3.1	LOC154449	
DMR6:170485001	6	170485001	1000	1	4.43E-05	-1.7422247	11	1.1	LOC105378157	
DMR7:16001	7	16001	1000	1	8.27E-05	0.9312912	8	0.8	LOC102723872	
DMR7:4405001	7	4405001	2000	1	9.81E-05	1.2894045	11	0.55		
DMR7:14072001	7	14072001	1000	1	9.32E-05	1.7878975	5	0.5	RPL6P21	
DMR7:24794001	7	24794001	1000	1	6.11E-05	1.1023466	10	1	LOC107986779;OSBPL3	
DMR7:59006001	7	59006001	1000	1	7.91E-05	-1.5475104	16	1.6		
DMR7:59176001	7	59176001	1000	1	5.63E-05	-1.5925456	16	1.6		
DMR7:59566001	7	59566001	2000	1	9.72E-05	-1.0355549	32	1.6		
DMR7:59953001	7	59953001	1000	1	1.37E-05	-1.5833046	16	1.6		
DMR7:68210001	7	68210001	1000	1	6.76E-05	1.2853382	8	0.8	LOC105375341	
DMR7:92174001	7	92174001	1000	1	1.53E-05	-2.0034871	10	1	CYP51A1-AS1;LRRD1	Cytoskeleton
DMR7:130895001	7	130895001	1000	1	6.34E-05	1.5087626	23	2.3	LINC-PINT	
DMR7:151269001	7	151269001	2000	1	6.49E-05	1.0633671	36	1.8	SMARCD3	Epigenetic
DMR8:38556001	8	38556001	1000	1	1.36E-05	1.3245675	14	1.4	LOC105379383;LOC105379384	
DMR8:44952001	8	44952001	1000	1	8.07E-05	-1.4282001	14	1.4		
DMR8:76677001	8	76677001	1000	1	7.90E-05	-2.0401056	22	2.2	ZFHX4-AS1;ZFHX4	Transcription
DMR8:90786001	8	90786001	1000	1	2.01E-06	1.5677394	7	0.7	LOC105375633;NECAB1	
DMR8:132543001	8	132543001	1000	1	6.04E-05	-1.9011115	5	0.5		
DMR9:35558001	9	35558001	1000	1	7.32E-05	-1.1467132	19	1.9	RUSC2;FAM166B	
DMR9:123155001	9	123155001	1000	1	5.08E-05	1.0075685	11	1.1	STRBP	Metabolism
DMR9:135726001	9	135726001	2000	1	1.82E-05	1.0125847	33	1.65	KCNT1	Transport
DMR10:16851001	10	16851001	2000	1	7.38E-05	0.9711177	9	0.45	CUBN	

DMR10:23350001	10	23350001	1000	1	2.41E-05	1.4408462	2	0.2	C10orf67;LOC105376453	
DMR10:28384001	10	28384001	1000	1	9.72E-05	1.0431167	8	0.8		
DMR10:29838001	10	29838001	2000	1	9.16E-05	1.4317403	19	0.95	LOC105376476;LOC102724333;LOC107984172	
DMR10:98352001	10	98352001	1000	1	4.45E-05	1.6393907	13	1.3	LOC105378449	
DMR11:1860001	11	1860001	2000	1	7.55E-05	1.0154789	8	0.4	LSP1;MIR4298	Cytoskeleton
DMR11:64534001	11	64534001	1000	1	4.54E-05	1.952258	24	2.4		
DMR11:133655001	11	133655001	1000	1	5.99E-05	-1.8184401	13	1.3		
DMR12:6963001	12	6963001	1000	1	1.11E-05	1.8223647	40	4	PTPN6;LOC105369634;MIR200CHG;MIR200C;MIR141;PHB2;SCARNA12;EMG1	Signaling;Epigenetic
DMR12:15343001	12	15343001	2000	1	9.48E-06	-1.3809054	36	1.8	PTPRO;LOC105369673	Receptor
DMR12:34196001	12	34196001	1000	1	4.43E-05	-1.7952688	9	0.9	RNA5SP357	
DMR12:41143001	12	41143001	1000	1	6.95E-05	-1.2748605	24	2.4		
DMR12:54601001	12	54601001	1000	1	1.45E-05	-2.1078768	6	0.6	GLYCAM1	
DMR12:56577001	12	56577001	1000	1	9.31E-05	-1.6033976	20	2	RBMS2	
DMR12:95655001	12	95655001	1000	1	9.71E-05	0.8474433	4	0.4	PGAM1P5;NTN4	Extracellular Matrix
DMR12:106858001	12	106858001	1000	1	5.68E-05	-1.8280689	8	0.8	RIC8B;LOC105369961	
DMR12:127123001	12	127123001	3000	1	2.85E-06	1.3227068	9	0.3	LOC107984449	
DMR12:129418001	12	129418001	1000	1	6.41E-05	-1.7440527	11	1.1	TMEM132D	
DMR12:130666001	12	130666001	2000	1	6.03E-05	1.028717	12	0.6	RIMBP2	
DMR14:79019001	14	79019001	2000	1	9.95E-05	1.6475593	14	0.7	NRXN3	
DMR14:96928001	14	96928001	2000	1	1.04E-05	1.1114969	9	0.45		
DMR14:97590001	14	97590001	1000	1	9.55E-05	1.3935561	10	1	LINC02325	
DMR14:101550001	14	101550001	1000	1	3.82E-05	0.9334778	7	0.7	LOC105370673;LOC105370674;DIO3OS;MIR1247	
DMR15:69078001	15	69078001	1000	1	8.78E-05	1.4686866	25	2.5	EWSAT1	
DMR16:6214001	16	6214001	2000	1	4.88E-05	1.0349426	10	0.5	RBFOX1	Translation
DMR16:30141001	16	30141001	1000	1	5.69E-05	-1.3966319	18	1.8		
DMR16:35425001	16	35425001	1000	1	3.00E-05	-1.7387292	42	4.2	AGGF1P5;C2orf69P3	
DMR17:11260001	17	11260001	1000	1	8.56E-05	1.0039081	12	1.2	SHISA6	
DMR17:79569001	17	79569001	2000	1	2.87E-05	1.0670788	15	0.75	RBFOX3	Translation
DMR18:3793001	18	3793001	1000	1	8.71E-05	1.0668757	15	1.5	DLGAP1	Cytoskeleton
DMR18:75454001	18	75454001	1000	1	5.71E-05	-1.4910477	21	2.1	SMIM21;LOC107985177	
DMR18:75970001	18	75970001	1000	1	9.19E-05	-1.2475598	21	2.1		
DMR19:4228001	19	4228001	1000	1	6.77E-05	0.9049058	5	0.5	ANKRD24;EBI3	Receptor
DMR19:5796001	19	5796001	1000	1	4.36E-05	1.0742285	16	1.6	DUS3L;NRTN	Translation;Growth Factors
DMR19:13272001	19	13272001	1000	1	7.56E-05	1.237798	7	0.7	CACNA1A	Transport
DMR19:16563001	19	16563001	1000	1	6.19E-05	1.1894561	22	2.2	SLC35E1;LOC105372295	Transport
DMR19:17445001	19	17445001	1000	1	7.03E-05	0.9351762	6	0.6	TMEM221;NXNL1	Metabolism
DMR19:18657001	19	18657001	2000	1	4.33E-05	1.0079134	25	1.25	KLHL26	
DMR19:26095001	19	26095001	2000	1	1.79E-05	-1.6617841	34	1.7		
DMR19:26576001	19	26576001	2000	1	4.11E-05	-1.34424	36	1.8		
DMR19:26733001	19	26733001	5000	1	5.15E-05	-1.737526	96	1.92		
DMR19:36470001	19	36470001	1000	1	3.97E-06	1.4484818	13	1.3	ZNF566	Transcription
DMR19:43392001	19	43392001	1000	1	9.85E-05	1.1930369	5	0.5	TEX101	
DMR19:46744001	19	46744001	1000	1	5.63E-05	1.1346909	19	1.9	STRN4;FKRP	
DMR19:48486001	19	48486001	1000	1	7.32E-06	1.3521993	40	4	CYTH2;LMTK3	Transcription;Signaling
DMR19:50456001	19	50456001	1000	1	9.71E-05	0.8489625	12	1.2	MYBPC2;FAM71E1	
DMR20:62269001	20	62269001	2000	1	2.76E-05	1.0652952	48	2.4	OSBPL2	
DMR21:5236001	21	5236001	1000	1	6.38E-05	-1.5498181	3	0.3	LOC102724200	
DMR21:44504001	21	44504001	2000	1	2.21E-05	-1.1735505	62	3.1	TSPEAR;TSPEAR-AS1	Signaling
DMR22:20382001	22	20382001	1000	1	4.16E-05	1.8328368	16	1.6	USP41	Protease
DMR22:37328001	22	37328001	2000	1	2.81E-05	0.981341	26	1.3	LOC105373024	
DMRX:2606001	X	2606001	2000	1	2.24E-05	1.0681555	24	1.2	LOC101928092;MIR6089;CD99P1;LINC00102	
DMRX:3792001	X	3792001	1000	1	5.64E-05	1.221659	7	0.7		
DMRX:6621001	X	6621001	2000	1	5.35E-05	1.1944019	10	0.5		
DMRX:120581001	X	120581001	2000	1	9.88E-05	1.6201251	16	0.8	CUL4B	Proteolysis

Supplemental Table S6
DMR Table Walkability Female 1e-04

DMR Name	Chr	Start	Length	# Sig Win	minP	maxLFC	CpG #	CpG Density	Gene Annotation	Gene Category
DMR1:64973001	1	64973001	1000	1	9.90E-05	-0.9440986	5	0.5	JAK1;LINC01359;SLC2A3P2	
DMR1:90024001	1	90024001	1000	1	2.63E-05	-1.535283	6	0.6	ZNF326	Cytoskeleton
DMR2:28998001	2	28998001	1000	1	7.91E-05	-1.3863437	18	1.8	TOGARAM2	Cytoskeleton
DMR2:73189001	2	73189001	1000	1	5.40E-05	1.1075019	10	1		
DMR2:75275001	2	75275001	1000	1	2.80E-05	-1.1305682	4	0.4	LOC105374811;LOC107985900	
DMR2:77725001	2	77725001	1000	1	3.45E-05	-1.08005	2	0.2		
DMR2:116753001	2	116753001	1000	1	6.92E-05	1.281977	11	1.1	MTCYBP39	
DMR3:30399001	3	30399001	1000	1	2.84E-05	-1.2009731	1	0.1	LOC101927995	
DMR3:41602001	3	41602001	1000	1	4.18E-07	-1.4479778	2	0.2	ULK4	Signaling
DMR3:137150001	3	137150001	1000	1	9.05E-05	1.2321107	7	0.7		
DMR3:174201001	3	174201001	1000	1	1.84E-05	-1.0955049	4	0.4	NLGN1	Cytoskeleton
DMR3:176751001	3	176751001	2000	1	1.94E-05	-1.0715161	4	0.2		
DMR3:192247001	3	192247001	1000	1	4.65E-05	-1.1805014	3	0.3	FGF12;FGF12-AS1	Growth Factors
DMR4:18611001	4	18611001	2000	1	2.43E-06	-1.0642423	5	0.25	LOC105374510	
DMR4:18704001	4	18704001	1000	1	5.78E-05	-1.21091	4	0.4	LOC105374510	
DMR4:48728001	4	48728001	1000	1	9.97E-05	-0.9985007	10	1	FRYL	Cytoskeleton
DMR4:112119001	4	112119001	1000	1	9.38E-05	-1.3408825	6	0.6	RPS12P8;LOC105377370	
DMR4:126942001	4	126942001	1000	1	9.94E-05	1.1085205	6	0.6		
DMR4:179492001	4	179492001	1000	1	2.68E-05	-1.3216715	3	0.3		
DMR5:139479001	5	139479001	1000	1	3.81E-05	1.4333919	11	1.1	SMIM33;STING1;LOC101059986	
DMR5:173304001	5	173304001	1000	1	9.89E-05	0.9993558	21	2.1	LOC105377731;MIR12118;STC2	Hormone
DMR5:173631001	5	173631001	1000	1	4.77E-05	-0.9499933	5	0.5		
DMR5:176694001	5	176694001	1000	1	2.71E-05	-1.2434265	24	2.4		
DMR5:177179001	5	177179001	2000	1	2.23E-05	1.0184414	30	1.5	NSD1	
DMR6:1542001	6	1542001	1000	1	9.69E-06	-1.2422598	2	0.2	LOC102723944;LOC105374882	
DMR6:4852001	6	4852001	1000	1	6.46E-06	-1.3493239	5	0.5	CDYL	
DMR6:74948001	6	74948001	1000	1	5.38E-06	-1.2057814	7	0.7		
DMR7:47635001	7	47635001	1000	1	3.29E-07	-1.3865523	27	2.7	LINC01447	
DMR7:76281001	7	76281001	2000	1	4.68E-05	-1.5482854	174	8.7	SRRM3	
DMR7:82393001	7	82393001	1000	1	4.09E-05	-1.4921216	4	0.4	CACNA2D1	Transport
DMR7:98400001	7	98400001	1000	1	7.86E-07	-2.0821247	55	5.5	BAIAP2L1	Cytoskeleton
DMR7:98561001	7	98561001	1000	1	4.52E-05	-0.8344638	6	0.6		
DMR7:149772001	7	149772001	1000	1	7.63E-05	-1.2116987	65	6.5	ZNF467;SSPOP	Extracellular Matrix
DMR8:64765001	8	64765001	1000	1	6.09E-05	1.3469994	9	0.9	CYP7B1	Metabolism
DMR8:65582001	8	65582001	1000	1	6.97E-05	-0.9367379	4	0.4		
DMR8:132059001	8	132059001	1000	1	5.40E-05	-1.1910812	4	0.4	OC90;HHLA1	Metabolism
DMR8:142485001	8	142485001	1000	1	7.77E-05	1.0173495	17	1.7	ADGRB1	Signaling
DMR9:69497001	9	69497001	1000	1	7.22E-05	-1.05342	12	1.2	APBA1	Transport
DMR9:115144001	9	115144001	1000	1	1.56E-05	-1.1082348	4	0.4	LOC101928748;DELEC1	
DMR9:116503001	9	116503001	1000	1	7.91E-05	-1.0143626	7	0.7	ASTN2;ASTN2-AS1	
DMR9:119835001	9	119835001	1000	1	9.94E-05	-0.9794907	1	0.1		
DMR10:11653001	10	11653001	2000	1	9.00E-05	-1.3007459	25	1.25	LOC105376413	
DMR10:17577001	10	17577001	1000	1	8.24E-05	1.1058569	21	2.1	PRPF38AP2	
DMR10:30778001	10	30778001	2000	1	5.00E-06	-1.0289078	16	0.8		
DMR10:33733001	10	33733001	1000	1	7.80E-05	-1.0098726	10	1		
DMR10:43325001	10	43325001	1000	1	5.64E-06	1.4764657	12	1.2	LOC105378271;LINC02633	
DMR10:119953001	10	119953001	1000	1	1.90E-05	-1.0815767	2	0.2	SEC23IP;MIR4682	Metabolism
DMR10:123992001	10	123992001	1000	1	5.65E-05	-1.2271973	38	3.8	YBX2P1	
DMR11:79524001	11	79524001	1000	1	7.31E-05	-1.2124632	18	1.8		
DMR11:87755001	11	87755001	1000	1	5.10E-05	-1.2150724	1	0.1	LOC107984361	
DMR12:1662001	12	1662001	2000	1	2.80E-05	-1.0075283	102	5.1	MIR3649	
DMR12:116737001	12	116737001	1000	1	2.38E-05	-1.2517435	51	5.1	SPRING1;RNFT2	
DMR13:17699001	13	17699001	2000	1	6.14E-05	1.0167566	34	1.7		
DMR13:22160001	13	22160001	1000	1	8.73E-05	1.2033797	9	0.9	LOC105370108	
DMR13:87709001	13	87709001	2000	1	7.06E-05	-1.1848751	11	0.55		
DMR13:110255001	13	110255001	1000	1	6.20E-05	-0.8780932	9	0.9	COL4A1	Extracellular Matrix
DMR14:70822001	14	70822001	1000	1	1.55E-06	-1.4374048	69	6.9	MAP3K9-DT	

DMR14:79262001	14	79262001	1000	1	9.81E-05	-1.2763606	2	0.2	NRXN3;LOC112268125	
DMR14:82810001	14	82810001	1000	1	6.67E-05	-1.0607631	6	0.6		
DMR14:95374001	14	95374001	2000	1	2.28E-05	-1.0630017	11	0.55	LOC107984710	
DMR15:45061001	15	45061001	1000	1	3.56E-05	-1.096146	21	2.1	SORD;LOC100422669	Metabolism
DMR15:79428001	15	79428001	1000	1	2.86E-05	-1.1612151	2	0.2	MINAR1	Receptor
DMR15:82058001	15	82058001	1000	1	9.97E-05	-0.9373824	18	1.8		
DMR16:80724001	16	80724001	1000	1	9.50E-05	-0.9517311	1	0.1	CDYL2	
DMR16:84618001	16	84618001	1000	1	4.27E-09	-2.0958324	50	5	COTL1	Cytoskeleton
DMR17:23059001	17	23059001	1000	1	1.09E-05	1.2422448	17	1.7		
DMR17:32552001	17	32552001	1000	1	4.66E-06	-1.325663	8	0.8	MYO1D	Cytoskeleton
DMR17:35175001	17	35175001	1000	1	5.27E-05	-0.9818084	4	0.4	UNC45B	
DMR17:65852001	17	65852001	1000	1	2.98E-05	-1.1438337	3	0.3	CEP112	
DMR19:125001	19	125001	1000	1	4.88E-05	-1.3033176	3	0.3		
DMR19:912001	19	912001	1000	1	5.33E-05	-1.2931278	70	7	R3HDM4;KISS1R	Signaling
DMR19:4145001	19	4145001	1000	1	5.48E-06	-1.0168025	10	1	CREB3L3	
DMR19:7394001	19	7394001	1000	1	4.90E-05	-1.6103007	33	3.3	ARHGEF18;LOC107985284	
DMR19:13618001	19	13618001	2000	1	8.46E-05	-1.0539533	12	0.6		
DMR19:16777001	19	16777001	1000	1	7.76E-05	-1.088569	0	0	NWD1	
DMR19:25334001	19	25334001	2000	1	3.00E-05	1.2073185	34	1.7		
DMR19:47581001	19	47581001	2000	1	1.89E-05	-1.2454288	19	0.95	ZNF541;RN7SL322P	
DMR20:21623001	20	21623001	1000	1	2.05E-05	-1.1502775	2	0.2	LINC01727;LINC01726	
DMR20:59265001	20	59265001	1000	1	4.95E-05	-1.1115943	0	0	ZNF831;LOC107985389	
DMR21:12803001	21	12803001	1000	1	1.02E-05	1.3068749	17	1.7		
DMR21:21991001	21	21991001	1000	1	4.07E-05	-1.3421353	6	0.6		
DMR21:23371001	21	23371001	1000	1	5.54E-06	1.0900801	12	1.2	D21S2088E	
DMR22:30158001	22	30158001	1000	1	1.21E-05	-1.0006222	8	0.8	HORMAD2	
DMR22:31741001	22	31741001	1000	1	1.37E-05	-1.0982798	22	2.2	PRR14L	
DMR22:47940001	22	47940001	1000	1	5.17E-05	1.1165879	15	1.5		
DMRX:20559001	X	20559001	1000	1	5.49E-06	-1.1440448	1	0.1		
DMRX:91879001	X	91879001	1000	1	9.67E-05	-1.2982338	10	1	PCDH11X	Cytoskeleton
DMRX:118694001	X	118694001	1000	1	7.20E-05	1.3426755	14	1.4	DOCK11	Transcription

Supplemental Table S7
DMR Table BMI Male 1e-04

DMR Name	Chr	Start	Length	# Sig Win	minP	maxLFC	CpG #	CpG Density	Gene Annotation	Gene Category
DMR1:25406001	1	25406001	1000	1	6.95E-05	-1.518728	36	3.6	RHCE;SDHDP7	Transport
DMR1:150654001	1	150654001	1000	1	3.38E-06	-1.998081	13	1.3	GOLPH3L	
DMR1:220884001	1	220884001	1000	1	8.40E-05	1.7693852	64	6.4	HLX-AS1;HLX	Development
DMR1:222699001	1	222699001	2000	1	3.94E-05	1.369562	53	2.65	AIDA	
DMR2:10044001	2	10044001	2000	1	7.08E-05	1.3732874	122	6.1	LOC112268412;LOC101929882;KLF11	Transcription
DMR2:16283001	2	16283001	1000	1	5.15E-05	-1.7241449	13	1.3		
DMR2:24925001	2	24925001	2000	1	2.56E-05	1.3641736	61	3.05	ADCY3	
DMR2:91747001	2	91747001	2000	1	7.37E-06	1.6614626	76	3.8	LOC100289350;KMT5AP2	
DMR2:144452001	2	144452001	1000	1	6.62E-05	-1.7204008	18	1.8	ZEB2	Transcription
DMR2:219243001	2	219243001	1000	1	5.32E-06	2.0837479	55	5.5	ANKZF1;GLB1L;STK16;TUBA4A;TUBA4B	Metabolism;Signaling;Cytoskeleton
DMR3:50355001	3	50355001	1000	1	7.83E-05	-1.8104613	14	1.4	ZMYND10;NPRL2;CYB561D2;TMEM115;CACNA2D2	Signaling;Transport;Transport
DMR3:72740001	3	72740001	1000	1	5.41E-05	-1.5351549	11	1.1	SHQ1;LOC107986097	
DMR3:90892001	3	90892001	2000	1	1.66E-05	-1.8470817	34	1.7		
DMR3:105504001	3	105504001	1000	1	1.91E-05	1.5871655	26	2.6	ALCAM	Immune
DMR3:198098001	3	198098001	8000	2	4.67E-05	1.3915452	363	4.5375	FRG2FP	
DMR4:63781001	4	63781001	1000	1	2.04E-05	1.3717343	19	1.9	LOC105377254	
DMR4:131735001	4	131735001	7000	1	7.00E-05	1.2116087	471	6.728571429		
DMR4:161685001	4	161685001	1000	1	7.00E-05	-1.6615773	6	0.6	FSTL5	Protease; Proteolysis
DMR4:182807001	4	182807001	3000	1	9.69E-06	1.5912646	109	3.633333333	TENM3	
DMR4:186202001	4	186202001	1000	1	6.13E-05	1.1966468	17	1.7	CYP4V2	Metabolism
DMR4:189703001	4	189703001	1000	1	2.20E-05	1.2808239	26	2.6	LOC105377616;RNU1-51P	
DMR5:23883001	5	23883001	1000	1	9.26E-05	-1.748314	9	0.9		
DMR5:58891001	5	58891001	1000	1	1.13E-05	-1.9257829	18	1.8		
DMR5:94106001	5	94106001	1000	1	2.90E-05	-2.0939328	3	0.3	FAM172A;LOC105379087	
DMR5:178860001	5	178860001	1000	1	2.60E-05	1.7264294	107	10.7	ZNF354B;LOC107986493	
DMR5:180405001	5	180405001	1000	1	7.42E-05	-1.5960672	8	0.8		
DMR6:45448001	6	45448001	1000	1	9.21E-05	-1.2757393	10	1	RUNX2	Transcription
DMR6:141085001	6	141085001	1000	1	9.86E-06	1.7327143	7	0.7		
DMR7:19821001	7	19821001	1000	1	9.59E-05	-1.5875744	7	0.7	LOC107986774;LOC105379720	
DMR7:50972001	7	50972001	1000	1	1.29E-05	-1.9166614	13	1.3		
DMR7:67324001	7	67324001	1000	1	4.64E-05	1.1739557	30	3	STAG3L4	Epigenetic
DMR7:69174001	7	69174001	1000	1	6.46E-05	-1.7334957	10	1	LOC105375344	
DMR7:78330001	7	78330001	1000	1	7.66E-05	1.6710949	29	2.9	MAGI2	
DMR7:94819001	7	94819001	2000	1	5.85E-05	-1.4483785	15	0.75		
DMR8:2674001	8	2674001	1000	1	9.37E-05	1.6608999	18	1.8	LOC101927815;LOC107986865	
DMR8:8219001	8	8219001	2000	1	7.28E-06	1.4293178	38	1.9	FAM85B;ENPP7P1;FAM86B3P	
DMR8:19609001	8	19609001	2000	1	1.51E-05	1.4987389	52	2.6	CSGALNACT1	Golgi
DMR8:30318001	8	30318001	1000	1	9.08E-05	-1.3055317	14	1.4		
DMR8:120554001	8	120554001	2000	1	8.74E-06	1.364851	41	2.05	SNTB1	
DMR8:129496001	8	129496001	1000	1	2.20E-05	1.4430879	36	3.6	CCDC26	
DMR9:87938001	9	87938001	2000	1	1.06E-05	1.3096332	66	3.3	LOC497256;LOC645937	
DMR9:102793001	9	102793001	1000	1	8.71E-06	-2.05	7	0.7		
DMR12:54370001	12	54370001	1000	1	7.20E-05	1.2002535	56	5.6	LOC102724050;GPR84;ZNF385A	Signaling
DMR12:55054001	12	55054001	1000	1	4.43E-05	-1.6712149	14	1.4		
DMR12:57534001	12	57534001	1000	1	3.27E-05	-1.8563956	16	1.6	MBD6;DCTN2	Cytoskeleton
DMR12:103836001	12	103836001	1000	1	4.26E-05	1.4295293	18	1.8	NT5DC3;LOC107984433;TTC41P	Signaling
DMR13:38320001	13	38320001	1000	1	6.37E-05	1.3267276	14	1.4		
DMR13:42385001	13	42385001	1000	1	3.12E-05	-1.8254206	8	0.8		
DMR14:20132001	14	20132001	1000	1	3.93E-05	-1.7919855	7	0.7	OR4K17;OR4N5	Receptor
DMR14:100782001	14	100782001	2000	1	8.35E-05	-1.7617142	40	2		
DMR15:20034001	15	20034001	4000	1	5.50E-05	-1.3754022	32	0.8	LOC646071	
DMR15:82067001	15	82067001	2000	1	4.40E-05	1.4633895	34	1.7		
DMR15:90817001	15	90817001	1000	1	9.60E-06	-2.0838819	15	1.5	BLM	Epigenetic
DMR16:8335001	16	8335001	1000	1	2.12E-05	1.5665136	5	0.5		
DMR16:66121001	16	66121001	1000	1	1.86E-05	-1.5112913	10	1		
DMR16:66579001	16	66579001	1000	1	5.35E-05	1.3329908	73	7.3	CKLF-CMTM1;CMTM1;CMTM2	Transport
DMR16:89330001	16	89330001	1000	1	1.18E-05	-1.8211503	27	2.7	ANKRD11;LOC100287036	
DMR17:355001	17	355001	2000	1	7.99E-05	1.5860889	124	6.2	RPH3AL	
DMR17:43867001	17	43867001	1000	1	2.29E-05	-1.9864733	3	0.3	CD300LG;MPP2	Immune;Cytoskeleton
DMR17:80826001	17	80826001	1000	1	2.12E-05	-1.8259791	34	3.4	RPTOR	
DMR17:82205001	17	82205001	1000	1	2.34E-05	-1.8933139	29	2.9	CCDC57	
DMR18:78062001	18	78062001	1000	1	1.67E-05	1.6238252	8	0.8		
DMR19:564001	19	564001	2000	1	3.83E-05	1.3354971	62	3.1	BSG-AS1;BSG	Cytoskeleton
DMR19:1440001	19	1440001	1000	1	4.09E-05	1.2455425	48	4.8	DAZAP1;RPS15;APC2	Translation
DMR19:2910001	19	2910001	1000	1	6.57E-05	1.3610733	29	2.9	ZNF57;LOC101928631	Transcription
DMR19:7924001	19	7924001	1000	1	3.73E-05	1.3601161	48	4.8	MAP2K7;TGFBR3L;SNAPC2;CTXN1;TIMM44	Signaling;Receptor;Transport
DMR19:43240001	19	43240001	1000	1	2.99E-05	1.1969141	4	0.4	LOC284344	
DMR20:278001	20	278001	2000	1	4.41E-05	1.4842069	113	5.65	C20orf96	
DMR20:2084001	20	2084001	1000	1	2.60E-05	-1.8559762	13	1.3	RPL7P2	
DMR20:27164001	20	27164001	4000	1	3.72E-05	1.1574257	56	1.4		

DMR20:30330001	20	30330001	1000	1	8.54E-05	-1.6774759	13	1.3		
DMR22:38286001	22	38286001	1000	1	8.96E-05	-1.4785605	17	1.7	TPTEP2-CSNK1E;CSNK1E	Signaling
DMR22:48127001	22	48127001	1000	1	2.43E-05	-1.6018537	7	0.7		
DMRX:790001	X	790001	1000	1	3.93E-05	-1.3893424	39	3.9		
DMRX:3915001	X	3915001	1000	1	1.32E-05	1.4192816	78	7.8	FAM239B;TRI-GAT1-3	
DMRX:22362001	X	22362001	1000	1	6.00E-06	1.8051026	23	2.3	PTCHD1-AS	
DMRX:54380001	X	54380001	1000	1	3.43E-05	1.3265	30	3		
DMRX:93696001	X	93696001	1000	1	1.86E-05	1.3865905	30	3	FAM133A	Metabolism
DMRX:103276001	X	103276001	1000	1	8.92E-05	2.4482017	40	4	TCEAL5	Transcription
DMRX:131082001	X	131082001	1000	1	5.78E-05	1.6714965	81	8.1	ARHGAP36	Signaling
DMRX:154427001	X	154427001	2000	1	4.90E-05	1.8096949	108	5.4	TAFAZZIN;CH17-340M24.3;ATP6AP1;GDI1	Metabolism;Signaling
DMRY:8465001	Y	8465001	1000	1	2.14E-05	-2.1877862	4	0.4		

Supplemental Table S8
DMR Table BMI Female 1e-04

DMR Name	Chr	Start	Length	# Sig Win	minP	maxLFC	CpG #	CpG Density	Gene Annotation	Gene Category
DMR1:629001	1	629001	6000	5	8.26E-10	0.9029436	159	2.65	LOC101928626;MTND1P23;MTND2P28;MTCO1P12;MIR12136;MTCO2P12;MTATP8P1;MTATP6P1;MTCO3P12	
DMR1:1284001	1	1284001	1000	1	2.70E-05	0.6574938	36	3.6	LINC01786;SCNN1D;ACAP3	Transport
DMR1:1324001	1	1324001	1000	1	2.30E-05	1.0134595	107	10.7	INTS11;CPTP;TAS1R3	Translation;Transport;Signaling
DMR1:1532001	1	1532001	2000	1	3.53E-05	0.6724353	64	3.2	ATAD3A;TMEM240;SSU72	Signaling
DMR1:2096001	1	2096001	3000	1	7.20E-06	0.7183669	100	3.3333333	PRKCZ	Signaling
DMR1:3166001	1	3166001	1000	1	7.43E-06	0.8973309	40	4	PRDM16;LOC105378606	Transcription
DMR1:4185001	1	4185001	2000	1	7.53E-05	-0.6393857	12	0.6	EEF1DP6	
DMR1:38632001	1	38632001	1000	1	6.03E-05	-0.9661123	3	0.3		
DMR1:55002001	1	55002001	2000	1	4.41E-05	-0.5491449	11	0.55	TMEM61;BSND	
DMR1:68244001	1	68244001	1000	1	7.70E-05	0.7850107	9	0.9	RPS7P4	
DMR1:78875001	1	78875001	4000	1	3.73E-05	-0.5994763	40	1		
DMR1:120701001	1	120701001	1000	1	6.97E-05	-0.553333	48	4.8		
DMR1:121878001	1	121878001	2000	1	1.58E-05	-0.6011456	41	2.05		
DMR1:156809001	1	156809001	1000	1	2.68E-07	0.6934077	18	1.8	PRCC;SH2D2A;NTRK1	Immune;Receptor
DMR1:160084001	1	160084001	1000	1	2.13E-05	0.5718328	112	11.2	KCNJ9;IGSF8	Transport;Immune
DMR1:161447001	1	161447001	4000	1	6.02E-05	0.5230778	427	10.675	TRG-TCC4-1;TRD-GTC2-1;TRL-CAG1-1;TRG-GCC1-1;TRE-CTC1-2;TRG-TCC2-2;TRD-GTC2-2;TRL-CAG1-2;TRG-GCC1-2;TRE-CTC1-3;TRG-TCC2-3;TRD-GTC2-3;TRL-CAG1-3;TRG-GCC1-3	
DMR1:161454001	1	161454001	4000	1	1.32E-05	0.4810077	412	10.3	TRE-CTC1-2;TRG-TCC2-2;TRD-GTC2-2;TRL-CAG1-2;TRG-GCC1-2;TRE-CTC1-3;TRG-TCC2-3;TRD-GTC2-3;TRL-CAG1-3;TRG-GCC1-3;TRE-CTC1-4;TRG-TCC2-4;TRD-GTC2-4;TRL-CAG1-4;TRG-GCC1-4	
DMR1:161469001	1	161469001	5000	1	8.11E-06	0.6406043	418	8.36	TRE-CTC1-4;TRG-TCC2-4;TRD-GTC2-4;TRL-CAG1-4;TRG-GCC1-4;TRE-CTC1-5;TRG-TCC2-5;TRD-GTC2-5;TRL-CAG1-5;TRG-GCC4-1	
DMR1:177485001	1	177485001	1000	1	9.82E-05	-0.8788415	10	1		
DMR1:178501001	1	178501001	1000	1	9.29E-05	0.8300962	18	1.8	CLEC20A;LOC101928866	
DMR1:181549001	1	181549001	1000	1	9.01E-06	0.9789601	11	1.1	CACNA1E	Transport
DMR1:205522001	1	205522001	2000	1	1.59E-06	0.9150359	36	1.8	CDK18	Signaling
DMR1:229796001	1	229796001	2000	1	1.25E-05	0.8662354	31	1.55	LOC105373161	
DMR1:230167001	1	230167001	2000	1	7.93E-05	0.5888667	30	1.5	GALNT2	Golgi
DMR2:1638001	2	1638001	1000	1	5.90E-06	0.9354255	16	1.6	PXDN	Metabolism
DMR2:3170001	2	3170001	2000	1	2.28E-05	0.5800025	61	3.05	LOC107985840	
DMR2:5147001	2	5147001	1000	1	6.83E-05	-0.6142574	12	1.2		
DMR2:33313001	2	33313001	1000	1	5.93E-06	-0.9932479	9	0.9	LTBP1	Extracellular Matrix
DMR2:53796001	2	53796001	2000	1	7.64E-05	0.7692415	27	1.35	ASB3;GPR75-ASB3;ERLEC1	
DMR2:61628001	2	61628001	2000	1	9.57E-05	0.7532805	38	1.9		
DMR2:63052001	2	63052001	4000	1	2.87E-05	0.9502806	210	5.25	EHBP1;LOC100132215;OTX1	Development
DMR2:64869001	2	64869001	1000	1	6.35E-05	0.8206268	16	1.6	LINC01800	
DMR2:69939001	2	69939001	2000	1	9.57E-05	0.6918547	27	1.35	MXD1;ASPRV1	Transcription;Epigenetic
DMR2:70017001	2	70017001	1000	1	4.01E-05	0.9804884	14	1.4	ASPRV1;PCBP1-AS1	Epigenetic
DMR2:87395001	2	87395001	6000	1	5.51E-05	0.7039139	81	1.35		
DMR2:87412001	2	87412001	8000	1	6.11E-05	0.7171048	63	0.7875		
DMR2:87421001	2	87421001	6000	1	1.51E-05	0.6166454	53	0.8833333		
DMR2:87428001	2	87428001	4000	1	4.14E-05	0.5911653	35	0.875	LINC01943	
DMR2:90256001	2	90256001	3000	1	5.34E-05	-0.5451574	10	0.3333333		
DMR2:90286001	2	90286001	5000	1	6.91E-06	0.8428482	54	1.08	LOC101926946	
DMR2:90323001	2	90323001	3000	2	2.33E-05	0.7115028	416	13.866667	IGKV1OR2-118	
DMR2:91504001	2	91504001	13000	1	2.68E-06	0.6862574	142	1.0923077	LOC101927984	
DMR2:91518001	2	91518001	4000	2	8.54E-05	0.59421	37	0.925		
DMR2:91523001	2	91523001	6000	1	9.92E-05	0.6146427	52	0.8666667		
DMR2:114060001	2	114060001	1000	1	8.36E-05	-0.5973718	9	0.9		
DMR2:117057001	2	117057001	1000	1	5.10E-05	-0.6308689	7	0.7		
DMR2:117616001	2	117616001	1000	1	6.17E-05	-0.8972195	4	0.4		
DMR2:161244001	2	161244001	2000	1	3.12E-05	0.7875883	64	3.2	TANK;LOC101929512;LINC01806	
DMR2:181678001	2	181678001	1000	1	7.96E-05	0.7120583	53	5.3	NEUROD1	Transcription

DMR2:183785001	2	183785001	1000	1	4.06E-05	-0.7487083	4	0.4		
DMR2:220055001	2	220055001	1000	1	6.42E-05	-0.919465	9	0.9		
DMR2:237144001	2	237144001	1000	1	2.42E-05	-0.6114048	7	0.7	LOC105373952	
DMR3:18820001	3	18820001	1000	1	4.81E-05	-0.6394955	6	0.6		
DMR3:20828001	3	20828001	1000	1	5.09E-05	-0.6820734	8	0.8		
DMR3:72592001	3	72592001	1000	1	9.72E-05	0.711245	16	1.6	LOC105377161	
DMR3:76286001	3	76286001	1000	1	3.23E-05	-0.8405727	5	0.5	ROBO2	
DMR3:157862001	3	157862001	2000	1	7.86E-05	-0.5814819	8	0.4		
DMR3:179739001	3	179739001	2000	1	8.31E-05	0.7012785	32	1.6	USP13	Protease
DMR3:184540001	3	184540001	1000	1	4.45E-05	0.6859643	20	2	LINC01839;LINC01840	
DMR3:194687001	3	194687001	1000	1	1.65E-05	0.7877262	98	9.8	FAM43A	Cytoskeleton
DMR3:195762001	3	195762001	1000	1	9.78E-05	0.5395722	111	11.1	MUC4	
DMR4:11001	4	11001	1000	1	8.13E-05	0.5992731	148	14.8		
DMR4:745001	4	745001	3000	2	2.91E-05	0.5567238	279	9.3	PCGF3;LOC107986246	Epigenetic
DMR4:11341001	4	11341001	1000	1	1.32E-05	-0.9466825	7	0.7		
DMR4:20812001	4	20812001	1000	1	1.76E-05	-0.6420393	10	1	KCNIP4	
DMR4:32656001	4	32656001	1000	1	3.84E-05	-1.0084505	3	0.3		
DMR4:61373001	4	61373001	1000	1	3.14E-06	1.0937762	16	1.6	ADGRL3	Signaling
DMR4:63592001	4	63592001	2000	1	1.27E-05	-0.9467269	14	0.7		
DMR4:82570001	4	82570001	1000	1	1.56E-05	-0.7010651	4	0.4	TMEM150C;LOC442113	
DMR4:148807001	4	148807001	1000	1	7.53E-05	-0.7322519	3	0.3	LOC107986195;LOC105377481	
DMR4:160449001	4	160449001	1000	1	9.34E-05	-0.9186622	6	0.6		
DMR4:175488001	4	175488001	1000	1	8.74E-05	-0.870987	6	0.6	TSEN2P1	
DMR4:189810001	4	189810001	1000	1	3.32E-05	0.5931771	74	7.4	FRG1-DT;LOC105377619	
DMR4:190120001	4	190120001	2000	1	8.91E-05	0.5006677	248	12.4	LOC107986338	
DMR5:3078001	5	3078001	1000	1	7.92E-05	-0.6743855	24	2.4		
DMR5:6830001	5	6830001	1000	1	9.14E-05	0.6343086	22	2.2	LINC02236;MIR4278;LOC105374641	
DMR5:10639001	5	10639001	2000	1	5.33E-06	0.6422965	213	10.65	ANKRD33B	
DMR5:37097001	5	37097001	1000	1	9.50E-05	-0.7282342	7	0.7	CPLANE1	
DMR5:115600001	5	115600001	1000	1	1.04E-05	-1.0151121	4	0.4	TMED7-TICAM2;TICAM2;TICAM2-AS1	Transport
DMR5:124724001	5	124724001	1000	1	3.56E-05	-0.9328187	9	0.9	ZNF608	
DMR5:159188001	5	159188001	1000	1	6.87E-05	0.9185596	11	1.1	RNF145	
DMR6:3814001	6	3814001	1000	1	5.93E-05	0.6771916	17	1.7		
DMR6:11402001	6	11402001	1000	1	4.40E-05	0.5403324	52	5.2	LOC105374927	
DMR6:14500001	6	14500001	1000	1	3.96E-05	0.6508941	27	2.7	LOC101928331;LOC101928354	
DMR6:44711001	6	44711001	1000	1	2.74E-05	-0.5482093	7	0.7		
DMR6:78127001	6	78127001	1000	1	1.93E-05	-0.8797075	12	1.2	LOC105377865	
DMR6:89161001	6	89161001	1000	1	6.59E-05	-0.5768949	8	0.8	PM20D2	Protease
DMR6:89986001	6	89986001	1000	1	8.56E-05	0.8332914	10	1	BACH2	
DMR6:102102001	6	102102001	1000	1	3.83E-05	-0.6215242	13	1.3		
DMR6:158239001	6	158239001	2000	1	1.02E-05	0.6832307	166	8.3	TULP4;SRP72P2	
DMR6:166783001	6	166783001	2000	1	1.20E-05	0.6243035	55	2.75	RPS6KA2	Golgi
DMR6:167989001	6	167989001	2000	1	4.55E-05	0.5324513	120	6	HGC6.3;KIF25-AS1;KIF25	Cytoskeleton
DMR6:168101001	6	168101001	2000	1	3.84E-05	1.076265	120	6	FRMD1;LOC107986548	
DMR7:44761001	7	44761001	2000	1	3.58E-05	0.7358443	68	3.4	ZMIZ2	
DMR7:66656001	7	66656001	1000	1	1.93E-05	1.0043176	14	1.4	LOC100996437;RABGEF1	Transcription
DMR7:106457001	7	106457001	1000	1	5.85E-05	0.9376022	12	1.2		
DMR7:145997001	7	145997001	1000	1	8.80E-07	1.0883101	6	0.6		
DMR7:146011001	7	146011001	1000	1	2.76E-05	-0.9630143	7	0.7		
DMR7:146552001	7	146552001	1000	1	6.10E-05	-0.6418456	9	0.9	CNTNAP2	
DMR7:149764001	7	149764001	2000	1	6.57E-06	0.6403564	177	8.85	ZNF467	
DMR7:150397001	7	150397001	2000	1	5.46E-05	0.5925552	160	8	ZNF775;LOC107986859;LOC728743	
DMR7:155358001	7	155358001	1000	1	4.68E-05	0.7926312	44	4.4	BLACE;LOC105375593	
DMR7:155492001	7	155492001	1000	1	5.05E-05	-0.6854404	12	1.2	LOC107986863;CNPY1	
DMR8:55910001	8	55910001	1000	1	4.12E-05	0.8799707	10	1	LYN;SNORA1B	
DMR8:60922001	8	60922001	1000	1	7.63E-05	0.5989909	58	5.8		
DMR8:74179001	8	74179001	1000	1	8.84E-05	0.6374046	36	3.6		
DMR8:93501001	8	93501001	1000	1	7.65E-05	-0.8739868	4	0.4	CIBAR1-DT;LOC105375641	
DMR8:97681001	8	97681001	3000	1	4.36E-05	0.8572107	45	1.5	MTDH	
DMR8:128228001	8	128228001	1000	1	5.22E-05	0.9744743	13	1.3	RN7SKP226	
DMR8:143858001	8	143858001	9000	1	1.33E-06	0.6370392	992	11.022222	EPPK1	Cytoskeleton
DMR8:143920001	8	143920001	6000	1	3.25E-05	0.5322273	532	8.8666667	PLEC	Cytoskeleton
DMR9:13653001	9	13653001	1000	1	9.20E-06	-1.0290028	8	0.8		
DMR9:106106001	9	106106001	1000	1	1.59E-05	-1.0462609	6	0.6		
DMR9:112558001	9	112558001	1000	1	4.48E-05	0.8804025	16	1.6	KIAA1958	
DMR9:123681001	9	123681001	1000	1	8.32E-05	0.7420936	7	0.7	DENND1A	
DMR9:134400001	9	134400001	1000	1	4.81E-05	0.6791124	24	2.4	RXRA	Transcription

DMR9:137161001	9	137161001	1000	1	7.65E-05	0.654934	70	7	GRIN1;LOC105376328;LRRC26;MIR3621;TMEM210	Receptor
DMR9:137774001	9	137774001	2000	1	8.50E-08	1.0410925	65	3.25	EHMT1;LOC651337	
DMR9:138150001	9	138150001	1000	1	2.53E-06	0.894349	79	7.9	IL9RP1;TUBBP5	
DMR9:138220001	9	138220001	1000	1	6.43E-05	0.6027703	38	3.8	LOC107987145;FAM157B	
DMR10:321001	10	321001	3000	1	6.45E-05	0.501453	242	8.0666667	DIP2C	
DMR10:2731001	10	2731001	1000	1	5.83E-05	-0.822033	13	1.3		
DMR10:4562001	10	4562001	1000	1	6.57E-05	-0.6355596	10	1		
DMR10:15089001	10	15089001	1000	1	4.11E-06	0.9079027	25	2.5	ACBD7-DCLRE1CP1;ACBD7;GAPDHP45;RPP38-DT;RPP38	Transport
DMR10:29268001	10	29268001	1000	1	6.79E-05	0.8266257	22	2.2		
DMR10:38744001	10	38744001	3000	1	4.96E-05	-0.5666748	12	0.4		
DMR10:42177001	10	42177001	1000	1	8.37E-05	0.4805073	126	12.6		
DMR10:42242001	10	42242001	2000	1	2.01E-05	0.5584126	206	10.3	LOC105378267;LOC101929373	
DMR10:46581001	10	46581001	2000	1	1.78E-05	0.8158303	37	1.85	SYT15;LOC101927699	Transport
DMR10:79296001	10	79296001	1000	1	5.25E-05	0.6792199	24	2.4	ZMIZ1	
DMR10:89018001	10	89018001	1000	1	2.06E-05	0.6745373	26	2.6	FAS	Receptor
DMR10:101829001	10	101829001	1000	1	5.20E-05	0.7454317	56	5.6	KCNIP2-AS1;KCNIP2	
DMR10:102695001	10	102695001	1000	1	7.39E-05	0.6236949	15	1.5	ARL3	
DMR11:10508001	11	10508001	2000	1	8.75E-07	0.7274818	60	3	AMPD3;MTRNR2L8;MIR4485;RNF141	Metabolism
DMR11:45371001	11	45371001	1000	1	5.51E-05	0.9935506	34	3.4	LINC02687	
DMR11:64302001	11	64302001	1000	1	1.65E-05	0.6720071	27	2.7	KCNK4;KCNK4-TEX40;CATSPERZ;ESRRA	Transport
DMR11:65496001	11	65496001	1000	1	7.50E-05	0.920657	24	2.4	LINC02736;MALAT1;TALAM1;MASCRNA	
DMR11:67309001	11	67309001	1000	1	5.24E-05	0.8852935	27	2.7	ANKRD13D;SSH3;LOC100130987	Signaling
DMR11:84308001	11	84308001	1000	1	1.77E-05	0.5621376	46	4.6	DLG2	Cytoskeleton
DMR11:118885001	11	118885001	1000	1	9.43E-05	0.7563378	21	2.1	CXCR5	
DMR11:124244001	11	124244001	1000	1	6.54E-05	-0.6353188	4	0.4	OR8G7P;OR8G1	Receptor
DMR11:124759001	11	124759001	1000	1	3.74E-05	0.9641608	65	6.5	VSIG2;ESAM;ESAM-AS1;MSANTD2	Immune
DMR12:36461001	12	36461001	3000	1	5.82E-05	-0.6983416	47	1.5666667		
DMR12:46213001	12	46213001	1000	1	5.65E-05	-0.7584479	7	0.7	SLC38A1	Transport
DMR12:73002001	12	73002001	3000	1	2.00E-05	-0.5886863	29	0.9666667	LOC105369838	
DMR12:91296001	12	91296001	2000	1	2.70E-05	0.6667256	24	1.2	LOC105369898	
DMR12:111791001	12	111791001	2000	1	3.30E-05	0.7177287	49	2.45	ALDH2;MIR6761	Metabolism
DMR12:131701001	12	131701001	1000	1	4.44E-05	0.5503995	33	3.3	LOC105370087;SFSWAP	Translation
DMR12:132343001	12	132343001	3000	1	5.68E-05	0.7449685	121	4.0333333		
DMR13:18212001	13	18212001	3000	1	5.50E-05	0.5923564	9	0.3	FAM230C;EIF3FP2	
DMR13:46453001	13	46453001	1000	1	2.40E-05	-0.9469366	12	1.2	LINC01198;LOC112268117;OR7E101P	
DMR13:51694001	13	51694001	2000	1	4.56E-05	-0.6870963	27	1.35	WDFY2	
DMR13:87084001	13	87084001	1000	1	1.51E-05	-0.8333306	6	0.6		
DMR13:101300001	13	101300001	2000	1	9.32E-05	-0.6896536	21	1.05	NALCN	Transport
DMR13:109424001	13	109424001	1000	1	5.85E-07	0.8096783	9	0.9		
DMR13:109765001	13	109765001	2000	1	2.87E-06	-1.4365122	17	0.85	IRS2	
DMR13:114075001	13	114075001	2000	1	5.08E-05	0.5688261	155	7.75	RASA3	Signaling
DMR14:23162001	14	23162001	1000	1	4.53E-05	0.8883226	9	0.9	SLC7A8;RNU6-1138P	Transport
DMR14:24260001	14	24260001	1000	1	5.11E-05	0.8583229	20	2	TGM1;RABGGTA	Transport;Metabolism
DMR14:73781001	14	73781001	1000	1	1.09E-05	0.7344218	22	2.2	MIDEAS;LOC100506476	
DMR14:98787001	14	98787001	2000	1	2.49E-06	-0.7573173	27	1.35	LOC105370658	
DMR14:104250001	14	104250001	2000	1	8.62E-06	0.5617382	134	6.7	LINC02691	
DMR15:20100001	15	20100001	6000	2	3.07E-06	0.6865514	54	0.9	RN7SL584P	
DMR15:20340001	15	20340001	6000	1	7.51E-05	0.5547148	66	1.1		
DMR15:21107001	15	21107001	5000	2	1.08E-08	0.8431403	49	0.98	RN7SL400P	
DMR15:24876001	15	24876001	1000	1	7.62E-05	-0.7313973	12	1.2	SNHG14;SNRPN	Translation
DMR15:40961001	15	40961001	1000	1	7.90E-05	0.5946387	48	4.8	CHAC1;LOC105370789	
DMR15:45061001	15	45061001	1000	1	7.53E-05	0.7791061	21	2.1	SORD;LOC100422669	Metabolism
DMR15:52305001	15	52305001	2000	1	9.87E-05	0.8033552	19	0.95	MYO5C;LOC105370819;MYO5A	Cytoskeleton
DMR15:70987001	15	70987001	1000	1	5.26E-05	-0.6793796	10	1	LRRC49	Cytoskeleton
DMR15:90059001	15	90059001	1000	1	9.25E-05	1.0092822	25	2.5	ZNF710	Transcription
DMR15:99436001	15	99436001	1000	1	1.23E-06	0.7786333	23	2.3		
DMR16:807001	16	807001	1000	1	8.62E-05	0.9589616	84	8.4	CHTF18;GNG13;PRR25	Transcription;Signaling
DMR16:4894001	16	4894001	1000	1	7.97E-05	0.6917811	28	2.8	PPL;LOC105371064	Cytoskeleton
DMR16:7650001	16	7650001	1000	1	3.74E-05	-0.6096622	11	1.1	RBFOX1	Translation
DMR16:12613001	16	12613001	2000	1	2.49E-05	0.6623136	35	1.75		
DMR16:24366001	16	24366001	1000	1	4.17E-07	-1.1311264	10	1	CACNG3	Transport

DMR16:27619001	16	27619001	1000	1	2.76E-05	1.0514555	11	1.1	KATNIP	
DMR16:50614001	16	50614001	1000	1	5.48E-05	-0.7290113	8	0.8	NKD1	
DMR16:55331001	16	55331001	1000	1	6.24E-06	0.6277321	56	5.6	IRX6	Development
DMR16:80696001	16	80696001	1000	1	5.29E-05	-0.7794969	5	0.5	CDYL2	
DMR16:87162001	16	87162001	2000	1	2.32E-05	0.5336263	54	2.7		
DMR16:89315001	16	89315001	1000	1	9.39E-06	0.6408912	36	3.6	ANKRD11;LOC100287036	
DMR17:753001	17	753001	1000	1	8.63E-05	0.5043061	50	5	GEMIN4;DBIL5P;GLOD4	
DMR17:1588001	17	1588001	4000	1	2.91E-05	0.7508036	148	3.7	SLC43A2	
DMR17:6470001	17	6470001	1000	1	3.29E-05	0.6452243	29	2.9	PITPNM3	Metabolism
DMR17:22880001	17	22880001	1000	1	9.22E-05	-0.8210642	19	1.9		
DMR17:38510001	17	38510001	1000	1	2.77E-05	0.7152725	145	14.5	ARHGAP23	
DMR17:75857001	17	75857001	1000	1	6.34E-05	0.6045264	32	3.2	WBP2	
DMR17:78126001	17	78126001	1000	1	8.70E-05	0.7977407	27	2.7	TMC6;TMC8	
DMR17:82829001	17	82829001	3000	1	9.95E-05	0.6642136	109	3.6333333	TBCD;ZNF750	Transcription
DMR17:82850001	17	82850001	1000	1	5.65E-05	0.6502566	5	0.5	TBCD;ZNF750	Transcription
DMR18:18585001	18	18585001	1000	1	9.64E-05	-0.8276133	16	1.6		
DMR18:46575001	18	46575001	1000	1	9.57E-05	0.758836	11	1.1	LOXHD1	
DMR18:75204001	18	75204001	2000	1	6.48E-06	0.9192432	137	6.85	ZADH2;TSHZ1	Metabolism;Transcription
DMR18:79617001	18	79617001	1000	1	6.95E-05	0.5899867	96	9.6	LOC105372228	
DMR19:1465001	19	1465001	2000	1	9.65E-05	0.8022891	175	8.75	APC2;C19orf25	
DMR19:4050001	19	4050001	1000	1	8.05E-05	0.7984285	32	3.2	ZBTB7A	Transcription
DMR19:9639001	19	9639001	1000	1	5.41E-05	-0.6412872	6	0.6	ZNF561-AS1;ZNF562	Transcription
DMR19:9736001	19	9736001	1000	1	1.66E-05	0.8548263	17	1.7		
DMR19:14076001	19	14076001	1000	1	6.59E-05	0.7443171	17	1.7	EEF1DP1;MISP3;MIR1199;C19orf67	
DMR19:17642001	19	17642001	1000	1	1.12E-05	-0.5666543	10	1	UNC13A	
DMR19:18386001	19	18386001	1000	1	1.29E-05	1.0088021	40	4	GDF15;MIR3189;LRRC25	Growth Factors
DMR19:19978001	19	19978001	1000	1	5.52E-05	-0.9466035	10	1	BNIP3P12;VN1R77P	
DMR19:21824001	19	21824001	1000	1	8.60E-05	-0.8641387	10	1	ZNF43;BNIP3P27	Transcription
DMR19:30732001	19	30732001	2000	1	3.63E-06	-0.8341187	25	1.25		
DMR19:39075001	19	39075001	1000	1	3.24E-05	0.8092532	18	1.8	ACP7	Signaling
DMR19:50644001	19	50644001	2000	1	4.38E-05	-0.5147226	15	0.75	SYT3;C19orf81	Transport
DMR19:58367001	19	58367001	2000	1	9.90E-06	0.5805546	202	10.1	ZNF497;LOC100419840;ZNF497-AS1;RNA5SP473;ZNF837;LOC105372484	Transcription
DMR20:21422001	20	21422001	1000	1	6.13E-05	0.8479022	6	0.6	LOC105372558;RN7SKP140	
DMR20:24903001	20	24903001	1000	1	5.33E-05	-0.7556059	4	0.4		
DMR20:26208001	20	26208001	1000	1	1.51E-05	0.5586071	115	11.5	MIR663AHG;MIR663A	
DMR20:46724001	20	46724001	1000	1	9.96E-05	-0.5232627	1	0.1	SLC2A10	
DMR21:8388001	21	8388001	16000	1	8.98E-05	0.3917041	2144	13.4	MIR6724-3;RNA45SN3;RNA18SN3;RNA5-8SN3;RNA28SN3;LOC105379508	
DMR21:10325001	21	10325001	2000	1	3.31E-06	0.7726074	11	0.55	EIF3FP1	
DMR21:36751001	21	36751001	1000	1	5.30E-05	0.8390528	17	1.7	SIM2;HLCS	Transcription;Metabolism
DMR21:41712001	21	41712001	1000	1	5.93E-05	0.7937168	29	2.9	LINC00479;LINC00112	
DMR21:42848001	21	42848001	2000	1	2.33E-05	0.5587678	153	7.65	WDR4	Translation
DMR22:10639001	22	10639001	3000	1	2.37E-05	-0.5836583	11	0.3666667		
DMR22:11430001	22	11430001	2000	1	5.90E-06	-0.8143191	17	0.85		
DMR22:16558001	22	16558001	4000	1	6.71E-05	0.7420686	32	0.8		
DMR22:18896001	22	18896001	1000	1	2.90E-06	0.6253853	11	1.1	FAM230F;DGCR6	
DMR22:29598001	22	29598001	1000	1	9.67E-05	0.5736335	60	6	NF2;RPEP4	Cytoskeleton
DMR22:30236001	22	30236001	1000	1	6.17E-05	0.8276158	13	1.3	LIF-AS1;LIF;LIF-AS2	Growth Factors
DMR22:35974001	22	35974001	1000	1	7.10E-05	0.8279914	19	1.9	RBFOX2	Translation
DMR22:38190001	22	38190001	1000	1	8.55E-06	0.9639368	16	1.6	PLA2G6	Metabolism
DMR22:38644001	22	38644001	2000	1	6.22E-05	-0.7244097	51	2.55	FAM227A	
DMR22:49630001	22	49630001	1000	1	9.82E-05	0.60846	30	3	MIR3667HG	
DMRMT:1	MT	1	16569	3	4.71E-08	0.7588823	435	2.6253848		
DMRX:811001	X	811001	1000	1	8.45E-05	-0.6603685	4	0.4		
DMRX:1519001	X	1519001	2000	1	6.64E-06	-0.642526	5	0.25	P2RY8	Signaling
DMRX:3555001	X	3555001	3000	1	4.16E-06	-0.5932161	59	1.9666667		
DMRX:6704001	X	6704001	2000	1	6.93E-05	-0.5669401	20	1		
DMRX:8566001	X	8566001	2000	1	7.04E-05	-0.9506594	28	1.4	ANOS1	Extracellular Matrix
DMRX:17531001	X	17531001	1000	1	4.21E-05	-0.8330937	6	0.6	NHS;LOC101928389	
DMRX:17770001	X	17770001	5000	1	2.36E-05	-0.6119217	35	0.7	FAM136GP	
DMRX:18689001	X	18689001	1000	1	9.76E-05	-0.7090139	19	1.9	PPEF1;PPEF1-AS1	Signaling
DMRX:19106001	X	19106001	1000	1	3.33E-05	-0.629619	19	1.9	ADGRG2	Signaling
DMRX:20810001	X	20810001	1000	1	6.73E-05	-0.7995027	2	0.2		
DMRX:36766001	X	36766001	2000	1	3.27E-05	-0.9125538	10	0.5		

DMRX:40151001	X	40151001	2000	1	6.97E-05	0.6129392	118	5.9	BCOR	
DMRX:47009001	X	47009001	1000	1	8.04E-05	-0.9779042	14	1.4	JADE3	Transcription
DMRX:47632001	X	47632001	3000	1	8.90E-05	-0.6224979	50	1.6666667	CFP;ELK1	Transcription
DMRX:48789001	X	48789001	3000	1	3.96E-05	-0.569799	41	1.3666667	GATA1;HDAC6	Transcription
DMRX:48912001	X	48912001	1000	1	7.99E-05	-0.844148	29	2.9	PQBP1;SLC35A2;PIM2;OTUD5	Cytoskeleton;Transport;Signaling;Protease
DMRX:52816001	X	52816001	1000	1	9.16E-05	-0.971397	14	1.4	XAGE5;LOC107985641	
DMRX:54415001	X	54415001	1000	1	4.49E-05	-0.657834	25	2.5		
DMRX:64849001	X	64849001	1000	1	9.57E-07	-0.8363688	5	0.5	LOC105373239	
DMRX:65318001	X	65318001	1000	1	6.36E-05	-0.8282763	10	1	ZC3H12B;MORF4L1P5	Translation
DMRX:72558001	X	72558001	2000	1	8.03E-05	-0.8674946	68	3.4	HDAC8	
DMRX:75634001	X	75634001	3000	1	2.36E-05	-0.609356	32	1.0666667	LOC107985664	
DMRX:75781001	X	75781001	1000	1	5.05E-06	-1.083344	19	1.9	LOC107985664;MAGEE2	Cytoskeleton
DMRX:82032001	X	82032001	1000	1	3.27E-05	-0.8960747	15	1.5		
DMRX:82266001	X	82266001	1000	1	3.02E-05	-0.7243944	10	1		
DMRX:87709001	X	87709001	1000	1	2.20E-05	-0.7985708	4	0.4	RPSAP15	
DMRX:91194001	X	91194001	1000	1	1.03E-05	-0.9100044	8	0.8		
DMRX:98208001	X	98208001	1000	1	4.81E-05	-1.0005179	7	0.7		
DMRX:100787001	X	100787001	3000	1	2.82E-05	-0.8187662	30	1		
DMRX:113586001	X	113586001	1000	1	3.55E-05	-0.8360888	17	1.7		
DMRX:121344001	X	121344001	5000	1	8.35E-05	-0.5863148	76	1.52	LOC112268302	
DMRX:130498001	X	130498001	5000	1	5.57E-05	-0.5515805	49	0.98	DENND10P1	
DMRX:131807001	X	131807001	2000	1	3.76E-05	-0.7638917	39	1.95	FIRRE;PNKDP1	
DMRX:139677001	X	139677001	1000	1	2.87E-05	-0.8764973	12	1.2	MCF2	Transcription
DMRX:147990001	X	147990001	2000	1	3.13E-05	-0.7791639	11	0.55	FMR1NB	
DMRX:148232001	X	148232001	1000	1	9.83E-05	-0.8821559	4	0.4		
DMRX:152488001	X	152488001	1000	1	2.33E-05	-0.8977671	5	0.5	KRT8P8	
DMRX:153306001	X	153306001	1000	1	7.48E-05	-0.8593804	17	1.7		
DMRX:153586001	X	153586001	1000	1	8.91E-05	-0.7443371	18	1.8	ATP2B3;CCNQ	Transport;Signaling
DMRY:6265001	Y	6265001	1000	1	5.33E-17	4.5645424	55	5.5	FAM197Y9;TSPY11P	
DMRY:9336001	Y	9336001	2000	2	1.27E-13	3.8725112	114	5.7	RBMY1GP;TTTY20;TSPY4	Epigenetic
DMRY:9342001	Y	9342001	1000	1	6.44E-08	2.2633671	26	2.6	TTTY20;TSPY4;FAM197Y8	Epigenetic
DMRY:9356001	Y	9356001	1000	1	8.45E-29	5.5924631	59	5.9	FAM197Y8;TSPY8	Epigenetic
DMRY:9358001	Y	9358001	1000	1	5.09E-16	3.9967251	43	4.3	FAM197Y8;TSPY8	Epigenetic
DMRY:9378001	Y	9378001	1000	1	5.10E-18	4.8620965	61	6.1	FAM197Y7;TSPY7P	
DMRY:9397001	Y	9397001	2000	2	4.41E-16	4.4274475	110	5.5	FAM197Y6;TSPY3	Epigenetic
DMRY:9465001	Y	9465001	1000	1	4.85E-22	5.7083062	51	5.1	FAM197Y5;TSPY1	Epigenetic
DMRY:9487001	Y	9487001	1000	1	4.41E-17	4.5257484	61	6.1	FAM197Y4;TSPY9P	Epigenetic
DMRY:9492001	Y	9492001	1000	1	5.83E-05	1.6675052	24	2.4	FAM197Y4;TSPY9P;FAM197Y3	Epigenetic
DMRY:9526001	Y	9526001	1000	1	8.74E-13	3.6669895	57	5.7	FAM197Y2;TSPY10	Epigenetic
DMRY:9528001	Y	9528001	1000	1	6.54E-16	4.5726445	46	4.6	FAM197Y2;TSPY10	Epigenetic
DMRY:11530001	Y	11530001	63000	7	7.58E-07	1.2715348	1725	2.7380952		
DMRY:15896001	Y	15896001	1000	1	6.46E-05	0.888468	15	1.5		
DMRY:26656001	Y	26656001	17000	1	7.40E-05	0.775486	322	1.8941176		
DMRY:56673001	Y	56673001	99000	46	3.77E-07	1.1852513	2798	2.8262626		

Supplemental Table S9
Male WGCNA Modules
(A) Dark Orange

Chr	Start	Stop	Annotation
1	2688001	2689000	TTC34
2	1656001	1657000	PXDN
4	9210001	9211000	USP17L10;USP17L11;USP17L12
4	9212001	9219000	USP17L10;USP17L11;USP17L12;USP17L10;USP17L11;USP17L12;USP17L10;USP17L11;USP17L12;USP17L13;USP17L10;USP17L11;USP17L12;USP17L13;USP17L10;USP17L11;USP17L12;USP17L13;USP17L10;USP17L11;USP17L12;USP17L13;USP17L10;USP17L11;USP17L12;USP17L13
4	9221001	9252000	USP17L10;USP17L11;USP17L12;USP17L13;USP17L14P;USP17L10;USP17L11;USP17L12;USP17L13;USP17L14P;USP17L11;USP17L12;USP17L13;USP17L14P;USP17L11;USP17L12;USP17L13;USP17L14P;USP17L15;USP17L11;USP17L12;USP17L13;USP17L14P;USP17L15;USP17L11;USP17L12;USP17L13;USP17L14P;USP17L15;USP17L12;USP17L13;USP17L14P;USP17L15;USP17L12;USP17L13;USP17L14P;USP17L15;USP17L12;USP17L13;USP17L14P;USP17L15;USP17L16P;USP17L12;USP17L13;USP17L14P;USP17L15;USP17L16P;USP17L12;USP17L13;USP17L14P;USP17L15;USP17L16P;USP17L13;USP17L14P;USP17L15;USP17L16P;USP17L13;USP17L14P;USP17L15;USP17L16P;USP17L17;USP17L13;USP17L14P;USP17L15;USP17L16P;USP17L17;USP17L13;USP17L14P;USP17L15;USP17L16P;USP17L17;USP17L13;USP17L14P;USP17L15;USP17L16P;USP17L17;USP17L14P;USP17L15;USP17L16P;USP17L17;USP17L14P;USP17L15;USP17L16P;USP17L17;USP17L18;USP17L14P;USP17L15;USP17L16P;USP17L17;USP17L18;USP17L14P;USP17L15;USP17L16P;USP17L17;USP17L18;USP17L14P;USP17L15;USP17L16P;USP17L17;USP17L18;USP17L15;USP17L16P;USP17L17;USP17L18;USP17L15;USP17L16P;USP17L17;USP17L18;USP17L19;USP17L15;USP17L16P;USP17L17;USP17L18;USP17L19;USP17L15;USP17L16P;USP17L17;USP17L18;USP17L19;USP17L15;USP17L16P;USP17L17;USP17L18;USP17L19;USP17L16P;USP17L17;USP17L18;USP17L19;USP17L16P;USP17L17;USP17L18;USP17L19;USP17L20;USP17L16P;USP17L17;USP17L18;USP17L19;USP17L20;USP17L16P;USP17L17;USP17L18;USP17L19;USP17L20;USP17L17;USP17L18;USP17L19;USP17L20
4	9254001	9273000	USP17L17;USP17L18;USP17L19;USP17L20;USP17L21;USP17L17;USP17L18;USP17L19;USP17L20;USP17L21;USP17L18;USP17L19;USP17L20;USP17L21;USP17L18;USP17L19;USP17L20;USP17L21;USP17L22;USP17L18;USP17L19;USP17L20;USP17L21;USP17L22;USP17L18;USP17L19;USP17L20;USP17L21;USP17L22;USP17L18;USP17L19;USP17L20;USP17L21;USP17L22;USP17L19;USP17L20;USP17L21;USP17L22;USP17L19;USP17L20;USP17L21;USP17L22;USP17L19;USP17L20;USP17L21;USP17L22;USP17L19;USP17L20;USP17L21;USP17L22;USP17L20;USP17L21;USP17L22;USP17L20;USP17L21;USP17L22;USP17L20;USP17L21;USP17L22;USP17L20;USP17L21;USP17L22;USP17L20;USP17L21;USP17L22;USP17L21;USP17L22;USP17L21;USP17L22;USP17L21;USP17L22
4	9323001	9356000	USP17L24;USP17L25;USP17L24;USP17L25;USP17L26;USP17L24;USP17L25;USP17L26;USP17L24;USP17L25;USP17L26;USP17L24;USP17L25;USP17L26;USP17L24;USP17L25;USP17L26;USP17L24;USP17L25;USP17L26;USP17L5;USP17L24;USP17L25;USP17L26;USP17L5;USP17L24;USP17L25;USP17L26;USP17L5;USP17L24;USP17L25;USP17L26;USP17L5;USP17L24;USP17L25;USP17L26;USP17L5;USP17L24;USP17L25;USP17L26;USP17L5;USP17L27;USP17L24;USP17L25;USP17L26;USP17L5;USP17L27;USP17L24;USP17L25;USP17L26;USP17L5;USP17L27;USP17L25;USP17L26;USP17L5;USP17L27;USP17L25;USP17L26;USP17L5;USP17L27;USP17L28;USP17L25;USP17L26;USP17L5;USP17L27;USP17L28;USP17L25;USP17L26;USP17L5;USP17L27;USP17L28;USP17L25;USP17L26;USP17L5;USP17L27;USP17L28;USP17L26;USP17L5;USP17L27;USP17L28;USP17L26;USP17L5;USP17L27;USP17L28;USP17L29;USP17L26;USP17L5;USP17L27;USP17L28;USP17L29;USP17L26;USP17L5;USP17L27;USP17L28;USP17L29;USP17L26;USP17L5;USP17L27;USP17L28;USP17L29;USP17L5;USP17L27;USP17L28;USP17L29;USP17L5;USP17L27;USP17L28;USP17L29;USP17L9P;USP17L5;USP17L27;USP17L28;USP17L29;USP17L9P;USP17L5;USP17L27;USP17L28;USP17L29;USP17L9P;USP17L27;USP17L28;USP17L29;USP17L9P;USP17L27;USP17L28;USP17L29;USP17L9P;USP17L27;USP17L28;USP17L29;USP17L9P;USP17L30;USP17L27;USP17L28;USP17L29;USP17L9P;USP17L30;USP17L27;USP17L28;USP17L29;USP17L9P;USP17L30
4	9357001	9369000	USP17L28;USP17L29;USP17L9P;USP17L30;USP17L6P;USP17L28;USP17L29;USP17L9P;USP17L30;USP17L6P;USP17L28;USP17L29;USP17L9P;USP17L30;USP17L6P;USP17L28;USP17L29;USP17L9P;USP17L30;USP17L6P;USP17L29;USP17L9P;USP17L30;USP17L6P;USP17L29;USP17L9P;USP17L30;USP17L6P;USP17L29;USP17L9P;USP17L30;USP17L6P;USP17L29;USP17L9P;USP17L30;USP17L6P;USP17L29;USP17L9P;USP17L30;USP17L6P;USP17L9P;USP17L30;USP17L6P;USP17L9P;USP17L30;USP17L6P;USP17L9P;USP17L30;USP17L6P
6	167940001	167941000	AFDN;LOC107986673
7	102665001	102666000	POLR2J2;SPDYE2B
19	55850001	55851000	NLRP4
20	29883001	29884000	RNA5SP532;DUX4L37
20	47222001	47223000	ZMYND8
22	49494001	49495000	MIR3667HG;LOC105373087

(B) Dark Turquoise

Chr	Start	Stop	Annotation
1	2677001	2678000	TTC34
1	2682001	2683000	TTC34
1	17220001	17221000	PADI1
1	39102001	39103000	MACF1
1	121413001	121414000	LINC02798
1	161416001	161417000	LOC148430;LOC102724602;TRUND-NNN3-1;TRE-TTC4-2
1	174665001	174666000	RABGAP1L
1	226535001	226536000	LOC105373116
2	18802001	18803000	LOC105373456
2	55465001	55466000	RNU6-221P
2	108646001	108647000	LIMS1
2	113973001	113974000	LINC01191;LOC100499194
2	158513001	158514000	PKP4
3	10329001	10330000	SEC13;ATP2B2;MIR378B
3	39203001	39204000	DSTNP4
3	97274001	97275000	EPHA6
3	136101001	136102000	PPP2R3A
3	138126001	138127000	A4GNT
3	196418001	196419000	UBXN7
4	11383001	11384000	RNPS1P1;HS3ST1
4	151406001	151407000	FHIP1A-DT;FHIP1A
4	190178001	190179000	DUX4
4	190181001	190182000	DUX4
5	69914001	69915000	GUSBP13
6	7437001	7438000	LOC102724234
6	26902001	26903000	POM121L6P;GUSBP2;LOC112267954
6	33346001	33347000	MYL12BP3
6	64295001	64296000	EYS
6	154391001	154392000	CNKSR3
6	160633001	160634000	LPA
8	74664001	74665000	MIR2052HG

8	85761001	85762000	REXO1L9P
8	85785001	85786000	REXO1L2P
8	85797001	85798000	REXO1L4P
8	85812001	85813000	REXO1L4P;REXO1L5P
8	85825001	85826000	REXO1L5P;REXO1L6P
9	35713001	35714000	TLN1;MIR6852
9	41756001	41757000	LOC105376063
9	69206001	69207000	TJP2
9	113440001	113441000	RGS3
9	130363001	130364000	HMCN2
9	131891001	131892000	MED27
10	32817001	32818000	CCDC7
10	84354001	84355000	CCSER2
10	86564001	86565000	LOC105378407;LOC105378406
10	100381001	100382000	OLMALINC
10	101653001	101654000	FBXW4
10	103725001	103726000	SH3PXD2A
10	133125001	133126000	ADGRA1
10	133688001	133689000	DUX4L22;DUX4L21;DUX4L20
11	1739001	1740000	IFITM10
11	20570001	20571000	LOC105376584;HMGB1P40
11	59791001	59792000	STX3;FABP5P7
12	10707001	10708000	YBX3
12	116735001	116736000	SPRING1;RNFT2
12	131842001	131843000	MMP17
13	31169001	31170000	HSPH1;LOC105370148
14	22741001	22742000	OXA1L-DT
15	89807001	89808000	ANPEP
16	3345001	3346000	OR2C1
16	8835001	8836000	PMM2
16	78443001	78444000	WWOX
16	89820001	89821000	FANCA;SPIRE2
17	36394001	36395000	LOC100420852;TBC1D3H;RNU6-1192P
17	43391001	43392000	LINC00910;RNU2-4P;RNU6-1137P;ARL4D
18	106001	107000	ROCK1P1;MIR8078
18	108001	110000	ROCK1P1;MIR8078;ROCK1P1;MIR8078
19	38236001	38237000	DPF1
19	46421001	46422000	CCDC8;PNMA8C
19	50103001	50104000	SNAR-A14;SNAR-A5;SNAR-A6;SNAR-A7;SNAR-A8
20	23127001	23128000	LINC00656
22	11936001	11937000	LOC102723769
22	28178001	28179000	TTC28
22	32769001	32770000	SYN3
22	34961001	34962000	LINC02885;LOC105373016;LOC105373015
22	41990001	41991000	SEPTIN3;WBP2NL;SLC25A5P1
X	155950001	155951000	VAMP7
Y	11305001	11306000	DUX4L16;DUX4L17
Y	11319001	11320000	DUX4L17;DUX4L18
Y	11324001	11326000	DUX4L17;DUX4L18;DUX4L19;DUX4L17;DUX4L18;DUX4L19
Y	11329001	11331000	DUX4L18;DUX4L19;PABPC1P5;DUX4L18;DUX4L19;PABPC1P5;SLC9B1P1
Y	56847001	56848000	CTBP2P1
Y	56850001	56851000	CTBP2P1

(C) Green Yellow

Chr	Start	Stop	Annotation
1	189001	190000	LOC100996442;DDX11L17;WASH9P;MIR6859-2
1	629001	635000	LOC101928626;MTND1P23;MTND2P28;MTCO1P12;MIR12136;MTCO2P12;MTATP8P1;MTATP6P1;MTCO3P12;LOC101928626;MTND1P23;MTND2P28;MTCO1P12;MIR12136;MTCO2P12;MTATP8P1;MTATP6P1;MTCO3P12;LOC101928626;MTND1P23;MTND2P28;MTCO1P12;MIR12136;MTCO2P12;MTATP8P1;MTATP6P1;MTCO3P12;LOC101928626;MTND1P23;MTND2P28;MTCO1P12;MIR12136;MTCO2P12;MTATP8P1;MTATP6P1;MTCO3P12;LOC101928626;MTND1P23;MTND2P28;MTCO1P12;MIR12136;MTCO2P12;MTATP8P1;MTATP6P1;MTCO3P12;LOC101928626;MTND1P23;MTND2P28;MTCO1P12;MIR12136;MTCO2P12;MTATP8P1;MTATP6P1;MTCO3P12
1	1065001	1066000	AGRN;LOC100288175;LOC105378948;RNF223
1	1566001	1567000	SSU72
1	1733001	1734000	CDK11A;SLC35E2A;LOC100129381
1	2516001	2517000	PANK4
1	3659001	3660000	WRAP73;TP73;TP73-AS3
1	3947001	3948000	LINC01346;LINC01345
1	4666001	4667000	AJAP1
1	5912001	5913000	NPHP4
1	6560001	6561000	NOL9;TAS1R1;LOC107984912
1	6966001	6967000	CAMTA1
1	7037001	7038000	CAMTA1
1	7234001	7235000	CAMTA1;LOC105376692
1	9247001	9248000	H6PD
1	11430001	11431000	MTCYBP45
1	12308001	12309000	VPS13D
1	13500001	13501000	LRRC38;LOC102724856
1	16580001	16581000	NBPF1
1	16694001	16695000	EIF1AXP1;ESPNP
1	18080001	18081000	LINC01654
1	18861001	18862000	TAS1R2;ALDH4A1
1	21263001	21264000	ECE1
1	22264001	22265000	LOC107985377;MIR4418
1	24122001	24123000	MYOM3;IL22RA1
1	24167001	24168000	IFNLR1
1	24959001	24960000	RUNX3;RUNX3-AS1
1	25574001	25575000	LDLRAP1
1	27232001	27233000	LOC105376890;WDTC1
1	39557001	39558000	PPIEL;PABPC4;PABPC4-AS1;SNORA55
1	39812001	39813000	LOC105378951;LINC02811;LOC105378665

1	43986001	43987000	ATP6V0B;B4GALT2;CCDC24;SLC6A9
1	44118001	44119000	KLF17;RN7SL479P;LOC100129492
1	47701001	47702000	LINC01738
1	48089001	48090000	LINC02794;CYP46A4P
1	55003001	55004000	BSND
1	65137001	65138000	AK4
1	67695001	67696000	GADD45A;GNG12
1	85031001	85032000	MCOLN3
1	85927001	85928000	COL24A1
1	89983001	89984000	LOC105378847;RN7SKP272;GEMIN8P4
1	92964001	92965000	DIPK1A;RNU6-970P;RN7SL692P
1	111689001	111690000	RAP1A
1	112371001	112372000	TXNP3
1	114488001	114489000	TRIM33
1	148124001	148125000	NBPF11;PFN1P4
1	148922001	148923000	PDE4DIP
1	151241001	151242000	PIP5K1A
1	153620001	153621000	S100A16;S100A14;S100A13;S100A1
1	156765001	156766000	PRCC
1	156996001	156997000	ARHGEF11;RN7SL612P
1	161148001	161149000	UFC1
1	161667001	161668000	FCGR2B
1	168147001	168148000	GPR161
1	173325001	173326000	TNFSF4;LOC100506023
1	173740001	173741000	KLHL20;RPS27P7
1	175995001	175996000	COP1
1	178040001	178041000	CRYZL2P-SEC16B;CRYZL2P
1	179093001	179094000	TOR3A;ABL2
1	181833001	181834000	RN7SKP229
1	183107001	183108000	LAMC1
1	203515001	203516000	OPTC;LOC105371688
1	205627001	205628000	ELK4;LOC100420878
1	210997001	210998000	KCNH1
1	211815001	211816000	LPGAT1
1	213628001	213629000	LOC105372912
1	214068001	214069000	LINC02775
1	219229001	219230000	LYPLAL1
1	229413001	229414000	RN7SKP276
1	231574001	231575000	TSNAX-DISC1;TSNAX
1	233318001	233319000	MAP3K21
1	242300001	242301000	PLD5
1	245543001	245544000	KIF26B
1	246419001	246420000	SMYD3
1	246979001	246980000	ZNF695;ZNF670-ZNF695
1	247521001	247522000	GCSAML;OR2C3;GCSAML-AS1
1	248922001	248923000	PGBD2;RNU6-1205P
2	217001	218000	SH3YL1
2	1080001	1081000	SNTG2
2	1289001	1290000	SNTG2
2	1555001	1556000	LOC102723730
2	2668001	2669000	LOC107985839
2	7878001	7879000	LOC101929861;LOC105373409;LOC101929551
2	10762001	10763000	ATP6V1C2;LOC105373428
2	11614001	11615000	GREB1
2	25916001	25917000	KIF3C
2	28042001	28043000	BABAM2
2	28589001	28590000	PLB1
2	37635001	37636000	CDC42EP3
2	37940001	37941000	RMDN2;RMDN2-AS1
2	39122001	39123000	SOS1
2	42278001	42279000	EML4;LOC107985874
2	44760001	44761000	CAMKMT
2	45737001	45738000	PRKCE
2	49082001	49083000	FSHR
2	64716001	64717000	SERTAD2
2	86930001	86931000	RGPD1;WBP1P1;NDUFB4P5
2	87824001	87826000	RGPD2;MTATP8P2;MTATP6P28;RGPD2;MTATP8P2;MTATP6P28
2	110693001	110694000	LOC105373554
2	111322001	111323000	MIR4435-2HG;MIR4435-2
2	119174001	119175000	RN7SL468P;LOC107985941
2	128500001	128501000	LOC105373611
2	130346001	130347000	TRE-TTC1-1;CCDC115;IMP4;PTPN18
2	131076001	131077000	FAM168B
2	148881001	148882000	USP8P2;KIF5C;KIF5C-AS1
2	152399001	152400000	FMNL2;NUDCP1
2	158861001	158862000	OR7E89P;OR7E28P
2	168704001	168705000	CERS6
2	170547001	170548000	MYO3B
2	171354001	171355000	METTL8
2	185552001	185553000	ELF2P4
2	200807001	200808000	BZW1-AS1;BZW1
2	202309001	202310000	NOP58
2	215545001	215546000	LOC102724861
2	216263001	216264000	MARCHF4
2	218348001	218349000	PNKD;CATIP-AS2;MIR6810;RPL19P5;CATIP
2	218720001	218721000	TTLL4
2	219519001	219520000	ASIC4-AS1;ASIC4

2	220081001	220082000	LOC105373893
2	235844001	235845000	AGAP1
2	235965001	235966000	AGAP1
2	236204001	236205000	ASB18;RNU1-31P
2	238110001	238111000	ESPNL
3	4273001	4274000	SUMF1
3	8717001	8718000	SSUH2
3	12032001	12033000	SYN2
3	12909001	12910000	LOC105376956;IQSEC1
3	32783001	32784000	CNOT10;RPL23AP43
3	43066001	43067000	GASK1A
3	47196001	47197000	KIF9-AS1
3	47597001	47598000	SMARCC1
3	49893001	49894000	MST1R;LOC102724438
3	53808001	53809000	CACNA1D;CHDH
3	57589001	57590000	PDE12;ARF4;ARF4-AS1
3	58374001	58375000	PXK
3	96617001	96619000	RNU6-1094P;MTRNR2L12;RPL18AP8;RNU6-1094P;MTRNR2L12;RPL18AP8
3	118718001	118719000	LOC105374060;LOC107983969
3	119109001	119110000	IGSF11
3	126318001	126319000	KLF15;LOC105374088
3	126721001	126722000	CHCHD6
3	127622001	127623000	MCM2;LOC107986126;PODXL2
3	128565001	128566000	LINC01565
3	128850001	128851000	MARK2P17;MIR12124;MARK3P3;MARK2P6
3	131119001	131120000	NEK11
3	136426001	136427000	STAG1;RNU6-1284P
3	136867001	136868000	NCK1-DT;NCK1
3	153240001	153241000	LOC105374164
3	174371001	174372000	RPL8P4
3	180800001	180801000	LOC101928882
3	183303001	183304000	MCF2L2;LOC107986160;LOC107986161
3	184188001	184189000	AP2M1;ABCF3
3	185003001	185004000	VPS8
3	194302001	194303000	LINC00887
3	195249001	195250000	XXYLT1
3	195808001	195809000	MUC4
3	196190001	196191000	LOC105374303;ZDHHC19
3	196540001	196541000	RPS29P3
3	196636001	196637000	LINC01063;NRROS
4	40001	41000	LOC105374377
4	508001	509000	ZNF721;PIGG
4	1494001	1495000	LOC105374348
4	3529001	3530000	LRPAP1
4	3594001	3595000	LINC00955
4	5594001	5595000	EVC2
4	7604001	7605000	SORCS2
4	7623001	7624000	SORCS2
4	7745001	7746000	SORCS2;AFAP1-AS1
4	8448001	8449000	ACOX3;LOC105374376;TRMT44
4	8486001	8487000	TRMT44
4	25919001	25920000	LOC105374538;SMIM20
4	38080001	38081000	TBC1D1
4	56479001	56480000	SRP72
4	68034001	68035000	POLR2MP1
4	82530001	82531000	TMEM150C
4	84301001	84302000	LOC101928978
4	88925001	88926000	FAM13A;LOC105377327
4	94157001	94158000	SMARCAD1-DT
4	121921001	121922000	TRPC3
4	128081001	128082000	LARP1B
4	128084001	128085000	LARP1B
4	139102001	139103000	ELF2;RN7SL382P
4	150737001	150738000	LRBA
4	160549001	160550000	LINC02477
4	174781001	174782000	GLRA3
4	182379001	182380000	TENM3
4	184172001	184173000	ENPP6
4	186031001	186032000	RNU4-64P
4	186700001	186701000	FAT1
5	280001	281000	PDCD6-DT;PDCD6-AHRR;PDCD6;LOC100421419
5	507001	508000	SLC9A3
5	1815001	1816000	NDUFS6
5	2706001	2707000	LOC107986398;IRX2;LSINCT5
5	6728001	6729000	TENT4A
5	11880001	11881000	CTNND2
5	21494001	21495000	GUSBP1
5	31716001	31717000	PDZD2
5	34185001	34186000	C1QTNF3;LOC643373;LOC646652
5	56258001	56259000	RNA5SP185
5	70051001	70052000	SERF1B;LOC107986372;SMN2
5	70204001	70205000	GUSBP14;LOC643367
5	75480001	75481000	CERT1
5	80650001	80652000	DHFR;MTRNR2L2;MSH3;DHFR;MTRNR2L2;MSH3
5	88379001	88380000	TMEM161B-DT;RPS3AP22
5	112399001	112400000	EPB41L4A
5	121995001	121996000	SRFBP1

5	132230001	132231000	P4HA2
5	132606001	132607000	RAD50
5	134923001	134929000	PCBD2;MTCYBP18;MTND6P4;MTND5P11;MTND4P12;MTND3P25;PCBD2;MTCYBP18;MTND6P4;MTND5P11;MTND4P12;MTND3P25;PCBD2;MTCYBP18;MTND6P4;MTND5P11;MTND4P12;MTND3P25;PCBD2;MTCYBP18;MTND6P4;MTND5P11;MTND4P12;MTND3P25;PCBD2;MTCYBP18;MTND6P4;MTND5P11;MTND4P12;MTND3P25;PCBD2;MTCYBP18;MTND6P4;MTND5P11;MTND4P12;MTND3P25
5	150605001	150606000	SYNPO
5	166129001	166130000	LOC107986370
5	176556001	176557000	CDHR2;RN7SL684P
5	177207001	177208000	NSD1
5	178313001	178314000	COL23A1
5	179296001	179297000	ADAMTS2
5	179631001	179632000	HNRNPH1;C5orf60
5	181324001	181325000	LOC105377771;LOC100132062;CICP15;SEPTIN14P5
6	7803001	7804000	BMP6
6	9867001	9868000	OFCC1
6	21890001	21891000	CASC15
6	25023001	25024000	RIPOR2;LOC102724765;ASS1P1;RN7SL334P
6	26210001	26211000	RPS10P1;H4C5;H2BC8;H2AC8;H3C5P
6	27828001	27829000	H4C11;H4C12;H2AC15;H2BC15
6	28960001	28961000	TRK-TTT3-3;TRM-CAT3-2;KRT18P1
6	30250001	30251000	HCG17;TRIM26BP;HLA-L
6	30596001	30597000	ABCF1;PPP1R10
6	31646001	31647000	PRRC2A;BAG6;APOM
6	31993001	31994000	C4A
6	33195001	33196000	COL11A2;RXRB;RNY4P10;SLC39A7;HSD17B8
6	35131001	35132000	TCP11
6	35567001	35568000	LOC101929309;FKBP5
6	35957001	35958000	SLC26A8
6	38165001	38166000	BTBD9
6	38863001	38864000	DNAH8
6	43151001	43152000	PTK7
6	56463001	56464000	DST
6	57534001	57535000	PRIM2
6	87676001	87677000	ORC3;AKIRIN2
6	107089001	107090000	BEND3
6	122863001	122864000	ATP5MGP2
6	151416001	151417000	RMND1;HSPA8P15
6	160639001	160640000	LPA
6	161610001	161611000	PRKN
6	167173001	167174000	LOC105378123;TCP10L2
6	167668001	167669000	LINC02538
6	168278001	168279000	CTAGE13P
6	168590001	168591000	SMOC2
7	222001	223000	FAM20C
7	327001	328000	LOC105375118
7	2773001	2774000	AMZ1;GNA12
7	5931001	5932000	CCZ1;LOC107986694;RSPH10B
7	6333001	6334000	FAM220A
7	27875001	27876000	JAZF1
7	30506001	30507000	GGCT
7	31014001	31015000	LOC105375222;LOC105375221
7	37169001	37170000	ELMO1
7	55927001	55928000	ZNF713
7	57404001	57405000	LOC105375299;LOC100653233;MIR3147
7	65839001	65840000	LOC84214
7	66632001	66633000	KCTD7
7	73174001	73175000	GTF2IP4
7	73697001	73698000	BUD23;STX1A;LOC105375350
7	73726001	73727000	STX1A;RN7SL265P;ABHD11-AS1;ABHD11
7	73767001	73768000	CLDN3
7	75125001	75126000	GTF2IRD2B
7	91938001	91939000	AKAP9
7	94797001	94798000	RN7SKP129
7	98386001	98387000	BAIAP2L1;RPS3AP26
7	100320001	100321000	SPDYE3;PMS2P1
7	100351001	100352000	STAG3L5P-PVRIG2P-PILRB;STAG3L5P;PVRIG2P;MIR6840;PILRB
7	100745001	100746000	ZAN
7	102162001	102163000	CUX1
7	107864001	107865000	LOC105375444;LOC105375445
7	120782001	120783000	TSPAN12
7	129869001	129870000	UBE2H
7	130819001	130820000	LOC105375508;LOC105375509;LOC347674;H4P1
7	131877001	131878000	LOC107986849
7	138545001	138546000	TRIM24
7	139568001	139569000	HIPK2
7	140466001	140467000	MKRN1
7	141925001	141926000	OR9A4;CLEC5A
7	149621001	149622000	ZNF767P;TRC-GCA19-1
7	149906001	149907000	ACTR3C
7	150960001	150961000	KCNH2
7	153530001	153531000	LOC107986750
7	157752001	157753000	PTPRN2
8	2142001	2143000	MYOM2
8	6734001	6735000	AGPAT5
8	8781001	8782000	MFHAS1
8	9715001	9716000	TNKS
8	10430001	10431000	MSRA

8	11777001	11778000	NEIL2;LOC105379243
8	22083001	22084000	DMTN;FHIP2B
8	22205001	22206000	BMP1
8	22240001	22241000	PHYHIP;MIR320A;POLR3D
8	22439001	22440000	SLC39A14;RNU6-336P;PPP3CC
8	23774001	23775000	LOC107986930
8	27554001	27555000	EPHX2
8	27948001	27949000	SCARA5
8	37788001	37789000	PLPBP;LOC105379381;ADGRA2
8	38422001	38423000	FGFR1
8	42434001	42435000	SLC20A2
8	42731001	42732000	CHRNB3;LOC100288666
8	47996001	47997000	TCONS_00068220;UBE2V2
8	55365001	55366000	XKR4
8	65616001	65617000	ARMC1
8	67111001	67112000	CSPP1
8	69807001	69808000	SLCO5A1;LOC100101127
8	85739001	85740000	LOC101929601;REXO1L11P;LOC101929627;REXO1L10P
8	85742001	85743000	LOC101929601;REXO1L11P;LOC101929627;REXO1L10P
8	112386001	112387000	CSMD3
8	134498001	134499000	ZFAT
8	140126001	140127000	TRAPPC9
8	142564001	142565000	MROH4P
8	143217001	143218000	GPIHBP1
8	143669001	143670000	LOC105375799
8	143793001	143794000	IQANK1;SCRIB
8	144737001	144738000	ZNF251;LOC107986986
8	144876001	144877000	ZNF250
9	14634001	14635000	ZDHHC21
9	16436001	16437000	BNC2
9	34608001	34609000	RPP25L;DCTN3
9	38999001	39000000	LOC101927042
9	40007001	40008000	LOC105376050
9	40322001	40323000	FAM95B1;LOC100419690
9	40576001	40577000	LOC102724580;BMS1P14;LOC107984006
9	41100001	41101000	LOC440896
9	41648001	41649000	LOC101928906;FAM27E2
9	72236001	72237000	GDA
9	77761001	77762000	GNAQ
9	86052001	86053000	GOLM1
9	92133001	92134000	LOC100128076;LINC00475;LOC112268047
9	93110001	93111000	CARD19
9	94346001	94347000	LOC100132077;LOC107987023;PTMAP12
9	95497001	95498000	PTCH1
9	95816001	95817000	LINC00476;LOC107987103
9	112802001	112803000	SNX30
9	120570001	120571000	CDK5RAP2
9	121792001	121793000	DAB2IP
9	121998001	121999000	TTLL11
9	128426001	128427000	CERCAM
9	128912001	128913000	LRRC8A;LOC101929291;PHYHD1
9	129931001	129932000	FNBP1
9	131054001	131055000	LAMC3
9	132948001	132949000	TSC1;GFI1B;MIR548AW
9	134209001	134210000	LOC107987137;LOC101928384
9	134304001	134305000	LINC02247
9	135821001	135822000	CAMSAP1
9	135967001	135968000	UBAC1
9	136521001	136522000	NOTCH1;MIR4673
9	136828001	136829000	RABL6;MIR4292
9	137865001	137866000	LOC100133077
10	1550001	1551000	ADARB2;ADARB2-AS1
10	3434001	3435000	LOC105376360;LINC02669
10	6018001	6019000	IL2RA
10	7798001	7799000	ATP5F1C;LOC105376392
10	7985001	7986000	TAF3
10	10776001	10777000	CELF2;SFTA1P
10	17008001	17009000	CUBN
10	28560001	28561000	WAC
10	28845001	28846000	C10orf126
10	44969001	44970000	TMEM72-AS1;RASSF4;LOC105378281;DEPP1
10	70294001	70295000	NPFFR1;LRRC20
10	70861001	70862000	SGPL1
10	71404001	71405000	CDH23
10	71667001	71668000	CDH23
10	72326001	72327000	DNAJB12
10	78462001	78463000	LOC107984245;LOC105378378;LOC107984246
10	79012001	79013000	ZMIZ1-AS1;LOC112268062
10	80274001	80275000	MAT1A
10	97187001	97188000	SLIT1;ARHGAP19-SLIT1;LOC112268065
10	100758001	100759000	PAX2
10	103639001	103640000	SH3PXD2A
10	103836001	103837000	SH3PXD2A
10	107951001	107952000	LINC01435
10	112325001	112326000	GUCY2GP
10	114935001	114936000	TRUB1
10	119182001	119183000	PRDX3

10	123801001	123802000	CPXM2
10	129474001	129475000	MGMT
10	132134001	132135000	JAKMIP3
10	132437001	132438000	LOC105378568;LINC02870
10	132820001	132821000	CFAP46
11	1939001	1940000	TNNT3;MRPL23;SNORD131
11	1964001	1965000	MRPL23
11	2529001	2530000	KCNQ1
11	9263001	9264000	DENND5A
11	10508001	10510000	AMPD3;MTRNR2L8;MIR4485;RNF141;AMPD3;MTRNR2L8;MIR4485;RNF141
11	14432001	14433000	LOC105376670
11	14536001	14537000	PSMA1
11	16499001	16500000	SOX6
11	19136001	19137000	ZDHHC13
11	19260001	19261000	CSRP3-AS1
11	20972001	20973000	NELL1
11	24328001	24329000	LOC105376595
11	61456001	61457000	SDHAF2
11	62059001	62060000	LINC02733
11	62658001	62659000	INTS5;LBHD1;C11orf98;CSKMT;SNORA57
11	64274001	64275000	PLCB3;BAD;GPR137
11	64832001	64833000	CDC42BPG
11	67049001	67050000	SYT12;MIR6860;RHOD
11	69492001	69493000	LINC01488
11	70117001	70118000	ANO1
11	70530001	70531000	SHANK2
11	71119001	71120000	SHANK2
11	71751001	71752000	ENPP7P8
11	89984001	89985000	TRIM64;LOC100420025;TRIM51EP;TRIM53AP
11	94038001	94039000	HEPHL1
11	94237001	94238000	LOC105369435
11	94522001	94523000	MRE11;C11orf97
11	107363001	107364000	CWF19L2
11	114252001	114253000	ZBTB16;NNMT
11	117427001	117428000	DSCAML1
11	119432001	119433000	USP2-AS1;THY1
11	119597001	119598000	KRT8P7
11	121321001	121322000	SC5D
11	123092001	123093000	CLMP
11	123510001	123511000	GRAMD1B
11	125206001	125207000	PKNOX2
11	125295001	125296000	PKNOX2
11	128789001	128790000	FLI1
11	133964001	133965000	IGSF9B
12	540001	541000	B4GALNT3
12	3553001	3554000	PRMT8
12	6282001	6283000	LOC105369625
12	6462001	6463000	TAPBPL;VAMP1
12	7694001	7695000	GDF3
12	12073001	12074000	BCL2L14
12	28247001	28248000	CCDC91
12	51260001	51261000	SMAGP;LOC102723983
12	55749001	55750000	GDF11;SARNP
12	57507001	57508000	MARS1;MIR6758;DDIT3
12	66057001	66058000	RNA5SP362
12	71731001	71732000	LOC107984530
12	72639001	72640000	TRHDE
12	108524001	108525000	FICD;SART3
12	108827001	108828000	SSH1;MIR619
12	109798001	109799000	TRPV4
12	109986001	109987000	GIT2
12	112164001	112165000	HECTD4;MIR6861
12	112580001	112581000	RPH3A
12	118066001	118067000	WSB2;VSIG10
12	123636001	123637000	EIF2B1;GTF2H3
12	123687001	123688000	TCTN2
12	124445001	124446000	NCOR2
12	124835001	124836000	SCARB1
12	129500001	129501000	TMEM132D
12	130424001	130425000	RIMBP2
12	131054001	131055000	ADGRD1
12	132067001	132068000	EP400
12	132533001	132534000	FBRSL1
12	132855001	132856000	CHFR
13	18883001	18884000	RNU6-76P;SNX19P2
13	20396001	20397000	CRYL1
13	29158001	29159000	MTUS2
13	30582001	30583000	HMGB1
13	31110001	31111000	WDR95P
13	32122001	32123000	FRY
13	32635001	32636000	PDS5B
13	34540001	34541000	LINC02343;LINC00457
13	73641001	73642000	LINC00393;LOC105370256
13	73777001	73778000	KLF12
13	73915001	73916000	KLF12
13	77961001	77962000	EDNRB
13	89279001	89280000	LINC00440

13	98113001	98114000	LOC105370328
13	98161001	98162000	FARP1
13	111896001	111897000	LINC00354
13	112679001	112680000	ATP11AUN;LOC102725228
13	112921001	112922000	MCF2L
13	113775001	113776000	TMEM255B
14	33929001	33930000	EGLN3
14	56670001	56671000	TMEM260
14	68855001	68856000	MAGOH3P
14	70478001	70479000	ADAM20P1
14	73501001	73502000	HEATR4;RIOX1;ACOT1
14	76850001	76851000	LRRC74A
14	79313001	79314000	NRXN3
14	89578001	89579000	FOXN3;FOXN3-AS2
14	92055001	92056000	ATXN3
14	92653001	92654000	RIN3
14	99699001	99700000	CYP46A1
14	99932001	99933000	EML1;LOC102724682
14	100558001	100559000	BEGAIN
14	105355001	105356000	PACS2
14	105461001	105462000	MTA1
14	105625001	105626000	IGH;MIR8071-1;IGHG4
15	20113001	20114000	LOC105379203
15	20490001	20491000	HERC2P3
15	20550001	20551000	GOLGA6L6;LOC100631265
15	20866001	20867000	POTEB2
15	21109001	21110000	RN7SL400P
15	22267001	22268000	REREP3
15	23340001	23341000	LOC102723623;LOC105376698
15	28417001	28418000	MIR4509-2
15	31484001	31485000	OTUD7A
15	36613001	36614000	CDIN1;LOC751603;LARP4P
15	36878001	36879000	LOC145845
15	40060001	40061000	SRP14-DT
15	43852001	43853000	WDR76
15	49354001	49355000	GALK2;FAM227B;LOC100420615
15	55326001	55327000	PIGBOS1;PIGB
15	55938001	55939000	NEDD4
15	57946001	57947000	ALDH1A2
15	64742001	64743000	LOC107984737;RBPMS2
15	65056001	65057000	SLC51B;RASL12
15	67478001	67479000	IQCH;IQCH-AS1
15	68237001	68238000	CLN6
15	70193001	70194000	RNU6-745P
15	74916001	74917000	FAM219B;COX5A
15	75233001	75234000	LOC105376731
15	78787001	78788000	ADAMTS7
15	84672001	84673000	SEC11A
15	89827001	89828000	LOC105370965;ARPIN-AP3S2;AP3S2
15	90612001	90613000	CRTC3;CRTC3-AS1
15	90865001	90866000	RN7SL363P;FURIN
15	92034001	92035000	SLCO3A1
15	92078001	92079000	SLCO3A1
15	97575001	97576000	LOC105371007
15	99001001	99002000	PGPEP1L;LOC105371013
15	101153001	101154000	LOC105371026
16	32001	33000	WASIR2;IL9RP3
16	463001	464000	RAB11FIP3
16	691001	692000	STUB1;JMJD8;WDR24;FBXL16
16	2058001	2059000	TSC2
16	2623001	2624000	LOC652276;PDPK2P
16	4057001	4058000	ADCY9
16	12180001	12181000	SNX29
16	12474001	12475000	SNX29
16	12988001	12989000	SHISA9
16	15510001	15511000	BMERB1;LOC105371102
16	15617001	15618000	MARF1;MIR6506
16	16011001	16012000	ABCC1
16	16315001	16316000	LOC105371099;MIR3670-2;MIR3180-2;PKD1P1;MIR6511A2
16	21872001	21873000	SMG1P4
16	22527001	22528000	NPIPB5;LOC105371131
16	22538001	22539000	NPIPB5;LOC105371131;OTOAP1
16	27244001	27245000	NSMCE1
16	29499001	29500000	LOC101928556;NPIPB12
16	29628001	29629000	CA5AP1
16	30026001	30027000	DOC2A;C16orf92;TLCD3B
16	30195001	30196000	CORO1A-AS1;CORO1A;BOLA2B;SLX1A;SLX1A-SULT1A3;SULT1A3;LOC613038
16	30336001	30337000	SMG1P5;LOC100421031
16	30881001	30882000	BCL7C;MIR4519;MIR762HG
16	33698001	33699000	BMS1P8
16	33990001	33991000	LOC102724223;BCAP31P1
16	68039001	68040000	DUS2
16	68335001	68336000	PRMT7;RNU4-30P
16	69325001	69326000	VPS4A;COG8;PDF
16	70145001	70146000	PDPR
16	75212001	75213000	CTRB2;CTRB1
16	84414001	84415000	ATP2C2

16	85145001	85146000	LINC02139;LOC105371382
16	85575001	85576000	GSE1
16	85936001	85937000	LINC02132;LOC105371388
16	87669001	87670000	JPH3
16	87734001	87735000	KLHDC4
16	88203001	88204000	LINC02182
16	88260001	88261000	LOC107984862
16	88471001	88472000	ZFPM1;MIR5189
16	88498001	88499000	ZFPM1
16	89153001	89154000	ACSF3;LINC00304
16	89161001	89162000	ACSF3;LINC00304;LINC02138;CDH15
16	89577001	89578000	CPNE7
16	89825001	89826000	FANCA;SPIRE2;LOC107984817
16	89840001	89841000	SPIRE2;LOC107984817;LOC105371419
16	90169001	90170000	FAM157C;LOC105376781;LOC101927999;CICP25;SEPTIN14P16
17	561001	562000	VPS53
17	815001	816000	NXN
17	825001	826000	NXN
17	945001	946000	NXN
17	1481001	1482000	MYO1C
17	1695001	1696000	TLCD2
17	4370001	4371000	UBE2G1;RN7SL774P
17	6466001	6467000	PITPNM3
17	8588001	8589000	MYH10
17	9172001	9173000	NTN1;LOC101928266
17	9519001	9520000	STX8
17	9631001	9632000	CFAP52
17	11919001	11920000	DNAH9
17	14020001	14021000	CDRT15P1;COX10-AS1
17	17488001	17489000	MED9;RASD1
17	18423001	18424000	LINC02076;YWHAEP2;KRT17P5;KRT17P2
17	22521001	22522000	MTND6P35;MTCYBP13;MTRNR2L1;MTND1P15;MTND2P13;MTCO1P13;NMTRS-TGA3-1;MTCO2P13;MTATP6P3;MTCO3P13;MTND4LP8
17	28900001	28901000	FLOT2;DHRS13;PHF12
17	29531001	29532000	TAOK1
17	30214001	30215000	SLC6A4;LOC107984991
17	32994001	32995000	SPACA3
17	35857001	35858000	TAF15;HEATR9;LOC107985049
17	36442001	36443000	TBC1D3F;TBC1D3JP
17	38439001	38440000	ARHGAP23
17	41940001	41941000	ACLY;ODAD4
17	42691001	42692000	CCR10;CNTNAP1;EZH1
17	45181001	45182000	LOC105371795;FMNL1-DT
17	49215001	49216000	GNGT2;ABI3;PHOSPHO1;LOC105371816
17	50449001	50450000	ACSF2;MRPS21P9
17	50529001	50530000	MYCBPAP;EPN3;LOC105371824
17	53106001	53107000	MTCO1P40
17	61379001	61380000	BCAS3
17	65174001	65175000	RGS9;ZNF848P
17	66364001	66365000	PRKCA;RN7SL735P
17	69462001	69463000	MAP2K6
17	72897001	72898000	SLC39A11
17	74884001	74885000	FADS6
17	77468001	77469000	SEPTIN9;LOC105371903
17	78909001	78910000	TIMP2;CEP295NL
17	80358001	80359000	RNF213;RNF213-AS1
17	81406001	81407000	BAHCC1;MIR4740
17	82248001	82249000	SLC16A3;CSNK1D
18	2842001	2843000	EMILIN2
18	20939001	20940000	ROCK1
18	24465001	24466000	HRH4
18	24500001	24501000	LOC105372026;LOC646629
18	35963001	35964000	LOC105372066;C18orf21
18	36648001	36649000	FHOD3
18	45697001	45698000	LOC105372093;LOC105372092
18	46461001	46462000	RNF165
18	48582001	48583000	CTIF
18	58903001	58904000	ZNF532
18	78646001	78647000	LOC105372221;LOC112268207
18	78901001	78902000	LOC105372224
18	79312001	79313000	ATP9B
18	79845001	79846000	KCNG2
18	80177001	80178000	PARD6G-AS1;PARD6G
19	423001	424000	SHC2
19	834001	835000	PLPPR3;AZU1;PRTN3
19	887001	888000	MED16;RNU6-9;R3HDM4
19	1528001	1529000	LOC107985337;PLK5
19	1893001	1894000	ABHD17A
19	1969001	1970000	CSNK1G2
19	2135001	2136000	AP3D1
19	2643001	2644000	GNG7
19	3109001	3110000	GNA11
19	3112001	3113000	GNA11
19	3149001	3150000	GNA15;GNA15-DT
19	4316001	4317000	FSD1;STAP2
19	5591001	5592000	SAFB2
19	5825001	5826000	NRTN;FUT6

19	5855001	5856000	FUT3;LOC101928844;FUT5
19	5888001	5889000	NDUFA11
19	6263001	6264000	MLLT1
19	6369001	6370000	ACER1;CLPP;ALKBH7;PSPN;GTF2F1
19	7594001	7595000	CAMSAP3
19	7706001	7707000	FCER2;LOC105372262
19	8568001	8569000	MYO1F
19	9599001	9600000	LOC105376914;ZNF561
19	12210001	12211000	LOC100289333
19	12579001	12580000	ZNF490
19	12951001	12952000	CALR;RAD23A;GADD45GIP1
19	13222001	13223000	CACNA1A
19	13389001	13390000	CACNA1A
19	16089001	16090000	TPM4
19	16350001	16351000	EPS15L1
19	17487001	17488000	SLC27A1;PGLS-DT
19	20356001	20357000	BNIP3P20
19	22636001	22637000	ZNF492
19	33059001	33060000	RHPN2
19	34352001	34353000	GARRE1;GPI
19	38047001	38048000	SIPA1L3
19	39419001	39420000	ZFP36;MIR4530;TRI-TAT1-1;PLEKHG2
19	41359001	41360000	TGFB1;B9D2;TMEM91
19	42294001	42295000	CIC;PAFAH1B3;PRR19
19	42677001	42678000	RPS10P28
19	43703001	43704000	LOC105372412
19	44473001	44474000	ZNF285B;ZNF180
19	44609001	44610000	IGSF23
19	45245001	45246000	EXOC3L2;MARK4
19	47295001	47296000	LOC105372427
19	47462001	47463000	SLC8A2
19	47922001	47923000	SNAR-C5;SNAR-A1;SNAR-A3;SNAR-C2
19	48757001	48758000	FUT1;FGF21
19	48980001	48981000	GYS1
19	49076001	49077000	KCNA7;RN7SL708P;SNRNP70
19	49123001	49124000	LIN7B;C19orf73;PPFIA3
19	49711001	49712000	CPT1C;RPS9P4
19	50096001	50097000	SNAR-A4;SNAR-A14;SNAR-A5;SNAR-A6
19	50100001	50101000	SNAR-A4;SNAR-A14;SNAR-A5;SNAR-A6;SNAR-A7
19	50485001	50486000	FAM71E1;EMC10
19	51190001	51191000	LOC101928517;SIGLEC20P;LOC100133225;SIGLEC21P
19	53496001	53497000	ZNF813;RPL39P37;VN1R103P;ZNF331
19	53987001	53988000	CACNG8;MIR935;CACNG6
19	54094001	54095000	OSCAR;NDUFA3
19	54425001	54426000	TTYH1
19	55326001	55327000	TMEM150B
19	55335001	55336000	TMEM150B;KMT5C
19	55465001	55466000	ISOC2;C19orf85
19	56046001	56047000	NLRP5
19	57162001	57163000	DUXA
19	58229001	58230000	LOC100419847;ZNF544
20	951001	952000	RSPO4
20	4871001	4872000	SLC23A2
20	4899001	4900000	SLC23A2
20	30824001	30825000	RNA5SP528
20	31646001	31647000	COX4I2
20	32099001	32100000	HCK;TM9SF4
20	33114001	33115000	BPIFB4
20	35814001	35815000	PHF20
20	36743001	36744000	NDRG3;DSN1
20	49283001	49284000	ZNFX1;ZFAS1;SNORD12C;SNORD12B;SNORD12
20	50310001	50311000	LINC01270;LINC01271
20	52291001	52292000	LOC105372666
20	56551001	56552000	FAM209B
20	57267001	57268000	BMP7;LOC112268270;LOC105372687
20	57681001	57682000	PMEPA1
20	57689001	57690000	PMEPA1
20	58466001	58467000	APCDD1L
20	59125001	59126000	ZNF831
20	61312001	61313000	CDH4
20	61773001	61774000	CDH4
20	61865001	61866000	CDH4
20	64105001	64106000	OPRL1;NPBWR2
20	64184001	64185000	MYT1
21	8850001	8851000	MTCO3P26;MTCO1P1;ASNSP2
21	9550001	9551000	NCOR1P4
21	31453001	31454000	TIAM1;LOC100129168
21	36540001	36541000	CLDN14
21	38623001	38624000	ERG
21	43957001	43958000	AGPAT3
21	45176001	45177000	ADARB1
21	45269001	45270000	POFUT2
21	45306001	45307000	LINC00205
21	45506001	45507000	COL18A1;SLC19A1
22	11277001	11278000	DUX4L40;PCMTD1P4;DUX4L41
22	11928001	11929000	LOC102723769
22	12101001	12102000	LOC107987323

22	12812001	12813000	LOC105379428
22	15713001	15714000	POTEH;POTEH-AS1
22	17745001	17746000	BID
22	17799001	17800000	MICAL3
22	19135001	19136000	TSSK1A;ESS2;TSSK2;LOC112268297
22	19506001	19507000	CDC45
22	21137001	21138000	POM121L7P;LOC112268299;E2F6P2
22	21219001	21220000	GGT2
22	25075001	25076000	KIAA1671
22	28645001	28646000	TTC28;RN7SL162P
22	30270001	30271000	OSM
22	30707001	30708000	OSBP2
22	31099001	31100000	SMTN;SELENOM;INPP5J;LOC105372995
22	31975001	31976000	LINC02558;RN7SL305P
22	32340001	32341000	SLC5A4;LOC107985561
22	32871001	32872000	SYN3;TIMP3
22	33045001	33046000	SYN3
22	33902001	33903000	LARGE1
22	37795001	37796000	H1-0
22	38076001	38077000	PICK1;SLC16A8;LOC105373027;BAIAP2L2
22	38551001	38552000	DMC1
22	40290001	40291000	TNRC6B
22	41973001	41974000	SEPTIN3
22	42904001	42905000	PACSIN2;LOC107985550
22	43498001	43499000	MPPED1
22	46393001	46394000	CELSR1
22	48562001	48563000	TAFA5;LOC105373083
22	50478001	50479000	SBF1;ADM2;MIOX
22	50495001	50496000	ADM2;MIOX;LMF2
22	50628001	50630000	ARSA;ARSA
X	464001	465000	LOC102724521
X	1268001	1269000	LOC107985697;CSF2RA
X	1317001	1318000	CSF2RA
X	10066001	10067000	WWC3
X	45041001	45042000	KDM6A
X	49552001	49553000	GAGE12D;GAGE12E;GAGE12F
X	114602001	114603000	HTR2C
X	116814001	116815000	LOC100126447
X	119321001	119322000	LOC101928336
X	121535001	121536000	LOC112268302
X	126472001	126473000	MTCO1P53;MTND4LP1;MTND4P24
X	141010001	141011000	SPANXB1
X	153178001	153179000	MAGEA1
X	155951001	155952000	VAMP7
Y	11311001	11312000	DUX4L16;DUX4L17;DUX4L18
Y	11327001	11328000	DUX4L18;DUX4L19;PABPC1P5

(D) Orange

Chr	Start	Stop	Annotation
1	6433001	6434000	ESPN;MIR4252
1	23689001	23690000	RPL11
1	169620001	169621000	SELP
2	85303001	85304000	TCF7L1
3	173010001	173011000	SPATA16
4	3433001	3434000	RGS12;HGFAC
4	49524001	49525000	LOC107986214
5	486001	488000	SLC9A3;SLC9A3-AS1;SLC9A3;SLC9A3-AS1
6	160628001	160629000	LPA
7	6889001	6890000	OR7E59P;LOC100420863
8	17543001	17544000	SLC7A2
9	84526001	84527000	LOC102724036
10	43369001	43370000	FXYD4
10	133216001	133217000	KNDC1;LOC105378573
13	51472001	51473000	RN7SL320P
14	34557001	34558000	LOC105370449;RNU1-28P;SNX6
14	101049001	101050000	MIR376C;MIR376A2;MIR654;MIR376B;MIR376A1;MIR300;MIR1185-1;MIR1185-2;MIR381HG;MIR381;MIR487B;MIR539;MIR889;MIR544A;MIR655;MIR487A;MIR382;MIR134;MIR668;MIR485;MIR323B;MIR154
15	97224001	97225000	LINC02253
16	69063001	69064000	TANGO6
16	89963001	89964000	DEF8;SNORA119;CENPBD1;AFG3L1P
17	59394001	59395000	YPEL2
17	76119001	76120000	LOC101928447
19	36301001	36310000	LOC100134317;LOC100134317;LOC100134317;LINC00665;LOC100134317;LINC00665;LOC100134317;LINC00665;LOC100134317;LINC00665;LOC100134317;LINC00665;LOC100134317;LINC00665;LOC100134317;LINC00665
19	37269001	37273000	LINC01535;LOC284412;LINC01535;LOC284412;LINC01535;LOC284412;LINC01535;LOC284412
19	37274001	37280000	LINC01535;LOC284412;LINC01535;LOC284412;LOC284412;LOC284412;LOC284412;LOC284412
19	37302001	37304000	ZNF875;ZNF875
19	45472001	45473000	FOSB
19	47950001	47951000	SNAR-A13;SNAR-C3;SNAR-C1
21	9826001	9832000	LINC01667;LINC01667;LINC01667;LINC01667;LINC01667;LINC01667
21	10123001	10124000	LOC105372733
21	17425001	17426000	RNU6-113P
22	12692001	12693000	DUX4L44
22	23777001	23778000	CHCHD10;LOC107985577;MMP11;SMARCB1
22	42129001	42130000	NDUFA6-DT;LOC105373047;CYP2D6;LOC112268294;LOC102723722;CYP2D7
X	1209001	1210000	CRLF2
Y	11323001	11324000	DUX4L17;DUX4L18;DUX4L19

(E) Red

Chr	Start	Stop	Annotation
1	10001	11000	DDX11L1;WASH7P;MIR6859-1
1	16001	17000	DDX11L1;WASH7P;MIR6859-1
1	268001	269000	LOC100996442;RPL23AP21
1	788001	789000	LOC100288069;LINC01409
1	1129001	1130000	LINC01342
1	1202001	1203000	TTLL10;TNFRSF18;TNFRSF4
1	1288001	1291000	LINC01786;SCNN1D;ACAP3;MIR6726;LINC01786;SCNN1D;ACAP3;MIR6726;SCNN1D;ACAP3;MIR6726
1	1386001	1387000	NDUFB4P8;CCNL2
1	1545001	1546000	TMEM240;SSU72
1	1547001	1548000	TMEM240;SSU72
1	1761001	1762000	LOC112268218;NADK
1	1933001	1934000	CFAP74
1	1945001	1946000	CFAP74;LOC107984872
1	1958001	1959000	CFAP74;LOC107984872
1	2062001	2063000	PRKCZ
1	2078001	2079000	PRKCZ
1	2080001	2081000	PRKCZ
1	2123001	2124000	PRKCZ
1	2262001	2263000	SKI
1	2472001	2473000	PLCH2
1	2634001	2635000	MMEL1;MMEL1-AS1;TTC34
1	2663001	2664000	TTC34
1	2671001	2672000	TTC34
1	2695001	2696000	TTC34;LOC105378603;LOC105378602
1	2747001	2748000	TTC34
1	3111001	3112000	PRDM16
1	3299001	3301000	PRDM16;LOC105378604;PRDM16;LOC105378604
1	3450001	3451000	ARHGEF16
1	3509001	3510000	MEGF6
1	3914001	3915000	LINC01134
1	4689001	4690000	AJAP1
1	4976001	4977000	LINC02781
1	5945001	5946000	NPHP4
1	6046001	6047000	KCNAB2
1	6442001	6443000	ESPN
1	6577001	6578000	TAS1R1;LOC107984912;ZBTB48
1	6694001	6695000	DNAJC11
1	7137001	7138000	CAMTA1
1	7255001	7256000	CAMTA1
1	7481001	7482000	CAMTA1
1	7513001	7514000	CAMTA1;LOC105376691
1	7972001	7973000	PARK7
1	8010001	8011000	ERRFI1
1	8145001	8146000	LOC107984915
1	8337001	8339000	SLC45A1;SLC45A1
1	8720001	8721000	RERE
1	9897001	9898000	CTNNBIP1
1	10038001	10039000	UBE4B
1	10303001	10304000	KIF1B;RNU6-37P;RN7SL731P
1	11421001	11422000	MTCYBP45
1	11433001	11434000	MTCYBP45
1	13555001	13556000	BRWD1P1
1	15127001	15128000	KAZN;TMEM51-AS1
1	16285001	16286000	FBXO42
1	16576001	16577000	NBPF1
1	16596001	16597000	NBPF1
1	16673001	16674000	LOC107985736;RNU1-3;TRG-CCC4-1;EIF1AXP1
1	16680001	16681000	TRG-CCC4-1;EIF1AXP1
1	16710001	16711000	ESPNP
1	16717001	16718000	ESPNP;TRV-CAC13-1;TRG-CCC5-1
1	16738001	16739000	RNU1-4
1	16741001	16742000	RNU1-4;CROCCP4
1	16751001	16752000	CROCCP4;MST1L
1	16762001	16763000	MST1L
1	16769001	16770000	MST1L
1	17216001	17217000	PADI1
1	17504001	17505000	ARHGEF10L
1	17590001	17591000	ARHGEF10L
1	18209001	18210000	IGSF21
1	19061001	19062000	LOC105376815
1	19111001	19112000	UBR4
1	19328001	19329000	SLC66A1;CAPZB
1	19703001	19704000	TMCO4
1	21052001	21053000	EIF4G3
1	21588001	21589000	ALPL;LINC02596;RAP1GAP
1	21593001	21594000	LINC02596;RAP1GAP
1	21884001	21885000	HSPG2
1	22153001	22154000	WNT4;LOC105376845;LOC105376850
1	22177001	22178000	LOC105376850
1	22252001	22253000	LOC107985377
1	22429001	22430000	ZBTB40
1	22541001	22542000	ZBTB40
1	22730001	22731000	EPHB2
1	23098001	23099000	LUZP1

1	23595001	23596000	LOC107984928
1	23857001	23858000	FUCA1
1	24557001	24558000	NCMAP-DT;NCMAP;RPL26P8
1	25391001	25392000	RHCE;SDHDP7
1	26010001	26011000	LOC101928216
1	26485001	26486000	HMGN2
1	26833001	26834000	ZDHHC18
1	27746001	27747000	FAM76A;RPEP3
1	28681001	28682000	GMEB1
1	29267001	29268000	PTPRU
1	29309001	29310000	PTPRU
1	29487001	29488000	LOC107984933
1	29642001	29643000	LOC107984934
1	30245001	30246000	LOC105378618
1	32821001	32822000	YARS1;S100PBP
1	32906001	32907000	TMEM54;LOC105378632
1	33160001	33161000	AZIN2;TRIM62
1	33644001	33645000	CSMD2
1	34794001	34795000	LOC105378642;GJB3;GJA4
1	35924001	35925000	AGO1;AGO3
1	36112001	36113000	COL8A2
1	36159001	36160000	TRAPPC3;MAP7D1
1	37740001	37741000	EPHA10
1	37910001	37911000	INPP5B
1	37971001	37972000	SF3A3
1	38036001	38037000	POU3F1
1	39524001	39525000	BMP8A;OXCT2P1;PPIEL
1	39683001	39684000	HPCAL4
1	39802001	39803000	LOC105378951;LINC02811;LOC105378665
1	40325001	40326000	COL9A2;RPL21P20
1	42231001	42232000	FOXJ3
1	42757001	42758000	P3H1;C1orf50
1	43560001	43561000	PTPRF
1	44086001	44087000	KLF17
1	46036001	46037000	MAST2;P3R3URF-PIK3R3;PIK3R3
1	47286001	47287000	STIL
1	47760001	47761000	TRABD2B
1	48547001	48548000	AGBL4
1	52724001	52725000	RN7SL62P;ZYG11B
1	52912001	52913000	ECHDC2;LOC112268226
1	53733001	53734000	GLIS1
1	54543001	54544000	HNRNPA1P63;ACOT11
1	55025001	55026000	BSND
1	59142001	59143000	HSD52
1	59588001	59589000	FGGY
1	60929001	60930000	LOC105378764
1	61563001	61564000	LOC105378766
1	63207001	63208000	LINC00466;LOC105378770
1	63310001	63311000	LINC00466;FOXD3-AS1
1	64759001	64760000	RAVER2
1	71684001	71685000	NEGR1
1	74247001	74248000	FPGT-TNNI3K;TNNI3K
1	77088001	77089000	PIGK
1	81303001	81304000	LOC107985018;ADGRL2
1	92724001	92725000	EVI5;LOC100289500
1	93588001	93589000	BCAR3;BCAR3-AS1
1	93832001	93833000	BCAR3
1	95183001	95184000	TLCD4;TLCD4-RWDD3;LOC101928118
1	96761001	96762000	PTBP2
1	99262001	99263000	PLPPR4
1	107056001	107057000	PRMT6
1	107695001	107696000	VAV3
1	108294001	108295000	SLC25A24P1
1	108475001	108476000	NBPF6
1	108570001	108571000	FAM102B
1	110170001	110171000	SLC6A17;SLC6A17-AS1
1	110226001	110227000	KCNC4
1	111618001	111619000	RAP1A;LINC01160;LOC107985184
1	111717001	111719000	RAP1A;INKA2;RAP1A;INKA2
1	112224001	112225000	LINC02884
1	112886001	112887000	LINC01357;MIR11399;RPL39P8
1	113636001	113637000	MAGI3
1	115753001	115754000	CASQ2
1	116515001	116516000	CD58
1	116930001	116931000	PTGFRN
1	116961001	116962000	PTGFRN;RNA5SP55
1	116964001	116965000	PTGFRN;RNA5SP55
1	118853001	118854000	LOC107985447
1	144816001	144817000	LOC105371216;LOC105371217
1	145577001	145578000	PDE4DIPP5;NBPF25P
1	145733001	145734000	CD160;RNF115
1	145793001	145794000	RNF115
1	146316001	146317000	SEC22B4P
1	148061001	148062000	PDE4DIPP1
1	150430001	150431000	RPRD2
1	151507001	151508000	CGN
1	151528001	151529000	CGN

1	151608001	151609000	SNX27
1	151696001	151697000	SNX27;CELF3
1	154066001	154067000	NUP210L
1	154428001	154429000	IL6R
1	154927001	154928000	LOC105371449;PMVK
1	155121001	155122000	EFNA1
1	156266001	156267000	SMG5
1	156398001	156399000	MIR9-1HG
1	156562001	156563000	IQGAP3
1	156666001	156667000	BCAN-AS1;BCAN;NES
1	156774001	156775000	PRCC
1	156983001	156984000	ARHGEF11;RN7SL612P
1	160389001	160390000	LOC105371466;RNU4-42P
1	160703001	160704000	CD48
1	161179001	161180000	PPOX;B4GALT3;ADAMTS4
1	161323001	161324000	SDHC
1	161818001	161819000	ATF6
1	163180001	163181000	RGS5;RGS5-AS1
1	167030001	167031000	MAEL
1	167282001	167283000	POU2F1
1	167779001	167780000	MPZL1
1	168240001	168241000	SFT2D2;ANKRD36BP1
1	168790001	168791000	LINC00626
1	171425001	171426000	RNU6-290P
1	171829001	171830000	RPLP1P3
1	172315001	172316000	DNM3
1	174236001	174237000	RABGAP1L
1	174437001	174438000	RABGAP1L;GPR52
1	175435001	175436000	TNR
1	175493001	175494000	TNR
1	175711001	175712000	TNR
1	175993001	175994000	COP1
1	181325001	181326000	CACNA1E
1	183617001	183618000	LOC107985235;ARPC5
1	183776001	183777000	RGL1
1	184917001	184919000	NIBAN1;NIBAN1
1	185049001	185050000	RNF2
1	197626001	197627000	DENND1B
1	200890001	200891000	LOC107985245;INAVA
1	200959001	200960000	MROH3P;KIF21B
1	201566001	201567000	NAV1
1	203381001	203382000	LOC102723529
1	203508001	203509000	OPTC;LOC105371688
1	204158001	204159000	ETNK2;REN
1	204182001	204183000	KISS1
1	204412001	204413000	PPP1R15B;PIK3C2B
1	204437001	204438000	PIK3C2B
1	205064001	205065000	CNTN2
1	206373001	206374000	SRGAP2
1	206454001	206455000	SRGAP2
1	206461001	206462000	SRGAP2;IKBKE
1	206467001	206468000	SRGAP2;IKBKE;MIR6769B
1	209245001	209246000	LOC105372896
1	210602001	210603000	HHAT
1	210653001	210654000	HHAT
1	211398001	211399000	LINC00467
1	212215001	212216000	LINC02608
1	212778001	212779000	NSL1
1	219868001	219869000	LOC105372926
1	222874001	222875000	DISP1
1	223657001	223658000	CAPN8
1	224585001	224586000	CNIH3;AKR1B1P1
1	225819001	225820000	EPHX1
1	226070001	226071000	H3-3A
1	226455001	226456000	RN7SKP165
1	227640001	227641000	ZNF678
1	228238001	228239000	OBSCN
1	228288001	228289000	OBSCN
1	228722001	228723000	RHOU
1	229217001	229218000	LINC02815
1	229412001	229413000	RN7SKP276
1	230817001	230818000	RNA5SP79
1	230914001	230915000	TTC13
1	234423001	234424000	TARBP1
1	234778001	234779000	LOC107985364
1	235909001	235910000	LOC105373215;LINC02768;RNU6-968P
1	235963001	235964000	LOC105373215;LINC02768
1	235993001	235994000	NID1
1	237566001	237567000	RYR2
1	237803001	237804000	RYR2
1	237941001	237942000	MTCO1P38;MTND2P27;NMTRP-TGG1-1;MTND1P25;NMTRL-TAA3-1;MTRNR2L11;MTCYBP15;MTND6P15;MTND5P18
1	239435001	239436000	CHRM3
1	240701001	240702000	LOC105373228
1	241108001	241109000	RGS7
1	242291001	242292000	PLD5
1	243239001	243240000	CEP170
1	244367001	244368000	C1orf100

1	244522001	244523000	CATSPERE
1	244606001	244607000	CATSPERE;CYCSP5
1	244618001	244619000	CATSPERE
1	245033001	245034000	EFCAB2;RNU6-1089P;LOC107985725;RNU6-999P
1	245173001	245174000	KIF26B
1	246292001	246293000	SMYD3;LOC107985599
1	246312001	246313000	SMYD3
1	246637001	246638000	CNST;LOC100887078;LOC107985100
1	246639001	246640000	CNST;LOC100887078;LOC107985100
1	246670001	246671000	CNST
1	246747001	246748000	SCCPDH;RPL35AP6
1	247445001	247446000	NLRP3;OR2B11
1	247466001	247467000	OR2B11
1	247845001	247846000	OR11L1
1	248945001	248947000	RPL23AP25;RPL23AP25
2	725001	726000	LOC105373358
2	1158001	1159000	SNTG2
2	1266001	1267000	SNTG2
2	1295001	1296000	SNTG2
2	1404001	1405000	TPO
2	1489001	1490000	TPO
2	1521001	1522000	TPO
2	2005001	2006000	MYT1L
2	3181001	3184000	LOC107985840;EIPR1;LOC107985840;EIPR1;LOC107985840;EIPR1
2	3239001	3240000	EIPR1
2	3314001	3315000	EIPR1
2	3667001	3668000	ALLC
2	3732001	3733000	DCDC2C
2	3750001	3751000	DCDC2C
2	3972001	3973000	LINC01304;LOC105373394
2	6760001	6761000	LINC00487
2	6942001	6943000	RNF144A
2	7919001	7920000	LINC00298
2	8203001	8204000	LINC00299
2	8638001	8639000	LOC105373411
2	8912001	8913000	MBOAT2
2	10024001	10025000	LOC107985852;LOC112268412
2	10276001	10277000	RN7SL66P
2	10278001	10279000	RN7SL66P;LOC101929691
2	10551001	10552000	LOC105373426
2	11666001	11667000	NTSR2;CDK8P1
2	11863001	11864000	LOC105373430
2	17649001	17650000	VSNL1
2	20105001	20106000	LAPTM4A-DT
2	23687001	23688000	KLHL29;ATAD2B
2	24803001	24804000	PTRHD1;LOC105369164;CENPO
2	25206001	25207000	LINC01381
2	25462001	25463000	DTNB
2	25976001	25977000	KIF3C
2	27078001	27079000	AGBL5;OST4;EMILIN1;KHK
2	28115001	28116000	BABAM2
2	31072001	31073000	GALNT14
2	31374001	31375000	XDH
2	32916001	32917000	LINC00486;LTBP1
2	33251001	33252000	LTBP1
2	37063001	37064000	HEATR5B
2	39596001	39597000	MAP4K3-DT
2	39733001	39734000	TMEM178A;THUMPD2
2	42158001	42159000	EML4-AS1
2	42560001	42561000	MTA3
2	43294001	43295000	THADA
2	43296001	43297000	THADA
2	46393001	46394000	EPAS1;LINC01820
2	46453001	46454000	RN7SL817P
2	46694001	46695000	SOCS5
2	47642001	47643000	MSH2
2	51805001	51806000	LOC730100
2	52023001	52024000	LOC730100
2	54041001	54042000	ACYP2;HMGB1P31
2	55597001	55598000	PPP4R3B
2	57546001	57547000	LOC105377632
2	60892001	60893000	REL
2	63707001	63708000	CSP1
2	64709001	64710000	SERTAD2
2	66392001	66393000	LINC01873
2	66911001	66912000	LINC01799;LINC01628
2	69497001	69498000	AAK1
2	69867001	69868000	GMCL1;RPL23AP92
2	70012001	70013000	ASPRV1;PCBP1-AS1
2	70194001	70195000	C2orf42
2	72674001	72675000	EXOC6B
2	72753001	72754000	EXOC6B
2	72890001	72891000	SPR
2	73391001	73392000	LOC105374804;ALMS1
2	73746001	73747000	TPRKB
2	74100001	74101000	TET3
2	74420001	74421000	C2orf81;HMGA1P8;WDR54;RTKN

2	74446001	74447000	RTKN;INO80B-WBP1;INO80B
2	75214001	75215000	LOC105374811
2	76575001	76576000	LOC105374814
2	82817001	82818000	MTND4P25;MTND5P27;MTND6P7;MTCYBP7;LOC105374831
2	83295001	83296000	LOC105374833
2	84282001	84283000	LOC107985905;FUNDC2P2
2	84287001	84288000	LOC107985905;FUNDC2P2
2	84910001	84911000	LOC105374837;LOC105374836;TMSB10;RPS2P17
2	86236001	86237000	REEP1
2	87797001	87798000	RGPD2
2	88690001	88691000	LOC102724805;RPIA
2	88919001	88920000	IGK;IGKV7-3;PGBD4P5
2	89583001	89585000	IGK;LOC107985911;IGK;LOC107985911
2	89811001	89812000	IGK
2	89814001	89815000	IGK
2	89816001	89817000	IGK
2	89822001	89823000	IGK
2	89826001	89842000	IGK;IGK;IGK;IGK;IGK;IGK;IGK;IGK;IGK;IGK;LOC105374862;IGK;LOC105374862;IGK;LOC105374862;IGK;LOC105374862;IGK;LOC105374862;IGK;LOC105374862;IGK;LOC105374862;IGKV2D-40
2	90300001	90301000	LOC101926946
2	91597001	91598000	LOC388996
2	91839001	91840000	ABCD1P5
2	94753001	94754000	LOC100419684;FAM95A;CYP4F32P;ANKRD20A8P
2	94809001	94810000	ANKRD20A8P
2	96033001	96034000	FAHD2CP;GPAT2;LOC105373493
2	96828001	96829000	CNNM3;ANKRD23
2	97895001	97896000	TMEM131
2	99567001	99568000	AFF3
2	100042001	100043000	AFF3
2	102327001	102328000	IL1RL1
2	107298001	107299000	LINC01789
2	108585001	108586000	LIMS1
2	109199001	109200000	SH3RF3
2	109498001	109500000	SH3RF3;SH3RF3
2	109604001	109605000	SEPTIN10;SOWAHC
2	110286001	110287000	MIR4436B2
2	110540001	110541000	RGPD6
2	111266001	111267000	MIR4435-2HG;LOC101927283
2	112495001	112496000	TTL
2	113594001	113595000	WASH2P;DDX11L2
2	114714001	114715000	DPP10
2	117026001	117027000	NMTRL-TAA2-1;MTND1P28;MTND2P21;MTCO1P43
2	118010001	118011000	CCDC93;RN7SL111P
2	119248001	119249000	STEAP3;STEAP3-AS1
2	119998001	119999000	RPL27P7;LOC107985817
2	120024001	120025000	EPB41L5
2	120258001	120259000	RALB
2	120840001	120841000	GLI2
2	121752001	121753000	TSN
2	121771001	121772000	TSN;LOC105373590
2	127075001	127076000	BIN1
2	127579001	127580000	MYO7B;LOC105373609
2	128451001	128452000	LOC105373611;RNA5SP103
2	128578001	128579000	LOC105373611
2	128610001	128611000	LOC105373611
2	130128001	130129000	POTEF;MED15P9
2	130217001	130218000	RHOQP3
2	130446001	130447000	CYP4F62P
2	130580001	130581000	PRSS40A
2	131281001	131282000	LOC440910;CYP4F31P
2	132272001	132273000	CDC27P1;LOC105373622;RNA28SP
2	132288001	132289000	RNA28SP
2	135736001	135737000	LOC107985946;UBXN4
2	136759001	136760000	THSD7B
2	137361001	137362000	THSD7B
2	137684001	137685000	THSD7B
2	140223001	140224000	MTCO1P44;MTND2P19;MTND1P27;LRP1B
2	144902001	144903000	TEX41
2	148190001	148191000	MBD5
2	149016001	149017000	KIF5C
2	151595001	151596000	NEB
2	152159001	152160000	STAM2
2	154957001	154958000	LOC105373696
2	155263001	155264000	MTCO1P45;LOC105373698;MTND2P20;ATP5F1AP2
2	155313001	155314000	MTND4P28;MTND5P30;MTND6P9;MTCYBP9
2	157369001	157370000	FAM133DP
2	158822001	158823000	DAPL1
2	158853001	158854000	OR7E89P;OR7E28P
2	159183001	159184000	TANC1;MIR6888
2	160209001	160210000	ITGB6
2	167668001	167669000	B3GALT1
2	169826001	169827000	METTL5;SNORD3K;UBR3
2	174407001	174408000	SCRN3
2	176797001	176798000	FUCA1P1;RPL29P8
2	177786001	177787000	PDE11A
2	177845001	177846000	PDE11A
2	178050001	178051000	PDE11A

2	178882001	178883000	CCDC141;LOC105373766
2	192134001	192135000	TMEFF2
2	197533001	197534000	HSPE1-MOB4;MOB4;RNY5P2
2	197709001	197710000	MARS2
2	199840001	199841000	FTCDNL1
2	202371001	202372000	BMPR2
2	202523001	202524000	BMPR2;RPL13AP12
2	203008001	203009000	NBEAL1
2	203243001	203244000	CYP20A1
2	203865001	203866000	CTLA4
2	203967001	203968000	ICOS
2	208785001	208786000	LOC101927960;LOC105373856
2	214104001	214105000	SPAG16;LOC101928084
2	214508001	214509000	VWC2L;VWC2L-IT1
2	215016001	215017000	ABCA12
2	217750001	217751000	DIRC3;RPL7L1P9
2	221569001	221570000	EPHA4
2	227624001	227625000	C2orf83;LOC729968
2	229623001	229624000	DNER;LOC105373923
2	230881001	230882000	ITM2C;GCSIR
2	230950001	230951000	GPR55;COX20P2
2	231611001	231612000	LOC105373928
2	231715001	231716000	PTMA;MIR1244-1
2	232153001	232154000	DIS3L2
2	232983001	232984000	NGEF
2	233384001	233385000	DGKD
2	233582001	233583000	UGT1A;UGT1A12P
2	233734001	233735000	UGT1A;UGT1A8;UGT1A10;UGT1A9;UGT1A7;UGT1A6;UGT1A5;UGT1A4;UGT1A3;DNAJB3
2	234847001	234848000	LOC101927896
2	234889001	234890000	LOC101927896
2	234970001	234971000	SH3BP4;LOC105373939
2	234998001	234999000	SH3BP4
2	235384001	235385000	LOC105373941
2	235750001	235751000	AGAP1;LOC105373942
2	235941001	235942000	AGAP1
2	236401001	236402000	IQCA1;RPL3P5
2	236969001	236970000	LOC105373951
2	237361001	237362000	COL6A3
2	237523001	237524000	MLPH;MTND5P46;MTND4P40
2	237559001	237560000	MLPH;RNU6-1140P;PRLH
2	238253001	238254000	PER2
2	238744001	238745000	LINC01937
2	239104001	239105000	HDAC4
2	239372001	239373000	HDAC4
2	239406001	239407000	HDAC4;HDAC4-AS1
2	240020001	240021000	NDUFA10;OR6B2
2	240478001	240479000	GPC1;ANKMY1
2	240728001	240729000	KIF1A
2	240868001	240869000	AGXT
2	241013001	241014000	SNED1;LOC107985787;SNED1-AS1
2	241229001	241230000	ANO7;LOC105376810;HDLBP
2	241231001	241232000	ANO7;LOC105376810;HDLBP
2	241666001	241667000	ATG4B;DTYMK
2	241700001	241701000	ING5
2	241718001	241720000	ING5;ING5
2	241763001	241764000	D2HGDH;LOC105373976
2	241944001	241945000	LINC01237
2	241985001	241986000	LINC01237;LINC01238
2	242019001	242020000	LINC01237
2	242183001	242184000	RPL23AP88
3	730001	731000	LINC01266
3	892001	893000	LOC107986059
3	1606001	1607000	RPL23AP38
3	2906001	2907000	CNTN4
3	4804001	4805000	ITPR1
3	9155001	9156000	SRGAP3
3	9642001	9643000	MTMR14
3	10197001	10198000	IRAK2
3	11028001	11029000	SLC6A1;SLC6A1-AS1
3	12173001	12174000	SYN2;MTCO1P5
3	12902001	12903000	LOC105376956;IQSEC1
3	13016001	13017000	IQSEC1
3	13032001	13033000	IQSEC1
3	13082001	13083000	IQSEC1
3	13307001	13308000	NUP210
3	13344001	13345000	NUP210
3	13553001	13554000	FBLN2
3	13714001	13715000	LINC00620
3	14138001	14139000	TMEM43;XPC
3	15193001	15194000	COL6A4P1
3	15303001	15304000	SH3BP5
3	19033001	19034000	LOC107986066
3	21725001	21726000	ZNF385D
3	24092001	24093000	NPM1P23;LINC00691
3	26165001	26166000	LOC105377002
3	27454001	27455000	SLC4A7;RPS20P15
3	30725001	30726000	GADL1

3	32390001	32391000	CMTM7
3	36904001	36905000	TRANK1
3	37208001	37209000	LOC112268444;LOC105377642
3	38065001	38066000	DLEC1
3	38307001	38308000	SLC22A14;RNU6-235P
3	38650001	38651000	SCN5A
3	41891001	41892000	ULK4
3	45006001	45007000	EXOSC7
3	45142001	45143000	CDCP1
3	46391001	46392000	CCR5AS
3	46662001	46663000	ALS2CL
3	47138001	47139000	SETD2
3	48640001	48641000	SLC26A6;MIR6824;CELSR3;MIR4793
3	48941001	48942000	ARIH2
3	49287001	49288000	C3orf62;USP4
3	49458001	49459000	RNA5SP130;DAG1
3	50231001	50232000	SLC38A3;GNAI2;MIR5787
3	50307001	50308000	HYAL3;NAA80;HYAL1;HYAL2
3	50742001	50743000	DOCK3
3	52377001	52378000	DNAH1;PPP2R5CP
3	54295001	54296000	CACNA2D3
3	57308001	57309000	DNAH12
3	60889001	60890000	FHIT
3	62852001	62853000	CADPS
3	63139001	63140000	LOC107986043
3	63734001	63735000	LOC100130345
3	64780001	64781000	ADAMTS9-AS2
3	70972001	70973000	FOXP1
3	71764001	71765000	EIF4E3;GPR27;PROK2
3	72915001	72916000	GXYLT2
3	75470001	75471000	LINC02018;ENPP7P2
3	77490001	77491000	ROBO2
3	81054001	81055000	LINC02027
3	88386001	88387000	CSNKA2IP
3	93869001	93870000	PROS1
3	97324001	97325000	EPHA6;LOC101929278
3	97642001	97643000	EPHA6
3	98246001	98247000	LOC101929320;OR5H7P
3	98664001	98665000	WWP1P1
3	99907001	99908000	CMSS1;FILIP1L
3	101514001	101515000	SENP7;FAM172BP
3	111549001	111550000	LOC105374039;CD96
3	112163001	112164000	SLC9C1
3	113375001	113376000	CFAP44
3	113459001	113460000	SPICE1
3	113574001	113575000	SIDT1
3	113924001	113925000	GRAMD1C;VPS26AP1
3	114247001	114248000	ZNF80
3	120626001	120627000	HGD
3	123349001	123350000	ADCY5
3	123443001	123444000	ADCY5
3	123984001	123985000	ROPN1
3	125166001	125167000	SLC12A8
3	126804001	126805000	CHCHD6
3	127016001	127018000	PLXNA1;PLXNA1
3	127024001	127025000	PLXNA1
3	128659001	128660000	RPN1
3	128834001	128835000	MARK2P17
3	128857001	128858000	MARK3P3;MARK2P6;LOC653712
3	128963001	128964000	CFAP92
3	129208001	129209000	RPS27P12
3	130017001	130018000	OR7E129P
3	136173001	136174000	MSL2
3	136243001	136244000	PCCB
3	138159001	138160000	DBR1
3	141734001	141735000	RNF7
3	141799001	141800000	GRK7
3	143722001	143723000	SLC9A9
3	143804001	143805000	SLC9A9
3	146354001	146355000	LOC100419967
3	155284001	155285000	STRIT1
3	155509001	155510000	PLCH1
3	156042001	156043000	VN2R1P;ALG1L15P
3	164646001	164647000	LOC105374191;LOC107986151
3	167747001	167748000	SERPINI1
3	170256001	170257000	PRKCI
3	170379001	170380000	SKIL
3	170819001	170820000	LOC105374211
3	170821001	170822000	LOC105374211
3	172285001	172286000	FNDC3B
3	176686001	176687000	LOC105374229
3	177246001	177247000	LOC107986053
3	177662001	177663000	LINC00578
3	182983001	182984000	DCUN1D1;LOC105374246
3	183103001	183104000	MCCC1;LOC105374247
3	184245001	184246000	VWA5B2;MIR1224;ALG3;EEF1AKMT4-ECE2;EEF1AKMT4
3	184379001	184380000	THPO;CHRD

3	184579001	184580000	EPHB3
3	185593001	185594000	SENP2
3	186230001	186231000	DGKG
3	186233001	186234000	DGKG
3	186818001	186819000	LINC02043
3	186894001	186895000	RPS20P14
3	188201001	188202000	LPP
3	188456001	188457000	LPP
3	192796001	192797000	MB21D2
3	192817001	192818000	MB21D2
3	194353001	194354000	CPN2;LRRC15
3	194554001	194555000	RPL23AP93
3	194570001	194571000	RPL23AP93
3	194803001	194804000	LOC105374292
3	194822001	194823000	LOC105374292
3	195931001	195932000	RNU2-11P;LOC727978;LOC100133326
3	196531001	196532000	RPS29P3
3	196729001	196730000	PIGX;PAK2
3	196898001	196899000	SENP5
3	196964001	196965000	PIGZ
3	197459001	197460000	LOC105374308
3	197520001	197521000	BDH1
3	197879001	197880000	LRCH3;IQCG
3	198014001	198015000	LMLN
3	198168001	198170000	FAM157A;FAM157A
4	38001	39000	LOC105374377
4	403001	404000	ZNF519P4
4	564001	566000	TMEM271;TMEM271
4	645001	646000	PDE6B;PDE6B-AS1
4	718001	719000	PCGF3
4	866001	867000	GAK;LOC105374341
4	874001	875000	GAK;LOC105374341
4	902001	903000	GAK
4	909001	910000	GAK
4	1224001	1225000	CTBP1-AS;CTBP1
4	1661001	1662000	FAM53A
4	1665001	1666000	FAM53A
4	2137001	2138000	POLN
4	2407001	2408000	ZFYVE28
4	2424001	2425000	ZFYVE28;CFAP99
4	2552001	2553000	FAM193A
4	2572001	2573000	FAM193A
4	2582001	2583000	FAM193A
4	3290001	3291000	RGS12
4	3637001	3638000	LOC112268461;LOC105374357
4	3669001	3670000	LINC02171
4	4127001	4128000	OR7E103P
4	6354001	6355000	PPP2R2C
4	6455001	6456000	PPP2R2C
4	7015001	7016000	TBC1D14
4	7030001	7031000	TBC1D14;LOC100129931;CCDC96
4	7754001	7755000	AFAP1-AS1;AFAP1
4	7757001	7758000	AFAP1-AS1;AFAP1
4	7806001	7807000	AFAP1
4	8587001	8588000	GPR78;CPZ
4	10507001	10508000	CLNK;LOC105374482
4	17954001	17955000	LCORL
4	23306001	23307000	LOC105374524;LOC105374523
4	23723001	23724000	LOC105374528
4	26426001	26427000	RBPJ
4	35481001	35482000	SEC63P2
4	37604001	37605000	C4orf19;RELL1
4	39247001	39248000	WDR19
4	39396001	39397000	RNU6-887P;KLB
4	41352001	41353000	LIMCH1
4	42370001	42371000	LOC105374428
4	43463001	43464000	LINC02383
4	47618001	47619000	CORIN;LOC105374444
4	47868001	47869000	LOC101927179;NFXL1
4	49151001	49157000	LOC101927209;LOC101927209;LOC101927209;LOC101927209;LOC101927209;LOC101927209
4	49160001	49161000	LOC101927209
4	49513001	49515000	ANKRD20A17P;LOC107986214;ANKRD20A17P;LOC107986214
4	52539001	52540000	LOC112268466
4	54020001	54021000	CHIC2
4	54025001	54026000	CHIC2
4	55919001	55920000	LOC105377663
4	56095001	56096000	CRACD;RNA5SP161
4	64607001	64609000	MTCO3P27;MTCYBP16;MTND6P16;MTND5P13;MTCO3P28;MTCO3P27;MTCYBP16;MTND6P16;MTND5P13;MTCO3P28
4	72431001	72432000	ADAMTS3
4	74792001	74793000	BTC
4	77170001	77171000	CCNG2;LOC107986292
4	78108001	78109000	FRAS1
4	79704001	79705000	LINC02469;LOC105377302
4	80687001	80688000	CFAP299
4	82974001	82975000	LIN54
4	86073001	86074000	MAPK10
4	86254001	86255000	MAPK10

4	88191001	88192000	ABCG2;RNU6-818P
4	88206001	88207000	ABCG2;RNU6-818P;RNU6ATAC31P
4	88401001	88402000	HERC6;LOC102723458
4	88920001	88921000	FAM13A;LOC105377327
4	90157001	90158000	CCSER1
4	90230001	90231000	CCSER1
4	90298001	90299000	CCSER1
4	92274001	92275000	LNCPRESS2
4	94639001	94640000	PDLIM5
4	95025001	95026000	BMPR1B
4	95332001	95333000	UNC5C
4	98555001	98556000	TSPAN5
4	107292001	107293000	LOC102725220
4	109013001	109014000	COL25A1
4	112764001	112765000	ANK2
4	115144001	115145000	MRPS33P3
4	118828001	118829000	SEC24D
4	119064001	119065000	SYNPO2
4	120744001	120745000	PRDM5
4	121180001	121181000	TNIP3
4	122944001	122945000	SPATA5
4	123136001	123137000	SPATA5
4	124284001	124285000	LOC105377406
4	124394001	124395000	LOC105377408
4	127654001	127655000	INTU
4	136845001	136846000	LINC02511
4	139633001	139634000	LOC101927490
4	140414001	140415000	CLGN
4	141689001	141690000	IL15
4	142019001	142020000	INPP4B
4	145846001	145847000	ZNF827
4	146350001	146351000	SLC10A7
4	147797001	147798000	ARHGAP10
4	150156001	150157000	DCLK2
4	150470001	150471000	LRBA
4	151298001	151299000	SH3D19;PRSS48;RNU6-1282P
4	152867001	152868000	ARFIP1
4	153348001	153349000	TRIM2;MND1
4	153488001	153489000	TMEM131L
4	153522001	153523000	TMEM131L;LOC105377498
4	155455001	155456000	LOC105377503;MTCYBP17;MTND6P17;MTND5P9;MTND4P8;MTND4LP29;MTND3P3;MTCO3P9;MTATP6P9;MTCO2P9;MTCO1P9;MTND2P33;NMTRQ-TTG15-1;MTND1P22
4	155496001	155497000	LOC105377505
4	162712001	162713000	LOC105377516
4	163161001	163162000	NAF1
4	163633001	163634000	MARCHF1;LOC107986325
4	171904001	171905000	GALNTL6
4	174403001	174404000	LOC105377547
4	181866001	181867000	TENM3
4	181895001	181896000	TENM3;CCNHP1
4	181986001	181987000	TENM3
4	182807001	182808000	TENM3
4	183389001	183390000	LOC100533630
4	184186001	184187000	ENPP6
4	185607001	185608000	SORBS2
4	186204001	186205000	CYP4V2
4	186325001	186326000	F11-AS1;SLC25A5P6
4	186485001	186486000	F11-AS1
4	187369001	187370000	LOC339975;LINC02514
4	187481001	187482000	LOC339975
4	188158001	188159000	TRIML1;LINC02434
4	188773001	188774000	LOC101930028;LINC02508
4	189789001	189790000	FRG1-DT
4	189881001	189882000	FRG1-DT;LINC01596
4	190122001	190123000	LOC107986338
5	51001	52000	LOC105374602;LOC100128803
5	157001	158000	PLEKHG4B;LOC107986394
5	164001	165000	PLEKHG4B;LOC107986394
5	216001	217000	CCDC127;SDHA
5	221001	222000	CCDC127;SDHA
5	481001	482000	SLC9A3;PP7080;SLC9A3-AS1
5	574001	575000	LOC105374607
5	753001	754000	ZDHHC11B
5	798001	799000	ZDHHC11
5	904001	905000	TRIP13
5	1029001	1030000	NKD2
5	1060001	1061000	SLC12A7;MIR4635
5	1366001	1367000	LINC01511;MTCO2P32
5	1429001	1430000	SLC6A3
5	1514001	1515000	LPCAT1;MIR6075
5	1637001	1638000	LOC728613
5	2004001	2005000	LOC105374618
5	4138001	4139000	LINC02063
5	6774001	6775000	LOC107986401;LOC102724943
5	7114001	7115000	LINC02196
5	7289001	7290000	LOC100130063
5	10308001	10309000	CMBL;LOC105374651

5	14405001	14406000	TRIO
5	14635001	14636000	CCT6P2
5	14712001	14713000	OTULIN;ANKH;LOC100130744
5	14867001	14868000	ANKH;UQCRBP3;HNRNPKP5
5	16505001	16506000	RETREG1
5	16699001	16700000	MYO10
5	18538001	18539000	RN7SL58P
5	21479001	21480000	GUSBP1
5	26877001	26878000	CDH9
5	32161001	32162000	GOLPH3
5	32958001	32959000	LINC02120
5	36120001	36121000	LMBRD2
5	37602001	37603000	WDR70
5	37905001	37906000	LINC02117
5	45347001	45348000	HCN1
5	54246001	54247000	ARL15
5	54740001	54741000	LOC102467080;LOC105378969
5	55927001	55928000	IL31RA;IL6ST
5	57518001	57519000	LOC102724122
5	60322001	60323000	PDE4D
5	60427001	60428000	PDE4D;RPL31P8
5	60780001	60781000	ELOVL7
5	60863001	60864000	ERCC8
5	61698001	61699000	C5orf64;LOC101928651
5	65992001	65993000	ERBIN
5	66751001	66752000	MAST4
5	67624001	67625000	LINC02242
5	68517001	68518000	LOC105379012;LOC105379011
5	69639001	69640000	LOC728488;GUSBP3
5	69652001	69653000	GUSBP3;LOC728499
5	69902001	69903000	LOC728506;GUSBP13
5	70071001	70072000	SMN2
5	70220001	70221000	GUSBP14;LOC643367
5	70973001	70974000	NAIP;LOC112267942
5	71222001	71223000	LOC728452;GUSBP17
5	71609001	71610000	MCCC2
5	73151001	73152000	LOC105379030;LOC101060067
5	75334001	75335000	HMGCR
5	76019001	76020000	SV2C
5	76258001	76259000	SV2C
5	76880001	76881000	S100Z
5	79100001	79101000	BHMT2
5	79427001	79428000	HOMER1
5	82933001	82934000	LOC107986430;LOC105379051
5	83178001	83179000	XRCC4
5	83798001	83799000	RNU4-11P
5	83811001	83812000	RNU4-11P
5	90821001	90822000	ADGRV1
5	94567001	94568000	KIAA0825;MTND5P12;MTND6P3;MTCYBP35;LOC105379086
5	95849001	95850000	LOC102724720;LINC01554
5	108846001	108847000	FER
5	109010001	109011000	FER;RNU6-47P
5	111303001	111304000	CAMK4
5	113752001	113753000	LOC105379127
5	116618001	116619000	SEMA6A-AS2
5	116945001	116946000	LOC105379137
5	118384001	118385000	LINC02208
5	119445001	119446000	RNA5SP190;HSD17B4
5	120331001	120332000	RNU6-718P
5	124881001	124882000	LOC105379157
5	125363001	125364000	LOC101927421
5	126672001	126673000	LOC105379162;BOLA3P3
5	132680001	132681000	IL4;LOC105379176;KIF3A
5	133395001	133396000	FSTL4;CTB-3M24.3
5	134405001	134406000	CDKN2AIPNL
5	134848001	134849000	C5orf24
5	135588001	135589000	CXCL14;SLC25A48
5	138785001	138786000	CTNNA1
5	139581001	139582000	UBE2D2
5	139734001	139735000	PSD2;PSD2-AS1
5	139783001	139784000	PSD2
5	140370001	140371000	SLC4A9
5	140638001	140639000	TMCO6;CD14;NDUFA2;IK;MIR3655
5	141531001	141532000	DIAPH1
5	141854001	141855000	PCDH1
5	141857001	141858000	PCDH1
5	142332001	142333000	SPRY4;SPRY4-AS1
5	142407001	142408000	SPRY4-AS1
5	149772001	149773000	PPARGC1B
5	151688001	151689000	SPARC;CLMAT3
5	157345001	157346000	CYFIP2;FNDC9
5	157694001	157695000	SOX30P1;LOC105377676
5	159980001	159981000	ADRA1B
5	160185001	160186000	FABP6;FABP6-AS1
5	168302001	168303000	WWC1
5	170589001	170590000	KCNIP1
5	171062001	171063000	RANBP17

5	171088001	171089000	RANBP17
5	171509001	171510000	LOC105377720
5	172064001	172065000	STK10
5	172992001	172993000	ATP6V0E1
5	173143001	173144000	CREBRF;BNIP1
5	174403001	174404000	LINC01411
5	175488001	175489000	SFXN1
5	176553001	176554000	RNF44;CDHR2;RN7SL684P
5	176638001	176639000	SNCB;MIR4281;EIF4E1B;TSPAN17
5	176824001	176825000	UNC5A
5	177319001	177320000	MXD3
5	177474001	177475000	DBN1;PDLIM7
5	177613001	177614000	B4GALT7;LOC202181
5	177960001	177961000	LOC100128340
5	178210001	178211000	HNRNPAB;PHYKPL
5	178291001	178292000	COL23A1
5	178366001	178367000	COL23A1;LOC105377757
5	178537001	178538000	COL23A1
5	178582001	178583000	COL23A1
5	178653001	178654000	VN2R2P
5	178793001	178794000	AACSP1
5	179102001	179103000	ADAMTS2
5	179890001	179891000	TBC1D9B
5	180063001	180064000	RNF130
5	180542001	180543000	CNOT6;LOC100329129
5	180670001	180671000	LOC100420514
5	180779001	180780000	MGAT1
6	198001	199000	LOC285766
6	326001	327000	DUSP22
6	471001	472000	LOC105374875
6	1047001	1048000	LINC01622
6	1069001	1070000	LINC01622
6	1402001	1403000	FOXF2
6	1581001	1582000	LOC107986555
6	1597001	1598000	FOXCUT
6	1944001	1945000	GMDS
6	2050001	2051000	GMDS
6	2065001	2066000	GMDS
6	3283001	3284000	PSMG4;SLC22A23
6	3608001	3609000	LOC100507336
6	4340001	4341000	LOC101927888
6	4513001	4514000	LOC105374894
6	5742001	5743000	FARS2
6	6734001	6735000	LOC101928004;LOC101928047
6	7526001	7527000	LOC644051
6	7616001	7617000	SNRNP48;RPL29P1
6	7841001	7842000	BMP6
6	8276001	8277000	LOC105374911
6	8773001	8774000	LOC100506207
6	11726001	11727000	ADTRP;LOC107986566;LOC107986569
6	11788001	11789000	ADTRP
6	12198001	12199000	HIVEP1
6	13158001	13159000	PHACTR1
6	14708001	14709000	LOC101928354
6	15500001	15501000	JARID2
6	16515001	16516000	ATXN1
6	16562001	16563000	ATXN1
6	17874001	17875000	KIF13A
6	17975001	17976000	KIF13A
6	19699001	19700000	LOC105374959
6	20308001	20309000	LOC101928573
6	20327001	20328000	LOC101928573;LOC107986576
6	21658001	21659000	CASC15
6	21730001	21731000	CASC15
6	22569001	22570000	LOC105374971;HDGFL1
6	23352001	23353000	LOC102724749;LOC105374976
6	26883001	26884000	POM121L6P;GUSBP2
6	26886001	26887000	POM121L6P;GUSBP2
6	26897001	26898000	POM121L6P;GUSBP2;LOC112267954
6	29473001	29474000	OR2H1;UBDP1;MAS1LP1
6	30032001	30033000	ZNRD1ASP;ETF1P1
6	30221001	30222000	TRIM26
6	30898001	30899000	DDR1;MIR4640;GTF2H4
6	30950001	30951000	MUCL3;HCG21
6	31573001	31574000	LOC100287329;LTA;TNF;LTB
6	31625001	31626000	AIF1;PRRC2A;SNORA38;MIR6832
6	31757001	31758000	MSH5-SAPCD1;MSH5;RNU6-850P;SAPCD1;SAPCD1-AS1;VWA7
6	33393001	33394000	RPL35AP4;KIFC1;RPL12P1
6	33447001	33448000	SYNGAP1;MIR5004;ZBTB9
6	34909001	34910000	ANKS1A
6	35335001	35336000	PPARD
6	36276001	36277000	PNPLA1
6	36949001	36950000	PI16
6	37021001	37022000	FGD2
6	37678001	37679000	MDGA1
6	39361001	39362000	KIF6;LOC107986594;LOC100124373
6	39378001	39379000	KIF6;LOC107986594

6	40992001	40993000	LOC101929555
6	41436001	41437000	LOC107986538
6	41461001	41462000	LOC112267957
6	41652001	41653000	MDFI
6	41750001	41751000	PGC
6	41916001	41917000	MED20;BYSL
6	41925001	41926000	MED20;BYSL;CCND3
6	43266001	43267000	TTBK1
6	44095001	44096000	POLR1C;RPL29P16
6	45554001	45555000	RUNX2;RUNX2-AS1
6	49796001	49797000	PGK2
6	52152001	52153000	MIR206;LINCMD1;MIR133B
6	52521001	52522000	TRAM2
6	57927001	57928000	GUSBP4;LINC00680-GUSBP4
6	68920001	68921000	ADGRB3
6	70785001	70786000	SMAP1
6	71068001	71069000	BECN1P2
6	72007001	72008000	RIMS1
6	73273001	73274000	KHDC1
6	73300001	73301000	KHDC1;RPSAP41;EIF3EP1
6	73781001	73782000	CD109
6	77735001	77736000	MEI4
6	80051001	80052000	TTK
6	80511001	80512000	LOC112267962
6	82103001	82104000	LINC02542;LOC107986617
6	83874001	83875000	CYB5R4
6	99390001	99391000	COQ3;PNISR
6	100596001	100597000	ASCC3
6	101791001	101792000	GRIK2
6	105029001	105030000	LIN28B
6	111232001	111233000	SLC16A10;LOC107986521
6	111430001	111431000	REV3L;BRD7P4
6	113229001	113230000	SOCS5P5
6	115961001	115962000	FRK
6	117292001	117293000	ROS1;RN7SKP18;RN7SKP51
6	119330001	119331000	MAN1A1;RNU6-194P
6	127594001	127595000	C6orf58;LOC105377995
6	128580001	128581000	EEF1DP5
6	129264001	129265000	LAMA2
6	129809001	129810000	B3GALNT2P1
6	131295001	131296000	LOC107986643
6	131408001	131409000	LOC105378005
6	134930001	134931000	ALDH8A1
6	135118001	135119000	LOC105378010
6	136882001	136883000	PEX7
6	141455001	141456000	LOC105378029
6	149260001	149261000	TAB2;TAB2-AS1
6	151093001	151094000	MTHFD1L;RPL32P16;MIR12131
6	151312001	151313000	AKAP12;RN7SKP268
6	151953001	151954000	ESR1
6	155076001	155077000	TIAM2
6	155122001	155123000	TIAM2
6	155879001	155880000	LOC105378072;LOC101928923
6	156035001	156036000	LOC101928923
6	156362001	156363000	LOC105378071;LOC107986662
6	157311001	157313000	TMEM242;LOC112267967;TMEM242;LOC112267967
6	157515001	157516000	ZDHHC14
6	157774001	157775000	SNX9
6	158093001	158094000	SYNJ2
6	159177001	159178000	LOC101929122;FNDC1
6	159195001	159196000	FNDC1
6	160069001	160070000	IGF2R
6	160956001	160957000	LOC102724087
6	161743001	161744000	PRKN
6	162044001	162045000	PRKN
6	162994001	162995000	PACRG
6	163218001	163219000	PACRG;LOC105378095
6	163665001	163666000	LOC102724152;LOC107986666
6	166422001	166423000	RPS6KA2
6	166857001	166858000	RPS6KA2
6	167201001	167202000	TCP10L2;LOC100420305
6	167522001	167523000	LOC107986546
6	167795001	167796000	LINC01558;LOC441179
6	168078001	168079000	FRMD1
6	168234001	168235000	LOC105378137;LOC101929420
6	168465001	168466000	SMOC2
6	170467001	170468000	LOC105378157
6	170720001	170721000	LOC101929692
6	170726001	170727000	LOC101929692
7	10001	11000	LOC102723872
7	150001	151000	LOC102723672;LOC100507642
7	164001	165000	LOC100507642;LOC105375115
7	178001	179000	LOC105375115;LOC105375116
7	378001	379000	LOC442497
7	389001	390000	LOC442497
7	628001	629000	PRKAR1B
7	823001	824000	SUN1

7	946001	947000	ADAP1
7	1004001	1005000	C7orf50
7	1509001	1510000	INTS1
7	1562001	1563000	TMEM184A;PSMG3;PSMG3-AS1
7	1586001	1587000	PSMG3-AS1;LOC105375122
7	1599001	1600000	PSMG3-AS1;LOC105375122;LOC105375123
7	1736001	1737000	ELFN1;ELFN1-AS1
7	1911001	1912000	MAD1L1
7	1959001	1960000	MAD1L1
7	1984001	1985000	MAD1L1
7	2044001	2045000	MAD1L1
7	2112001	2113000	MAD1L1;LOC105375127
7	2146001	2147000	MAD1L1
7	2281001	2282000	SNX8;IMMP1LP3
7	2371001	2372000	EIF3B;KIF19BP
7	2609001	2610000	IQCE;LOC107986760
7	2734001	2735000	AMZ1;GNA12
7	2913001	2914000	CARD11
7	2939001	2940000	CARD11;CARD11-AS1
7	3938001	3939000	SDK1
7	4207001	4208000	SDK1
7	4709001	4710000	FOXK1
7	5077001	5078000	RBAK-RBAKDN;RBAK;RBAKDN
7	5226001	5227000	WIPI2
7	5328001	5329000	TNRC18
7	5560001	5561000	LOC100288712
7	5654001	5655000	RNF216;RNF216-IT1
7	6126001	6127000	USP42
7	6656001	6657000	ZNF316
7	6741001	6742000	PMS2CL
7	6923001	6924000	ALG1L5P;FAM86LP
7	6958001	6959000	LOC112267992;LOC107986763
7	8353001	8354000	LOC100505938
7	12498001	12499000	LOC105375156;LOC102725191;TAS2R2P
7	14174001	14175000	DGKB
7	16217001	16218000	CRPPA;RPL36AP29;CRPPA-AS1
7	17136001	17137000	LOC107986772;LOC101927609
7	20969001	20970000	LINC01162
7	21461001	21462000	SP4;MIR1183
7	21674001	21675000	DNAH11
7	23194001	23195000	NUP42
7	24970001	24971000	OSBPL3
7	25604001	25605000	LOC646588
7	25704001	25705000	LOC646588
7	27274001	27275000	RPL35P4
7	30440001	30441000	NOD1
7	31205001	31206000	LOC107986781
7	32514001	32515000	AVL9
7	34301001	34302000	RNU6-438P
7	36403001	36404000	ANLN
7	36671001	36672000	AOAH
7	38188001	38189000	STARD3NL
7	39543001	39544000	LOC105375240
7	39774001	39775000	LINC00265;RNU6-719P
7	40148001	40149000	SUGCT;THUMPD3P1
7	42870001	42871000	LOC107986735
7	42873001	42874000	LOC107986735
7	42918001	42919000	C7orf25;PSMA2;LOC105375252
7	43319001	43320000	HECW1
7	44002001	44003000	POLR2J4;SPDYE1
7	44853001	44854000	H2AZ2;LINC01952
7	46326001	46327000	LOC105375265
7	47547001	47548000	TNS3
7	47977001	47978000	HUS1;LOC105375270;SUN3
7	54777001	54778000	SEC61G-DT
7	55443001	55444000	LANCL2;VOPP1
7	56484001	56485000	LOC100240728;RBM22P3
7	56804001	56805000	LOC112267979;LOC100533652;CICP28;LOC401357;LOC112267995
7	56993001	56994000	TNRC18P3
7	57189001	57190000	GUSBP10;MTND1P4;NMTRQ-TTG13-1;MTND2P6;MTCO1P10;MTCO2P10;MTATP6P10;MTCO3P4;MTND4LP4;MTND4P5;MTND5P7;MTCYBP5
7	57240001	57241000	LOC105375298
7	63565001	63566000	SLC29A4P2;TNRC18P2
7	63772001	63773000	LOC102724777;CICP24;LOC112267980
7	64258001	64259000	ZNF679
7	64535001	64536000	ZNF680
7	64875001	64876000	ZNF273
7	66393001	66394000	LINC00174
7	66695001	66696000	RABGEF1
7	69810001	69811000	AUTS2
7	70747001	70748000	AUTS2;LOC105375347
7	71598001	71599000	GALNT17
7	71656001	71657000	GALNT17
7	71888001	71889000	CALN1
7	72869001	72870000	SPDYE7P;POM121
7	72912001	72913000	POM121
7	72964001	72965000	NSUN5P2;TRIM74;LOC100101148

7	72995001	72996000	TRIM74;STAG3L3
7	73083001	73084000	SPDYE9;PMS2P6
7	73191001	73192000	GTF2IP4;PHBP5
7	73322001	73323000	TRIM50;FKBP6
7	75004001	75005000	CASTOR2
7	75342001	75343000	SPDYE15;PMS2P2
7	75499001	75500000	SPDYE5;PMS2P3
7	75585001	75586000	HIP1
7	75705001	75706000	HIP1
7	75780001	75783000	CCL26;CCL26;CCL26
7	75947001	75948000	POR;SNORA14A
7	76021001	76022000	STYXL1
7	76540001	76541000	SPDYE16;LOC100133091
7	76991001	76992000	DTX2P1;DTX2P1-UPK3BP1-PMS2P11
7	77058001	77059000	PMS2P9;SPDYE18
7	77065001	77066000	SPDYE18;LOC100422695
7	77708001	77709000	RSBN1L
7	78272001	78273000	MAGI2
7	79234001	79235000	MAGI2
7	84065001	84066000	SEMA3A
7	96291001	96292000	SLC25A13;RPL21P74
7	97160001	97161000	SDHAF3;LOC107984034;LOC105375417
7	98175001	98176000	LMTK2
7	100151001	100152000	LAMTOR4;TRAPPC14;MIR4658;GAL3ST4
7	100447001	100448000	PPP1R35-AS1;C7orf61
7	100846001	100847000	SLC12A9;SLC12A9-AS1;LOC107986830
7	100951001	100953000	LOC105375431;MUC3A;LOC105375431;MUC3A
7	100958001	100959000	LOC105375431;MUC3A
7	101010001	101011000	MUC12;MUC12-AS1;MUC17
7	101194001	101195000	MOGAT3;DGAT2L7P;RPSAP46
7	101206001	101207000	MOGAT3;DGAT2L7P;RPSAP46;PLOD3
7	101936001	101937000	CUX1
7	102157001	102158000	CUX1
7	102205001	102206000	CUX1
7	102216001	102217000	CUX1
7	102469001	102470000	ALKBH4;LRWD1;MIR5090;MIR4467;POLR2J;RASA4B
7	102493001	102494000	RASA4B
7	102529001	102530000	POLR2J3;UPK3BL2
7	102555001	102556000	POLR2J3;SPDYE2
7	102559001	102560000	POLR2J3;SPDYE2
7	102622001	102623000	RASA4
7	102628001	102629000	POLR2J2;UPK3BL1
7	102638001	102639000	POLR2J2;UPK3BL1
7	103109001	103110000	ARMC10;NAPEPLD
7	104933001	104934000	LHFPL3-AS2;KMT2E
7	105853001	105854000	ATXN7L1
7	105865001	105866000	ATXN7L1
7	106712001	106713000	CTB-30L5.1
7	106868001	106869000	PIK3CG
7	112922001	112923000	BMT2
7	114492001	114493000	FOXP2
7	114679001	114680000	FOXP2
7	115029001	115030000	MDFIC
7	116972001	116973000	ST7;TPM3P1
7	120009001	120010000	RNU1-29P
7	129475001	129476000	STRIP2;SNRPGP3;RNU1-72P
7	129883001	129884000	UBE2H
7	131561001	131562000	PODXL
7	131943001	131944000	LOC101928782
7	133903001	133904000	EXOC4
7	134766001	134767000	LOC107986850
7	135316001	135317000	LOC107984123;SLC23A4P
7	135384001	135385000	CNOT4
7	135559001	135560000	NUP205
7	136012001	136013000	LOC105375523
7	137730001	137731000	DGKI;RPL6P19
7	139465001	139466000	KLRG2
7	140007001	140008000	TBXAS1
7	142780001	142781000	TRB;PRSS2;WBP1LP1;TRBD1;TRBJ1-1;TRBJ1-2;TRBJ1-3;TRBJ1-4;TRBJ1-5;TRBJ1-6
7	143353001	143354000	CLCN1;FAM131B;FAM131B-AS1
7	143539001	143540000	RPL26P22;PAICSP5
7	143787001	143788000	PAICSP6;RPL26P24
7	144232001	144233000	ARHGEF35-AS1;OR2A42;OR2A1-AS1
7	144277001	144278000	ARHGEF35-AS1;OR2A1-AS1;ARHGEF34P;CTAGE8
7	144372001	144373000	ARHGEF5
7	144375001	144376000	ARHGEF5
7	144387001	144388000	ARHGEF5;NOBOX
7	144775001	144776000	TPK1
7	146363001	146364000	CNTNAP2
7	148640001	148641000	LOC643438;LOC100301516
7	149021001	149022000	PDIA4
7	150270001	150271000	ACTR3C
7	150277001	150278000	ACTR3C
7	150387001	150388000	ZNF775;ZNF775-AS1;LOC107986859
7	150791001	150792000	LOC105375566;TMEM176B;TMEM176A
7	151715001	151716000	PRKAG2
7	151781001	151782000	PRKAG2

7	151895001	151896000	RNU6-604P
7	152384001	152385000	KMT2C
7	152403001	152404000	KMT2C
7	152816001	152817000	ACTR3B
7	153282001	153283000	LOC102723686
7	153951001	153952000	DPP6
7	154028001	154029000	DPP6
7	154420001	154421000	DPP6
7	154646001	154647000	DPP6
7	155209001	155210000	LOC105375586
7	155255001	155256000	LOC105375587;LOC105375588
7	155285001	155286000	INSIG1-DT
7	156962001	156963000	NOM1
7	157521001	157522000	LOC105375610
7	157581001	157582000	PTPRN2;MIR153-2
7	157620001	157621000	PTPRN2;LOC105375615;LOC105375614
7	157725001	157726000	PTPRN2
7	157895001	157896000	PTPRN2
7	158151001	158152000	PTPRN2
7	158274001	158275000	PTPRN2
7	158324001	158325000	PTPRN2
7	158595001	158596000	PTPRN2;LINC01022;MIR5707
7	158925001	158926000	DYNC2I1
7	159011001	159012000	LINC00689
8	300001	301000	LOC105377773
8	977001	978000	DLGAP2
8	1297001	1298000	DLGAP2;LOC286083
8	1316001	1317000	DLGAP2
8	1320001	1321000	DLGAP2
8	1356001	1357000	DLGAP2
8	1373001	1374000	DLGAP2;LOC105379585
8	1520001	1521000	DLGAP2
8	1812001	1813000	LOC105377779;MIR596
8	1931001	1932000	ARHGEF10;LOC100131395
8	1947001	1948000	ARHGEF10;LOC100131395
8	2056001	2057000	MYOM2
8	2322001	2323000	LOC105377783
8	2652001	2653000	LOC101927815;LOC107986865
8	4308001	4309000	CSMD1
8	4453001	4454000	CSMD1
8	4834001	4835000	CSMD1;PAICSP4
8	6656001	6657000	MCPH1;MCPH1-AS1
8	7570001	7571000	LOC105377800;FAM90A21P;FAM90A22P;FAM90A23P
8	7578001	7579000	LOC105377800;FAM90A21P;FAM90A22P;FAM90A23P
8	8183001	8184000	FAM85B;ENPP7P1
8	8787001	8788000	MFHAS1
8	10144001	10145000	MSRA
8	10571001	10572000	LOC105379237
8	10725001	10726000	SOX7;LOC102723313
8	10898001	10899000	XKR6
8	11052001	11053000	XKR6;LOC101929269
8	11329001	11330000	MTMR9;LOC101929290;SLC35G5;TDH
8	11674001	11675000	GATA4
8	11789001	11790000	NEIL2;LOC105379243;SUB1P1;FDFT1
8	12197001	12198000	FAM86B1;FAM85A;ENPP7P12
8	12429001	12430000	ALG1L12P;FAM86B2;LOC100506990;DEFB109E
8	12588001	12589000	LOC729732
8	12595001	12596000	LOC729732
8	14506001	14507000	SGCZ
8	14804001	14805000	SGCZ
8	17584001	17585000	PDGFRL
8	17599001	17600000	PDGFRL;LOC107986919
8	18080001	18081000	ASAH1;LOC101929066;MRPS18CP3;LOC100133073
8	18827001	18828000	PSD3
8	19249001	19250000	LOC100128993;LOC442382;LOC105379300
8	19464001	19465000	CSGALNACT1
8	20152001	20153000	SLC18A1
8	22062001	22063000	DMTN
8	22720001	22721000	PEBP4
8	22788001	22789000	PEBP4
8	22804001	22805000	PEBP4;LOC105379325
8	22821001	22822000	PEBP4;LOC105379325
8	23348001	23349000	LOXL2;LOC100507156
8	26210001	26211000	LOC105379336
8	27545001	27546000	EPHX2
8	28037001	28038000	NUGGC
8	28722001	28723000	EXTL3
8	30234001	30235000	HSPA8P11
8	30392001	30393000	RBPMS-AS1;RBPMS
8	31074001	31075000	WRN
8	38236001	38237000	DDHD2
8	39010001	39011000	ADAM9;SNORD38D
8	41753001	41754000	ANK1
8	41796001	41797000	ANK1;LOC105379392
8	42274001	42275000	IKBKB-DT;IKBKB
8	47099001	47100000	LOC105375816;LOC105375817
8	47179001	47180000	LOC100287846

8	47768001	47769000	PRKDC
8	47946001	47947000	PRKDC;LOC105375818
8	47984001	47985000	MCM4;RNU6-519P;TCONS_00068220
8	48701001	48702000	LOC101929268;EFCAB1
8	48725001	48726000	EFCAB1
8	54042001	54043000	LOC105375839;LYPLA1
8	54103001	54104000	LYPLA1
8	55098001	55099000	XKR4
8	56913001	56914000	RNU6-13P
8	58915001	58916000	TOX
8	60229001	60230000	CA8
8	60554001	60555000	RAB2A
8	60853001	60854000	CHD7
8	61011001	61012000	CLVS1
8	66268001	66269000	LOC102724687
8	67604001	67605000	CPA6;LOC105375886
8	68973001	68974000	LINC01592
8	69869001	69870000	LOC100288097
8	73198001	73199000	LOC107986891
8	73806001	73807000	UBE2W
8	77416001	77417000	LOC105375909
8	79622001	79623000	LOC105375916;STMN2
8	79730001	79731000	LOC107986893
8	79785001	79786000	LINC01607;LOC101927040
8	79985001	79986000	TPD52;MRPS28
8	80158001	80159000	TPD52
8	80981001	80982000	PAG1
8	84468001	84469000	RALYL
8	84620001	84621000	RALYL
8	85725001	85726000	REXO1L12P;LOC101929601;REXO1L11P
8	85727001	85728000	LOC101929601;REXO1L11P
8	85743001	85744000	LOC101929601;REXO1L11P;LOC101929627;REXO1L10P
8	85767001	85768000	REXO1L9P;REXO1L2P
8	88687001	88688000	LOC105375630
8	93777001	93778000	TMEM67
8	94483001	94484000	RAD54B;VIRMA
8	94965001	94966000	NDUFAF6
8	97821001	97822000	LAPTM4B
8	98588001	98589000	STK3
8	98741001	98742000	STK3
8	100232001	100233000	SPAG1;LOC105375669;LOC105375668
8	100712001	100713000	PABPC1;MIR7705
8	101264001	101265000	LOC107984005;LINC02844
8	104455001	104456000	DPYS;LOC105375691
8	104565001	104566000	LRP12;NDUFA5P2
8	117426001	117427000	LOC105375716
8	122887001	122888000	ZHX2
8	123166001	123167000	TBC1D31;TRM-CAT1-1
8	123214001	123215000	FAM83A;MIR4663;C8orf76
8	124963001	124964000	ZNF572
8	127221001	127222000	CCAT1
8	135436001	135437000	LOC101927872
8	137413001	137414000	LOC101927915
8	137826001	137827000	LOC401478
8	137830001	137831000	LOC401478
8	138085001	138086000	LOC401478
8	138489001	138490000	FAM135B
8	139648001	139649000	KCNK9
8	140264001	140265000	TRAPPC9;LOC105375779
8	140351001	140352000	TRAPPC9
8	140794001	140795000	PTK2
8	141009001	141010000	PTK2
8	141133001	141134000	DENND3-AS1;DENND3
8	141229001	141230000	SLC45A4
8	141269001	141270000	SLC45A4
8	141414001	141415000	PTP4A3
8	142769001	142770000	LYNX1-SLURP2;SLURP2;LYNX1
8	142920001	142921000	CYP11B2;LOC105375794
8	143692001	143693000	ZNF707;LINC02878
8	143796001	143797000	IQANK1;SCRIB
8	144016001	144017000	SPATC1
8	144348001	144349000	TMEM249;FBXL6;SLC52A2
8	144576001	144577000	ARHGAP39
8	144624001	144625000	ARHGAP39
9	40001	41000	WASHC1;MIR1302-9HG;MIR1302-9;FAM138C
9	126001	127000	FOXD4;CBWD1;LOC105375942
9	3313001	3314000	RFX3
9	5104001	5105000	JAK2;INSL6;MTND1P11;MTND2P36;MTCO1P11;MTCO2P11;MTATP6P11;MTCO3P11;MTND3P14;MTND4LP6;MTND4P14;MTND5P14;TCF3P1;LOC107987044;IGHEP2
9	15765001	15766000	CCDC171
9	16768001	16769000	BNC2;LSM1P1
9	17203001	17204000	CNTLN
9	19706001	19707000	SLC24A2;C11orf98P1
9	20232001	20233000	SLC24A2
9	22660001	22661000	LINC01239;LOC107987054
9	30802001	30803000	KRT18P36
9	32393001	32394000	ACO1

9	34182001	34183000	UBAP1;LOC114224;RPL35AP2
9	34720001	34721000	CCL21;FAM205A
9	34935001	34936000	PHF24
9	35362001	35363000	UNC13B
9	36409001	36410000	RNF38;MRPS21P4
9	36615001	36616000	MELK
9	36900001	36901000	PAX5;MIR4476
9	38198001	38199000	LOC107987064
9	38483001	38484000	TCEA1P3
9	40270001	40272000	ANKRD20A2P;ANKRD20A2P
9	41002001	41003000	LOC107986997;FRG1HP;PGM5P2
9	41268001	41269000	LOC107986999;LOC105376060
9	41354001	41355000	LOC107984035
9	42900001	42901000	ANKRD20A7P;LOC112268044
9	61203001	61204000	SPATA31A7;FAM74A4
9	62415001	62416000	FGF7P6
9	62805001	62806000	LINC01410;RNA5SP283
9	62810001	62811000	LINC01410;RNA5SP283
9	62832001	62833000	LOC100132249;PTGER4P2-CDK2AP2P2
9	63776001	63777000	CDK2AP2P3;PTGER4P3;LOC107987021
9	63824001	63825000	LINC00537;DUX4L50;MIR4477B;FRG1JP
9	64487001	64488000	CNN2P3;GXYLT1P6
9	65198001	65199000	BMS1P12
9	65714001	65715000	CBWD5;LOC105379250
9	66174001	66175000	LOC642929;MEP1AP3
9	67389001	67390000	LOC101927602
9	67907001	67908000	ANKRD20A1;LOC644249
9	69130001	69131000	TJP2
9	69407001	69408000	LOC105376074
9	70038001	70039000	MAMDC2
9	71201001	71202000	TRPM3
9	72613001	72614000	TMC1
9	72840001	72841000	TMC1
9	75994001	75995000	PCSK5
9	76034001	76035000	PCSK5
9	76175001	76176000	PCSK5
9	81773001	81774000	LOC101927502
9	81881001	81882000	RNA5SP287;LOC105376108
9	85021001	85022000	NTRK2;LOC105376118
9	87413001	87414000	LOC112268032
9	87890001	87891000	LOC497256;SPATA31E1
9	88180001	88181000	LOC389768;LOC100129340
9	88381001	88382000	SPIN1
9	89377001	89378000	SECISBP2;SEMA4D
9	90206001	90207000	OR7E31P
9	91043001	91044000	LOC100507103
9	91893001	91894000	ROR2;LOC101927935
9	92077001	92078000	SPTLC1
9	92109001	92110000	SPTLC1;LOC100420896;MTATP6P29;MTCO3P29;MTND3P23;MTND4P15
9	92554001	92555000	CENPP
9	93294001	93295000	WNK2
9	93515001	93516000	FAM120A
9	94261001	94262000	LINC02603;ZNF169
9	94277001	94278000	ZNF169;VDAC1P11
9	94934001	94935000	AOPEP;LOC101928119
9	94972001	94973000	AOPEP
9	95543001	95544000	LOC105376157
9	97516001	97517000	TMOD1;LOC105376168
9	97790001	97791000	PTCSC2
9	98336001	98337000	GABBR2
9	100731001	100732000	ACTG1P19
9	104949001	104950000	LOC105376196
9	105213001	105214000	LOC112268038
9	105494001	105495000	FSD1L
9	106932001	106933000	ZNF462
9	108031001	108032000	RPL31P43
9	110169001	110170000	PALM2AKAP2;LOC105376217
9	110209001	110210000	C9orf152
9	110792001	110793000	MUSK;LOC107987115
9	111135001	111136000	LOC105376219
9	111637001	111638000	LOC107987116;DNAJC25-GNG10;DNAJC25
9	113542001	113543000	RGS3
9	113632001	113633000	LOC105376222
9	114208001	114209000	COL27A1;MIR455
9	114659001	114660000	TEX53;LOC107987121;TEX48
9	115110001	115111000	TNC;LOC101928748
9	116364001	116365000	PAPPA;PAPPA-AS2
9	119228001	119229000	BRINP1
9	120448001	120449000	CDK5RAP2
9	121566001	121567000	LOC107987016;DAB2IP
9	121983001	121984000	TTLL11
9	121995001	121996000	TTLL11
9	122530001	122531000	OR1J2;OR1J4;OR1N1
9	123057001	123058000	RABGAP1;LOC100422501
9	124628001	124629000	NR6A1
9	127908001	127909000	ST6GALNAC6;ST6GALNAC4
9	128102001	128103000	SLC25A25;SLC25A25-AS1

9	129055001	129056000	MIGA2
9	129158001	129159000	PTPA
9	129261001	129262000	LOC105376290
9	129428001	129429000	LOC105376291
9	129523001	129524000	LINC00963
9	129690001	129691000	PRRX2
9	129724001	129725000	PRRX2;PRRX2-AS1
9	129977001	129978000	FNBP1
9	130066001	130067000	GPR107
9	130478001	130480000	ASS1;ASS1
9	130692001	130693000	PRDM12;EXOSC2
9	130904001	130905000	QRFP;FIBCD1
9	131567001	131568000	PRRT1B;RAPGEF1
9	132176001	132177000	NTNG2
9	132352001	132353000	SETX
9	132451001	132452000	CFAP77
9	133717001	133718000	SARDH
9	133856001	133857000	VAV2
9	134172001	134173000	RNU6ATAC
9	134541001	134542000	LOC100506532
9	135261001	135262000	LOC107987138
9	135456001	135457000	SOCS5P2;PPP1R26-AS1
9	135679001	135680000	LCN9;LOC105376317;LOC105376318
9	135682001	135683000	LOC105376317;LOC105376318;SOHLH1
9	135733001	135734000	KCNT1
9	136247001	136248000	QSOX2;CCDC187
9	136408001	136409000	SNAPC4;ENTR1;PMPCA
9	136446001	136447000	INPP5E;SEC16A
9	137023001	137024000	ABCA2;LINC02908;FUT7
9	137050001	137051000	NPDC1;ENTPD2;LOC105376327
9	137327001	137328000	EXD3
9	137378001	137379000	EXD3
9	137511001	137512000	PNPLA7
9	137786001	137787000	EHMT1;LOC651337;LOC100418938
9	137859001	137860000	LOC100133077
9	137911001	137912000	CACNA1B
10	10001	11000	LOC102723376
10	338001	339000	DIP2C
10	362001	363000	DIP2C;LOC105376340
10	830001	831000	LARP4B
10	995001	996000	LOC107984285;GTPBP4
10	3948001	3949000	LOC105376367
10	5966001	5967000	IL15RA;LOC107984200
10	6074001	6075000	RPL32P23
10	6478001	6479000	PRKCQ
10	6558001	6559000	PRKCQ
10	6800001	6801000	LINC00707
10	7590001	7591000	ITIH5
10	7634001	7635000	ITIH5
10	7856001	7857000	TAF3
10	10712001	10713000	CELF2
10	10921001	10922000	CELF2;LOC105376405
10	12065001	12066000	DHTKD1
10	12076001	12077000	DHTKD1
10	12177001	12178000	SEC61A2;NUDT5
10	12229001	12230000	CDC123
10	12556001	12557000	CAMK1D
10	12819001	12820000	CAMK1D
10	13167001	13168000	BTBD7P1;RPL36AP36;MCM10
10	15004001	15005000	OR7E26P;OR7E115P
10	15025001	15026000	ACBD7-DCLRE1CP1;DCLRE1CP1;OLAH
10	16841001	16842000	CUBN
10	17429001	17430000	ST8SIA6;LOC105376436
10	17721001	17722000	STAM
10	18364001	18365000	CACNB2
10	20806001	20807000	NEBL;MTND1P21
10	21089001	21090000	NEBL
10	22613001	22614000	PIP4K2A
10	23283001	23284000	C10orf67
10	24498001	24499000	KIAA1217
10	24758001	24759000	LOC105376456
10	24887001	24888000	PRTFDC1
10	26505001	26506000	APBB1IP;RNA5SP307
10	28293001	28294000	MPP7;MIR8086;MPP7-DT
10	28430001	28431000	LINC02652;RPSAP10
10	29640001	29641000	SVIL
10	29651001	29652000	SVIL
10	29695001	29696000	SVIL;CKS1BP2
10	29745001	29746000	SVIL
10	30393001	30394000	CCND3P1
10	31073001	31074000	LOC105376481
10	35005001	35006000	CUL2
10	35703001	35704000	RPL7P37
10	38448001	38449000	LINC00999;CICP9;SEPTIN14P10
10	38487001	38488000	LOC107984177
10	38489001	38490000	LOC107984177
10	42436001	42437000	CCNYL2

10	43119001	43120000	RET
10	43197001	43198000	RASGEF1A
10	43339001	43340000	LOC105378271;RNU6ATAC11P;LOC107984226
10	43842001	43843000	LINC00619
10	45573001	45574000	MARCHF8
10	48106001	48107000	PTPN20CP
10	58326001	58327000	LOC112268068;UBE2D1
10	63595001	63596000	REEP3;LOC105378329
10	67559001	67560000	CTNNA3
10	68847001	68848000	STOX1;RNU6-697P
10	69816001	69817000	COL13A1;LOC105378347
10	69950001	69951000	COL13A1;LOC107984242
10	69968001	69969000	COL13A1;LOC107984242
10	70295001	70296000	LRRC20
10	70975001	70976000	LOC105378350
10	72810001	72811000	MCU
10	72831001	72832000	MCU
10	73502001	73503000	PPP3CB;PPP3CB-AS1;USP54
10	73697001	73698000	BMS1P4-AGAP5;AGAP5;BMS1P4;RNA5SP320
10	74039001	74040000	VCL
10	76476001	76477000	LRMDA
10	77975001	77976000	POLR3A
10	79301001	79302000	ZMIZ1
10	79434001	79435000	ZCCHC24
10	79699001	79700000	NUTM2B-AS1;LOC107984248;NUTM2B
10	79861001	79862000	LOC100421010;NPAP1P2;CTSLP6
10	86665001	86666000	OPN4;LOC105378409;LDB3
10	89534001	89535000	SLC16A12
10	93108001	93109000	NIP7P1
10	96383001	96384000	TLL2
10	99178001	99179000	HPSE2
10	99527001	99528000	LINC01475;NKX2-3
10	99873001	99874000	DNMBP
10	99940001	99941000	DNMBP;DNMBP-AS1
10	101662001	101663000	FBXW4
10	101822001	101823000	OGA;KCNIP2-AS1;KCNIP2
10	101973001	101974000	ARMH3
10	102700001	102701000	ARL3
10	102790001	102791000	WBP1L
10	102809001	102810000	WBP1L;RNU6-1231P
10	103627001	103628000	SH3PXD2A
10	109916001	109917000	XPNPEP1
10	113034001	113035000	TCF7L2
10	113059001	113060000	TCF7L2
10	113349001	113350000	RNU7-165P
10	114274001	114275000	VWA2;AFAP1L2
10	114453001	114454000	ABLIM1
10	116356001	116357000	CCDC172
10	116572001	116573000	PNLIP;PNLIPP1
10	117083001	117084000	SHTN1
10	117232001	117233000	LOC105378500;SLC18A2
10	117433001	117434000	LOC105378502
10	118181001	118182000	CASC2
10	119050001	119051000	EIF3A;SNORA19
10	120792001	120793000	LOC105378516;WDR11-AS1
10	120912001	120913000	WDR11;LOC105378519
10	121977001	121978000	NSMCE4A
10	121997001	121998000	TACC2
10	124024001	124025000	CHST15
10	125835001	125836000	BCCIP;DHX32
10	125891001	125892000	DHX32;RNU2-42P;FANK1
10	126951001	126952000	DOCK1
10	126965001	126966000	DOCK1
10	127052001	127053000	DOCK1
10	127229001	127230000	DOCK1
10	128106001	128107000	MKI67
10	129567001	129568000	MGMT
10	129704001	129705000	MGMT;LOC105378560
10	129879001	129880000	EBF3
10	130206001	130207000	LINC02646
10	130964001	130965000	MIR378C
10	131218001	131219000	TCERG1L;LOC105378563
10	131770001	131771000	LINC01164
10	131866001	131867000	LOC105378565;LOC105378566
10	132189001	132190000	JAKMIP3;LOC105378567;DPYSL4
10	132352001	132353000	LRRC27
10	132362001	132363000	LRRC27
10	132364001	132365000	LRRC27
10	132423001	132424000	PWWP2B;LOC105378568
10	132589001	132590000	INPP5A
10	132697001	132698000	INPP5A
10	132762001	132763000	INPP5A
10	132864001	132865000	CFAP46;LOC105378571
10	132928001	132929000	CFAP46
10	132946001	132947000	CFAP46;LOC105378572;LINC01166
10	133133001	133134000	ADGRA1;RPL5P28
10	133252001	133253000	MIR202HG;MIR202;ADAM8

10	133454001	133455000	SCART1;LOC105378576
11	191001	192000	LOC388572;SCGB1C1;ODF3
11	268001	269000	NLRP6
11	301001	302000	PGGHG;IFITM5;MRPS24P1;IFITM2
11	411001	413000	PKP3;SIGIRR;ANO9;PKP3;SIGIRR;ANO9
11	642001	643000	DRD4;DEAF1
11	964001	966000	AP2A2;AP2A2
11	980001	981000	AP2A2
11	1048001	1049000	LINC02688
11	1110001	1111000	MUC2
11	1147001	1148000	MUC5AC
11	1192001	1193000	MUC5AC
11	1341001	1342000	LINC02689
11	1626001	1627000	KRTAP5-4;KRTAP5-5
11	1661001	1662000	FAM99A
11	1804001	1805000	LOC390029
11	1945001	1946000	TNNT3;MRPL23;SNORD131
11	2014001	2015000	MRPL23
11	2414001	2415000	TRPM5
11	2547001	2548000	KCNQ1
11	3146001	3147000	OSBPL5;LOC107987158
11	3482001	3483000	LOC105376526
11	4116001	4117000	RRM1
11	5104001	5105000	OR52J1P
11	5932001	5933000	LOC112268071;OR56A3
11	6075001	6076000	OR52X1P
11	6414001	6415000	APBB1
11	6642001	6643000	DCHS1
11	8351001	8352000	STK33
11	14023001	14024000	SPON1
11	14993001	14994000	OR7E41P
11	18385001	18386000	MIR3159;LDHA
11	19621001	19622000	NAV2
11	19758001	19759000	NAV2;MIR4694
11	20569001	20570000	LOC105376584;HMGB1P40
11	20833001	20834000	NELL1
11	22652001	22653000	GAS2
11	23184001	23185000	LINC02718
11	27222001	27223000	BBOX1-AS1;LOC105376600
11	27629001	27630000	BDNF-AS;LINC00678
11	30461001	30462000	MPPED2
11	30527001	30528000	MPPED2
11	32097001	32098000	RCN1
11	33576001	33577000	KIAA1549L
11	36109001	36110000	LDLRAD3
11	36554001	36555000	RAG1
11	39734001	39735000	LOC105376637
11	43721001	43722000	HSD17B12;RPL23AP63
11	44753001	44754000	TSPAN18
11	44903001	44904000	TSPAN18
11	45908001	45909000	MAPK8IP1;C11orf94;PEX16
11	46204001	46205000	LINC02710
11	46327001	46328000	CREB3L1;DGKZ
11	46605001	46606000	HARBI1
11	46849001	46850000	CKAP5;LRP4-AS1;LRP4
11	46895001	46896000	LRP4
11	47252001	47253000	ACP2;NR1H3
11	49121001	49122000	TRIM77BP
11	50077001	50078000	LOC101060224
11	57092001	57093000	LOC105369309
11	57309001	57310000	TNKS1BP1
11	57717001	57718000	MED19;TMX2-CTNND1;TMX2;PPIAP42
11	58921001	58922000	GLYATL1
11	61513001	61514000	LOC105369329;MIR4488;LRRC10B;SYT7
11	61551001	61552000	SYT7
11	61825001	61826000	FADS1;MIR1908;FADS2
11	61853001	61854000	FADS2
11	61905001	61906000	RAB3IL1
11	62237001	62238000	SCGB1D2
11	62496001	62497000	AHNAK
11	63607001	63608000	PLAAT3;LOC107984336
11	64203001	64204000	STIP1;FERMT3
11	64411001	64412000	LOC105369341
11	64559001	64560000	SLC22A11
11	64739001	64740000	RASGRP2;PYGM
11	64814001	64815000	MEN1;CDC42BPG
11	64828001	64829000	CDC42BPG
11	65397001	65398000	FRMD8
11	65619001	65620000	MAP3K11;PCNX3
11	66251001	66252000	PACS1;KLC2;KLC2-AS1
11	66626001	66627000	RBM14-RBM4;RBM14
11	66628001	66629000	RBM14-RBM4;RBM14;RBM4
11	66706001	66707000	SPTBN2;RN7SL12P
11	66984001	66985000	C11orf86
11	67633001	67634000	NUDT8;TBX10;ACY3
11	68161001	68162000	KMT5B
11	68334001	68335000	LRP5

11	68420001	68421000	LRP5
11	69666001	69667000	LTO1
11	69780001	69781000	FGF4
11	70148001	70149000	ANO1;LOC105369372
11	70273001	70274000	PPFIA1;H2AZP4;MIR548K
11	70411001	70412000	CTTN
11	70445001	70446000	CTTN
11	70526001	70527000	SHANK2
11	70792001	70793000	SHANK2
11	70843001	70844000	SHANK2
11	71234001	71235000	SHANK2
11	71917001	71918000	LOC100133315
11	72693001	72694000	ARAP1;ARAP1-AS2
11	72701001	72702000	ARAP1;ARAP1-AS2;RPS12P20
11	73100001	73101000	FCHSD2
11	73292001	73293000	P2RY6
11	73786001	73787000	MRPL48
11	73937001	73938000	PAAF1
11	74030001	74031000	C2CD3
11	74764001	74765000	RNF169
11	75242001	75243000	TPBGL
11	75529001	75530000	GDPD5;LOC105369389
11	76282001	76283000	LOC105369395
11	76697001	76698000	GUCY2EP
11	78204001	78205000	USP35
11	81554001	81555000	MTCO3P25;MTND3P11;MTND4P36;MTND5P38;MTND6P25;MTCYBP25
11	83216001	83217000	ANKRD42;CKS1BP4;RPL32P24
11	85326001	85327000	DLG2;LOC100419092
11	85484001	85485000	DLG2
11	87002001	87003000	FZD4-DT;LOC105369422
11	87815001	87816000	LOC107984361;RAB38;MTCYBP41
11	88056001	88057000	RAB38
11	88396001	88397000	LOC101929174
11	89745001	89746000	UBTFL2
11	94235001	94236000	LOC105369435
11	94432001	94433000	MRE11
11	95024001	95025000	KDM4E;LOC100420802
11	96445001	96446000	JRKL-AS1
11	102441001	102442000	TMEM123
11	102607001	102608000	MMP20;LOC101928477
11	103408001	103409000	DYNC2H1;MTCO3P15;MTATP6P15;MTCO2P15;MTCO1P15;MTND2P26;MTND1P36
11	108012001	108013000	CUL5
11	108026001	108027000	CUL5
11	108263001	108264000	ATM
11	108835001	108836000	DDX10
11	109867001	109868000	LOC105369483;LOC107984384
11	110792001	110793000	HNRNPA1P60
11	112746001	112747000	LOC105369496
11	113154001	113155000	NCAM1
11	114447001	114448000	REXO2;LOC100422382
11	115797001	115798000	LINC02698;LOC107987165
11	116346001	116347000	LOC107987166
11	116475001	116476000	LOC107987166
11	116752001	116753000	BUD13
11	116891001	116892000	SIK3
11	117209001	117210000	LOC100652768;TAGLN;PCSK7
11	117299001	117301000	BACE1;BACE1-AS;BACE1;BACE1-AS
11	117441001	117442000	DSCAML1
11	117518001	117519000	DSCAML1
11	117820001	117821000	DSCAML1;FXYD6-FXYD2;FXYD2
11	118315001	118316000	CD3E
11	118433001	118434000	KMT2A
11	118607001	118608000	ARCN1;PHLDB1
11	119383001	119384000	USP2;USP2-AS1
11	120028001	120029000	LOC105369527
11	120450001	120451000	ARHGEF12
11	120516001	120517000	GRIK4
11	120596001	120597000	GRIK4
11	120796001	120797000	GRIK4
11	120853001	120854000	GRIK4
11	122360001	122361000	MIR100HG
11	124895001	124896000	ROBO4;LOC107984406
11	125330001	125331000	PKNOX2
11	125392001	125393000	PKNOX2
11	126399001	126400000	ST3GAL4
11	126402001	126403000	ST3GAL4
11	129870001	129871000	TMEM45B;NFRKB
11	129874001	129875000	NFRKB
11	130144001	130145000	APLP2
11	130350001	130351000	ZBTB44-DT
11	130480001	130481000	ADAMTS15
11	132429001	132430000	OPCML
11	134387001	134388000	B3GAT1
12	10001	11000	DDX11L8;WASH8P
12	268001	269000	SLC6A13
12	314001	315000	KDM5A
12	504001	505000	B4GALNT3

12	1265001	1266000	ERC1
12	1623001	1624000	WNT5B
12	1920001	1921000	CACNA2D4;LOC112268103;LINC00940
12	2255001	2257000	CACNA1C;CACNA1C
12	2534001	2535000	CACNA1C
12	2589001	2590000	CACNA1C
12	2602001	2603000	CACNA1C
12	2972001	2973000	TEAD4
12	3108001	3109000	TSPAN9
12	3957001	3958000	LOC105369608
12	4115001	4116000	RPL18P9
12	4281001	4282000	CCND2-AS1;CCND2
12	4370001	4371000	TIGAR;FGF23
12	4639001	4640000	AKAP3;LOC100420673;NDUFA9
12	5119001	5120000	LOC105369617
12	5501001	5502000	NTF3
12	5589001	5590000	ANO2;LOC101901829
12	6119001	6120000	VWF
12	6548001	6549000	GAPDH;IFFO1;NOP2
12	6757001	6758000	MLF2;PTMS
12	7934001	7935000	SLC2A3
12	8624001	8625000	HADHAP2
12	8844001	8845000	A2ML1
12	9930001	9931000	CLEC2A;LINC02470
12	9962001	9963000	CLEC12A-AS1;CLEC12A
12	10179001	10180000	OLR1;TMEM52B
12	12964001	12965000	GPRC5D-AS1;GPRC5D;HEBP1
12	14235001	14236000	MRPS18CP4
12	14710001	14711000	C12orf60
12	19733001	19734000	RNU1-146P
12	20818001	20819000	SLCO1B3;SLCO1B3-SLCO1B7
12	21085001	21086000	SLCO1B3-SLCO1B7
12	21383001	21384000	SLCO1A2;IAPP
12	24877001	24878000	BCAT1
12	25169001	25170000	DNAI7
12	27783001	27784000	MANSC4;KLHL42
12	29140001	29141000	FAR2
12	31089001	31090000	DDX11
12	31247001	31248000	MTND5P43;MTND6P26;MTCYBP26;RPL13AP22;MREGP1
12	31758001	31759000	LOC105369724;IFITM3P2
12	32474001	32475000	FGD4;RNU6-494P
12	41545001	41546000	PDZRN4;LOC107984498
12	44111001	44112000	TMEM117
12	45795001	45796000	ARID2
12	46423001	46424000	LOC100288798
12	48783001	48784000	ADCY6;ADCY6-DT
12	50067001	50068000	ASIC1
12	50393001	50394000	FAM186A;LARP4
12	51491001	51492000	SLC4A8
12	51913001	51914000	ACVRL1
12	52084001	52085000	ATG101;SMIM41;OR7E47P;LOC112268096
12	52702001	52703000	KRT77
12	53399001	53400000	SP1
12	53410001	53411000	SP1
12	53620001	53621000	ATF7-NPFF;ATF7
12	54474001	54475000	LOC102724050;GTSF1
12	55686001	55687000	METTL7B;ITGA7
12	56194001	56195000	SMARCC2;TRS-CGA4-1;LOC107984468;RNF41
12	57248001	57249000	NDUFA4L2;STAC3;R3HDM2
12	57330001	57331000	R3HDM2
12	57477001	57478000	GLI1;ARHGAP9
12	66791001	66792000	GRIP1
12	68314001	68315000	LOC105369818;MDM1
12	69473001	69474000	FRS2
12	71875001	71876000	TBC1D15
12	73001001	73002000	LOC105369838
12	73005001	73006000	LOC105369838
12	75844001	75845000	LOC105369844
12	75982001	75983000	LOC105369844
12	80247001	80248000	OTOGL
12	80453001	80454000	PTPRQ
12	89499001	89500000	POC1B;CENPCP1
12	89973001	89974000	LOC105369890
12	93073001	93074000	LOC643339;RPL41P5
12	93394001	93395000	NUDT4
12	95005001	95006000	NDUFA12
12	95431001	95432000	LOC107984545;RNU6-735P
12	96728001	96729000	CFAP54
12	98750001	98751000	ANKS1B
12	99717001	99718000	ANKS1B
12	106142001	106143000	NUAK1
12	109908001	109909000	TCHP
12	110009001	110010000	ANKRD13A
12	110408001	110409000	ANAPC7
12	111078001	111079000	CUX2
12	111160001	111161000	CUX2
12	112193001	112194000	HECTD4

12	112483001	112484000	PTPN11
12	113195001	113196000	DDX54;RITA1;IQCD
12	113291001	113292000	TPCN1;MIR6762;LOC107984436;SLC8B1
12	113318001	113319000	SLC8B1
12	113845001	113846000	RBM19
12	113958001	113959000	RBM19;TRE-CTC16-1
12	115438001	115439000	LOC105370003
12	115618001	115619000	LOC105370003;LOC105370002
12	117053001	117055000	TESC;TESC
12	117312001	117313000	NOS1
12	119784001	119785000	CIT
12	120180001	120181000	GCN1
12	120217001	120218000	PXN-AS1;PXN
12	120278001	120279000	NME2P1
12	120502001	120503000	DYNLL1;LOC107987181;NRAV;COQ5
12	121005001	121006000	HNF1A;C12orf43
12	121614001	121615000	RNU6-1004P
12	121924001	121925000	PSMD9;CFAP251
12	122517001	122518000	RSRC2;KNTC1
12	123048001	123049000	PITPNM2
12	123493001	123494000	RILPL1
12	123580001	123581000	TMED2-DT;TMED2
12	123899001	123900000	DNAH10
12	124184001	124185000	ZNF664-RFLNA
12	124212001	124213000	ZNF664-RFLNA
12	124228001	124229000	ZNF664-RFLNA
12	124270001	124271000	ZNF664-RFLNA
12	124358001	124359000	NCOR2
12	125018001	125019000	BRI3BP;THRIL
12	125210001	125211000	TMEM132B
12	125296001	125297000	TMEM132B;LOC107984445
12	125299001	125300000	TMEM132B;LOC107984445
12	126152001	126153000	LOC107984447
12	127099001	127100000	LOC107984449
12	128323001	128324000	TMEM132C
12	128867001	128868000	GLT1D1
12	130440001	130441000	RIMBP2
12	130450001	130451000	RIMBP2
12	130808001	130809000	STX2
12	130999001	131000000	ADGRD1;ADGRD1-AS1
12	131012001	131013000	ADGRD1
12	131122001	131123000	ADGRD1
12	131798001	131799000	SFSWAP;RNA5SP378
12	132141001	132142000	LOC107987169;DDX51;NOC4L
12	132427001	132428000	LOC105370092
12	132577001	132578000	FBRSL1;MIR6763
12	132680001	132681000	POLE;PXMP2
12	132733001	132734000	RNA5SP379;ANKLE2
12	132781001	132782000	GOLGA3
12	132803001	132804000	GOLGA3
12	132807001	132808000	GOLGA3
12	132939001	132940000	ZNF605
13	19138001	19139000	CENPIP1;RNU6-52P
13	19498001	19499000	TPTE2
13	19692001	19693000	PSPC1
13	19735001	19736000	PSPC1;RN7SL166P
13	20518001	20519000	CRYL1
13	21533001	21534000	MICU2;FNTAP2
13	21554001	21555000	MICU2;RNU6-59P
13	23115001	23116000	LOC105370112
13	23193001	23194000	SGCG
13	25033001	25034000	LOC105370119
13	25749001	25750000	ATP8A2
13	27049001	27050000	LOC105370124
13	27449001	27450000	MTIF3
13	28019001	28020000	FLT3
13	28032001	28033000	FLT3
13	29243001	29244000	MTUS2
13	34483001	34484000	LINC02343;LINC00457
13	35318001	35319000	NBEA
13	36380001	36381000	SPART
13	38019001	38020000	LINC02334
13	41436001	41437000	OR7E36P;OR7E155P;OR7E37P
13	42064001	42065000	DGKH;MAPK6P3
13	43431001	43432000	ENOX1
13	44552001	44553000	TSC22D1
13	44927001	44928000	LOC107984619;TRE-TTC2-1
13	45485001	45486000	COG3
13	48034001	48035000	NUDT15
13	48428001	48429000	RB1;LPAR6
13	49018001	49019000	FNDC3A
13	49302001	49303000	CDADC1;CAB39L
13	49678001	49679000	EBPL
13	49729001	49730000	KPNA3
13	50172001	50173000	DLEU1;ST13P4
13	51863001	51864000	CCDC70
13	52243001	52244000	TPTE2P2

13	52724001	52725000	CNMD
13	57393001	57394000	RPL31P53
13	60326001	60327000	LOC105370228
13	60804001	60805000	LINC01442
13	66738001	66739000	PCDH9
13	67911001	67912000	OR7E111P;OR7E33P
13	67914001	67915000	OR7E33P
13	73034001	73035000	PSMD10P3
13	77200001	77201000	MYCBP2
13	85124001	85125000	LINC00375;LOC105370291
13	92462001	92463000	GPC5
13	95175001	95176000	ABCC4
13	95258001	95259000	ABCC4
13	95602001	95603000	DZIP1
13	99242001	99243000	UBAC2
13	99312001	99313000	UBAC2;GPR183
13	99524001	99525000	TM9SF2
13	100183001	100184000	PCCA
13	100962001	100963000	NALCN-AS1
13	103301001	103302000	LOC105370338
13	103391001	103392000	LOC105370339
13	103393001	103394000	LOC105370339
13	107217001	107218000	NALF1
13	107672001	107673000	NALF1
13	107782001	107783000	NALF1;NALF1-IT1;LOC107984581
13	108346001	108347000	LOC105370355
13	109544001	109545000	LOC107984602
13	110182001	110183000	COL4A1
13	110233001	110234000	COL4A1
13	111189001	111190000	ARHGEF7;SALL4P4
13	112462001	112463000	LOC105370372
13	112599001	112600000	TUBGCP3
13	112724001	112725000	ATP11A
13	112781001	112782000	ATP11A
13	113268001	113269000	CUL4A;MIR8075;LDHBP1
13	113418001	113419000	ADPRHL1
13	113832001	113833000	GAS6-AS1;GAS6
13	114125001	114126000	RASA3;LOC107987192;LOC107984593
13	114354001	114355000	LOC112268113
14	16034001	16035000	DUX4L48;PCMTD1P6;DUX4L49
14	18691001	18692000	RPL22P20;NEK2P3
14	19089001	19090000	DUXAP9;BMS1P18
14	19268001	19269000	NBEAP6;TOMM40P1;LINC01297-DUXAP10-NBEAP6
14	19861001	19862000	OR4K3
14	21163001	21164000	OR5AU1;SMARCE1P3
14	22259001	22260000	TRA;TRAV37
14	22638001	22639000	OR6J1
14	22807001	22808000	SLC7A7
14	23413001	23414000	MYH6;MYH7;MHRT;MIR208B
14	23684001	23685000	LOC105370408
14	24202001	24203000	TM9SF1;TSSK4;CHMP4A
14	24277001	24278000	RABGGTA;LOC102725044
14	24286001	24287000	LOC102725044;DHRS1
14	24931001	24932000	STXBP6
14	33623001	33624000	NPAS3
14	34468001	34469000	SPTSSA
14	35237001	35238000	PRORP;RPL7AP3
14	36705001	36706000	SLC25A21
14	39404001	39405000	FBXO33
14	47108001	47109000	MDGA2
14	48255001	48256000	LOC105370482
14	48260001	48261000	LOC105370482
14	49719001	49720000	KLHDC1
14	50945001	50946000	PYGL;LOC105370492;LOC400212
14	50956001	50957000	LOC105370492;LOC400212
14	51030001	51031000	TRIM9
14	54592001	54593000	SAMD4A
14	55440001	55441000	TBPL2;LOC107984664
14	57311001	57312000	LOC105370516
14	57342001	57343000	LOC105370516
14	61386001	61387000	PRKCH
14	62014001	62015000	SYT16
14	63938001	63939000	SYNE2
14	64148001	64149000	SYNE2
14	65633001	65634000	FUT8
14	67341001	67342000	PALS1;ATP6V1D
14	67767001	67768000	ZFYVE26
14	70377001	70378000	SYNJ2BP-COX16;SYNJ2BP
14	70617001	70618000	TTC9-DT;RN7SL77P
14	72195001	72196000	RGS6
14	72670001	72671000	DPF3
14	72680001	72681000	DPF3
14	72984001	72985000	ZFYVE1
14	73128001	73129000	RBM25;PSEN1
14	73130001	73131000	RBM25;PSEN1
14	73248001	73249000	PAPLN;PAPLN-AS1;RNU6-419P
14	74729001	74730000	FCF1

14	75451001	75452000	JDP2
14	75695001	75696000	TTLL5
14	76467001	76468000	ESRRB
14	77993001	77994000	NRXN3
14	81207001	81208000	GTF2A1;SNORA79
14	86079001	86080000	LINC02328
14	88448001	88449000	SPATA7
14	88482001	88483000	PTPN21
14	88495001	88496000	PTPN21
14	89292001	89293000	FOXN3;CAP2P1
14	89325001	89326000	FOXN3
14	91643001	91644000	CATSPERB
14	92171001	92172000	CPSF2
14	92463001	92464000	SLC24A4
14	93076001	93077000	ITPK1;ITPK1-AS1
14	95103001	95104000	DICER1
14	95418001	95419000	SYNE3
14	95502001	95503000	SYNE3
14	96478001	96479000	AK7
14	97052001	97053000	LOC105370647
14	99668001	99669000	HHIPL1
14	99726001	99727000	CYP46A1
14	99939001	99940000	EML1;LOC102724682;RNU1-47P
14	100050001	100051000	EVL
14	100412001	100413000	WDR25;LOC105370664
14	100503001	100504000	WDR25
14	100549001	100550000	BEGAIN
14	101709001	101710000	LINC02320
14	101993001	101994000	DYNC1H1
14	102235001	102236000	MOK
14	102772001	102773000	TRAF3
14	103160001	103161000	LOC105370685
14	104582001	104584000	C14orf180;TMEM179;C14orf180;TMEM179
14	105541001	105542000	LOC105370697;ATP5MC1P1;ELK2BP;LOC105370698
14	105579001	105580000	IGH;IGHA2
14	105768001	105769000	IGH;LOC105378184;IGHG3
15	20073001	20074000	BCAR1P1
15	20153001	20154000	BMS1P15
15	20249001	20250000	RHPN2P1
15	20270001	20271000	RHPN2P1
15	20398001	20399000	HERC2P3
15	20414001	20415000	HERC2P3
15	20430001	20431000	HERC2P3
15	21343001	21344000	LOC112268154;LONRF2P4
15	22223001	22224000	LOC101928039;MIR1268A
15	22278001	22279000	REREP3
15	22504001	22505000	HERC2P2
15	23131001	23132000	LOC101060118;GOLGA6L1;LOC100631267
15	23328001	23329000	LOC100996611;LOC102723623;LOC105376698
15	28651001	28652000	HERC2P9
15	29017001	29018000	APBA2
15	29137001	29138000	FAM189A1
15	29801001	29802000	TJP1
15	30552001	30553000	GOLGA8Q;RN7SL796P
15	32242001	32243000	LOC102724078
15	32489001	32490000	LOC101060588;LOC100653133;LOC107987215
15	33445001	33446000	RYR3
15	34184001	34185000	KATNBL1
15	39589001	39590000	THBS1;FSIP1
15	40141001	40142000	MTND5P37;MTND4P37
15	40188001	40189000	BUB1B;LOC107984763
15	41472001	41473000	RTF1;LOC105370790
15	42715001	42716000	STARD9;CDAN1
15	45215001	45216000	H3P39
15	45886001	45887000	LOC105370802
15	48864001	48865000	SHC4
15	51188001	51189000	MIR4713HG
15	51335001	51336000	CYP19A1;GLDN
15	51998001	51999000	LOC112268149
15	52289001	52290000	MYO5C;LOC105370819
15	53141001	53142000	LOC107983981
15	55983001	55984000	NEDD4
15	58153001	58154000	AQP9;MTCO3P23;MTND3P12;MTND4LP23;MTND5P32;MTND6P23;MTCYBP23
15	61532001	61533000	LOC107984782
15	63068001	63069000	TPM1
15	64419001	64420000	TRIP4
15	64441001	64442000	TRIP4;LOC105370861
15	64747001	64748000	LOC107984737;RBPMS2
15	64863001	64864000	PLEKHO2
15	65193001	65194000	CLPX;CILP
15	65434001	65435000	DPP8
15	66346001	66347000	TIPIN;SCARNA14
15	66659001	66660000	LINC01169
15	67032001	67033000	LOC102723493
15	68331001	68333000	ITGA11;ITGA11
15	69307001	69308000	PAQR5-DT;PAQR5
15	70559001	70560000	LOC107984781

15	72395001	72396000	TMEM202
15	73947001	73948000	LOXL1
15	74037001	74038000	PML
15	74249001	74250000	CCDC33
15	74416001	74417000	SEMA7A;MIR6881
15	74809001	74810000	CSK;LMAN1L
15	75333001	75334000	COMMD4
15	75872001	75873000	UBE2Q2
15	76173001	76174000	TMEM266;LOC101929439
15	77682001	77683000	LINGO1
15	81147001	81148000	CFAP161
15	81851001	81852000	LOC102724001
15	81983001	81984000	LOC105370922
15	82938001	82939000	HOMER2;LOC105370928
15	84812001	84813000	ZNF592;ALPK3
15	85512001	85513000	AKAP13
15	88357001	88358000	LOC105370959
15	88452001	88453000	MRPL46
15	88497001	88498000	DET1
15	88654001	88655000	ISG20
15	89031001	89032000	CARMAL
15	89046001	89047000	CARMAL
15	89581001	89582000	TICRR
15	89829001	89830000	LOC105370965;ARPIN-AP3S2;AP3S2
15	90256001	90257000	CIB1;TTLL13P;NGRN
15	90645001	90646000	CRTC3;CRTC3-AS1;HSPE1P3
15	90888001	90889000	FURIN;FES
15	90917001	90918000	MAN2A2
15	93092001	93093000	RGMA;LOC101927025
15	93266001	93267000	LOC105370982
15	97336001	97337000	LINC02253
15	100234001	100235000	ADAMTS17
15	100334001	100335000	ADAMTS17;SPATA41
15	100711001	100712000	LOC105371024
15	100762001	100763000	LOC105371024
15	101021001	101022000	LRRK1
15	101969001	101970000	MIR1302-10;WASH3P;MIR6859-3;DDX11L9
15	101981001	101982000	WASH3P;MIR6859-3;DDX11L9
16	48001	49000	POLR3K;SNRNP25;RHBDF1
16	148001	149000	NPRL3;HBZ
16	257001	258000	FAM234A
16	607001	608000	RAB40C;LOC101929280
16	682001	683000	RHOT2;RHBDL1;STUB1-DT;STUB1;JMJD8;WDR24;FBXL16
16	1301001	1302000	UBE2I
16	1479001	1480000	CLCN7;RPS3AP2;PTX4
16	1485001	1486000	CLCN7;RPS3AP2;PTX4;TELO2
16	1491001	1492000	PTX4;TELO2
16	1497001	1498000	PTX4;TELO2
16	1767001	1768000	MAPK8IP3;NME3;MRPS34;EME2;SPSB3
16	1898001	1899000	LINC02124
16	1957001	1958000	RPL3L;NDUFB10;RPS2;SNORA10;SNORA64;SNHG9;SNORA78;RNF151
16	1973001	1974000	RPS2;SNORA64;SNHG9;SNORA78;RNF151;TBL3;NOXO1
16	1999001	2000000	SYNGR3;ZNF598
16	2245001	2246000	E4F1;DNASE1L2;ECI1;LOC107984839;RNPS1
16	2247001	2248000	DNASE1L2;ECI1;LOC107984839;RNPS1
16	2434001	2435000	ABCA17P;CCNF
16	2523001	2524000	ATP6V0C;AMDHD2;CEMP1;MIR3178
16	2613001	2614000	PDPK1;LOC652276;PDPK2P
16	2966001	2967000	LOC105371055;KREMEN2;PAQR4;PKMYT1
16	3009001	3010000	LOC101929613;LOC107984895;LOC105371056;CLDN9;CLDN6
16	3398001	3399000	LOC105371059;ZSCAN32;ZNF174
16	3414001	3415000	ZNF174
16	3791001	3792000	CREBBP
16	3806001	3807000	CREBBP
16	4038001	4039000	ADCY9
16	4825001	4826000	GLYR1
16	5225001	5226000	LOC105371067;LINC02164
16	6280001	6281000	RBFOX1
16	7158001	7159000	RBFOX1
16	8617001	8618000	METTL22
16	8777001	8778000	ABAT
16	8780001	8781000	ABAT
16	9526001	9527000	LOC101927026
16	10627001	10628000	TEKT5
16	11125001	11126000	CLEC16A;LOC105371081
16	11173001	11174000	CLEC16A
16	11222001	11223000	LOC107984859
16	11785001	11786000	ZC3H7A
16	12007001	12008000	SNX29
16	12073001	12074000	SNX29;RPS23P6
16	12238001	12239000	SNX29
16	12280001	12281000	SNX29
16	12719001	12720000	CPPED1;MIR4718
16	12756001	12757000	CPPED1;LOC105371090;LOC105371089
16	12979001	12980000	SHISA9
16	14752001	14753000	LOC105376747;NPIPA2;LOC105376749
16	14918001	14919000	LOC101927469;MIR3180-1;PKD1P3-NPIPA1;PKD1P3;LOC100288162;MIR6511A1

16	14921001	14922000	LOC101927469;MIR3180-1;PKD1P3-NPIPA1;PKD1P3;LOC100288162;MIR6511A1;MIR6770-1
16	15292001	15293000	LOC105371097
16	15627001	15628000	MARF1
16	15663001	15664000	NDE1
16	15714001	15715000	NDE1;MYH11
16	16007001	16008000	ABCC1
16	16324001	16325000	PKD1P1;MIR6511A2;MIR6770-2
16	16360001	16361000	PKD1P1;PKD1P2;MIR6511A3
16	16368001	16369000	PKD1P2;MIR6511A3;NPIPA7
16	18330001	18331000	LOC105376753;PKD1P4-NPIPA8;NPIPA8;PKD1P4
16	18346001	18347000	PKD1P4-NPIPA8;PKD1P4;MIR6511A4
16	18349001	18350000	PKD1P4-NPIPA8;PKD1P4;MIR6511A4;NPIPA9;PKD1P5-LOC105376752
16	19602001	19603000	VPS35L
16	19852001	19853000	IQCK;GPRC5B
16	20329001	20330000	GP2;UMOD
16	20721001	20722000	LOC100887080;THUMPD1
16	20758001	20759000	ACSM3
16	21411001	21412000	NPIPB3
16	21853001	21854000	LOC112268174;NPIPB4
16	22544001	22545000	NPIPB5;LOC105371131;OTOAP1
16	24570001	24571000	RBBP6
16	24677001	24678000	TNRC6A;LINC01567
16	24759001	24760000	TNRC6A
16	25904001	25905000	HS3ST4
16	27739001	27740000	KATNIP;LOC105371157
16	28054001	28055000	GSG1L
16	28264001	28265000	SBK1
16	28723001	28724000	EIF3C;MIR6862-2
16	28873001	28874000	SH2B1;ATP2A1;ATP2A1-AS1
16	28903001	28904000	ATP2A1;RABEP2
16	29333001	29334000	SNX29P2
16	29452001	29453000	SMG1P6;BOLA2-SMG1P6;LOC606724;BOLA2;SLX1B;SLX1B-SULT1A4;SULT1A4
16	29575001	29576000	SMG1P2
16	30051001	30052000	TLCD3B;LOC112694756
16	30072001	30073000	LOC112694756;ALDOA;PPP4C
16	30188001	30189000	CORO1A-AS1;CORO1A;BOLA2B;SLX1A;SLX1A-SULT1A3
16	30232001	30233000	LOC101929894;NPIPB13
16	30241001	30242000	LOC101929894;NPIPB13
16	30714001	30715000	SRCAP;SNORA30
16	30723001	30724000	SRCAP
16	30860001	30861000	BCL7C
16	31137001	31138000	KAT8;PRSS8;PRSS36
16	32474001	32475000	ABCD1P3
16	32842001	32843000	IGHV2OR16-5
16	32919001	32920000	IGHV3OR16-6
16	33114001	33115000	HERC2P8
16	33593001	33594000	LOC102724181
16	33611001	33612000	LOC102724181
16	33615001	33616000	LOC102724181
16	33688001	33689000	BMS1P8
16	33920001	33921000	ARHGAP23P1
16	35907001	35908000	PPP1R1AP2
16	46636001	46637000	RAB43P1
16	47839001	47840000	LINC02192
16	48539001	48540000	LOC112268166;N4BP1
16	48605001	48606000	N4BP1;LOC105371239
16	48686001	48687000	LOC105371240
16	49502001	49503000	ZNF423
16	53519001	53520000	LOC105371268
16	53622001	53623000	RPGRIP1L
16	55872001	55873000	CES5A
16	57136001	57137000	CPNE2;LOC105371289
16	57138001	57139000	CPNE2;LOC105371289
16	57414001	57415000	CCL17
16	57493001	57494000	DOK4
16	57580001	57581000	ADGRG5;LOC105371291;LOC107984889
16	57613001	57614000	LOC105371291;HMGB3P32;ADGRG1
16	57904001	57905000	CNGB1
16	58257001	58258000	CCDC113;LOC105371293
16	58610001	58611000	CNOT1
16	65359001	65360000	LINC00922
16	66292001	66293000	RNA5SP428
16	67392001	67393000	LRRC36;TPPP3;RNU1-123P;ZDHHC1
16	67415001	67416000	ZDHHC1
16	67692001	67693000	GFOD2
16	68296001	68297000	SLC7A6;SLC7A6OS
16	69514001	69515000	LOC105371325
16	69599001	69600000	NFAT5
16	69757001	69758000	NOB1;NONOP1;WWP2
16	70016001	70017000	PDXDC2P-NPIPB14P;PDXDC2P
16	70135001	70136000	PDPR
16	70274001	70275000	AARS1;RN7SL279P
16	70665001	70666000	IL34;MTSS2
16	70730001	70731000	VAC14
16	70774001	70775000	VAC14;VAC14-AS1;TRG-GCC3-1;TRG-GCC2-4
16	70791001	70792000	VAC14;TRG-GCC5-1;TRG-GCC2-5
16	71879001	71880000	ZNF821

16	72023001	72024000	DHODH
16	72935001	72936000	ZFHX3
16	74314001	74315000	PSMD7;RPL21P118
16	74497001	74498000	GLG1
16	75120001	75121000	ZNRF1;LDHD;ZFP1
16	75870001	75871000	LOC105371348;RNA5SP430
16	76633001	76634000	LINC02125
16	77713001	77714000	NUDT7
16	78579001	78580000	WWOX
16	78769001	78770000	WWOX
16	80381001	80382000	DYNLRB2-AS1
16	81642001	81643000	CMIP;LOC105371362
16	81711001	81712000	CMIP
16	81785001	81786000	PLCG2
16	81860001	81861000	PLCG2
16	81978001	81979000	SDR42E1
16	83119001	83120000	CDH13
16	83301001	83302000	CDH13
16	84360001	84361000	ATP2C2
16	84634001	84635000	LOC105371376
16	85214001	85215000	GSE1;MIR12128
16	85254001	85255000	GSE1
16	85453001	85454000	GSE1
16	85477001	85479000	GSE1;GSE1
16	85527001	85528000	GSE1
16	85583001	85584000	GSE1
16	85638001	85639000	GSE1
16	85657001	85658000	GSE1;RN7SL381P
16	86291001	86292000	LINC01081;LINC02135
16	86747001	86748000	LINC02188
16	87718001	87719000	KLHDC4
16	87731001	87732000	KLHDC4
16	87934001	87935000	CA5A;LOC107984816
16	88474001	88475000	ZFPM1;MIR5189
16	88828001	88829000	GALNS;LOC107987238
16	88929001	88930000	CBFA2T3;LOC105371409;LOC107984871;LOC100129697
16	89066001	89067000	LOC105371411;LOC105371412
16	89108001	89109000	ACSF3
16	89271001	89272000	ANKRD11
16	89461001	89462000	ANKRD11;LOC101927817;LOC100130664
16	89621001	89622000	DPEP1
16	89646001	89647000	DPEP1;CHMP1A
16	89718001	89719000	VPS9D1;VPS9D1-AS1;ZNF276
16	89824001	89825000	FANCA;SPIRE2;LOC107984817
16	89835001	89836000	SPIRE2;LOC107984817
16	89851001	89852000	SPIRE2;LOC105371419
16	90014001	90015000	DBNDD1;GAS8
16	90041001	90042000	GAS8;URAHP
17	136001	137000	LOC101929823;SCGB1C2;DOC2B
17	246001	247000	RPH3AL
17	811001	812000	NXN
17	824001	825000	NXN
17	1016001	1017000	ABR;LOC112268190;LOC105371481;MIR3183
17	1044001	1045000	ABR
17	1357001	1358000	YWHAE
17	1478001	1479000	MYO1C
17	1669001	1670000	PRPF8
17	1704001	1705000	TLCD2;MIR22HG;MIR22
17	1730001	1731000	WDR81
17	1745001	1746000	WDR81;SERPINF2
17	1846001	1847000	RPA1
17	2188001	2189000	SMG6
17	2807001	2808000	RAP1GAP2
17	3177001	3178000	LOC100288728
17	3580001	3581000	TRPV1
17	3595001	3596000	TRPV1
17	3785001	3786000	ITGAE
17	3966001	3967000	ATP2A3
17	4094001	4095000	ZZEF1
17	4148001	4149000	ZZEF1;CYB5D2
17	4176001	4177000	ANKFY1
17	4332001	4333000	UBE2G1
17	4468001	4469000	SPNS3
17	4633001	4634000	LOC105371498;ALOX15
17	5071001	5072000	LOC100421332;ZFP3
17	5244001	5245000	ZNF594-DT;SCIMP
17	5545001	5546000	NLRP1;LOC105371507
17	5726001	5727000	LOC100128284
17	6317001	6318000	BTF3P14
17	6583001	6584000	KIAA0753
17	6667001	6668000	ALOX15P1
17	6987001	6988000	ALOX12-AS1;ALOX12
17	7408001	7409000	TMEM256-PLSCR3;TMEM256;NLGN2
17	7429001	7430000	NLGN2;SPEM1;SPEM2;SPEM3;TMEM102;FGF11
17	7465001	7466000	CHRNB1;ZBTB4
17	7499001	7500000	POLR2A
17	7555001	7556000	TNFSF12-TNFSF13;TNFSF12;TNFSF13;SENP3;SENP3-EIF4A1

17	7593001	7594000	LOC100996842;MPDU1;SOX15;FXR2
17	8697001	8698000	LOC105371525
17	8866001	8867000	PIK3R6;LOC107985071
17	8947001	8948000	PIK3R5
17	9054001	9055000	NTN1
17	9275001	9276000	STX8
17	9321001	9322000	STX8
17	9329001	9330000	STX8
17	9394001	9395000	STX8
17	9528001	9529000	STX8
17	9715001	9716000	USP43
17	9897001	9898000	GLP2R;RCVRN
17	10652001	10653000	MYH3
17	11799001	11800000	DNAH9
17	11924001	11925000	DNAH9
17	12589001	12590000	LINC00670;LOC105371540
17	14046001	14047000	COX10-AS1
17	15254001	15255000	PMP22;MIR4731
17	15338001	15339000	TEKT3
17	15727001	15728000	ZNF286A-TBC1D26;ZNF286A;TBC1D26;TBC1D26-AS1
17	16145001	16146000	NCOR1;RNU6-862P;SNORD163
17	17229001	17230000	FLCN
17	17394001	17395000	RPL13P12
17	17502001	17503000	MED9;RASD1;PEMT
17	17527001	17528000	PEMT
17	17669001	17670000	SMCR2
17	18202001	18203000	ALKBH5
17	19088001	19089000	KYNUP2;LOC102724624
17	19214001	19215000	KYNUP3;LOC388436
17	19224001	19225000	LOC388436
17	19382001	19383000	B9D1;MAPK7;MFAP4
17	19535001	19536000	SLC47A1
17	19582001	19583000	SLC47A1;SLC47A1P1
17	19587001	19588000	SLC47A1;SLC47A1P1
17	20950001	20951000	LOC339260;SPECC1P2
17	21425001	21426000	KCNJ12
17	22527001	22528000	MTND6P35;MTCYBP13;MTRNR2L1;MTND1P15;MTND2P13;MTCO1P13;NMTRS-TGA3-1;MTCO2P13;MTATP6P3;MTCO3P13;MTND4LP8;MTND6P34
17	27936001	27937000	LINC01992
17	30294001	30295000	BLMH;RNU6-1267P
17	30583001	30584000	LOC101927093;SMURF2P1-LRRC37BP1
17	30594001	30595000	SMURF2P1-LRRC37BP1;SMURF2P1
17	31177001	31178000	NF1
17	31377001	31378000	NF1
17	31472001	31473000	RAB11FIP4;RN7SL79P
17	31762001	31763000	GPR160P2
17	32285001	32286000	RHBDL3;LOC105371732
17	32438001	32439000	NUDT15P2;PSMD11
17	32888001	32889000	LOC102724715
17	32992001	32993000	SPACA3
17	34033001	34034000	ASIC2;LOC107987247;TLK2P1
17	34944001	34945000	CCT6B
17	35735001	35736000	AP2B1;RASL10B;GAS2L2
17	36327001	36328000	LOC101060212;TBC1D3G
17	36618001	36619000	MRM1
17	37057001	37058000	AATF;LOC105371753
17	37334001	37335000	ACACA
17	37565001	37566000	SYNRG
17	39752001	39753000	GRB7;IKZF3
17	40040001	40041000	MED24
17	40213001	40214000	WIPF2
17	41880001	41881000	ACLY
17	42047001	42048000	ZNF385C;C17orf113
17	42167001	42168000	RAB5C-AS1;KCNH4
17	42576001	42577000	COASY;MLX;PSMC3IP;RETREG3
17	42652001	42653000	ATP5MGP7;HMGB3P27;TUBG2
17	44058001	44059000	LSM12
17	44980001	44981000	LOC112268183;LOC107987243
17	45255001	45256000	FMNL1;MAP3K14-AS1;SPATA32;MAP3K14
17	45590001	45591000	DND1P1;LOC105376839;MAPK8IP1P2
17	47193001	47194000	CDC27
17	47487001	47489000	MRPL45P2;MRPL45P2
17	49010001	49011000	IGF2BP1
17	50266001	50267000	LOC105371819;LOC105371822;TMEM92
17	56985001	56986000	LOC105371836;SCPEP1
17	60116001	60117000	LOC100996660
17	60682001	60683000	BCAS3
17	61601001	61602000	NACA2
17	62224001	62225000	LOC100996361
17	62803001	62804000	MARCHF10;MARCHF10-DT
17	63704001	63705000	MAP3K3;LIMD2;LOC729683;STRADA
17	64050001	64051000	ERN1
17	66121001	66122000	CEP112
17	66601001	66602000	PRKCA
17	68018001	68019000	TRR-CCG2-1
17	69447001	69448000	MAP2K6
17	71021001	71022000	LOC100131241

17	72484001	72485000	LINC00673
17	73366001	73367000	SDK2
17	73504001	73505000	SDK2;LOC101928251
17	73605001	73606000	SDK2
17	74530001	74531000	CD300LB;LOC107985074;CD300C
17	74592001	74593000	CD300LD;CD300LD-AS1;LOC101928343
17	75441001	75442000	TMEM94
17	76851001	76852000	LINC00868
17	77276001	77277000	LACAT1;SEPTIN9-DT;SEPTIN9
17	77541001	77542000	LOC400622;LOC100507351
17	78038001	78039000	TNRC6C
17	78502001	78503000	DNAH17;DNAH17-AS1
17	78627001	78628000	SCAT1
17	78692001	78693000	CYTH1;RNU6-638P
17	78796001	78797000	USP36
17	78863001	78864000	TIMP2
17	78870001	78871000	TIMP2
17	78888001	78889000	TIMP2;CEP295NL
17	78898001	78899000	TIMP2;CEP295NL
17	79121001	79123000	RBFOX3;RBFOX3
17	79329001	79330000	RBFOX3
17	79422001	79423000	RBFOX3
17	79554001	79555000	RBFOX3
17	79730001	79731000	ENPP7
17	79941001	79942000	TBC1D16
17	80021001	80022000	TBC1D16
17	80064001	80065000	CCDC40
17	80096001	80097000	CCDC40;MIR1268B;GAA
17	80254001	80255000	SLC26A11;RNF213
17	80289001	80290000	RNF213
17	80742001	80743000	RPTOR
17	80845001	80846000	RPTOR
17	80878001	80879000	RPTOR
17	80882001	80883000	RPTOR
17	81062001	81063000	BAIAP2
17	81110001	81111000	BAIAP2;LOC107987250;AATK
17	81212001	81213000	CEP131
17	81262001	81263000	SLC38A10
17	81627001	81628000	NPLOC4;TSPAN10
17	81946001	81947000	PYCR1;MYADML2;NOTUM
17	82172001	82173000	CCDC57
17	82313001	82314000	LOC107985059;CD7;SECTM1
17	82358001	82359000	TEX19
17	82641001	82642000	WDR45B
17	82846001	82847000	TBCD;ZNF750
17	83018001	83019000	B3GNTL1
17	83223001	83224000	LOC101929650;LOC100505909;RPL23AP87
18	10001	11000	LINC02564
18	3286001	3287000	MYL12B
18	3659001	3660000	DLGAP1;LOC105371967;LOC107985137
18	3696001	3697000	DLGAP1
18	3741001	3742000	DLGAP1
18	3793001	3794000	DLGAP1
18	4476001	4477000	ELOCP27
18	6612001	6613000	LOC107985176
18	8422001	8423000	COP1P1
18	10334001	10335000	LOC105371986
18	10644001	10645000	PMM2P1;LOC107987262
18	11029001	11030000	PIEZO2
18	11635001	11636000	NPIPB1P
18	11996001	11997000	IMPA2
18	12612001	12613000	SPIRE1
18	13126001	13127000	CEP192
18	14448001	14449000	LONRF2P1
18	21348001	21349000	GREB1L
18	23383001	23384000	TMEM241
18	23577001	23578000	NPC1
18	23661001	23662000	ANKRD29
18	26969001	26970000	CHST9
18	33731001	33732000	ASXL3
18	34870001	34871000	DTNA
18	35668001	35669000	GALNT1;LOC105372065
18	39568001	39569000	MIR924HG
18	42555001	42556000	LINC00907
18	46131001	46132000	HAUS1;RNU6-1278P
18	46931001	46932000	KATNAL2
18	46946001	46947000	KATNAL2
18	47021001	47022000	KATNAL2;ELOA3BP;ELOA3P
18	47027001	47028000	KATNAL2;ELOA3BP;ELOA3P;ELOA2
18	48860001	48861000	CTIF
18	49217001	49218000	DYM
18	50106001	50107000	MYO5B
18	50830001	50831000	MRO;RPL17P46
18	52792001	52793000	DCC
18	58194001	58195000	NEDD4L;LOC105372143
18	58689001	58690000	MALT1;LOC105372146;MRPL37P1
18	58909001	58910000	ZNF532

18	59038001	59039000	OACYLP
18	59237001	59238000	GRP
18	61514001	61515000	CDH20
18	62011001	62012000	LOC105372158
18	64249001	64250000	LINC01924;LINC01538
18	66545001	66546000	CDH19
18	70651001	70652000	GTSCR1
18	73250001	73251000	LINC02864
18	74161001	74162000	TIMM21
18	74955001	74956000	ZNF407
18	76130001	76131000	LOC339298
18	76406001	76407000	ZNF516
18	76413001	76414000	ZNF516
18	76532001	76533000	LINC00908
18	77252001	77254000	GALR1;GALR1
18	77407001	77408000	LOC107985171
18	77865001	77866000	RNA5SP461
18	77990001	77991000	LINC01029;LOC105372218
18	78565001	78566000	LOC105372220
18	79200001	79201000	ATP9B
18	79346001	79347000	ATP9B;LOC107985149
18	79439001	79440000	NFATC1
18	79456001	79457000	NFATC1;LOC101927897
18	79634001	79635000	LOC284241
18	79660001	79661000	LOC284241
18	79697001	79698000	CTDP1;LOC105376875
18	79715001	79716000	CTDP1
18	79731001	79732000	CTDP1
18	79835001	79836000	KCNG2
18	79902001	79903000	KCNG2;SLC66A2
18	80045001	80046000	RBFA
19	61001	62000	WASH5P;LOC105376912;MIR1302-11
19	337001	338000	MIER2
19	487001	488000	ODF3L2;MADCAM1
19	502001	503000	MADCAM1;MADCAM1-AS1;TPGS1
19	543001	544000	CDC34;GZMM
19	592001	593000	BSG;HCN2;LOC100420586
19	608001	609000	HCN2;LOC107987266;POLRMT
19	614001	615000	HCN2;LOC107987266;POLRMT
19	624001	625000	HCN2;POLRMT;LOC105372234
19	674001	675000	FSTL3
19	739001	740000	PALM;MISP
19	956001	957000	ARID3A
19	974001	975000	ARID3A;WDR18
19	1255001	1256000	LOC102723811;MIDN;LOC107985303;LOC105372236
19	1643001	1644000	TCF3
19	1820001	1821000	ATP8B3;REXO1;MIR1909;LOC100288123
19	2012001	2013000	BTBD2;LOC107985278
19	2177001	2178000	DOT1L
19	2209001	2210000	DOT1L
19	2395001	2396000	TMPRSS9
19	2401001	2402000	TMPRSS9
19	2542001	2543000	GNG7;ELOCP28
19	2568001	2569000	GNG7
19	2649001	2650000	GNG7
19	2735001	2736000	SLC39A3
19	2759001	2760000	SGTA
19	2762001	2763000	SGTA
19	2806001	2807000	THOP1
19	3039001	3040000	TLE2
19	3077001	3078000	LOC105372242
19	3236001	3237000	CELF5
19	3279001	3280000	CELF5
19	3362001	3363000	NFIC
19	3611001	3612000	TBXA2R;CACTIN-AS1;CACTIN
19	3624001	3625000	CACTIN;PIP5K1C
19	3660001	3661000	PIP5K1C
19	3664001	3665000	PIP5K1C
19	3771001	3772000	APBA3;LOC105372248;MRPL54;RAX2;MATK
19	3838001	3839000	ZFR2
19	3952001	3953000	NMRK2;DAPK3;MIR637
19	4012001	4013000	PIAS4
19	4048001	4049000	PIAS4;ZBTB7A
19	4950001	4951000	UHRF1
19	5014001	5015000	KDM4B
19	5097001	5098000	KDM4B
19	5146001	5147000	KDM4B
19	5208001	5209000	PTPRS
19	5299001	5300000	PTPRS;LOC105372253
19	5553001	5554000	TINCR
19	5563001	5564000	TINCR
19	5731001	5732000	CATSPERD
19	5852001	5853000	FUT3;LOC101928844
19	5854001	5855000	FUT3;LOC101928844
19	5944001	5945000	RANBP3
19	6716001	6717000	C3
19	7017001	7018000	LOC112268245;MBD3L2B

19	7279001	7280000	INSR
19	7555001	7556000	PNPLA6
19	7851001	7852000	EVI5L
19	7908001	7909000	LRRC8E;MAP2K7;TGFBR3L
19	7996001	7997000	ELAVL1
19	8123001	8124000	FBN3
19	8428001	8429000	MARCHF2
19	8466001	8467000	HNRNPM
19	8543001	8544000	MYO1F
19	8839001	8840000	ZNF558;MBD3L1;MUC16
19	8890001	8891000	MUC16
19	9899001	9900000	OLFM2
19	9965001	9966000	COL5A3
19	10016001	10017000	COL5A3;RDH8
19	10141001	10142000	DNMT1
19	10148001	10149000	DNMT1
19	10297001	10298000	ICAM4;ICAM5;ZGLP1
19	10329001	10330000	RAVER1;ICAM3
19	10594001	10595000	AP1M2;LOC100820734;SLC44A2
19	11159001	11160000	SPC24;KANK2
19	11164001	11165000	SPC24;KANK2
19	11187001	11188000	KANK2
19	11445001	11446000	ODAD3;PRKCSH;ELAVL3
19	11584001	11585000	ACP5;ZNF627;GAPDHP76
19	11691001	11692000	ZNF833P
19	11895001	11896000	ZNF69
19	12539001	12540000	ZNF564
19	12641001	12642000	ZNF791;LOC105372279;RPL10P16;MAN2B1
19	12741001	12742000	TRIR;GET3;BEST2
19	12785001	12786000	HOOK2;MIR5684;JUNB
19	12992001	12993000	NFIX
19	12998001	12999000	NFIX
19	13122001	13123000	TRMT1;NACC1
19	13184001	13185000	LOC107985288
19	13254001	13255000	CACNA1A
19	13286001	13287000	CACNA1A
19	13776001	13777000	MRI1;C19orf53
19	14430001	14432000	PKN1;PKN1
19	14584001	14585000	CLEC17A;RN7SL337P;RN7SL842P
19	15087001	15088000	OR1I1
19	15829001	15830000	ZNF861P;LINC01764;UCA1
19	15887001	15888000	CYP4F2
19	16873001	16874000	SIN3B
19	17181001	17182000	MYO9B
19	17593001	17594000	COLGALT1;RPL21P130;UNC13A
19	17623001	17624000	UNC13A
19	18005001	18006000	KCNN1;ARRDC2
19	18009001	18010000	KCNN1;ARRDC2
19	18223001	18224000	PDE4C
19	18346001	18347000	PGPEP1
19	18537001	18538000	FKBP8
19	18585001	18586000	UBA52;REX1BD;CRLF1
19	18771001	18772000	CRTC1
19	18880001	18881000	CERS1;GDF1
19	19150001	19151000	BORCS8-MEF2B;MEF2B
19	19498001	19499000	GATAD2A
19	19575001	19576000	PBX4
19	20524001	20525000	LOC105372316;ZNF737
19	21535001	21536000	ZNF429
19	21884001	21885000	LOC105372324;LOC105372326;LOC112268249
19	22542001	22543000	LOC105376917;LINC01233
19	24047001	24048000	ZNF254
19	30084001	30085000	LOC107985271
19	32614001	32615000	ANKRD27;SNORA68B
19	32999001	33000000	RHPN2
19	33428001	33429000	PEPD
19	35020001	35021000	GRAMD1A;SCN1B
19	35140001	35141000	LGI4;FXYD1;FXYD7
19	35308001	35309000	MAG
19	35558001	35559000	ATP4A
19	35596001	35597000	LINC01766
19	35738001	35739000	KMT2B;IGFLR1;U2AF1L4;PSENEN;LIN37
19	35766001	35767000	HSPB6;PROSER3;ARHGAP33
19	35792001	35793000	ARHGAP33;LINC01529;PRODH2
19	36097001	36098000	WDR62
19	36109001	36110000	WDR62;OVOL3;POLR2I;TBCB
19	36440001	36441000	ZNF566
19	36688001	36689000	ZNF567;LINC01534
19	37265001	37266000	LINC01535;LOC284412
19	37457001	37458000	ZNF569;ZNF570
19	37684001	37685000	ZNF781;SELENOKP1
19	38078001	38079000	SIPA1L3
19	38223001	38224000	DPF1
19	38440001	38441000	RYR1
19	38460001	38461000	RYR1
19	38478001	38479000	RYR1
19	38804001	38805000	RNU6-140P;LGALS4

19	38943001	38944000	MRPS12;FBXO17
19	39337001	39338000	GMFG;SAMD4B
19	39395001	39396000	SAMD4B;PAF1;MED29
19	39901001	39902000	FCGBP
19	40350001	40351000	C19orf47;PLD3
19	40531001	40532000	SPTBN4
19	40607001	40608000	LTBP4
19	40685001	40686000	NUMBL;COQ8B
19	41217001	41218000	CYP2S1;AXL
19	41230001	41231000	AXL
19	41377001	41378000	TMEM91;EXOSC5
19	41392001	41393000	TMEM91;EXOSC5;BCKDHA
19	41917001	41918000	ARHGEF1;ERFL
19	42017001	42018000	GRIK5
19	42170001	42171000	POU2F2
19	43357001	43358000	PRG1;CD177
19	43502001	43503000	PHLDB3;ETHE1
19	43624001	43625000	ZNF428;SRRM5;LOC105372411;CADM4
19	43730001	43731000	IRGC;SMG9
19	44454001	44455000	ZNF229;ZNF285B
19	44696001	44697000	CEACAM16-AS1;LOC107985306;CEACAM16
19	45316001	45317000	CKM
19	45927001	45928000	NOVA2
19	46001001	46002000	CCDC61
19	46312001	46313000	HIF3A;RNU6-924P
19	46732001	46733000	STRN4
19	46847001	46848000	AP2S1
19	46940001	46941000	ARHGAP35
19	47008001	47009000	ARHGAP35
19	47261001	47262000	CCDC9
19	47283001	47284000	INAFM1;LOC105372427
19	47397001	47398000	MEIS3
19	47440001	47441000	SLC8A2
19	47475001	47476000	SLC8A2;KPTN;NAPA-AS1
19	47585001	47586000	RN7SL322P
19	47625001	47626000	BICRA
19	47651001	47652000	BICRA
19	47873001	47874000	LINC01595;SULT2A1
19	47905001	47906000	SNAR-A12;SNAR-C5
19	47957001	47958000	SNAR-C3;SNAR-C1;BSPH1
19	47975001	47976000	BSPH1
19	48241001	48242000	CARD8;VN1R97P
19	48611001	48612000	FAM83E;SPACA4;RPL18;SPHK2
19	48650001	48651000	CA11;SEC1P
19	48701001	48702000	FUT2;LOC105447645
19	48743001	48744000	RASIP1;IZUMO1;FUT1
19	48989001	48990000	GYS1;RUVBL2
19	49100001	49101000	SNRNP70
19	49183001	49184000	TRPM4
19	49233001	49234000	LOC107985340
19	49335001	49336000	SLC6A16;CD37;TEAD2
19	49479001	49480000	ALDH16A1;FLT3LG;RPL13A;SNORD32A
19	49547001	49549000	RCN3;NOSIP;RCN3;NOSIP
19	49636001	49637000	PRR12;RRAS;SCAF1
19	50094001	50095000	SNAR-A4;SNAR-A14;SNAR-A5;SNAR-A6
19	50117001	50118000	SNAR-A7;SNAR-A8;SNAR-A9;SNAR-A10
19	50139001	50140000	SNAR-B1;LOC105372436;SNAR-B2;SNAR-D
19	50206001	50207000	MYH14
19	50246001	50247000	MYH14
19	50260001	50261000	MYH14
19	50517001	50518000	JOSD2;ASPDH;LRRC4B
19	50601001	50602000	SNAR-F
19	50767001	50768000	GPR32P1;GPR32
19	50957001	50958000	KLK5;KLK6;LOC105372442
19	51847001	51848000	ZNF577;VN1R98P
19	51857001	51858000	ZNF577;VN1R98P;LOC100419706
19	51989001	51990000	ZNF350-AS1;ZNF350;ZNF615
19	53270001	53271000	VN1R4
19	53582001	53583000	ZNF331
19	53775001	53776000	RNU6-165P;HMGN1P32;SEPTIN7P8;RPLP1P12
19	54015001	54016000	CACNG6
19	54143001	54144000	CNOT3;LOC102724273
19	54187001	54188000	MBOAT7;TSEN34
19	54226001	54227000	LILRB3;LOC100421130;LOC107985279;LILRA6
19	54476001	54477000	CDC42EP5
19	54625001	54626000	LILRB1
19	55285001	55286000	HSPBP1;BRSK1
19	55336001	55337000	TMEM150B;KMT5C
19	55345001	55346000	KMT5C;COX6B2;FAM71E2
19	55393001	55394000	TMEM238;RPL28;MIR6805;UBE2S;SNORD157
19	55480001	55481000	ZNF628;NAT14;SSC5D
19	55604001	55605000	FIZ1;ZNF524;ZNF865;LOC107985322
19	55636001	55637000	LOC107983998;ZNF581;ZNF580
19	55934001	55935000	NLRP13
19	56114001	56115000	ZNF787
19	56283001	56284000	ZSCAN5A;EDDM13;LOC105372470
19	56619001	56620000	ZNF71-SMIM17;ZNF71

19	56734001	56735000	LOC105372472
19	57166001	57167000	DUXA;LOC100132009
19	57816001	57817000	ZNF552;FKBP1AP1
19	58021001	58022000	VN2R19P
19	58113001	58114000	ZSCAN18
19	58267001	58268000	ZNF544;ZNF8-DT
19	58326001	58327000	LOC105372480;LOC100887072;ZSCAN22;MIR6806
19	58369001	58370000	ZNF497;LOC100419840;ZNF497-AS1;RNA5SP473;ZNF837;LOC105372484
20	897001	898000	ANGPT4
20	901001	902000	ANGPT4
20	1278001	1279000	SNPH
20	1892001	1893000	LOC107984088;LOC102724545;SIRPA
20	3310001	3311000	DNAAF9
20	3497001	3498000	ATRN;SF3A3P1
20	3628001	3629000	ATRN
20	3858001	3859000	MAVS
20	16387001	16388000	KIF16B
20	16439001	16440000	KIF16B
20	17905001	17906000	RNU6-192P;LOC105372549
20	18040001	18041000	OVOL2
20	22662001	22663000	LINC01747
20	23189001	23190000	LOC100505664
20	24448001	24449000	GAPDHP53
20	25331001	25332000	ABHD12
20	25453001	25454000	GINS1;NINL
20	25800001	25801000	FAM182B
20	29409001	29410000	LOC105379476;DUX4L34;FRG2EP
20	29470001	29471000	FRG1EP
20	29499001	29500000	FRG1EP
20	29882001	29883000	RNA5SP532;DUX4L37
20	30514001	30515000	LOC110467526;LOC105379481
20	30632001	30633000	LOC112268265
20	30676001	30677000	ANKRD20A21P
20	31469001	31470000	DEFB124;REM1
20	31536001	31537000	HM13
20	31666001	31667000	BCL2L1
20	32574001	32575000	NOL4L
20	32774001	32775000	DNMT3B
20	33912001	33913000	TPM3P2
20	33999001	34000000	RALY-AS1;RALY
20	34092001	34093000	RALY;EIF2S2
20	34290001	34291000	AHCY
20	34561001	34562000	MAP1LC3A;PIGU
20	35287001	35288000	MMP24-AS1-EDEM2;MMP24OS;EIF6;FAM83C-AS1;FAM83C
20	36229001	36230000	EPB41L1;LOC105372602;AAR2
20	36421001	36422000	DLGAP4
20	36426001	36427000	DLGAP4
20	36441001	36442000	DLGAP4
20	37017001	37018000	RBL1;RPS3AP3
20	37334001	37335000	LOC105372606;SRC
20	37414001	37415000	SRC
20	39801001	39802000	LOC105372614
20	42529001	42530000	PTPRT
20	43625001	43626000	IFT52
20	43712001	43713000	MYBL2
20	44188001	44189000	JPH2;OSER1
20	44220001	44221000	OSER1;OSER1-DT
20	44939001	44941000	PABPC1L;TOMM34;PABPC1L;TOMM34
20	45405001	45406000	SYS1-DBNDD2;LOC107985404;DBNDD2;LOC107985405
20	46116001	46117000	CD40
20	46176001	46177000	CDH22
20	46186001	46187000	CDH22
20	46547001	46548000	OCSTAMP;SLC13A3
20	46595001	46596000	SLC13A3
20	46679001	46680000	SLC13A3;TP53RK
20	47666001	47667000	NCOA3;SULF2
20	47969001	47970000	LOC105372637;LOC112268266
20	48018001	48019000	LOC107985436;LOC107985437;LOC105372639
20	48502001	48503000	RNU7-144P;LOC105372647
20	48510001	48511000	LOC105372647
20	48551001	48552000	LOC105372646
20	48748001	48749000	PREX1
20	48786001	48787000	PREX1
20	49408001	49409000	LOC105372649;KCNB1
20	50371001	50372000	LOC105372657
20	50835001	50836000	BCAS4;TMSB4XP6
20	51002001	51003000	KCNG1
20	51406001	51407000	LOC105372663;NFATC2
20	51486001	51487000	NFATC2
20	51525001	51526000	NFATC2
20	51630001	51631000	ATP9A
20	51761001	51762000	ATP9A
20	53605001	53606000	ZNF217;LOC105372672
20	53876001	53877000	SUMO1P1
20	57316001	57317000	LOC105372687;MIR4325
20	57378001	57379000	RAE1;RBM38-AS1
20	58535001	58536000	APCDD1L-DT

20	58575001	58576000	APCDD1L-DT
20	58581001	58582000	APCDD1L-DT
20	58999001	59000000	NELFCD;CTSZ
20	59127001	59128000	ZNF831
20	59723001	59724000	PHACTR3
20	61523001	61524000	CDH4
20	61990001	61991000	TAF4;LOC105372704
20	62205001	62206000	MTG2;HRH3
20	62279001	62280000	OSBPL2
20	62335001	62336000	LAMA5;MIR4758
20	62349001	62350000	LAMA5;LAMA5-AS1
20	62833001	62834000	COL9A3;TCFL5
20	63019001	63020000	LINC01749;LOC105372718
20	63671001	63672000	RTEL1-TNFRSF6B;RTEL1
20	63785001	63786000	ZBTB46
20	64012001	64013000	PRPF6
20	64053001	64054000	TCEA2;SOX18
21	6632001	6633000	LOC102724701
21	8202001	8205000	MIR6724-1;RNA45SN2;MIR3648-1;RNA18SN2;RNA5-8SN2;MIR6724-1;RNA45SN2;MIR3648-1;RNA18SN2;RNA5-8SN2;RNA28SN2;MIR6724-1;RNA45SN2;MIR3648-1;RNA18SN2;RNA5-8SN2;RNA28SN2
21	8219001	8249000	RNA45SN2;RNA18SN2;RNA5-8SN2;RNA28SN2;LOC105379506;LOC107987293;RNA45SN2;RNA18SN2;RNA5-8SN2;RNA28SN2;LOC105379506;LOC107987293;RNA45SN2;RNA18SN2;RNA5-8SN2;RNA28SN2;LOC105379506;LOC107987293;RNA45SN2;RNA5-8SN2;RNA28SN2;LOC105379506;LOC107987293;RNA45SN2;RNA28SN2;LOC105379506;LOC107987293;RNA45SN2;RNA28SN2;LOC105379506;LOC107987293;RNA45SN2;RNA28SN2;LOC105379506;LOC107987293;RNA45SN2;RNA28SN2;LOC105379506;LOC107987293;RNA45SN2;RNA28SN2;LOC105379506;LOC107987293;RNA45SN2;RNA28SN2;LOC105379506;LOC107987293;RNA45SN2;LOC105379506;LOC107987293;LOC105379506;LOC107987293;CDC27P9;LOC105379506;LOC107987293;CDC27P9;LOC105379506;LOC107987293;CDC27P9;LOC105379506;LOC107987293;CDC27P9;LOC107987293;CDC27P9;LOC107987293;CDC27P9;LOC107987293;CDC27P9;LOC107987293;CDC27P9;CDC27P9;CDC27P9;MIR6724-2;CDC27P9;MIR6724-2;CDC27P9;MIR6724-2;CDC27P9;MIR6724-2;CDC27P9;MIR6724-2;RNA18SP5;CDC27P9;MIR6724-2;RNA18SP5;CDC27P9;MIR6724-2;RNA18SP5;CDC27P9;MIR6724-2;RNA18SP5;RNA5-8SP10;CDC27P9;MIR6724-2;RNA18SP5;RNA5-8SP10;CDC27P9;MIR6724-2;RNA18SP5;RNA5-8SP10;LOC110467529
21	8385001	8388000	MIR6724-3;RNA45SN3;RNA18SN3;RNA5-8SN3;MIR6724-3;RNA45SN3;RNA18SN3;RNA5-8SN3;RNA28SN3;MIR6724-3;RNA45SN3;RNA18SN3;RNA5-8SN3;RNA28SN3
21	8403001	8418000	RNA45SN3;RNA18SN3;RNA5-8SN3;RNA28SN3;LOC105379508;RNA45SN3;RNA18SN3;RNA5-8SN3;RNA28SN3;LOC105379508;RNA45SN3;RNA5-8SN3;RNA28SN3;LOC105379508;RNA45SN3;RNA28SN3;LOC105379508;RNA45SN3;RNA28SN3;LOC105379508;RNA45SN3;RNA28SN3;LOC105379508;RNA45SN3;RNA28SN3;LOC105379508;RNA45SN3;RNA28SN3;LOC105379508;RNA45SN3;RNA28SN3;LOC105379508;RNA45SN3;LOC105379508;LOC105379508;CDC27P10;LOC105379508;CDC27P10;LOC105379508;CDC27P10;LOC105379508;CDC27P10;CDC27P10
21	8419001	8432000	CDC27P10;CDC27P10;CDC27P10;CDC27P10;MIR6724-4;CDC27P10;MIR6724-4;RNA45SN1;CDC27P10;MIR6724-4;RNA45SN1;CDC27P10;MIR6724-4;RNA45SN1;CDC27P10;MIR6724-4;RNA45SN1;MIR10396B;RNA18SN1;CDC27P10;MIR6724-4;RNA45SN1;MIR10396B;RNA18SN1;CDC27P10;MIR6724-4;RNA45SN1;MIR10396B;RNA18SN1;CDC27P10;MIR6724-4;RNA45SN1;MIR10396B;RNA18SN1;RNA5-8SN1;CDC27P10;MIR6724-4;RNA45SN1;MIR10396B;RNA18SN1;RNA5-8SN1;CDC27P10;MIR6724-4;RNA45SN1;MIR10396B;RNA18SN1;RNA5-8SN1;RNA28SN1
21	8447001	8463000	RNA45SN1;RNA18SN1;RNA5-8SN1;RNA28SN1;LOC105379507;LOC107987294;RNA45SN1;RNA18SN1;RNA5-8SN1;RNA28SN1;LOC105379507;LOC107987294;RNA45SN1;RNA5-8SN1;RNA28SN1;LOC105379507;LOC107987294;RNA45SN1;RNA28SN1;LOC105379507;LOC107987294;RNA45SN1;RNA28SN1;LOC105379507;LOC107987294;RNA45SN1;RNA28SN1;LOC105379507;LOC107987294;RNA45SN1;RNA28SN1;LOC105379507;LOC107987294;RNA45SN1;RNA28SN1;LOC105379507;LOC107987294;RNA45SN1;RNA28SN1;LOC105379507;LOC107987294;RNA45SN1;RNA28SN1;LOC105379507;LOC107987294;CDC27P11;LOC105379507;LOC107987294;CDC27P11;LOC105379507;LOC107987294;CDC27P11;LOC105379507;LOC107987294;CDC27P11;LOC105379507;LOC107987294;CDC27P11;LOC107987294;CDC27P11;LOC107987294;CDC27P11
21	8464001	8473000	LOC107987294;CDC27P11;CDC27P11;CDC27P11;CDC27P11;CDC27P11;CDC27P11;CDC27P11;CDC27P11;CDC27P11
21	8785001	8786000	LINC01666
21	8806001	8812000	LINC01666;SNX18P10;LINC01666;SNX18P10;LINC01666;SNX18P10;LINC01666;SNX18P10;LOC107985475;LINC01666;SNX18P10;LOC107985475;LINC01666;SNX18P10;LOC107985475
21	8816001	8817000	LOC107985475
21	8882001	8883000	ANKRD20A16P
21	8885001	8886000	ANKRD20A16P
21	8989001	8990000	MIR3648-2;MIR10396A
21	9133001	9134000	TEKT4P2
21	9339001	9340000	LOC101930100
21	9541001	9542000	NCOR1P4
21	9546001	9547000	NCOR1P4
21	9816001	9817000	LINC01667
21	10354001	10355000	VN1R7P
21	10363001	10364000	VN1R7P
21	10432001	10433000	BAGE2
21	10439001	10440000	BAGE2
21	10452001	10453000	BAGE2
21	10518001	10519000	BAGE2;TPTE
21	10655001	10657000	IGHV1OR21-1;IGHV1OR21-1
21	16613001	16614000	MIR99AHG
21	18069001	18070000	CHODL
21	25880001	25881000	APP
21	26927001	26928000	ADAMTS5
21	28163001	28164000	LINC01695
21	31565001	31566000	TIAM1;LOC150051
21	32261001	32262000	MIS18A
21	33398001	33399000	IFNGR2
21	34719001	34720000	CLIC6;LINC00160;LOC107985515
21	34843001	34844000	RUNX1;LOC102724584
21	35405001	35406000	LOC100506403
21	36033001	36034000	SETD4
21	36276001	36277000	DOP1B
21	36661001	36662000	LOC105369308

21	37719001	37720000	KCNJ6;LOC107985507;KCNJ6-AS1
21	39031001	39032000	LOC102724740;LOC107985484
21	39442001	39443000	GET1-SH3BGR;LCA5L;SH3BGR;MIR6508
21	40616001	40617000	DSCAM;DSCAM-IT1
21	41722001	41723000	LINC00479;LINC00112
21	42106001	42107000	UMODL1;UMODL1-AS1
21	42222001	42223000	ABCG1;LOC105372814;RNA5SP492
21	42410001	42411000	UBASH3A;RNU6-1149P
21	42480001	42481000	RSPH1
21	42920001	42921000	NDUFV3;ERVH48-1
21	43028001	43029000	PKNOX1
21	43679001	43680000	RRP1B
21	44089001	44090000	TRAPPC10
21	44420001	44421000	TRPM2;TRPM2-AS
21	44699001	44700000	TSPEAR;KRTAP10-12;KRTAP10-13P
21	44771001	44772000	UBE2G2
21	45208001	45209000	ADARB1
21	45334001	45335000	LOC105372838;LINC00316
21	45393001	45394000	LL21NC02-21A1.1
21	45553001	45554000	SLC19A1
21	45738001	45739000	PCBP3
21	46035001	46036000	LOC105372842
21	46370001	46371000	PCNT
21	46699001	46700000	RPL23AP4
22	10739001	10740000	LOC105379418
22	11701001	11703000	LOC107987320;LOC107987320
22	11815001	11817000	LOC107984037;LOC107984037
22	11819001	11821000	LOC107984037;LOC107984030;LOC107984037;LOC107984030
22	11837001	11838000	LOC107984037;LOC107984030
22	11907001	11908000	LOC102723769
22	11917001	11918000	LOC102723769
22	12131001	12132000	LOC105379516;MTCO1P34;MTCO3P34
22	12170001	12173000	LOC105379516;LOC107987322;LOC105379516;LOC107987322;LOC105379516;LOC107987322
22	12175001	12178000	LOC105379516;LOC105379516;LOC105379516
22	15582001	15583000	LOC100420175;LOC100132723;LOC100422530
22	15776001	15777000	PSLNR;LOC107987324;DUXAP8
22	15855001	15856000	NBEAP3;TOMM40P2
22	17265001	17266000	CECR3
22	17517001	17518000	CECR2;DNAJA1P6
22	18785001	18786000	GGT3P
22	18849001	18850000	LOC102725072;BCRP7
22	19059001	19060000	DGCR2;LOC100129262
22	20458001	20459000	KLHL22;LOC100420177
22	21049001	21050000	P2RX6P;LRRC74B
22	21121001	21122000	BCRP2;POM121L7P;LOC112268299
22	21300001	21301000	POM121L8P;BCRP6;FAM230H
22	21600001	21601000	UBE2L3
22	21603001	21604000	UBE2L3
22	21970001	21971000	TOP3B
22	23164001	23165000	RSPH14;RAB36
22	23802001	23803000	SMARCB1
22	23815001	23816000	SMARCB1
22	23986001	23987000	DDT;GSTT2;GSTT4
22	24706001	24707000	LOC105372962;CRIP1P4
22	25031001	25032000	KIAA1671
22	29514001	29515000	THOC5
22	30544001	30545000	SEC14L6;SIRPAP1;GAL3ST1
22	32331001	32332000	SLC5A4;LOC107985561
22	32658001	32659000	SYN3;LOC105373002
22	32759001	32760000	SYN3
22	32825001	32826000	SYN3;TIMP3
22	33485001	33486000	LARGE1
22	34087001	34088000	LINC01643
22	34759001	34760000	LINC02885;LOC105373014
22	35407001	35408000	MCM5
22	35767001	35768000	RBFOX2
22	36025001	36026000	RBFOX2
22	36562001	36563000	CACNG2
22	36630001	36631000	CACNG2
22	36656001	36657000	CACNG2
22	36963001	36964000	CSF2RBP1;LL22NC01-81G9.3
22	37261001	37262000	LOC107985576
22	37298001	37299000	CYTH4;LOC105373024
22	37798001	37799000	H1-0;GCAT
22	38616001	38617000	FAM227A
22	38875001	38876000	CBX6
22	38991001	38992000	APOBEC3B;APOBEC3B-AS1
22	39045001	39046000	APOBEC3F
22	39102001	39103000	LOC101927202;APOBEC3H
22	39359001	39360000	SYNGR1
22	39511001	39512000	MIEF1;LOC105373035;ATF4
22	39572001	39573000	CACNA1I
22	40262001	40263000	TNRC6B
22	40284001	40285000	TNRC6B
22	41250001	41251000	CHADL;RANGAP1;MIR6889
22	41336001	41337000	ZC3H7B
22	41536001	41537000	ACO2;POLR3H

22	41545001	41546000	POLR3H;LOC105373044
22	41552001	41553000	POLR3H;LOC105373044;CSDC2
22	41583001	41584000	CSDC2;PMM1
22	41820001	41821000	CCDC134;SREBF2-AS1
22	42945001	42946000	PACSIN2
22	43058001	43059000	TTLL1-AS1;TTLL1
22	43180001	43181000	TTLL12
22	43228001	43229000	SCUBE1
22	43984001	43985000	SAMM50
22	44811001	44812000	PRR5-ARHGAP8;ARHGAP8
22	44868001	44869000	PRR5-ARHGAP8;ARHGAP8;LOC105373062
22	44926001	44927000	PHF21B
22	45419001	45420000	SMC1B;RIBC2
22	45667001	45668000	ATXN10
22	46395001	46396000	CELSR1
22	46623001	46624000	GRAMD4
22	46852001	46853000	TBC1D22A
22	47018001	47019000	TBC1D22A
22	47086001	47087000	TBC1D22A
22	47492001	47493000	LINC01644
22	48559001	48560000	TAFA5;LOC105373083
22	48612001	48613000	TAFA5
22	48674001	48675000	TAFA5
22	49562001	49563000	MIR3667HG
22	49663001	49664000	MIR3667HG
22	50157001	50158000	MOV10L1
22	50212001	50213000	SELENOO;LOC105373095;TUBGCP6
22	50335001	50336000	DENND6B;PPP6R2
22	50599001	50600000	CHKB-DT;MAPK8IP2
22	50625001	50626000	ARSA
22	50807001	50809000	RPL23AP82;RPL23AP82
X	270001	271000	PLCXD1
X	315001	316000	GTPBP6;LINC00685
X	367001	368000	PPP2R3B
X	423001	424000	LOC102724521
X	1198001	1199000	CRLF2
X	1221001	1222000	CRLF2
X	1339001	1340000	IL3RA;LOC101928032;LOC101928055
X	1347001	1348000	IL3RA;LOC101928032;LOC101928055
X	1414001	1415000	ASMTL-AS1;ASMTL
X	1449001	1450000	ASMTL
X	1609001	1610000	AKAP17A;ASMT
X	1712001	1713000	LOC107985706
X	1893001	1894000	LOC107985677
X	2355001	2356000	DHRSX
X	2713001	2714000	CD99
X	2734001	2735000	CD99
X	16182001	16183000	MAGEB17
X	16888001	16889000	SLC35C2P1
X	17772001	17773000	FAM136GP
X	18230001	18231000	BEND2;SCML2
X	18764001	18765000	PPEF1
X	19413001	19414000	MAP3K15
X	27292001	27293000	LOC105373150
X	30553001	30554000	TASL
X	39277001	39278000	LOC105373175
X	42768001	42769000	PPP1R2C
X	46951001	46952000	JADE3
X	48885001	48886000	TIMM17B
X	49215001	49216000	CACNA1F
X	49339001	49340000	GAGE12J;GAGE13;GAGE12B
X	49532001	49533000	GAGE12B;GAGE12C;GAGE12D
X	49534001	49535000	GAGE12B;GAGE12C;GAGE12D
X	49558001	49559000	GAGE12D;GAGE12E;GAGE12F
X	49562001	49563000	GAGE12E;GAGE12F;GAGE12G
X	49571001	49572000	GAGE12F;GAGE12G;GAGE12H
X	49591001	49592000	GAGE12H;GAGE2A;GAGE1
X	56768001	56769000	NBDY
X	56780001	56781000	NBDY
X	63429001	63430000	LINC01278
X	64848001	64849000	LOC105373239
X	65133001	65134000	ZC3H12B
X	65365001	65366000	ZC3H12B;PRXL2CP1
X	71628001	71629000	CXCR3
X	72715001	72716000	PHKA1;PHKA1-AS1
X	72844001	72845000	DMRTC1B
X	73127001	73128000	NAP1L6P
X	80406001	80407000	TENT5D
X	97514001	97515000	DIAPH2
X	113111001	113112000	LOC101928437
X	113177001	113178000	LOC101928437
X	124754001	124755000	TENM1
X	136511001	136512000	HTATSF1
X	143510001	143511000	SPANXN3
X	147234001	147235000	LOC105373347;MIR513A2;MIR506;MIR507;MIR508
X	149657001	149658000	LOC100420321
X	151914001	151915000	MAGEA4-AS1;MAGEA4

X	155304001	155305000	CLIC2
X	155493001	155494000	TMLHE-AS1;TMLHE
X	155880001	155881000	DPH3P2;VAMP7
X	156030001	156031000	WASH6P;DDX11L16
Y	11200001	11201000	LOC105379273
Y	11297001	11305000	DUX4L16;DUX4L16;DUX4L16;DUX4L16;DUX4L16;DUX4L16;DUX4L16;DUX4L16;DUX4L17
Y	11331001	11332000	DUX4L18;DUX4L19;PABPC1P5;SLC9B1P1
Y	26638001	26639000	PARP4P1
Y	56858001	56859000	CTBP2P1

(F) Yellow

Chr	Start	Stop	Annotation
1	94001	95000	LOC100996442
1	379001	380000	LOC112268260
1	432001	433000	LOC112268260;WBP1LP7
1	434001	435000	LOC112268260;WBP1LP7
1	668001	669000	WBP1LP6
1	821001	822000	FAM87B;LINC00115;LINC01128
1	908001	909000	LOC284600;LINC02593
1	975001	976000	KLHL17;PLEKHN1;PERM1
1	1004001	1005000	HES4;RPL39P12;ISG15
1	1871001	1872000	GNB1
1	1950001	1951000	CFAP74;LOC107984872
1	2435001	2436000	PLCH2
1	2568001	2569000	TNFRSF14;LOC100996583
1	2654001	2655000	TTC34
1	2693001	2694000	TTC34;LOC105378603;LOC105378602
1	2758001	2759000	TTC34;LOC105378601;LOC112268220;LOC107985732;LOC105378600;LOC105378598
1	2798001	2799000	TTC34
1	3954001	3956000	LINC01346;LINC01345;LINC01346;LINC01345
1	4184001	4185000	EEF1DP6
1	4435001	4436000	LOC105376674
1	4644001	4645000	AJAP1
1	4738001	4739000	AJAP1
1	6011001	6012000	KCNAB2
1	6021001	6022000	KCNAB2
1	6106001	6107000	KCNAB2;CHD5;LOC112268285
1	6278001	6279000	ACOT7
1	6482001	6483000	PLEKHG5
1	6563001	6565000	NOL9;TAS1R1;LOC107984912;NOL9;TAS1R1;LOC107984912
1	6569001	6570000	TAS1R1;LOC107984912;ZBTB48
1	6809001	6810000	CAMTA1
1	6840001	6841000	CAMTA1;RPL37P9
1	6917001	6918000	CAMTA1
1	6929001	6930000	CAMTA1
1	6952001	6953000	CAMTA1
1	6968001	6969000	CAMTA1
1	7011001	7012000	CAMTA1
1	7096001	7097000	CAMTA1
1	7115001	7116000	CAMTA1
1	7148001	7149000	CAMTA1
1	7160001	7161000	CAMTA1
1	7223001	7224000	CAMTA1;RNU1-8P
1	7325001	7326000	CAMTA1
1	7429001	7430000	CAMTA1
1	7655001	7656000	CAMTA1
1	7686001	7687000	CAMTA1;LOC105376689
1	7861001	7862000	UTS2
1	7986001	7987000	PARK7
1	8256001	8257000	LOC105376695
1	8509001	8510000	RERE
1	8613001	8614000	RERE
1	8634001	8635000	RERE
1	8727001	8728000	RERE
1	8921001	8923000	LOC112268261;LOC112268261
1	8924001	8925000	LOC112268261
1	8926001	8927000	LOC112268261
1	8963001	8964000	CA6
1	8973001	8974000	CA6;RN7SL451P
1	9092001	9093000	GPR157
1	9191001	9192000	MIR34AHG;LNCTAM34A
1	9352001	9353000	SPSB1
1	9356001	9357000	SPSB1
1	10754001	10755000	CASZ1
1	10785001	10786000	CASZ1
1	10790001	10791000	CASZ1
1	11057001	11058000	MASP2;SRM;EXOSC10
1	11131001	11132000	MTOR
1	11184001	11185000	MTOR;ANGPTL7
1	11250001	11251000	MTOR
1	11371001	11372000	LOC105376739;LOC105376740
1	11419001	11420000	MTCYBP45
1	11502001	11503000	DISP3;LOC112268221
1	11523001	11524000	DISP3
1	11530001	11531000	DISP3
1	11706001	11707000	DRAXIN
1	11765001	11766000	LOC101060126;C1orf167

1	11958001	11959000	PLOD1
1	11972001	11973000	PLOD1;MFN2
1	12084001	12085000	TNFRSF8;RNU6-777P;RPL23AP89
1	12105001	12106000	TNFRSF8
1	12178001	12179000	TNFRSF1B
1	12652001	12653000	AADACL4
1	12715001	12716000	AADACL3
1	13037001	13038000	PRAMEF28P
1	13559001	13560000	BRWD1P1
1	13562001	13564000	BRWD1P1;BRWD1P1
1	13598001	13599000	PDPN
1	13622001	13623000	PDPN;LOC107984919;RNA5SP41
1	13712001	13713000	PRDM2
1	13812001	13813000	PRDM2
1	13991001	13992000	KAZN
1	14581001	14582000	KAZN
1	14720001	14721000	KAZN
1	14894001	14895000	KAZN;LOC107985469
1	14901001	14902000	KAZN;LOC107985469
1	14927001	14928000	KAZN
1	15023001	15024000	KAZN
1	15336001	15337000	FHAD1;FHAD1-AS1
1	15441001	15442000	CTRC
1	16060001	16061000	CLCNKB;FAM131C
1	16064001	16065000	CLCNKB;FAM131C
1	16133001	16134000	EPHA2
1	16561001	16562000	PDE4DIPP8;LOC101056699;NBPF1
1	16566001	16567000	NBPF1
1	16606001	16607000	NBPF1;LOC105376794
1	16609001	16610000	NBPF1;LOC105376794;CROCCP2
1	16620001	16621000	NBPF1;LOC105376794;CROCCP2
1	16896001	16897000	LOC107985101;LOC105376805;TRN-GTT4-1;RNU1-2
1	16952001	16953000	CROCC
1	17076001	17077000	PADI2
1	17088001	17089000	PADI2
1	17305001	17306000	PADI4
1	17340001	17341000	PADI4
1	17346001	17347000	PADI4
1	17426001	17427000	RCC2
1	17700001	17701000	ARHGEF10L
1	17710001	17711000	LINC02810
1	18164001	18165000	IGSF21;IGSF21-AS1
1	18173001	18174000	IGSF21;IGSF21-AS1
1	18243001	18244000	IGSF21
1	19060001	19061000	LOC105376815
1	19071001	19072000	UBR4
1	19759001	19760000	TMCO4
1	19965001	19966000	RN7SL304P;PLA2G2A
1	20072001	20073000	PLA2G5
1	20185001	20186000	PLA2G2C;UBXN10
1	20197001	20198000	UBXN10;LOC105376825
1	20321001	20322000	VWA5B1
1	20368001	20369000	VWA5B1;LINC01141
1	20513001	20514000	MUL1
1	20557001	20558000	FAM43B
1	20672001	20673000	KIF17
1	21223001	21224000	ECE1
1	21352001	21353000	ECE1
1	21631001	21632000	RAP1GAP
1	21833001	21834000	LDLRAD2;HSPG2
1	21894001	21895000	HSPG2
1	22000001	22001000	CELA3A;RN7SL186P;RNU6-776P
1	22178001	22179000	LOC105376850
1	22411001	22412000	LOC105376856
1	22769001	22770000	EPHB2
1	22898001	22899000	EPHB2
1	23003001	23004000	TEX46
1	23060001	23061000	KDM1A
1	23093001	23094000	KDM1A;LUZP1
1	23214001	23215000	HTR1D
1	23406001	23407000	TCEA3
1	24077001	24078000	MYOM3;MYOM3-AS1
1	24168001	24169000	IFNLR1
1	24184001	24185000	IFNLR1
1	24215001	24216000	LINC02800
1	24219001	24220000	LINC02800
1	24516001	24517000	RCAN3
1	24669001	24670000	SRRM1
1	24867001	24868000	LOC105376876
1	25050001	25051000	LINC02793
1	25292001	25293000	RSRP1;RHD;SDHDP6
1	25295001	25296000	RSRP1;RHD;SDHDP6
1	25494001	25495000	MACO1
1	26164001	26165000	FAM110D;C1orf232;ZNF593
1	26295001	26296000	UBXN11
1	26989001	26990000	RPL12P13;RPL32P6;OSTCP2;TRNP1
1	27364001	27365000	MAP3K6;FCN3

1	27532001	27533000	LOC105376892;AHDC1
1	27562001	27563000	AHDC1
1	27861001	27862000	PPP1R8
1	27957001	27958000	SMPDL3B;XKR8
1	28268001	28269000	SESN2
1	28851001	28852000	OPRD1
1	28855001	28856000	OPRD1
1	29212001	29213000	MECR
1	29324001	29325000	PTPRU;LINC01756
1	29482001	29483000	LOC107984933
1	29697001	29698000	LOC107984934
1	30208001	30209000	LOC105378617
1	30732001	30733000	MATN1;MATN1-AS1;LAPTM5;MIR4420
1	30815001	30816000	LINC01778
1	30880001	30881000	SDC3
1	30896001	30897000	SDC3
1	31387001	31388000	LOC105378623;LOC107985470
1	31428001	31429000	SERINC2
1	31481001	31482000	LOC105379772;LOC441880
1	31664001	31665000	PEF1-AS1;COL16A1;LOC101929444
1	31708001	31709000	COL16A1
1	31729001	31730000	ADGRB2
1	31746001	31747000	ADGRB2
1	32272001	32273000	LCK
1	32339001	32340000	HDAC1;MARCKSL1;LOC105378629
1	32346001	32347000	MARCKSL1;LOC105378629;TSSK3
1	32432001	32433000	LRRC37A12P
1	32471001	32472000	ZBTB8B
1	33251001	33252000	ZNF362
1	33307001	33308000	ZNF362;A3GALT2
1	33379001	33380000	PHC2
1	34216001	34217000	C1orf94
1	34632001	34633000	LOC105378641
1	34636001	34637000	LOC105378641
1	34901001	34903000	DLGAP3;DLGAP3
1	35148001	35149000	LOC105378644
1	35437001	35438000	RNY5P1;KIAA0319L
1	35681001	35682000	LOC105378646
1	35689001	35690000	LOC105378646
1	36330001	36331000	SH3D21;EVA1B;STK40
1	36334001	36336000	SH3D21;EVA1B;STK40;SH3D21;STK40
1	36472001	36473000	MRPS15;CSF3R
1	36485001	36486000	CSF3R
1	36493001	36494000	CSF3R
1	36693001	36694000	LOC107984941;LOC105378648
1	36881001	36882000	GRIK3
1	36962001	36963000	GRIK3
1	37374001	37375000	LOC107984942
1	37725001	37726000	EPHA10
1	37908001	37909000	INPP5B
1	37913001	37914000	INPP5B
1	38090001	38091000	MIR3659HG;MIR3659
1	38418001	38420000	LOC105378657;LOC107984943;LOC105378657;LOC107984943
1	38425001	38428000	LOC105378657;LOC107984943;LOC105378657;LOC107984943;LOC105378657;LOC107984943
1	38430001	38431000	LOC105378657;LOC107984943
1	38687001	38688000	LOC105378660
1	39510001	39511000	BMP8A;TRK-CTT12-1;OXCT2P1
1	39821001	39822000	LINC02811;LOC105378667
1	39965001	39966000	MFSD2A;RPS2P12
1	40324001	40325000	COL9A2;RPL21P20
1	40424001	40425000	SMAP2
1	40426001	40427000	SMAP2
1	40659001	40660000	RIMS3;LOC105378675;LOC105378674
1	40809001	40810000	KCNQ4;RN7SL326P
1	40836001	40837000	KCNQ4
1	41358001	41359000	FOXO6
1	41426001	41427000	LOC105378678
1	41472001	41473000	LOC105378678;RNA5SP45;EDN2
1	41486001	41487000	EDN2
1	41764001	41765000	HIVEP3
1	42168001	42169000	GUCA2A;FOXJ3
1	43291001	43292000	C1orf210;TIE1
1	43308001	43309000	TIE1
1	43452001	43453000	SZT2;SZT2-AS1;MIR6735;HYI
1	43523001	43524000	PTPRF
1	43563001	43564000	PTPRF
1	43571001	43572000	PTPRF
1	43575001	43576000	PTPRF
1	43594001	43595000	PTPRF
1	43681001	43682000	KDM4A
1	43823001	43824000	ST3GAL3
1	43921001	43922000	ST3GAL3
1	43939001	43940000	ST3GAL3;ARTN;LOC105378688;LOC101929609;IPO13
1	44074001	44075000	KLF17
1	44122001	44123000	KLF17;RN7SL479P;LOC100129492
1	44127001	44128000	KLF17;RN7SL479P;LOC100129492;KLF18
1	44244001	44245000	ERI3;ERI3-IT1;SNORA110

1	44456001	44457000	RNF220
1	44500001	44501000	RNF220
1	44571001	44572000	RNF220
1	44602001	44603000	RNF220
1	44677001	44678000	TMEM53;SNORD145;ARMH1
1	44705001	44706000	ARMH1;LOC105378690
1	44716001	44717000	ARMH1;LOC105378690;RNU5F-1
1	44966001	44967000	EIF2B3;CCNB1IP1P1
1	45318001	45319000	HPDL
1	45322001	45323000	HPDL;MUTYH
1	45875001	45876000	MAST2;LOC105378694
1	46109001	46110000	P3R3URF-PIK3R3;PIK3R3
1	46341001	46342000	NSUN4
1	46479001	46480000	DMBX1
1	46512001	46513000	DMBX1
1	46541001	46542000	TMEM275;MKNK1-AS1;KNCN
1	47198001	47199000	PDZK1IP1
1	47948001	47949000	TRABD2B
1	48055001	48056000	LINC02794
1	48233001	48234000	SLC5A9
1	48687001	48688000	AGBL4
1	48714001	48715000	AGBL4
1	51964001	51965000	RAB3B;RNA5SP48
1	52612001	52613000	GPX7
1	52633001	52634000	SHISAL2A
1	52914001	52915000	ECHDC2;LOC112268226
1	53040001	53041000	SCP2
1	53184001	53185000	LOC105378724;CPT2P1
1	53503001	53504000	GLIS1
1	53614001	53615000	GLIS1
1	53654001	53655000	GLIS1
1	53795001	53796000	NDC1
1	53964001	53965000	LRRC42;HNRNPA3P12
1	54118001	54119000	TCEANC2
1	54171001	54172000	CYB5RL
1	54417001	54418000	SSBP3
1	54567001	54568000	ACOT11
1	54771001	54772000	PARS2;TTC22
1	54900001	54901000	DHCR24-DT
1	54995001	54996000	TMEM61;BSND
1	56214001	56215000	RPSAP20
1	56568001	56569000	PLPP3
1	57507001	57508000	DAB1
1	57510001	57511000	DAB1
1	58396001	58397000	DAB1
1	58693001	58694000	MYSM1
1	58721001	58722000	LOC112268263
1	59299001	59300000	FGGY-DT;FGGY
1	59580001	59581000	FGGY
1	60029001	60030000	C1orf87
1	60921001	60922000	LOC105378764
1	62088001	62089000	PATJ;MIR3116-1;MIR3116-2
1	62194001	62195000	PIGPP2;RPS15AP7;L1TD1
1	62266001	62267000	KANK4
1	62481001	62482000	DOCK7
1	62599001	62600000	DOCK7;ANGPTL3
1	63425001	63426000	ALG6
1	63434001	63435000	ALG6;ITGB3BP
1	63573001	63574000	EFCAB7
1	63709001	63710000	LOC105378771
1	64078001	64079000	ROR1
1	64699001	64700000	CACHD1
1	65001001	65002000	JAK1;LINC01359
1	65162001	65163000	AK4;RPS29P7
1	65210001	65211000	AK4
1	65335001	65336000	DNAJC6
1	65563001	65564000	LEPR
1	65646001	65648000	LEPR;LEPR
1	66254001	66255000	PDE4B
1	67153001	67154000	IL23R
1	67157001	67158000	IL23R
1	67791001	67792000	GNG12
1	68882001	68883000	LOC105378783
1	69928001	69929000	LRRC7;PIN1P1
1	70368001	70369000	HHLA3;HHLA3-AS1
1	74391001	74392000	FPGT-TNNI3K;TNNI3K
1	74528001	74529000	FPGT-TNNI3K;TNNI3K;LRRC53
1	74571001	74572000	ERICH3;ERICH3-AS1
1	74747001	74748000	TYW3
1	74750001	74751000	TYW3
1	76533001	76534000	ST6GALNAC3
1	76897001	76898000	ST6GALNAC5
1	78045001	78046000	LOC100131495;GIPC2
1	81295001	81296000	LOC107985018
1	81440001	81441000	ADGRL2
1	82665001	82666000	LOC105378814
1	82955001	82956000	LINC01362

1	82961001	82962000	LINC01362
1	85539001	85540000	DDAH1
1	85570001	85571000	DDAH1;CCN1
1	86342001	86343000	ODF2L
1	86484001	86485000	CLCA1
1	86683001	86684000	CLCA4-AS1
1	88457001	88458000	LOC105378839
1	89046001	89047000	LOC105378841;GBP1
1	92007001	92008000	BRDT
1	92236001	92237000	C1orf146;ACTBP12;GLMN
1	93105001	93106000	MTF2
1	93836001	93837000	BCAR3;MIR760
1	94027001	94028000	ABCA4
1	94108001	94109000	ABCA4
1	94219001	94220000	ARHGAP29
1	94572001	94573000	LOC105378861
1	94999001	95000000	ALG14
1	95031001	95032000	ALG14;LOC105378863
1	95102001	95103000	TLCD4
1	96245001	96246000	LINC01787
1	96806001	96807000	PTBP2
1	97908001	97909000	DPYD
1	98215001	98216000	LINC01776
1	98941001	98942000	PLPPR5
1	99087001	99088000	LOC100129620
1	99642001	99643000	PALMD
1	99788001	99789000	RNU4-75P
1	100631001	100632000	LINC01349
1	101815001	101816000	OLFM3
1	102932001	102933000	COL11A1
1	103922001	103923000	LOC100131348
1	108312001	108313000	SLC25A24P1
1	108473001	108475000	NBPF6;NBPF6
1	108613001	108614000	FAM102B
1	108617001	108618000	FAM102B
1	109118001	109119000	C1orf194;ELAPOR1
1	109302001	109303000	MYBPHL;SORT1
1	109319001	109320000	MYBPHL;SORT1
1	109408001	109409000	PSMA5
1	109486001	109487000	SYPL2;ATXN7L2;CYB561D1
1	109572001	109573000	GNAI3
1	109747001	109748000	GSTM3;EPS8L3
1	109889001	109890000	LINC01768
1	109918001	109919000	CSF1
1	110068001	110069000	ALX3
1	110107001	110108000	LINC01397;UBL4B
1	110147001	110148000	SLC6A17
1	110183001	110184000	SLC6A17
1	110444001	110445000	PROK1
1	110617001	110618000	KCNA2
1	110722001	110723000	LOC107985174
1	111407001	111408000	OVGP1
1	111829001	111830000	KCND3
1	112322001	112323000	LINC02884
1	112432001	112433000	CTTNBP2NL
1	112493001	112494000	WNT2B
1	112688001	112689000	MOV10
1	112811001	112812000	LINC01356
1	112813001	112815000	LINC01356;LINC01356
1	113610001	113611000	MAGI3
1	114080001	114081000	SYT6
1	114218001	114219000	LOC107985443
1	115303001	115304000	NGF-AS1;NGF
1	116516001	116517000	CD58
1	116870001	116871000	LOC105378927
1	116957001	116958000	PTGFRN;RNA5SP55
1	116962001	116963000	PTGFRN;RNA5SP55
1	117916001	117917000	GDAP2
1	117979001	117980000	SPAG17
1	119049001	119050000	WARS2;WARS2-IT1
1	119805001	119806000	REG4
1	120133001	120134000	LOC105378940;RNU6-465P
1	120595001	120596000	PDE4DIPP2
1	121269001	121270000	SRGAP2C
1	121291001	121292000	SRGAP2C
1	143318001	143319000	LOC101929814;LOC105371172;LOC105371175
1	143886001	143887000	H2BP2;FCGR1CP
1	144659001	144660000	LOC105371215;LOC101927429
1	144783001	144784000	LOC100996737
1	144864001	144865000	LOC102724269;LOC107985528
1	145010001	145011000	SRGAP2B
1	145523001	145524000	PDE4DIPP5
1	146721001	146722000	HYDIN2
1	146781001	146782000	HYDIN2
1	147587001	147588000	BCL9
1	147714001	147715000	LOC102723321;LOC105371230
1	148808001	148809000	PDE4DIP

1	149369001	149370000	SEC22B2P
1	149489001	149490000	NBPF19
1	149923001	149924000	SV2A;SF3B4;MTMR11
1	149944001	149945000	MTMR11;OTUD7B
1	150030001	150031000	LOC105371427
1	150512001	150513000	TARS2;ECM1;FALEC
1	151276001	151278000	PSMD4;ZNF687-AS1;ZNF687;PSMD4;ZNF687-AS1;ZNF687
1	151764001	151765000	RPS11P3;MRPL9;OAZ3;TDRKH
1	152100001	152101000	TCHH
1	153571001	153572000	S100A2
1	153680001	153681000	ILF2;TRX-CAT1-1;NPR1;MIR8083
1	153752001	153753000	INTS3
1	153973001	153974000	SLC39A1;CREB3L4;JTB;RAB13
1	154491001	154492000	SHE
1	154516001	154517000	TDRD10
1	154592001	154593000	CHRNB2;ADAR
1	154720001	154721000	KCNN3
1	154808001	154809000	KCNN3
1	154946001	154947000	PMVK;PBXIP1
1	155179001	155180000	KRTCAP2;TRIM46;MUC1
1	155287001	155288000	HCN3;PKLR
1	155298001	155299000	HCN3;PKLR;FDPS
1	155967001	155968000	ARHGEF2
1	156173001	156174000	SEMA4A
1	156552001	156553000	IQGAP3
1	156585001	156586000	TTC24;NAXE;GPATCH4
1	156653001	156654000	BCAN-AS1;BCAN
1	156808001	156809000	PRCC;SH2D2A;NTRK1
1	156812001	156813000	SH2D2A;NTRK1
1	156872001	156873000	NTRK1
1	156889001	156890000	NTRK1;PEAR1
1	156905001	156906000	PEAR1
1	156989001	156990000	ARHGEF11;RN7SL612P
1	157037001	157038000	ARHGEF11
1	157190001	157191000	LOC107985211
1	157996001	157997000	LOC105371459;KIRREL1
1	158071001	158072000	KIRREL1
1	158180001	158181000	LOC105371460;ELL2P1;CD1D
1	159024001	159025000	IFI16
1	159713001	159714000	CRPP1;CRP
1	159844001	159845000	SLAMF8;SNHG28;VSIG8;LOC107985216
1	159864001	159865000	SNHG28;VSIG8;LOC107985216;CFAP45
1	159982001	159983000	SLAMF9;LINC01133;FCRL6P1
1	160071001	160072000	KCNJ10;KCNJ9
1	160188001	160189000	ATP1A4;CASQ1
1	160364001	160365000	NCSTN;NHLH1
1	160379001	160380000	NHLH1;LOC105371466
1	161067001	161068000	ARHGAP30;NECTIN4
1	161745001	161746000	RN7SL466P;DUSP12
1	161914001	161915000	ATF6
1	164677001	164678000	PBX1
1	164849001	164850000	PBX1
1	165469001	165470000	LRRC52-AS1
1	166247001	166248000	LOC112268276
1	166291001	166292000	LOC112268276
1	167678001	167679000	RCSD1
1	167724001	167725000	TRP-CGG1-1;TRP-AGG2-1;MPZL1
1	167838001	167839000	ADCY10
1	168008001	168009000	DCAF6;MIR1255B2
1	168031001	168032000	DCAF6
1	168423001	168424000	LOC100505918
1	168491001	168492000	LOC101928565
1	169684001	169685000	LOC107985745;SELL
1	170093001	170094000	KIFAP3
1	171828001	171829000	RPLP1P3
1	172035001	172036000	DNM3
1	174742001	174743000	RABGAP1L
1	174797001	174798000	RABGAP1L
1	175648001	175649000	TNR
1	176930001	176931000	ASTN1
1	177241001	177243000	BRINP2;LOC105371625;BRINP2;LOC105371625
1	177353001	177354000	LINC01645
1	177995001	177996000	CRYZL2P-SEC16B
1	178041001	178042000	CRYZL2P-SEC16B;CRYZL2P
1	178788001	178789000	RALGPS2
1	178824001	178825000	RALGPS2
1	179288001	179289000	SOAT1
1	179972001	179974000	CEP350;RPSAP16;CEP350;RPSAP16
1	180172001	180173000	QSOX1
1	180193001	180194000	QSOX1
1	180258001	180259000	LHX4
1	180659001	180660000	XPR1
1	180897001	180898000	XPR1;LOC107985230;LINC02816
1	181450001	181451000	CACNA1E
1	181586001	181587000	CACNA1E
1	181606001	181607000	CACNA1E
1	181781001	181782000	CACNA1E;RNA5SP70

1	183346001	183347000	NMNAT2
1	183592001	183593000	NCF2;LOC100526839;LOC107985235
1	183614001	183615000	LOC107985235;ARPC5
1	183618001	183619000	LOC107985235;ARPC5
1	183659001	183660000	RGL1;APOBEC4
1	183866001	183867000	RGL1
1	184111001	184112000	LOC102724830
1	184447001	184448000	C1orf21
1	184897001	184898000	NIBAN1
1	185375001	185376000	LOC107985455
1	185950001	185951000	HMCN1
1	185952001	185953000	HMCN1
1	186306001	186308000	PRG4;TPR;RNU6-1240P;PRG4;TPR;RNU6-1240P
1	200162001	200163000	NR5A2
1	200180001	200181000	NR5A2
1	200375001	200376000	LINC00862
1	200653001	200654000	DDX59
1	200768001	200769000	CAMSAP2
1	201097001	201098000	CACNA1S
1	201111001	201112000	CACNA1S;ASCL5
1	201207001	201208000	IGFN1
1	201642001	201643000	NAV1
1	201810001	201811000	NAV1;IPO9-AS1;MIR1231
1	201915001	201916000	LMOD1
1	201918001	201919000	LMOD1
1	201923001	201924000	LMOD1
1	201931001	201932000	LMOD1
1	202239001	202240000	LGR6;LOC101929388
1	202287001	202288000	LGR6
1	202616001	202617000	SYT2
1	202819001	202820000	KDM5B;PCAT6;MGAT4EP
1	203121001	203122000	ADORA1
1	203140001	203141000	ADORA1
1	203304001	203305000	LINC01136;BTG2
1	203514001	203515000	OPTC;LOC105371688
1	204195001	204196000	KISS1;GOLT1A
1	204365001	204367000	PLEKHA6;LINC00628;PLEKHA6;LINC00628
1	204561001	204562000	MDM4;LOC105371692;RNA5SP74
1	204671001	204672000	LRRN2
1	204678001	204679000	LRRN2
1	204933001	204934000	NFASC
1	205007001	205008000	NFASC
1	205245001	205246000	TMCC2;TMCC2-AS1
1	205517001	205518000	CDK18
1	205638001	205639000	ELK4
1	205887001	205888000	LOC284581
1	205975001	205976000	SLC26A9-AS1;RAB7B
1	205989001	205990000	RAB7B
1	206287001	206288000	SRGAP2
1	206491001	206492000	IKBKE
1	206581001	206582000	RASSF5;EIF2D
1	206762001	206763000	LOC105372877;IL10;IL19
1	206914001	206915000	IL24;FCMR;RPL13AP8
1	206923001	206924000	FCMR;PIGR
1	208149001	208150000	PLXNA2
1	208704001	208705000	LOC105372892;RPS26P13
1	210113001	210114000	SYT14
1	210796001	210797000	KCNH1
1	211546001	211547000	LOC107985261;LOC107985260
1	212008001	212009000	INTS7
1	212295001	212296000	LINC02608;PPP2R5A
1	212645001	212646000	LINC02773;LOC100129948
1	212846001	212847000	SPATA45;RPS28P2;FLVCR1-DT
1	213360001	213361000	RPS6KC1
1	213417001	213418000	LOC105372912
1	213593001	213594000	LOC105372912
1	215003001	215004000	KCNK2
1	216008001	216009000	USH2A
1	216011001	216012000	USH2A
1	219157001	219158000	LYPLAL1-DT
1	219703001	219704000	LOC105372926
1	219737001	219738000	LOC105372926
1	220595001	220596000	MARK1
1	220801001	220802000	MTARC1
1	221826001	221827000	LINC01655
1	221966001	221967000	LINC02257;LINC02474
1	221968001	221969000	LINC02257;LINC02474
1	221971001	221972000	LINC02257;LINC02474
1	221975001	221976000	LINC02257;LINC02474
1	222463001	222464000	TRT-TGT2-1;CICP13;LOC728417
1	223088001	223089000	LOC102724046;LOC107985323
1	223109001	223110000	LOC107985323;TLR5
1	223377001	223378000	LOC105373025;RNU4-57P
1	223631001	223632000	CAPN8
1	223659001	223660000	CAPN8
1	223755001	223756000	CAPN2
1	224829001	224830000	LOC105373108

1	224888001	224889000	LOC105373109
1	224892001	224893000	LOC105373109
1	225116001	225117000	DNAH14
1	225373001	225374000	DNAH14
1	225387001	225388000	DNAH14
1	225776001	225777000	SRP9
1	225854001	225855000	EPHX1;TMEM63A
1	225947001	225948000	LEFTY2
1	226078001	226079000	H3-3A;LINC01703
1	226199001	226200000	LOC101927247;RPL34P7
1	226590001	226591000	STUM
1	226960001	226961000	COQ8A
1	227443001	227444000	BTF3P9
1	227560001	227561000	RNA5SP77;ZNF678
1	227562001	227563000	RNA5SP77;ZNF678;MRPL57P2
1	227791001	227792000	SNAP47;LOC105373289
1	227936001	227937000	WNT9A;MIR5008
1	228046001	228047000	WNT3A
1	228216001	228217000	OBSCN-AS1;OBSCN
1	228373001	228374000	OBSCN;LOC101927401
1	228677001	228678000	RHOU;FTH1P2
1	228681001	228683000	RHOU;FTH1P2;RHOU;FTH1P2
1	229176001	229177000	LINC02815;LINC02814
1	229416001	229417000	RN7SKP276
1	229418001	229419000	RN7SKP276
1	229644001	229645000	URB2
1	229874001	229875000	LOC105373162;LINC01682
1	230156001	230157000	GALNT2
1	230174001	230175000	GALNT2
1	230292001	230293000	GALNT2
1	230601001	230602000	LOC107985357;LINC01737;RPS24P4
1	230603001	230604000	LOC107985357;LINC01737;RPS24P4
1	230606001	230608000	LOC107985357;RPS24P4;LOC107985357;RPS24P4
1	230777001	230779000	CAPN9;LOC107985359;CAPN9;LOC107985359
1	230963001	230964000	TTC13
1	231571001	231572000	TSNAX-DISC1;TSNAX
1	231718001	231719000	TSNAX-DISC1;DISC1
1	231843001	231844000	TSNAX-DISC1;DISC1;LOC105373170
1	232230001	232231000	RN7SL299P
1	232232001	232233000	RN7SL299P
1	232400001	232402000	SIPA1L2;SIPA1L2
1	232637001	232638000	SIPA1L2
1	232909001	232910000	LOC101927711;LOC105373201
1	232991001	232992000	NTPCR;PCNX2
1	233040001	233041000	PCNX2
1	233222001	233223000	PCNX2
1	233282001	233283000	PCNX2;RPS7P3
1	233688001	233689000	LOC107985362
1	234308001	234309000	SLC35F3;MIR4671
1	234326001	234327000	SLC35F3
1	234482001	234483000	TARBP1
1	234662001	234664000	LOC101927787;LOC101927787
1	234783001	234784000	LOC107985364
1	235361001	235362000	LOC100418822;TBCE
1	235538001	235539000	MTND6P14;MTND5P19;MTND4P10;MTND4LP21;MTND3P8;MTCO3P46;GNG4
1	235541001	235543000	MTND6P14;MTND5P19;MTND4P10;MTND4LP21;MTND3P8;MTCO3P46;GNG4;MTND6P14;MTND5P19;MTND4P10;MTND4LP21;MTND3P8;MTCO3P46;GNG4
1	235673001	235674000	LYST
1	235723001	235724000	LYST
1	236161001	236162000	GPR137B
1	236247001	236248000	ERO1B
1	236844001	236845000	MTR
1	236935001	236938000	LOC107985368;LOC107985368;LOC107985368
1	237696001	237697000	RYR2
1	237949001	237951000	MTCO1P38;MTND2P27;NMTRP-TGG1-1;MTND1P25;NMTRL-TAA3-1;MTRNR2L11;MTCYBP15;MTND6P15;MTND5P18;MTCO1P38;MTND2P27;NMTRP-TGG1-1;MTND1P25;NMTRL-TAA3-1;MTRNR2L11;MTCYBP15;MTND6P15;MTND5P18
1	239378001	239379000	CHRM3
1	239760001	239761000	CHRM3
1	240285001	240286000	FMN2
1	240323001	240324000	FMN2
1	240536001	240537000	GREM2
1	240702001	240703000	LOC105373228
1	241436001	241437000	LOC105373230
1	241438001	241439000	LOC105373230
1	241921001	241922000	RPL23AP20
1	242188001	242189000	PLD5;RN7SKP12
1	242191001	242192000	PLD5;RN7SKP12
1	242262001	242263000	PLD5;LOC105373235
1	242388001	242389000	PLD5;LOC100421344
1	242463001	242464000	PLD5
1	244082001	244083000	LOC112268257
1	244374001	244375000	C1orf100
1	244638001	244639000	CATSPERE
1	244711001	244713000	DESI2;DESI2
1	245041001	245042000	EFCAB2;LOC107985725;RNU6-999P
1	245090001	245091000	EFCAB2

1	245124001	245125000	EFCAB2;RNU1-132P
1	245143001	245144000	RNU1-132P
1	245181001	245182000	KIF26B
1	245261001	245262000	KIF26B
1	245692001	245693000	KIF26B;LOC105373265
1	245798001	245799000	SMYD3;LOC105373270
1	245839001	245840000	SMYD3
1	245946001	245947000	SMYD3
1	246102001	246103000	SMYD3;SMYD3-AS1
1	246193001	246194000	SMYD3;RNU6-1283P
1	246730001	246731000	SCCPDH
1	246833001	246834000	AHCTF1
1	246963001	246964000	ZNF695;ZNF670-ZNF695
1	247277001	247278000	LOC105373273
1	247284001	247285000	LOC105373273
1	247334001	247335000	ZNF496;LOC107985115
1	247337001	247338000	ZNF496;LOC107985115
1	247416001	247417000	NLRP3
1	247446001	247447000	NLRP3;OR2B11
1	248171001	248172000	LOC100216488;OR2M2
1	248338001	248339000	OR14C36
1	248692001	248693000	OR14I1;LYPD9P
1	248742001	248743000	LYPD8;LOC107985747
1	248754001	248755000	LYPD8
1	248812001	248813000	SH3BP5L
2	361001	363000	LOC105373351;LOC105373351
2	919001	920000	LOC105373481
2	1411001	1412000	TPO
2	1529001	1530000	TPO
2	1711001	1712000	PXDN
2	1718001	1719000	PXDN
2	1942001	1943000	MYT1L
2	1991001	1992000	MYT1L
2	1993001	1994000	MYT1L
2	2251001	2252000	MYT1L
2	2307001	2308000	MYT1L
2	2848001	2849000	LOC105373390
2	3138001	3139000	LOC107985840
2	3304001	3305000	EIPR1
2	3352001	3353000	EIPR1
2	3361001	3362000	EIPR1
2	3370001	3371000	EIPR1;TRAPPC12
2	3744001	3745000	DCDC2C
2	3748001	3749000	DCDC2C
2	3776001	3777000	DCDC2C
2	3803001	3804000	DCDC2C
2	3816001	3817000	DCDC2C
2	3819001	3820000	DCDC2C
2	3838001	3839000	DCDC2C
2	4136001	4137000	LOC105373397;LOC107985841
2	6935001	6936000	RNF144A
2	7876001	7877000	LOC101929861;LOC105373409;LOC101929551
2	7985001	7986000	LINC00298
2	8172001	8173000	LINC00299
2	8190001	8191000	LINC00299
2	8623001	8624000	LOC105373411
2	8626001	8627000	LOC105373411
2	8706001	8707000	LOC107985849
2	8809001	8810000	KIDINS220
2	8839001	8840000	KIDINS220
2	9315001	9316000	ASAP2
2	9776001	9777000	LOC100996549
2	10207001	10208000	RRM2;LOC105373424
2	10399001	10400000	HPCAL1
2	10429001	10430000	HPCAL1;ODC1
2	10524001	10525000	LOC105373426
2	10561001	10562000	NOL10
2	10688001	10689000	NOL10;RN7SL832P
2	10840001	10841000	PDIA6
2	11112001	11113000	FLJ33534;RPL6P4
2	11304001	11305000	ROCK2;AIDAP1
2	11564001	11565000	GREB1;RNU2-13P;RNA5SP85
2	11585001	11586000	GREB1;RN7SL674P
2	11587001	11588000	GREB1;RN7SL674P
2	11637001	11638000	GREB1
2	11685001	11686000	LPIN1;LOC100506405
2	11856001	11858000	LOC105373430;LOC105373430
2	11876001	11877000	LOC105373430
2	12089001	12090000	MIR3681HG
2	12133001	12134000	MIR3681HG
2	12163001	12164000	MIR3681HG
2	12570001	12572000	MIR3681HG;MIR3681HG
2	13165001	13166000	LOC105373436;LOC105373484
2	13168001	13169000	LOC105373436;LOC105373484
2	15463001	15464000	NBAS
2	15918001	15919000	MYCNUT
2	17669001	17670000	SMC6

2	17711001	17712000	SMC6
2	19002001	19003000	LINC01376
2	19024001	19025000	LINC01376
2	20087001	20088000	LAPTM4A-DT
2	20500001	20501000	LOC107985856
2	20511001	20512000	LOC107985856
2	20592001	20593000	HS1BP3;HS1BP3-IT1
2	20648001	20649000	HS1BP3;LOC105373466
2	20768001	20769000	LDAH
2	21636001	21637000	LOC101929230
2	23049001	23050000	LOC107985792
2	23354001	23355000	LOC105374323
2	23384001	23385000	LOC105374323;KLHL29
2	23472001	23473000	KLHL29
2	23544001	23545000	KLHL29
2	23606001	23607000	KLHL29;LOC102723401
2	23871001	23872000	ATAD2B;PGAM1P6
2	24060001	24061000	FKBP1B;SF3B6
2	24167001	24168000	FAM228B;LOC105374328;FAM228A
2	24229001	24231000	ITSN2;ITSN2
2	24299001	24300000	ITSN2
2	24897001	24898000	ADCY3
2	25019001	25020000	DNAJC27-AS1
2	25249001	25250000	DNMT3A
2	25316001	25317000	DNMT3A
2	25386001	25387000	DTNB
2	25533001	25534000	DTNB
2	25559001	25560000	DTNB
2	25676001	25677000	DTNB
2	25992001	25993000	KIF3C;UQCRHP2
2	26150001	26151000	EMP2P1;SMARCE1P6
2	26336001	26337000	ADGRF3;SELENOI
2	26553001	26554000	OTOF;FAM166C
2	26788001	26790000	SLC35F6;CENPA;SLC35F6;CENPA
2	26945001	26946000	DPYSL5
2	27137001	27138000	ABHD1;PREB;PRR30
2	27797001	27798000	RBKS
2	28426001	28427000	FOSL2
2	28638001	28639000	PLB1
2	28880001	28881000	TRMT61B
2	28950001	28951000	WDR43
2	28988001	28989000	TOGARAM2
2	29014001	29015000	TOGARAM2
2	29339001	29340000	ALK;LOC101929386
2	29391001	29392000	ALK
2	29750001	29751000	ALK
2	29859001	29860000	ALK
2	29907001	29908000	ALK
2	30131001	30132000	LOC101929418
2	30248001	30249000	LBH
2	30442001	30443000	LCLAT1
2	31582001	31583000	SRD5A2
2	31863001	31864000	MEMO1
2	32177001	32178000	SLC30A6
2	32993001	32994000	LTBP1
2	33009001	33010000	LTBP1
2	33039001	33040000	LTBP1
2	33585001	33586000	FAM98A
2	33842001	33843000	LINC01317;SLC25A5P2
2	34695001	34696000	LINC01320;LOC107985865;LOC105374458
2	34697001	34698000	LINC01320;LOC107985865;LOC105374458
2	34803001	34804000	RN7SL602P
2	34805001	34807000	RN7SL602P;RN7SL602P
2	36455001	36456000	CRIM1
2	36538001	36540000	CRIM1;CRIM1
2	36555001	36556000	CRIM1;FEZ2
2	36601001	36602000	FEZ2
2	36603001	36605000	FEZ2;FEZ2
2	37173001	37174000	SULT6B1
2	37368001	37369000	QPCT;LOC107985868
2	37384001	37385000	LOC107985868
2	37863001	37864000	LINC00211
2	37979001	37980000	RMDN2;RMDN2-AS1
2	38059001	38060000	RMDN2;LOC107985872;CYP1B1
2	38773001	38774000	TTC39DP;NPLP1;GEMIN6
2	41941001	41942000	LINC01914;LINC02898
2	41990001	41991000	LOC105374517
2	42040001	42041000	PKDCC
2	42107001	42108000	LOC105374531
2	42525001	42526000	MTA3
2	43049001	43050000	LINC01819
2	43219001	43220000	ZFP36L2;LINC01126
2	44352001	44353000	PREPL;CAMKMT
2	44918001	44919000	LINC01833
2	45110001	45111000	LOC107985809
2	45187001	45188000	LINC01121
2	45344001	45345000	LOC105374577

2	45700001	45701000	PRKCE;TRQ-TTG5-1
2	46031001	46032000	PRKCE
2	46082001	46083000	PRKCE
2	46256001	46257000	LOC101926974;LOC105374581;RPL36AP14
2	46391001	46392000	EPAS1;LINC01820
2	46764001	46765000	SOCS5
2	46811001	46812000	LINC01118
2	47042001	47043000	TTC7A
2	47347001	47348000	EPCAM-DT
2	47390001	47391000	EPCAM
2	47538001	47539000	MSH2;KCNK12;MSH2-OT1
2	47588001	47589000	MSH2
2	48741001	48742000	STON1-GTF2A1L;LHCGR
2	48945001	48946000	FSHR
2	48987001	48988000	FSHR
2	52025001	52027000	LOC730100;LOC730100
2	52399001	52400000	LOC730100;LINC01867
2	53857001	53858000	GPR75-ASB3;MIR3682;GPR75;PSME4
2	53973001	53974000	PSME4;ACYP2
2	54508001	54509000	SPTBN1
2	54519001	54520000	SPTBN1;SPTBN1-AS1;RPL23AP32
2	58461001	58462000	LOC644456
2	58534001	58535000	LINC01122
2	60467001	60468000	BCL11A
2	62169001	62170000	PIGPP1
2	62561001	62562000	PSAT1P2;RSL24D1P2
2	63019001	63020000	EHBP1
2	64255001	64256000	LOC100507006
2	64257001	64258000	LOC100507006
2	64938001	64939000	LINC02245;LOC107985888
2	65281001	65282000	ACTR2
2	66631001	66632000	LINC01798
2	68731001	68732000	ARHGAP25
2	68805001	68806000	ARHGAP25
2	69445001	69446000	NFU1
2	70470001	70471000	TGFA;TGFA-IT1;LOC107985895
2	71086001	71087000	NAGK
2	71352001	71353000	ZNF638
2	71588001	71589000	DYSF
2	71607001	71609000	DYSF;DYSF
2	71690001	71691000	DYSF
2	71928001	71929000	LOC107985896
2	72040001	72041000	LOC105374798
2	72488001	72489000	EXOC6B
2	72742001	72743000	EXOC6B
2	72959001	72960000	SFXN5
2	73484001	73485000	ALMS1
2	73508001	73509000	ALMS1
2	73705001	73706000	LOC112268418;NAT8B
2	73895001	73896000	ACTG2
2	74249001	74250000	SLC4A5
2	75178001	75179000	TACR1;LOC105374811
2	75216001	75219000	LOC105374811;LOC105374811;LOC105374811
2	77093001	77094000	LRRTM4
2	77113001	77114000	LRRTM4
2	77970001	77971000	LOC101927967
2	79096001	79097000	REG1B
2	79789001	79790000	CTNNA2
2	81524001	81525000	LOC102724542
2	82816001	82817000	MTND4P25;MTND5P27;MTND6P7;MTCYBP7;LOC105374831
2	82820001	82821000	MTND4P25;MTND5P27;MTND6P7;MTCYBP7;LOC105374831
2	84445001	84446000	SUCLG1
2	84682001	84683000	DNAH6;LOC107985906
2	84719001	84720000	DNAH6;LOC107985906
2	84830001	84831000	TRABD2A
2	85184001	85185000	TCF7L1;TCF7L1-IT1
2	85309001	85310000	TCF7L1;LOC100996506;TGOLN2
2	85385001	85386000	ELMOD3;CAPG
2	85667001	85668000	SFTPB
2	85684001	85685000	LOC105374842;GPR160P1;GNLY
2	85982001	85983000	LOC105374843
2	87225001	87226000	CENPNP1;LOC107985771
2	87240001	87241000	LOC107985771;LINC01955
2	87286001	87287000	LOC107985908
2	87357001	87358000	LOC105377635
2	88437001	88438000	RPL38P6;FOXI3
2	88585001	88586000	LOC101928371;EIF2AK3
2	89056001	89057000	IGK;IGKV1-13
2	89581001	89582000	IGK;LOC107985911
2	89758001	89759000	IGK;ABCD1P1
2	90148001	90150000	IGK;IGKV1D-13;IGK;IGKV1D-13;IGKV1D-12
2	91618001	91619000	LOC388996
2	91631001	91632000	LOC388996;LSP1P4
2	94201001	94202000	BMS1P23;LOC107985915
2	94232001	94233000	LOC107985915
2	94595001	94596000	LOC100133920;LOC100509620
2	94947001	94948000	LOC442028;LOC105373487

2	94994001	94995000	RN7SL575P
2	95038001	95039000	MAL
2	95046001	95047000	MAL
2	95062001	95063000	MAL
2	95270001	95271000	PROM2
2	95633001	95634000	UBTFL3;LOC105373490
2	96607001	96608000	KANSL3
2	96650001	96651000	FER1L5
2	96711001	96712000	FER1L5;LMAN2L
2	96776001	96777000	CNNM4
2	96913001	96914000	FAM178B;LOC101927053
2	96939001	96940000	FAM178B
2	97202001	97203000	ANKRD36
2	97706001	97707000	C2orf92;ZAP70
2	97788001	97789000	TMEM131
2	98126001	98127000	VWA3B
2	98171001	98172000	VWA3B
2	98187001	98188000	VWA3B
2	98213001	98214000	VWA3B;ATP5F1BP1
2	98712001	98713000	MGAT4A
2	98772001	98773000	LINC02611;RNU4-84P
2	98895001	98896000	CRACDL
2	99191001	99192000	MITD1;MRPL30
2	99500001	99501000	REV1
2	99970001	99971000	AFF3
2	100143001	100144000	AFF3
2	100279001	100280000	LONRF2
2	100388001	100389000	CHST10
2	100810001	100811000	NPAS2
2	100945001	100946000	NPAS2
2	101139001	101140000	TBC1D8
2	101369001	101370000	CREG2;LOC107985789
2	101856001	101857000	MAP4K4
2	102003001	102004000	IL1R2
2	102103001	102104000	IL1R1
2	102781001	102782000	TMEM182
2	104984001	104985000	MRPS9-AS2
2	105102001	105103000	MRPS9;MRPS9-AS1;LOC105373527
2	105950001	105951000	LOC105373531
2	106160001	106161000	UXS1
2	106174001	106175000	UXS1
2	106453001	106454000	RGPD3
2	106469001	106470000	RGPD3;LOC105373534;LOC107985931
2	106724001	106725000	LOC102724744
2	106874001	106875000	ST6GAL2
2	107566001	107567000	LINC01886
2	108474001	108475000	GCC2
2	108734001	108735000	RANBP2
2	109335001	109336000	SH3RF3;LOC107985773
2	109405001	109406000	SH3RF3
2	110415001	110416000	LIMS4;LOC100288570
2	110564001	110565000	RGPD6
2	110598001	110599000	RGPD6
2	112080001	112081000	TMEM87B
2	112092001	112093000	TMEM87B
2	112164001	112165000	FBLN7
2	112418001	112419000	RGPD8
2	112719001	112720000	NT5DC4
2	113213001	113214000	PSD4;PAX8
2	113603001	113604000	WASH2P;DDX11L2;RPL23AP7
2	113922001	113923000	ACTR3
2	115189001	115190000	DPP10
2	117134001	117135000	RNU7-190P
2	119198001	119199000	LOC107985941
2	119294001	119295000	C2orf76
2	119455001	119456000	SCTR
2	119512001	119513000	SCTR
2	119659001	119660000	CFAP221
2	120214001	120215000	MTATP6P26;MTCO3P43;MTND3P10;MTND4P26;MTND5P28;TMEM185B
2	120303001	120304000	RALB
2	120333001	120334000	LOC105373989
2	120777001	120778000	GLI2
2	120794001	120795000	GLI2
2	121048001	121049000	LOC105373587
2	121768001	121769000	TSN;LOC105373590
2	122134001	122135000	LOC105373592
2	122147001	122148000	LOC105373592
2	122398001	122399000	LOC105373592
2	124381001	124382000	CNTNAP5
2	124860001	124861000	CNTNAP5
2	126638001	126639000	LOC105373602
2	127059001	127060000	BIN1
2	127352001	127353000	MAP3K2
2	127488001	127489000	IWS1
2	127619001	127620000	MYO7B;LOC101927834
2	127871001	127872000	LOC107985803;AMMECR1L
2	127951001	127952000	SAP130

2	128009001	128010000	SAP130
2	128513001	128514000	LOC105373611;ISCA1P6
2	128559001	128560000	LOC105373611
2	129256001	129257000	LINC01854;LOC105373612
2	130279001	130280000	MTND1P29;NMTRQ-TTG7-1;MTND2P22;MTCO1P7;MTCO2P7;MTATP6P7;MTND3P15;MTND4LP15;MTND4P27;MTND5P29;MTND6P8;MTCYBP8
2	130281001	130282000	MTND1P29;NMTRQ-TTG7-1;MTND2P22;MTCO1P7;MTCO2P7;MTATP6P7;MTND3P15;MTND4LP15;MTND4P27;MTND5P29;MTND6P8;MTCYBP8
2	130512001	130513000	POTEI;CFC1B
2	130609001	130610000	CFC1;POTEJ
2	130833001	130834000	ARHGEF4
2	131365001	131366000	LOC100420006;RAB6D;MTCYBP10;MTND6P10;MTND5P23;MTND4P21;MTND4LP28
2	131511001	131512000	SMPD4BP
2	131541001	131542000	CCDC74A;MED15P4
2	131803001	131804000	CDRT15P3
2	132470001	132471000	GPR39
2	133012001	133013000	NCKAP5
2	134143001	134144000	MGAT5
2	134459001	134460000	MGAT5;TMEM163
2	134583001	134584000	TMEM163
2	134852001	134853000	ACMSD;MIR5590
2	135446001	135447000	ZRANB3
2	136966001	136967000	THSD7B
2	137685001	137686000	THSD7B
2	137884001	137885000	LOC101928273
2	140426001	140428000	LRP1B;LRP1B
2	140430001	140431000	LRP1B
2	140463001	140464000	LRP1B
2	141166001	141167000	LRP1B;RNU6-904P
2	142935001	142936000	KYNU
2	143092001	143094000	ARHGAP15;MTCYBP11;MTND6P11;MTND5P24;MTND4P22;MTND3P9;MTCO3P5;MTATP6P5;MTCO2P5;ARHGAP15;MTCYBP11;MTND6P11;MTND5P24;MTND4P22;MTND3P9;MTCO3P5;MTATP6P5;MTCO2P5
2	143664001	143665000	ARHGAP15;ARHGAP15-AS1
2	143980001	143981000	GTDC1
2	145088001	145089000	LOC100505498
2	145872001	145873000	LOC105373665;LOC105373666
2	148098001	148099000	MBD5
2	148518001	148519000	MBD5
2	149002001	149003000	KIF5C
2	149369001	149370000	LYPD6
2	149407001	149408000	LYPD6
2	149778001	149779000	MMADHC-DT;LINC01931
2	149851001	149852000	MMADHC-DT;LINC01931
2	151253001	151254000	RBM43
2	151311001	151313000	LOC107985827;LOC107985827
2	151314001	151315000	LOC107985827
2	152502001	152503000	FMNL2
2	152751001	152752000	ARL6IP6
2	153980001	153981000	GALNT13
2	154868001	154869000	KCNJ3
2	155264001	155265000	MTCO1P45;LOC105373698;MTND2P20;ATP5F1AP2
2	156013001	156014000	LINC01876
2	156661001	156662000	LINC01958
2	158230001	158231000	CCDC148-AS1;CCDC148
2	159000001	159001000	TANC1;BTF3L4P2
2	162046001	162047000	DPP4
2	165246001	165247000	SCN2A
2	165603001	165604000	CSRNP3
2	166299001	166300000	SCN1A-AS1;SCN9A
2	167436001	167437000	B3GALT1
2	168577001	168578000	CERS6;MIR4774
2	169015001	169016000	ABCB11
2	169217001	169218000	LRP2
2	169272001	169273000	LRP2
2	169636001	169637000	PPIG;CCDC173
2	169732001	169733000	PHOSPHO2-KLHL23;KLHL23
2	170742001	170743000	LOC101926913;EIF2S2P4
2	170977001	170978000	GORASP2
2	171176001	171177000	TLK1
2	172902001	172903000	RAPGEF4;ALDH7A1P2
2	172919001	172920000	RAPGEF4
2	173235001	173236000	MAP3K20;MAP3K20-AS1
2	174049001	174050000	RPSAP24
2	175021001	175022000	RNU6-763P;RNU6-1290P
2	176769001	176770000	LOC101929963
2	177351001	177352000	LOC100130691;H3P7
2	177903001	177904000	PDE11A
2	178848001	178849000	CCDC141
2	178999001	179000000	CCDC141;RPS6P2
2	179053001	179055000	CCDC141;CCDC141
2	179056001	179057000	CCDC141
2	182328001	182329000	PDE1A
2	182823001	182824000	FRZB
2	186645001	186646000	ITGAV
2	187558001	187559000	TFPI
2	188035001	188036000	LINC01090
2	188126001	188127000	LINC01090

2	188165001	188166000	LINC01090
2	188169001	188170000	LINC01090
2	188171001	188172000	LINC01090
2	188174001	188175000	LINC01090
2	189022001	189023000	COL3A1;COL5A2
2	189756001	189757000	ANKAR;OSGEPL1;OSGEPL1-AS1;ORMDL1
2	190263001	190264000	HIBCH
2	191023001	191024000	STAT1;LOC105373805;LOC107985785;STAT4
2	191206001	191207000	LOC105373804
2	191269001	191270000	MYO1B
2	192133001	192134000	TMEFF2
2	194131001	194132000	GLULP6
2	194133001	194134000	GLULP6
2	195561001	195562000	LINC01827
2	197026001	197027000	ANKRD44;HNRNPA3P15;RPL4P7
2	197135001	197136000	ANKRD44
2	197581001	197582000	RFTN2
2	197586001	197587000	RFTN2
2	198292001	198293000	LINC01923
2	198729001	198730000	LOC105373831
2	199185001	199186000	RNU7-147P
2	199263001	199264000	SATB2
2	200313001	200314000	SPATS2L
2	200640001	200641000	AOX1
2	200891001	200892000	PPIL3;RNU6-312P;NIF3L1;RNU6-762P
2	201035001	201036000	FAM126B
2	201594001	201595000	C2CD6
2	201689001	201690000	MPP4
2	202338001	202339000	RN7SL753P;PIMREGP1;LOC107985976
2	202960001	202961000	CARF
2	203880001	203881000	CTLA4
2	203933001	203934000	LOC101927840;ICOS
2	203966001	203967000	ICOS
2	205395001	205396000	PARD3B
2	206809001	206810000	CPO
2	207075001	207076000	KLF7
2	207963001	207964000	PLEKHM3
2	208216001	208217000	RPSAP27;TPT1P2
2	208218001	208219000	RPSAP27;TPT1P2
2	208221001	208222000	TPT1P2
2	208749001	208750000	LOC101927960;LOC105373857
2	212985001	212986000	LOC102725082
2	215090001	215092000	ABCA12;ABCA12
2	215093001	215095000	ABCA12;ABCA12
2	215305001	215306000	ATIC
2	215328001	215329000	ATIC
2	216135001	216136000	XRCC5
2	216181001	216182000	XRCC5;LOC100421349
2	216485001	216486000	SMARCAL1;RPL37A-DT
2	216639001	216640000	IGFBP2
2	216670001	216671000	IGFBP2;IGFBP5
2	216826001	216827000	LOC101928278
2	217358001	217359000	DIRC3
2	217991001	217992000	TNS1;TNS1-AS1
2	218030001	218031000	RUFY4
2	218071001	218072000	RUFY4;CXCR2P1
2	218424001	218425000	VIL1
2	218430001	218431000	VIL1
2	218661001	218662000	ZNF142;BCS1L;RNF25
2	219016001	219017000	LOC100129175;CFAP65
2	219493001	219494000	SPEG;ASIC4-AS1;SPEGNB;GMPPA
2	219546001	219547000	ASIC4;CHPF;TMEM198;MIR3132;OBSL1
2	219608001	219609000	STK11IP
2	222772001	222773000	LOC105373903
2	222871001	222872000	ACSL3
2	223908001	223910000	WDFY1;WDFY1;GTF3AP3
2	224299001	224301000	LOC100289117;LOC100289117
2	224383001	224384000	FAM124B
2	224808001	224809000	DOCK10
2	226954001	226955000	RHBDD1
2	227130001	227131000	COL4A4
2	227753001	227754000	RNA5SP121
2	228542001	228543000	LINC01807
2	229543001	229544000	DNER
2	229724001	229725000	DNER
2	230177001	230178000	SP110
2	230575001	230576000	TPM3P8;LOC112268431
2	230708001	230709000	LINC01907;CAB39
2	231054001	231055000	C2orf72;LOC112268432;PSMD1
2	231281001	231282000	ARMC9
2	231377001	231378000	ARMC9
2	231389001	231390000	ARMC9;B3GNT7
2	231929001	231930000	NPPC
2	232020001	232022000	DIS3L2;DIS3L2
2	232528001	232529000	PRSS56;CHRND
2	232603001	232604000	LOC105373929;EFHD1
2	232890001	232891000	NGEF

2	233181001	233182000	INPP5D
2	233216001	233218000	INPP5D;INPP5D
2	233312001	233313000	SAG
2	233396001	233397000	DGKD
2	233792001	233793000	MROH2A
2	233952001	233953000	TRPM8
2	234075001	234076000	SPP2
2	234683001	234684000	LINC01173
2	234904001	234905000	LOC105373937
2	235140001	235141000	CEP19P1
2	235373001	235374000	LOC105373941
2	235822001	235823000	AGAP1
2	235841001	235842000	AGAP1
2	235939001	235940000	AGAP1
2	235967001	235968000	AGAP1
2	236014001	236015000	AGAP1
2	236042001	236043000	AGAP1;RNU7-127P
2	236046001	236047000	AGAP1;RNU7-127P
2	236103001	236104000	AGAP1
2	236116001	236117000	AGAP1
2	236365001	236366000	IQCA1;LOC105373945
2	236403001	236404000	IQCA1;RPL3P5
2	236544001	236545000	ACKR3;LOC105373991
2	236605001	236606000	LOC107986001;LOC105373946;LOC105373947
2	237415001	237416000	COL6A3
2	237500001	237501000	MLPH;MIR6811
2	237763001	237764000	LRRFIP1
2	237791001	237792000	LRRFIP1;RBM44
2	238109001	238110000	UBE2F-SCLY;SCLY;ESPNL
2	238208001	238209000	ILKAP
2	238235001	238236000	LINC02610;TARDBPP3;HES6;PER2
2	238563001	238564000	LINC01107;LOC112268434
2	238871001	238872000	TWIST2
2	239120001	239121000	HDAC4
2	239269001	239270000	HDAC4
2	239281001	239283000	HDAC4;HDAC4
2	239929001	239930000	NDUFA10
2	240055001	240056000	OR6B3
2	240226001	240227000	LOC107985836
2	240588001	240589000	RNPEPL1;CAPN10-DT;CAPN10
2	240611001	240612000	GPR35
2	240614001	240615000	GPR35
2	240623001	240624000	GPR35
2	240639001	240640000	GPR35
2	240693001	240694000	AQP12B;LOC285191;AQP12A
2	240776001	240777000	KIF1A
2	241095001	241097000	SNED1;MTERF4;SNED1;MTERF4;PASK
2	241309001	241310000	HDLBP;SEPTIN2
2	241619001	241620000	THAP4
2	241997001	241998000	LINC01237;LOC285097
3	119001	120000	LOC107986057
3	859001	861000	LOC107986058;LOC107986058
3	893001	894000	LOC107986059
3	3735001	3736000	LOC100130207
3	5015001	5016000	LOC105376934
3	5699001	5700000	LOC105376939;LOC105376938
3	8291001	8292000	LMCD1-AS1
3	8454001	8455000	LMCD1-AS1
3	8463001	8464000	LMCD1-AS1
3	9159001	9160000	SRGAP3
3	9213001	9214000	SRGAP3;SRGAP3-AS3
3	9496001	9497000	LHFPL4
3	9680001	9681000	MTMR14
3	9708001	9709000	MTMR14;CPNE9
3	9769001	9770000	OGG1;CAMK1;TADA3
3	9856001	9857000	MARK2P1;CIDEC
3	10294001	10295000	GHRLOS;LINC00852;GHRL;SEC13
3	10450001	10451000	ATP2B2
3	10612001	10613000	ATP2B2
3	10668001	10669000	ATP2B2
3	10839001	10840000	SLC6A11
3	11212001	11213000	HRH1;LOC102723663
3	11371001	11372000	ATG7
3	11753001	11754000	TAMM41
3	12357001	12358000	PPARG
3	12443001	12444000	PPARG
3	13049001	13050000	IQSEC1;RPL39P17
3	13652001	13653000	LOC100293612;LINC00620
3	13675001	13676000	LINC00620
3	13703001	13704000	LINC00620
3	13934001	13935000	VN1R20P;FGD5P1;TPRXL
3	14382001	14383000	LOC105376959
3	14729001	14730000	C3orf20
3	15280001	15281000	SH3BP5
3	15336001	15337000	SH3BP5;RNU6-454P
3	15763001	15764000	ANKRD28;LOC101927647
3	16366001	16367000	OXNAD1;RFTN1

3	16387001	16388000	OXNAD1;RFTN1
3	16493001	16494000	RFTN1
3	16531001	16532000	LINC00690
3	16959001	16960000	PLCL2
3	17083001	17084000	PLCL2
3	17332001	17333000	TBC1D5
3	18517001	18518000	SATB1-AS1
3	20068001	20069000	KAT2B
3	21039001	21040000	LOC105376987
3	21190001	21191000	LOC105376988
3	21192001	21193000	LOC105376988
3	23931001	23932000	NKIRAS1;RPL15
3	24160001	24161000	THRB;THRB-AS2
3	24871001	24872000	RARB
3	25304001	25305000	RARB
3	25584001	25585000	RARB
3	26160001	26161000	LOC105377002
3	26163001	26165000	LOC105377002;LOC105377002
3	27407001	27408000	SLC4A7
3	31764001	31765000	OSBPL10
3	31775001	31776000	OSBPL10
3	32074001	32075000	NIFKP7
3	33008001	33009000	GLB1;LOC107986073
3	33109001	33110000	RN7SL296P;CRTAP
3	33428001	33429000	FBXL2;UBP1
3	33714001	33715000	CLASP2
3	37165001	37166000	LRRFIP2;LOC112268444
3	37207001	37208000	LOC112268444;LOC105377642
3	37498001	37499000	ITGA9
3	37999001	38000000	VILL;PLCD1
3	38073001	38074000	DLEC1
3	38115001	38116000	DLEC1;LOC105377033;ACAA1
3	38413001	38414000	XYLB
3	38507001	38508000	EXOG;DDTP1
3	38737001	38738000	SCN10A
3	39467001	39468000	LOC105377038;MOBP
3	40443001	40444000	ENTPD3-AS1
3	41822001	41823000	ULK4
3	41964001	41965000	ULK4;RPL36P20
3	42735001	42736000	CCDC13;CCDC13-AS1
3	44346001	44347000	TOPAZ1;LOC105377055;TCAIM
3	44673001	44674000	ZKSCAN7-AS1;LOC100419752
3	44855001	44857000	KIF15;HNRNPA1P77;MIR564;TMEM42;KIF15;MIR564;TMEM42
3	45074001	45075000	CDCP1
3	46390001	46391000	CCR5AS
3	46478001	46479000	LTF
3	46749001	46750000	PRSS45P;PRSS43P
3	46844001	46845000	PRSS42P
3	46923001	46924000	CCDC12
3	47375001	47376000	PTPN23
3	47866001	47867000	MAP4
3	48567001	48568000	PFKFB4;UCN2;COL7A1
3	48590001	48591000	COL7A1;UQCRC1
3	48629001	48630000	TMEM89;SLC26A6;MIR6824;CELSR3
3	48638001	48639000	SLC26A6;MIR6824;CELSR3;MIR4793
3	48643001	48644000	SLC26A6;MIR6824;CELSR3;MIR4793
3	48684001	48685000	NCKIPSD;IP6K2
3	49126001	49127000	USP19;LAMB2
3	49159001	49160000	LAMB2P1;CCDC71
3	49766001	49767000	IP6K1;COX6CP14;PHF5EP
3	50125001	50126000	RBM5;SEMA3F-AS1
3	50141001	50142000	SEMA3F-AS1
3	50534001	50535000	LOC105377083
3	51403001	51404000	RBM15B;DCAF1
3	51457001	51458000	DCAF1
3	51693001	51694000	TEX264;RNA5SP132
3	51714001	51715000	TEX264;GRM2
3	51876001	51877000	RN7SL504P;IQCF5-AS1;IQCF5
3	51896001	51897000	IQCF1
3	51958001	51959000	PARP3;GPR62;PCBP4;ABHD14B
3	52081001	52082000	POC1A
3	52224001	52225000	ALAS1;TLR9;TWF2
3	52339001	52340000	DNAH1
3	52342001	52343000	DNAH1
3	52376001	52377000	DNAH1;PPP2R5CP
3	52379001	52380000	DNAH1;PPP2R5CP
3	52774001	52775000	NEK4;ITIH1
3	53067001	53068000	SERBP1P3;RFT1
3	53140001	53141000	RFT1
3	53490001	53491000	CACNA1D
3	53534001	53535000	CACNA1D
3	53568001	53569000	CACNA1D
3	53587001	53588000	CACNA1D
3	53641001	53642000	CACNA1D
3	54596001	54597000	CACNA2D3
3	54627001	54628000	CACNA2D3;LOC107986088;RPS15P5;ESRG
3	54639001	54640000	CACNA2D3;ESRG

3	54922001	54923000	CACNA2D3;LRTM1
3	55076001	55077000	CACNA2D3
3	55474001	55475000	WNT5A
3	55776001	55777000	ERC2
3	57304001	57305000	DNAH12
3	57307001	57308000	DNAH12
3	58326001	58327000	RPP14;HTD2;PXK
3	58441001	58442000	PDHB
3	58594001	58596000	FAM107A;FAM107A
3	58817001	58818000	CFAP20DC;CFAP20DC-AS1
3	58843001	58844000	CFAP20DC;CFAP20DC-AS1
3	59151001	59152000	LOC105377110
3	59312001	59313000	LOC105377110
3	59326001	59327000	LOC105377110
3	59953001	59954000	FHIT
3	63924001	63925000	ATXN7
3	64077001	64078000	PRICKLE2-AS1;LINC00994
3	64209001	64210000	PRICKLE2;PRICKLE2-AS3
3	65185001	65186000	LOC105377127;LINC02040
3	65420001	65421000	MAGI1
3	65495001	65496000	MAGI1
3	65541001	65542000	MAGI1
3	66183001	66184000	SLC25A26;LOC107986095
3	66185001	66186000	SLC25A26;LOC107986095
3	66188001	66189000	SLC25A26;LOC107986095
3	66776001	66777000	LOC105377144
3	67796001	67797000	SUCLG2-AS1
3	69119001	69120000	LMOD3
3	71226001	71227000	FOXP1
3	71790001	71791000	PROK2;LOC105377156
3	71825001	71826000	RN7SL271P
3	72025001	72027000	LINC00877;LINC00877
3	72577001	72578000	RNU1-62P;LOC105377161
3	72672001	72673000	LOC105377161;LOC105377162
3	73447001	73448000	PDZRN3
3	75472001	75473000	LINC02018;ENPP7P2
3	75475001	75476000	LINC02018;ENPP7P2
3	75724001	75725000	ZNF717
3	76537001	76538000	ROBO2
3	76583001	76584000	ROBO2
3	76691001	76692000	ROBO2
3	78587001	78588000	ROBO1
3	78806001	78807000	ROBO1
3	81067001	81068000	LINC02027
3	93939001	93941000	PROS1;HSPE1P17;PROS1;HSPE1P17
3	94097001	94099000	NSUN3;NSUN3
3	98060001	98061000	OR5BM1P;OR5AC1
3	98195001	98196000	LOC105373998;LOC105373997;OR5H5P
3	98249001	98250000	OR5H7P
3	98785001	98786000	ST3GAL6;PDLIM1P4;DCBLD2
3	100922001	100923000	ABI3BP;LOC100421580
3	104445001	104446000	LOC105374020
3	106900001	106901000	MTND4P16;MTND4LP3;MTND3P6;MTCO3P35;MTND5P16;MTND6P6;MTND1P16;NMTRQ-TTG4-1;MTND2P14
3	106923001	106925000	LOC107986021;LOC107986021
3	107441001	107442000	LINC01990
3	110636001	110637000	LOC105374037
3	112419001	112421000	LOC112268446;LOC112268446
3	112422001	112423000	LOC112268446
3	112993001	112994000	GTPBP8;NEPRO
3	113655001	113656000	USF3
3	114295001	114296000	TIGIT
3	114307001	114308000	TIGIT;ZBTB20;MIR568
3	115196001	115197000	LOC101926886
3	115794001	115795000	LSAMP
3	115797001	115798000	LSAMP
3	116106001	116110000	LSAMP;LSAMP;LSAMP;LSAMP
3	117676001	117677000	LOC100421670;LINC02024
3	117753001	117754000	LOC107986022;LOC101926953
3	118659001	118660000	LOC105374060
3	119646001	119647000	RPL10P7;POPDC2
3	119978001	119979000	GSK3B
3	120452001	120453000	FSTL1
3	120493001	120494000	LOC107986120;LOC107986121
3	122822001	122823000	SLC49A4
3	123348001	123349000	ADCY5
3	124296001	124297000	KALRN
3	124562001	124563000	KALRN;LOC105374076
3	124631001	124632000	KALRN;LOC100420144
3	124671001	124672000	KALRN
3	124757001	124758000	UMPS;ITGB5
3	124775001	124776000	ITGB5
3	124867001	124868000	ITGB5;ENO1P3
3	124882001	124883000	ITGB5
3	125067001	125068000	RNA5SP137
3	125598001	125599000	OSBPL11
3	125800001	125801000	LOC112267908;LOC105374312;MIR548I1;RPS3AP14
3	125867001	125868000	LINC02614;ENPP7P4

3	126035001	126036000	SLC41A3
3	126052001	126053000	SLC41A3
3	126316001	126317000	KLF15;LOC105374088
3	126476001	126477000	ZXDC;UROC1
3	126978001	126979000	PLXNA1
3	127487001	127488000	LINC01471
3	127545001	127546000	LINC01471;LINC02034
3	127657001	127658000	PODXL2
3	128285001	128286000	EEFSEC
3	128420001	128421000	EEFSEC
3	128784001	128785000	RAB7A;RN7SL698P
3	128818001	128819000	RAB7A
3	128832001	128833000	MARK2P17
3	128839001	128842000	MARK2P17;MIR12124;MARK3P3;MARK2P17;MIR12124;MARK3P3;MARK2P17;MIR12124;MARK3P3;MARK2P6
3	128961001	128962000	CFAP92
3	129263001	129264000	COPG1;MIR6826
3	129320001	129321000	H1-10;H1-10-AS1;NUP210P3
3	129484001	129485000	IFT122
3	130008001	130009000	RPS17P9
3	130150001	130151000	LOC105374103;LOC107986131
3	130212001	130213000	COL6A4P2
3	130512001	130513000	COL6A6
3	132230001	132231000	CPNE4;TRC-GCA6-1;TRC-GCA9-1
3	132653001	132654000	NPHP3-ACAD11;ACAD11;HSPA8P19;UBA5
3	132882001	132883000	NPHP3-AS1
3	133431001	133432000	BFSP2;BFSP2-AS1
3	133433001	133434000	BFSP2;BFSP2-AS1
3	133967001	133968000	SLCO2A1
3	134000001	134001000	SLCO2A1
3	134201001	134202000	RYK
3	134623001	134624000	CEP63;KY
3	136563001	136564000	STAG1
3	137004001	137005000	IL20RB
3	138286001	138287000	ARMC8;NME9
3	138534001	138536000	CEP70;CEP70
3	138673001	138674000	PIK3CB
3	140291001	140292000	CLSTN2
3	141128001	141129000	SPSB4
3	141230001	141231000	PXYLP1
3	141270001	141271000	PXYLP1
3	141339001	141340000	ZBTB38
3	141511001	141512000	RASA2
3	141618001	141619000	RASA2
3	141699001	141700000	LINC02618;TPT1P3
3	141702001	141703000	LINC02618;TPT1P3
3	141763001	141764000	GRK7
3	142507001	142508000	ATR
3	142826001	142827000	PCOLCE2;RPL8P3
3	142851001	142852000	PCOLCE2
3	143517001	143518000	SLC9A9;ST13P15
3	143798001	143799000	SLC9A9
3	145551001	145552000	LOC105374142
3	145554001	145556000	LOC105374142;LOC105374142
3	146165001	146166000	PLOD2;LOC105374145
3	148156001	148157000	HNRNPA1P20
3	148956001	148957000	LOC107986045
3	150601001	150602000	SELENOT;LOC677762
3	151811001	151812000	AADACL2-AS1;LOC105374160;AADAC
3	151813001	151814000	AADACL2-AS1;LOC105374160;AADAC
3	153226001	153227000	LOC105374164
3	153228001	153230000	LOC105374164;LOC105374164
3	155563001	155564000	PLCH1
3	156835001	156836000	LEKR1
3	158834001	158835000	MFSD1;GPR79
3	159431001	159432000	IQCJ-SCHIP1;SCHIP1
3	162154001	162155000	LOC107986048
3	164023001	164025000	LOC102724419;LOC102724419
3	166811001	166812000	CBX1P5
3	167433001	167434000	SERPINI2
3	167436001	167437000	SERPINI2
3	168931001	168932000	LINC02082
3	169433001	169434000	MECOM
3	170682001	170684000	SLC7A14-AS1;SLC7A14-AS1
3	170686001	170688000	SLC7A14-AS1;SLC7A14-AS1
3	170820001	170821000	LOC105374211
3	170822001	170823000	LOC105374211
3	170912001	170913000	EIF5A2
3	171159001	171160000	TNIK;LOC105374216
3	171783001	171784000	PLD1
3	172113001	172114000	FNDC3B
3	172427001	172428000	BZW1P1
3	172511001	172512000	TNFSF10
3	172716001	172717000	NCEH1
3	173159001	173160000	LOC105374220
3	173560001	173561000	NLGN1
3	174264001	174265000	NLGN1
3	177670001	177671000	LINC00578

3	178211001	178212000	LOC107986155
3	179507001	179508000	LOC107986157
3	179517001	179518000	LOC107986157
3	179524001	179525000	LOC107986157
3	179611001	179612000	MRPL47;NDUFB5
3	179688001	179689000	USP13
3	179778001	179779000	USP13
3	181088001	181089000	SOX2-OT
3	181705001	181706000	SOX2-OT;SOX2
3	182505001	182506000	LINC01995
3	182509001	182510000	LINC01995
3	182675001	182676000	LINC02031
3	182952001	182953000	DCUN1D1;LOC100505642
3	183039001	183040000	MCCC1
3	183630001	183631000	LOC105374248;LOC107986162;KLHL24
3	183803001	183804000	YEATS2;YEATS2-AS1
3	183911001	183912000	CYP2AB1P;ABCC5
3	184246001	184247000	VWA5B2;MIR1224;ALG3;EEF1AKMT4-ECE2;EEF1AKMT4
3	184333001	184334000	EIF4G1;SNORD66;FAM131A
3	184351001	184352000	FAM131A;CLCN2;POLR2H
3	184372001	184373000	POLR2H;THPO;CHRD
3	184375001	184376000	POLR2H;THPO;CHRD
3	184502001	184503000	LOC391600;LINC01839
3	184558001	184559000	LINC01839;LINC01840;EPHB3
3	185201001	185202000	EHHADH-AS1;EHHADH
3	185400001	185401000	MAP3K13
3	186260001	186261000	DGKG
3	186306001	186307000	DGKG
3	186485001	186486000	LINC02052;LINC02051
3	186627001	186628000	AHSG;FETUB
3	186661001	186662000	FETUB;LOC105374258;HRG
3	186718001	186719000	LOC105374258;KNG1
3	187055001	187056000	ST6GAL1
3	187204001	187205000	LOC101929106;RTP1
3	187790001	187791000	LOC105374263
3	187891001	187892000	LOC105374264
3	188050001	188051000	LOC107986166
3	188179001	188180000	LPP;FLJ42393
3	188262001	188263000	LPP
3	188308001	188309000	LPP
3	188361001	188362000	LPP
3	188581001	188582000	LPP
3	189319001	189320000	TPRG1
3	190145001	190146000	P3H2-AS1
3	191323001	191324000	UTS2B;CCDC50
3	192225001	192226000	FGF12
3	192350001	192351000	FGF12
3	193456001	193457000	ATP13A4
3	193596001	193597000	ATP13A4;OPA1
3	193707001	193708000	OPA1
3	193862001	193863000	LOC105374287
3	194147001	194148000	HES1;RN7SL215P
3	194156001	194157000	LOC107986172
3	194202001	194203000	LINC02036
3	194225001	194226000	LINC02036
3	194337001	194338000	CPN2
3	194347001	194348000	CPN2;LRRC15
3	194609001	194610000	TMEM44;LOC105374291
3	194681001	194682000	LSG1;FAM43A
3	194724001	194725000	LOC105374292;LINC01968
3	194845001	194846000	LOC107986174;LOC107986173
3	195079001	195080000	XXYLT1
3	195128001	195129000	XXYLT1;MIR3137
3	195697001	195698000	SDHAP2;MIR570HG;MIR570;SMBD1P
3	195778001	195780000	MUC4;MUC4
3	195782001	195784000	MUC4;MUC4
3	195785001	195787000	MUC4;MUC4
3	195802001	195803000	MUC4
3	195811001	195812000	MUC4
3	195845001	195846000	LINC01983;LOC107984010;KIF3AP1
3	196345001	196346000	TM4SF19-DYNLT2B;TM4SF19;RNU6-910P;UBXN7
3	196487001	196488000	RNF168;LOC105374306
3	196634001	196635000	LINC01063;NRROS
3	197515001	197516000	LINC02012;BDH1
3	197552001	197553000	BDH1
3	197646001	197647000	LOC107986176;LOC112268458
3	197720001	197721000	RUBCN
3	197756001	197757000	RUBCN;FYTTD1
3	197925001	197926000	IQCG
3	198038001	198039000	LMLN;LMLN-AS1
4	44001	45000	LOC105374377;ZNF595
4	51001	52000	LOC105374377;ZNF595
4	109001	110000	LOC100129037
4	475001	476000	ABCA11P;ZNF721;LOC100533735
4	709001	710000	LOC107986245;PCGF3
4	945001	946000	TMEM175
4	1296001	1297000	LOC105374347;LOC105374346;MAEA

4	1498001	1500000	LOC105374348;LOC105374348
4	1968001	1969000	NSD2;SCARNA22
4	2295001	2296000	ZFYVE28
4	2391001	2392000	ZFYVE28
4	2409001	2410000	ZFYVE28;CFAP99
4	2645001	2646000	FAM193A
4	2895001	2896000	ADD1
4	3184001	3185000	HTT
4	3573001	3574000	LINC00955
4	3627001	3628000	LOC112268461
4	3932001	3933000	LOC100420818;ALG1L7P;FAM86EP
4	4000001	4001000	LOC105374359
4	4191001	4192000	OTOP1
4	4224001	4225000	OTOP1
4	5385001	5386000	STK32B
4	5701001	5702000	EVC2;EVC
4	5746001	5747000	EVC
4	6066001	6067000	JAKMIP1
4	6086001	6087000	JAKMIP1
4	6149001	6150000	JAKMIP1
4	6234001	6235000	JAKMIP1-DT
4	6340001	6341000	PPP2R2C
4	6368001	6369000	PPP2R2C
4	6510001	6511000	PPP2R2C
4	6596001	6597000	MAN2B2
4	6764001	6765000	LOC105374367
4	6794001	6795000	KIAA0232
4	6879001	6880000	KIAA0232
4	6934001	6935000	TBC1D14
4	6964001	6965000	TBC1D14
4	7334001	7335000	SORCS2;TRSUP-TTA3-1
4	7441001	7443000	SORCS2;PSAPL1;SORCS2;PSAPL1
4	7574001	7575000	SORCS2;LOC107986258
4	7617001	7618000	SORCS2
4	7625001	7626000	SORCS2
4	7677001	7678000	SORCS2
4	7848001	7849000	AFAP1
4	7896001	7897000	AFAP1
4	7950001	7951000	LOC389199
4	7970001	7971000	ABLIM2
4	8035001	8036000	ABLIM2
4	8485001	8486000	TRMT44
4	8741001	8742000	LOC101928532
4	10113001	10114000	WDR1;RNA5SP155
4	11448001	11449000	LOC107986178
4	14852001	14853000	LINC00504
4	14884001	14885000	LINC00504
4	14986001	14987000	CPEB2-DT
4	15734001	15735000	BST1;RPL10AP7;LOC100288771
4	16054001	16055000	PROM1
4	16056001	16057000	PROM1
4	16108001	16109000	LOC107986261
4	17649001	17650000	FAM184B
4	17741001	17742000	FAM184B
4	17782001	17783000	FAM184B
4	18009001	18010000	LCORL
4	19016001	19017000	LOC107986263
4	19890001	19891000	LOC105374511
4	19892001	19893000	LOC105374511
4	20434001	20435000	SLIT2
4	22122001	22123000	RNU6-420P
4	24324001	24325000	PPARGC1A
4	24326001	24328000	PPARGC1A;PPARGC1A
4	24672001	24673000	LINC02473
4	25170001	25171000	SEPSECS;SEPSECS-AS1
4	25250001	25252000	PI4K2B;PI4K2B
4	25273001	25274000	PI4K2B
4	25576001	25577000	LOC101929161
4	25594001	25595000	RNU7-126P
4	25719001	25720000	SEL1L3;MTND4P9;MTND4LP22;MTND3P5;MTCO3P44
4	25843001	25844000	SEL1L3
4	26721001	26722000	TBC1D19
4	26731001	26732000	TBC1D19
4	29805001	29806000	LOC105374562
4	36346001	36347000	DTHD1;LOC105374399
4	39769001	39770000	UBE2K;RN7SL558P;ZBTB12BP
4	39836001	39837000	PDS5A
4	40594001	40595000	RBM47
4	40609001	40610000	RBM47
4	40835001	40836000	APBB2
4	40878001	40879000	APBB2
4	40890001	40891000	APBB2
4	41130001	41131000	APBB2
4	41195001	41196000	APBB2
4	41482001	41483000	LIMCH1
4	42547001	42548000	ATP8A1
4	47070001	47071000	GABRB1

4	47520001	47521000	ATP10D
4	48555001	48556000	FRYL
4	49516001	49517000	ANKRD20A17P;LOC107986214
4	52734001	52735000	ERVMER34-1
4	52810001	52811000	LINC01618
4	52817001	52818000	LINC01618
4	54653001	54654000	KIT
4	54708001	54709000	KIT
4	54755001	54756000	LOC105377657
4	55077001	55078000	KDR
4	56060001	56061000	CRACD
4	61766001	61767000	ADGRL3;RPS12P9
4	61768001	61769000	ADGRL3;RPS12P9
4	61944001	61945000	ADGRL3
4	64609001	64610000	MTCO3P27;MTCYBP16;MTND6P16;MTND5P13;MTCO3P28
4	67502001	67503000	CENPC
4	67672001	67673000	UBA6
4	67749001	67750000	GNRHR
4	68514001	68515000	UGT2B29P
4	68672001	68673000	UGT2B15
4	70513001	70514000	AMTN
4	71032001	71033000	DCK
4	71036001	71037000	DCK
4	74453001	74454000	AREG
4	74589001	74590000	LOC107986229
4	75017001	75018000	PARM1
4	75020001	75021000	PARM1
4	75985001	75986000	SDAD1;SDAD1-AS1
4	77011001	77012000	SEPTIN11;LOC105377291
4	78117001	78118000	FRAS1
4	78349001	78350000	FRAS1
4	78578001	78579000	ANXA3
4	78714001	78715000	LOC101928893
4	79555001	79557000	LINC00989;LOC107986294;LINC00989;LOC107986294
4	79559001	79560000	LINC00989;LOC107986294
4	80820001	80821000	CFAP299
4	80860001	80861000	CFAP299
4	82291001	82292000	LOC105377308;VAMP9P
4	82617001	82618000	LINC00575
4	83506001	83507000	RPL30P5
4	86257001	86258000	MAPK10
4	86896001	86897000	C4orf36
4	87047001	87048000	AFF1
4	88212001	88213000	ABCG2;RNU6ATAC31P
4	88266001	88267000	PPM1K;RNU6-112P
4	88345001	88346000	PPM1K-DT
4	88347001	88348000	PPM1K-DT
4	88473001	88474000	LOC102723458;HERC5
4	88491001	88492000	HERC5
4	88826001	88827000	FAM13A
4	90680001	90681000	CCSER1
4	92272001	92273000	LNCPRESS2
4	92441001	92442000	GRID2
4	94840001	94841000	BMPR1B
4	95070001	95071000	BMPR1B
4	98321001	98322000	RAP1GDS1
4	98597001	98598000	TSPAN5
4	102314001	102315000	SLC39A8
4	102614001	102615000	NFKB1;LOC105377347
4	102660001	102661000	MANBA;RPL21P49
4	105592001	105593000	ARHGEF38
4	105824001	105825000	GSTCD;GSTCD-AS1
4	106575001	106576000	LOC105377356
4	107294001	107295000	LOC102725220
4	108397001	108399000	ZACNP1;RPSAP34;ZACNP1;RPSAP34
4	109628001	109629000	MCUB
4	110205001	110206000	ELOVL6;LOC107986303
4	111338001	111339000	RNU6-289P
4	112243001	112244000	AP1AR
4	112934001	112935000	ANK2
4	113253001	113254000	ANK2
4	117602001	117604000	LINC01378;LINC01378
4	118698001	118699000	METTL14
4	118732001	118733000	SEC24D
4	119156001	119157000	MYOZ2;LOC105379404
4	119361001	119362000	KLHL2P1;RNU4-33P
4	120742001	120743000	PRDM5
4	120870001	120871000	PRDM5
4	121706001	121707000	ANXA5
4	122806001	122807000	RPL34P12
4	123054001	123055000	SPATA5
4	127697001	127698000	INTU
4	128474001	128475000	LINC02615
4	134013001	134014000	PABPC4L
4	138929001	138931000	LOC105377448;LOC105377448
4	138957001	138958000	LOC105377448
4	140976001	140977000	RNF150

4	141039001	141040000	RNF150
4	142704001	142705000	INPP4B
4	145699001	145700000	C4orf51
4	145834001	145835000	ZNF827
4	145836001	145837000	ZNF827
4	146058001	146059000	LOC105377468
4	147844001	147845000	ARHGAP10
4	147964001	147965000	ARHGAP10
4	148119001	148120000	NR3C2
4	149381001	149382000	IQCM
4	150096001	150097000	DCLK2
4	151813001	151814000	LOC105377488
4	155217001	155218000	NPY2R;NPY2R-AS1
4	155307001	155308000	LOC105377502
4	155452001	155454000	LOC105377503;MTCYBP17;MTND6P17;MTND5P9;MTND4P8;MTND4LP29;MTND3P3;MTCO3P9;MTATP6P9;MTCO2P9;MTCO1P9;MTND2P33;NMTRQ-TTG15-1;MTND1P22;LOC105377503;MTCYBP17;MTND6P17;MTND5P9;MTND4P8;MTND4LP29;MTND3P3;MTCO3P9;MTATP6P9;MTCO2P9;MTCO1P9;MTND2P33;NMTRQ-TTG15-1;MTND1P22
4	156105001	156106000	LOC102724785
4	156107001	156108000	LOC102724785
4	157179001	157180000	GLRB
4	157266001	157267000	GRIA2
4	159198001	159199000	RAPGEF2
4	162714001	162715000	LOC105377516
4	163093001	163095000	MIR4454;MIR4454
4	163096001	163097000	MIR4454
4	163103001	163104000	MIR4454;NAF1;LOC101928081
4	166045001	166046000	TLL1;RNA5SP170
4	168563001	168564000	PALLD
4	168673001	168674000	PALLD
4	168693001	168694000	PALLD;RNU6-1336P
4	168976001	168977000	CBR4;LOC107986200
4	169053001	169054000	LOC105377527
4	169649001	169650000	CLCN3
4	169903001	169904000	LOC105377530
4	172639001	172640000	GALNTL6;GALNTL6-AS1
4	172956001	172958000	GALNTL6;GALNTL6
4	172959001	172960000	GALNTL6
4	174488001	174489000	HPGD
4	175724001	175725000	GPM6A
4	176639001	176640000	LOC100421630
4	177724001	177725000	LINC01098
4	180203001	180204000	LOC105377567
4	180244001	180245000	LOC105377567
4	181656001	181657000	TENM3
4	181745001	181746000	TENM3
4	182243001	182244000	TENM3
4	182283001	182284000	TENM3
4	182813001	182814000	TENM3
4	182877001	182878000	LOC105377576
4	182933001	182934000	LOC105377577
4	182974001	182975000	LOC107986327
4	183155001	183156000	WWC2
4	183313001	183314000	WWC2;CLDN22;CLDN24
4	183333001	183334000	LOC107986217
4	183535001	183536000	LOC101929996
4	183865001	183866000	STOX2
4	183886001	183887000	STOX2
4	183967001	183968000	STOX2
4	184032001	184034000	STOX2;LOC107986330;STOX2;LOC107986330
4	184152001	184153000	ENPP6
4	184195001	184196000	ENPP6
4	184410001	184411000	IRF2
4	184455001	184456000	IRF2
4	184750001	184751000	ACSL1
4	184996001	184997000	LINC02437
4	185328001	185330000	SNX25;SNX25
4	185532001	185533000	PDLIM3
4	185781001	185782000	SORBS2
4	185813001	185814000	SORBS2
4	185939001	185940000	SORBS2
4	186160001	186161000	FAM149A;RPSAP70;LTO1P1
4	186197001	186198000	FLJ38576;CYP4V2
4	186243001	186244000	KLKB1
4	186247001	186248000	KLKB1
4	186252001	186253000	KLKB1
4	186450001	186451000	F11-AS1
4	186478001	186479000	F11-AS1
4	186491001	186492000	F11-AS1;LOC105377596;RNU6-1055P
4	186631001	186632000	FAT1;LOC107986334
4	187304001	187305000	LOC339975
4	187315001	187316000	LOC339975
4	187345001	187346000	LOC339975
4	187432001	187433000	LOC339975;LINC02515;LOC100652955
4	188432001	188433000	LOC105377609
4	188503001	188504000	LINC01060
4	189483001	189484000	HSP90AA4P

4	189927001	189928000	FRG1-DT
4	190020001	190021000	RNA5SP174;RNA5SP175;DUX4L9;FRG2
4	190177001	190178000	DUX4
4	190179001	190180000	DUX4
4	190182001	190183000	DUX4
5	50001	51000	LOC105374602;LOC100128803
5	97001	98000	PLEKHG4B
5	103001	105000	PLEKHG4B;PLEKHG4B
5	108001	109000	PLEKHG4B
5	317001	318000	PDCD6-AHRR;PDCD6;AHRR
5	352001	353000	PDCD6-AHRR;AHRR
5	390001	391000	PDCD6-AHRR;AHRR
5	394001	395000	PDCD6-AHRR;AHRR;LOC100310782
5	403001	404000	PDCD6-AHRR;AHRR;LOC100310782
5	427001	428000	PDCD6-AHRR;AHRR
5	492001	493000	SLC9A3
5	583001	584000	LOC105374607
5	697001	698000	TPPP;LOC101929898
5	733001	734000	ZDHHC11B
5	857001	858000	ZDHHC11;BRD9
5	1024001	1025000	NKD2
5	1042001	1043000	NKD2;SLC12A7
5	1098001	1099000	SLC12A7
5	1196001	1198000	SLC6A19;SLC6A19
5	1230001	1231000	SLC6A19;SLC6A18
5	1286001	1287000	TERT
5	1433001	1434000	SLC6A3
5	1503001	1504000	LPCAT1;MIR6075
5	1527001	1528000	LPCAT1
5	1807001	1808000	MRPL36;NDUFS6
5	1817001	1818000	NDUFS6
5	1848001	1849000	LINC02116
5	1874001	1875000	IRX4;IRX4-AS1
5	1926001	1927000	LOC112267929;LOC105374618
5	2301001	2302000	LOC100506858
5	2702001	2703000	LOC107986398;IRX2;LSINCT5
5	2717001	2718000	IRX2;LSINCT5
5	3043001	3044000	LOC105374621
5	3420001	3421000	LINC01019
5	3784001	3785000	LOC105374625
5	5040001	5041000	LINC01020
5	5426001	5427000	LOC101929200;ICE1
5	6465001	6466000	UBE2QL1;LOC105374639
5	6467001	6468000	UBE2QL1;LOC105374639
5	6600001	6601000	NSUN2
5	6682001	6683000	SRD5A1;LINC02102
5	6797001	6798000	LINC02236
5	6850001	6851000	LOC105374642;RN7SKP79
5	7061001	7062000	LINC02196
5	7267001	7268000	LOC105374643
5	7288001	7289000	LOC100130063
5	8415001	8416000	LINC02226
5	8615001	8616000	MTND6P2;MTCYBP37
5	8618001	8619000	MTND6P2;MTCYBP37
5	9182001	9183000	SEMA5A
5	9389001	9390000	SEMA5A
5	9391001	9392000	SEMA5A
5	10230001	10231000	ATPSCKMT
5	10331001	10332000	LOC105374651;LOC101929977
5	10459001	10460000	ROPN1L
5	10620001	10621000	ANKRD33B
5	10629001	10630000	ANKRD33B
5	10997001	10998000	LOC105374654;CTNND2
5	11005001	11006000	CTNND2
5	11590001	11591000	CTNND2
5	11817001	11818000	CTNND2
5	12491001	12492000	LOC105374655
5	12664001	12665000	LINC01194
5	12666001	12667000	LINC01194
5	13774001	13775000	DNAH5
5	14398001	14399000	TRIO
5	14419001	14420000	TRIO
5	14438001	14439000	TRIO
5	14678001	14679000	OTULIN
5	16788001	16789000	MYO10
5	17400001	17401000	LINC02217
5	17777001	17778000	LOC105374666
5	18537001	18538000	RN7SL58P
5	18539001	18541000	RN7SL58P;RN7SL58P
5	18543001	18544000	RN7SL58P
5	19637001	19638000	CDH18
5	22096001	22097000	CDH12
5	22098001	22100000	CDH12;CDH12
5	25118001	25119000	LINC02228
5	25910001	25911000	MSNP1
5	29392001	29393000	LINC02064
5	31509001	31510000	DROSHA

5	31761001	31762000	PDZD2
5	32215001	32216000	RPL27P10
5	33985001	33986000	SLC45A2;AMACR;C1QTNF3-AMACR
5	34174001	34175000	C1QTNF3;LOC643373
5	34893001	34894000	TTC23L;RPL21P54
5	34995001	34996000	AGXT2
5	35664001	35665000	SPEF2
5	35798001	35799000	SPEF2;LOC105374724
5	36138001	36139000	LMBRD2;MIR580
5	37754001	37755000	WDR70
5	38467001	38468000	EGFLAM;LOC105374732;LIFR
5	38562001	38563000	LIFR;LIFR-AS1;MIR3650
5	40044001	40045000	LINC00603
5	42489001	42490000	GHR
5	43072001	43073000	LOC648987;LOC100132356;LOC100506639
5	43448001	43449000	TMEM267
5	52924001	52925000	ITGA1
5	53201001	53202000	RPL13AP13
5	53204001	53206000	RPL13AP13;RPL13AP13
5	53617001	53618000	NDUFS4
5	53942001	53944000	ARL15;MIR581;ARL15;MIR581
5	54747001	54748000	LOC102467080;LOC105378969
5	55279001	55280000	DHX29;LOC105378973
5	56942001	56943000	MIER3
5	58481001	58482000	GAPT
5	60127001	60128000	PDE4D
5	60907001	60908000	ERCC8
5	61329001	61330000	LOC105378994;ZSWIM6
5	65047001	65048000	LOC107986419
5	65402001	65403000	ADAMTS6
5	65558001	65559000	CENPK;PPWD1
5	65756001	65757000	NLN
5	69447001	69448000	MARVELD2
5	69998001	69999000	LOC105369228;LOC112267941
5	70518001	70519000	LOC107986356;GUSBP15;LOC340089
5	70780001	70781000	GUSBP16;LOC101929599
5	71584001	71585000	BDP1;MCCC2
5	71597001	71598000	MCCC2
5	71890001	71891000	LOC105379027
5	72195001	72196000	MAP1B
5	73130001	73131000	TMEM171;LOC105379030
5	73720001	73721000	ARHGEF28
5	73737001	73738000	ARHGEF28
5	75613001	75614000	ANKDD1B
5	76359001	76360000	SV2C
5	76492001	76493000	IQGAP2
5	76620001	76621000	IQGAP2;F2RL2
5	76884001	76885000	S100Z
5	77504001	77505000	WDR41
5	77884001	77885000	LOC101929154
5	77899001	77900000	LOC101929154
5	78285001	78286000	AP3B1
5	79329001	79330000	JMY;LOC102724530
5	79856001	79858000	LOC102724557;LOC105379048;LOC105379048
5	80028001	80029000	THBS4;RBX1P2;TRMT112P2
5	80253001	80254000	SERINC5
5	80794001	80795000	MSH3
5	80953001	80954000	RASGRF2-AS1;RASGRF2
5	81356001	81357000	ACOT12
5	81676001	81677000	SSBP2
5	81738001	81739000	SSBP2
5	82975001	82976000	LOC105379051;ST13P12
5	88109001	88110000	LOC105379070
5	88329001	88330000	TMEM161B-DT
5	90201001	90202000	LINC01339
5	90846001	90847000	ADGRV1
5	93922001	93923000	FAM172A
5	94564001	94565000	KIAA0825;MTND5P12;MTND6P3;MTCYBP35
5	95534001	95535000	TTC37
5	95545001	95546000	TTC37;ARSK
5	95590001	95591000	ARSK
5	96050001	96051000	LOC101929710;LOC105379096;MIR583HG
5	96168001	96171000	LOC101929710;MIR583HG;LOC105379675;LOC101929710;MIR583HG;LOC105379675;LOC101929710;MIR583HG;LOC105379675
5	96457001	96458000	LOC101929710;LOC102724070
5	98081001	98082000	LINC01846
5	99014001	99015000	CHD1-DT
5	108993001	108994000	FER
5	109303001	109304000	LOC285638
5	112176001	112177000	EPB41L4A
5	112286001	112287000	EPB41L4A
5	112651001	112652000	LOC102467216
5	115152001	115153000	TRIM36
5	115957001	115958000	LINCADL;LVRN
5	116579001	116580000	SEMA6A;SEMA6A-AS2
5	116801001	116802000	LOC105379133
5	118341001	118342000	LINC02208
5	118949001	118950000	DTWD2

5	118952001	118953000	DTWD2
5	119123001	119124000	DMXL1;SEPTIN7P10;LAMTOR3P2
5	119135001	119136000	DMXL1;SEPTIN7P10;LAMTOR3P2;RNU6-701P
5	119192001	119193000	DMXL1
5	119633001	119634000	FAM170A
5	120699001	120701000	PRR16;PRR16;RNU4-69P
5	122621001	122622000	LINC02201
5	123021001	123022000	SNX24;RN7SL689P;PPIC
5	123166001	123167000	PRDM6
5	124308001	124309000	LINC01170;LOC107986448
5	124967001	124968000	LOC107986391
5	124970001	124971000	LOC107986391
5	126674001	126675000	LOC105379162
5	128523001	128524000	FBN2
5	128732001	128733000	LOC105379168
5	131572001	131574000	RAPGEF6;RAPGEF6
5	131575001	131576000	RAPGEF6
5	131771001	131772000	FNIP1
5	132059001	132060000	LOC105379174;IL3
5	132832001	132833000	SHROOM1;RNU7-15P
5	133218001	133219000	FSTL4;CTB-49A3.2
5	133296001	133297000	FSTL4
5	133338001	133339000	FSTL4
5	133486001	133487000	FSTL4
5	133909001	133910000	WSPAR
5	133925001	133926000	WSPAR;LOC105379182
5	133935001	133936000	LOC105379182
5	135400001	135401000	MACROH2A1
5	135731001	135732000	SLC25A48
5	136090001	136091000	VTRNA2-1
5	136301001	136302000	TRPC7;TRPC7-AS2
5	136997001	136998000	SPOCK1
5	137403001	137404000	SPOCK1
5	138926001	138927000	CTNNA1
5	138935001	138936000	CTNNA1
5	138945001	138946000	CTNNA1;SIL1
5	139044001	139045000	SIL1;RPL12P21
5	139655001	139656000	CXXC5;CXXC5-AS1
5	139765001	139766000	PSD2
5	139815001	139816000	PSD2
5	139825001	139826000	PSD2
5	139885001	139886000	NRG2
5	140014001	140015000	NRG2
5	140516001	140517000	ANKHD1-EIF4EBP3;ANKHD1
5	140544001	140545000	ANKHD1-EIF4EBP3;ANKHD1;EIF4EBP3;SRA1
5	140555001	140556000	ANKHD1-EIF4EBP3;EIF4EBP3;SRA1;APBB3;MIR6831;SLC35A4
5	141676001	141677000	ARAP3
5	141818001	141819000	LOC107986454
5	142161001	142162000	NDFIP1;LOC107986455
5	142200001	142201000	LOC107986455
5	142436001	142437000	SPRY4-AS1
5	142588001	142589000	FGF1
5	142849001	142850000	ARHGAP26;ARHGAP26-AS1
5	142930001	142931000	ARHGAP26
5	145964001	145965000	LOC107986458;SH3RF2
5	147250001	147251000	STK32A
5	147393001	147394000	STK32A;DPYSL3
5	147430001	147431000	DPYSL3
5	147868001	147869000	C5orf46;SCGB3A2
5	147871001	147872000	C5orf46;SCGB3A2
5	147874001	147875000	C5orf46;SCGB3A2
5	150127001	150128000	PDGFRB
5	150131001	150132000	PDGFRB
5	150280001	150281000	CAMK2A
5	150455001	150456000	RPS14
5	150992001	150993000	LOC105378228
5	152658001	152659000	LINC01470
5	153577001	153578000	GRIA1
5	153700001	153701000	GRIA1
5	153971001	153972000	LOC107986464;LOC107983980
5	154402001	154403000	GALNT10;SAP30L-AS1
5	154452001	154453000	SAP30L-AS1;SAP30L
5	154930001	154931000	GEMIN5
5	154935001	154936000	GEMIN5;MRPL22
5	157990001	157991000	MARK2P11;LOC345471
5	159449001	159450000	LINC01845
5	159540001	159541000	LOC105377684
5	160228001	160229000	FABP6
5	160635001	160636000	ATP10B
5	161358001	161359000	GABRB2
5	163334001	163335000	LOC105377700
5	163339001	163340000	LOC105377700
5	164493001	164494000	LOC105377703;LOC102546299
5	166132001	166133000	LOC107986370
5	166222001	166223000	LOC105377705
5	166874001	166875000	LOC105377706
5	167358001	167359000	TENM2

5	167889001	167890000	TENM2
5	168157001	168158000	TENM2;MIR12125
5	168355001	168356000	WWC1
5	168572001	168573000	PANK3
5	169047001	169048000	SLIT3;SLIT3-AS1
5	169901001	169902000	DOCK2;INSYN2B
5	170267001	170268000	LCP2
5	170382001	170383000	KCNIP1;KCNMB1;KCNIP1-OT1
5	170552001	170553000	KCNIP1
5	170637001	170638000	KCNIP1;KCNIP1-AS1
5	170764001	170766000	LOC107986475;LOC107986475
5	171002001	171003000	RANBP17
5	171004001	171005000	RANBP17
5	171369001	171370000	RN7SL339P;RPSAP71;SNORA70J;RPL10P8
5	171435001	171436000	FGF18
5	171437001	171438000	FGF18
5	171448001	171449000	FGF18
5	171739001	171740000	LOC105377724;LOC105377723
5	172090001	172091000	STK10
5	172169001	172170000	STK10
5	172567001	172568000	LINC01944
5	172603001	172604000	LOC105377729
5	172656001	172657000	NEURL1B;LOC100130394;MIR5003
5	172707001	172708000	LOC107986479
5	173167001	173168000	BNIP1
5	173336001	173337000	STC2
5	173449001	173450000	LOC105377732
5	173477001	173478000	LOC105377732
5	173557001	173558000	LOC105377733
5	173563001	173564000	LOC105377733
5	174542001	174543000	LOC105377739
5	175434001	175435000	DRD1
5	175821001	175822000	CPLX2;LOC105377744
5	175823001	175824000	CPLX2;LOC105377744
5	176030001	176031000	THOC3
5	176133001	176134000	FAM153B;LOC107986487;SEPHS1P3;LOC643201
5	176375001	176376000	ARL10;MIR1271;NOP16
5	176562001	176563000	CDHR2
5	176617001	176618000	GPRIN1;SNCB
5	176635001	176636000	SNCB;MIR4281;EIF4E1B
5	176665001	176666000	TSPAN17
5	176746001	176747000	LINC01574
5	176897001	176898000	HK3;UIMC1
5	176921001	176922000	UIMC1
5	176980001	176981000	UIMC1
5	177097001	177098000	FGFR4
5	177218001	177219000	NSD1
5	177380001	177381000	RGS14;SLC34A1
5	177462001	177463000	PRR7;DBN1
5	177490001	177491000	DBN1;PDLIM7
5	177502001	177503000	PDLIM7;DOK3;DDX41
5	177507001	177508000	PDLIM7;DOK3;DDX41
5	177605001	177606000	TMED9;B4GALT7
5	177828001	177829000	LOC107986490;OR1X5P
5	177887001	177888000	LOC728554
5	178089001	178090000	LOC105377754
5	178262001	178263000	COL23A1
5	178305001	178306000	COL23A1
5	178547001	178548000	COL23A1
5	178721001	178722000	ZNF354A
5	178833001	178834000	LOC100129457
5	178843001	178844000	LOC100129457
5	179062001	179063000	ZNF354C
5	179234001	179235000	ADAMTS2
5	179311001	179312000	ADAMTS2
5	179321001	179322000	ADAMTS2
5	179610001	179611000	RUFY1;RUFY1-AS1;HNRNPH1
5	180262001	180263000	MAPK9;LOC100419721
5	180743001	180744000	OR2Y1
5	181233001	181234000	TRIM41;RACK1;SNORD96A;SNORD95
5	181384001	181385000	WBP1LP4
6	89001	90000	WBP1LP12
6	396001	397000	IRF4
6	696001	697000	EXOC2
6	1010001	1011000	LINC01622
6	1688001	1689000	GMDS;LOC107986513
6	1966001	1967000	GMDS
6	2113001	2114000	GMDS
6	2422001	2423000	GMDS-DT
6	2628001	2629000	LINC01600;LINC02521
6	2694001	2695000	MYLK4
6	3132001	3133000	BPHL
6	3170001	3171000	TUBB2BP1
6	3436001	3437000	SLC22A23
6	3606001	3607000	LOC100507336
6	4180001	4181000	LOC102724096;LOC100129052
6	4459001	4460000	LOC107986561;LOC105374894

6	4471001	4472000	LOC107986561;LOC105374894
6	4521001	4522000	LOC105374894
6	4549001	4550000	LOC105374894
6	4558001	4559000	LOC105374894
6	5239001	5240000	LYRM4-AS1;LYRM4
6	5285001	5286000	FARS2
6	5363001	5364000	FARS2
6	5387001	5388000	FARS2
6	5513001	5514000	FARS2
6	5683001	5684000	FARS2;LOC101927950;LOC107986515
6	5989001	5990000	LOC105374898;NRN1
6	6322001	6323000	F13A1
6	6792001	6793000	LOC101928004;BTF3P7
6	6929001	6930000	RN7SL554P
6	7098001	7099000	RREB1
6	7272001	7273000	SSR1
6	7282001	7283000	SSR1
6	7448001	7449000	LOC102724234
6	7500001	7501000	RPS26P29
6	7782001	7783000	BMP6
6	7868001	7869000	BMP6
6	10339001	10341000	LOC105374920;LOC105374921;LOC105374920;LOC105374921
6	10561001	10562000	GCNT2
6	10568001	10569000	GCNT2;RPL21P63
6	10753001	10754000	TMEM14B;RNA5SP203;MAK
6	10771001	10772000	MAK
6	10778001	10779000	MAK
6	10824001	10825000	MAK
6	10970001	10971000	SYCP2L;ELOVL2
6	11228001	11229000	NEDD9
6	11282001	11283000	NEDD9;LOC105374925
6	11321001	11322000	NEDD9;LOC105374925
6	12114001	12115000	HIVEP1
6	12253001	12254000	EDN1
6	12898001	12899000	PHACTR1
6	13058001	13059000	PHACTR1
6	13201001	13202000	PHACTR1
6	13354001	13355000	LOC105374936;GFOD1
6	13572001	13573000	LOC105374937;SIRT5
6	13649001	13650000	RANBP9
6	14571001	14572000	LOC101928354
6	14676001	14678000	LOC101928354;LOC101928354
6	14869001	14870000	LOC105374944;LOC105374945
6	15185001	15186000	LOC105374946
6	16264001	16265000	GMPR
6	16315001	16316000	ATXN1
6	16588001	16589000	ATXN1
6	17494001	17495000	CAP2;LOC101928491
6	18214001	18215000	KDM1B;DEK
6	18231001	18232000	KDM1B;DEK
6	18448001	18449000	RNF144B
6	18754001	18755000	LOC105374957
6	18756001	18757000	LOC105374957
6	19337001	19338000	LOC107986575
6	19698001	19699000	LOC105374959
6	19845001	19846000	ID4
6	20206001	20207000	MBOAT1
6	20274001	20275000	LOC101928573
6	20672001	20673000	CDKAL1
6	21042001	21043000	CDKAL1
6	21102001	21103000	CDKAL1
6	24505001	24506000	GPLD1;ALDH5A1
6	24549001	24550000	KIAA0319
6	24587001	24588000	KIAA0319;KRT8P43
6	26382001	26383000	BTN3A2;BTN2A2
6	26552001	26553000	HMGN4;TRA-CGC1-1;TRI-AAT5-1;TRP-AGG2-2;TRK-CTT2-4
6	26808001	26809000	LOC105374992;TRM-CAT5-2
6	29250001	29251000	LOC101929006
6	29427001	29428000	OR5V1;OR12D1;OR11A1
6	29773001	29774000	LOC353010
6	30085001	30086000	RNF39
6	30248001	30249000	HCG17;TRIM26BP
6	30633001	30634000	MRPS18B;ATAT1;PTMAP1
6	30737001	30738000	FLOT1;IER3-AS1;IER3
6	31012001	31013000	MUC22
6	31031001	31033000	MUC22;MUC22
6	31352001	31353000	HLA-B;MIR6891
6	31499001	31500000	MICB-DT;MICB
6	31535001	31536000	RPL15P4;MCCD1;ATP6V1G2-DDX39B;DDX39B;SNORD117;SNORD84;DDX39B-AS1;ATP6V1G2
6	31562001	31563000	NFKBIL1;LOC100287329;LTA
6	31572001	31573000	LOC100287329;LTA;TNF;LTB
6	31574001	31575000	LOC100287329;LTA;TNF;LTB
6	31604001	31605000	UQCRHP1
6	31881001	31882000	SLC44A4;EHMT2;LOC107986588
6	32287001	32288000	TSBP1-AS1;TSBP1
6	32293001	32294000	TSBP1-AS1;TSBP1
6	32707001	32708000	MTCO3P1

6	32936001	32937000	HLA-DMB
6	32954001	32955000	HLA-DMA
6	33081001	33082000	HLA-DPA1;HLA-DPB1;RPL32P1;HLA-DPA2
6	33160001	33161000	COL11A2
6	33185001	33186000	COL11A2;RXRB
6	33212001	33213000	SLC39A7;HSD17B8;MIR219A1;RING1;ZNF70P1
6	33595001	33596000	GGNBP1;LINC00336
6	33639001	33640000	ITPR3;LOC101929188;LOC105375023
6	33815001	33816000	LOC105375025
6	33826001	33827000	LOC105375025
6	33900001	33901000	LINC01016;LOC105375026;MIR7159;LOC107986590
6	34022001	34023000	GRM4
6	34077001	34079000	GRM4;GRM4
6	34140001	34141000	GRM4
6	34375001	34376000	NUDT3;RPS10-NUDT3
6	34517001	34518000	PACSIN1
6	34785001	34786000	UHRF1BP1
6	35309001	35310000	DEF6
6	36246001	36248000	LOC105375036;PNPLA1;LOC105375036;PNPLA1
6	36292001	36293000	PNPLA1
6	36698001	36699000	RAB44
6	36746001	36747000	GPR166P;CPNE5
6	36929001	36930000	C6orf89
6	36943001	36944000	PI16
6	37358001	37359000	RNF8;RN7SL273P
6	37511001	37512000	LINC02520
6	37564001	37565000	MIR4462
6	38187001	38188000	BTBD9
6	38614001	38615000	BTBD9
6	38835001	38836000	DNAH8
6	39074001	39075000	GLP1R;LOC105375046;MIR9983
6	39116001	39117000	SAYSD1;ANKRD18EP
6	39122001	39123000	SAYSD1;ANKRD18EP
6	39841001	39842000	DAAM2
6	40897001	40898000	LOC101929555
6	40956001	40957000	LOC101929555
6	41433001	41434000	LOC107986538
6	41645001	41646000	MDFI
6	41658001	41659000	MDFI;NPM1P51
6	41691001	41692000	TFEB
6	42198001	42199000	GUCA1B;MRPS10
6	42287001	42288000	TRERF1
6	42796001	42797000	BICRAL;LOC401261
6	42981001	42982000	PEX6;MEA1;PPP2R5D
6	43219001	43220000	CUL9;DNPH1
6	43447001	43448000	ABCC10;DLK2;LOC107986597
6	43717001	43718000	POLR1C
6	43802001	43803000	POLR1C;LOC105375070
6	44026001	44027000	POLR1C;SCIRT
6	44152001	44153000	POLR1C;TMEM63B;LOC107986599;CAPN11
6	44182001	44183000	POLR1C;CAPN11;LOC105375072;MYMX
6	46255001	46256000	LOC105375079;RCAN2
6	47317001	47318000	TNFRSF21
6	47363001	47364000	B3GNTL1P2
6	51939001	51940000	PKHD1
6	51942001	51943000	PKHD1
6	51948001	51949000	PKHD1
6	52267001	52268000	MCM3
6	52547001	52548000	TRAM2;LOC107986603
6	53035001	53036000	CILK1
6	53172001	53173000	GCM1
6	53237001	53238000	GCM1;HMGB1P20
6	53384001	53385000	RPA3P2
6	53864001	53865000	LRRC1
6	54306001	54307000	LOC105375098;TINAG
6	55198001	55199000	HCRTR2
6	56012001	56015000	LOC105375100;LOC105375100;LOC105375100
6	56362001	56363000	COL21A1
6	56997001	56998000	BEND6;FTH1P15
6	57918001	57919000	GUSBP4;LINC00680-GUSBP4
6	62280001	62281000	KHDRBS2
6	63950001	63952000	EYS;LOC107986608;EYS;LOC107986608
6	63955001	63956000	EYS;LOC107986608
6	70288001	70289000	COL9A1
6	70302001	70303000	COL9A1
6	70770001	70771000	SMAP1
6	70880001	70881000	B3GAT2;LOC105377850
6	71880001	71881000	RIMS1
6	71919001	71920000	RIMS1
6	72897001	72898000	KCNQ5
6	72935001	72936000	KCNQ5;LOC105377855
6	73306001	73307000	KHDC1
6	73765001	73766000	CD109
6	77250001	77251000	LOC101928570
6	77744001	77745000	MEI4
6	79036001	79037000	PHIP
6	79657001	79658000	SH3BGRL2

6	79694001	79695000	SH3BGRL2
6	80484001	80485000	LOC112267962
6	80487001	80488000	LOC112267962
6	80512001	80513000	LOC112267962
6	80515001	80516000	LOC112267962
6	80650001	80652000	LOC112267962;LOC112267962
6	81469001	81470000	LOC105377871
6	82023001	82024000	LINC02542;LOC107986617
6	82071001	82072000	LINC02542;LOC107986617
6	84058001	84059000	MRAP2
6	89161001	89162000	PM20D2
6	89285001	89286000	GABRR2
6	89504001	89505000	ANKRD6;LOC105377889
6	89836001	89837000	MDN1;CASP8AP2
6	90002001	90003000	BACH2;RN7SKP110
6	90029001	90030000	BACH2
6	90044001	90045000	BACH2
6	98901001	98902000	FBXL4
6	99400001	99401000	COQ3;PNISR
6	100412001	100413000	SIM1;SIM1-AS1
6	101114001	101115000	LOC107984041
6	101856001	101857000	GRIK2
6	104458001	104459000	LOC105377918
6	104844001	104845000	HACE1
6	105878001	105879000	LOC105377923
6	106533001	106534000	CRYBG1
6	106716001	106717000	LOC105377927;LINC02532
6	106946001	106947000	LOC107986534
6	107026001	107027000	MTRES1
6	107859001	107860000	SEC63
6	109237001	109238000	CCDC162P
6	109288001	109289000	CCDC162P;PTCHD3P3
6	109325001	109326000	CCDC162P;RPL7P28;LOC101927538
6	111003001	111004000	RPF2;RNU6-906P
6	111183001	111184000	SLC16A10
6	111306001	111307000	REV3L
6	111867001	111868000	FYN;LOC102724646
6	112065001	112066000	CCN6;TUBE1
6	112171001	112172000	LAMA4
6	113578001	113579000	RPS27AP11
6	114013001	114014000	HDAC2-AS2;NUDT19P3
6	114015001	114016000	HDAC2-AS2;NUDT19P3
6	114426001	114427000	LOC107986638
6	116303001	116304000	DSE
6	119770001	119771000	LOC105377975
6	121834001	121835000	LOC105377979
6	122541001	122542000	PKIB
6	123473001	123474000	TRDN;TRDN-AS1
6	123557001	123558000	TRDN;LOC105377982
6	123582001	123584000	TRDN;TRDN
6	123585001	123586000	TRDN
6	123587001	123588000	TRDN
6	125002001	125003000	RNF217
6	125343001	125344000	LOC107986640
6	125778001	125779000	TRE-CTC1-7;NCOA7
6	127722001	127723000	THEMIS
6	128258001	128259000	PTPRK
6	128433001	128434000	PTPRK
6	128910001	128911000	LAMA2
6	128998001	128999000	LAMA2
6	130054001	130055000	L3MBTL3
6	130064001	130065000	L3MBTL3
6	130197001	130198000	SAMD3
6	131123001	131124000	LOC102723445;AKAP7
6	131296001	131297000	LOC107986643
6	131343001	131344000	LOC105378005
6	131404001	131405000	LOC105378005
6	131407001	131408000	LOC105378005
6	131678001	131679000	ENPP3
6	131905001	131906000	ENPP1
6	133992001	133993000	TBPL1;SLC2A12
6	133994001	133995000	TBPL1;SLC2A12
6	135287001	135288000	AHI1
6	135290001	135291000	AHI1
6	135956001	135957000	PDE7B
6	136063001	136064000	PDE7B;LOC644135
6	136552001	136553000	MAP7;RN7SKP299;MAP3K5
6	136802001	136803000	MAP3K5
6	136969001	136970000	RPL35AP3
6	137536001	137537000	BTF3L4P3
6	137846001	137847000	WAKMAR2;LOC107986649
6	138980001	138981000	REPS1
6	141918001	141919000	LOC105378031
6	142017001	142018000	LOC105378031
6	142019001	142020000	LOC105378031
6	142829001	142830000	HIVEP2
6	143155001	143156000	AIG1

6	143790001	143791000	PHACTR2
6	143955001	143956000	PLAGL1
6	144406001	144407000	UTRN;LOC100420214;SNORA98
6	145372001	145373000	EPM2A
6	145634001	145635000	EPM2A
6	145646001	145647000	EPM2A
6	147631001	147633000	SAMD5;SAMD5
6	147945001	147946000	SAMD5
6	148219001	148220000	SASH1
6	148413001	148414000	SASH1
6	148507001	148508000	SASH1
6	148556001	148557000	SASH1
6	148797001	148799000	UST;UST
6	148985001	148986000	UST
6	149032001	149033000	UST;UST-AS2
6	149214001	149215000	TAB2
6	149378001	149379000	TAB2
6	150052001	150053000	PHBP1;ULBP3
6	150054001	150055000	PHBP1;ULBP3
6	150056001	150057000	ULBP3
6	150168001	150169000	PPP1R14C
6	150190001	150191000	PPP1R14C
6	150201001	150202000	PPP1R14C
6	150333001	150334000	RNU4-7P
6	150640001	150641000	PLEKHG1
6	150754001	150755000	PLEKHG1
6	150812001	150813000	PLEKHG1
6	151320001	151321000	AKAP12;RN7SKP268
6	151448001	151449000	RMND1;ARMT1
6	151587001	151589000	CCDC170;CCDC170
6	151669001	151670000	ESR1
6	151803001	151804000	ESR1
6	151876001	151877000	ESR1
6	151961001	151962000	ESR1
6	152263001	152264000	SYNE1
6	152494001	152496000	SYNE1;SYNE1
6	154501001	154502000	CNKSR3;LOC101928868
6	154553001	154554000	MTRES1P1
6	154859001	154860000	MIR1273C
6	155113001	155114000	TIAM2
6	155155001	155156000	TIAM2
6	156914001	156915000	ARID1B
6	157131001	157132000	ARID1B
6	157427001	157428000	ZDHHC14
6	157681001	157682000	ZDHHC14
6	157702001	157703000	LOC107986663
6	157704001	157706000	LOC107986663;LOC107986663
6	157892001	157893000	SNX9;SNX9-AS1
6	157974001	157975000	SYNJ2
6	157977001	157978000	SYNJ2
6	158006001	158007000	SYNJ2;SYNJ2-IT1
6	158171001	158172000	SERAC1;GTF2H5
6	158524001	158525000	CACYBPP3
6	158602001	158603000	TMEM181;TATDN2P2;MIR7161
6	158627001	158628000	TMEM181;DYNLT1
6	158705001	158706000	SYTL3
6	158853001	158854000	OSTCP1
6	158880001	158882000	LINC02901;LINC02901
6	158885001	158887000	LINC02901;LINC02901
6	158888001	158889000	LINC02901
6	159005001	159006000	RSPH3;TAGAP-AS1
6	159012001	159013000	TAGAP-AS1
6	159819001	159820000	PNLDC1
6	159903001	159904000	MAS1
6	160008001	160009000	IGF2R;AIRN
6	160084001	160085000	IGF2R;CHP1P2
6	160131001	160132000	SLC22A1
6	160229001	160230000	SLC22A2
6	160233001	160234000	SLC22A2
6	160841001	160842000	LOC107986665
6	160847001	160848000	LOC107986665
6	160850001	160851000	LOC107986665
6	160852001	160853000	LOC107986665
6	160856001	160857000	LOC107986665
6	161082001	161083000	MAP3K4
6	161177001	161178000	AGPAT4
6	161356001	161357000	PRKN
6	161514001	161515000	PRKN
6	161550001	161551000	PRKN
6	161564001	161566000	PRKN;PRKN
6	162133001	162134000	PRKN
6	162543001	162544000	PRKN
6	162843001	162844000	PACRG
6	162846001	162847000	PACRG
6	163021001	163022000	PACRG
6	163196001	163197000	PACRG;PACRG-AS3;LOC105378095
6	163312001	163313000	PACRG;PACRG-AS1

6	163538001	163539000	QKI
6	163658001	163659000	LOC102724152
6	163713001	163714000	LOC107986666;LOC105378104
6	164017001	164018000	LOC105378102
6	164414001	164415000	LOC105379712
6	164438001	164439000	LOC107986667
6	165275001	165276000	C6orf118
6	165389001	165390000	PDE10A
6	165861001	165862000	PDE10A
6	166088001	166089000	LOC729681
6	166105001	166106000	LOC729681
6	166236001	166237000	LOC101929297;GNG5P1
6	166240001	166241000	LOC101929297;GNG5P1
6	166339001	166340000	SFT2D1;HNRNPA1P49;LOC100289495
6	166748001	166750000	RPS6KA2;RPS6KA2
6	166931001	166932000	RNASET2
6	167509001	167510000	LOC107986546
6	167552001	167553000	LOC105378129
6	167554001	167555000	LOC105378129
6	167790001	167791000	LINC01558;LOC441179
6	167882001	167883000	AFDN
6	167999001	168000000	KIF25-AS1;KIF25
6	169291001	169292000	VTA1P1
6	169376001	169377000	LINC02519
6	169808001	169809000	LINC00242;LINC00574
6	169891001	169892000	LOC105378149
6	170018001	170019000	LOC105378150
6	170172001	170173000	LOC102724511
6	170376001	170377000	FAM120B
6	170493001	170494000	LOC105378157
6	170501001	170502000	LOC105378157
6	170656001	170657000	LOC101929692;WBP1LP8
6	170658001	170659000	LOC101929692;WBP1LP8
6	170660001	170661000	LOC101929692;WBP1LP8
7	16001	17000	LOC102723872
7	106001	107000	LOC105375114
7	188001	190000	LOC105375116;FAM20C;LOC105375116;FAM20C
7	202001	203000	FAM20C
7	215001	216000	FAM20C
7	603001	604000	PRKAR1B;PRKAR1B-AS1;LOC105375119
7	611001	612000	PRKAR1B;PRKAR1B-AS1;LOC105375119
7	661001	662000	PRKAR1B
7	736001	737000	PRKAR1B;DNAAF5
7	787001	788000	DNAAF5
7	919001	920000	ADAP1
7	1001001	1002000	C7orf50
7	1011001	1012000	C7orf50
7	1059001	1060000	C7orf50;GPR146;LOC107986755
7	1061001	1062000	C7orf50;GPR146;LOC107986755
7	1642001	1644000	LOC107986757;LOC107986757
7	1711001	1712000	ELFN1;LOC105375124
7	1932001	1933000	MAD1L1
7	2612001	2613000	IQCE;LOC107986760
7	2793001	2794000	GNA12
7	2945001	2946000	CARD11;CARD11-AS1
7	3008001	3009000	CARD11;RN7SKP130
7	3099001	3100000	LOC105375130
7	3137001	3138000	LOC100129603
7	3269001	3270000	SDK1-AS1
7	3361001	3362000	SDK1
7	3381001	3382000	SDK1
7	3414001	3415000	SDK1
7	3474001	3475000	SDK1;LOC100421742
7	3652001	3653000	SDK1
7	3695001	3696000	SDK1
7	3743001	3744000	SDK1
7	3920001	3921000	SDK1
7	3936001	3937000	SDK1
7	3941001	3942000	SDK1
7	4052001	4053000	SDK1
7	4157001	4158000	SDK1
7	4240001	4241000	SDK1
7	4262001	4263000	SDK1
7	5034001	5035000	SPDYE19P;LOC105375134
7	5076001	5077000	RBAK-RBAKDN;RBAK;RBAKDN
7	5308001	5309000	SLC29A4;TNRC18
7	5431001	5432000	TNRC18;LOC100129484
7	5492001	5493000	FBXL18;MIR589
7	5974001	5975000	RSPH10B;PMS2
7	6093001	6094000	USP42
7	6750001	6751000	PMS2CL;RSPH10B2
7	6790001	6791000	RSPH10B2;CCZ1B;LOC107986696
7	6904001	6905000	LOC100420863
7	6907001	6908000	LOC100420863
7	6948001	6949000	FAM86LP;LOC112267992
7	7466001	7467000	COL28A1
7	7770001	7771000	UMAD1

7	8302001	8303000	LOC100505938
7	10606001	10607000	MGC4859
7	12590001	12591000	LOC107986769;SCIN;LOC107986768
7	12660001	12661000	SCIN
7	12663001	12664000	SCIN
7	14886001	14887000	DGKB
7	16799001	16800000	AGR2
7	18196001	18197000	HDAC9
7	18662001	18663000	HDAC9
7	18793001	18794000	HDAC9
7	19155001	19156000	FERD3L
7	20122001	20125000	LOC101927668;LOC105375181;LOC101927668;LOC105375181;LOC101927668;LOC105375181;MACC1
7	21593001	21594000	DNAH11
7	22883001	22884000	LOC107986776;RPL12P10
7	22915001	22916000	FAM126A
7	22925001	22926000	FAM126A
7	23322001	23323000	IGF2BP3
7	24025001	24026000	LOC105375188
7	24030001	24031000	LOC105375188
7	24401001	24402000	LOC107986777
7	25483001	25484000	LOC105375196
7	25745001	25746000	LOC646588
7	26062001	26063000	LOC105375199
7	26084001	26085000	LOC105375199
7	26330001	26331000	SNX10
7	26467001	26468000	LOC441204
7	26503001	26504000	LOC441204
7	26631001	26632000	LOC101928077;LINC02860
7	27802001	27803000	TAX1BP1
7	28103001	28104000	JAZF1
7	28119001	28120000	JAZF1;RNU6-979P
7	28474001	28475000	CREB5
7	28827001	28828000	CREB5
7	29000001	29001000	LOC100506497;CPVL
7	29813001	29814000	WIPF3
7	30773001	30774000	INMT-MINDY4;MINDY4
7	30802001	30803000	INMT-MINDY4;MINDY4
7	30986001	30987000	GHRHR;LOC105375222;LOC105375221
7	31144001	31145000	LOC107986781
7	32212001	32213000	PDE1C
7	32380001	32381000	PDE1C
7	32511001	32512000	AVL9
7	32687001	32688000	DPY19L1P1
7	32834001	32835000	DPY19L1P2
7	32996001	32997000	FKBP9;RNU6-388P
7	33010001	33011000	FKBP9;RNU6-388P;NT5C3A
7	33526001	33527000	BBS9
7	33914001	33915000	BMPER
7	34302001	34304000	RNU6-438P;RNU6-438P
7	35118001	35119000	LOC105375228;DPY19L2P1
7	35603001	35604000	LOC105375231;LOC101928421
7	37063001	37064000	ELMO1
7	37175001	37176000	ELMO1
7	37923001	37924000	SFRP4;EPDR1
7	38211001	38212000	STARD3NL
7	40244001	40245000	SUGCT;LOC105375245
7	40865001	40866000	SUGCT;LOC105375242
7	42335001	42336000	LOC105375249
7	42686001	42687000	LINC01448
7	42871001	42873000	LOC107986735;LOC107986735
7	43494001	43495000	HECW1
7	43504001	43505000	HECW1;LUARIS
7	43529001	43530000	HECW1;LUARIS
7	44868001	44869000	PURB
7	45660001	45662000	ADCY1;ADCY1
7	45939001	45940000	LOC102723446
7	47330001	47331000	TNS3
7	47548001	47549000	TNS3
7	47864001	47865000	PKD1L1
7	48046001	48047000	C7orf57
7	48463001	48464000	ABCA13
7	48626001	48627000	ABCA13
7	50349001	50350000	IKZF1
7	50566001	50567000	DDC
7	50648001	50649000	GRB10
7	50671001	50672000	GRB10
7	50748001	50749000	GRB10
7	52333001	52334000	LOC107986796
7	52335001	52336000	LOC107986796
7	54663001	54664000	RPL31P35
7	54787001	54788000	SEC61G-DT
7	55439001	55440000	LANCL2;VOPP1
7	55561001	55562000	VOPP1
7	55963001	55964000	MRPS17;NIPSNAP2
7	56344001	56345000	RNU6-1335P
7	57008001	57009000	TNRC18P3;SLC29A4P1

7	57186001	57187000	GUSBP10;MTND1P4;NMTRQ-TTG13-1;MTND2P6;MTCO1P10;MTCO2P10;MTATP6P10;MTCO3P4;MTND4LP4;MTND4P5;MTND5P7;MTCYBP5
7	57217001	57218000	LOC105375297;RNU7-157P
7	57219001	57221000	LOC105375297;RNU7-157P;LOC105375297;RNU7-157P
7	63627001	63628000	MIR4283-2
7	64104001	64105000	GUSBP6;MTND4P2;MTND4LP2;MTND3P2;MTCO3P8;MTATP6P18;MTCO2P8;MTCO1P8;MTND2P4;MTND1P2
7	64107001	64108000	GUSBP6;MTND4P2;MTND4LP2;MTND3P2;MTCO3P8;MTATP6P18;MTCO2P8;MTCO1P8;MTND2P4;MTND1P2
7	64112001	64113000	GUSBP6;MTND4P2;MTND4LP2;MTND3P2;MTCO3P8;MTATP6P18;MTCO2P8;MTCO1P8;MTND2P4;MTND1P2
7	64682001	64683000	ZNF107;BNIP3P11;MIR6839
7	64685001	64686000	ZNF107;BNIP3P11;MIR6839
7	64838001	64839000	ZNF138
7	65722001	65723000	LOC441242;INTS4P2
7	65841001	65842000	LOC84214
7	65963001	65964000	VKORC1L1;GUSB
7	66732001	66733000	RABGEF1
7	66905001	66906000	LOC107983971;LINC02604;RPL31P38
7	67279001	67280000	PMS2P4;SPDYE21
7	67283001	67284000	PMS2P4;SPDYE21
7	69333001	69334000	MTCO2P25;MTCO1P25
7	70387001	70388000	AUTS2
7	71161001	71162000	GALNT17
7	71382001	71383000	GALNT17
7	71950001	71951000	CALN1;RPS28P6
7	72183001	72184000	CALN1
7	72501001	72502000	CALN1
7	73052001	73053000	SPDYE11
7	73299001	73300000	POM121B;NSUN5
7	73600001	73601000	MLXIPL
7	73689001	73690000	DNAJC30;BUD23;STX1A
7	74221001	74222000	LAT2;RFC2
7	74250001	74251000	RFC2
7	74528001	74529000	GTF2IRD1;WBSCR23
7	74568001	74569000	GTF2IRD1
7	74909001	74910000	PMS2P5;SPDYE12P
7	74912001	74913000	PMS2P5;SPDYE12P
7	75088001	75089000	GTF2IRD2B
7	75312001	75313000	SPDYE14
7	75495001	75496000	POM121C;SPDYE5
7	75500001	75501000	SPDYE5;PMS2P3
7	75584001	75585000	HIP1
7	75605001	75606000	HIP1
7	75662001	75663000	HIP1
7	75741001	75742000	HIP1
7	75789001	75790000	CCL26
7	75990001	75991000	POR;TMEM120A;STYXL1
7	76045001	76046000	STYXL1;MDH2
7	76049001	76050000	STYXL1;MDH2
7	76498001	76499000	DTX2
7	76565001	76566000	LOC100133091
7	77341001	77342000	GSAP
7	77343001	77344000	GSAP
7	77345001	77346000	GSAP
7	78035001	78036000	MAGI2
7	78598001	78599000	MAGI2
7	79323001	79324000	MAGI2
7	80814001	80815000	SEMA3C
7	83370001	83371000	SEMA3E
7	83464001	83465000	SEMA3E;LOC105375378
7	83479001	83480000	SEMA3E;LOC105375378
7	87041001	87042000	ELAPOR2
7	89336001	89337000	ZNF804B
7	90352001	90353000	LOC107986715;LOC101927446;GTPBP10
7	91117001	91118000	CDK14;PTP4A1P3
7	91862001	91863000	MTERF1
7	92423001	92424000	TMBIM7P
7	92484001	92485000	GATAD1;ERVW-1;PEX1
7	92629001	92630000	CDK6;LOC112268009
7	93362001	93363000	VPS50
7	95514001	95515000	ASB4
7	95995001	95996000	DYNC1I1
7	96260001	96261000	SLC25A13
7	96797001	96798000	LOC105375414
7	98168001	98169000	LMTK2
7	98243001	98244000	TECPR1
7	98860001	98861000	TMEM130
7	99401001	99402000	ARPC1B;PDAP1;BUD31;MIR12119
7	100131001	100132000	TAF6;CNPY4;MBLAC1;RPL7P60;LAMTOR4
7	100308001	100309000	SPDYE3
7	100375001	100376000	STAG3L5P-PVRIG2P-PILRB;PILRB;PILRA
7	100384001	100385000	PILRA
7	100484001	100485000	TSC22D4;NYAP1
7	100734001	100735000	ZAN
7	100751001	100753000	ZAN;ZAN
7	100804001	100805000	ZAN;EPHB4
7	100840001	100841000	SLC12A9;SLC12A9-AS1
7	100923001	100924000	RPS29P15
7	100927001	100928000	RPS29P15

7	100954001	100957000	LOC105375431;MUC3A;LOC105375431;MUC3A;LOC105375431;MUC3A
7	100960001	100961000	LOC105375431;MUC3A;MUC12
7	101091001	101092000	TRIM56
7	101135001	101136000	SERPINE1
7	101143001	101144000	SERPINE1
7	101996001	101997000	CUX1
7	102132001	102133000	CUX1
7	102191001	102192000	CUX1
7	102268001	102269000	CUX1
7	102355001	102356000	SPDYE6;LOC100630923;LOC100289561
7	102429001	102430000	LOC100630923;PRKRIP1;LOC105375433;ORAI2
7	102514001	102515000	RASA4B
7	102521001	102522000	RASA4B
7	102554001	102555000	POLR2J3;UPK3BL2;SPDYE2
7	102620001	102621000	RASA4
7	102871001	102872000	FBXL13
7	103153001	103154000	NAPEPLD;LOC105375434;RPL23AP95
7	105112001	105113000	KMT2E;SRPK2
7	105278001	105279000	SRPK2
7	105659001	105661000	ATXN7L1;ATXN7L1
7	105810001	105811000	ATXN7L1;RPL13AP16
7	106567001	106568000	CTB-30L5.1
7	106606001	106607000	CTB-30L5.1
7	106829001	106830000	LINC02577
7	107519001	107520000	COG5
7	108164001	108165000	NRCAM;LOC102724363
7	108275001	108276000	NRCAM;RNU7-83P
7	111757001	111758000	DOCK4
7	111781001	111782000	DOCK4
7	111850001	111851000	DOCK4
7	112013001	112014000	DOCK4
7	112375001	112376000	LOC105375456;MTND5P8;MTND6P24;MTCYBP24
7	112617001	112618000	LOC100996249;LOC101928012
7	114447001	114448000	FOXP2
7	116565001	116566000	CAV1;COMETT
7	117222001	117224000	ST7;LOC105375466;ST7;LOC105375466
7	118224001	118225000	ANKRD7
7	120010001	120014000	RNU1-29P;RNU1-29P;RNU1-29P;RNU1-29P
7	120778001	120779000	TSPAN12
7	120960001	120961000	ING3
7	122818001	122820000	CADPS2;CADPS2
7	124961001	124963000	POT1-AS1;POT1-AS1
7	125180001	125181000	LOC101928283
7	125262001	125263000	LOC101928283;LOC101928254
7	127259001	127260000	GRM8
7	128120001	128121000	LOC105375493
7	128201001	128202000	MIR129-1
7	128596001	128597000	LOC101928451
7	128726001	128727000	FAM71F1
7	128905001	128906000	KCP;LOC105375497;LOC392787
7	129124001	129125000	CYCSP20;LOC407835
7	129837001	129838000	UBE2H
7	130073001	130074000	KLHDC10
7	130163001	130164000	TMEM209
7	130283001	130284000	CPA2;CPA4
7	130317001	130318000	CPA4;LOC105375503
7	130485001	130486000	MESTIT1;MEST
7	130522001	130523000	COPG2
7	130524001	130525000	COPG2
7	130527001	130528000	COPG2
7	130532001	130533000	COPG2
7	130534001	130535000	COPG2;COPG2IT1
7	130536001	130537000	COPG2;COPG2IT1
7	130538001	130539000	COPG2;COPG2IT1
7	131018001	131019000	LINC-PINT
7	131079001	131080000	LINC-PINT
7	131562001	131563000	PODXL
7	132083001	132084000	LOC100533635
7	132615001	132616000	PLXNA4
7	132633001	132634000	PLXNA4
7	132647001	132648000	PLXNA4;FLJ40288
7	132884001	132885000	CHCHD3
7	133442001	133443000	EXOC4
7	133894001	133895000	EXOC4
7	133904001	133905000	EXOC4
7	135242001	135243000	STRA8
7	137546001	137547000	DGKI
7	137678001	137679000	DGKI
7	137827001	137828000	DGKI
7	137969001	137970000	CREB3L2
7	138618001	138619000	SVOPL
7	138914001	138915000	KIAA1549
7	138937001	138938000	KIAA1549
7	139283001	139284000	UBN2
7	139461001	139463000	KLRG2;KLRG2
7	139466001	139468000	KLRG2;KLRG2
7	139822001	139823000	TBXAS1

7	139873001	139875000	TBXAS1;LOC107986853;TBXAS1;LOC107986853
7	139929001	139930000	TBXAS1;LOC105375532
7	139952001	139953000	TBXAS1;LOC105375532
7	140600001	140601000	DENND2A
7	140655001	140656000	LOC105375535;RN7SL771P;LOC107986718
7	140698001	140699000	ADCK2;NDUFB2-AS1;NDUFB2
7	140760001	140761000	BRAF
7	141108001	141109000	TMEM178B
7	141246001	141247000	TMEM178B
7	141278001	141279000	TMEM178B
7	141336001	141337000	TMEM178B
7	141563001	141564000	AGK
7	141579001	141580000	AGK
7	142829001	142830000	LOC105375541
7	142954001	142955000	KEL
7	143064001	143065000	OR6W1P
7	143283001	143284000	TMEM139-AS1;TMEM139;CASP2;RN7SL535P
7	143329001	143330000	CLCN1
7	143374001	143375000	FAM131B;FAM131B-AS1;LOC100507507;ZYX;MIR6892
7	143384001	143385000	FAM131B;LOC100507507;ZYX;MIR6892;EPHA1
7	143419001	143420000	EPHA1-AS1
7	144208001	144209000	ARHGEF35-AS1
7	144342001	144343000	OR2A1-AS1
7	144364001	144367000	OR2A1-AS1;ARHGEF5;OR2A1-AS1;ARHGEF5;ARHGEF5
7	144442001	144443000	PPIAP83;RNU6ATAC40P;TPK1
7	146247001	146248000	CNTNAP2
7	146585001	146586000	CNTNAP2;LOC100420074
7	147287001	147288000	CNTNAP2
7	148159001	148160000	CNTNAP2
7	148209001	148210000	CNTNAP2
7	148312001	148313000	CNTNAP2
7	148330001	148331000	CNTNAP2
7	148972001	148973000	RNY4
7	149201001	149202000	ZNF282
7	149204001	149205000	ZNF282
7	149616001	149617000	ZNF767P;TRS-AGA5-1;TRC-GCA19-1
7	149804001	149805000	SSPOP
7	149895001	149896000	ACTR3C
7	149972001	149973000	ACTR3C
7	149990001	149991000	ACTR3C
7	149996001	149997000	ACTR3C
7	150308001	150309000	ACTR3C;LRRC61
7	150310001	150312000	ACTR3C;LRRC61;ACTR3C;LRRC61
7	150396001	150397000	ZNF775;LOC107986859;LOC728743
7	150450001	150451000	LINC00996;GIMAP8
7	150687001	150688000	EIF2AP3;GIMAP2
7	150848001	150849000	LOC105375567;AOC1
7	151255001	151256000	SMARCD3
7	151270001	151271000	SMARCD3
7	151469001	151470000	RN7SL76P;RHEB
7	151752001	151753000	PRKAG2
7	151886001	151887000	PRKAG2;PRKAG2-AS1
7	151899001	151900000	RNU6-604P
7	152266001	152268000	KMT2C;KMT2C
7	152404001	152405000	KMT2C
7	152407001	152408000	KMT2C
7	152786001	152787000	ACTR3B
7	152902001	152903000	ACTR3B
7	152972001	152973000	ACTR3B
7	153381001	153382000	LOC105375577
7	153950001	153951000	DPP6
7	154015001	154016000	DPP6
7	154079001	154080000	DPP6
7	154100001	154101000	DPP6
7	154386001	154387000	DPP6
7	154656001	154657000	DPP6
7	154788001	154789000	DPP6
7	155236001	155237000	LOC105375586;LOC105375587
7	155318001	155319000	INSIG1
7	156448001	156449000	LOC285889
7	156464001	156465000	LINC01006
7	156483001	156484000	LINC01006
7	156518001	156519000	LINC01006;LOC112268012
7	156777001	156778000	LMBR1
7	157887001	157888000	PTPRN2
7	158053001	158054000	PTPRN2
7	158385001	158386000	PTPRN2
7	158458001	158459000	PTPRN2
7	158694001	158695000	NCAPG2
7	158729001	158731000	RPL21P76;ESYT2;RPL21P76;ESYT2
8	146001	148000	WBP1LP3;WBP1LP3
8	205001	206000	SEPTIN14P8;RPL23AP53
8	273001	274000	LOC101927566
8	755001	756000	DLGAP2
8	758001	759000	DLGAP2
8	895001	896000	DLGAP2;LOC112268020
8	939001	940000	DLGAP2

8	980001	981000	DLGAP2
8	1029001	1030000	DLGAP2
8	1089001	1090000	DLGAP2
8	1165001	1166000	DLGAP2
8	1174001	1175000	DLGAP2
8	1344001	1345000	DLGAP2
8	1387001	1388000	DLGAP2
8	1521001	1522000	DLGAP2
8	1824001	1826000	LOC105377779;MIR596;ARHGEF10;MIR596;ARHGEF10
8	2100001	2101000	MYOM2
8	2112001	2113000	MYOM2
8	2118001	2120000	MYOM2;MYOM2
8	2395001	2396000	LOC105377784
8	2561001	2562000	LOC101927815
8	2882001	2883000	LOC105377785
8	2891001	2892000	LOC105377785
8	2986001	2987000	LOC105377785;CSMD1
8	3316001	3317000	CSMD1
8	3324001	3325000	CSMD1
8	3651001	3652000	CSMD1
8	3700001	3701000	CSMD1;RNA5SP251
8	3743001	3744000	CSMD1
8	3852001	3853000	CSMD1;LOC105377790
8	4227001	4228000	CSMD1
8	4401001	4403000	CSMD1;CSMD1
8	4500001	4501000	CSMD1
8	4676001	4677000	CSMD1
8	4855001	4856000	CSMD1
8	4862001	4863000	CSMD1
8	5592001	5593000	LOC105377794
8	5605001	5606000	LOC105377794
8	6012001	6013000	LOC105377795
8	6409001	6410000	MCPH1-DT;MCPH1
8	6437001	6438000	MCPH1
8	6601001	6602000	MCPH1
8	6826001	6827000	XKR5;GS1-24F4.2
8	6828001	6829000	XKR5;GS1-24F4.2
8	7086001	7087000	LOC107986875
8	7605001	7606000	LOC107986910
8	9059001	9060000	ERI1;SNORD3I
8	9062001	9064000	ERI1;SNORD3I;RNU7-55P;ERI1;SNORD3I;RNU7-55P
8	9096001	9097000	ERI1
8	9329001	9330000	LOC157273
8	10174001	10175000	MSRA
8	10352001	10353000	MSRA
8	10385001	10386000	MSRA
8	10574001	10575000	LOC105379237
8	10576001	10577000	LOC105379237
8	10610001	10611000	RP1L1
8	10623001	10624000	RP1L1
8	10626001	10627000	RP1L1
8	10658001	10659000	RP1L1;MIR4286
8	10694001	10695000	C8orf74;RNA5SP252
8	11144001	11145000	XKR6
8	11420001	11421000	FAM167A-AS1;FAM167A
8	11559001	11560000	BLK;LOC105379241
8	11637001	11638000	LOC105379242
8	12484001	12485000	LOC100506990;ENPP7P6
8	12493001	12494000	LOC100506990;ENPP7P6
8	12578001	12579000	LOC729732;RPS3AP34
8	12623001	12624000	LOC729732;RPS3AP35
8	13075001	13076000	DLC1
8	13249001	13250000	DLC1
8	16456001	16457000	LOC101929028
8	16919001	16920000	LOC105379297
8	17147001	17148000	RPL9P20;ZDHHC2;LOC107986918
8	17166001	17167000	ZDHHC2;LOC107986918
8	17401001	17402000	MTMR7
8	17404001	17405000	MTMR7
8	18081001	18082000	ASAH1;LOC101929066;MRPS18CP3;LOC100133073
8	18835001	18836000	PSD3
8	19482001	19483000	CSGALNACT1
8	20273001	20274000	LZTS1;LZTS1-AS1
8	20660001	20661000	LOC105379315
8	20671001	20672000	LOC105379315
8	20813001	20814000	LOC105379315
8	21765001	21766000	GFRA2
8	21783001	21784000	GFRA2
8	21885001	21886000	LOC107986923
8	21910001	21911000	DOK2;XPO7
8	22051001	22052000	FGF17;DMTN
8	22053001	22054000	FGF17;DMTN
8	22077001	22078000	DMTN
8	22797001	22798000	PEBP4
8	22819001	22820000	PEBP4;LOC105379325
8	22913001	22914000	PEBP4
8	23011001	23012000	RHOBTB2;TNFRSF10B

8	23250001	23251000	CHMP7
8	23290001	23291000	R3HCC1;LOXL2
8	23365001	23366000	LOXL2;LOC100507156
8	23424001	23425000	ENTPD4
8	24318001	24319000	ADAM28;LOC101929294
8	25387001	25388000	DOCK5
8	26414001	26415000	BNIP3L
8	26642001	26643000	DPYSL2
8	27342001	27343000	PTK2B
8	27643001	27644000	SCARA3
8	28481001	28482000	FBXO16
8	28712001	28713000	EXTL3
8	29154001	29155000	KIF13B
8	29878001	29879000	LOC105379354
8	30032001	30033000	MAP2K1P1
8	31040001	31041000	PURG;WRN
8	31991001	31992000	NRG1
8	32527001	32528000	NRG1
8	33444001	33445000	FUT10;LOC100507379
8	33579001	33580000	RN7SL621P
8	37066001	37067000	LOC105379377
8	37526001	37527000	LINC01605
8	37565001	37566000	LINC01605;LOC105379379
8	38454001	38455000	FGFR1
8	40648001	40649000	ZMAT4;LOC107986938
8	40760001	40761000	ZMAT4
8	40884001	40885000	ZMAT4
8	41028001	41029000	RNU6-356P
8	41164001	41165000	LOC105379771
8	41752001	41753000	ANK1
8	41865001	41866000	ANK1
8	42307001	42308000	IKBKB;LOC105379395
8	42911001	42912000	HOOK3
8	42931001	42932000	HOOK3
8	43117001	43118000	POMK
8	47366001	47367000	SPIDR;LOC107986940
8	47990001	47992000	TCONS_00068220;UBE2V2;TCONS_00068220;UBE2V2
8	47993001	47994000	TCONS_00068220;UBE2V2
8	48002001	48003000	TCONS_00068220;UBE2V2
8	48583001	48584000	LOC101929268
8	50687001	50688000	SNTG1
8	51724001	51725000	PXDNL;BTF3P1
8	52200001	52201000	ST18;LOC101929341
8	52375001	52376000	ST18
8	53956001	53957000	RGS20;TCEA1
8	53960001	53961000	RGS20;TCEA1
8	54399001	54400000	LOC105375840
8	55982001	55983000	LYN;RN7SL798P
8	56430001	56431000	LOC105375849;PENK
8	56471001	56472000	PENK-AS1;SEPTIN10P1
8	56923001	56924000	RNU6-13P
8	58890001	58891000	TOX
8	59069001	59070000	TOX
8	60207001	60208000	CA8
8	60243001	60244000	CA8
8	60306001	60307000	LOC105375864
8	60398001	60399000	PDCL3P1;LINC01301
8	61272001	61273000	CLVS1
8	61297001	61298000	CLVS1
8	61914001	61915000	LINC02842;C1GALT1P3
8	62667001	62668000	NKAIN3
8	64241001	64242000	LINC01414
8	66916001	66917000	MCMDC2;SNHG6;SNORD87
8	67173001	67174000	CSPP1;ARFGEF1
8	67583001	67584000	CPA6;LOC105375886;MTCYBP30;MTND6P30;MTND5P44;MTND4P39;MTND4LP27;MTCO3P3;MTATP6P12
8	67587001	67588000	CPA6;LOC105375886;MTCYBP30;MTND6P30;MTND5P44;MTND4P39;MTND4LP27;MTCO3P3;MTATP6P12
8	67720001	67721000	CPA6
8	68412001	68413000	C8orf34
8	68706001	68707000	C8orf34
8	68976001	68977000	LINC01592
8	69020001	69021000	LINC01592
8	69056001	69057000	LINC01592
8	71274001	71276000	EYA1;EYA1
8	73348001	73349000	RDH10-AS1
8	74665001	74666000	MIR2052HG
8	75300001	75301000	HIGD1AP6
8	76258001	76259000	LOC102724858;RNU2-54P
8	76652001	76653000	ZFHX4-AS1
8	76829001	76830000	ZFHX4
8	78843001	78844000	LOC105375914;LINC02605
8	79760001	79761000	HEY1;LINC01607;LOC101927040
8	79808001	79810000	LINC01607;LOC101927040;RNU7-85P;LINC01607;LOC101927040;RNU7-85P
8	79994001	79995000	TPD52;MRPS28
8	80691001	80692000	ZNF704
8	80780001	80781000	ZNF704;RN7SL308P
8	80923001	80924000	LOC107986894
8	80982001	80983000	PAG1

8	81822001	81823000	SNX16
8	81842001	81843000	SNX16;LINC02235
8	81914001	81915000	LINC02235
8	81916001	81918000	LINC02235;LINC02235
8	82117001	82118000	LINC02839
8	85720001	85721000	REXO1L12P
8	86226001	86227000	SLC7A13
8	88742001	88743000	LOC105375630
8	88745001	88746000	LOC105375630
8	88747001	88748000	LOC105375630
8	88859001	88860000	LOC105375630;LOC105375631
8	89788001	89789000	RIPK2
8	90092001	90093000	CALB1
8	90293001	90294000	LINC00534
8	90608001	90609000	LINC01030
8	90792001	90793000	LOC105375633;NECAB1
8	92151001	92152000	RPS26P10
8	95301001	95302000	CFAP418-AS1
8	95489001	95490000	CFAP418-AS1
8	97072001	97073000	CPQ;LOC101927066
8	97201001	97205000	LOC101927066;LOC101927066;LOC101927066;LOC101927066
8	97431001	97432000	LOC101927066
8	97829001	97831000	LAPTM4B;LAPTM4B
8	98181001	98182000	LOC105375660;NIPAL2
8	98838001	98839000	STK3
8	99126001	99127000	VPS13B
8	100506001	100507000	LOC105375670;ANKRD46
8	100557001	100558000	ANKRD46;GAPDHP62
8	100749001	100750000	LOC105375673;RNU6ATAC41P
8	101114001	101115000	LOC105375674
8	101679001	101680000	GRHL2;NCALD
8	102913001	102914000	MAILR;RPL5P24
8	103190001	103191000	BAALC
8	103317001	103318000	FZD6;SNORD173
8	106587001	106588000	OXR1;OXR1-AS1
8	106622001	106623000	OXR1;OXR1-AS1
8	107307001	107308000	ANGPT1
8	114285001	114286000	LOC105375710
8	118543001	118544000	SAMD12
8	118914001	118915000	TNFRSF11B
8	119000001	119001000	COLEC10
8	122528001	122529000	SMILR
8	122831001	122832000	ZHX2
8	122916001	122917000	ZHX2
8	123101001	123102000	TBC1D31
8	123645001	123646000	KLHL38
8	123811001	123812000	FAM91A1
8	124009001	124010000	FER1L6;FER1L6-AS1
8	124548001	124549000	TATDN1;NDUFB9;MTSS1
8	124663001	124664000	MTSS1
8	124972001	124973000	ZNF572
8	125428001	125429000	TRIB1
8	125596001	125598000	LOC105375746;LOC105375746
8	125638001	125639000	LOC105375746
8	126567001	126568000	LRATD2;LOC105375751
8	127136001	127137000	LOC105375752
8	127257001	127258000	CASC21
8	127337001	127338000	CASC21;CASC8
8	127432001	127433000	CASC8
8	127435001	127437000	CASC8;CASC8
8	128452001	128453000	LINC00824
8	129593001	129594000	CCDC26
8	129639001	129640000	CCDC26
8	130073001	130075000	ASAP1;RNU6-1255P;ASAP1-IT2;ASAP1;RNU6-1255P;ASAP1-IT2
8	130121001	130122000	ASAP1
8	130447001	130448000	ASAP1
8	130855001	130856000	ADCY8;LOC105375762
8	131722001	131723000	LOC100420215
8	132620001	132621000	DNAAF11
8	132906001	132907000	TG
8	133007001	133008000	TG;RPL21P78;LOC105375768
8	133106001	133107000	TG;SLA
8	133667001	133668000	LOC105375773
8	134522001	134523000	ZFAT
8	134578001	134579000	ZFAT
8	134677001	134678000	ZFAT
8	135260001	135261000	LINC01591
8	137827001	137828000	LOC401478
8	137838001	137839000	LOC401478
8	137870001	137871000	LOC401478
8	138179001	138180000	FAM135B
8	138181001	138182000	FAM135B
8	138284001	138285000	FAM135B
8	138865001	138866000	COL22A1
8	139747001	139748000	TRAPPC9
8	139816001	139817000	TRAPPC9
8	140062001	140063000	TRAPPC9

8	140154001	140155000	TRAPPC9
8	140188001	140189000	TRAPPC9
8	140194001	140195000	TRAPPC9
8	140285001	140287000	TRAPPC9;LOC105375779;TRAPPC9;LOC105375779
8	141061001	141062000	LOC105375784
8	141347001	141349000	LINC01300;GPR20;LINC01300;GPR20
8	141385001	141386000	LOC101928037;LOC105375790;PTP4A3
8	141451001	141452000	MROH5;LOC105375789;HNRNPA1P38
8	141516001	141517000	MROH5;LOC105375791
8	141970001	141971000	LOC100131146
8	142185001	142186000	MIR4472-1;LOC105375792
8	142212001	142213000	LINC00051;TSNARE1
8	142272001	142273000	TSNARE1
8	142318001	142319000	TSNARE1
8	142478001	142479000	ADGRB1
8	142766001	142767000	LYNX1-SLURP2;SLURP2;LYNX1
8	143015001	143016000	LY6E-DT;LY6E
8	143181001	143183000	LOC107986906;LOC107986906
8	143390001	143391000	RHPN1
8	143418001	143419000	LOC107986984;MAFA-AS1;MAFA
8	143648001	143649000	ZNF623
8	143666001	143667000	LOC105375799
8	143723001	143724000	LOC101928160;MAPK15;FAM83H;MIR4664
8	143874001	143875000	EPPK1
8	144005001	144006000	SPATC1
8	144022001	144023000	SPATC1
8	144062001	144063000	OPLAH;MIR6846;EXOSC4
8	144280001	144281000	BOP1
8	144340001	144341000	SCRT1
8	144488001	144489000	PPP1R16A;LOC101928953
8	144661001	144662000	ARHGAP39
8	144694001	144695000	ARHGAP39;LOC100996662
8	144758001	144759000	ZNF251;LOC107986986;LOC105375805
8	144822001	144823000	ZNF517;LOC100130027;ZNF7
8	144959001	144960000	ZNF16
9	209001	210000	LOC105375943;DOCK8;DOCK8-AS1
9	585001	586000	KANK1
9	954001	955000	DMRT1
9	1368001	1369000	LOC102723803
9	1699001	1700000	LOC105375951
9	1714001	1715000	LOC105375951
9	1941001	1942000	LOC105375951
9	2102001	2103000	SMARCA2
9	2172001	2173000	SMARCA2;RNU2-25P;LOC107987043
9	2512001	2514000	LOC101930048;LOC101930048
9	3815001	3816000	GLIS3
9	3900001	3901000	GLIS3;GLIS3-AS1
9	3926001	3927000	GLIS3
9	4496001	4497000	SLC1A1
9	4528001	4529000	SLC1A1
9	4829001	4830000	RCL1
9	4990001	4991000	JAK2;INSL6
9	5095001	5096000	JAK2;INSL6;PDSS1P1;MTND6P5;MTND5P36;MTND1P11;MTND2P36;MTCO1P11;MTCO2P11;MTATP6P11;MTCO3P11
9	6020001	6021000	RANBP6
9	6782001	6783000	KDM4C
9	8675001	8676000	PTPRD
9	12825001	12826000	LURAP1L
9	13039001	13040000	LOC100130801
9	13814001	13815000	LOC101929507
9	13987001	13988000	LOC101929507;PES1P2
9	14220001	14221000	NFIB
9	14338001	14339000	NFIB
9	15055001	15056000	CLCN3P1;PSIP1P1
9	15517001	15518000	PSIP1;LOC107987048;RN7SL98P;FTH1P12
9	16052001	16053000	CCDC171
9	16264001	16265000	C9orf92
9	17271001	17272000	CNTLN
9	17623001	17624000	SH3GL2
9	18196001	18197000	ADAMTSL1
9	19024001	19025000	SAXO1;PSMC3P1
9	19030001	19031000	SAXO1;PSMC3P1
9	19427001	19428000	ACER2
9	19987001	19988000	SLC24A2
9	20234001	20235000	SLC24A2
9	21489001	21490000	MIR31HG;IFNE
9	22127001	22128000	CDKN2B-AS1
9	22662001	22663000	LINC01239;LOC107987054
9	23725001	23726000	ELAVL2
9	25669001	25670000	TUSC1
9	33242001	33243000	SPINK4;BAG1
9	33387001	33388000	AQP7
9	33404001	33405000	AQP7;LOC105376020
9	33434001	33435000	AQP3
9	34828001	34829000	PHF24;FAM205BP
9	35151001	35152000	LOC730110
9	35154001	35155000	LOC730110;UNC13B
9	35322001	35323000	UNC13B

9	35487001	35488000	ATP8B5P;RUSC2;LOC105376026
9	35607001	35608000	TESK1;MIR4667;CD72
9	35648001	35649000	LOC101926948;SIT1;RMRP;CCDC107;ARHGEF39
9	35684001	35685000	CA9;TPM2
9	35837001	35838000	FAM221B;TMEM8B
9	36188001	36189000	CLTA
9	36327001	36328000	LOC102724322;RNF38
9	36779001	36780000	LOC105376030
9	36964001	36965000	PAX5
9	38346001	38347000	LOC107987065;LOC105376040
9	38650001	38651000	YWHABP1
9	38680001	38681000	RNU6-765P
9	38962001	38963000	LOC101927042
9	39842001	39843000	FGF7P3
9	40219001	40220000	LOC102724431;LOC105369249;ANKRD20A2P
9	40357001	40358000	CNN2P2;GXYLT1P3
9	40490001	40491000	AQP7P5;LOC102724580
9	41868001	41869000	VN2R6P
9	42658001	42659000	FGF7P4
9	43118001	43119000	LOC102724922;LOC102724904
9	61071001	61072000	LOC101930090
9	62493001	62494000	LINC01189
9	62839001	62840000	LOC100132249;PTGER4P2-CDK2AP2P2;PTGER4P2;CDK2AP2P2
9	63780001	63781000	CDK2AP2P3;PTGER4P3;LOC107987021
9	63783001	63784000	CDK2AP2P3;PTGER4P3;LOC107987021
9	63814001	63815000	LINC00537;RNA5SP284;DUX4L50;MIR4477B
9	63819001	63820000	LINC00537;RNA5SP284;DUX4L50;MIR4477B
9	64445001	64446000	ANKRD20A4-ANKRD20A20P;ANKRD20A20P;SNX18P9
9	64451001	64452000	ANKRD20A4-ANKRD20A20P
9	67152001	67153000	CNTNAP3P2
9	67339001	67340000	VN2R7P
9	67401001	67402000	LOC101927602
9	68329001	68331000	LOC101929800;LOC101929800
9	68827001	68828000	PIP5K1B
9	70823001	70824000	TRPM3;LOC107987078
9	72200001	72201000	GDA
9	73318001	73319000	LOC101927281
9	75178001	75179000	LOC105376090
9	76665001	76666000	LOC105376095;PRUNE2
9	76671001	76672000	LOC105376095;PRUNE2
9	76718001	76719000	PRUNE2
9	76766001	76767000	PRUNE2;PCA3
9	78186001	78187000	ASS1P3;RPL21P84
9	78247001	78248000	CEP78
9	81915001	81916000	LOC105376108;SPATA31D5P
9	81968001	81969000	LOC105376108;SPATA31D2P;LOC105376107
9	82061001	82062000	LOC105376107;SPATA31B1P
9	82416001	82417000	LOC112268046
9	82704001	82705000	LOC107987087
9	83750001	83751000	GKAP1
9	83975001	83976000	HNRNPK;MIR7-1;RMI1
9	84334001	84335000	SLC28A3
9	84766001	84767000	NTRK2
9	84840001	84841000	NTRK2
9	85429001	85430000	LOC105376121
9	87043001	87044000	LINC02893
9	87412001	87413000	LOC112268032
9	87415001	87416000	LOC112268032
9	87663001	87664000	DAPK1
9	87873001	87874000	LOC107987091;LOC497256;SPATA31E1
9	87909001	87910000	LOC497256;SPATA31C1
9	88132001	88133000	SPATA31C2
9	88177001	88178000	LOC389768;LOC100129340
9	88563001	88564000	NXNL2
9	89166001	89167000	SHC3
9	89371001	89373000	SECISBP2;SEMA4D;SECISBP2;SEMA4D
9	89404001	89405000	SEMA4D
9	89492001	89493000	SEMA4D
9	89546001	89547000	LOC105376137
9	89671001	89672000	LOC100129066
9	89693001	89694000	LOC100129066;LOC105376140
9	89729001	89730000	LOC100129066
9	90413001	90414000	LINC01508
9	90424001	90425000	LINC01508
9	90489001	90490000	LINC01501
9	91230001	91231000	AUH
9	91710001	91711000	LOC105376147
9	91784001	91785000	ROR2
9	92073001	92074000	SPTLC1
9	92541001	92542000	CENPP;ECM2
9	92889001	92890000	LOC642943;SNX18P2
9	92952001	92953000	FGD3
9	92959001	92960000	FGD3
9	93031001	93032000	FGD3
9	93118001	93119000	CARD19;NINJ1
9	93139001	93140000	NINJ1
9	93293001	93294000	WNK2

9	93474001	93475000	FAM120A
9	93587001	93588000	PHF2;MIR548AU
9	93624001	93625000	PHF2
9	93673001	93674000	PHF2
9	94435001	94436000	MFSD14B;YRDCP1
9	94633001	94634000	FBP1
9	94640001	94641000	FBP1
9	94648001	94649000	FBP1
9	94845001	94846000	AOPEP
9	95475001	95476000	PTCH1;LOC100507346
9	95559001	95560000	LOC105376157
9	96005001	96006000	ERCC6L2
9	96133001	96134000	LOC112268039
9	97206001	97207000	ANKRD18CP;ZNF322P1
9	97216001	97217000	ANKRD18CP;LOC107987025
9	97719001	97720000	PTCSC2
9	97742001	97743000	PTCSC2
9	97762001	97763000	PTCSC2
9	98536001	98537000	GABBR2
9	98642001	98643000	GABBR2
9	98702001	98703000	GABBR2
9	98884001	98885000	LOC102724684;NME2P3
9	99444001	99445000	LOC107987011
9	99670001	99671000	LOC101928438
9	99870001	99871000	NR4A3
9	100430001	100431000	MSANTD3
9	100465001	100466000	MSANTD3-TMEFF1;TMEFF1
9	100474001	100475000	MSANTD3-TMEFF1;TMEFF1
9	100528001	100529000	MSANTD3-TMEFF1;TMEFF1
9	101539001	101540000	RNF20
9	103005001	103006000	CYLC2
9	105251001	105252000	LOC112268038;SLC44A1
9	105580001	105581000	FKTN
9	105781001	105782000	TMEM38B
9	107109001	107110000	LOC340512;LOC100128086
9	107375001	107376000	LOC107987111
9	107392001	107393000	LOC107987111
9	107718001	107719000	LOC105376207
9	107944001	107945000	LOC105376211
9	107986001	107987000	LOC105376211
9	109083001	109084000	TMEM245
9	109214001	109215000	EPB41L4B
9	109220001	109221000	EPB41L4B
9	109278001	109279000	EPB41L4B
9	109386001	109387000	PTPN3
9	109816001	109817000	PALM2AKAP2
9	110173001	110174000	PALM2AKAP2;LOC105376217
9	110677001	110678000	MUSK
9	111843001	111844000	RNU6-1013P
9	112077001	112078000	SUSD1
9	112105001	112106000	SUSD1
9	112155001	112156000	SUSD1
9	112843001	112845000	SNX30;SNX30
9	113326001	113327000	WDR31
9	113396001	113397000	ALAD
9	113450001	113451000	RGS3
9	113614001	113615000	LOC105376222
9	113764001	113765000	LOC100288542
9	113933001	113935000	ZNF618;ZNF618
9	114291001	114292000	COL27A1
9	114375001	114376000	AKNA
9	114378001	114379000	AKNA
9	114621001	114622000	TMEM268
9	114898001	114899000	TNFSF8
9	114931001	114932000	TNFSF8
9	115179001	115180000	DELEC1
9	115932001	115933000	LINC00474
9	116293001	116294000	PAPPA;PAPPA-AS2
9	116472001	116473000	ASTN2
9	116936001	116937000	ASTN2
9	117760001	117761000	LOC105376244
9	119251001	119252000	BRINP1
9	120104001	120105000	LOC107987124
9	120382001	120383000	CDK5RAP2
9	121087001	121088000	CNTRL
9	121576001	121577000	LOC107987016;DAB2IP
9	121631001	121632000	DAB2IP
9	121644001	121645000	DAB2IP
9	121661001	121662000	DAB2IP
9	121791001	121792000	DAB2IP
9	121806001	121807000	LOC107987125;TTLL11
9	121838001	121839000	TTLL11
9	121941001	121942000	TTLL11
9	121987001	121988000	TTLL11
9	122124001	122125000	MIR4478;NDUFA8
9	122238001	122239000	LHX6;RBM18
9	122329001	122330000	MRRF

9	122372001	122374000	PTGS1;PTGS1
9	122870001	122872000	RC3H2;SNORD90;RC3H2;SNORD90
9	123106001	123107000	RABGAP1;STRBP;MIR600HG;MIR600
9	123155001	123156000	STRBP
9	123193001	123194000	STRBP
9	123259001	123260000	STRBP
9	123346001	123347000	CRB2
9	123355001	123356000	CRB2
9	123460001	123461000	DENND1A
9	123858001	123859000	DENND1A
9	123882001	123883000	DENND1A
9	124035001	124036000	LHX2;LOC107987037
9	124294001	124295000	NEK6
9	124423001	124424000	PSMB7
9	124894001	124895000	GOLGA1;RNU4-82P
9	125206001	125207000	LOC105376271;RABEPK
9	125289001	125290000	GAPVD1
9	126153001	126154000	LOC105376274
9	126275001	126277000	LOC101929116;LOC101929116
9	126366001	126367000	MVB12B
9	126418001	126419000	MVB12B;NRON
9	126468001	126469000	MVB12B
9	126847001	126848000	ZBTB43
9	127413001	127414000	SLC2A8
9	127440001	127441000	ZNF79;RPL12;SNORA65
9	127513001	127514000	LRSAM1;NIBAN2
9	127577001	127578000	NIBAN2
9	127629001	127630000	STXBP1
9	127664001	127665000	STXBP1
9	127762001	127763000	SH2D3C
9	127860001	127861000	ENG;AK1;MIR4672
9	127875001	127876000	AK1;MIR4672;ST6GALNAC6
9	128254001	128255000	DNM1;MIR199B;MIR3154;GOLGA2
9	128654001	128655000	DYNC2I2;VTI1BP4;LOC105376287;HMGA1P4
9	129034001	129035000	SH3GLB2;MIGA2
9	129242001	129243000	LOC105376290
9	129339001	129340000	LINC01503
9	129343001	129344000	LINC01503
9	129590001	129591000	LOC105376292
9	129735001	129736000	PTGES
9	129815001	129816000	TOR1B;TOR1A
9	129839001	129840000	C9orf78;USP20
9	130135001	130136000	GPR107;GPRACR
9	130168001	130170000	NCS1;NCS1
9	130283001	130284000	HMCN2
9	130356001	130357000	HMCN2
9	130378001	130379000	HMCN2
9	130949001	130950000	FIBCD1;LOC105376297
9	131065001	131066000	LAMC3
9	131457001	131458000	PRRC2B
9	131516001	131517000	POMT1;LOC105376301;UCK1
9	131548001	131551000	PRRT1B;PRRT1B;PRRT1B
9	131613001	131614000	RAPGEF1
9	131699001	131700000	RAPGEF1
9	132315001	132316000	SETX
9	132799001	132800000	AK8
9	132885001	132886000	AK8;SPACA9;TSC1
9	133124001	133125000	RALGDS
9	133523001	133524000	MYMK;ADAMTSL2
9	133755001	133756000	VAV2
9	133914001	133915000	VAV2
9	134368001	134369000	RXRA
9	134514001	134516000	LOC100506532;LOC100506532
9	134550001	134551000	LOC100506532
9	134655001	134656000	COL5A1;COL5A1-AS1
9	134686001	134687000	COL5A1
9	134847001	134848000	COL5A1;LOC101448202;MIR3689C;MIR3689A;MIR3689D1;MIR3689B;MIR3689D2;MIR3689E;MIR3689F
9	134883001	134884000	FCN2
9	134905001	134906000	FCN1
9	135129001	135130000	OLFM1
9	135242001	135243000	LOC107987138
9	135263001	135264000	LOC107987138
9	135963001	135964000	UBAC1
9	136038001	136039000	NACC2
9	136747001	136748000	LCN10;LCN6;LOC100128593;MIR6722;LCN8
9	136776001	136779000	LOC107987143;ATP6V1G1P3;LOC107987143;ATP6V1G1P3;LOC107987143;ATP6V1G1P3
9	136960001	136961000	LCN12;LINC02692
9	137386001	137387000	EXD3
9	137452001	137453000	NSMF;MIR7114;PNPLA7
9	137488001	137489000	PNPLA7
9	137491001	137492000	PNPLA7
9	137519001	137520000	PNPLA7
9	137563001	137564000	DPH7
9	137569001	137571000	DPH7;DPH7
9	137586001	137587000	DPH7;ZMYND19
9	137598001	137599000	ZMYND19;ARRDC1
9	137668001	137669000	EHMT1

9	137721001	137722000	EHMT1
9	137848001	137849000	MIR602
9	137967001	137968000	CACNA1B
9	138045001	138046000	CACNA1B;LOC101928786
10	258001	259000	ZMYND11;LOC107984190;LOC107984191
10	358001	359000	DIP2C;LOC105376340
10	560001	561000	DIP2C
10	1011001	1012000	GTPBP4;IDI2
10	1116001	1117000	WDR37
10	1347001	1348000	ADARB2;LOC105376342
10	1349001	1350000	ADARB2;LOC105376342
10	1547001	1549000	ADARB2;ADARB2-AS1;ADARB2;ADARB2-AS1
10	1569001	1570000	ADARB2
10	2015001	2016000	LINC00700
10	3063001	3064000	PFKP-DT;PFKP
10	3650001	3651000	LOC105376360;LOC107984193
10	3660001	3661000	LOC105376360
10	3932001	3933000	LINC02660;LOC105376367
10	3992001	3993000	MIR6078
10	4064001	4065000	LOC101927964
10	4088001	4089000	LOC101927964;LOC107984196
10	4149001	4150000	LOC105376368
10	4334001	4335000	LOC105376370
10	5382001	5383000	UCN3
10	5645001	5646000	ASB13
10	5659001	5660000	ASB13
10	5941001	5942000	FBH1;LOC105376384;IL15RA
10	6085001	6086000	RBM17
10	6407001	6408000	PRKCQ
10	6470001	6471000	PRKCQ
10	6495001	6496000	PRKCQ;LOC107984202
10	6520001	6521000	PRKCQ
10	6563001	6564000	PRKCQ
10	6570001	6571000	PRKCQ;PRKCQ-AS1
10	6632001	6633000	LINC02648
10	6773001	6774000	LINC00706;LINC00707
10	6785001	6786000	LINC00706;LINC00707
10	6801001	6802000	LINC00707
10	7041001	7042000	LOC105376387
10	7551001	7552000	LOC105376391;ITIH5
10	7587001	7588000	ITIH5
10	7938001	7939000	TAF3
10	7946001	7947000	TAF3
10	8447001	8448000	LOC105376397
10	9880001	9881000	LINC02663
10	10695001	10696000	CELF2
10	10836001	10837000	CELF2
10	10971001	10972000	CELF2
10	11070001	11071000	CELF2;CELF2-AS2
10	11247001	11248000	CELF2
10	11365001	11366000	LOC105376409
10	11628001	11629000	LOC105376413
10	11737001	11738000	ECHDC3
10	11745001	11746000	ECHDC3
10	11769001	11770000	ECHDC3;LOC105379850
10	11885001	11886000	PROSER2-AS1
10	12035001	12036000	UPF2;LOC105376416
10	12472001	12473000	CAMK1D;LOC107984209
10	12624001	12625000	CAMK1D
10	12730001	12731000	CAMK1D;MIR548Q
10	13724001	13725000	FRMD4A;LOC105376426;RNA5SP301
10	13734001	13735000	FRMD4A;LOC105376426;RNA5SP301
10	14176001	14177000	FRMD4A
10	14314001	14315000	FRMD4A
10	14320001	14321000	FRMD4A
10	14888001	14889000	SUV39H2;DCLRE1C
10	15091001	15092000	ACBD7-DCLRE1CP1;ACBD7;GAPDHP45;RPP38-DT;RPP38
10	15116001	15117000	LOC105376431;NMT2
10	15262001	15263000	FAM171A1
10	15372001	15373000	FAM171A1
10	15546001	15547000	ITGA8
10	15566001	15567000	ITGA8
10	16296001	16297000	LINC02654
10	16506001	16507000	PTER;C1QL3
10	16636001	16637000	RSU1
10	17465001	17466000	ST8SIA6;LOC105376437
10	17472001	17473000	LOC105376437
10	18306001	18307000	CACNB2
10	19419001	19420000	MALRD1
10	19699001	19700000	MALRD1
10	20122001	20123000	PLXDC2
10	22853001	22854000	LOC105376451
10	22994001	22995000	ARMC3;LOC107984215;RNA5SP304
10	24289001	24290000	KIAA1217
10	24718001	24719000	ARHGAP21
10	24944001	24945000	PRTFDC1;THNSL1
10	24980001	24981000	THNSL1;ENKUR

10	25048001	25049000	ENKUR
10	25442001	25443000	GPR158
10	25718001	25719000	LINC00836
10	26002001	26003000	MYO3A
10	26250001	26251000	GAD2
10	26444001	26445000	APBB1IP
10	26708001	26709000	PDSS1;HSPA8P3
10	26808001	26809000	ABI1
10	27814001	27815000	ODAD2
10	28233001	28234000	MPP7
10	28623001	28624000	WAC
10	29074001	29075000	LOC107984174
10	29230001	29231000	LOC105376472
10	30919001	30920000	ZNF438;DDX10P1
10	31193001	31194000	LINC02664
10	31259001	31260000	LINC02664;RNA5SP309
10	31699001	31700000	MACORIS
10	31706001	31707000	MACORIS
10	34101001	34102000	PARD3
10	34142001	34143000	PARD3
10	34654001	34655000	PARD3;RPL37P18
10	34936001	34937000	RNU6-847P;RNU6-193P
10	35141001	35142000	CREM;RNU7-77P
10	35378001	35379000	CCNY
10	35402001	35404000	CCNY;CCNY
10	35407001	35408000	CCNY
10	35413001	35414000	CCNY
10	36433001	36434000	MTND5P17;MTND4P18
10	37142001	37143000	ANKRD30A;RNU6-811P
10	37996001	37997000	ZNF25-DT
10	42144001	42145000	KSR1P1
10	42146001	42147000	KSR1P1
10	42776001	42777000	LOC105378269;BMS1
10	43341001	43342000	LOC105378271;RNU6ATAC11P;LOC107984226
10	43569001	43570000	ZNF239
10	43799001	43800000	LOC105378275;HNRNPA3P1
10	43853001	43854000	LINC00619;LINC00840
10	44880001	44881000	TMEM72-AS1
10	45571001	45573000	MARCHF8;MARCHF8
10	45574001	45575000	MARCHF8
10	46051001	46052000	MSMB
10	46217001	46218000	ANTXRLP1;AHCYP1
10	46559001	46560000	GPRIN2
10	47523001	47524000	ANXA8;BMS1P2-AGAP9;AGAP9;LOC107987147;RNA5SP312
10	47655001	47656000	ANXA8
10	48165001	48166000	FRMPD2
10	48447001	48448000	MAPK8;ARHGAP22
10	48590001	48591000	ARHGAP22
10	48761001	48762000	WDFY4
10	48800001	48801000	WDFY4
10	49313001	49314000	C10orf71
10	49536001	49537000	ERCC6
10	49630001	49632000	CHAT;CHAT
10	49926001	49927000	PARG
10	52034001	52038000	PRKG1;PRKG1;PRKG1;PRKG1
10	52546001	52547000	LOC105378305
10	54167001	54168000	PCDH15
10	59284001	59285000	FAM13C
10	59482001	59483000	LOC107984235
10	60599001	60600000	ANK3
10	60990001	60991000	RHOBTB1
10	61659001	61660000	CABCOCO1
10	62181001	62182000	RTKN2
10	67068001	67069000	CTNNA3;LRRTM3
10	68630001	68631000	TET1;COX20P1
10	68781001	68782000	CCAR1
10	68904001	68905000	STOX1;DDX50;RNU6-571P
10	69170001	69171000	VPS26A;RPS12P17;SUPV3L1
10	69485001	69486000	TSPAN15
10	69501001	69502000	TSPAN15
10	69559001	69560000	TSPAN15
10	69775001	69776000	RPL5P26
10	69828001	69829000	COL13A1;LOC105378347
10	69841001	69842000	COL13A1
10	69880001	69881000	COL13A1
10	70937001	70938000	LINC02622
10	71324001	71325000	SLC29A3
10	71403001	71404000	CDH23
10	71482001	71483000	CDH23;LOC105378356
10	71536001	71537000	CDH23
10	71585001	71586000	CDH23
10	71629001	71630000	CDH23
10	71689001	71690000	CDH23
10	73789001	73790000	FUT11;LOC107984243;CHCHD1;ZSWIM8;ZSWIM8-AS1
10	74114001	74115000	VCL;AP3M1
10	74218001	74219000	ADK
10	74572001	74573000	ADK

10	74964001	74965000	KAT6B
10	75374001	75375000	ZNF503;SPA17P1
10	76093001	76094000	LRMDA
10	77066001	77067000	KCNMA1
10	77538001	77539000	KCNMA1
10	77607001	77608000	KCNMA1
10	78939001	78940000	ZMIZ1-AS1
10	78954001	78955000	ZMIZ1-AS1
10	79034001	79035000	ZMIZ1-AS1
10	79142001	79143000	ZMIZ1
10	79318001	79319000	ZMIZ1;LOC107984247
10	80160001	80161000	ANXA11
10	80327001	80328000	DYDC1
10	80520001	80521000	TSPAN14;LOC101929574
10	81906001	81907000	NRG3;LOC107984294
10	86189001	86190000	GRID1
10	86226001	86227000	GRID1
10	86266001	86267000	GRID1;MIR346
10	86284001	86285000	GRID1
10	86292001	86293000	GRID1
10	86294001	86295000	GRID1
10	86332001	86333000	GRID1
10	86340001	86341000	GRID1
10	88442001	88443000	RNLS
10	88536001	88537000	RNLS
10	89144001	89145000	LOC100289238
10	90808001	90809000	HTR7
10	90811001	90812000	HTR7
10	90838001	90839000	HTR7
10	91306001	91307000	HECTD2-AS1
10	91591001	91592000	HECTD2-AS1
10	91903001	91904000	FGFBP3
10	91987001	91988000	BTAF1
10	92600001	92601000	KIF11;RPL11P4
10	93315001	93316000	MYOF
10	93415001	93416000	MYOF
10	95337001	95338000	SORBS1
10	95414001	95415000	SORBS1
10	95497001	95498000	SORBS1
10	96316001	96317000	DNTT
10	96687001	96688000	PIK3AP1
10	96689001	96690000	PIK3AP1
10	96733001	96734000	RNU6-1274P
10	96736001	96737000	RNU6-1274P
10	97038001	97039000	SLIT1
10	97213001	97214000	ARHGAP19-SLIT1;LOC112268065;LOC105378447;ARHGAP19
10	97365001	97366000	RRP12
10	97501001	97502000	MMS19;UBTD1
10	97880001	97881000	GOLGA7B;CRTAC1;MIR3085
10	97895001	97896000	CRTAC1
10	97928001	97929000	CRTAC1
10	99347001	99348000	CNNM1
10	99717001	99718000	ENTPD7;COX15
10	99880001	99881000	DNMBP
10	100057001	100058000	CPN1;MTATP8P4
10	100091001	100092000	CPN1
10	100100001	100101000	CYP2C23P
10	101472001	101473000	BTRC
10	101834001	101835000	KCNIP2-AS1;KCNIP2;LOC107984029
10	102221001	102222000	ELOVL3;PITX3;GBF1
10	102414001	102415000	PSD;FBXL15;CUEDC2
10	102457001	102458000	C10orf95-AS1;C10orf95;MFSD13A
10	102626001	102627000	SUFU
10	103020001	103021000	CNNM2
10	103212001	103213000	ST13P13
10	103570001	103571000	NEURL1
10	103666001	103667000	SH3PXD2A
10	104088001	104089000	COL17A1
10	104913001	104914000	SORCS3
10	105060001	105061000	SORCS3
10	105733001	105734000	LINC02627
10	106018001	106019000	LOC105378468
10	110204001	110205000	MXI1
10	110446001	110447000	LOC105378482;HMGB3P5
10	110685001	110686000	RBM20
10	110816001	110817000	RBM20
10	110910001	110911000	PDCD4;BBIP1;SHOC2
10	111397001	111398000	LOC105378484
10	111815001	111816000	LOC105378486
10	112231001	112232000	LOC101927600
10	112913001	112914000	LOC107984158
10	113058001	113059000	TCF7L2
10	113148001	113149000	TCF7L2
10	114004001	114005000	LOC105378492
10	114249001	114250000	VWA2;AURKAP2
10	116479001	116480000	PNLIPRP3
10	116485001	116486000	PNLIPRP3

10	117500001	117501000	EMX2OS
10	118238001	118239000	LOC101927760
10	118741001	118742000	CACUL1
10	119028001	119029000	NANOS1;EIF3A
10	119140001	119141000	DENND10;SFXN4
10	119167001	119168000	SFXN4;PRDX3
10	120750001	120751000	LOC105378516
10	120846001	120848000	WDR11-AS1;WDR11;WDR11-AS1;WDR11
10	120850001	120851000	WDR11-AS1;WDR11
10	121797001	121798000	ATE1
10	121838001	121839000	ATE1
10	122097001	122098000	TACC2
10	122182001	122183000	TACC2
10	122401001	122402000	PLEKHA1
10	122673001	122674000	LOC112577516
10	123383001	123384000	LINC02641
10	123393001	123394000	LINC02641
10	123790001	123791000	CPXM2
10	124305001	124306000	LOC107984184
10	124871001	124872000	NPM1P31
10	125451001	125452000	LOC105378543
10	125535001	125536000	LOC105378544;LOC105378545
10	125926001	125927000	FANK1
10	126051001	126052000	ADAM12
10	126113001	126114000	ADAM12
10	126133001	126134000	ADAM12
10	126233001	126234000	ADAM12
10	126944001	126945000	DOCK1
10	127275001	127276000	DOCK1
10	127740001	127741000	FOXI2
10	129793001	129794000	LINC02666
10	130024001	130025000	C10orf143
10	130051001	130052000	C10orf143
10	130287001	130288000	LINC02646;LOC100419870
10	130416001	130417000	LINC02646
10	130971001	130972000	MIR378C
10	131241001	131242000	TCERG1L
10	131768001	131769000	LINC01164
10	131899001	131900000	LOC100134362;PPP2R2D
10	131913001	131914000	PPP2R2D
10	132131001	132132000	JAKMIP3
10	132139001	132140000	JAKMIP3
10	132253001	132254000	STK32C
10	132273001	132274000	STK32C
10	132430001	132431000	LOC105378568
10	132462001	132463000	LOC105378570;LOC105378569
10	132514001	132515000	LOC107984282
10	132637001	132638000	INPP5A
10	132862001	132863000	CFAP46;LOC105378571
10	132963001	132964000	LINC01166;LINC01167;LINC01168
10	133320001	133321000	TUBGCP2;ZNF511;CALY
10	133530001	133531000	LOC107984284;CYP2E1
10	133687001	133688000	DUX4L22;DUX4L21;DUX4L20
10	133689001	133690000	DUX4L21;DUX4L20
10	133763001	133764000	DUX4L13;DUX4L14;DUX4L15
11	375001	376000	B4GALNT4
11	954001	955000	AP2A2;RNU6-1025P
11	1016001	1017000	AP2A2;MUC6
11	1094001	1095000	MUC2
11	1185001	1186000	MUC5AC
11	1687001	1688000	FAM99B;LINC02708;KRTAP5-6
11	1777001	1778000	LOC105376517
11	1844001	1845000	SYT8;TNNI2;LOC107984299;LSP1
11	1864001	1865000	LSP1;MIR4298
11	1894001	1895000	LSP1;LINC01150
11	2124001	2125000	INS-IGF2;IGF2;MIR483
11	2483001	2484000	KCNQ1
11	2864001	2865000	KCNQ1-AS1;KCNQ1DN
11	2905001	2906000	SLC22A18AS;SLC22A18
11	2966001	2967000	NAP1L4;SNORA54
11	3648001	3650000	ART5;ART1;ART5;ART1
11	3652001	3653000	ART5;ART1
11	3658001	3659000	ART1;CHRNA10
11	4221001	4222000	RDXP1
11	4223001	4225000	SSU72P5;SSU72P5
11	4321001	4322000	SSU72P3
11	5164001	5165000	OR52A1;OR51A1P
11	5422001	5423000	OR51B5;OR51Q1;OR51K1P
11	5928001	5929000	LOC112268071
11	5931001	5932000	LOC112268071
11	6318001	6319000	CAVIN3;LOC101927825
11	6483001	6484000	TRIM3;ARFIP2;TIMM10B;LOC644169
11	6646001	6647000	DCHS1
11	7458001	7459000	SYT9;LOC100506258
11	7516001	7517000	OLFML1;PPFIBP2
11	7518001	7519000	OLFML1;PPFIBP2
11	7606001	7607000	PPFIBP2

11	7697001	7698000	OVCH2;LOC105376533
11	8024001	8025000	CASC23;TUB
11	8220001	8221000	LMO1;LOC105376536
11	8337001	8338000	STK33
11	8702001	8703000	DENND2B
11	8737001	8739000	DENND2B;DENND2B
11	8781001	8782000	DENND2B;LOC105376539;LOC102724784
11	8995001	8996000	NRIP3;NRIP3-DT
11	9185001	9186000	DENND5A
11	10394001	10395000	CAND1.11;RNU6ATAC33P
11	11429001	11430000	GALNT18
11	11525001	11526000	GALNT18
11	11966001	11967000	USP47;DKK3
11	12149001	12150000	MICAL2
11	12234001	12235000	MICAL2
11	12275001	12276000	MICAL2
11	12504001	12505000	PARVA
11	13310001	13311000	ARNTL
11	13324001	13325000	ARNTL
11	13332001	13333000	ARNTL
11	13348001	13349000	ARNTL;RN7SKP151
11	13351001	13353000	ARNTL;RN7SKP151;ARNTL;RN7SKP151
11	13409001	13410000	BTBD10
11	13875001	13876000	LINC02545
11	14236001	14237000	SPON1
11	17012001	17013000	PLEKHA7;LOC112268085;LOC107984416
11	17746001	17747000	KCNC1
11	17751001	17752000	KCNC1
11	17896001	17897000	SERGEF
11	18002001	18003000	SERGEF
11	18644001	18645000	SPTY2D1;LOC105376578
11	19027001	19028000	MRGPRX9P
11	19604001	19605000	NAV2
11	19998001	19999000	NAV2
11	20019001	20020000	NAV2
11	20568001	20569000	LOC105376584;HMGB1P40
11	20572001	20573000	LOC105376584;HMGB1P40
11	21155001	21156000	NELL1
11	22020001	22021000	LOC102723370
11	23185001	23186000	LINC02718
11	24711001	24712000	LUZP2
11	25146001	25147000	LOC100130747
11	27190001	27191000	BBOX1-AS1
11	27430001	27431000	LGR4
11	27634001	27635000	BDNF-AS;LINC00678
11	28309001	28310000	METTL15
11	28511001	28512000	METTL15
11	28603001	28604000	LINC02758
11	30228001	30229000	ARL14EP-DT;FSHB
11	30955001	30956000	DCDC1
11	31754001	31755000	ELP4
11	32945001	32946000	QSER1
11	33429001	33430000	KIAA1549L
11	33593001	33594000	KIAA1549L
11	34137001	34138000	NAT10
11	35240001	35241000	CD44
11	35498001	35499000	PAMR1
11	35976001	35977000	LDLRAD3;LOC100420018
11	36178001	36179000	LDLRAD3
11	36301001	36302000	PRR5L
11	36350001	36351000	PRR5L
11	36368001	36369000	PRR5L
11	36424001	36425000	PRR5L
11	39737001	39738000	LOC105376637
11	44230001	44231000	EXT2
11	44508001	44509000	LOC105376645;LOC105376646
11	44912001	44913000	TSPAN18
11	45178001	45179000	PRDM11
11	45393001	45396000	LINC02687;LINC02687;LINC02687
11	46097001	46098000	PHF21A
11	46203001	46204000	LINC02710
11	46248001	46249000	LINC02489
11	46320001	46321000	CREB3L1
11	46377001	46378000	DGKZ;MIR4688;MDK;CHRM4
11	47151001	47152000	C11orf49;LOC107984329
11	47229001	47230000	DDB2;ACP2
11	47341001	47342000	MYBPC3
11	47358001	47359000	MYBPC3;SPI1
11	47468001	47469000	CELF1
11	49324001	49325000	LOC729960
11	49779001	49780000	GRM5P1
11	56639001	56640000	OR5M12P;OR5AP1P;OR5AP2
11	57179001	57180000	LOC105369309;LRRC55
11	57522001	57523000	SLC43A1;RN7SL605P;TIMM10
11	57664001	57665000	CLP1;ZDHHC5
11	57697001	57698000	ZDHHC5;MED19
11	58896001	58897000	GLYATL2;GLYATL1P2;GLYATL1

11	59769001	59770000	STX3
11	60938001	60939000	TMEM132A;SLC15A3
11	60951001	60952000	SLC15A3
11	61107001	61108000	CD5
11	61339001	61340000	DDB1;TKFC;CYB561A3
11	61416001	61417000	CPSF7
11	61496001	61497000	PPP1R32;LOC105369329
11	61517001	61518000	MIR4488;LRRC10B;SYT7
11	61550001	61551000	SYT7
11	61592001	61593000	SYT7;LOC105369331;LOC101927495
11	61774001	61776000	MYRF;MYRF
11	61962001	61963000	BEST1;LOC107984334;FTH1;LOC399900
11	62405001	62406000	ASRGL1;LOC107984335;LOC102723765
11	62472001	62473000	AHNAK
11	62489001	62490000	AHNAK
11	62571001	62572000	EEF1G;MIR6747;TUT1
11	63456001	63459000	PLAAT5;PLAAT5;PLAAT5
11	63816001	63817000	SPINDOC
11	64010001	64011000	MACROD1;LOC101927673
11	64026001	64027000	MACROD1;FLRT1
11	64351001	64352000	CCDC88B;MIR7155;RPS6KA4
11	64581001	64582000	SLC22A11;SLC22A12
11	64629001	64630000	NRXN2
11	64634001	64635000	NRXN2;NRXN2-AS1
11	64671001	64672000	NRXN2
11	64700001	64701000	NRXN2
11	64799001	64800000	MAP4K2;MEN1
11	65189001	65191000	LOC728975;CAPN1;LOC728975;CAPN1
11	65318001	65319000	LOC105369344;CDC42EP2
11	65548001	65549000	SCYL1;LTBP3
11	66214001	66215000	PACS1
11	66279001	66280000	RAB1B;LOC107984340;CNIH2;YIF1A
11	66302001	66303000	TMEM151A
11	66324001	66325000	CD248;RIN1
11	66390001	66391000	BRD9P1;RNU1-84P
11	66431001	66432000	NPAS4;MRPL11
11	66575001	66576000	CTSF
11	67035001	67036000	SYT12;MIR6860
11	67368001	67369000	LOC100130987;RN7SKP239;CLCF1
11	67668001	67669000	ALDH3B2
11	67762001	67763000	LOC100420907;LOC105369358
11	67764001	67765000	LOC100420907;LOC105369358
11	68422001	68423000	LRP5
11	68580001	68581000	PPP6R3
11	68681001	68682000	LOC107984343;GAL
11	68687001	68688000	LOC107984343;GAL
11	68691001	68692000	LOC107984343;GAL
11	68720001	68721000	TESMIN
11	68741001	68742000	TESMIN
11	69064001	69065000	TPCN2
11	69076001	69077000	TPCN2;MIR3164
11	69167001	69168000	LOC338694;SMIM38;LOC105369366
11	70032001	70033000	ANO1
11	70058001	70059000	ANO1;LINC02753;LOC105369371
11	70060001	70062000	ANO1;LINC02753;LOC105369371;ANO1;LINC02753;LOC105369371
11	70065001	70066000	ANO1;LINC02753;LOC105369371;LINC02584;RNU6-1175P
11	70168001	70169000	ANO1;LOC101928473
11	70208001	70209000	FADD
11	70283001	70284000	PPFIA1;H2AZP4;MIR548K
11	70632001	70633000	SHANK2;SHANK2-AS1
11	70732001	70733000	SHANK2
11	70862001	70863000	SHANK2;SHANK2-AS3;MIR3664
11	71242001	71243000	SHANK2
11	71520001	71521000	S100A11P3;KRTAP5-7
11	72114001	72115000	LRTOMT;TOMT;ANAPC15
11	72241001	72242000	INPPL1;PHOX2A;LOC220077
11	72568001	72569000	LINC01537;PDE2A
11	72630001	72631000	PDE2A;RNU7-105P
11	72639001	72640000	PDE2A
11	72699001	72700000	ARAP1;ARAP1-AS2;RPS12P20
11	73487001	73488000	FAM168A
11	74021001	74022000	C2CD3
11	75131001	75132000	OR2AT1P;LOC107984358
11	75346001	75347000	ARRB1
11	75500001	75501000	GDPD5;LOC105369389
11	75581001	75582000	SERPINH1;LOC105369391;MAP6
11	75610001	75611000	MAP6
11	76052001	76053000	UVRAG
11	76176001	76177000	WNT11
11	77037001	77038000	B3GNT6;LOC105369399
11	77170001	77171000	MYO7A
11	77289001	77290000	GDPD4
11	77303001	77304000	GDPD4;TOMM20P1
11	78085001	78086000	NDUFC2-KCTD14;NDUFC2;LOC105369401
11	78090001	78091000	LOC105369401;ALG8
11	78729001	78730000	TENM4
11	78782001	78783000	TENM4

11	79307001	79308000	TENM4
11	79350001	79351000	TENM4
11	79373001	79374000	TENM4
11	82890001	82891000	PRCP
11	83448001	83449000	DLG2
11	84074001	84075000	DLG2
11	86326001	86327000	HIKESHI;RN7SL225P;LOC105369421
11	87009001	87010000	FZD4-DT;LOC105369422
11	89713001	89714000	TRIM77
11	92397001	92398000	FAT3
11	94541001	94542000	C11orf97;FUT4;PIWIL4-AS1
11	94643001	94644000	PIWIL4-AS1;LINC02700
11	94646001	94647000	PIWIL4-AS1;LINC02700
11	95021001	95023000	LOC100420801;KDM4E;LOC100420802;LOC100420801;KDM4E;LOC100420802
11	96500001	96501000	JRKL-AS1;LINC02737
11	99432001	99433000	CNTN5
11	99816001	99817000	CNTN5
11	100131001	100132000	CNTN5
11	101219001	101220000	LOC100420510
11	102222001	102223000	YAP1
11	102272001	102273000	LOC105369460
11	102291001	102292000	LOC105369460;RPS6P17
11	102373001	102374000	BIRC2
11	102729001	102730000	MMP8
11	107444001	107445000	CWF19L2
11	107817001	107818000	SLC35F2
11	108941001	108942000	DDX10
11	111457001	111458000	BTG4;LOC107984386;LOC644277
11	112358001	112359000	LINC02762;RNU6-44P;LOC107984387
11	112476001	112477000	LOC107984389;LINC02763
11	113208001	113209000	NCAM1
11	113332001	113333000	TTC12
11	113415001	113416000	DRD2
11	113648001	113649000	LOC107984390
11	113836001	113837000	USP28
11	114225001	114226000	ZBTB16
11	115526001	115527000	LOC105369511
11	115572001	115573000	RPL12P46
11	115575001	115576000	RPL12P46
11	115709001	115710000	LINC02698
11	115731001	115732000	LINC02698;LOC101928985
11	115904001	115905000	LINC02698
11	116638001	116639000	LOC107984372;LINC02702
11	117213001	117214000	TAGLN;PCSK7
11	117440001	117441000	DSCAML1
11	117460001	117461000	DSCAML1
11	117486001	117487000	DSCAML1
11	117501001	117502000	DSCAML1
11	117605001	117606000	DSCAML1
11	117635001	117636000	DSCAML1
11	117639001	117640000	DSCAML1
11	117713001	117714000	DSCAML1
11	117810001	117812000	DSCAML1;LOC107984394;FXYD6-FXYD2;FXYD2;DSCAML1;LOC107984394;FXYD6-FXYD2;FXYD2
11	117880001	117881000	FXYD6-FXYD2;FXYD6
11	118052001	118053000	SMIM35
11	118718001	118719000	LOC105369519
11	119430001	119432000	USP2-AS1;THY1;USP2-AS1;THY1
11	119460001	119461000	USP2-AS1
11	119495001	119496000	USP2-AS1
11	119668001	119669000	NECTIN1
11	119734001	119735000	NECTIN1;NECTIN1-DT
11	120184001	120185000	LOC105369530
11	120266001	120267000	POU2F3;LOC649133;LOC105369531
11	120508001	120509000	GRIK4
11	120562001	120563000	GRIK4
11	120625001	120626000	GRIK4;ELOCP22
11	120692001	120693000	GRIK4
11	120783001	120784000	GRIK4
11	120892001	120893000	GRIK4;LOC101929227
11	120902001	120903000	GRIK4;LOC101929227
11	121485001	121486000	SORL1;LOC105369535
11	121638001	121641000	SORL1;SORL1;SORL1
11	122232001	122233000	MIR100HG
11	122551001	122552000	TRK-TTT2-1
11	122989001	122990000	BSX
11	123324001	123325000	LINC02727
11	123448001	123449000	GRAMD1B
11	123464001	123465000	GRAMD1B
11	123469001	123470000	GRAMD1B
11	123868001	123869000	OR6M3P
11	124001001	124002000	OR10G6;OR10G5P
11	125195001	125196000	PKNOX2
11	125208001	125209000	PKNOX2
11	125355001	125356000	PKNOX2
11	125393001	125394000	PKNOX2
11	126002001	126003000	CDON
11	126435001	126436000	KIRREL3

11	126457001	126458000	KIRREL3
11	126478001	126479000	KIRREL3
11	126719001	126720000	KIRREL3;LOC105369561
11	126730001	126731000	KIRREL3;LOC105369561
11	127096001	127097000	LOC101929473
11	127278001	127279000	LINC02712
11	128553001	128554000	ETS1
11	128561001	128562000	ETS1;LOC105369565
11	128615001	128616000	LOC105369568
11	128619001	128620000	LOC105369568
11	129984001	129985000	PRDM10
11	130255001	130256000	ZBTB44
11	130398001	130399000	ZBTB44-DT;ADAMTS8
11	130400001	130401000	ZBTB44-DT;ADAMTS8
11	130467001	130468000	ADAMTS15
11	130535001	130536000	LOC105369574;BAK1P2
11	130566001	130567000	LOC105369574
11	130568001	130569000	LOC105369574
11	130985001	130986000	LOC105369577
11	131455001	131456000	NTM
11	131826001	131827000	NTM
11	131865001	131866000	NTM
11	131868001	131869000	NTM;LOC107984413
11	131926001	131927000	NTM
11	132108001	132109000	NTM
11	132291001	132292000	NTM;NTM-IT
11	132469001	132470000	OPCML
11	132624001	132625000	OPCML
11	132824001	132825000	OPCML
11	132978001	132979000	OPCML;LOC105369580
11	133326001	133327000	OPCML
11	133344001	133345000	OPCML
11	133437001	133438000	OPCML
11	133933001	133934000	IGSF9B
11	134032001	134033000	LINC02730;LINC02731
11	134416001	134417000	B3GAT1
11	134440001	134441000	B3GAT1-DT
11	134716001	134717000	LINC02706
12	19001	20000	DDX11L8;WASH8P
12	39001	40000	WASH8P;LOC107987170;FAM138D
12	82001	84000	IQSEC3;IQSEC3
12	99001	100000	IQSEC3;LOC105369593
12	132001	133000	IQSEC3;LOC574538
12	149001	150000	IQSEC3;LOC574538
12	414001	415000	CCDC77
12	448001	449000	CCDC77
12	463001	464000	B4GALNT3
12	545001	546000	B4GALNT3
12	1121001	1122000	ERC1
12	1552001	1553000	WNT5B;FBXL14
12	1930001	1931000	CACNA2D4;LOC112268103;LINC00940
12	2065001	2066000	CACNA1C
12	2125001	2126000	CACNA1C;LOC107984131
12	2263001	2264000	CACNA1C;CACNA1C-IT3
12	2416001	2417000	CACNA1C
12	2454001	2455000	CACNA1C
12	2461001	2462000	CACNA1C
12	2552001	2553000	CACNA1C
12	2576001	2577000	CACNA1C
12	2611001	2612000	CACNA1C
12	2621001	2622000	CACNA1C
12	3005001	3006000	TEAD4
12	3043001	3044000	TEAD4
12	3192001	3193000	TSPAN9
12	3249001	3250000	TSPAN9
12	3447001	3448000	PRMT8
12	3473001	3474000	PRMT8;RPS26P44;THCAT155
12	3508001	3509000	PRMT8
12	3645001	3646000	CRACR2A
12	3751001	3752000	CRACR2A;LOC107984495
12	3920001	3921000	PARP11-AS1;LOC105369608
12	4023001	4024000	LOC105369611
12	4583001	4584000	DYRK4
12	4661001	4662000	NDUFA9
12	5261001	5262000	LOC105369617
12	5278001	5279000	LOC105369617
12	5562001	5563000	ANO2
12	5604001	5605000	ANO2
12	5621001	5622000	ANO2
12	5693001	5694000	ANO2
12	5706001	5707000	ANO2
12	5740001	5741000	ANO2
12	5849001	5850000	ANO2
12	5859001	5861000	ANO2;ANO2
12	5965001	5966000	VWF
12	5980001	5981000	VWF
12	6047001	6048000	VWF;SNORA120

12	6464001	6465000	TAPBPL;VAMP1
12	6662001	6663000	ING4;ZNF384
12	6694001	6695000	ZNF384;PIANP
12	6748001	6749000	MLF2
12	6770001	6771000	PTMS;LAG3
12	6831001	6832000	GPR162;P3H3;GNB3
12	6948001	6949000	ATN1;C12orf57;RNU7-1;PTPN6
12	7142001	7143000	CLSTN3
12	7159001	7160000	CLSTN3
12	7996001	7997000	RPS20P28
12	8128001	8129000	CLEC4A;POU5F1P3
12	8279001	8280000	LOC112268090
12	8292001	8293000	LOC112268090
12	8662001	8663000	MFAP5
12	8924001	8926000	PHC1;PHC1
12	9093001	9094000	KLRG1;A2M
12	9269001	9270000	LINC00987
12	9328001	9329000	LOC107984489;LOC105369649
12	9379001	9380000	LINC02367;LOC101928030
12	9715001	9716000	LINC02390;CLECL1
12	9963001	9964000	CLEC12A-AS1;CLEC12A
12	9966001	9967000	CLEC12A
12	10336001	10337000	KLRD1;LINC02617
12	10338001	10339000	KLRD1;LINC02617
12	10368001	10369000	LINC02598;KLRK1-AS1;KLRC4-KLRK1;KLRK1
12	10792001	10793000	TAS2R7
12	11467001	11468000	LOC440084
12	11501001	11502000	IQSEC3P2
12	11809001	11810000	ETV6
12	12047001	12049000	BCL2L14;BCL2L14
12	12060001	12061000	BCL2L14
12	12063001	12064000	BCL2L14
12	12069001	12070000	BCL2L14
12	12078001	12079000	BCL2L14
12	12294001	12295000	RNU6-318P
12	13032001	13033000	GPRC5D-AS1
12	13530001	13531000	GRIN2B
12	13856001	13857000	GRIN2B
12	13881001	13882000	GRIN2B
12	13959001	13960000	GRIN2B
12	15316001	15317000	PTPRO
12	19640001	19641000	LOC101928387
12	20090001	20091000	LINC02398;LOC105369685
12	21316001	21317000	SLCO1A2
12	22113001	22114000	SULT6B2P
12	22492001	22493000	C2CD5
12	24166001	24167000	SOX5
12	24382001	24383000	SOX5
12	24650001	24651000	LOC105369698;RPL21P102
12	24714001	24715000	LOC105369698;RN7SL38P
12	25120001	25121000	DNAI7
12	25167001	25169000	DNAI7;DNAI7
12	25260001	25261000	KRAS
12	25394001	25395000	RNU4-67P
12	26142001	26143000	SSPN
12	26630001	26631000	ITPR2
12	27079001	27080000	C12orf71
12	27534001	27535000	PPFIBP1
12	27712001	27713000	MRPS35-DT;HMGB1P49;MRPS35
12	29274001	29275000	FAR2;LOC100506606
12	29550001	29551000	TMTC1
12	30044001	30045000	LOC105369715
12	30058001	30059000	LOC105369715
12	30927001	30928000	TSPAN11
12	31364001	31365000	LINC02387
12	31896001	31897000	LINC02422;RPLP2P4
12	31898001	31899000	RPLP2P4
12	32648001	32649000	FGD4
12	32814001	32815000	PKP2
12	34217001	34218000	DUX4L27
12	41048001	41049000	CNTN1
12	41244001	41245000	PDZRN4
12	42031001	42032000	LOC105378247
12	42338001	42339000	PPHLN1
12	45308001	45309000	ANO6
12	45942001	45943000	SCAF11
12	46487001	46488000	LOC100288798
12	47139001	47140000	PCED1B;IFITM3P6
12	48111001	48113000	SENP1;PFKM;SENP1;PFKM
12	48324001	48325000	H1-7;ZNF641
12	48472001	48474000	C12orf54;ANP32D;C12orf54;ANP32D
12	49146001	49147000	LOC105369760
12	49253001	49254000	TUBA1C
12	49387001	49388000	SPATS2;LOC100335030
12	49402001	49404000	SPATS2;LOC100335030;SPATS2
12	49541001	49542000	KCNH3;LOC105369761
12	49577001	49578000	MCRS1;FAM186B

12	49579001	49580000	FAM186B
12	49892001	49893000	FAIM2
12	50068001	50069000	ASIC1
12	50105001	50107000	SMARCD1;GPD1;COX14;SMARCD1;GPD1;COX14
12	50628001	50629000	DIP2B;RNU6-769P
12	51914001	51915000	ACVRL1
12	52087001	52088000	ATG101;SMIM41;OR7E47P;LOC112268096
12	52313001	52314000	KRT86;KRT83
12	52611001	52612000	KRT72;KRT73;KRT73-AS1
12	53520001	53521000	ATF7-NPFF;ATF7;LOC100652999
12	53684001	53685000	ATP5MC2
12	53710001	53711000	CALCOCO1
12	53724001	53725000	CALCOCO1;LOC105369774
12	54161001	54162000	SMUG1;LOC102724030
12	54227001	54228000	CBX5;MIR3198-2
12	54513001	54514000	NCKAP1L
12	54932001	54933000	LOC105369778
12	55840001	55841000	DNAJC14;TMEM198B;MMP19
12	56021001	56022000	IKZF4;LOC105369781;LOC105369780
12	56180001	56181000	SMARCC2;TRS-CGA4-1;LOC107984468
12	56235001	56236000	NABP2;SLC39A5;ANKRD52
12	56766001	56767000	HSD17B6
12	57200001	57201000	LRP1;MIR1228
12	57223001	57224000	LRP1;NXPH4;SHMT2
12	57464001	57465000	INHBE;GLI1;ARHGAP9
12	57497001	57498000	ARHGAP9;MARS1
12	57588001	57589000	KIF5A;PIP4K2C
12	57597001	57598000	PIP4K2C;DTX3
12	57700001	57701000	OS9
12	57728001	57729000	OS9;AGAP2;AGAP2-AS1
12	57862001	57863000	LOC283387
12	57871001	57872000	LOC283387
12	57897001	57898000	LOC283387;LOC101927608
12	57950001	57951000	ATP23
12	58105001	58106000	LOC105369784
12	58686001	58687000	LINC02388;LOC100506869
12	59106001	59107000	LRIG3-DT
12	59748001	59749000	SLC16A7
12	61592001	61593000	DUX4L52
12	62760001	62761000	PPM1H;GAPDHP44
12	63808001	63809000	RXYLT1;RXYLT1-AS1
12	65516001	65517000	LOC100507065
12	66350001	66351000	HELB;GRIP1
12	66497001	66498000	GRIP1
12	67031001	67032000	GRIP1
12	68052001	68053000	LOC107984526
12	68346001	68348000	LOC105369819;LOC105369819
12	68962001	68963000	CPM;PRELID2P1
12	69258001	69259000	CPSF6
12	69832001	69833000	RAB3IP;MYRFL
12	69867001	69868000	MYRFL
12	70550001	70551000	PTPRB;LOC105369828
12	72208001	72209000	TRHDE
12	72519001	72520000	TRHDE
12	72552001	72553000	TRHDE
12	73004001	73005000	LOC105369838
12	75741001	75742000	LOC105369844
12	76295001	76296000	LNCOG
12	77655001	77656000	NAV3
12	78024001	78025000	NAV3
12	79944001	79945000	PPP1R12A;PPP1R12A-AS1
12	80786001	80788000	LIN7A;LIN7A
12	81125001	81126000	ACSS3;LOC107984535
12	81975001	81976000	LINC02426
12	83094001	83095000	TMTC2
12	85411001	85412000	LINC02820
12	85822001	85823000	RASSF9
12	88138001	88139000	CEP290;TMTC3
12	91619001	91620000	LOC105369896
12	92821001	92822000	EEA1
12	92922001	92923000	EEA1
12	93277001	93278000	LOC643339
12	93324001	93325000	LOC643339;LOC105369909
12	93352001	93353000	LOC643339
12	94187001	94188000	PLXNC1
12	94193001	94194000	PLXNC1
12	95451001	95452000	LOC107984545
12	95750001	95751000	NTN4
12	96232001	96233000	ELK3
12	96256001	96257000	ELK3;LOC107984546
12	96572001	96573000	CFAP54
12	97480001	97481000	RMST
12	98747001	98748000	ANKS1B
12	99692001	99693000	ANKS1B
12	99715001	99717000	ANKS1B;ANKS1B
12	99720001	99721000	ANKS1B
12	100243001	100244000	DEPDC4

12	101330001	101331000	UTP20
12	101915001	101916000	DRAM1
12	102499001	102500000	LOC105369944
12	102577001	102578000	LOC105369944
12	103049001	103050000	C12orf42
12	103492001	103493000	C12orf42;LOC105369945
12	103709001	103710000	STAB2
12	103903001	103904000	TTC41P
12	104272001	104273000	TXNRD1;RPL18AP3
12	104356001	104357000	TXNRD1
12	105377001	105378000	C12orf75;LOC105369957
12	105380001	105381000	C12orf75;LOC105369957
12	105386001	105387000	LOC105369957
12	105413001	105414000	LOC105369957
12	106484001	106485000	POLR3B
12	106600001	106601000	LOC100287944;RFX4
12	106707001	106708000	LOC100287944;RFX4
12	108574001	108575000	ISCU
12	108597001	108598000	TMEM119
12	108813001	108814000	SSH1;LOC101929204
12	108925001	108926000	SVOP;RNU6-361P
12	109511001	109513000	UBE3B;UBE3B
12	110405001	110406000	ANAPC7
12	110409001	110411000	ANAPC7;ANAPC7
12	110697001	110698000	HVCN1
12	111136001	111137000	CUX2
12	111185001	111186000	CUX2
12	111188001	111189000	CUX2
12	111253001	111254000	CUX2
12	111297001	111298000	CUX2;LOC105369983;MIR6760
12	111580001	111581000	ATXN2;IFITM3P5
12	112484001	112485000	PTPN11
12	112800001	112801000	RPH3A
12	113076001	113077000	DTX1
12	113389001	113390000	PLBD2;SDS
12	113439001	113440000	SDSL
12	113665001	113666000	LOC105369991
12	113831001	113832000	RBM19
12	113842001	113843000	RBM19
12	114830001	114832000	LOC107984437;LOC107984437
12	115012001	115013000	LOC102723639
12	115380001	115381000	LOC105370003
12	115711001	115712000	LOC105370003;RN7SL865P
12	115737001	115738000	LOC105370003
12	115951001	115952000	MED13L
12	116360001	116361000	LOC105370005
12	116746001	116747000	SPRING1;RNFT2;RPL21P105
12	116821001	116822000	RNFT2
12	117020001	117021000	FBXW8
12	117161001	117162000	FBXO21
12	117178001	117179000	FBXO21
12	117370001	117371000	NOS1
12	117630001	117631000	KSR2
12	117794001	117795000	KSR2
12	117957001	117958000	KSR2
12	118156001	118157000	TAOK3
12	119144001	119145000	SRRM4;LOC112268101
12	119544001	119545000	CCDC60;LOC105370027
12	120074001	120075000	BICDL1
12	120115001	120116000	RAB35
12	120233001	120234000	PXN
12	120255001	120256000	PXN;RPS20P31
12	120547001	120549000	RNF10;RNF10
12	120591001	120592000	POP5;RPL11P5
12	120622001	120623000	CABP1-DT
12	121012001	121013000	HNF1A;C12orf43;OASL
12	121563001	121564000	KDM2B
12	121750001	121751000	TMEM120B
12	122019001	122020000	BCL7A
12	122636001	122637000	KNTC1
12	123466001	123467000	SNRNP35;RILPL1
12	123536001	123537000	RILPL1;MIR3908
12	123809001	123810000	DNAH10
12	124194001	124195000	ZNF664-RFLNA
12	124238001	124239000	ZNF664-RFLNA
12	124258001	124259000	ZNF664-RFLNA
12	124478001	124479000	NCOR2
12	124532001	124533000	NCOR2
12	124896001	124897000	LOC105370051;RNU6-927P
12	124929001	124930000	TRA-TGC3-2;TRD-GTC2-9;TRF-GAA1-4;RPL22P19;TRD-GTC2-10;TRA-TGC4-1
12	125120001	125121000	AACS
12	125494001	125495000	TMEM132B
12	126181001	126182000	LINC02467
12	127154001	127155000	LOC107984449;LOC107984450;LOC105370062
12	129004001	129005000	LOC105370073;NLRP9P1
12	129023001	129024000	LOC105370073;NLRP9P1
12	129025001	129026000	LOC105370073;NLRP9P1

12	129156001	129157000	TMEM132D
12	129443001	129444000	TMEM132D
12	129746001	129747000	TMEM132D
12	130600001	130601000	RIMBP2
12	130616001	130617000	RIMBP2
12	130627001	130628000	RIMBP2;LOC105370079
12	130761001	130762000	LOC105370080;LOC107984451
12	130771001	130772000	LOC105370080;LOC107984451
12	130775001	130776000	LOC107984451
12	130957001	130958000	ADGRD1
12	130982001	130983000	ADGRD1;ADGRD1-AS1
12	131049001	131050000	ADGRD1
12	131071001	131072000	ADGRD1;LOC107984452
12	131258001	131259000	LOC105370082
12	131260001	131261000	LOC105370082
12	131303001	131304000	RPS6P20;LOC100128002;LINC02415;LOC105370083;RNA5SP376
12	131656001	131657000	LINC02414;LOC105370086;RNA5SP377;LOC100996701
12	131682001	131684000	LOC105370087;LOC105370087
12	131857001	131859000	MMP17;MMP17
12	132054001	132055000	EP400
12	132095001	132096000	EP400P1
12	132314001	132315000	GALNT9
12	132635001	132636000	POLE
12	132664001	132665000	POLE
12	132897001	132898000	CHFR;LOC101928530;LOC647503
12	132953001	132954000	ZNF605
12	133002001	133003000	ZNF26;RNU4ATAC12P
13	18257001	18258000	LOC101060145;GGT4P
13	18303001	18304000	KMT5AP1
13	19664001	19665000	MPHOSPH8;PSPC1
13	20130001	20131000	LOC105370101;GJA3
13	20151001	20152000	GJA3
13	20397001	20398000	CRYL1
13	20700001	20701000	IFT88;IL17D
13	21489001	21490000	H2BP6;MICU2
13	21690001	21691000	FGF9
13	22238001	22239000	LINC00540
13	23383001	23384000	SACS;RPLP1P13
13	23954001	23955000	ANKRD20A19P;LOC105370115
13	25152001	25153000	AMER2
13	25203001	25204000	RPL23AP69
13	25358001	25359000	NUP58;ELOBP1
13	25570001	25571000	ATP8A2
13	25576001	25577000	ATP8A2
13	25876001	25877000	ATP8A2
13	26406001	26407000	CDK8
13	26591001	26592000	WASF3
13	26659001	26660000	WASF3
13	26663001	26664000	WASF3
13	27026001	27027000	RPS20P32
13	27100001	27101000	USP12
13	27183001	27184000	USP12-AS2;LINC02340
13	27338001	27339000	RNU6-70P
13	27594001	27595000	LNX2
13	27812001	27813000	PLUT
13	28473001	28474000	FLT1
13	28500001	28501000	FLT1
13	29161001	29162000	MTUS2
13	29200001	29201000	MTUS2
13	29244001	29245000	MTUS2
13	29492001	29493000	MTUS2;MTUS2-AS1
13	29703001	29704000	LOC105370139
13	30167001	30168000	LINC00385
13	30339001	30340000	LINC00426
13	30542001	30543000	HMGB1;RBM22P2;MFAP1P1
13	30665001	30666000	USPL1;TRN-GTT2-4
13	30671001	30672000	TRN-GTT2-4
13	31055001	31056000	LOC102723392
13	31732001	31733000	RXFP2
13	31852001	31853000	LOC105370152;EEF1DP3
13	32077001	32078000	FRY
13	32383001	32384000	BRCA2;IFIT1P1
13	32638001	32639000	PDS5B
13	32893001	32894000	LINC00423
13	33020001	33021000	KL
13	33451001	33452000	STARD13
13	33881001	33882000	RFC3
13	34507001	34508000	LINC02343;LINC00457
13	35706001	35707000	LINC00445
13	35796001	35797000	DCLK1
13	35920001	35921000	DCLK1
13	36317001	36318000	SPART
13	36850001	36851000	SMAD9
13	37714001	37715000	TRPC4
13	37971001	37973000	LINC02334;LINC02334
13	39005001	39006000	PROSER1
13	39013001	39014000	PROSER1

13	39431001	39432000	LHFPL6
13	39458001	39459000	LHFPL6
13	39696001	39697000	COG6
13	40212001	40213000	LINC00548
13	40216001	40217000	LINC00548;RPL17P51;RNY3P9
13	40338001	40339000	LINC00598
13	40736001	40737000	MIR320D1;MRPS31
13	40830001	40831000	TPTE2P5
13	41725001	41726000	VWA8
13	42870001	42871000	LINC00428
13	43940001	43941000	LOC107984576
13	44362001	44363000	LOC105370297
13	45050001	45051000	LOC105370188;LOC107984596;RN7SKP3
13	45133001	45134000	GTF2F2;RN7SKP4;RNU6-69P
13	45229001	45230000	GTF2F2;LOC105370190
13	45506001	45507000	COG3
13	45967001	45968000	ZC3H13
13	46198001	46199000	LOC105370192;LOC107984578
13	46402001	46403000	RNU6-68P
13	46635001	46636000	LRCH1
13	46637001	46638000	LRCH1
13	46660001	46661000	LRCH1
13	46727001	46728000	LRCH1
13	48141001	48142000	LOC105370198;POLR2KP2
13	48192001	48193000	LOC105370198
13	48427001	48428000	RB1;LPAR6
13	48492001	48493000	RCBTB2
13	48774001	48775000	PSME2P2
13	48871001	48872000	LOC107984559
13	49157001	49158000	FNDC3A
13	49347001	49348000	CAB39L
13	49716001	49717000	KPNA3
13	50324001	50325000	DLEU1
13	50787001	50788000	DLEU7;LOC107984567
13	50944001	50945000	RNASEH2B
13	51071001	51072000	GUCY1B2
13	51170001	51171000	C13orf42;RPL5P31
13	51173001	51174000	C13orf42
13	51214001	51215000	FAM124A
13	51293001	51294000	FAM124A
13	51972001	51973000	ATP7B;FABP5P2
13	54127001	54128000	LINC00458
13	54129001	54130000	LINC00458
13	54131001	54133000	LINC00458;LINC00458
13	59506001	59507000	LOC107984625
13	63218001	63219000	LINC00376
13	63730001	63731000	LINC00395;LOC105370235;OR7E156P
13	66227001	66228000	MIR4704
13	73784001	73785000	KLF12
13	74240001	74241000	LINC00402
13	75007001	75008000	LOC107984620
13	75338001	75339000	TBC1D4
13	75398001	75399000	TBC1D4
13	75694001	75695000	LMO7;FAM204CP
13	75860001	75861000	LMO7
13	77802001	77803000	LOC112268121
13	77933001	77937000	EDNRB;EDNRB;EDNRB;EDNRB
13	79361001	79362000	RBM26
13	80106001	80107000	LOC105370275
13	80716001	80717000	LOC105370278
13	83909001	83910000	VENTXP2
13	83913001	83914000	VENTXP2
13	91748001	91749000	GPC5
13	92398001	92399000	GPC5
13	93609001	93610000	GPC6
13	93848001	93849000	GPC6;GPC6-AS2
13	94238001	94239000	GPC6
13	94502001	94503000	DCT
13	94557001	94558000	DCT;TRF-GAA1-5
13	94792001	94793000	LOC101927284
13	95025001	95026000	RNU6-62P;ABCC4
13	95122001	95123000	ABCC4
13	95632001	95633000	DZIP1
13	95693001	95694000	DNAJC3;MTND5P2;MTND6P18;MTCYBP3
13	96158001	96159000	HS6ST3
13	96184001	96185000	HS6ST3
13	97642001	97643000	LOC105370324
13	98330001	98331000	FARP1;LOC105370327
13	98370001	98371000	FARP1
13	98462001	98463000	FARP1;STK24
13	98816001	98817000	DOCK9
13	98848001	98849000	DOCK9
13	99206001	99207000	UBAC2-AS1;UBAC2;RN7SKP9;H2AZP3
13	99315001	99316000	UBAC2;GPR183
13	99525001	99527000	TM9SF2;TM9SF2;RNY3P6
13	99747001	99748000	CLYBL;LOC101927437
13	100265001	100266000	PCCA

13	100482001	100483000	PCCA;PCCA-AS1;LOC107984574
13	101001001	101002000	NALCN-AS1
13	101224001	101225000	NALCN
13	101255001	101256000	NALCN
13	102353001	102354000	FGF14;FGF14-IT1
13	102867001	102868000	BIVM-ERCC5;ERCC5
13	102875001	102876000	BIVM-ERCC5;ERCC5;METTL21EP
13	106592001	106593000	LOC105370348
13	106775001	106776000	LOC105370349
13	107449001	107450000	NALF1
13	108592001	108593000	MYO16
13	108633001	108634000	MYO16
13	108714001	108715000	MYO16
13	109085001	109086000	MYO16
13	109796001	109797000	IRS2;LOC105370360
13	110108001	110109000	RN7SL783P
13	110290001	110291000	COL4A1
13	110369001	110370000	COL4A2
13	110407001	110408000	COL4A2
13	110564001	110565000	RAB20
13	110679001	110680000	CARS2
13	110696001	110697000	CARS2
13	110770001	110771000	RPL21P107;LOC107984613
13	111131001	111132000	ARHGEF7
13	111600001	111601000	LINC02337
13	112667001	112668000	ATP11AUN
13	112673001	112674000	ATP11AUN
13	112713001	112715000	ATP11A;ATP11A
13	112722001	112723000	ATP11A
13	112828001	112829000	ATP11A
13	113112001	113113000	F7;F10
13	113281001	113282000	LDHBP1
13	113521001	113522000	TMCO3
13	113760001	113761000	LOC105377805;LINC00552;TMEM255B
13	113796001	113797000	TMEM255B
13	113976001	113977000	C13orf46;RASA3
13	114005001	114006000	RASA3
13	114007001	114008000	RASA3
13	114231001	114232000	CFAP97D2;CDC16
14	18880001	18881000	NEK2P1
14	19303001	19304000	LINC01297-DUXAP10-NBEAP6;DUXAP10;BMS1P17
14	20382001	20383000	TEP1
14	20701001	20702000	ANG;RNASE4
14	20726001	20727000	RANBP20P;EDDM3A;LOC107984671;EDDM3DP
14	20814001	20815000	LOC105370397
14	21022001	21023000	NDRG2;MIR6717;TPPP2;RNASE13
14	21052001	21053000	NDRG2;RNASE7;LOC105370398;RNASE8;ARHGEF40
14	21434001	21435000	CHD8;RN7SL650P
14	23021001	23022000	PSMB5
14	23046001	23047000	PSMB11;CDH24
14	23403001	23404000	MYH6;MYH7
14	23410001	23411000	MYH6;MYH7;MHRT;MIR208B
14	23531001	23532000	THTPA;ZFHX2
14	23533001	23534000	THTPA;ZFHX2
14	23550001	23551000	THTPA;ZFHX2;AP1G2
14	24267001	24268000	TGM1;RABGGTA;LOC102725044
14	24307001	24308000	LOC102725044;DHRS1;NOP9;CIDEB;LTB4R2;LTB4R
14	24422001	24423000	NYNRIN;KHNYN;CBLN3
14	25430001	25431000	LOC112268135;LOC401767
14	29680001	29681000	PRKD1
14	30424001	30425000	LOC112267868
14	32069001	32070000	ARHGAP5;ARHGAP5-AS1
14	32637001	32638000	AKAP6
14	33278001	33279000	NPAS3
14	33354001	33355000	NPAS3
14	33473001	33474000	NPAS3
14	33716001	33717000	NPAS3;SNORA89
14	34007001	34008000	LOC102724945
14	35267001	35268000	PRORP;RPL9P3
14	37355001	37356000	MIPOL1
14	39036001	39037000	SEC23A
14	39168001	39169000	TRAPPC6B;PNN
14	39657001	39658000	LOC105370461
14	39733001	39734000	LOC105370461
14	45282001	45283000	RNU6-552P;DNAJC19P9
14	45481001	45482000	LOC105370476
14	46274001	46275000	LINC00871
14	47106001	47107000	MDGA2
14	48201001	48202000	LOC101927483
14	48256001	48258000	LOC105370482;LOC105370482
14	48259001	48260000	LOC105370482
14	48262001	48263000	LOC105370482
14	49366001	49367000	ATP5MC2P2
14	49711001	49712000	KLHDC1
14	50090001	50091000	LINC01599;VCPKMT
14	50894001	50895000	ABHD12B
14	50896001	50897000	ABHD12B;PYGL

14	51093001	51094000	TRIM9
14	51335001	51336000	LINC00519;LINC00640;LOC105370495
14	51363001	51364000	LINC00640
14	51778001	51779000	LOC101927598;OR7E159P
14	52737001	52738000	PSMC6;STYX
14	53180001	53181000	LOC105370502
14	53914001	53915000	LOC107984676
14	53922001	53923000	LOC107984676
14	54648001	54649000	SAMD4A
14	56205001	56206000	PELI2;LOC107984656
14	56240001	56241000	PELI2
14	56664001	56665000	TMEM260
14	56674001	56675000	RPL36AP1
14	57035001	57036000	LOC440180
14	57607001	57608000	SLC35F4
14	58335001	58336000	ARID4A
14	59350001	59351000	DAAM1
14	59446001	59447000	GPR135
14	59748001	59749000	RTN1
14	61190001	61191000	PRKCH
14	61413001	61414000	PRKCH
14	61595001	61596000	FLJ22447
14	61771001	61772000	SNAPC1
14	62087001	62088000	SYT16
14	63209001	63210000	LOC105370531;RHOJ
14	63313001	63314000	GPHB5
14	63424001	63425000	PPP2R5E
14	63920001	63921000	SYNE2
14	64766001	64767000	SPTB
14	64858001	64859000	SPTB
14	64872001	64873000	SPTB;LOC105370534
14	65267001	65268000	RPL21P7;RPL36AP2
14	65791001	65792000	LOC107984641
14	66674001	66675000	GPHN
14	67398001	67399000	PLEK2
14	67464001	67465000	LOC105370542;TMEM229B
14	68534001	68535000	RAD51B
14	68610001	68611000	RAD51B;LOC107984016
14	68744001	68746000	LOC105370547;RNU6-921P;LOC107984647;LOC105370547;RNU6-921P;LOC107984647
14	68899001	68900000	ACTN1
14	68901001	68902000	ACTN1
14	69472001	69473000	SLC39A9;PLEKHD1
14	69817001	69818000	LOC100506358
14	70296001	70297000	LOC101928046
14	70347001	70348000	SYNJ2BP-COX16;COX16
14	70675001	70676000	TTC9
14	70903001	70905000	PCNX1;PCNX1
14	72000001	72001000	RGS6
14	72600001	72601000	RGS6;DPF3
14	72648001	72649000	DPF3
14	72728001	72729000	DPF3
14	73132001	73133000	RBM25;PSEN1
14	73236001	73237000	PAPLN;PAPLN-AS1;RNU6-419P
14	73811001	73812000	LOC100506476
14	74926001	74927000	RPS6KL1
14	75068001	75069000	ACYP1;ZC2HC1C
14	75583001	75584000	LOC102724153;FLVCR2
14	75767001	75768000	TTLL5
14	76504001	76505000	ESRRB
14	77215001	77217000	TMEM63C;TMEM63C
14	77226001	77228000	TMEM63C;TMEM63C
14	77297001	77298000	POMT2
14	77863001	77864000	ADCK1
14	77875001	77876000	ADCK1
14	78532001	78533000	NRXN3
14	78649001	78650000	NRXN3
14	79130001	79131000	NRXN3
14	79845001	79846000	NRXN3
14	80498001	80499000	CEP128
14	80714001	80715000	CEP128
14	80989001	80990000	TSHR;BHLHB9P1
14	81129001	81130000	TSHR;LOC101928462
14	81426001	81427000	STON2
14	84174001	84175000	MTND4P33;MTND6P27;MTCYBP27
14	86071001	86072000	LINC02328;LINC02316
14	87387001	87388000	LINC02296
14	87989001	87990000	GALC;RNU6-835P
14	88280001	88281000	KCNK10
14	88548001	88549000	PTPN21
14	88811001	88812000	RNU4-92P
14	89362001	89363000	FOXN3;LOC101928817
14	89400001	89401000	FOXN3
14	90562001	90563000	TTC7B
14	90619001	90620000	TTC7B
14	90742001	90743000	TTC7B
14	90815001	90816000	TTC7B;LINC02321
14	90874001	90875000	RPS6KA5

14	91044001	91045000	RPS6KA5
14	91103001	91104000	DGLUCY
14	91381001	91382000	CCDC88C;LOC107984673;LOC105370624
14	91910001	91911000	FBLN5
14	92042001	92044000	TRIP11;TRIP11
14	92048001	92049000	TRIP11;ATXN3
14	92128001	92129000	NDUFB1;CPSF2
14	92390001	92391000	SLC24A4
14	92521001	92522000	LOC105370707;RIN3
14	92656001	92657000	RIN3
14	92888001	92889000	LINC02833
14	92944001	92945000	CHGA;ITPK1
14	93560001	93562000	UNC79;UNC79
14	93770001	93771000	PRIMA1
14	94020001	94021000	CCDC197;OTUB2
14	94490001	94491000	SERPINA12
14	94620001	94621000	SERPINA3
14	95259001	95260000	CLMN
14	95525001	95526000	SYNE3;LOC101929107;LOC112268122;SNHG10;SCARNA13;GLRX5
14	95761001	95762000	LOC107984703;LOC105370644
14	95900001	95901000	TUNAR
14	96439001	96440000	AK7
14	96498001	96499000	AK7;PAPOLA-DT;PAPOLA
14	97538001	97539000	LINC02325
14	98239001	98240000	LOC105370656
14	98796001	98797000	LOC105370658
14	98964001	98965000	RPL3P4
14	98988001	98989000	LOC107984696
14	99130001	99131000	LOC105370659
14	99163001	99164000	BCL11B
14	99802001	99803000	EML1
14	100590001	100591000	BEGAIN
14	100686001	100687000	LOC105370668
14	100737001	100738000	DLK1
14	100828001	100829000	MEG3
14	100872001	100873000	MIR493;MIR337;MIR665;RTL1;MIR431;MIR433;MIR127
14	100905001	100906000	RTL1;MEG8;SNORD112;MIR370
14	101362001	101363000	LOC107984697;LOC105370671
14	101727001	101728000	LINC02320;LINC00239
14	101871001	101872000	PPP2R5C
14	102158001	102159000	WDR20
14	102230001	102231000	WDR20;MOK
14	102236001	102237000	MOK
14	102238001	102239000	MOK
14	102245001	102246000	MOK
14	102258001	102259000	MOK
14	102365001	102366000	CINP;TECPR2
14	102410001	102411000	TECPR2
14	102417001	102418000	TECPR2
14	102921001	102922000	TRAF3;AMN
14	103080001	103081000	LBHD2
14	103082001	103083000	LBHD2
14	104004001	104005000	TDRD9;RN7SL634P
14	104093001	104094000	ASPG
14	104592001	104593000	C14orf180;TMEM179
14	104655001	104656000	LINC02280
14	104664001	104665000	LINC02280
14	104672001	104673000	LINC02280;MIR4710
14	105318001	105319000	BRF1;PACS2
14	105521001	105522000	TMEM121;LOC105370697
14	105628001	105629000	IGH;MIR8071-1;IGHG4
14	105647001	105648000	IGH;MIR8071-2;IGHG2;LOC112268138;LOC105378187
14	105744001	105745000	IGH;IGHG1;LOC105378184
14	105746001	105747000	IGH;IGHG1;LOC105378184
14	105773001	105775000	IGH;LOC105378184;IGHG3;IGH;LOC105378184;IGHG3;ATP6V1G1P1
14	105866001	105867000	IGH;IGHM;MIR4539;MIR4507;MIR4538;MIR4537;IGHJ6;IGHJ3P;IGHJ5;IGHJ4;IGHJ3;IGHJ2P;IGHJ2;IGHJ1;IGHD7-27;IGHJ1P
14	105931001	105932000	IGH;FAM30A;IGHV6-1
14	105947001	105948000	IGH;IGHV6-1;IGHVII-1-1
14	106390001	106392000	IGH;IGHV3-35;IGHV3-36;IGHV3-37;IGH;IGHV3-35;IGHV3-36;IGHV3-37
15	20024001	20025000	FAM30B
15	20220001	20221000	RHPN2P1
15	20416001	20417000	HERC2P3
15	20441001	20442000	HERC2P3
15	20530001	20531000	LOC100996379;LOC112268160;GOLGA6L6
15	21311001	21312000	RNU6-1235P
15	22265001	22266000	LOC101928039;REREP3
15	22279001	22280000	REREP3
15	22470001	22471000	GOLGA6L22;LOC112268161;HERC2P7
15	22570001	22571000	HERC2P2
15	23439001	23440000	HERC2P6;GOLGA6L2
15	24687001	24688000	NPAP1
15	25903001	25904000	LINC02346
15	26649001	26650000	GABRB3
15	27265001	27266000	GABRG3
15	27270001	27271000	GABRG3;RNA5SP391
15	27319001	27320000	GABRG3
15	27382001	27383000	GABRG3

15	27397001	27398000	GABRG3
15	27455001	27456000	GABRG3
15	27770001	27772000	OCA2;OCA2
15	27816001	27817000	OCA2
15	27980001	27981000	OCA2
15	28294001	28295000	HERC2
15	28497001	28498000	MIR4509-3;ABCB10P4
15	28546001	28547000	LOC100631268;LOC100132202
15	28652001	28654000	HERC2P9;HERC2P9
15	28953001	28954000	APBA2
15	29064001	29065000	APBA2
15	29140001	29141000	FAM189A1
15	29177001	29178000	FAM189A1
15	29383001	29384000	FAM189A1
15	29846001	29847000	TJP1
15	30016001	30017000	NCAPGP2
15	30173001	30174000	LOC105370747
15	30424001	30425000	GOLGA8R;LOC101927788;LOC100288482;LOC105376704
15	31051001	31052000	TRPM1
15	31168001	31169000	TRPM1
15	31391001	31392000	KLF13;LOC105370939
15	31699001	31700000	OTUD7A
15	32155001	32156000	CHRNA7;LOC105370754;LOC102724078
15	32373001	32374000	LOC112268159
15	32491001	32492000	LOC101060588;LOC100653133;LOC107987215
15	32889001	32890000	FMN1
15	33033001	33034000	FMN1
15	33058001	33059000	FMN1
15	33359001	33360000	RYR3
15	33709001	33710000	RYR3
15	33873001	33874000	RYR3;AVEN
15	33946001	33947000	AVEN
15	33997001	33998000	AVEN;CHRM5
15	33999001	34000000	AVEN;CHRM5
15	34300001	34301000	SLC12A6
15	34350001	34351000	NOP10;NUTM1;LPCAT4
15	35240001	35242000	DPH6;ANP32AP1;DPH6;ANP32AP1
15	35547001	35548000	DPH6;DPH6-DT
15	36636001	36637000	CDIN1;TPST2P1
15	36938001	36939000	MEIS2
15	37000001	37001000	MEIS2
15	38263001	38264000	LOC107984760;SPRED1
15	39993001	39994000	EIF2AK4
15	40347001	40348000	CCDC9B;PHGR1;DISP2
15	40355001	40356000	PHGR1;DISP2
15	40881001	40882000	RHOV
15	40959001	40960000	CHAC1;LOC105370789
15	41628001	41629000	LOC105370791;MGA
15	42137001	42138000	PLA2G4F
15	42425001	42426000	ZNF106;RNU6-188P
15	42473001	42474000	ZNF106
15	43220001	43221000	EPB42
15	44205001	44206000	FRMD5
15	44438001	44439000	CTDSPL2;HNRNPMP1
15	45348001	45349000	RNU6-953P
15	45722001	45723000	LOC105370802
15	45792001	45793000	LOC105370802
15	46642001	46643000	LOC105370803
15	47767001	47768000	SEMA6D
15	49264001	49265000	GALK2
15	50267001	50268000	HDC;GABPB1
15	52247001	52248000	MYO5C
15	52344001	52345000	MYO5A
15	52834001	52835000	LOC107983981
15	53006001	53007000	LOC107983981
15	53431001	53432000	LOC105370826
15	56139001	56140000	RFX7
15	56673001	56674000	ZNF280D
15	57247001	57248000	TCF12;HNRNPA3P11
15	57409001	57410000	CGNL1;RNU6-844P
15	57499001	57500000	CGNL1;LOC107984759
15	57753001	57755000	LOC105370834;LOC105370834
15	58154001	58155000	AQP9;MTCO3P23;MTND3P12;MTND4LP23;MTND5P32;MTND6P23;MTCYBP23
15	58984001	58985000	RNF111
15	59645001	59646000	GTF2A2
15	60355001	60356000	ANXA2
15	60418001	60419000	ICE2
15	61077001	61078000	RORA;LOC107984805
15	61418001	61419000	LOC105370847
15	62351001	62352000	MIR6085;HMGN1P26
15	62948001	62949000	LOC100287243
15	63417001	63418000	LINC02568
15	64442001	64443000	TRIP4;LOC105370861
15	64499001	64500000	ZNF609
15	64836001	64837000	PIF1;PLEKHO2
15	64840001	64841000	PLEKHO2
15	64862001	64863000	PLEKHO2

15	65104001	65105000	UBAP1L
15	66832001	66833000	LOC105370869
15	68421001	68423000	ITGA11;ITGA11
15	69181001	69183000	GLCE;GLCE
15	69410001	69411000	PAQR5;KIF23-AS1;KIF23
15	69440001	69441000	KIF23;LOC100421811
15	69598001	69599000	PCAT29
15	70629001	70630000	SALRNA2
15	70827001	70828000	LARP6
15	71203001	71204000	THSD4
15	72319001	72320000	CELF6
15	73351001	73352000	HCN4;LOC105370890
15	73709001	73710000	CD276
15	73745001	73746000	INSYN1
15	73759001	73760000	INSYN1;INSYN1-AS1
15	73767001	73768000	INSYN1-AS1
15	73820001	73821000	LOC105370892
15	73896001	73897000	TBC1D21
15	74208001	74209000	STRA6;CCDC33
15	74299001	74300000	CCDC33
15	74354001	74355000	CYP11A1;PPIAP46
15	74414001	74415000	SEMA7A;MIR6881
15	74443001	74444000	SEMA7A;UBL7
15	74638001	74639000	CLK3;EDC3
15	74743001	74744000	CYP1A2
15	75027001	75028000	SCAMP5;PPCDC
15	75265001	75266000	GOLGA6C;RN7SL489P
15	75289001	75290000	GOLGA6D;RN7SL327P;DNM1P34
15	75474001	75475000	PTPN9;LOC105370902
15	75612001	75613000	SNUPN
15	75679001	75680000	CSPG4
15	75701001	75702000	CSPG4
15	75703001	75704000	CSPG4
15	75924001	75925000	FBXO22;NRG4
15	76153001	76154000	TMEM266
15	76280001	76281000	ETFA
15	76724001	76725000	SCAPER
15	77045001	77046000	PSTPIP1;TSPAN3
15	77463001	77464000	HMG20A
15	77674001	77675000	LINGO1;LINGO1-AS2
15	77721001	77722000	LINGO1
15	77782001	77783000	LINGO1
15	77871001	77872000	CSPG4P13
15	77888001	77889000	CSPG4P13
15	78334001	78335000	CRABP1
15	78385001	78386000	RWDD1P1
15	78455001	78456000	IREB2
15	78682001	78683000	LOC112268142;LOC105370913
15	78770001	78771000	ADAMTS7
15	79024001	79025000	RASGRF1
15	79031001	79032000	RASGRF1
15	79384001	79385000	TMED3
15	79438001	79439000	MINAR1
15	79918001	79919000	ST20-MTHFS;ST20;ST20-AS1
15	80055001	80056000	ZFAND6
15	80108001	80109000	ZFAND6
15	80443001	80444000	ARNT2
15	80461001	80462000	ARNT2
15	80506001	80507000	ARNT2
15	80509001	80510000	ARNT2
15	80735001	80736000	ABHD17C
15	81282001	81283000	IL16
15	81951001	81952000	LOC102724001
15	82007001	82008000	LOC105370922;LOC100288241
15	82434001	82435000	GOLGA6L9;DNM1P36;LOC102724034
15	82524001	82525000	GOLGA6L17P;DNM1P38
15	82703001	82704000	AP3B2;LOC338963
15	82705001	82706000	AP3B2;LOC338963
15	82872001	82873000	HOMER2
15	82934001	82935000	HOMER2;LOC105370928
15	83109001	83110000	TM6SF1;HDGFL3
15	83116001	83117000	TM6SF1;HDGFL3
15	83695001	83696000	ADAMTSL3
15	84008001	84009000	ADAMTSL3
15	84114001	84115000	EFL1P1
15	84240001	84241000	GOLGA2P7;GOLGA6L4;UBE2Q2P8
15	85102001	85103000	PDE8A
15	85435001	85436000	AKAP13
15	85446001	85447000	AKAP13
15	85568001	85569000	AKAP13
15	86529001	86530000	AGBL1
15	86587001	86588000	AGBL1
15	87459001	87460000	LOC102724465
15	87465001	87466000	LOC102724465;RNU6-185P
15	88028001	88029000	NTRK3
15	88125001	88126000	NTRK3
15	88547001	88551000	DET1;DET1;DET1;DET1

15	89048001	89049000	CARMAL
15	89736001	89737000	WDR93;MESP1
15	89746001	89747000	WDR93;MESP1;MRPL15P1
15	89754001	89755000	WDR93;MESP1;MRPL15P1
15	89905001	89906000	ARPIN-AP3S2;ARPIN;RNU7-111P
15	90054001	90055000	ZNF710
15	90099001	90100000	IDH2;IDH2-DT
15	90136001	90138000	IDH2-DT;IDH2-DT
15	90179001	90180000	SEMA4B
15	90210001	90211000	SEMA4B;RPS12P26
15	90534001	90535000	CRTC3
15	90646001	90647000	CRTC3;CRTC3-AS1
15	91018001	91019000	VPS33B;LOC105370970;VPS33B-DT
15	91039001	91040000	VPS33B-DT;LOC390638
15	91981001	91982000	SLCO3A1;LOC107984747
15	92018001	92019000	SLCO3A1
15	92032001	92033000	SLCO3A1
15	92145001	92146000	SLCO3A1
15	92296001	92297000	NPM1P5
15	92426001	92427000	ST8SIA2;ENO1P2
15	92467001	92468000	ST8SIA2;LOC104613533;C15orf32
15	92652001	92653000	FAM174B;LOC105370975
15	93312001	93313000	LOC105370982
15	93468001	93469000	LOC105370982
15	93478001	93479000	LOC105370982
15	93773001	93774000	LOC107983974
15	94389001	94390000	MCTP2
15	94581001	94582000	LINC02852
15	94754001	94757000	LOC105370988;LOC105370988;LOC105370988
15	95088001	95089000	LOC105370991
15	95881001	95882000	LOC107984800
15	96373001	96374000	LOC101927263
15	97859001	97860000	LINC00923
15	98412001	98413000	LINC02351
15	98503001	98504000	FAM169B
15	98505001	98506000	FAM169B
15	98824001	98825000	IGF1R
15	98946001	98947000	IGF1R
15	99120001	99121000	SYNM
15	100021001	100022000	ADAMTS17
15	100100001	100101000	ADAMTS17;LOC107984740
15	100139001	100140000	ADAMTS17
15	100257001	100258000	ADAMTS17
15	100297001	100298000	ADAMTS17
15	100320001	100321000	ADAMTS17
15	100441001	100442000	CERS3-AS1;CERS3
15	100443001	100444000	CERS3-AS1;CERS3
15	100445001	100446000	CERS3-AS1;CERS3
15	100706001	100707000	LOC105371024
15	100718001	100719000	LOC105371024
15	100720001	100721000	LOC105371024
15	100767001	100768000	LOC105371024
15	101073001	101074000	LRRK1;LOC105371026
15	101122001	101123000	LOC105371026
15	101154001	101155000	LOC105371026
15	101893001	101895000	WBP1LP5;WBP1LP5
16	101001	102000	NPRL3
16	503001	504000	RAB11FIP3
16	584001	585000	PIGQ;RAB40C;LOC101929280
16	591001	592000	PIGQ;RAB40C;LOC101929280
16	632001	633000	RAB40C;WFIKKN1;METTL26;TRG-CCC2-2;LOC100287175;MCRIP2
16	778001	779000	MSLN;MSLNL;MIR662;RPUSD1;CHTF18
16	936001	937000	LMF1;LMF1-AS1
16	1102001	1103000	C1QTNF8;LOC107984906
16	1113001	1115000	LOC107984906;LOC107984906
16	1231001	1232000	CACNA1H;TPSG1;TPSB2;TPSAB1
16	1275001	1276000	TPSP1
16	1568001	1570000	IFT140;IFT140
16	1581001	1582000	IFT140;LOC105371046
16	1621001	1622000	IFT140;CRAMP1
16	1950001	1951000	MSRB1;RPL3L;NDUFB10
16	2230001	2231000	E4F1;DNASE1L2;ECI1
16	2483001	2484000	NTN3;TBC1D24
16	2512001	2513000	TBC1D24;ATP6V0C;AMDHD2
16	2761001	2762000	SRRM2-AS1;SRRM2;ELOB
16	2835001	2836000	ZG16B;PRSS30P
16	2856001	2857000	PRSS22;LOC101929566
16	3349001	3350000	OR2C1
16	3368001	3369000	LOC105371059;MTCO2P28;MTCO1P28;MTND2P34;MTND1P8;MTRNR2L4
16	4049001	4050000	ADCY9
16	4360001	4361000	CORO7-PAM16;PAM16;CORO7
16	4595001	4596000	C16orf96
16	5459001	5460000	RBFOX1
16	6290001	6291000	RBFOX1
16	6441001	6442000	RBFOX1
16	7488001	7489000	RBFOX1
16	8629001	8630000	METTL22

16	8640001	8641000	METTL22
16	8711001	8712000	ABAT;RNU7-63P
16	8725001	8726000	ABAT
16	8761001	8762000	ABAT
16	8834001	8835000	PMM2
16	8837001	8838000	PMM2
16	8843001	8845000	PMM2;LOC100130283;CARHSP1;PMM2;LOC100130283;CARHSP1
16	9114001	9115000	C16orf72
16	9435001	9436000	LOC101927026;LINC01177
16	9481001	9482000	LOC101927026
16	9576001	9577000	LOC101927026
16	9883001	9884000	GRIN2A
16	10037001	10038000	GRIN2A;LOC105371076
16	10480001	10481000	ATF7IP2
16	10720001	10721000	MTCYBP33;MTND6P33;MTND5P33;MTND4P34;MTND4LP24;MTND3P13;MTCO3P24;MTATP6P24
16	10729001	10730000	MTCYBP33;MTND6P33;MTND5P33;MTND4P34;MTND4LP24;MTND3P13;MTCO3P24;MTATP6P24
16	10867001	10868000	LOC105371080;CIITA
16	10869001	10870000	LOC105371080;CIITA
16	11270001	11271000	LOC105371082;TNP2;PRM3;PRM2;PRM1
16	11485001	11486000	LOC400499
16	11487001	11488000	LOC400499
16	11523001	11524000	LOC400499
16	11664001	11665000	SNN
16	11696001	11697000	TXNDC11
16	12163001	12164000	SNX29
16	12203001	12204000	SNX29
16	12272001	12273000	SNX29
16	13355001	13356000	SHISA9
16	14477001	14478000	PARN;LOC105371094
16	14751001	14752000	LOC105376747;NPIPA2;LOC105376749
16	14873001	14874000	NOMO1
16	14904001	14905000	NOMO1;LOC101927469;MIR3179-1;MIR3670-1;MIR3180-1;PKD1P3-NPIPA1
16	14961001	14962000	PKD1P3-NPIPA1;NPIPA1
16	15131001	15132000	PDXDC1;NPIPP1;PKD1P6-NPIPP1;PKD1P6;MIR6511B2
16	15447001	15448000	BMERB1
16	15496001	15497000	BMERB1;LOC105371102
16	15601001	15602000	MARF1;MIR6506
16	15767001	15768000	MYH11
16	15898001	15899000	CEP20;LOC107984869
16	16092001	16093000	ABCC1;RPL17P40
16	16327001	16328000	PKD1P1;MIR6511A2;MIR6770-2
16	16379001	16380000	PKD1P2;NPIPA7;LOC105371098
16	17480001	17481000	XYLT1;LOC107987234;LOC105371106
16	18332001	18333000	LOC105376753;PKD1P4-NPIPA8;NPIPA8;PKD1P4
16	18341001	18342000	LOC105376753;PKD1P4-NPIPA8;NPIPA8;PKD1P4;MIR6511A4
16	18372001	18373000	NPIPA9;PKD1P5-LOC105376752;PKD1P5;MIR6770-3
16	18535001	18536000	NOMO2
16	18990001	18991000	TMC7
16	19224001	19225000	SYT17
16	20403001	20404000	PDILT;ACSM5
16	20610001	20611000	ACSM5P1;LOC107984831
16	21439001	21440000	LOC100190986;SMG1P3
16	21507001	21508000	SMG1P3;MIR3680-1;SLC7A5P2
16	21987001	21988000	UQCRC2;PDZD9;LOC100420644
16	22495001	22496000	SMG1P1
16	22499001	22500000	SMG1P1;NPIPB5
16	22900001	22901000	HS3ST2
16	23563001	23564000	EARS2;UBFD1
16	23757001	23758000	CHP2
16	23927001	23928000	PRKCB
16	24124001	24125000	PRKCB
16	24126001	24127000	PRKCB
16	24292001	24293000	CACNG3
16	24957001	24958000	ARHGAP17
16	25148001	25149000	LCMT1;LCMT1-AS2
16	25913001	25914000	HS3ST4
16	25979001	25980000	HS3ST4
16	26373001	26374000	LOC102723536
16	27168001	27169000	LINC02129
16	27228001	27229000	KDM8;NSMCE1
16	27259001	27260000	NSMCE1;NSMCE1-DT
16	27305001	27306000	IL4R
16	27472001	27473000	GTF3C1
16	27530001	27531000	GTF3C1
16	27546001	27547000	GTF3C1;KATNIP
16	27637001	27638000	KATNIP
16	27785001	27786000	KATNIP;GSG1L
16	27927001	27928000	GSG1L
16	28051001	28052000	GSG1L
16	28053001	28054000	GSG1L
16	28495001	28496000	CLN3;APOBR;IL27
16	28501001	28502000	CLN3;APOBR;IL27
16	28860001	28861000	SH2B1
16	28870001	28871000	SH2B1;ATP2A1;ATP2A1-AS1
16	28883001	28885000	SH2B1;ATP2A1;ATP2A1-AS1;SH2B1;ATP2A1;ATP2A1-AS1
16	29297001	29298000	LOC105371159;LOC107984833;SNX29P2
16	29418001	29419000	SMG1P6

16	29814001	29815000	KIF22;MAZ;LOC112268170;PRRT2;PAGR1;MVP
16	29897001	29898000	SEZ6L2;ASPHD1;KCTD13
16	30102001	30103000	YPEL3;YPEL3-DT;GDPD3
16	30478001	30479000	ITGAL;RNU7-61P
16	30544001	30545000	ZNF747;LOC107984875;LOC100127965;ZNF764
16	30707001	30708000	LOC730183;SRCAP;RNU6-1043P;SNORA30
16	30720001	30721000	SRCAP;SNORA30
16	30737001	30738000	SRCAP;TMEM265
16	30862001	30863000	BCL7C
16	30960001	30961000	ORAI3;SETD1A
16	31015001	31016000	STX1B
16	31253001	31254000	ITGAM
16	31310001	31311000	ITGAM
16	32867001	32868000	BCAP31P2;SLC6A10P
16	32876001	32877000	BCAP31P2;SLC6A10P
16	33590001	33591000	LOC102724181
16	33935001	33936000	ARHGAP23P1;IGHV3OR16-7
16	34134001	34135000	DUX4L45;PCMTD1P2;DUX4L46;DUX4L47
16	46813001	46814000	C16orf87
16	46919001	46920000	GPT2
16	47400001	47401000	ITFG1
16	47407001	47408000	ITFG1
16	47944001	47945000	LOC105371237
16	48635001	48636000	LOC105371240
16	49109001	49110000	LOC105371241
16	49462001	49463000	LOC105371244;LOC105371245;LINC02179
16	49760001	49761000	ZNF423;MRPS21P8
16	50043001	50044000	CNEP1R1;LOC100130602
16	50110001	50111000	HEATR3
16	50374001	50375000	BRD7
16	50571001	50572000	NKD1
16	50695001	50696000	NOD2
16	51014001	51015000	LINC02127
16	52449001	52450000	TOX3
16	53815001	53816000	FTO
16	53911001	53912000	FTO
16	54290001	54291000	IRX3
16	54910001	54911000	CRNDE
16	55443001	55444000	MMP2-AS1
16	56269001	56270000	GNAO1
16	56331001	56332000	GNAO1
16	56347001	56348000	GNAO1
16	56400001	56401000	AMFR
16	56674001	56675000	LOC101927536;MT1G;MT1H;MT1IP;MT1X
16	56733001	56734000	NUP93-DT;LOC105371287;NUP93
16	56951001	56952000	HERPUD1;CETP
16	56975001	56976000	CETP
16	57053001	57054000	NLRC5
16	57129001	57130000	CPNE2;LOC105371289
16	57270001	57271000	PLLP
16	57358001	57359000	CCL22
16	57388001	57389000	CX3CL1;CCL17
16	57515001	57517000	CCDC102A;CCDC102A
16	57625001	57626000	ADGRG1
16	57651001	57652000	ADGRG1
16	57728001	57729000	DRC7;LOC107984852;KATNB1
16	57845001	57846000	KIFC3
16	58016001	58017000	USB1;MMP15
16	58119001	58120000	CFAP20;LOC101927556
16	59893001	59894000	LINC02141
16	61428001	61429000	LOC105371302
16	64989001	64990000	CDH11
16	65275001	65277000	LINC00922;LINC00922
16	65295001	65296000	LINC00922
16	67283001	67285000	PLEKHG4;KCTD19;PLEKHG4;KCTD19
16	67450001	67451000	ATP6V0D1
16	67454001	67455000	ATP6V0D1
16	67522001	67523000	LOC100505942;RIPOR1
16	67729001	67730000	GFOD2;RANBP10
16	68693001	68694000	CDH3;HSPE1P5
16	68870001	68871000	TANGO6
16	69066001	69067000	TANGO6
16	69925001	69926000	WWP2;MIR140
16	70427001	70428000	ST3GAL2
16	70620001	70621000	IL34
16	70622001	70623000	IL34
16	70830001	70831000	HYDIN
16	70978001	70979000	HYDIN
16	71165001	71166000	HYDIN
16	71376001	71377000	CALB2;LOC105371332
16	71382001	71383000	CALB2;LOC105371332
16	71777001	71778000	AP1G1;LOC105371337
16	71827001	71828000	LOC100420489
16	71850001	71851000	ATXN1L;ZNF821
16	72137001	72138000	PMFBP1
16	72157001	72158000	PMFBP1
16	72610001	72611000	LINC01572

16	72907001	72908000	ZFHX3
16	72949001	72950000	ZFHX3
16	72973001	72974000	ZFHX3
16	74213001	74214000	PSMD7-DT;PPIAP49
16	74377001	74378000	PDPR2P;NPIPB15
16	74470001	74471000	GLG1
16	74883001	74884000	WDR59
16	75428001	75429000	CFDP1
16	76348001	76349000	CNTNAP4
16	76471001	76472000	CNTNAP4
16	77193001	77194000	MON1B;SYCE1L
16	77478001	77479000	LOC105376775
16	77498001	77499000	LOC105376775
16	78181001	78182000	WWOX
16	78412001	78413000	WWOX
16	78989001	78990000	WWOX
16	79824001	79825000	LINC01228
16	80188001	80189000	DYNLRB2-AS1
16	81040001	81041000	CENPN;CENPN-AS1;ATMIN
16	81110001	81111000	PKD1L2;RNU6-1191P
16	81216001	81217000	PKD1L2
16	81646001	81647000	CMIP
16	81704001	81705000	CMIP
16	81817001	81818000	PLCG2
16	81878001	81879000	PLCG2
16	81934001	81935000	PLCG2
16	82070001	82071000	HSD17B2;LOC105371363
16	82113001	82114000	MTCYBP28;MTND6P28
16	82672001	82673000	CDH13
16	82895001	82896000	CDH13
16	83394001	83395000	CDH13;LOC105371368
16	83590001	83591000	CDH13
16	83751001	83752000	CDH13
16	83925001	83926000	MLYCD;LOC105371372;LOC105371371
16	83967001	83968000	OSGIN1;NECAB2
16	83975001	83976000	OSGIN1;NECAB2
16	84106001	84107000	MBTPS1
16	84228001	84229000	KCNG4
16	84257001	84258000	RNA5SP433
16	84443001	84444000	ATP2C2
16	84603001	84605000	COTL1;COTL1
16	84614001	84615000	COTL1
16	84930001	84931000	LOC101928474;LOC123862;LINC02176
16	85004001	85005000	ZDHHC7
16	85139001	85141000	LINC02139;LOC105371382;LINC02139;LOC105371382
16	85151001	85152000	LINC02139;LOC105371382
16	85248001	85249000	GSE1
16	85347001	85349000	GSE1;GSE1
16	85405001	85406000	GSE1
16	85519001	85520000	GSE1
16	85522001	85523000	GSE1
16	85683001	85684000	GSE1;GINS2
16	85726001	85727000	C16orf74
16	85759001	85760000	C16orf74
16	85782001	85783000	EMC8;RNU1-103P;LOC101928557
16	86224001	86225000	LINC01081
16	86263001	86264000	LINC01081
16	86279001	86280000	LINC01081;LINC02135
16	86930001	86931000	LOC105371393
16	87213001	87214000	LOC101928708
16	87222001	87223000	LOC101928708
16	87700001	87701000	JPH3;KLHDC4;LOC100129215
16	87881001	87882000	CA5A
16	88273001	88274000	LOC107984862
16	88492001	88493000	ZFPM1
16	88555001	88556000	LOC107984905;ZC3H18-AS1
16	88607001	88608000	ZC3H18
16	88614001	88615000	ZC3H18
16	88704001	88705000	RNF166;CTU2
16	88713001	88714000	RNF166;CTU2;PIEZO1;MIR4722
16	88741001	88742000	PIEZO1;LOC100289580;LOC339059
16	88955001	88956000	CBFA2T3;LOC100129697
16	89062001	89063000	LOC105371411;LOC105371412
16	89091001	89092000	ACSF3
16	89112001	89113000	ACSF3
16	89251001	89252000	LOC105371413
16	89447001	89448000	ANKRD11;LOC101927817;RNU6-430P
16	90121001	90122000	FAM157C;LOC105376781
16	90166001	90167000	FAM157C;LOC105376781;LOC101927999;CICP25
16	90218001	90219000	LINC02193;LOC107987240
17	148001	149000	SCGB1C2;DOC2B
17	158001	159000	DOC2B
17	238001	239000	RPH3AL
17	504001	505000	VPS53
17	642001	643000	VPS53
17	649001	650000	VPS53;RPS4XP17
17	671001	672000	VPS53

17	856001	857000	NXN
17	892001	893000	NXN
17	1053001	1054000	ABR
17	1074001	1075000	ABR
17	1146001	1147000	ABR;LOC105371480;MRPL14P1
17	1172001	1173000	ABR;LOC105371479
17	1285001	1286000	TRARG1
17	1473001	1474000	MYO1C
17	1506001	1507000	INPP5K;PITPNA-AS1
17	1566001	1567000	PITPNA;SLC43A2
17	1705001	1706000	TLCD2;MIR22HG;MIR22
17	1738001	1739000	WDR81;SERPINF2
17	1742001	1743000	WDR81;SERPINF2
17	1967001	1968000	RTN4RL1
17	2060001	2061000	MIR132;MIR212;HIC1;SMG6;LOC107984988
17	2186001	2187000	SMG6
17	2294001	2295000	SMG6;SRR
17	2411001	2412000	MNT;LOC284009;METTL16
17	2779001	2780000	RAP1GAP2
17	3422001	3423000	OR3A3;OR1E2
17	3520001	3521000	SPATA22;TRPV3
17	3527001	3528000	TRPV3
17	3543001	3544000	TRPV3
17	3553001	3554000	TRPV3
17	3601001	3602000	TRPV1;SHPK
17	3621001	3622000	SHPK;LOC100422717
17	3648001	3649000	CTNS;LOC105371493;LOC105371492
17	3656001	3657000	CTNS;LOC105371493;LOC105371492;P2RX5-TAX1BP3;TAX1BP3;RPL21P125
17	3748001	3750000	ITGAE;ITGAE
17	3751001	3752000	ITGAE
17	3763001	3764000	ITGAE
17	3925001	3926000	P2RX1;ATP2A3
17	3929001	3930000	ATP2A3
17	3938001	3939000	ATP2A3
17	4158001	4159000	CYB5D2;ANKFY1
17	4169001	4170000	ANKFY1
17	4244001	4245000	ANKFY1
17	4295001	4296000	UBE2G1;MFSD1P1
17	4590001	4591000	SMTNL2
17	4684001	4685000	PELP1
17	4706001	4707000	PELP1;LOC101559451;RPS12P29;ARRB2
17	4811001	4812000	PLD2
17	4873001	4874000	MINK1;RN7SL784P
17	4951001	4952000	RNF167;PFN1;ENO3;SPAG7
17	5100001	5101000	ZFP3;ZNF232
17	5122001	5123000	ZNF232;ZNF232-AS1;USP6
17	5126001	5127000	ZNF232;USP6
17	5497001	5498000	MIS12;LOC728392;NLRP1
17	5543001	5544000	NLRP1;LOC105371507
17	5820001	5821000	LOC339166
17	5929001	5930000	LOC339166
17	6171001	6172000	LOC105371508
17	6458001	6459000	LOC107985017;PIMREG;PITPNM3
17	6636001	6637000	KIAA0753;TXNDC17;MED31
17	6668001	6669000	ALOX15P1
17	6714001	6715000	SLC13A5
17	6999001	7000000	ALOX12-AS1;ALOX12
17	7071001	7072000	CLEC10A
17	7123001	7124000	ASGR2;LOC105371512
17	7208001	7209000	DLG4;ACADVL
17	7430001	7431000	SPEM1;SPEM2;SPEM3;TMEM102;FGF11
17	7636001	7637000	SHBG;SAT2;ATP1B2
17	7654001	7655000	ATP1B2
17	7696001	7697000	TP53;WRAP53;EFNB3
17	7709001	7710000	WRAP53;EFNB3;DNAH2
17	7754001	7755000	DNAH2;RPL29P2
17	7794001	7795000	DNAH2
17	8044001	8045000	ALOX15B
17	8114001	8115000	ALOXE3;TRK-TTT3-5;TRQ-CTG1-5;TRL-TAG1-1;HES7;TRR-TCT2-1
17	8312001	8313000	ARHGEF15
17	8622001	8623000	MYH10
17	8755001	8756000	LOC105371529;SPDYE4;LOC105371530
17	8818001	8819000	PIK3R6
17	8841001	8842000	PIK3R6
17	8881001	8883000	LOC107985063;PIK3R5;LOC107985063;PIK3R5
17	9015001	9016000	NTN1
17	9139001	9140000	NTN1
17	9156001	9157000	NTN1
17	9278001	9279000	STX8
17	9281001	9282000	STX8
17	9414001	9415000	STX8
17	9678001	9679000	USP43
17	9712001	9713000	USP43
17	9840001	9841000	GLP2R
17	9945001	9946000	GAS7
17	9975001	9976000	GAS7
17	10169001	10170000	GAS7

17	10576001	10577000	MYHAS
17	10808001	10809000	TMEM220-AS1;TMEM238L
17	11271001	11272000	SHISA6
17	11385001	11386000	SHISA6
17	11533001	11534000	SHISA6
17	11623001	11624000	DNAH9
17	11782001	11783000	DNAH9
17	11800001	11801000	DNAH9
17	12068001	12069000	MAP2K4
17	12698001	12699000	MYOCD;MYOCD-AS1
17	12853001	12854000	ARHGAP44
17	12876001	12877000	ARHGAP44
17	12901001	12902000	ARHGAP44
17	12989001	12990000	ARHGAP44;ELAC2
17	13871001	13872000	LOC100506974
17	14395001	14396000	LOC107985080
17	14419001	14420000	LOC107985080
17	15098001	15099000	CDRT8
17	15250001	15251000	PMP22;MIR4731
17	15464001	15465000	TVP23C-CDRT4;CDRT4;CDRT3
17	15739001	15740000	ZNF286A-TBC1D26;TBC1D26;TBC1D26-AS1;LOC105371559
17	15804001	15806000	LINC02087;LINC02087
17	16074001	16075000	NCOR1;RPL22P21
17	16143001	16144000	NCOR1;RNU6-862P;SNORD163
17	16267001	16268000	PIGL
17	16918001	16919000	TBC1D27P
17	16990001	16991000	LOC284191;LINC02090
17	17276001	17277000	COPS3
17	17336001	17337000	NT5M
17	17503001	17504000	MED9;RASD1;PEMT
17	18489001	18490000	LGALS9C;NOS2P2
17	18877001	18878000	PRPSAP2
17	18964001	18965000	SLC5A10;FAM83G
17	19537001	19538000	SLC47A1
17	20000001	20001000	SPECC1
17	20454001	20455000	NOS2P3;LGALS9B
17	20567001	20568000	COTL1P2;LOC105371580
17	20607001	20608000	LOC401875;LINC02088
17	21074001	21075000	LINC01563
17	21426001	21427000	KCNJ12
17	21689001	21690000	KCNJ18
17	21711001	21712000	KCNJ18
17	22519001	22521000	MTND6P35;MTCYBP13;MTRNR2L1;MTND1P15;MTND2P13;MTCO1P13;NMTRS-TGA3-1;MTCO2P13;MTATP6P3;MTND6P35;MTCYBP13;MTRNR2L1;MTND1P15;MTND2P13;MTCO1P13;NMTRS-TGA3-1;MTCO2P13;MTATP6P3;MTCO3P13
17	28214001	28215000	NLK;LOC105371710
17	28382001	28383000	SARM1
17	28472001	28473000	RPS7P1;SLC13A2
17	28769001	28770000	FAM222B
17	29037001	29038000	PIPOX
17	29081001	29082000	MYO18A;TIAF1
17	29109001	29110000	MYO18A
17	30414001	30415000	CPD
17	30703001	30704000	LOC105371723;RN7SL316P;SUZ12P1
17	30719001	30720000	SUZ12P1
17	31430001	31431000	RAB11FIP4;LOC105371725
17	31473001	31474000	RAB11FIP4;RN7SL79P
17	31846001	31847000	COPRS
17	32022001	32023000	LRRC37B
17	32761001	32762000	MYO1D
17	32773001	32774000	MYO1D
17	32991001	32992000	SPACA3
17	32993001	32994000	SPACA3
17	33133001	33134000	ASIC2;LOC105371737
17	33249001	33250000	ASIC2
17	34011001	34012000	ASIC2;LOC107987247
17	34184001	34185000	LINC01989
17	35032001	35033000	RFFL;RAD51L3-RFFL
17	35062001	35063000	RFFL;RAD51L3-RFFL
17	35193001	35194000	UNC45B;LOC100130114;SLC35G3
17	36094001	36095000	CCL3-AS1;CCL3;LOC101927369;CCL4
17	36604001	36605000	DHRS11;LOC107985031;MRM1
17	37025001	37026000	AATF;MIR2909
17	37205001	37206000	ACACA
17	37521001	37522000	DUSP14;SYNRG
17	37537001	37538000	SYNRG
17	37650001	37651000	DDX52;LOC105371755;LOC105371756
17	38425001	38426000	ARHGAP23
17	38485001	38486000	ARHGAP23
17	38696001	38697000	LOC105371764;LOC105371763;MIR4734;MLLT6
17	39004001	39005000	LOC105371767
17	39032001	39033000	LOC105371767;LINC02079;LRRC37A11P
17	39186001	39187000	LOC105371768;CACNB1
17	39709001	39710000	ERBB2
17	40016001	40017000	CSF3;MED24;MIR6884
17	40041001	40042000	MED24
17	40266001	40268000	WIPF2;WIPF2

17	41205001	41206000	KRTAP9-12P
17	41308001	41309000	KRTAP29-1;KRTAP16-1;KRTAP17-1
17	41705001	41706000	GAST
17	41863001	41864000	KLHL11;ACLY
17	42165001	42166000	RAB5C-AS1;KCNH4
17	42171001	42172000	KCNH4
17	42175001	42176000	KCNH4;HCRT
17	42367001	42368000	STAT3
17	42394001	42395000	STAT3;CAVIN1
17	42772001	42773000	RAMP2;VPS25;WNK4
17	43243001	43244000	RNU2-1
17	43415001	43416000	RNU6-470P
17	43912001	43913000	MPP2;LOC105371787;FAM215A;LOC107985086;LRRC37A10P
17	44258001	44259000	SLC4A1
17	44312001	44313000	RUNDC3A-AS1;RNU6-453P;RUNDC3A;SLC25A39
17	44480001	44481000	GPATCH8
17	44751001	44752000	DBF4B;ADAM11
17	44787001	44788000	ADAM11;GJC1
17	44889001	44890000	EFTUD2;RN7SL405P;CCDC103
17	44913001	44914000	CCDC103;GFAP;FAM187A;LOC105371793
17	45389001	45390000	ARHGAP27
17	45426001	45427000	ARHGAP27;PLEKHM1
17	45547001	45548000	LOC105369225;RDM1P1
17	45658001	45659000	LINC02210-CRHR1;LOC105371802
17	45796001	45797000	LINC02210-CRHR1;CRHR1
17	45848001	45849000	MAPT-AS1;SPPL2C
17	45964001	45965000	MAPT
17	46241001	46242000	MAPK8IP1P1
17	46287001	46288000	ARL17B;LRRC37A
17	46572001	46573000	ARL17A;LRRC37A2
17	46778001	46779000	LRRC37A2;WNT3
17	46927001	46928000	LRRC37A2;RNU6ATAC3P;GOSR2
17	47811001	47812000	OSBPL7
17	47866001	47867000	SP6
17	47922001	47923000	SP2;SP2-AS1
17	47936001	47937000	SP2;SP2-AS1;PNPO
17	47979001	47980000	CDK5RAP3
17	48801001	48802000	TTLL6
17	49265001	49266000	FLJ40194
17	49465001	49466000	LINC02075
17	49562001	49563000	NGFR-AS1
17	49591001	49592000	NXPH3;SPOP
17	49725001	49726000	FAM117A
17	49807001	49808000	KAT7
17	49852001	49853000	TAC4;FLJ45513
17	49855001	49856000	TAC4;FLJ45513
17	50049001	50050000	PICART1;ITGA3
17	50283001	50284000	TMEM92;TMEM92-AS1
17	50351001	50352000	XYLT2
17	50385001	50386000	EME1;LRRC59
17	50650001	50651000	ABCC3
17	52485001	52486000	LINC01982
17	55518001	55519000	LOC105371833
17	56105001	56106000	ANKFN1
17	56892001	56893000	MTVR2;TRIM25;MIR3614
17	56984001	56985000	LOC105371836;SCPEP1
17	57081001	57082000	AKAP1
17	57106001	57107000	AKAP1
17	57446001	57447000	MSI2
17	57627001	57628000	MSI2;RN7SL449P
17	57784001	57785000	RN7SKP94
17	58187001	58188000	EPX
17	58321001	58322000	TSPOAP1;TSPOAP1-AS1;MIR142
17	58375001	58376000	RNF43
17	59177001	59178000	PRR11;SPDYE22P
17	59179001	59180000	PRR11;SPDYE22P
17	59396001	59397000	YPEL2
17	59449001	59450000	LINC01476
17	59494001	59495000	LINC01476;LOC105371845
17	60022001	60023000	TBC1D3P1-DHX40P1;TBC1D3P1;LOC105371848
17	60385001	60386000	USP32
17	60598001	60599000	PPM1D
17	61341001	61342000	BCAS3
17	61345001	61346000	BCAS3
17	62734001	62735000	MARCHF10;LOC105371855
17	62798001	62799000	MARCHF10;MARCHF10-DT
17	62819001	62820000	MARCHF10-DT
17	62945001	62946000	MIR633;TRMT112P3
17	63440001	63441000	CYB561
17	63539001	63540000	KCNH6
17	64237001	64238000	TEX2
17	64611001	64612000	SMURF2
17	64894001	64895000	LOC105376844;LRRC37A3
17	65158001	65159000	RGS9
17	65183001	65184000	RGS9;ZNF848P
17	65529001	65530000	AXIN2
17	65568001	65569000	AXIN2

17	65682001	65683000	CEP112
17	65715001	65716000	CEP112
17	65720001	65721000	CEP112
17	65848001	65849000	CEP112
17	65884001	65885000	CEP112;LOC105371867
17	66065001	66066000	CEP112
17	66186001	66187000	CEP112
17	66329001	66330000	PRKCA
17	66425001	66426000	PRKCA;PRKCA-AS1
17	66436001	66438000	PRKCA;PRKCA
17	66776001	66777000	PRKCA
17	66979001	66980000	CACNG4
17	67026001	67027000	CACNG4
17	67152001	67153000	HELZ
17	67216001	67217000	HELZ;RPL36AP48
17	68683001	68684000	LINC01482
17	68994001	68995000	ABCA9;ABCA9-AS1
17	69003001	69004000	ABCA9;ABCA9-AS1
17	69456001	69457000	MAP2K6
17	69799001	69800000	LINC01483
17	72132001	72133000	SOX9
17	72886001	72887000	SLC39A11
17	72915001	72916000	SLC39A11
17	72978001	72979000	SLC39A11
17	73026001	73027000	SLC39A11
17	73068001	73069000	SLC39A11;ATG12P1
17	73087001	73088000	SLC39A11
17	73216001	73217000	COG1;FAM104A
17	73374001	73375000	SDK2
17	73445001	73446000	SDK2
17	73719001	73720000	LOC107985091
17	73732001	73733000	LOC107985091;LOC100134391
17	73947001	73948000	LOC105371888
17	74098001	74100000	LINC02074;LINC02074
17	74302001	74303000	DNAI2;LOC105371891
17	74345001	74346000	KIF19;LOC107985078
17	74514001	74515000	CD300LB
17	74561001	74562000	CD300H
17	74618001	74619000	LOC101928343;CD300E
17	74786001	74787000	NAT9;TMEM104
17	74865001	74866000	GRIN2C;FDXR
17	75082001	75083000	TRIM80P;SLC16A5
17	75255001	75256000	GGA3;MRPS7
17	75486001	75488000	TMEM94;TMEM94
17	75622001	75623000	MYO15B;RECQL5
17	76130001	76131000	LOC101928447;FOXJ1;RNF157-AS1
17	76527001	76528000	CYGB;PRCD
17	76548001	76549000	CYGB;PRCD;SNHG16;SNORD1C
17	76576001	76577000	ST6GALNAC2
17	76609001	76611000	ST6GALNAC1;ST6GALNAC1
17	76638001	76639000	ST6GALNAC1
17	76649001	76650000	ST6GALNAC1;PTMAP13;RNU6-227P
17	76931001	76932000	MGAT5B
17	77136001	77137000	SEC14L1;LOC105371901
17	77248001	77249000	LACAT1
17	77270001	77271000	LACAT1;SEPTIN9-DT
17	77389001	77390000	SEPTIN9;MIR4316
17	77804001	77805000	LOC105371908
17	77824001	77825000	LOC105371908
17	77889001	77890000	LINC01973;LOC107985087
17	77917001	77918000	RNU1-80P;LOC105371909
17	77953001	77954000	TNRC6C
17	78042001	78043000	TNRC6C
17	78148001	78149000	TMC8;C17orf99;EIF5AP2
17	78212001	78213000	AFMID;BIRC5
17	78382001	78383000	SOCS3-DT;PGS1
17	78541001	78542000	DNAH17;RN7SL454P
17	78671001	78672000	LOC112268196;CYTH1
17	78768001	78769000	CYTH1
17	78806001	78807000	USP36
17	78844001	78845000	USP36;RPL9P29;TIMP2
17	79127001	79128000	RBFOX3
17	79249001	79250000	RBFOX3
17	79261001	79262000	RBFOX3
17	79268001	79269000	RBFOX3
17	79335001	79336000	RBFOX3
17	79367001	79368000	RBFOX3
17	79380001	79381000	RBFOX3
17	79491001	79492000	RBFOX3
17	79580001	79581000	RBFOX3
17	79617001	79618000	RBFOX3
17	79645001	79646000	RBFOX3
17	80162001	80163000	CARD14
17	80401001	80402000	RNF213;RNF213-AS1
17	80407001	80408000	RNF213;RNF213-AS1;ENDOV
17	80592001	80593000	RPTOR;LOC105371922;RPL31P7
17	80635001	80636000	RPTOR

17	80683001	80684000	RPTOR
17	80751001	80752000	RPTOR
17	80763001	80764000	RPTOR
17	80879001	80880000	RPTOR
17	81372001	81373000	LOC100130370
17	81477001	81478000	LOC112268189;LINC01971
17	81580001	81581000	NPLOC4
17	81625001	81626000	NPLOC4
17	81810001	81811000	LOC105376789;GCGR
17	82429001	82430000	HEXD;HEXD-IT1
17	82703001	82704000	RAB40B;LOC105376791;LOC101929552
17	82766001	82767000	TBCD
17	83108001	83109000	LOC107987251;LOC101930496
17	83127001	83128000	LOC101930496
17	83161001	83162000	LOC101929650
17	83218001	83219000	LOC101929650;LOC100505909;RPL23AP87
18	107001	108000	ROCK1P1;MIR8078
18	463001	464000	COLEC12
18	917001	918000	ADCYAP1;LINC01904
18	1785001	1786000	LOC105371959
18	2548001	2549000	METTL4
18	2668001	2669000	SMCHD1
18	3938001	3939000	DLGAP1
18	4049001	4050000	DLGAP1
18	4408001	4409000	DLGAP1
18	5518001	5519000	EPB41L3
18	5635001	5636000	EPB41L3;LOC100286986
18	6139001	6140000	L3MBTL4
18	6141001	6142000	L3MBTL4
18	6186001	6187000	L3MBTL4
18	6190001	6191000	L3MBTL4
18	6393001	6394000	L3MBTL4
18	6512001	6513000	LINC01387
18	6524001	6525000	LINC01387
18	6727001	6728000	ARHGAP28-AS1;ARHGAP28
18	7630001	7631000	PTPRM
18	7762001	7763000	PTPRM
18	8152001	8153000	PTPRM;LOC107985119
18	8354001	8355000	PTPRM;LOC100192426
18	9255001	9256000	ANKRD12
18	9815001	9816000	RAB31
18	9836001	9837000	RAB31;RN7SL862P;RNA5SP449
18	10372001	10373000	LOC105371986
18	10745001	10746000	PIEZO2
18	10763001	10764000	PIEZO2;MIR6788
18	10785001	10786000	PIEZO2
18	10898001	10899000	PIEZO2
18	11657001	11658000	MIR7153
18	12094001	12095000	LOC100533852;ANKRD62;RNU6-324P;SDHDP1
18	12423001	12424000	PRELID3A;LOC105371998
18	12477001	12478000	SPIRE1
18	12880001	12881000	PTPN2
18	12997001	12998000	SEH1L;CEP192
18	13233001	13234000	LDLRAD4;C18orf15
18	13400001	13401000	LDLRAD4
18	13428001	13429000	LDLRAD4;LDLRAD4-AS1
18	13461001	13462000	LDLRAD4;MIR5190
18	13745001	13746000	RNMT
18	13822001	13824000	MC5R;MC5R
18	14642001	14643000	LOC105372004;SNX19P3;GTF2IP8
18	14921001	14922000	LOC105372010
18	20938001	20939000	ROCK1
18	21019001	21020000	ROCK1
18	21108001	21109000	ROCK1
18	22321001	22322000	LOC101927548
18	22329001	22330000	LOC101927548
18	22985001	22986000	RBBP8
18	23151001	23152000	CABLES1
18	23738001	23739000	LAMA3
18	23779001	23780000	LAMA3
18	23830001	23831000	LAMA3
18	25316001	25317000	ZNF521
18	26528001	26529000	KCTD1
18	26643001	26644000	KCTD1;LOC102725227
18	27353001	27354000	LOC105372040;UBA52P9
18	31112001	31113000	DSC2;DSCAS
18	31170001	31171000	DSCAS;DSC1
18	34485001	34486000	DTNA
18	34702001	34703000	DTNA
18	35358001	35359000	ZNF396
18	35846001	35847000	NRBF2P1
18	35848001	35849000	NRBF2P1
18	36253001	36254000	MOCOS
18	36716001	36717000	FHOD3
18	36742001	36743000	FHOD3
18	37122001	37123000	KIAA1328
18	37247001	37248000	CELF4

18	37318001	37319000	CELF4
18	37414001	37415000	CELF4
18	37511001	37512000	CELF4
18	42463001	42465000	LINC00907;LINC00907
18	42556001	42557000	LINC00907
18	42737001	42738000	RIT2
18	42740001	42742000	RIT2;RIT2
18	42991001	42994000	RIT2;RIT2;RIT2
18	45422001	45423000	SLC14A2;SLC14A2-AS1
18	45841001	45842000	EPG5;SIGLEC15
18	46341001	46342000	RNF165
18	46398001	46399000	RNF165;LOC105372095
18	46557001	46558000	LOXHD1
18	46698001	46699000	ST8SIA5
18	46927001	46928000	PIAS2;KATNAL2
18	47541001	47542000	MIR4527HG
18	48320001	48321000	ZBTB7C
18	48360001	48361000	ZBTB7C
18	48602001	48603000	CTIF
18	48621001	48622000	CTIF
18	48856001	48857000	CTIF
18	48962001	48963000	LOC105372108
18	49019001	49020000	LOC100129878
18	49935001	49936000	MYO5B
18	49944001	49945000	MYO5B;RNA5SP457
18	50112001	50113000	MYO5B
18	50157001	50158000	MYO5B
18	50695001	50696000	MAPK4
18	50716001	50717000	MAPK4
18	51505001	51506000	LINC01630
18	51572001	51573000	LINC01630
18	54537001	54538000	DYNAP
18	55718001	55719000	LOC105372130
18	56730001	56731000	WDR7
18	57008001	57009000	WDR7
18	57068001	57069000	LINC-ROR
18	57764001	57765000	ATP8B1;RNU6-742P;MRPS17P7
18	58124001	58125000	NEDD4L
18	58131001	58132000	NEDD4L
18	58278001	58279000	NEDD4L
18	58375001	58376000	NEDD4L
18	58445001	58446000	MIR122HG;MIR122;MIR3591
18	60513001	60514000	MRPS5P4
18	62330001	62331000	TNFRSF11A
18	62543001	62544000	ZCCHC2
18	62987001	62988000	PHLPP1
18	63199001	63200000	BCL2
18	63209001	63210000	BCL2
18	67709001	67710000	DSEL-AS1;LOC105372173;FAM32DP
18	69902001	69903000	CD226
18	70751001	70752000	LOC105372185
18	72833001	72834000	NETO1
18	73325001	73326000	LINC02582
18	73327001	73328000	LINC02582
18	74138001	74139000	FBXO15;TIMM21
18	74246001	74247000	CYB5A
18	74268001	74269000	CYB5A
18	74426001	74427000	DIPK1C
18	74545001	74546000	CNDP1
18	74587001	74588000	CNDP1;ZNF407;ZNF407-AS1
18	74727001	74728000	ZNF407
18	74978001	74979000	ZNF407
18	75067001	75068000	ZNF407
18	76151001	76152000	LOC339298
18	76394001	76395000	ZNF516
18	76698001	76699000	LINC01879;ARL2BPP1
18	76792001	76793000	ZNF236-DT;RNU6-346P
18	76860001	76861000	ZNF236
18	77872001	77873000	RNA5SP461
18	78563001	78564000	LOC105372220
18	78629001	78630000	LOC105372221
18	79043001	79044000	LOC105372225
18	79297001	79298000	ATP9B;LOC105372226
18	79357001	79358000	ATP9B;LOC107985149
18	79425001	79426000	NFATC1
18	79534001	79535000	NFATC1;LOC102723506
18	79539001	79540000	NFATC1
18	79601001	79602000	LOC105372228
18	79649001	79650000	LOC284241
18	79742001	79743000	CTDP1
18	79885001	79888000	KCNG2;KCNG2;KCNG2
18	79919001	79920000	SLC66A2
18	79994001	79995000	TXNL4A
18	80018001	80019000	TXNL4A
19	78001	79000	WASH5P;LOC105376912;MIR1302-11;FAM138F
19	302001	303000	MIER2
19	376001	377000	THEG

19	396001	397000	C2CD4C
19	444001	445000	SHC2;RNA5SP462
19	514001	515000	MADCAM1;MADCAM1-AS1;TPGS1
19	545001	546000	CDC34;GZMM
19	738001	739000	PALM;MISP
19	841001	842000	AZU1;PRTN3;ELANE
19	892001	893000	MED16;RNU6-9;R3HDM4
19	1657001	1658000	TCF3
19	2013001	2015000	BTBD2;LOC107985278;BTBD2;LOC107985278
19	2300001	2301000	LINGO3;LOC101928572
19	2365001	2366000	SPPL2B
19	2419001	2420000	TMPRSS9;TIMM13;LMNB2
19	2602001	2603000	GNG7;RN7SL121P
19	2695001	2696000	GNG7
19	2769001	2770000	SGTA
19	2801001	2802000	THOP1
19	3188001	3189000	S1PR4;NCLN
19	3409001	3410000	NFIC
19	3419001	3420000	NFIC
19	3459001	3460000	NFIC
19	3480001	3481000	SMIM24;LOC105372246;DOHH
19	3647001	3648000	PIP5K1C
19	3784001	3785000	MATK
19	3875001	3876000	ZFR2;FTLP5;ATCAY
19	3938001	3939000	ATCAY;NMRK2
19	4121001	4122000	MAP2K2
19	4197001	4199000	ANKRD24;ANKRD24
19	4266001	4267000	YJU2
19	4318001	4319000	FSD1;STAP2
19	4426001	4427000	CHAF1A
19	4662001	4663000	TNFAIP8L1;MYDGF
19	5090001	5091000	KDM4B
19	5247001	5248000	PTPRS
19	5296001	5297000	PTPRS;LOC105372253
19	5304001	5305000	PTPRS;LOC105372253
19	5329001	5330000	PTPRS;LOC105372252
19	5551001	5553000	TINCR;TINCR
19	5733001	5734000	CATSPERD
19	5849001	5850000	FUT6;FUT3;LOC101928844
19	5857001	5858000	FUT3;LOC101928844;FUT5
19	5890001	5891000	NDUFA11
19	6150001	6151000	LOC105372255;ACSBG2
19	6161001	6162000	LOC105372255;ACSBG2
19	6235001	6236000	MLLT1
19	6534001	6535000	TNFSF9
19	6541001	6542000	TNFSF9
19	6586001	6587000	CD70;RPL7P50
19	6678001	6679000	TNFSF14;C3
19	6693001	6694000	C3
19	6718001	6719000	C3
19	6731001	6732000	GPR108;MIR6791;TRIP10
19	6777001	6778000	SH2D3A;VAV1
19	6824001	6825000	VAV1
19	6877001	6878000	ADGRE1
19	6924001	6925000	ADGRE1;LOC105372256
19	7031001	7032000	MBD3L2B;MBD3L5;MBD3L4
19	7255001	7256000	INSR
19	7268001	7269000	INSR
19	7289001	7290000	INSR
19	7460001	7461000	ARHGEF18
19	7644001	7645000	PCP2;STXBP2
19	8328001	8329000	NDUFA7;RPS28;KANK3
19	8363001	8364000	ANGPTL4
19	8500001	8501000	PRAM1;ZNF414
19	8536001	8537000	MYO1F
19	8574001	8575000	MYO1F;ADAMTS10
19	8662001	8663000	NFILZ
19	8676001	8677000	NFILZ
19	8932001	8933000	MUC16
19	9046001	9047000	TRQ-TTG8-1
19	9395001	9396000	LOC112268250
19	9604001	9605000	LOC105376914;ZNF561
19	9641001	9642000	ZNF561-AS1;ZNF562
19	9742001	9744000	ZNF846;ZNF846
19	9863001	9864000	OLFM2
19	9886001	9887000	OLFM2
19	9930001	9931000	OLFM2
19	9967001	9968000	COL5A3
19	9970001	9973000	COL5A3;COL5A3;COL5A3
19	9974001	9975000	COL5A3
19	10011001	10012000	COL5A3;RDH8
19	10032001	10033000	RDH8;MIR5589;C3P1
19	10070001	10071000	C3P1
19	10239001	10240000	S1PR2;MIR4322
19	10488001	10489000	KEAP1
19	10540001	10541000	ATG4D
19	10991001	10992000	SMARCA4

19	11177001	11178000	KANK2
19	11229001	11230000	DOCK6;ANGPTL8
19	11261001	11262000	DOCK6
19	11358001	11359000	CCDC159;PLPPR2
19	11465001	11466000	ELAVL3
19	11495001	11496000	ZNF653;MIR7974;ECSIT
19	11510001	11511000	ZNF653;ECSIT;RN7SL833P
19	11513001	11514000	ZNF653;ECSIT;RN7SL833P;RPL18AP13;RPS12P32
19	11544001	11545000	CNN1;ELOF1
19	11607001	11608000	ZNF627
19	12872001	12873000	MAST1;DNASE2
19	12983001	12984000	DAND5
19	13208001	13209000	LOC107985288;CACNA1A
19	13230001	13231000	CACNA1A
19	13278001	13279000	CACNA1A
19	13806001	13807000	ZSWIM4;LOC107985334;RN7SL619P
19	13816001	13817000	ZSWIM4;LOC107985334;RN7SL619P
19	13929001	13930000	CC2D1A;PODNL1
19	13989001	13990000	RFX1;LOC107985309
19	14175001	14176000	ADGRL1-AS1;ADGRL1
19	14460001	14462000	PKN1;PKN1
19	14474001	14475000	PKN1;PTGER1;GIPC1
19	14567001	14568000	TECR;NDUFB7
19	14729001	14730000	ZNF333;ADGRE2
19	14773001	14774000	ADGRE2
19	15051001	15052000	CASP14
19	15884001	15885000	CYP4F2
19	16368001	16369000	EPS15L1
19	16880001	16881000	SIN3B;F2RL3
19	17146001	17147000	MYO9B
19	17432001	17433000	MVB12A;TMEM221
19	17538001	17539000	NIBAN3
19	17597001	17598000	RPL21P130;UNC13A
19	17631001	17632000	UNC13A
19	17674001	17675000	UNC13A
19	18019001	18020000	ARRDC2
19	18347001	18348000	PGPEP1
19	18647001	18648000	KLHL26
19	18770001	18771000	CRTC1
19	18803001	18804000	LOC107985321
19	18879001	18880000	CERS1;GDF1
19	19000001	19001000	SUGP2
19	19091001	19092000	SLC25A42
19	19207001	19208000	RFXANK;NR2C2AP;NCAN
19	19431001	19432000	GATAD2A;MIR640
19	19440001	19441000	GATAD2A;MIR640
19	19534001	19535000	NDUFA13;YJEFN3;CILP2
19	19949001	19950000	BNIP3P12
19	20213001	20214000	LOC105372310;LOC105372311;BNIP3P17
19	20476001	20477000	LOC105372316
19	21132001	21133000	ZNF714;VN1R81P;ZNF431
19	23026001	23027000	LINC01859
19	23031001	23032000	LINC01859
19	27844001	27845000	LOC101927151;LOC105372348
19	28432001	28433000	LOC100420587
19	28459001	28460000	LOC100420587
19	29307001	29308000	VSTM2B-DT
19	29696001	29697000	C19orf12
19	29698001	29699000	C19orf12
19	30278001	30279000	ZNF536
19	31287001	31288000	TSHZ3
19	31552001	31553000	LINC02841
19	32729001	32730000	LOC105372368;TDRD12
19	32847001	32848000	SLC7A9
19	32944001	32945000	CEP89
19	32982001	32983000	CEP89;FAAP24;RHPN2
19	33142001	33143000	WDR88
19	33383001	33384000	CEBPG;PEPD
19	33493001	33494000	PEPD
19	33506001	33507000	PEPD
19	33513001	33515000	PEPD;LOC105372370;PEPD;LOC105372370
19	34325001	34326000	GARRE1
19	34504001	34505000	WTIP
19	34781001	34782000	ZNF599
19	34869001	34870000	LOC401913
19	34961001	34962000	ZNF792
19	34999001	35000000	GRAMD1A
19	35005001	35006000	GRAMD1A
19	35045001	35046000	SCN1B;HPN
19	35075001	35076000	HPN;HPN-AS1;LOC100421064;LOC100420797
19	35077001	35078000	HPN-AS1;LOC100421064;LOC100420797
19	35079001	35080000	HPN-AS1;LOC100421064;LOC100420797
19	35104001	35105000	HPN-AS1
19	35134001	35135000	FXYD3;LGI4;FXYD1;FXYD7
19	35342001	35343000	CD22;MIR5196;FFAR1
19	35375001	35376000	GPR42;EEF1A1P7
19	35527001	35528000	SBSN;GAPDHS

19	35655001	35656000	COX6B1
19	35676001	35677000	UPK1A;UPK1A-AS1;TYMSP2
19	35734001	35735000	KMT2B;IGFLR1;U2AF1L4
19	35755001	35756000	U2AF1L4;PSENEN;LIN37;HSPB6;PROSER3
19	35779001	35780000	PROSER3;ARHGAP33;LINC01529
19	35797001	35798000	ARHGAP33;LINC01529;PRODH2
19	35854001	35855000	NPHS1;KIRREL2;LOC107985317
19	35870001	35871000	KIRREL2;LOC107985317;APLP1
19	35876001	35877000	KIRREL2;LOC107985317;APLP1;RN7SL402P
19	35957001	35958000	LOC105372383
19	36028001	36029000	LOC101927572;CLIP3;THAP8
19	36080001	36081000	WDR62
19	36156001	36158000	CAPNS1;COX7A1;CAPNS1;COX7A1
19	36401001	36402000	ZFP82
19	37375001	37376000	ZNF527
19	37377001	37378000	ZNF527
19	37534001	37535000	ZNF793
19	37857001	37858000	WDR87BP;LOC105372395
19	37942001	37943000	SIPA1L3
19	38044001	38046000	SIPA1L3;SIPA1L3
19	38061001	38062000	SIPA1L3
19	38196001	38197000	SIPA1L3;RN7SL663P
19	38233001	38234000	DPF1
19	38245001	38246000	PPP1R14A
19	38420001	38421000	RASGRP4
19	38441001	38442000	RYR1
19	38449001	38450000	RYR1
19	38464001	38465000	RYR1
19	38486001	38487000	RYR1
19	38500001	38501000	RYR1
19	38508001	38509000	RYR1
19	38511001	38512000	RYR1
19	38526001	38527000	RYR1;LOC107985290
19	38536001	38537000	RYR1;LOC107985290
19	38546001	38547000	RYR1
19	38572001	38573000	RYR1
19	38584001	38585000	RYR1;MAP4K1
19	38607001	38608000	MAP4K1;MAP4K1-AS1
19	38613001	38614000	MAP4K1;EIF3K
19	38752001	38753000	CAPN12
19	38801001	38802000	LGALS7B;RNU6-140P;LGALS4
19	38816001	38817000	LGALS4;ECH1
19	38952001	38953000	FBXO17
19	39076001	39077000	ACP7
19	39134001	39135000	PAK4
19	39275001	39276000	IFNL4P1;IFNL2
19	39277001	39278000	IFNL2
19	39297001	39298000	IFNL1;LRFN1
19	39343001	39345000	GMFG;SAMD4B;GMFG;SAMD4B
19	39452001	39453000	SUPT5H
19	39474001	39475000	SUPT5H;SNORD175;TIMM50
19	39819001	39820000	DYRK1B;MIR6719
19	39877001	39878000	FCGBP
19	40225001	40226000	MAP3K10;TTC9B;CCNP;AKT2
19	40274001	40275000	AKT2;MIR641
19	40320001	40322000	C19orf47;C19orf47
19	40504001	40505000	SPTBN4
19	40527001	40529000	SPTBN4;SPTBN4
19	40588001	40590000	SHKBP1;LTBP4;SHKBP1;LTBP4
19	40764001	40765000	SNRPA
19	40781001	40782000	MIA;MIA-RAB4B;RAB4B-EGLN2;RAB4B
19	40821001	40822000	CYP2T1P;CYP2F2P
19	40936001	40937000	CYP2B7P
19	41007001	41008000	CYP2B6
19	41026001	41027000	CYP2B6;CYP2A7P1
19	41060001	41061000	CYP2G2P
19	41117001	41118000	CYP2F1
19	41131001	41132000	CYP2F1;CYP2T3P
19	41196001	41197000	RN7SL718P;CYP2S1
19	41323001	41324000	CCDC97;TGFB1
19	41422001	41423000	BCKDHA;B3GNT8;DMAC2
19	41888001	41889000	CD79A;ARHGEF1
19	41925001	41926000	ERFL;LOC100505585
19	41967001	41968000	RABAC1;ATP1A3
19	42002001	42003000	ATP1A3;GRIK5
19	42021001	42022000	GRIK5
19	42130001	42131000	POU2F2;POU2F2-AS1;MIR4323
19	42335001	42336000	TMEM145;MEGF8
19	42363001	42364000	MEGF8
19	42383001	42384000	MEGF8;CNFN
19	42406001	42407000	LOC101930071;LIPE-AS1;LIPE
19	43741001	43742000	SMG9
19	43783001	43784000	KCNN4
19	44453001	44454000	ZNF229
19	44528001	44529000	CEACAM20;CEACAM22P
19	44759001	44760000	BCL3;MIR8085
19	44799001	44800000	CBLC;BCAM

19	44848001	44850000	NECTIN2;NECTIN2
19	44870001	44871000	NECTIN2
19	45087001	45088000	GEMIN7;GEMIN7-AS1;PPP1R37
19	45160001	45161000	NKPD1;LOC105372420;TRAPPC6A
19	45237001	45239000	EXOC3L2;LOC100420902;EXOC3L2;LOC100420902
19	45317001	45318000	CKM
19	45342001	45343000	RPS16P9;KLC3;ERCC2
19	45422001	45423000	ERCC1
19	45489001	45490000	RTN2;PPM1N
19	45517001	45518000	VASP;LOC107985315;OPA3
19	45622001	45623000	EML2
19	45658001	45659000	RN7SL836P;GIPR
19	45662001	45663000	GIPR
19	45858001	45859000	SYMPK;FOXA3
19	45876001	45877000	FOXA3;IRF2BP1
19	45933001	45934000	NOVA2
19	45968001	45969000	NOVA2
19	45988001	45989000	CCDC61
19	46010001	46011000	CCDC61;MIR769;PGLYRP1
19	46106001	46107000	IGFL2;LOC100533846
19	46108001	46109000	IGFL2;LOC100533846
19	46630001	46631000	PTGIR;GNG8
19	46695001	46696000	PRKD2;RN7SL364P
19	46697001	46698000	PRKD2;RN7SL364P
19	46727001	46728000	PRKD2;LOC107987269;STRN4
19	46766001	46767000	FKRP;SLC1A5
19	46858001	46859000	AP2S1;ARHGAP35
19	47134001	47135000	SAE1
19	47270001	47271000	CCDC9;INAFM1;LOC105372427
19	47350001	47351000	C5AR2;DHX34
19	47393001	47394000	MEIS3
19	47398001	47399000	MEIS3
19	47403001	47404000	MEIS3
19	47443001	47444000	SLC8A2
19	47468001	47469000	SLC8A2;KPTN
19	47728001	47729000	EHD2
19	47781001	47782000	SELENOW
19	47842001	47843000	CRX
19	47875001	47876000	LINC01595;SULT2A1
19	47911001	47912000	SNAR-A12;SNAR-C5;SNAR-A1
19	47948001	47949000	SNAR-C4;SNAR-A13;SNAR-C3;SNAR-C1
19	48011001	48012000	ELSPBP1
19	48128001	48129000	LIG1
19	48144001	48145000	LIG1;LOC107985293
19	48160001	48161000	LIG1;LOC107985293;ZSWIM9
19	48228001	48229000	CARD8
19	48279001	48280000	ZNF114-AS1;ZNF114
19	48396001	48397000	KDELR1;LOC107984140;GRIN2D
19	48399001	48400000	KDELR1;LOC107984140;GRIN2D
19	48415001	48416000	GRIN2D
19	48463001	48464000	GRWD1;KCNJ14;CYTH2
19	48484001	48485000	CYTH2;LMTK3
19	48492001	48493000	CYTH2;LMTK3
19	48503001	48504000	LMTK3
19	48516001	48517000	LMTK3
19	48707001	48708000	FUT2;LOC105447645;MAMSTR
19	48821001	48822000	BCAT2;HSD17B14
19	48995001	48996000	GYS1;RUVBL2
19	49069001	49070000	NTF4;KCNA7
19	49160001	49161000	PPFIA3;HRC;TRPM4
19	49167001	49168000	TRPM4
19	49364001	49366000	TEAD2;DKKL1;LOC101928295;TEAD2;DKKL1;LOC101928295
19	49412001	49413000	KASH5;PTH2
19	49427001	49428000	KASH5;PTH2;GFY;SLC17A7
19	49481001	49482000	ALDH16A1;FLT3LG;RPL13A;SNORD32A;SNORD33;SNORD34;SNORD35A
19	49511001	49512000	COX6CP7;FCGRT
19	49525001	49526000	FCGRT;RCN3
19	49544001	49545000	RCN3
19	49594001	49595000	PRRG2;PRR12
19	49615001	49616000	PRR12
19	49621001	49622000	PRR12
19	49623001	49624000	PRR12
19	49763001	49764000	TSKS;LOC105372434;RNU6-841P;AP2A1
19	49769001	49770000	TSKS;RNU6-841P;AP2A1
19	49780001	49781000	AP2A1
19	49893001	49894000	TBC1D17;MIR4750;IL4I1
19	49933001	49934000	IL4I1;NUP62;ATF5;MIR4751
19	49973001	49974000	SIGLEC16;VRK3
19	49994001	49995000	VRK3
19	50072001	50074000	LOC400710;LOC400710
19	50154001	50155000	LOC105372436;IZUMO2
19	50267001	50268000	MYH14
19	50319001	50320000	MYH14;KCNC3;LOC105372437
19	50436001	50437000	SPIB;MYBPC2
19	50487001	50488000	EMC10
19	50508001	50509000	EMC10;JOSD2;ASPDH;LRRC4B
19	50578001	50579000	LRRC4B

19	50661001	50663000	C19orf81;SHANK1;C19orf81;SHANK1
19	50715001	50716000	SHANK1;CLEC11A
19	50756001	50757000	GPR32P1
19	50791001	50792000	LOC105372440;LOC105372439;ACP4;C19orf48;SNORD88B;SNORD88A
19	50822001	50823000	LINC01869;KLK1;KLK15;LOC105372441
19	50826001	50827000	LINC01869;KLK1;KLK15;LOC105372441
19	50832001	50833000	KLK1;KLK15;LOC105372441
19	50949001	50950000	KLK5;KLK6;LOC105372442
19	50970001	50971000	KLK6;LOC105372442;KLK7
19	51018001	51019000	KLK9;KLK10;KLK11;KLK12
19	51082001	51083000	KLK14
19	51112001	51113000	CTU1;SIGLEC18P
19	51323001	51324000	IGLON5;VSIG10L
19	51325001	51326000	IGLON5;VSIG10L
19	51380001	51381000	NKG7;LIM2;LOC105372446;C19orf84
19	51412001	51413000	LOC105372446;NIFKP6;SIGLEC10;SIGLEC10-AS1
19	51578001	51579000	ZNF175
19	51591001	51592000	ZNF175;LINC01530;LOC339352
19	51595001	51596000	ZNF175;LINC01530;LOC339352
19	51695001	51696000	SPACA6P-AS;SPACA6;MIR99B;MIRLET7E;MIR125A
19	51706001	51707000	SPACA6;HAS1
19	51716001	51717000	SPACA6;HAS1
19	51725001	51726000	HAS1
19	51796001	51797000	FPR3;ZNF577
19	52094001	52095000	ZNF841
19	52119001	52120000	ZNF616
19	52133001	52134000	ZNF616;RPL37P23
19	52493001	52494000	ZNF578
19	52624001	52625000	ZNF701;ZNF83
19	52630001	52632000	ZNF701;ZNF83;ZNF701;ZNF83
19	52636001	52637000	ZNF701;ZNF83
19	52719001	52720000	ZNF611
19	52756001	52757000	LOC105372452;LOC105372451;ZNF600
19	52820001	52821000	ZNF28;PABPN1P2
19	53273001	53274000	VN1R4;FAM90A27P
19	53340001	53341000	ZNF845
19	53479001	53480000	ZNF813;TPM3P6
19	53832001	53833000	NLRP12
19	53834001	53835000	NLRP12
19	53892001	53893000	PRKCG
19	53983001	53984000	CACNG8;MIR935;CACNG6
19	53986001	53987000	CACNG8;MIR935;CACNG6
19	54106001	54107000	OSCAR;NDUFA3;TFPT;PRPF31
19	54144001	54145000	CNOT3;LOC102724273
19	54505001	54506000	LAIR2;LOC105372460
19	54545001	54546000	KIR3DX1;LOC107985347
19	54724001	54725000	KIR3DL3
19	54817001	54818000	KIR2DL4;KIR3DL1
19	54833001	54834000	KIR3DL1;KIR2DS4
19	54896001	54897000	FCAR;NCR1
19	54898001	54899000	FCAR;NCR1
19	54900001	54901000	FCAR;NCR1
19	55008001	55010000	NLRP2;GP6-AS1;GP6;NLRP2;GP6-AS1;GP6
19	55011001	55012000	NLRP2;GP6-AS1;GP6
19	55084001	55085000	EPS8L1;PPP1R12C
19	55125001	55126000	PPP1R12C;MIR7975;TNNT1
19	55128001	55129000	MIR7975;TNNT1
19	55131001	55132000	MIR7975;TNNT1
19	55133001	55134000	MIR7975;TNNT1
19	55144001	55145000	TNNT1;TNNI3
19	55151001	55152000	TNNT1;TNNI3;DNAAF3;DNAAF3-AS1
19	55154001	55155000	TNNT1;TNNI3;DNAAF3;DNAAF3-AS1
19	55200001	55201000	PTPRH
19	55286001	55287000	HSPBP1;BRSK1
19	55545001	55547000	SBK2;SBK3;SBK2;SBK3
19	55553001	55554000	SBK3
19	55579001	55580000	ZNF579
19	55671001	55672000	U2AF2;EPN1
19	56148001	56149000	ZNF444
19	56176001	56177000	GALP
19	56183001	56184000	GALP;ZSCAN5B
19	56691001	56692000	LOC105372473;LOC105372472
19	56765001	56766000	LOC105372472;OR5AH1P;ZIM2-AS1;ZIM2
19	57137001	57138000	USP29;ZIM3
19	57352001	57353000	ZNF304
19	57369001	57370000	ZNF304;ZNF547;TRAPPC2B
19	57395001	57396000	ZNF548
19	57415001	57416000	ZNF17
19	57683001	57684000	ZSCAN4;ZNF551
19	57835001	57836000	FKBP1AP1;ZNF587B
19	57961001	57962000	RPL19P19;C19orf18
19	58097001	58098000	ZSCAN18
20	177001	178000	DEFB128
20	356001	358000	LOC101929937;NRSN2;LOC101929937;NRSN2
20	915001	917000	ANGPT4;LOC105372492;ANGPT4;LOC105372492
20	1187001	1188000	TMEM74B;LOC105372496
20	1727001	1728000	SIRPB3P

20	1732001	1733000	SIRPB3P
20	1861001	1862000	LOC107984088
20	1906001	1907000	SIRPA
20	1937001	1938000	SIRPA;PDYN-AS1
20	1990001	1991000	PDYN-AS1;PDYN
20	2130001	2131000	STK35
20	2212001	2213000	LOC388780
20	2472001	2473000	SNRPB;SNORD119;ZNF343
20	2691001	2692000	LOC105372507;EBF4
20	2822001	2823000	C20orf141;TMEM239
20	2845001	2846000	PCED1A;VPS16
20	2865001	2866000	VPS16;PTPRA
20	3032001	3033000	PTPRA;GNRH2
20	3088001	3089000	AVP;RN7SL555P
20	3126001	3127000	UBOX5-AS1;UBOX5
20	3195001	3196000	DDRGK1
20	3534001	3535000	ATRN
20	3977001	3978000	RNF24
20	4229001	4230000	ADRA1D
20	4881001	4882000	SLC23A2
20	4983001	4984000	SLC23A2
20	4995001	4996000	SLC23A2
20	5922001	5923000	CHGB;KANK1P1
20	6012001	6013000	MCM8-AS1;RN7SL498P;CRLS1
20	6063001	6064000	LRRN4
20	7996001	7997000	TMX4
20	8370001	8371000	PLCB1
20	9304001	9305000	PLCB4
20	10212001	10213000	SNAP25-AS1;SNAP25
20	10550001	10551000	SLX4IP
20	10616001	10617000	SLX4IP;LOC100421490
20	10859001	10860000	LOC105372525
20	11695001	11696000	LOC107985384
20	13362001	13363000	TASP1
20	13460001	13461000	TASP1
20	13861001	13862000	SEL1L2
20	14414001	14415000	MACROD2
20	14538001	14539000	MACROD2;RNF11P2
20	15151001	15153000	MACROD2;MACROD2
20	15497001	15498000	MACROD2
20	16339001	16340000	KIF16B
20	16537001	16538000	KIF16B
20	17745001	17746000	BANF2
20	17913001	17914000	RNU6-192P;LOC105372549
20	18083001	18084000	RPL15P1
20	18176001	18177000	KAT14
20	18273001	18274000	RNU2-56P;LOC105372551
20	18519001	18520000	SEC23B
20	18602001	18603000	DTD1;RN7SL638P;RNU6ATAC34P
20	19328001	19329000	SLC24A3
20	19469001	19470000	SLC24A3
20	19752001	19755000	LOC107987276;RIN2;LOC107987276;RIN2;LOC107987276;RIN2
20	20078001	20079000	CFAP61
20	20468001	20469000	RALGAPA2
20	20633001	20634000	RALGAPA2
20	20754001	20755000	LOC105372555
20	21142001	21143000	KIZ;RNA5SP477
20	21573001	21574000	LINC01727
20	22519001	22520000	LOC105372564
20	23088001	23089000	CD93
20	23310001	23311000	NXT1-AS1;LOC105372571
20	24242001	24243000	RNU1-23P
20	24335001	24336000	LOC105372577
20	24617001	24619000	SYNDIG1;SYNDIG1
20	25217001	25218000	ENTPD6
20	29085001	29086000	FRG1DP
20	29756001	29757000	LOC107987278;CDC27P3;DUX4L36
20	30518001	30519000	LOC110467526;LOC105379481
20	30520001	30521000	LOC110467526;LOC105379481
20	30698001	30699000	ANKRD20A21P
20	31468001	31469000	DEFB124;REM1
20	31779001	31780000	TPX2
20	31825001	31827000	MYLK2;MYLK2
20	31833001	31834000	MYLK2
20	31856001	31857000	DUSP15
20	31924001	31925000	TTLL9
20	31983001	31984000	XKR7
20	32051001	32052000	RNA5SP482;HCK
20	32092001	32093000	HCK
20	32131001	32132000	TM9SF4
20	32139001	32140000	TM9SF4
20	32544001	32545000	NOL4L;LOC105372592
20	32562001	32563000	NOL4L
20	32568001	32569000	NOL4L
20	32575001	32577000	NOL4L;NOL4L
20	32779001	32780000	DNMT3B
20	32971001	32972000	EFCAB8

20	33025001	33026000	BPIFB2;BPIFB6
20	33099001	33100000	BPIFB4
20	33681001	33682000	NECAB3;E2F1
20	34085001	34086000	RALY;EIF2S2
20	34291001	34292000	AHCY
20	34997001	34998000	MYH7B;LOC107985393;MIR499A;MIR499B;TRPC4AP
20	35059001	35060000	TRPC4AP
20	35495001	35496000	CEP250;CEP250-AS1
20	35584001	35585000	FER1L4;RPL37P1
20	35597001	35598000	FER1L4;RPL37P1
20	35654001	35655000	CPNE1;RNU6-759P;RBM12
20	36124001	36125000	EPB41L1
20	36217001	36218000	EPB41L1;LOC105372602
20	36225001	36226000	EPB41L1;LOC105372602
20	36360001	36362000	DLGAP4;DLGAP4
20	36458001	36459000	DLGAP4
20	36627001	36628000	SLA2;HNRNPA3P2
20	37193001	37195000	RPN2;RPN2
20	37341001	37342000	LOC105372606;SRC
20	37449001	37450000	RPL7AP14
20	37529001	37530000	BLCAP;NNAT;PPIAP3
20	37846001	37847000	CTNNBL1
20	38043001	38044000	TTI1;RPRD1B
20	38141001	38142000	TGM2
20	38148001	38149000	TGM2
20	38589001	38590000	ADIG
20	38593001	38595000	ADIG;ARHGAP40;ADIG;ARHGAP40
20	38769001	38770000	ACTR5;RN7SKP173
20	38845001	38846000	PPP1R16B
20	38903001	38904000	PPP1R16B
20	38974001	38975000	DHX35;NPM1P19
20	40503001	40504000	LOC107985446
20	40515001	40516000	LOC107985446
20	41410001	41411000	CHD6
20	41559001	41560000	CHD6
20	42356001	42357000	PTPRT
20	42781001	42782000	PTPRT
20	43427001	43428000	EIF4EBP2P1
20	43547001	43548000	L3MBTL1
20	43775001	43776000	LOC101927200
20	43931001	43932000	TOX2
20	44132001	44133000	JPH2
20	44142001	44143000	JPH2
20	44158001	44159000	JPH2
20	44272001	44274000	GDAP1L1;GDAP1L1
20	44399001	44400000	HNF4A;HNF4A-AS1;MIR3646
20	44412001	44413000	HNF4A;MIR3646
20	44613001	44614000	PKIG;ADA
20	44774001	44775000	RIMS4
20	44953001	44954000	TOMM34;STK4-AS1
20	45585001	45586000	WFDC8
20	45760001	45761000	WFDC3;SPINT5P
20	45763001	45765000	WFDC3;WFDC3
20	45804001	45805000	DNTTIP1;UBE2C
20	46049001	46050000	SLC12A5
20	46263001	46264000	CDH22
20	46265001	46266000	CDH22
20	46303001	46304000	CDH22
20	46563001	46564000	SLC13A3
20	47160001	47161000	EYA2;RPS2P54;MIR3616
20	47707001	47708000	SULF2
20	47723001	47724000	SULF2
20	47743001	47744000	SULF2
20	47750001	47751000	SULF2
20	47801001	47802000	LOC107985401
20	47852001	47853000	LOC105372636;SRMP1
20	47866001	47867000	SRMP1;RNA5SP486
20	47895001	47897000	LOC105376980;RNU7-92P;LOC105376980;RNU7-92P
20	48120001	48121000	LOC105372640
20	48123001	48125000	LOC105372640;LOC105372640
20	48516001	48517000	LOC105372647;LOC105372646
20	48545001	48546000	LOC105372646
20	48669001	48670000	PREX1
20	48690001	48691000	PREX1
20	48741001	48742000	PREX1
20	48789001	48790000	PREX1
20	48875001	48876000	LOC105372648
20	48911001	48912000	ARFGEF2
20	49402001	49403000	LOC105372649;KCNB1
20	49519001	49520000	PTGIS
20	49543001	49544000	PTGIS
20	49632001	49633000	B4GALT5
20	50298001	50299000	LINC01270
20	50610001	50611000	RIPOR3;MIR1302-5;RPL36P2
20	50684001	50685000	RIPOR3;LOC105372659
20	50855001	50856000	BCAS4
20	50999001	51000000	KCNG1

20	51553001	51554000	NFATC2
20	51904001	51905000	RNU6-347P;RNU7-6P
20	52307001	52308000	LOC105372666
20	53012001	53013000	TSHZ2
20	53214001	53215000	TSHZ2
20	54541001	54542000	DOK5
20	56389001	56390000	AURKA;CSTF1
20	56575001	56576000	LINC01716
20	57198001	57199000	BMP7
20	57357001	57359000	RAE1;MTND1P9;NMTRL-TAA5-1;MTRNR2L3;RAE1;MTND1P9;NMTRL-TAA5-1;MTRNR2L3
20	57525001	57527000	CTCFL;CTCFL
20	57533001	57534000	CTCFL
20	58090001	58091000	HSPD1P19
20	58097001	58098000	HSPD1P19
20	58227001	58228000	ANKRD60;PPP4R1L
20	58268001	58269000	PPP4R1L
20	58315001	58316000	PPP4R1L;RAB22A
20	58426001	58427000	VAPB
20	58557001	58558000	APCDD1L-DT
20	58721001	58722000	STX16-NPEPL1;NPEPL1
20	58871001	58872000	GNAS;LOC101927932
20	59004001	59005000	NELFCD;CTSZ
20	59662001	59663000	PHACTR3
20	59721001	59722000	PHACTR3
20	61458001	61459000	CDH4
20	61484001	61485000	CDH4
20	61573001	61574000	CDH4
20	61727001	61728000	CDH4;LOC100128310
20	62106001	62107000	LOC105372706
20	62373001	62374000	LAMA5;LOC105372708
20	62431001	62432000	RBBP8NL;LOC105372710
20	62498001	62499000	LOC105372711
20	62636001	62637000	SLCO4A1
20	62934001	62935000	DIDO1;GID8
20	63543001	63544000	PTK6;SRMS;FNDC11
20	63757001	63758000	ZBTB46
20	64088001	64089000	RGS19;OPRL1;LKAAEAR1
20	64095001	64096000	OPRL1;NPBWR2
20	64097001	64098000	OPRL1;NPBWR2
20	64142001	64143000	LOC105372727
20	64180001	64181000	MYT1
20	64327001	64328000	IQSEC3P3
21	8828001	8829000	LOC107985475
21	9338001	9339000	LOC101930100
21	9361001	9362000	LOC101930100;LOC101927345;SNX18P12
21	9699001	9700000	LOC105379513
21	9775001	9777000	LINC01667;LINC01667
21	10336001	10337000	EIF3FP1
21	10425001	10426000	BAGE2
21	10441001	10442000	BAGE2
21	10451001	10452000	BAGE2
21	10596001	10597000	TPTE
21	13359001	13360000	FGF7P2
21	13855001	13856000	CYP4F29P
21	13897001	13898000	SNX18P13
21	14928001	14929000	LINC02246
21	25988001	25989000	APP
21	26035001	26036000	APP
21	26229001	26230000	MARCKSP1
21	26425001	26426000	CYYR1-AS1;LOC105372758
21	26428001	26429000	CYYR1-AS1;LOC105372758
21	26855001	26856000	ADAMTS1;LOC105372760
21	29051001	29052000	USP16;CCT8
21	30208001	30209000	LINC00307;CLDN8
21	30374001	30375000	KRTAP13-2;MIR4327
21	31118001	31119000	TIAM1
21	31257001	31258000	TIAM1;LOC105372777
21	31409001	31411000	TIAM1;TIAM1
21	31913001	31914000	HUNK
21	32501001	32502000	EVA1C;EXOSC3P1
21	32587001	32588000	CFAP298-TCP10L;TCP10L;LOC105372782
21	32590001	32591000	CFAP298-TCP10L;TCP10L;LOC105372782;CFAP298
21	33169001	33170000	LINC01548;LOC105372787
21	33739001	33740000	ITSN1
21	33862001	33863000	ITSN1
21	34054001	34055000	LOC105372790
21	34123001	34124000	MRPS6
21	34531001	34532000	RCAN1
21	34923001	34924000	RUNX1
21	35498001	35499000	LOC100506403
21	36258001	36259000	DOP1B
21	36277001	36278000	DOP1B
21	36482001	36483000	LOC105369301;CLDN14;PSMD4P1
21	36525001	36526000	CLDN14
21	36817001	36818000	HLCS;LOC105369305
21	36902001	36903000	HLCS
21	37245001	37246000	VPS26C

21	37829001	37830000	KCNJ6
21	38397001	38398000	ERG
21	38437001	38438000	ERG
21	38595001	38596000	ERG
21	38667001	38668000	ERG
21	39012001	39013000	LOC102724740;LOC105372803
21	39175001	39176000	PCBP2P1;PSMG1;BRWD1
21	39476001	39477000	GET1-SH3BGR;SH3BGR
21	39632001	39633000	B3GALT5;IGSF5
21	39664001	39666000	B3GALT5;IGSF5;B3GALT5;IGSF5
21	39894001	39895000	PCP4
21	40334001	40335000	DSCAM
21	40348001	40349000	DSCAM
21	40384001	40385000	DSCAM;DSCAM-AS1
21	40686001	40687000	DSCAM
21	40751001	40752000	DSCAM
21	41387001	41388000	MX2
21	41469001	41470000	MX1;TMPRSS2
21	41493001	41494000	TMPRSS2
21	41633001	41634000	LOC105372812
21	41864001	41865000	PRDM15
21	42144001	42145000	UMODL1
21	42352001	42354000	TFF2;LOC105372815;TFF1;TFF2;LOC105372815;TFF1
21	42592001	42593000	LINC01671
21	42651001	42652000	LOC101928255;PDE9A
21	43179001	43181000	LOC107987300;CRYAA;LOC105372821;LOC107987300;CRYAA;LOC105372821
21	43335001	43336000	LINC00322
21	43631001	43632000	HSF2BP
21	43651001	43652000	HSF2BP;RRP1B
21	43710001	43711000	PDXK
21	43917001	43918000	AGPAT3;RNU6-859P
21	44058001	44059000	TRAPPC10
21	44065001	44066000	TRAPPC10
21	44262001	44263000	DNMT3L
21	44294001	44295000	AIRE;PFKL
21	44308001	44309000	AIRE;PFKL
21	44369001	44370000	TRPM2
21	44465001	44466000	LRRC3-DT;LRRC3;MTCYBP21;MTND6P21;MTND5P1
21	44523001	44524000	TSPEAR;TSPEAR-AS1;TSPEAR-AS2
21	44598001	44599000	TSPEAR;KRTAP10-6;KRTAP10-7
21	44627001	44628000	TSPEAR;KRTAP10-9;KRTAP10-10
21	44697001	44698000	TSPEAR;KRTAP10-12;KRTAP10-13P
21	44894001	44895000	ITGB2
21	44936001	44937000	ITGB2;ITGB2-AS1;LINC01547;LOC112268278;FAM207A
21	45058001	45059000	LOC105372836
21	45255001	45256000	LINC00334;POFUT2
21	45500001	45502000	COL18A1;SLC19A1;COL18A1;SLC19A1
21	45550001	45551000	SLC19A1
21	45750001	45751000	PCBP3
21	45760001	45761000	PCBP3
21	45784001	45785000	PCBP3
21	45797001	45798000	PCBP3
21	45893001	45894000	PCBP3
21	45908001	45909000	PCBP3
22	10734001	10735000	LOC105379418
22	10744001	10745000	LOC105379418
22	11615001	11616000	DUX4L43;CDC27P8;LOC100996699
22	11826001	11827000	LOC107984037;LOC107984030
22	12166001	12167000	LOC105379516;LOC107987322
22	15721001	15722000	POTEH
22	16614001	16615000	TPTEP1
22	16661001	16662000	LOC112268292;PARP4P3;ANKRD62P1-PARP4P3;ANKRD62P1
22	17452001	17453000	CECR2
22	17463001	17464000	CECR2
22	17793001	17794000	MICAL3
22	17917001	17918000	MICAL3
22	17984001	17985000	MICAL3;MIR648
22	18034001	18035000	MICAL3;LINC01634
22	18130001	18131000	TUBA8
22	18142001	18143000	USP18
22	18350001	18351000	GGTLC5P;FAM247D
22	19351001	19352000	LOC105372859;HIRA
22	19401001	19402000	HIRA
22	19936001	19937000	TXNRD2;COMT
22	19951001	19952000	TXNRD2;COMT
22	20123001	20124000	TRMT2A;MIR6816;RANBP1;SNORA77B;ZDHHC8
22	20492001	20493000	KLHL22;RN7SL812P;KRT18P5;LOC101928824
22	20610001	20611000	CCDC74BP1;SMPD4P1
22	20795001	20796000	PI4KA;SERPIND1
22	21051001	21052000	P2RX6P;LRRC74B
22	21203001	21204000	FAM247A;GGT2
22	21262001	21263000	GGT2;E2F6P3
22	21593001	21594000	UBE2L3
22	21818001	21819000	MAPK1
22	22558001	22559000	IGL;PRAME;LL22NC03-63E9.3
22	22645001	22646000	IGL;BCRP4;POM121L1P;GGTLC2;LOC129026
22	23130001	23131000	RSPH14;GNAZ;RN7SL6P

22	23176001	23177000	RSPH14;RAB36;BCRP8;BCR
22	23291001	23292000	BCR;LOC107985554
22	23534001	23535000	LOC107985580;PCAT14
22	23757001	23758000	ZNF70;VPREB3;C22orf15;CHCHD10
22	23841001	23842000	SMARCB1;DERL3
22	23912001	23913000	LOC107985558
22	23954001	23955000	GSTT2B
22	24002001	24003000	GSTT4;LOC105372959;CABIN1
22	24287001	24288000	SPECC1L-ADORA2A;SPECC1L
22	24624001	24625000	GGT1;BCRP3
22	24649001	24650000	BCRP3;POM121L10P
22	25092001	25093000	KIAA1671;KIAA1671-AS1
22	25768001	25770000	MYO18B;MYO18B
22	26017001	26018000	MYO18B
22	26159001	26160000	LOC102724801;LINC02559;LOC105372971;SEZ6L
22	26571001	26572000	TPST2;HMGB1P10
22	26616001	26617000	CRYBB1;CRYBA4
22	26747001	26748000	MIATNB
22	27312001	27313000	LINC02554;LOC105372979
22	27563001	27564000	LOC105372981
22	27761001	27762000	MN1
22	27984001	27985000	TTC28-AS1;TTC28
22	28831001	28832000	ZNRF3;LOC646408
22	28838001	28839000	ZNRF3;LOC646408;LOC107985549
22	28897001	28898000	ZNRF3
22	29421001	29422000	RFPL1;LOC102723305
22	29650001	29651000	NF2
22	29677001	29678000	NF2
22	30248001	30249000	LIF-AS1;LIF;LIF-AS2
22	30259001	30260000	LIF;OSM
22	30930001	30931000	LOC107985544;MORC2-AS1;MORC2
22	30944001	30945000	MORC2
22	32282001	32283000	SLC5A4-AS1;SLC5A4;CPSF1P1
22	32505001	32508000	FBXO7;SYN3;FBXO7;SYN3;FBXO7;SYN3
22	32533001	32534000	SYN3
22	32765001	32766000	SYN3
22	33283001	33284000	LARGE1
22	33320001	33321000	LARGE1
22	33756001	33757000	LARGE1;LARGE-AS1
22	33930001	33931000	LARGE1;LARGE1-AS1
22	35340001	35341000	TOM1;MIR3909;MIR6069
22	35411001	35412000	MCM5
22	35561001	35563000	RASD2;RASD2
22	35630001	35631000	MB;LOC284912
22	35990001	35991000	RBFOX2
22	36175001	36176000	APOL3;MTCO1P20;MTCO2P20;MTATP6P20;MTCO3P20;MTCYBP34;MTND1P10
22	36258001	36259000	APOL1;LOC112268296
22	36595001	36596000	CACNG2
22	36607001	36608000	CACNG2
22	36771001	36772000	LOC105373021;IFT27
22	36849001	36850000	NCF4-AS1
22	36864001	36865000	NCF4-AS1;NCF4
22	36951001	36952000	CSF2RBP1
22	37063001	37064000	KCTD17;RN7SKP214;TMPRSS6
22	37099001	37100000	TMPRSS6
22	37150001	37151000	IL2RB
22	37231001	37232000	RAC2
22	37273001	37274000	LOC107985576;CYTH4
22	37286001	37287000	CYTH4;LOC105373024
22	37387001	37388000	ELFN2
22	37483001	37484000	MFNG;CARD10
22	37493001	37494000	MFNG;CARD10
22	37719001	37720000	TRIOBP
22	38067001	38068000	PICK1
22	38108001	38109000	BAIAP2L2;PLA2G6
22	38161001	38163000	PLA2G6;PLA2G6
22	38248001	38249000	TMEM184B
22	38632001	38633000	FAM227A
22	38725001	38726000	GTPBP1;PRDX3P1;SUN2
22	38737001	38738000	GTPBP1;SUN2
22	38795001	38796000	DNAL4
22	38814001	38815000	NPTXR
22	38834001	38835000	NPTXR
22	38970001	38971000	APOBEC3A;LOC105373033;LOC107985562
22	39052001	39053000	APOBEC3F
22	39056001	39057000	APOBEC3F;LOC107985563
22	39067001	39068000	LOC107985563;APOBEC3G
22	39087001	39088000	APOBEC3G;LOC101927202;APOBEC3H
22	39220001	39221000	PDGFB
22	39622001	39623000	CACNA1I
22	39753001	39754000	ENTHD1;MTFR2P2
22	39803001	39804000	ENTHD1
22	40416001	40417000	SGSM3;MRTFA
22	41696001	41697000	SNU13;C22orf46;MEI1
22	41757001	41758000	MEI1
22	41821001	41822000	CCDC134;SREBF2-AS1
22	42285001	42286000	TCF20

22	42796001	42797000	DNM1P18;LOC100422416;ARFGAP3
22	42820001	42821000	ARFGAP3
22	43095001	43096000	TTLL1;RPS25P10
22	43206001	43207000	SCUBE1;SCUBE1-AS2
22	43217001	43218000	SCUBE1;SCUBE1-AS2
22	43249001	43250000	SCUBE1
22	43277001	43278000	SCUBE1;SCUBE1-AS1
22	43485001	43486000	MPPED1
22	43497001	43498000	MPPED1
22	43500001	43501000	MPPED1
22	43579001	43580000	EFCAB6;LOC101927474
22	43665001	43666000	EFCAB6
22	43690001	43691000	EFCAB6
22	43736001	43737000	EFCAB6
22	43879001	43880000	PNPLA5
22	43979001	43981000	SAMM50;SAMM50
22	44007001	44008000	PARVB;LOC107987326
22	44125001	44126000	PARVB
22	44207001	44208000	PARVG
22	44225001	44226000	LOC107985571
22	44267001	44268000	SHISAL1
22	44582001	44583000	LINC00207
22	44803001	44804000	PRR5-ARHGAP8;ARHGAP8
22	44914001	44915000	PHF21B
22	45173001	45174000	NUP50-DT;NUP50;LOC112267891;LOC105373064
22	45294001	45295000	UPK3A
22	45325001	45326000	FAM118A
22	45516001	45517000	FBLN1
22	45648001	45649000	LOC105373069;LOC105373067;LOC105373068
22	45650001	45651000	LOC105373069;LOC105373067;LOC105373068
22	45681001	45682000	ATXN10
22	45887001	45888000	LOC107985535;LOC105373071
22	46488001	46489000	CELSR1;FAM136EP
22	46659001	46660000	GRAMD4
22	46769001	46770000	TBC1D22A
22	46824001	46825000	TBC1D22A
22	46896001	46897000	TBC1D22A
22	47117001	47118000	TBC1D22A;LPEQ6126
22	47153001	47154000	TBC1D22A
22	47625001	47626000	LINC00898;LOC284930
22	48338001	48339000	LOC105373080
22	48534001	48535000	TAFA5;LOC284933
22	48857001	48858000	LINC01310
22	49501001	49502000	MIR3667HG;LOC105373087
22	49505001	49506000	MIR3667HG;LOC105373087
22	49558001	49559000	MIR3667HG
22	49582001	49583000	MIR3667HG
22	49841001	49842000	LOC105377205;LOC105373091
22	50057001	50058000	TTLL8;MLC1
22	50228001	50229000	TUBGCP6
22	50346001	50347000	PPP6R2
22	50405001	50406000	PPP6R2;MIR12114
22	50561001	50562000	KLHDC7B;SYCE3;LOC107985568;CHKB-CPT1B;CPT1B
22	50671001	50672000	SHANK3
X	432001	433000	LOC102724521
X	457001	458000	LOC102724521
X	1210001	1211000	CRLF2
X	1291001	1292000	CSF2RA;LOC112268304;MIR3690;RNA5SP498
X	1308001	1309000	CSF2RA;RNA5SP498
X	1395001	1396000	SLC25A6;LOC105373102;LINC00106;ASMTL-AS1;ASMTL
X	1460001	1461000	ASMTL;P2RY8
X	1518001	1519000	P2RY8
X	1604001	1605000	AKAP17A;ASMT
X	1877001	1878000	LOC107985677
X	2276001	2277000	DHRSX
X	2283001	2284000	DHRSX
X	2294001	2295000	DHRSX
X	2394001	2395000	DHRSX
X	2501001	2502000	DHRSX;ZBED1
X	2560001	2561000	LOC101928092
X	2643001	2644000	CD99P1
X	2749001	2750000	CD99;XG
X	6833001	6834000	PUDP
X	17770001	17772000	FAM136GP;FAM136GP
X	37458001	37459000	PRRG1
X	46656001	46657000	SLC9A7;PGAM1P7
X	48788001	48789000	GATA1
X	48973001	48974000	KCND1;GRIPAP1
X	56771001	56772000	NBDY
X	72879001	72880000	DMRTC1
X	96626001	96628000	SKP2P1;SKP2P1
X	106467001	106468000	LOC105373306;NAP1L4P2
X	111105001	111106000	PAK3
X	114601001	114602000	HTR2C
X	114606001	114607000	HTR2C
X	116817001	116818000	LOC100126447
X	119322001	119323000	LOC101928336

X	130278001	130279000	ZNF280C
X	131712001	131713000	FIRRE
X	143502001	143503000	SPANXN3
X	150516001	150517000	MAMLD1
X	153860001	153861000	L1CAM
X	155887001	155888000	VAMP7
Y	5326001	5327000	PCDH11Y
Y	11309001	11311000	DUX4L16;DUX4L17;DUX4L16;DUX4L17
Y	11312001	11314000	DUX4L16;DUX4L17;DUX4L18;DUX4L16;DUX4L17;DUX4L18
Y	11318001	11319000	DUX4L16;DUX4L17;DUX4L18
Y	11326001	11327000	DUX4L17;DUX4L18;DUX4L19;PABPC1P5
Y	11328001	11329000	DUX4L18;DUX4L19;PABPC1P5
Y	13135001	13136000	LOC112268312
Y	56848001	56849000	CTBP2P1
Y	56851001	56852000	CTBP2P1
Y	56861001	56862000	CTBP2P1

Supplemental Table 10
Female WGCNA Yellow Module

Chr	Start	Stop	Annotation	Category
1	137001	138000	LOC100996442;SEPTIN14P18;CICP27;LOC729737	
1	790001	791000	LINC01409	
1	909001	910000	LOC284600;LINC02593	
1	982001	983000	PLEKHN1;PERM1	
1	1009001	1010000	HES4;RPL39P12;ISG15	Transcription
1	1127001	1129000	LINC01342;LINC01342	;
1	1140001	1141000	LINC01342	
1	1219001	1220000	TNFRSF4;SDF4	Receptor;Signaling
1	1233001	1234000	SDF4;B3GALT6;C1QTNF12	Signaling;Golgi;Hormone
1	1288001	1289000	LINC01786;SCNN1D;ACAP3;MIR6726	Transport
1	1337001	1338000	CPTP;TAS1R3;DVL1;MIR6808	Transport;Signaling;Cytoskeleton
1	1340001	1341000	TAS1R3;DVL1;MIR6808	Signaling;Cytoskeleton
1	1410001	1411000	MRPL20-AS1;MRPL20;RN7SL657P;MRPL20-DT;LOC107984868;ANKRD65;LOC105378585	Translation
1	1483001	1484000	ATAD3B	
1	1504001	1505000	ATAD3B;ATAD3A	
1	1592001	1593000	FNDC10;LOC105378586	
1	1605001	1607000	FNDC10;LOC105378586;MIB2;FNDC10;LOC105378586;MIB2	Proteolysis;Proteolysis
1	1896001	1897000	GNB1;LOC105378949	Signaling
1	1937001	1939000	CFAP74;CFAP74	;
1	2001001	2002000	CFAP74	
1	2014001	2015000	LOC105378589;GABRD	Ion Channel
1	2038001	2039000	GABRD;LOC105378590	Ion Channel
1	2400001	2401000	MORN1;RER1;PEX10	Signaling;Transcription
1	2654001	2655000	TTC34	
1	2661001	2662000	TTC34	
1	2786001	2787000	TTC34;LOC105378598	
1	3141001	3142000	PRDM16	Transcription
1	3147001	3148000	PRDM16	Transcription
1	3266001	3267000	PRDM16	Transcription
1	3442001	3444000	PRDM16;PRDM16	Transcription;Transcription
1	3486001	3487000	ARHGEF16;MEGF6	Transcription;Extracellular Matrix
1	3643001	3644000	WRAP73;TP73	Transcription
1	3658001	3660000	WRAP73;TP73;TP73-AS3;WRAP73;TP73;TP73-AS3	Transcription;Transcription
1	3693001	3695000	TP73;TP73	Transcription;Transcription
1	3875001	3876000	DFFB;LOC107984910	Transcription
1	3930001	3931000	LINC01346	
1	3979001	3980000	LINC02780	
1	4007001	4009000	LINC02780;LINC02780	;
1	4733001	4735000	AJAP1;AJAP1	;
1	6063001	6064000	KCNAB2	
1	6137001	6138000	CHD5;LOC105376687	
1	6174001	6175000	CHD5	
1	6217001	6218000	LOC107985733;RNF207;ICMT	Golgi
1	6276001	6277000	ACOT7	Metabolism
1	6442001	6443000	ESPN	Cytoskeleton
1	6480001	6482000	PLEKHG5;PLEKHG5	;
1	7203001	7204000	CAMTA1	Transcription
1	7215001	7216000	CAMTA1;RNU1-8P	Transcription
1	7414001	7415000	CAMTA1	Transcription
1	7419001	7420000	CAMTA1	Transcription
1	7450001	7451000	CAMTA1;CAMTA1-AS1	Transcription
1	7664001	7665000	CAMTA1	Transcription
1	7862001	7863000	UTS2	
1	7963001	7964000	PARK7	
1	8511001	8513000	RERE;RERE	;
1	8977001	8978000	CA6;RN7SL451P	
1	9095001	9096000	GPR157	Signaling
1	9238001	9239000	H6PD	Metabolism
1	9545001	9546000	SLC25A33	Transport
1	9619001	9620000	TMEM201;LOC105376712;PIK3CD	Signaling
1	9654001	9655000	PIK3CD;PIK3CD-AS1;RPL26P7	Signaling
1	9698001	9700000	PIK3CD;PIK3CD	Signaling;Signaling
1	9715001	9717000	PIK3CD;PIK3CD	Signaling;Signaling

1	9905001	9906000	CTNNBIP1	
1	10367001	10368000	KIF1B;LOC105376725	Cytoskeleton
1	10473001	10474000	DFFA;PEX14	Transport
1	10534001	10535000	PEX14	Transport
1	10571001	10572000	PEX14	Transport
1	10780001	10781000	CASZ1	Transcription
1	10892001	10893000	HSPE1P24	
1	11356001	11357000	LOC105376739	
1	11852001	11853000	CLCN6;NPPA-AS1;NPPA;NPPB	Transport
1	12257001	12258000	VPS13D	Transport
1	12440001	12441000	VPS13D	Transport
1	12916001	12917000	PRAMEF7;RNU6-1072P;PRAMEF29P	
1	13298001	13299000	PRAMEF33	
1	13590001	13591000	PDPN	
1	13644001	13646000	LOC107984919;LOC107984919	;
1	13800001	13801000	PRDM2	Transcription
1	13991001	13992000	KAZN	
1	14175001	14176000	KAZN	
1	15059001	15061000	KAZN;KAZN	;
1	15141001	15142000	TMEM51-AS1	
1	15159001	15160000	TMEM51-AS1;TMEM51;TMEM51-AS2	
1	15235001	15236000	ZBTB2P1;FHAD1	
1	15498001	15499000	CELA2B;CASP9	Protease;Protease
1	15578001	15579000	DNAJC16;AGMAT	Transcription;Metabolism
1	15710001	15711000	PLEKHM2	
1	15998001	15999000	SRARP	
1	16546001	16547000	TRG-CCC1-1;TRV-CAC11-1;PDE4DIPP8;LOC101056699	
1	16632001	16633000	LOC105376794;CROCCP2	Epigenetic
1	16724001	16725000	ESPNP;TRV-CAC13-1;TRG-CCC5-1	
1	17761001	17762000	ACTL8	Cytoskeleton
1	17802001	17803000	ACTL8	Cytoskeleton
1	18049001	18050000	LOC105376812	
1	19518001	19519000	LOC105376819;LOC105376818;RNU4-28P;LOC105376817	
1	19746001	19747000	TMCO4	
1	19765001	19767000	TMCO4;TMCO4	;
1	19864001	19865000	LOC105376823	
1	20021001	20023000	PLA2G5;PLA2G5	Metabolism;Metabolism
1	20151001	20152000	PLA2G2F;LOC117779438	Metabolism
1	20154001	20155000	PLA2G2F;LOC117779438;PLA2G2C	Metabolism
1	20268001	20269000	LOC105376828	
1	20690001	20691000	KIF17	Cytoskeleton
1	21336001	21337000	ECE1	Protease
1	21807001	21808000	LDLRAD2	
1	21888001	21889000	HSPG2	Extracellular Matrix
1	21903001	21904000	HSPG2;RPL21P29	Extracellular Matrix
1	22155001	22156000	LOC105376845;LOC105376850	
1	22270001	22271000	MIR4418	
1	22417001	22418000	LOC105376856	
1	22667001	22668000	C1QB	
1	22758001	22759000	EPHB2	Receptor
1	23152001	23153000	LUZP1;RNU6-514P	
1	23852001	23853000	FUCA1	Metabolism
1	24353001	24354000	GRHL3;STPG1	Transcription;Development
1	24444001	24445000	NIPAL3	
1	24911001	24912000	RUNX3;MIR6731	Transcription
1	25827001	25828000	SELENON;MTFR1L;LOC646471;AUNIP	
1	26069001	26070000	TRIM63	Proteolysis
1	26337001	26338000	CRYBG2;RPL34P4	
1	26449001	26450000	DHDDS;RPL17P9	Metabolism
1	26818001	26819000	RN7SL165P;ZDHHC18	
1	27091001	27092000	SLC9A1	Transport
1	27100001	27102000	SLC9A1;SLC9A1	Transport;Transport
1	27385001	27386000	CD164L2;GPR3	Signaling
1	27396001	27397000	GPR3;WASF2	Signaling;Cytoskeleton
1	27895001	27896000	THEMIS2;RPA2	
1	28344001	28345000	MED18	Transcription

1	28400001	28401000	PHACTR4	Signaling
1	28707001	28708000	GMEB1	
1	29279001	29280000	PTPRU	Signaling
1	29618001	29619000	LOC107984934	
1	30747001	30748000	LAPTM5;MIR4420;LOC105378620	Transport
1	30766001	30768000	LAPTM5;LOC105378621;LAPTM5;LOC105378621	Transport;Transport
1	30843001	30844000	LINC01778;RN7SKP91	
1	31557001	31558000	LOC105378626	
1	31708001	31710000	COL16A1;COL16A1	Extracellular Matrix;Extracellular Matrix
1	31756001	31758000	ADGRB2;MIR4254;ADGRB2;MIR4254	Signaling;Signaling
1	32068001	32069000	KHDRBS1;TMEM39B	Translation
1	32183001	32184000	KPNA6;TXLNA	Transport;Transport
1	32187001	32188000	TXLNA	Transport
1	32386001	32387000	BSDC1	
1	32589001	32590000	ZBTB8A	Cytoskeleton
1	32906001	32907000	TMEM54;LOC105378632	
1	33180001	33181000	TRIM62	Proteolysis
1	33269001	33270000	ZNF362	Transcription
1	33508001	33510000	ZSCAN20;LOC100422287;CSMD2;ZSCAN20;LOC100422287;CSMD2	Transcription;Transcription
1	34130001	34131000	CSMD2	
1	34496001	34498000	LOC105378641;LOC105378641	;
1	34571001	34572000	LOC105378641	
1	34609001	34610000	LOC105378641	
1	35127001	35128000	EFCAB14P1	
1	35962001	35963000	AGO3	Translation
1	36391001	36392000	STK40;LSM10	Signaling
1	36407001	36408000	LSM10;RNU4-27P;OSCP1	
1	36881001	36882000	GRIK3	Receptor
1	36893001	36894000	GRIK3	Receptor
1	37005001	37006000	GRIK3	Receptor
1	37621001	37622000	RSPO1	
1	38425001	38426000	LOC105378657;LOC107984943	
1	38699001	38700000	LOC105378660;HSPA5P1	
1	39264001	39265000	MACF1	Cytoskeleton
1	39523001	39524000	BMP8A;OXCT2P1;PPIEL	Growth Factors
1	39728001	39729000	LOC100128091;RNU7-121P;PPIE	Transcription
1	39861001	39862000	TRIT1	Translation
1	40217001	40218000	RLF	Transcription
1	40654001	40655000	RIMS3;LOC105378675	Transport
1	41016001	41018000	CTPS1;SLFNL1-AS1;SLFNL1;CTPS1;SLFNL1-AS1;SLFNL1;SCMH1	Metabolism;Metabolism;Epigenetic
1	41422001	41423000	LOC105378678	
1	41526001	41528000	HIVEP3;HIVEP3	;
1	41544001	41545000	HIVEP3;LOC100418723	
1	41675001	41676000	HIVEP3	
1	41815001	41816000	HIVEP3	
1	41845001	41846000	HIVEP3	
1	41940001	41941000	HIVEP3	
1	42015001	42016000	HIVEP3	
1	42276001	42277000	FOXJ3	
1	42750001	42751000	CLDN19;P3H1	Cell Junction;Extracellular Matrix
1	42864001	42865000	LOC339539	
1	44744001	44745000	KIF2C	Cytoskeleton
1	46492001	46493000	DMBX1	Development
1	47819001	47820000	TRABD2B	Protease
1	48230001	48231000	SLC5A9	Transport
1	48237001	48238000	SLC5A9	Transport
1	49479001	49480000	AGBL4;AGBL4-IT1	Protease
1	49552001	49553000	AGBL4	Protease
1	49749001	49750000	AGBL4	Protease
1	50843001	50844000	FAF1;MRPS6P2	
1	51219001	51220000	LINC01562	
1	51730001	51731000	OSBPL9	
1	52347001	52348000	ZFYVE9;CC2D1B	
1	52471001	52472000	TUT4	Metabolism
1	53096001	53097000	SLC1A7	Transport
1	53099001	53100000	SLC1A7	Transport

1	53533001	53534000	GLIS1	Transcription
1	53588001	53589000	GLIS1	Transcription
1	53691001	53692000	GLIS1;RNU7-95P	Transcription
1	53790001	53791000	NDC1	
1	54345001	54347000	SSBP3;SSBP3	Transcription;Transcription
1	54999001	55000000	TMEM61;BSND	
1	55400001	55401000	RNU6-830P	
1	59636001	59637000	FGGY	Metabolism
1	59650001	59651000	FGGY	Metabolism
1	60264001	60265000	LINC02778;LOC105378761;LOC107984962	
1	60943001	60944000	NFIA-AS2	
1	64245001	64246000	UBE2U	
1	64527001	64528000	CACHD1	Transport
1	65236001	65237000	AK4	Signaling
1	65335001	65336000	DNAJC6	Transport
1	66784001	66785000	DYNLT5	
1	66819001	66820000	DNAI4	
1	67902001	67903000	GNG12-AS1	
1	67988001	67989000	GNG12-AS1	
1	70295001	70296000	ANKRD13C	
1	74747001	74748000	TYW3	Epigenetic
1	75149001	75150000	LHX8	Development
1	75700001	75701000	SLC44A5	Transport
1	75861001	75862000	MSH4	Transcription
1	76156001	76157000	ST6GALNAC3	
1	76897001	76899000	ST6GALNAC5;ST6GALNAC5	;
1	77310001	77311000	AK5	Signaling
1	77532001	77533000	AK5	Signaling
1	77544001	77545000	AK5	Signaling
1	83686001	83687000	LINC01725	
1	83849001	83850000	LINC01725;LOC101927560	
1	84151001	84152000	PRKACB;LOC107985046	Signaling
1	84219001	84220000	PRKACB;LOC107985046	Signaling
1	84483001	84484000	RPF1	Translation
1	85475001	85476000	DDAH1;LOC107985054	Metabolism
1	86670001	86671000	CLCA4-AS1	
1	86826001	86827000	LOC105378831	
1	87231001	87232000	LINC02801	
1	89138001	89139000	LOC105378842;GBP7	Signaling
1	89845001	89846000	LRRC8D	Cytoskeleton
1	89977001	89978000	LOC105378847;RN7SKP272	
1	90236001	90237000	LOC105378849	
1	91387001	91388000	HFM1	Transcription
1	91961001	91962000	BRDT	
1	93542001	93543000	FNBP1L	
1	94979001	94980000	ALG14	Transport
1	95134001	95135000	TLCD4;TLCD4-RWDD3	
1	95170001	95171000	TLCD4;TLCD4-RWDD3;LOC101928118	
1	96476001	96477000	LOC105378866	
1	96806001	96807000	PTBP2	
1	99042001	99043000	LOC100129620	
1	100357001	100358000	CDC14A	Signaling
1	103716001	103717000	AMYP1	
1	106081001	106082000	LOC105378885;LOC126987	
1	107712001	107713000	VAV3	
1	108159001	108160000	SLC25A24	Transport
1	108583001	108584000	FAM102B	
1	110312001	110314000	RBM15-AS1;RBM15-AS1	;
1	111159001	111160000	CEPT1	Transport
1	111613001	111614000	RAP1A;LINC01160;LOC107985184	Signaling
1	111871001	111873000	KCND3;KCND3	Transport;Transport
1	111895001	111896000	KCND3	Transport
1	113020001	113021000	LRIG2-DT	
1	113530001	113531000	MAGI3	
1	114112001	114113000	SYT6	Transport
1	114597001	114598000	DENND2C	

1	114933001	114934000	SYCP1	
1	116140001	116141000	MAB21L3	
1	117028001	117029000	CD101;LOC101929099	Immune
1	117175001	117176000	VTCN1	Immune
1	117648001	117649000	VDAC2P3	
1	120102001	120103000	LOC105378939	
1	121007001	121009000	PDE4DIPP4;TRF-GAA10-1;TRG-CCC6-1;PDE4DIPP4;TRF-GAA10-1;TRG-CCC6-1	;
1	121422001	121423000	LINC02798	
1	144152001	144154000	LOC105371207;LOC105371206;LOC105371207;LOC105371206	;
1	144166001	144167000	LOC105371206	
1	144755001	144756000	LOC100996737	
1	144814001	144815000	LOC105371216;LOC105371217	
1	144869001	144870000	LOC102724269;LOC107985528	
1	146056001	146057000	LOC107985594;RNVU1-6;LINC01719;NBPF10	
1	149060001	149061000	PDE4DIP;NBPF9	
1	149166001	149167000	RNVU1-24;LOC730257	
1	150304001	150305000	MRPS21	Translation
1	150498001	150499000	TARS2;MIR6878;ECM1	Translation
1	150594001	150595000	LOC107985203	
1	150635001	150636000	ENSA;LOC112268239	
1	150916001	150917000	CTXND2;LOC107985204;SETDB1	Epigenetic
1	151343001	151344000	RFX5;RFX5-AS1	Transcription
1	151428001	151429000	POGZ	Transcription
1	151898001	151899000	THEM4	Metabolism
1	153317001	153318000	PGLYRP3;PGLYRP4	
1	153380001	153381000	S100A12;LAPTM4BP1;S100A8	Signaling
1	153689001	153690000	NPR1;MIR8083;GEMIN2P1	Signaling
1	153846001	153847000	GATAD2B;LOC645965	Transcription
1	153874001	153875000	GATAD2B	Transcription
1	153938001	153939000	LOC101928059;DENND4B;CRTC2	Transcription
1	153951001	153952000	DENND4B;CRTC2;SLC39A1	Transcription;Transport
1	154287001	154288000	RNU6-239P;RNU6-121P	
1	154788001	154789000	KCNN3	Transport
1	154986001	154987000	CKS1B;FLAD1;LENEP	Cytoskeleton;Transport
1	155297001	155298000	HCN3;PKLR	Transport;Signaling
1	155771001	155772000	GON4L	Transcription
1	156004001	156005000	ARHGEF2-AS1;LOC107985208;SSR2	Transport
1	156046001	156047000	UBQLN4;LAMTOR2	
1	156072001	156073000	RAB25;MEX3A;LMNA	Metabolism
1	156757001	156758000	HDGF;PRCC	Transcription
1	156888001	156889000	NTRK1;PEAR1	Receptor;Signaling
1	157994001	157995000	LOC105371459;KIRREL1	
1	158267001	158268000	CD1A;HMGN1P5	Immune
1	158337001	158338000	CD1B	Immune
1	160994001	160995000	F11R	
1	161194001	161195000	B4GALT3;ADAMTS4;NDUFS2	Golgi;Protease;Metabolism
1	161422001	161424000	LOC102724602;TRUND-NNN3-1;TRE-TTC4-2;TRN-GTT2-2;LOC102724602;TRUND-NNN3-1;TRE-TTC4-2;TRN-GTT2-2	;
1	161458001	161459000	TRD-GTC2-2;TRL-CAG1-2;TRG-GCC1-2;TRE-CTC1-3;TRG-TCC2-3;TRD-GTC2-3;TRL-CAG1-3;TRG-GCC1-3;TRE-CTC1-4;TRG-TCC2-4;TRD-GTC2-4;TRL-CAG1-4;TRG-GCC1-4	
1	161464001	161465000	TRE-CTC1-3;TRG-TCC2-3;TRD-GTC2-3;TRL-CAG1-3;TRG-GCC1-3;TRE-CTC1-4;TRG-TCC2-4;TRD-GTC2-4;TRL-CAG1-4;TRG-GCC1-4;TRE-CTC1-5;TRG-TCC2-5;TRD-GTC2-5;TRL-CAG1-5	
1	161466001	161467000	TRL-CAG1-3;TRG-GCC1-3;TRE-CTC1-4;TRG-TCC2-4;TRD-GTC2-4;TRL-CAG1-4;TRG-GCC1-4;TRE-CTC1-5;TRG-TCC2-5;TRD-GTC2-5;TRL-CAG1-5	
1	161468001	161469000	TRG-GCC1-3;TRE-CTC1-4;TRG-TCC2-4;TRD-GTC2-4;TRL-CAG1-4;TRG-GCC1-4;TRE-CTC1-5;TRG-TCC2-5;TRD-GTC2-5;TRL-CAG1-5	
1	162198001	162199000	NOS1AP	Cytoskeleton
1	164575001	164577000	PBX1;PBX1	Development;Development
1	164773001	164774000	PBX1;PBX1-AS1	Development
1	165860001	165861000	UCK2	Signaling
1	166226001	166227000	LOC112268276	
1	166334001	166335000	LOC112268276	
1	168432001	168433000	LOC100505918	
1	168638001	168639000	LOC105371604	
1	169404001	169405000	BLZF1;CCDC181	Transcription

1	169541001	169542000	F5	Metabolism
1	170666001	170667000	LOC105371610;PRRX1	
1	171677001	171678000	PFN1P1;RPL4P3	
1	171691001	171692000	RPL4P3;VAMP4	
1	172078001	172079000	DNM3	Transport
1	173986001	173987000	RC3H1	
1	173994001	173995000	RC3H1	
1	174203001	174204000	RABGAP1L	Signaling
1	175382001	175383000	TNR	Signaling
1	175578001	175579000	TNR	Signaling
1	176019001	176020000	COP1	Proteolysis
1	177988001	177990000	CRYZL2P-SEC16B;SEC16B;CRYZL2P-SEC16B;SEC16B	;
1	179078001	179079000	FAM20B;TOR3A	Transcription
1	179638001	179639000	TDRD5	Cytoskeleton
1	179798001	179799000	FAM163A;MIR12116	
1	180153001	180154000	LOC105371636;QSOX1	Metabolism
1	180196001	180197000	QSOX1	Metabolism
1	180307001	180308000	ACBD6	
1	180680001	180681000	XPR1	Transport
1	181093001	181094000	IER5	
1	181530001	181531000	CACNA1E	Transport
1	182010001	182011000	LOC105371640	
1	182337001	182338000	RNU6-152P;RPL18P2;EIF1P3	
1	183197001	183198000	LAMC2	Extracellular Matrix
1	183302001	183303000	NMNAT2	Metabolism
1	183455001	183456000	LOC105371646;SMG7-AS1	
1	183489001	183490000	SMG7	Metabolism
1	183870001	183871000	RGL1	Transcription
1	185227001	185228000	SWT1;RPL5P5	
1	186314001	186315000	PRG4;TPR;RNU6-1240P	Transport
1	189226001	189227000	LOC105371657	
1	189491001	189492000	LOC105371657	
1	194358001	194359000	LOC107985242	
1	196881001	196882000	LOC100996886;CFHR4	
1	198314001	198315000	NEK7	Signaling
1	200051001	200052000	NR5A2;RNU6-570P	Transcription
1	201190001	201191000	IGFN1	
1	201413001	201414000	TNNI1	Cytoskeleton
1	201673001	201674000	NAV1	
1	201794001	201795000	NAV1;IPO9-AS1	
1	202134001	202135000	GPR37L1;LOC105371683;ARL8A	Signaling
1	202204001	202205000	LGR6	
1	202245001	202246000	LGR6;LOC101929388	
1	202377001	202378000	PPP1R12B;CYCSP4	Signaling
1	202625001	202626000	SYT2	Transport
1	203660001	203661000	ATP2B4;NSA2P1	Transport
1	204212001	204213000	GOLT1A;LOC100420418;PLEKHA6	Metabolism
1	204269001	204270000	PLEKHA6	
1	204375001	204376000	PLEKHA6;LINC00628	
1	204458001	204459000	PIK3C2B	Signaling
1	204474001	204475000	PIK3C2B	Signaling
1	205066001	205067000	CNTN2	Cytoskeleton
1	205182001	205183000	DSTYK	Signaling
1	205333001	205334000	KLHDC8A	
1	205784001	205785000	RAB29;LOC105371702;SLC41A1	Transport
1	205973001	205974000	SLC26A9-AS1;RAB7B	
1	205990001	205991000	RAB7B	
1	206648001	206649000	DYRK3	
1	206925001	206926000	FCMR;PIGR	Immune
1	206937001	206938000	PIGR	Immune
1	207645001	207646000	CR1;CD46P1;CR1L	
1	208084001	208085000	PLXNA2;LOC105372887	
1	208713001	208714000	LOC105372892	
1	209536001	209537000	LOC105372898	
1	210023001	210024000	SYT14	
1	211183001	211184000	RPS25P2	

1	212390001	212391000	PACC1	
1	212830001	212831000	RPS5P4;SPATA45	
1	213165001	213166000	RPS6KC1	Signaling
1	213787001	213788000	LOC105372912	
1	213808001	213809000	LOC105372912	
1	215226001	215227000	KCNK2	Transport
1	216394001	216395000	USH2A	Extracellular Matrix
1	217642001	217643000	SPATA17	
1	219850001	219851000	LOC105372926	
1	220611001	220612000	MARK1	Signaling
1	220704001	220705000	C1orf115	
1	221545001	221546000	LOC107985462;LOC100132179;LOC105372937	
1	222479001	222480000	CICP13;LOC728417;LOC105372956	
1	222553001	222554000	HHIPL2;TAF1A	Signaling
1	223252001	223253000	SUSD4	
1	224014001	224016000	SEPTIN7P13;SEPTIN7P13	;
1	224214001	224215000	LOC101927143;LOC101927164	
1	224319001	224320000	NVL	
1	224407001	224408000	WDR26;MIR4742	
1	225397001	225398000	DNAH14;LBR	Cytoskeleton;Metabolism
1	225950001	225951000	LEFTY2	Growth Factors
1	226506001	226507000	LOC105373115	
1	227702001	227703000	ZNF847P	
1	228338001	228339000	OBSCN	
1	228608001	228610000	RNA5S1;RNA5S2;RNA5S3;RNA5S4;RNA5S1;RNA5S2;RNA5S3;RNA5S4;RNA5S5	;
1	228612001	228613000	RNA5S1;RNA5S2;RNA5S3;RNA5S4;RNA5S5;RNA5S6	
1	228615001	228616000	RNA5S1;RNA5S2;RNA5S3;RNA5S4;RNA5S5;RNA5S6;RNA5S7;RNA5S8	
1	228618001	228620000	RNA5S1;RNA5S2;RNA5S3;RNA5S4;RNA5S5;RNA5S6;RNA5S7;RNA5S8;RNA5S9;RNA5S1;RNA5S2;RNA5S3;RNA5S4;RNA5S5;RNA5S6;RNA5S7;RNA5S8;RNA5S9	;
1	228624001	228625000	RNA5S3;RNA5S4;RNA5S5;RNA5S6;RNA5S7;RNA5S8;RNA5S9;RNA5S10;RNA5S11;RNA5S12	
1	228628001	228629000	RNA5S5;RNA5S6;RNA5S7;RNA5S8;RNA5S9;RNA5S10;RNA5S11;RNA5S12;RNA5S13	
1	228631001	228632000	RNA5S6;RNA5S7;RNA5S8;RNA5S9;RNA5S10;RNA5S11;RNA5S12;RNA5S13;RNA5S14;RNA5S15	
1	228633001	228637000	RNA5S7;RNA5S8;RNA5S9;RNA5S10;RNA5S11;RNA5S12;RNA5S13;RNA5S14;RNA5S15;RNA5S16;RNA5S8;RNA5S9;RNA5S10;RNA5S11;RNA5S12;RNA5S13;RNA5S14;RNA5S15;RNA5S16;RHOU;DUSP5P1;RNA5S8;RNA5S9;RNA5S10;RNA5S11;RNA5S12;RNA5S13;RNA5S14;RNA5S15;RNA5S16;RHOU;DUSP5P1;RNA5S8;RNA5S9;RNA5S10;RNA5S11;RNA5S12;RNA5S13;RNA5S14;RNA5S15;RNA5S16;RHOU;DUSP5P1;RNA5S17	;Signaling;Signaling;Signaling
1	228638001	228639000	RNA5S9;RNA5S10;RNA5S11;RNA5S12;RNA5S13;RNA5S14;RNA5S15;RNA5S16;RHOU;DUSP5P1;RNA5S17;RNA5SP18	Signaling
1	228641001	228642000	RNA5S11;RNA5S12;RNA5S13;RNA5S14;RNA5S15;RNA5S16;RHOU;DUSP5P1;RNA5S17;RNA5SP18	Signaling
1	229174001	229175000	LINC02815;LINC02814	
1	229495001	229496000	NUP133	
1	229869001	229870000	LOC105373162;LINC01682	
1	230150001	230151000	GALNT2	Golgi
1	230741001	230742000	AGT;CAPN9	Protease; Proteolysis;Protease
1	231231001	231232000	TRIM67;C1orf131;GNPAT	Proteolysis;Metabolism
1	231650001	231651000	TSNAX-DISC1;DISC1	
1	231845001	231847000	TSNAX-DISC1;DISC1;LOC105373170;TSNAX-DISC1;DISC1;LOC105373170	;
1	234957001	234958000	LOC105373211;LOC101927851	
1	235966001	235967000	LOC105373215;LINC02768;NID1	Receptor
1	236457001	236458000	EDARADD	Cytoskeleton
1	236685001	236687000	ACTN2;ACTN2	;
1	236900001	236901000	MTR;LOC105373218	
1	236984001	236985000	LOC107985368;RPL35P1	
1	237900001	237901000	LOC100130331;ZP4	
1	239436001	239437000	CHRM3	Signaling
1	239629001	239630000	CHRM3;LOC105373225	Signaling
1	239869001	239870000	CHRM3	Signaling
1	240319001	240320000	FMN2	
1	240519001	240520000	GREM2	
1	241126001	241127000	RGS7;MIR3123	
1	241435001	241436000	LOC105373230	
1	241720001	241721000	WDR64	

1	241769001	241770000	WDR64	
1	242442001	242443000	PLD5	Metabolism
1	243164001	243165000	CEP170	Cytoskeleton
1	243276001	243277000	SDCCAG8;FCF1P7	
1	243480001	243481000	SDCCAG8;AKT3	Signaling
1	243914001	243915000	LINC02774	
1	244764001	244765000	LOC107985372	
1	245179001	245180000	KIF26B	Cytoskeleton
1	245365001	245366000	KIF26B	Cytoskeleton
1	245552001	245553000	KIF26B	Cytoskeleton
1	245681001	245682000	KIF26B;LOC105373265	Cytoskeleton
1	245839001	245840000	SMYD3	Epigenetic
1	246485001	246486000	SMYD3	Epigenetic
1	246625001	246626000	CNST;LOC100887078	
1	246785001	246786000	KIF28P;LINC01341	Cytoskeleton
1	246790001	246792000	KIF28P;LINC01341;KIF28P;LINC01341	Cytoskeleton;Cytoskeleton
1	247478001	247479000	OR2W5P	
1	247565001	247566000	GCSAML;LOC102724446	
1	247892001	247894000	OR2W3;OR2W3	Receptor;Receptor
1	248657001	248658000	OR2T27	Receptor
1	248810001	248811000	SH3BP5L	Cytoskeleton
2	319001	320000	LOC105373346;LINC01865	
2	654001	656000	TMEM18;TMEM18	;
2	725001	726000	LOC105373358	
2	793001	794000	LINC01115	
2	860001	861000	LINC01115	
2	909001	911000	LINC01939;LOC105373481;LINC01939;LOC105373481	;
2	956001	957000	SNTG2-AS1;SNTG2	
2	1124001	1125000	SNTG2	
2	1233001	1234000	SNTG2	
2	1448001	1449000	TPO	Metabolism
2	1521001	1523000	TPO;TPO	Metabolism;Metabolism
2	2510001	2511000	LOC105373389	
2	3038001	3039000	LINC01250	
2	3059001	3060000	LINC01250	
2	3203001	3204000	EIPR1	
2	3218001	3219000	EIPR1	
2	3281001	3282000	EIPR1	
2	3315001	3316000	EIPR1	
2	3448001	3449000	TRAPPC12	Protease
2	3682001	3683000	ALLC;GAPDHP48	
2	6769001	6770000	LINC00487	
2	6903001	6904000	RSAD2;GRASLND	
2	7337001	7338000	LOC107985846	
2	8172001	8173000	LINC00299	
2	8596001	8597000	LOC105373411;SNRPEP5	
2	8633001	8634000	LOC105373411	
2	9144001	9145000	LOC105373417	
2	9304001	9306000	ASAP2;ASAP2	;
2	9317001	9318000	ASAP2	
2	9787001	9788000	LOC100996549	
2	10014001	10015000	LOC107985852	
2	10142001	10145000	RRM2;RRM2;RRM2	Metabolism;Metabolism;Metabolism
2	10399001	10400000	HPCAL1	
2	10639001	10640000	NOL10	
2	10687001	10688000	NOL10;RN7SL832P	
2	11429001	11430000	LOC105373429	
2	11554001	11555000	GREB1;TRG-CCC7-1;RNU2-13P;RNA5SP85	
2	11605001	11606000	GREB1	
2	11627001	11628000	GREB1	
2	12163001	12164000	MIR3681HG	
2	13671001	13672000	LOC105373438	
2	16395001	16397000	LOC107985855;LOC107985855	;
2	20229001	20231000	SDC1;DRG1P1;SDC1;DRG1P1	Receptor;Receptor
2	20434001	20435000	LOC105373465	
2	20522001	20523000	LOC102724948;NDUFAF2P1	

2	23436001	23437000	KLHL29	Cytoskeleton
2	23489001	23490000	KLHL29	Cytoskeleton
2	23559001	23560000	KLHL29	Cytoskeleton
2	23713001	23714000	KLHL29;ATAD2B	Cytoskeleton;Epigenetic
2	24811001	24812000	CENPO;ADCY3	
2	25046001	25047000	DNAJC27-AS1;EFR3B	
2	25568001	25569000	DTNB	Proteolysis
2	26118001	26119000	RAB10	
2	26483001	26484000	OTOF	Transport
2	26525001	26526000	OTOF	Transport
2	27350001	27351000	GTF3C2;LOC105374363	
2	27803001	27804000	RBKS	Metabolism
2	28272001	28273000	BABAM2	
2	29411001	29412000	ALK	Receptor
2	30227001	30228000	LBH	
2	32253001	32254000	NLRC4	
2	32916001	32917000	LINC00486;LTBP1	Extracellular Matrix
2	32989001	32990000	LTBP1	Extracellular Matrix
2	34023001	34024000	LINC01317	
2	34697001	34698000	LINC01320;LOC107985865;LOC105374458	
2	36964001	36965000	STRN	
2	37173001	37174000	SULT6B1	Transport
2	38084001	38085000	CYP1B1	Metabolism
2	38260001	38261000	LOC105374467	
2	40874001	40875000	LOC105374497	
2	41567001	41568000	LOC105374506	
2	41889001	41890000	LINC01913	
2	42106001	42107000	LOC105374531	
2	43047001	43048000	LINC01819	
2	43718001	43719000	PLEKHH2	
2	43997001	43998000	LRPPRC	
2	44252001	44253000	PPM1B	
2	44385001	44386000	CAMKMT	Golgi
2	44918001	44919000	LINC01833	
2	45221001	45222000	LINC01121;LOC105374576	
2	47587001	47588000	MSH2	Transcription
2	47777001	47778000	MSH6	Transcription
2	48506001	48508000	PPP1R21;RNU6-282P;PPP1R21;RNU6-282P	;
2	49880001	49881000	RPL7P13	
2	51593001	51594000	LOC730100	
2	53804001	53805000	GPR75-ASB3;ERLEC1	
2	56847001	56848000	LOC107985886	
2	60751001	60752000	PAPOLG	Translation
2	60808001	60809000	PAPOLG	Translation
2	60846001	60847000	LINC01185;RPL21P33	
2	60907001	60908000	REL;RNU4-51P	Transcription
2	61500001	61501000	XPO1	Transport
2	61572001	61573000	LOC100422418	
2	63038001	63039000	EHBP1;LOC100132215	
2	63081001	63082000	LOC100420499	
2	64093001	64094000	PELI1	Proteolysis
2	64471001	64472000	LGALSL	Extracellular Matrix
2	65996001	65997000	LOC105369168	
2	66160001	66161000	LOC105369168	
2	67022001	67023000	LOC105374785	
2	68246001	68247000	PPP3R1;LOC107985892	
2	69087001	69088000	ANTXR1	Cytoskeleton
2	69377001	69378000	GFPT1	Metabolism
2	69578001	69579000	AAK1	Signaling
2	70168001	70169000	C2orf42	
2	70465001	70466000	TGFA;TGFA-IT1	Growth Factors
2	70997001	70998000	TEX261;LOC105374796	
2	71250001	71251000	LOC105374797	
2	71569001	71570000	DYSF	Transport
2	71662001	71663000	DYSF	Transport
2	73011001	73012000	SFXN5;LOC107985897	Transport

2	73055001	73056000	SFXN5;LOC105374800	Transport
2	73232001	73233000	SMYD5;PRADC1;CCT7	Translation
2	74885001	74886000	HK2	Signaling
2	74920001	74921000	LINC01291;LOC105374809	
2	76905001	76906000	LRRTM4	Receptor
2	77754001	77755000	LOC101927967	
2	77820001	77821000	LOC101927967	
2	79110001	79111000	REG1A	
2	79728001	79729000	CTNNA2	Cytoskeleton
2	81411001	81412000	CHMP4AP1	
2	81608001	81609000	LOC102724542	
2	81621001	81622000	LOC102724542	
2	82853001	82854000	DHFRP3	
2	84909001	84910000	LOC105374837;LOC105374836;TMSB10;RPS2P17	
2	85244001	85245000	TCF7L1;LOC105374839	Transcription
2	85365001	85366000	ELMOD3;RN7SL113P	Cytoskeleton
2	85382001	85383000	ELMOD3	Cytoskeleton
2	85777001	85778000	ATOH8;MIR6071	Transcription
2	85817001	85818000	LOC284950;RN7SKP83	
2	87429001	87430000	LINC01943	
2	87628001	87629000	MIR4435-1	
2	87797001	87798000	RGPD2	Cytoskeleton
2	87935001	87936000	RGPD2	Cytoskeleton
2	88112001	88113000	SMYD1;FABP1	Epigenetic
2	88404001	88405000	RNU6-1007P	
2	88870001	88871000	IGK;IGKJ5;IGKJ4;IGKJ3;IGKJ2;IGKJ1	
2	89653001	89654000	IGK	
2	89796001	89797000	IGK	
2	89806001	89807000	IGK	
2	89811001	89812000	IGK	
2	89815001	89816000	IGK	
2	89818001	89819000	IGK	
2	89822001	89823000	IGK	
2	89825001	89833000	IGK;IGK;IGK;IGK;IGK;IGK;IGK;IGK	;;;;;;;
2	89835001	89837000	IGK;LOC105374862;IGK;LOC105374862	;
2	89839001	89842000	IGK;LOC105374862;IGK;LOC105374862;IGK;LOC105374862;IGKV2D-40	;;Immune
2	90293001	90294000	LOC101926946	
2	91501001	91502000	LOC101927984	
2	91503001	91505000	LOC101927984;LOC101927984	;
2	91597001	91599000	LOC388996;LOC388996	;
2	91723001	91724000	NKAIN1P2	
2	94876001	94877000	LOC442028;TEKT4	Cytoskeleton
2	95145001	95146000	ZNF514	
2	95530001	95531000	TRIM64FP;LOC100419920;LOC107985917	
2	95794001	95795000	LOC105373492;GPAT2P1	
2	96739001	96740000	LMAN2L	Transport
2	96952001	96953000	FAM178B;RNA5SP101	
2	96965001	96966000	FAM178B;RNA5SP101	
2	96985001	96986000	FAM178B	
2	96990001	96991000	FAM178B;LOC100420569	
2	97887001	97888000	TMEM131	
2	98555001	98556000	INPP4A	
2	99143001	99144000	TSGA10;C2orf15	Epigenetic
2	99558001	99559000	AFF3	Transcription
2	99650001	99651000	AFF3	Transcription
2	99891001	99892000	AFF3	Transcription
2	100007001	100008000	AFF3	Transcription
2	100022001	100023000	AFF3	Transcription
2	100226001	100227000	LINC01104	
2	100269001	100270000	LONRF2	Proteolysis
2	100674001	100675000	LINC01868	
2	101129001	101130000	TBC1D8	Signaling
2	101456001	101457000	RFX8	Transcription
2	104771001	104772000	HMGB3P11	
2	105774001	105775000	NCK2	Cytoskeleton
2	106260001	106261000	SRSF3P4	

2	106313001	106314000	RPL27AP4	
2	107463001	107464000	LINC01885	
2	108461001	108462000	GCC2	Cytoskeleton
2	108987001	108988000	EDAR	
2	109259001	109260000	SH3RF3;SNRPGP9	
2	110352001	110353000	LOC105375809;LOC442041	
2	110622001	110623000	LOC105373553	
2	110807001	110808000	ACOXL	Metabolism
2	111959001	111960000	MERTK;SLC30A6P1	Receptor
2	112411001	112412000	RGPD8	Cytoskeleton
2	113231001	113233000	PAX8;PAX8-AS1;PAX8;PAX8-AS1	;
2	113258001	113259000	PAX8;PAX8-AS1	
2	114852001	114853000	DPP10	Protease
2	115275001	115276000	DPP10	Protease
2	118963001	118964000	MARCO	Extracellular Matrix
2	119238001	119239000	STEAP3;STEAP3-AS1	
2	120333001	120334000	LOC105373989	
2	120354001	120355000	LOC105373989;INHBB	Growth Factors
2	121058001	121059000	LOC105373587	
2	121266001	121267000	TFCP2L1	Transcription
2	122135001	122136000	LOC105373592	
2	122167001	122168000	LOC105373592	
2	124556001	124557000	CNTNAP5	
2	126466001	126467000	LOC105373601	
2	126533001	126534000	LOC105373602	
2	126887001	126888000	LOC105373603	
2	127169001	127170000	NIFKP9	
2	127222001	127223000	CYP27C1	Metabolism
2	127425001	127426000	PROC;MIR4783;LOC105373608	Protease
2	127487001	127488000	IWS1	
2	127552001	127553000	MYO7B;LOC105373609	Cytoskeleton
2	129255001	129256000	LINC01854;LOC105373612	
2	130075001	130076000	POTEF	Transport
2	130278001	130279000	MTND1P29;NMTRQ-TTG7-1;MTND2P22;MTCO1P7;MTCO2P7;MTATP6P7;MTND3P15;MTND4LP15;MTND4P27;MTND5P29;MTND6P8;MTCYBP8	
2	130551001	130552000	PRSS40B	
2	130611001	130613000	POTEJ;POTEJ;RNU6-848P	Transport;Transport
2	130656001	130658000	POTEJ;POTEJ	Transport;Transport
2	131355001	131356000	LOC100420006;SSBP3P2;RAB6D	
2	131426001	131427000	GNAQP1	
2	131533001	131534000	CCDC74A;MED15P4	
2	132287001	132288000	RNA28SP	
2	132522001	132523000	GPR39;RN7SKP103	Signaling
2	132723001	132724000	NCKAP5	
2	133333001	133334000	NCKAP5;RNU6-579P	
2	135002001	135003000	MAP3K19	
2	135718001	135719000	R3HDM1	
2	138917001	138918000	LOC105373639;LOC105373640	
2	142689001	142690000	LOC107985823	
2	143244001	143245000	ARHGAP15	Signaling
2	144212001	144213000	GTDC1	Golgi
2	144584001	144585000	LINC01412	
2	146229001	146230000	LOC105373667	
2	146403001	146404000	LOC105373667	
2	148424001	148425000	MBD5	
2	151297001	151298000	NMI;LOC107985827	Transcription
2	151555001	151556000	NEB	
2	151798001	151799000	LOC101929356;ARL5A	Signaling
2	152107001	152108000	CACNB4;STAM2	Transport;Cytoskeleton
2	152566001	152567000	FMNL2	
2	153007001	153008000	LOC105373691	
2	154290001	154292000	GALNT13;PHBP4;GALNT13;PHBP4	Golgi;Golgi
2	154477001	154478000	LOC105373692;LOC105373694;LOC105373693	
2	157407001	157408000	LOC105373711;CYTIP	
2	157742001	157743000	ACVR1	Signaling

2	158057001	158058000	UPP2;PTP4A1P1	Signaling
2	159437001	159438000	BAZ2B	Epigenetic
2	160398001	160400000	RBMS1;MIR4785;RBMS1;MIR4785	;
2	162569001	162570000	KCNH7	Transport
2	165973001	165974000	LOC102724058	
2	166014001	166015000	LOC102724058;SCN1A	Transport
2	167872001	167873000	B3GALT1;B3GALT1-AS1	Golgi
2	168089001	168090000	STK39	
2	169763001	169764000	LOC171417;PTCHD3P2	
2	169952001	169953000	UBR3	Proteolysis
2	170672001	170673000	LOC100130256	
2	170717001	170719000	LOC101926913;LINC01124;SP5;LOC101926913;LINC01124;SP5	Transcription;Transcription
2	171265001	171266000	LOC107983995;LOC105373737	
2	171327001	171328000	METTL8	Epigenetic
2	171453001	171454000	DCAF17	
2	171886001	171887000	SLC25A12	Transport
2	172590001	172591000	PDK1	Signaling
2	175692001	175693000	LOC107985962	
2	175813001	175814000	LOC107985962	
2	176282001	176283000	MTX2	
2	178379001	178380000	OSBPL6	
2	178531001	178532000	TTN-AS1;TTN	
2	179010001	179011000	CCDC141;RPS6P2	
2	179700001	179701000	ZNF385B;LOC107985967	
2	181409001	181410000	LINC01934	
2	182153001	182154000	PDE1A	Signaling
2	182394001	182395000	PDE1A	Signaling
2	182899001	182900000	NCKAP1	
2	183069001	183070000	KRT8P10;DUSP19	Signaling
2	183211001	183212000	LOC105373774	
2	184672001	184673000	ZNF804A	
2	186030001	186031000	LINC01473	
2	186473001	186474000	LOC105373785	
2	187542001	187543000	LOC105373786;TFPI	Protease; Proteolysis
2	188165001	188166000	LINC01090	
2	188254001	188255000	LINC01090	
2	188555001	188556000	GULP1	Cytoskeleton
2	189756001	189757000	ANKAR;OSGEPL1;OSGEPL1-AS1;ORMDL1	Translation
2	189850001	189851000	PMS1;LOC105373796	Transcription
2	190365001	190366000	INPP1	Signaling
2	190680001	190681000	NAB1	
2	191095001	191096000	STAT4	Transcription
2	194812001	194813000	LOC105376755	
2	195946001	195947000	DNAH7	Cytoskeleton
2	196134001	196135000	STK17B	Signaling
2	196798001	196799000	GTF3C3;C2orf66	Transcription
2	197313001	197314000	ANKRD44;LOC105373824	Cytoskeleton
2	197373001	197374000	NPM1P46	
2	197382001	197383000	NPM1P46;SF3B1	Translation
2	198091001	198093000	PLCL1;PLCL1	Metabolism;Metabolism
2	198724001	198725000	LOC105373831	
2	199181001	199182000	RNU7-147P	
2	200490001	200491000	SPATS2L;KCTD18	Cytoskeleton
2	201835001	201836000	CDK15;UBE2V1P11	Signaling
2	202338001	202339000	RN7SL753P;PIMREGP1;LOC107985976	
2	202417001	202418000	BMPR2	Signaling
2	202438001	202439000	BMPR2	Signaling
2	202699001	202700000	FAM117B	
2	202931001	202932000	CARF;KRT8P52	Transcription
2	203170001	203171000	NBEAL1	
2	203263001	203264000	CYP20A1;RN7SL670P	Metabolism
2	203276001	203277000	CYP20A1	Metabolism
2	203344001	203345000	ABI2	Cytoskeleton
2	203444001	203445000	RAPH1	Cytoskeleton
2	203454001	203455000	RAPH1	Cytoskeleton
2	204946001	204947000	PARD3B	

2	205322001	205323000	PARD3B	
2	206043001	206044000	INO80D;RPL27P8	
2	207729001	207730000	CCNYL1	
2	207841001	207842000	PLEKHM3	
2	209697001	209698000	MAP2	
2	210173001	210174000	KANSL1L	
2	210620001	210621000	CPS1;CPS1-IT1	Metabolism
2	212236001	212237000	ERBB4	Receptor
2	213189001	213190000	LOC107985981	
2	213705001	213706000	SPAG16	Cytoskeleton
2	214873001	214874000	SNHG31	
2	215082001	215083000	ABCA12	Transport
2	215701001	215702000	LINC00607	
2	216485001	216486000	SMARCAL1;RPL37A-DT	Transcription
2	217068001	217070000	LOC101928278;LOC105373873;LOC101928278;LOC105373873	;
2	217133001	217134000	LOC101928278	
2	217283001	217284000	DIRC3-AS1;DIRC3	
2	217332001	217333000	DIRC3-AS1;DIRC3	
2	217975001	217976000	TNS1;TNS1-AS1	Cytoskeleton
2	218675001	218676000	RNF25;STK36	Signaling
2	219434001	219435000	DES;SPEG	
2	219478001	219479000	SPEG;ASIC4-AS1	
2	219579001	219581000	OBSL1;INHA;OBSL1;INHA;RN7SKP213	Growth Factors;Growth Factors
2	219751001	219752000	LOC105373887	
2	220484001	220485000	LOC105373893	
2	220849001	220850000	LOC107985988;LOC107985990	
2	221608001	221609000	LOC105373897	
2	222343001	222344000	CT75	
2	222893001	222894000	ACSL3;ATG12P2	Metabolism
2	224832001	224833000	DOCK10	Transcription
2	226649001	226650000	MIR5702	
2	227526001	227527000	AGFG1	
2	229354001	229355000	DNER	
2	230027001	230028000	LOC107985996;SLC16A14	Transport
2	230091001	230092000	LOC107985995	
2	230923001	230924000	GPR55	Signaling
2	231577001	231578000	RPL23AP26	
2	231655001	231656000	RN7SL499P	
2	231971001	231972000	DIS3L2	Transcription
2	232359001	232360000	ECEL1P3	
2	232395001	232396000	ECEL1P2	
2	232414001	232415000	ALPG;LOC107985999;ECEL1P1	Signaling
2	232556001	232557000	CHRNG;TIGD1;MIR5001;EIF4E2	Ion Channel;Epigenetic;Translation
2	232900001	232901000	NGEF	Transcription
2	233058001	233060000	INPP5D;INPP5D	;
2	233158001	233159000	INPP5D	
2	233479001	233480000	DGKD;USP40	Signaling;Protease
2	234975001	234976000	SH3BP4;LOC105373939	
2	235012001	235013000	SH3BP4	
2	235063001	235064000	SH3BP4	
2	235636001	235637000	AGAP1	
2	235911001	235912000	AGAP1	
2	235999001	236000000	AGAP1	
2	237517001	237518000	MLPH;MIR6811;MTND5P46;MTND4P40	Cytoskeleton
2	237537001	237538000	MLPH	Cytoskeleton
2	238038001	238039000	UBE2F-SCLY;UBE2F;RNU6-1333P	Proteolysis
2	238115001	238117000	ESPNL;ESPNL	Cytoskeleton;Cytoskeleton
2	238515001	238516000	LINC01107	
2	238853001	238854000	LOC100287387;TWIST2	Transcription
2	238902001	238903000	TWIST2	Transcription
2	238909001	238910000	TWIST2;LINC01940	Transcription
2	239283001	239284000	HDAC4	
2	239397001	239398000	HDAC4;HDAC4-AS1	
2	239775001	239776000	LOC150935	
2	239967001	239968000	NDUFA10	Signaling
2	239979001	239980000	NDUFA10	Signaling

2	240021001	240022000	NDUFA10;OR6B2	Signaling;Signaling
2	240042001	240043000	OR6B3	Signaling
2	240138001	240140000	COPS9;OTOS;COPS9;OTOS	;
2	240465001	240466000	GPC1;LOC100130449;MIR149;ANKMY1	
2	240619001	240620000	GPR35	Signaling
2	241136001	241137000	PASK	Signaling
2	241165001	241166000	PPP1R7;LOC105373971	Signaling
2	241174001	241175000	PPP1R7;LOC105373971	Signaling
2	241198001	241199000	LOC105373971;ANO7	
2	241545001	241546000	BOK-AS1;LOC105373974	
2	241644001	241645000	THAP4;ATG4B	Protease
2	241865001	241866000	PDCD1;LOC105373977;RTP5	
2	241888001	241889000	LOC105373978;LINC01237;FAM240C	
2	242003001	242004000	LINC01237;LOC285097	
2	242016001	242017000	LINC01237;LOC285097	
2	242024001	242026000	LINC01237;LINC01237	;
2	242183001	242184000	RPL23AP88	
3	10001	11000	LINC01986	
3	820001	821000	LINC01266	
3	5084001	5085000	UBTFL8	
3	6170001	6171000	LOC105376942	
3	6749001	6750000	GRM7-AS3	
3	7639001	7640000	GRM7	Signaling
3	8739001	8740000	SSUH2;CAV3	Cytoskeleton
3	9080001	9081000	SRGAP3	Signaling
3	9442001	9443000	SETD5	
3	9552001	9553000	LHFPL4	
3	9743001	9744000	BRPF1;OGG1	Transcription;Transcription
3	9769001	9770000	OGG1;CAMK1;TADA3	Transcription;Signaling;Epigenetic
3	10023001	10024000	MARK2P2;CIDECP1;FANCD2	
3	10365001	10366000	ATP2B2	Transport
3	11202001	11203000	HRH1;LOC102723663	Signaling
3	11485001	11486000	ATG7	Proteolysis
3	12260001	12261000	GSTM5P1	
3	12580001	12581000	MKRN2;RAF1	Proteolysis;Signaling
3	12724001	12725000	TMEM40	
3	12793001	12794000	KRT18P17;CAND2	Proteolysis
3	12875001	12876000	LOC105376956;LINC02022	
3	13082001	13083000	IQSEC1	Transcription
3	13090001	13091000	IQSEC1	Transcription
3	13378001	13379000	NUP210	Transport
3	13935001	13936000	VN1R20P;FGD5P1;TPRXL	
3	14471001	14472000	SLC6A6	Transport
3	14702001	14703000	C3orf20	
3	15272001	15273000	SH3BP5-AS1;SH3BP5	Cytoskeleton
3	15288001	15289000	SH3BP5	Cytoskeleton
3	15957001	15958000	LOC107986064	
3	16409001	16410000	RFTN1	
3	17559001	17560000	TBC1D5	Signaling
3	18519001	18520000	SATB1-AS1	
3	23301001	23302000	UBE2E2	Proteolysis
3	23326001	23327000	UBE2E2	Proteolysis
3	23909001	23910000	NKIRAS1;RPL15	Translation
3	23952001	23953000	NKIRAS1;NR1D2	Transcription
3	24282001	24283000	THRB	Transcription
3	25553001	25554000	RARB	Transcription
3	27655001	27656000	LOC107986071	
3	28400001	28401000	ZCWPW2	
3	28608001	28609000	LINC00693	
3	28662001	28663000	LINC00693	
3	29483001	29484000	RBMS3	
3	30430001	30431000	LOC101927995;LOC105377013	
3	31870001	31871000	OSBPL10	
3	32104001	32105000	GPD1L	Metabolism
3	32355001	32356000	CMTM8	Transport
3	33075001	33077000	GLB1;SUMO2P10;GLB1;SUMO2P10	Metabolism;Metabolism

3	33484001	33485000	RNA5SP128	
3	37166001	37167000	LRRFIP2;LOC112268444	Transcription
3	37193001	37194000	LOC112268444;RPS16P4;LOC105377642	
3	37232001	37233000	GOLGA4-AS1	
3	37525001	37526000	ITGA9	Extracellular Matrix
3	37681001	37682000	ITGA9	Extracellular Matrix
3	38064001	38065000	DLEC1	
3	38155001	38156000	LOC101928234;OXSR1	
3	38318001	38319000	SLC22A14;DLEC1P1	Transport
3	38721001	38722000	SCN10A	Transport
3	39792001	39793000	LOC105377039	
3	40247001	40248000	MYRIP;EIF1B-AS1	Cytoskeleton
3	41452001	41453000	ULK4	Signaling
3	41844001	41846000	ULK4;ULK4	Signaling;Signaling
3	41903001	41904000	ULK4	Signaling
3	42023001	42024000	LOC107986077;TRAK1	Transport
3	42536001	42537000	VIPR1;VIPR1-AS1	Receptor
3	42929001	42930000	ZNF662;KRBOX1-AS1;KRBOX1	Transcription
3	43080001	43081000	POMGNT2;LOC107986079	Golgi
3	43472001	43473000	ANO10	
3	44832001	44833000	KIF15	Cytoskeleton
3	45524001	45525000	LARS2	Translation
3	45601001	45602000	LIMD1	Transcription
3	45637001	45638000	LIMD1	Transcription
3	45901001	45902000	LZTFL1;CCR9	Transcription
3	46230001	46231000	CCR3	
3	46745001	46746000	PRSS46P;PRSS45P;PRSS43P	Protease
3	46902001	46903000	PTH1R	Receptor
3	46996001	46997000	NBEAL2	
3	47090001	47091000	SETD2	Epigenetic
3	47481001	47482000	SCAP;LOC105377073	
3	47678001	47679000	SMARCC1	Epigenetic
3	47794001	47795000	DHX30	Transcription
3	47941001	47942000	MAP4	
3	48126001	48127000	RPL17P16	
3	48272001	48273000	ZNF589	Transcription
3	48735001	48736000	PRKAR2A	Signaling
3	48877001	48878000	SLC25A20	Transport
3	48900001	48901000	SLC25A20	Transport
3	48902001	48904000	SLC25A20;SLC25A20	Transport;Transport
3	49766001	49767000	IP6K1;COX6CP14;PHF5EP	Signaling
3	49821001	49822000	UBA7;TRAIP	Proteolysis
3	49894001	49895000	MST1R;LOC102724438	Receptor
3	50198001	50199000	SEMA3F;GNAT1;SLC38A3	Signaling;Signaling;Transport
3	50208001	50209000	SLC38A3	Transport
3	50631001	50632000	MAPKAPK3	Signaling
3	51491001	51492000	DCAF1	
3	52321001	52322000	DNAH1	Cytoskeleton
3	54295001	54296000	CACNA2D3	Transport
3	54328001	54329000	CACNA2D3	Transport
3	54348001	54349000	CACNA2D3	Transport
3	54640001	54641000	CACNA2D3;ESRG	Transport
3	54709001	54710000	CACNA2D3	Transport
3	56257001	56258000	ERC2	Transport
3	56434001	56435000	ERC2;LOC105377099	Transport
3	56445001	56446000	ERC2;LOC105377099	Transport
3	57477001	57478000	DNAH12	
3	57554001	57555000	DNAH12;RNU6-483P;PDE12	Translation
3	57619001	57620000	PDE12;RNU6ATAC26P;DENND6A	Translation
3	57837001	57838000	SLMAP	Cytoskeleton
3	58252001	58253000	ABHD6	Metabolism
3	59110001	59111000	LOC105377110	
3	59333001	59334000	LOC105377110	
3	62852001	62853000	CADPS	Transport
3	63736001	63737000	LOC100130345	
3	64328001	64329000	PRICKLE2	Cytoskeleton

3	64960001	64961000	ADAMTS9-AS2;LOC105377124	
3	65538001	65539000	MAGI1	
3	65977001	65978000	MAGI1	
3	66816001	66817000	LOC105377144	
3	67835001	67836000	SUCLG2-AS1	
3	69188001	69189000	FRMD4B	
3	69741001	69742000	MITF	
3	69818001	69819000	MITF	
3	71260001	71261000	FOXP1	
3	71435001	71436000	FOXP1	
3	72110001	72111000	LINC00877	
3	72678001	72679000	LOC105377161	
3	73447001	73448000	PDZRN3	
3	75152001	75153000	LOC107986099	
3	75425001	75426000	ALG1L6P;FAM86DP;LINC02018	
3	75513001	75514000	LINC02018;SNRPCP10	
3	76143001	76144000	ROBO2	
3	76224001	76225000	ROBO2	
3	76348001	76349000	ROBO2;LOC107986020	
3	76628001	76629000	ROBO2	
3	76967001	76968000	ROBO2	
3	78769001	78770000	ROBO1;RPS12P6	
3	78806001	78807000	ROBO1	
3	81986001	81987000	LINC02008	
3	84951001	84952000	CADM2	
3	85568001	85569000	CADM2	
3	97988001	97989000	GABRR3	Ion Channel
3	98835001	98836000	DCBLD2	
3	99830001	99831000	HP09053;CMSS1;FILIP1L	Metabolism
3	99850001	99851000	CMSS1;FILIP1L	Metabolism
3	99970001	99971000	CMSS1;FILIP1L;MIR3921	Metabolism
3	100708001	100709000	TFG	Transport
3	101466001	101467000	SENP7;BTF3P16	Protease
3	106436001	106437000	LOC101929485	
3	106487001	106488000	LOC101929485	
3	106554001	106555000	LOC101929485	
3	106900001	106901000	MTND4P16;MTND4LP3;MTND3P6;MTCO3P35;MTND5P16;MTND6P6;MTND1P16;NMTRQ-TTG4-1;MTND2P14	
3	107768001	107769000	BBX	Transcription
3	109410001	109412000	H3P12;LINC01205;H3P12;LINC01205	;
3	110573001	110574000	LOC105374037	
3	111628001	111629000	CD96;NT5C3AP2	Immune
3	113472001	113473000	SPICE1	
3	113702001	113703000	USF3	
3	114884001	114885000	ZBTB20;ZBTB20-AS3	Transcription
3	116206001	116207000	LSAMP	Immune
3	116248001	116249000	LSAMP	Immune
3	118572001	118573000	LOC105374060	
3	121314001	121315000	STXBP5L	Transport
3	121501001	121502000	POLQ;RPL7AP11	Transcription
3	122102001	122103000	CD86	Immune
3	122235001	122236000	CASR	Signaling
3	122561001	122562000	LOC105374071;PARP9;DTX3L	Proteolysis
3	123566001	123567000	HACD2	
3	123577001	123578000	HACD2;MYLK-AS1	
3	124652001	124653000	KALRN	Transcription
3	125455001	125456000	SNX4	Cytoskeleton
3	125488001	125489000	SNX4	Cytoskeleton
3	126480001	126481000	ZXDC;UROC1	Transcription;Metabolism
3	126824001	126825000	CHCHD6	
3	127333001	127334000	LINC02016	
3	128413001	128414000	EEFSEC	Translation
3	128638001	128639000	RPN1	Golgi
3	128645001	128646000	RPN1	Golgi
3	128797001	128798000	RAB7A;RPS15AP16	
3	128818001	128820000	RAB7A;RAB7A	;

3	129189001	129190000	CNBP;LOC105374101	Metabolism
3	129682001	129683000	TMCC1	
3	129772001	129773000	TMCC1	
3	129894001	129895000	TMCC1;TMCC1-DT	
3	130098001	130099000	ALG1L2;LINC02014;FAM86HP	Golgi
3	130148001	130150000	LOC105374103;LOC107986131;LOC105374103;LOC107986131	;
3	133835001	133836000	SRPRB;RAB6B	Transport
3	134889001	134890000	EPHB1	Receptor
3	135470001	135471000	LOC105374122	
3	138352001	138353000	MRAS	Signaling
3	138437001	138438000	ESYT3	
3	139441001	139442000	COPB2-DT	
3	139634001	139635000	NMNAT3	Metabolism
3	140403001	140404000	CLSTN2	Transport
3	140547001	140548000	CLSTN2	Transport
3	141804001	141806000	GRK7;GRK7	Signaling;Signaling
3	141941001	141942000	TFDP2	Transcription
3	143666001	143667000	SLC9A9;LOC102724120	Transport
3	145680001	145681000	GM2AP1	
3	148714001	148715000	AGTR1	Signaling
3	148753001	148754000	AGTR1	Signaling
3	150120001	150121000	LOC105374313	
3	153388001	153389000	LINC02006	
3	154058001	154059000	ARHGEF26-AS1	
3	154940001	154941000	LOC105374171	
3	155074001	155075000	MME	Protease
3	155659001	155660000	PLCH1;RPL6P7	Metabolism
3	156035001	156036000	VN2R1P;ALG1L15P	
3	156809001	156810000	LINC00886;PA2G4P4	
3	157022001	157023000	LEKR1;RN7SKP177	
3	157217001	157218000	LOC101928236	
3	159714001	159715000	IQCJ-SCHIP1;SCHIP1;LOC100420738	
3	159926001	159927000	IL12A-AS1	
3	160150001	160151000	IL12A-AS1	
3	160193001	160194000	IL12A-AS1	
3	160350001	160351000	TRIM59-IFT80;IFT80;RPL35AP10	
3	160865001	160866000	PPM1L	Signaling
3	162191001	162192000	LOC107986048	
3	164482001	164483000	LOC105374191	
3	167075001	167076000	PPIAP74;LOC105374196	
3	169188001	169189000	MECOM	Transcription
3	171968001	171969000	LOC105374217	
3	172164001	172165000	FNDC3B	Proteolysis
3	173893001	173894000	NLGN1	Cytoskeleton
3	174469001	174470000	NAALADL2	Protease
3	174934001	174935000	NAALADL2	Protease
3	175173001	175174000	NAALADL2	Protease
3	176063001	176064000	EI24P1	
3	177290001	177291000	LINC00501;ASS1P7	
3	177324001	177325000	LINC00501	
3	177748001	177749000	LINC00578	
3	178556001	178557000	KCNMB2-AS1;KCNMB2	Transport
3	179423001	179424000	GNB4	Signaling
3	179521001	179523000	LOC107986157;LOC107986157	;
3	179650001	179651000	H3P13;USP13	Protease
3	182992001	182993000	DCUN1D1;LOC105374246	
3	183749001	183750000	YEATS2	Transcription
3	184219001	184220000	VWA5B2	
3	184247001	184248000	VWA5B2;MIR1224;ALG3;EEF1AKMT4-ECE2;EEF1AKMT4	Golgi;Protease;Epigenetic
3	184304001	184305000	PSMD2;EIF4G1	Protease;Translation
3	184370001	184371000	CLCN2;POLR2H;THPO;CHRD	Transport;Transcription
3	184902001	184903000	VPS8	Cytoskeleton
3	185374001	185375000	MAP3K13	Signaling
3	185395001	185396000	MAP3K13	Signaling
3	185476001	185477000	MAP3K13;LOC101929018;TMEM41A	Signaling
3	186282001	186283000	DGKG	Signaling

3	186306001	186307000	DGKG	Signaling
3	186347001	186348000	DGKG	Signaling
3	186968001	186969000	ST6GAL1	Transport
3	189466001	189467000	LOC105374270	
3	190565001	190566000	IL1RAP	Receptor
3	191147001	191148000	LOC107986171	
3	193456001	193457000	ATP13A4	
3	193566001	193567000	ATP13A4	
3	194025001	194026000	LINC02028	
3	194609001	194610000	TMEM44;LOC105374291	
3	194862001	194863000	LOC107986173	
3	195628001	195629000	MUC20P1;LOC107986029;LOC105374298	
3	195785001	195786000	MUC4	
3	196273001	196274000	PCYT1A	Transport
3	196467001	196468000	RNF168	
3	196761001	196762000	PAK2	Signaling
3	197015001	197016000	MELTF	Transport
3	197051001	197052000	DLG1	Cytoskeleton
3	197331001	197333000	LOC101926923;LOC101926923	;
3	197430001	197431000	LOC105374308	
3	197459001	197461000	LOC105374308;LOC105374308	;
3	197719001	197721000	RUBCN;RUBCN	;
3	198168001	198170000	FAM157A;FAM157A	;
4	109001	110000	LOC100129037	
4	447001	448000	ABCA11P;ZNF721	Transcription
4	467001	468000	ABCA11P;ZNF721;LOC100533735	Transcription
4	585001	586000	TMEM271;LOC105374338;PDE6B	Signaling
4	665001	666000	PDE6B;PDE6B-AS1;ATP5ME;MYL5	Signaling;Metabolism;Cytoskeleton
4	752001	753000	PCGF3;LOC100129917	Epigenetic
4	1026001	1027000	FGFRL1;LOC105374343	Receptor
4	1070001	1071000	RNF212	
4	1299001	1300000	LOC105374347;MAEA	
4	1315001	1316000	MAEA	
4	1349001	1350000	MAEA;UVSSA	
4	1354001	1356000	UVSSA;UVSSA	;
4	1586001	1587000	FAM53A	
4	1650001	1651000	FAM53A	
4	1888001	1889000	NSD2	
4	1905001	1906000	NSD2	
4	1985001	1987000	NSD2;NELFA;MIR943;NSD2;NELFA;MIR943	;
4	2297001	2298000	ZFYVE28	
4	2350001	2351000	ZFYVE28;LOC105374352	
4	2483001	2484000	RNF4	
4	2967001	2969000	NOP14;GRK4;NOP14;GRK4	Metabolism;Signaling;Metabolism;Signaling
4	3179001	3180000	HTT	
4	3186001	3187000	HTT	
4	3294001	3295000	RGS12	
4	3309001	3311000	RGS12;LOC100286945;RGS12;LOC100286945	;
4	3433001	3434000	RGS12;HGFAC	Protease
4	4246001	4247000	TMEM128	
4	4382001	4383000	LOC112268462;NSG1	
4	4664001	4665000	STX18-AS1;SNORD162	
4	4674001	4675000	STX18-AS1;SNORD162	
4	6342001	6343000	PPP2R2C	Signaling
4	6511001	6512000	PPP2R2C	Signaling
4	6599001	6600000	MAN2B2	
4	6667001	6668000	LINC02482;LOC93622	
4	7226001	7227000	SORCS2	Transport
4	7526001	7527000	SORCS2	Transport
4	7578001	7579000	SORCS2	Transport
4	7602001	7603000	SORCS2	Transport
4	7885001	7886000	AFAP1	
4	8200001	8201000	SH3TC1	
4	8265001	8266000	LOC105374373;HTRA3	Protease
4	8268001	8269000	LOC105374373;HTRA3	Protease
4	8478001	8479000	TRMT44	

4	8842001	8843000	HMX1;LOC105374474	Development
4	8878001	8879000	HMX1	Development
4	9247001	9249000	USP17L16P;USP17L17;USP17L18;USP17L19;USP17L16P;USP17L17;USP17L18;USP17L19;USP17L20	Protease;Protease
4	9252001	9253000	USP17L17;USP17L18;USP17L19;USP17L20;USP17L21	Protease
4	9270001	9271000	USP17L21;USP17L22	Protease
4	9336001	9337000	USP17L24;USP17L25;USP17L26;USP17L5;USP17L27	Protease
4	9340001	9342000	USP17L25;USP17L26;USP17L5;USP17L27;USP17L28;USP17L25;USP17L26;USP17L5;USP17L27;USP17L28	Protease;Protease
4	9524001	9525000	LOC101928948	
4	9866001	9867000	SLC2A9	
4	10435001	10436000	ZNF518B	Transcription
4	10480001	10481000	LOC100130072;CLNK;LOC105374482	Cytoskeleton
4	12900001	12901000	LOC105374493	
4	15279001	15281000	C1QTNF7-AS1;C1QTNF7-AS1	;
4	15484001	15485000	CC2D2A	
4	16393001	16394000	LOC105374505	
4	16554001	16555000	LOC105374505;LDB2	Transcription
4	17061001	17062000	MTND5P4	
4	17608001	17609000	LAP3;MED28	Protease;Transcription
4	17659001	17660000	FAM184B	
4	18217001	18218000	LOC107986262	
4	18224001	18225000	LOC107986262	
4	19889001	19890000	LOC105374511	
4	22418001	22419000	ADGRA3	Signaling
4	22485001	22486000	ADGRA3	Signaling
4	23295001	23297000	LOC105374524;LOC105374523;LOC105374524;LOC105374523	;
4	23711001	23712000	LOC105374528	
4	24263001	24264000	PPARGC1A	Transcription
4	24385001	24386000	PPARGC1A	Transcription
4	25112001	25113000	LOC105374534;SEPSECS	
4	25243001	25245000	LOC105374535;PI4K2B;PI4K2B	Signaling;Signaling
4	25332001	25333000	ZCCHC4	Epigenetic
4	25514001	25515000	LOC645433	
4	26249001	26250000	RBPJ	Transcription
4	26270001	26271000	RBPJ	Transcription
4	26605001	26606000	TBC1D19	
4	26694001	26695000	TBC1D19	
4	27241001	27242000	LINC02261	
4	28256001	28257000	LOC105374557	
4	29805001	29806000	LOC105374562	
4	31537001	31538000	LINC02501	
4	31990001	31991000	LOC107986222;LINC02506	
4	32159001	32160000	LINC02506	
4	32246001	32247000	LOC102723846	
4	33947001	33948000	LOC101928622	
4	36999001	37000000	LINC02616	
4	38477001	38478000	LINC01258	
4	38734001	38735000	LOC105374413	
4	38753001	38754000	LOC105374413;LOC105374412;RNA5SP158	
4	39306001	39307000	RFC1;RNU6-32P	Transcription
4	39308001	39309000	RFC1	Transcription
4	39857001	39858000	PDS5A	Epigenetic
4	39967001	39968000	PDS5A;PABPC1P1	Epigenetic
4	40096001	40097000	N4BP2	
4	40147001	40149000	N4BP2;N4BP2	;
4	40569001	40570000	RBM47	Metabolism
4	40693001	40694000	LOC107986225	
4	40835001	40836000	APBB2	
4	41195001	41196000	APBB2	
4	41502001	41503000	LIMCH1	Cytoskeleton
4	41980001	41981000	DCAF4L1;SLC30A9	Transport
4	42084001	42085000	SLC30A9	Transport
4	42360001	42361000	LOC105374428	
4	44181001	44182000	KCTD8	Cytoskeleton
4	46298001	46299000	GABRA2	Ion Channel

4	49155001	49156000	LOC101927209	
4	49166001	49167000	LOC101927209	
4	49173001	49174000	LOC101927209	
4	49201001	49202000	LOC101927209;LOC100419003	
4	49502001	49503000	ANKRD20A17P	
4	49591001	49592000	SNX18P25	
4	52748001	52749000	ERVMER34-1;LOC107986281	Epigenetic
4	53033001	53034000	SCFD2	Transport
4	53207001	53208000	SCFD2	Transport
4	53247001	53248000	SCFD2	Transport
4	55079001	55080000	KDR	Receptor
4	55404001	55405000	TMEM165	
4	56287001	56288000	CRACD;RNU6-197P	
4	61663001	61664000	ADGRL3	Signaling
4	64767001	64768000	RPS6P5;LOC107986284	
4	69532001	69533000	UGT2B4	
4	73430001	73431000	ALB;AFP	Transport
4	73750001	73751000	CXCL8	Growth Factors
4	75445001	75446000	LOC107986230	
4	76190001	76191000	SCARB2	Transport
4	76267001	76269000	FAM47E;LOC105377286;FAM47E-STBD1;FAM47E;LOC105377286;FAM47E-STBD1	;
4	76526001	76527000	SHROOM3;RNU6-145P	Cytoskeleton
4	76553001	76554000	SHROOM3	Cytoskeleton
4	76997001	76998000	SEPTIN11;LOC105377291;LOC107986290	Cytoskeleton
4	77608001	77609000	CXCL13	Growth Factors
4	77940001	77941000	MRPL1;LOC102724916	Translation
4	78619001	78620000	ANXA3	Signaling
4	79556001	79557000	LINC00989;LOC107986294	
4	79674001	79675000	LINC02469	
4	80868001	80869000	CFAP299	Development
4	82943001	82944000	LIN54	
4	83294001	83295000	COQ2;HPSE	Metabolism;Metabolism
4	84608001	84609000	CDS1	Transport
4	86169001	86170000	MAPK10;MAPK10-AS1	Signaling
4	86174001	86175000	MAPK10;MAPK10-AS1	Signaling
4	86876001	86877000	RPL6P13;C4orf36	
4	87513001	87514000	SPARCL1	Extracellular Matrix
4	88199001	88200000	ABCG2;RNU6-818P;RNU6ATAC31P	Transport
4	89306001	89307000	GPRIN3	
4	90130001	90131000	CCSER1	
4	90369001	90370000	CCSER1;RN7SKP248	
4	92588001	92589000	GRID2	Receptor
4	92881001	92882000	GRID2	Receptor
4	94314001	94315000	HPGDS	Transport
4	94603001	94604000	PDLIM5	Cytoskeleton
4	97907001	97908000	STPG2;CRYZP2	Development
4	98578001	98579000	TSPAN5	
4	98998001	98999000	METAP1;MIR3684	Protease
4	99015001	99016000	METAP1;ABT1P1	Protease
4	99651001	99652000	C4orf54	
4	102483001	102484000	LOC105377621	
4	102626001	102627000	NFKB1;LOC105377347;MANBA	Transcription;Golgi
4	102730001	102731000	MANBA;LRRC37A15P;KRT8P46;LOC100288914	Golgi
4	105455001	105456000	PPA2	Signaling
4	108763001	108764000	ETNPPL	
4	109138001	109139000	COL25A1	Extracellular Matrix
4	109203001	109204000	COL25A1	Extracellular Matrix
4	109689001	109690000	MCUB;CASP6	Protease
4	110039001	110040000	ELOVL6	Metabolism
4	112378001	112379000	ALPK1	Signaling
4	112713001	112714000	ANK2	
4	113366001	113367000	ANK2	
4	114835001	114836000	NDST4	Transport
4	115143001	115144000	MRPS33P3	
4	122825001	122826000	RPL34P12;FGF2	Growth Factors
4	123136001	123137000	SPATA5	

4	123724001	123725000	LINC01091	
4	124094001	124095000	LOC105377407	
4	129963001	129964000	LINC02465	
4	131104001	131105000	LOC105377422	
4	131727001	131729000	RN7SL205P;RN7SL205P	;
4	137420001	137422000	STMN1P2;STMN1P2	;
4	137734001	137735000	LOC105377444	
4	139027001	139028000	NOCT	Translation
4	139288001	139289000	MGARP;NDUFC1	Metabolism
4	139620001	139621000	LOC101927490	
4	141115001	141116000	RNF150	
4	143171001	143172000	LOC105377623	
4	144684001	144685000	HHIP	Signaling
4	145833001	145834000	ZNF827	Transcription
4	146286001	146287000	SLC10A7;LOC105377472	Transport
4	147016001	147017000	LOC105377475	
4	147455001	147456000	PRMT5P1	
4	147507001	147508000	EDNRA;GTF2F2P1	
4	148032001	148033000	ARHGAP10	Signaling
4	149144001	149145000	LINC02430;LINC02355	
4	149778001	149779000	IQCM	
4	149786001	149787000	IQCM;AKIRIN2P1	
4	150582001	150583000	LRBA;LOC729558;MAB21L2	
4	151029001	151030000	AK4P6	
4	151412001	151413000	FHIP1A-DT;FHIP1A	
4	151824001	151825000	LOC105377488	
4	152520001	152521000	FBXW7	Proteolysis
4	152617001	152618000	RPS14P6;TMEM154	
4	153581001	153582000	TMEM131L	
4	153990001	153991000	LOC101927947	
4	154135001	154136000	LOC101927947	
4	156125001	156126000	LOC102724785	
4	158367001	158368000	LOC105377510	
4	161090001	161091000	LOC105377514	
4	165456001	165457000	CPE	Protease
4	165548001	165549000	NOL8P1	
4	165755001	165756000	LINC01179	
4	167063001	167064000	SPOCK3	Extracellular Matrix
4	169671001	169672000	CLCN3	Transport
4	174181001	174182000	LINC02268	
4	176269001	176270000	ASB5	
4	177226001	177227000	LOC105377557	
4	177908001	177909000	LINC01098;LINC01099	
4	182243001	182244000	TENM3	
4	182285001	182286000	TENM3	
4	182326001	182327000	TENM3	
4	182392001	182393000	TENM3	
4	182777001	182778000	TENM3;LOC105377571	
4	182987001	182988000	LOC107986327	
4	183078001	183079000	LOC105377578	
4	183285001	183286000	WWC2	
4	183543001	183545000	LOC101929996;LOC101929996	;
4	183553001	183554000	LOC101929996;LOC105377579;LOC105377580	
4	183884001	183886000	STOX2;STOX2	;
4	183892001	183893000	STOX2	
4	184256001	184257000	RPL6P16;LOC105377582;LOC105377584	
4	185720001	185721000	SORBS2	
4	186290001	186291000	F11;F11-AS1	Protease
4	186432001	186433000	F11-AS1	
4	186435001	186436000	F11-AS1	
4	186446001	186447000	F11-AS1	
4	186896001	186897000	LOC102723906;LOC105377599;MRPS36P2	
4	188458001	188459000	LOC105377609;LINC01060	
4	189546001	189547000	LOC105377615	
4	189846001	189847000	FRG1-DT	
4	189881001	189882000	FRG1-DT;LINC01596	

4	189948001	189949000	FRG1-DT;FRG1	Translation
4	189984001	189985000	TUBB7P	
4	190069001	190071000	CLUHP4;DBET;DUX4L8;DUX4L7;DUX4L6;DUX4L5;CLUHP4;DBET;DUX4L8;DUX4L7;DUX4L6;DUX4L5	Development;Development
4	190122001	190123000	LOC107986338	
4	190181001	190182000	DUX4	Development
5	265001	267000	SDHA;PDCD6-DT;PDCD6-AHRR;PDCD6;SDHA;PDCD6-DT;PDCD6-AHRR;PDCD6	Metabolism;Cytoskeleton;Metabolism;Cytoskeleton
5	344001	345000	PDCD6-AHRR;AHRR	Transcription
5	376001	377000	PDCD6-AHRR;AHRR	Transcription
5	393001	395000	PDCD6-AHRR;AHRR;LOC100310782;PDCD6-AHRR;AHRR;LOC100310782	Transcription;Transcription
5	397001	399000	PDCD6-AHRR;AHRR;LOC100310782;PDCD6-AHRR;AHRR;LOC100310782	Transcription;Transcription
5	547001	548000	LOC105374606	
5	633001	634000	CEP72	
5	649001	650000	CEP72;TPPP	Cytoskeleton
5	856001	857000	ZDHHC11;BRD9	Epigenetic
5	882001	883000	BRD9;TRIP13	Epigenetic
5	1042001	1043000	NKD2;SLC12A7	Transport
5	1048001	1049000	NKD2;SLC12A7	Transport
5	1149001	1150000	SLC12A7	Transport
5	1213001	1214000	SLC6A19	Transport
5	1273001	1274000	TERT	Transcription
5	1388001	1390000	LINC01511;SLC6A3;LINC01511;SLC6A3	Transport;Transport
5	1482001	1483000	LPCAT1	Metabolism
5	1622001	1623000	LOC728613;LOC100132773;LOC112267946	
5	1985001	1986000	LOC105374618;LOC105374616	
5	3480001	3481000	LINC01019	
5	3502001	3503000	LINC01019;LINC01017	
5	3781001	3782000	LOC105374625	
5	4775001	4776000	LOC107986400;LINC02114	
5	4843001	4844000	LOC107986400	
5	5458001	5459000	ICE1	Golgi
5	6587001	6588000	LINC01018	
5	6869001	6870000	LOC105374642	
5	10458001	10459000	ROPN1L	
5	13466001	13467000	LOC105374660	
5	13991001	13992000	DNAH5	Cytoskeleton
5	14310001	14311000	TRIO	Transcription
5	15503001	15504000	FBXL7	
5	17230001	17231000	BASP1;RNU6-1003P	
5	17368001	17369000	LINC02111	
5	17386001	17387000	LINC02111	
5	17518001	17520000	H3P18;TAF11L3;TAF11L4;TAF11L5;TAF11L6;H3P18;TAF11L3;TAF11L4;TAF11L5;TAF11L6	Transcription;Transcription
5	17794001	17795000	LOC105374666	
5	19549001	19550000	CDH18	Cytoskeleton
5	21478001	21479000	GUSBP1	
5	21492001	21494000	GUSBP1;GUSBP1	;
5	22639001	22640000	CDH12	Cytoskeleton
5	24104001	24105000	LINC02899	
5	25901001	25902000	MSNP1	
5	31703001	31704000	PDZD2	Cytokine
5	31832001	31833000	PDZD2;RPL9P17;RNU6-760P;RPL21P56	Cytokine
5	32002001	32003000	PDZD2	Cytokine
5	32128001	32129000	GOLPH3	
5	32561001	32562000	SUB1	Transcription
5	33049001	33050000	LOC105374715	
5	33861001	33862000	ADAMTS12	Protease
5	33884001	33885000	ADAMTS12;RNU6-923P	Protease
5	33998001	33999000	AMACR;C1QTNF3-AMACR	
5	35139001	35140000	PRLR	Receptor
5	37132001	37133000	CPLANE1;RBISP2	
5	37143001	37144000	CPLANE1	
5	37192001	37193000	CPLANE1	
5	37475001	37476000	WDR70	
5	39415001	39416000	DAB2	Cytoskeleton
5	40597001	40598000	LOC105374737	
5	40817001	40818000	RPL37	Translation

5	42488001	42490000	GHR;GHR	Receptor;Receptor
5	43681001	43682000	NNT	Metabolism
5	52048001	52049000	LINC02118	
5	52781001	52782000	ITGA1;PELO	Extracellular Matrix;Translation
5	53964001	53965000	ARL15	
5	54017001	54018000	ARL15	
5	54146001	54147000	ARL15	
5	55429001	55430000	MTREX;PLPP1	Transcription;Signaling
5	56258001	56259000	RNA5SP185	
5	56903001	56904000	MAP3K1;LOC105378980;SETD9	
5	57603001	57604000	LINCR-0003	
5	58152001	58153000	PGAM1P1	
5	61329001	61330000	LOC105378994;ZSWIM6	
5	61392001	61394000	ZSWIM6;RPL3P6;ZSWIM6;RPL3P6	;
5	62148001	62149000	LOC105378998;RN7SKP157	
5	62543001	62544000	IPO11	Transport
5	68955001	68956000	LOC105379015;LOC107984116	
5	69299001	69300000	CCDC125	
5	69419001	69420000	RAD17;MARVELD2	Transcription
5	69894001	69895000	LOC728506	
5	70099001	70100000	NAIPP2	
5	70423001	70424000	LOC107986356;GTF2H2B	
5	70493001	70494000	LOC107986356;GUSBP15;LOC441081	
5	70542001	70543000	LOC107986356;GUSBP15	
5	70681001	70683000	LOC107986356;LOC107986355;LOC107986358;LOC107986356;LOC107986355	;
5	71220001	71222000	LOC728452;GUSBP17;LOC728452;GUSBP17	;
5	71650001	71651000	MCCC2	Metabolism
5	73643001	73644000	ARHGEF28;LOC107986423	
5	76437001	76438000	IQGAP2	Signaling
5	76619001	76620000	IQGAP2;F2RL2	Signaling;Signaling
5	78376001	78377000	SCAMP1	Transport
5	78438001	78439000	SCAMP1	Transport
5	79507001	79508000	HOMER1;RPL29P15	
5	79849001	79850000	LOC102724557;LOC105379048	
5	80047001	80048000	THBS4	
5	80661001	80662000	DHFR;MTRNR2L2;MSH3	Metabolism;Transcription
5	81208001	81209000	RASGRF2	Transcription
5	82896001	82897000	LOC107986430	
5	84182001	84183000	EDIL3	Metabolism
5	87270001	87272000	RASA1;RASA1	Signaling;Signaling
5	88605001	88606000	LINC00461;H3P23	
5	89004001	89005000	MEF2C-AS1	
5	90849001	90850000	ADGRV1	Signaling
5	91124001	91125000	ADGRV1	Signaling
5	93867001	93868000	FAM172A	
5	94011001	94012000	FAM172A	
5	94824001	94825000	MCTP1;RPL7P18	
5	95973001	95974000	LOC101929710;FABP5P5	
5	96364001	96365000	LOC101929710	
5	96592001	96593000	LOC101929710	
5	96923001	96924000	ERAP1;ERAP2	Protease
5	99013001	99014000	CHD1-DT	
5	99064001	99065000	LOC107986436	
5	100051001	100052000	MTCYBP22;MTND6P22;MTND5P10;MTND4P35;MTND3P19;MTCO3P22;MTATP6P2;MTATP8P3;MTCO2P22;MTCO1P22	
5	103827001	103828000	LOC105379107	
5	108121001	108122000	FBXL17	
5	108782001	108783000	FER;RACK1P1	
5	108805001	108806000	FER	
5	108807001	108808000	FER	
5	109235001	109236000	LOC285638	
5	111702001	111703000	STARD4-AS1	
5	112286001	112287000	EPB41L4A	
5	112699001	112700000	APC	
5	113752001	113753000	LOC105379127	
5	114058001	114059000	KCNN2	Transport

5	114258001	114259000	KCNN2;RN7SKP89	Transport
5	115226001	115227000	PGGT1B	Metabolism
5	115577001	115578000	TMED7-TICAM2;TICAM2	Transport
5	118573001	118574000	LINC02216	
5	118630001	118631000	LINC02215	
5	118923001	118924000	DTWD2	
5	118982001	118983000	DTWD2;PTMAP2;MIR1244-2	
5	119320001	119321000	TNFAIP8	
5	122411001	122412000	SNCAIP	
5	123378001	123380000	CEP120;CEP120	;
5	126176001	126178000	LINC02039;LINC02039	;
5	126447001	126448000	GRAMD2B	
5	126874001	126875000	MARCHF3	Proteolysis
5	128399001	128400000	FBN2	Extracellular Matrix
5	128932001	128933000	LOC105379168	
5	129606001	129607000	ADAMTS19	Protease
5	129645001	129646000	ADAMTS19	Protease
5	130016001	130017000	CHSY3	Golgi
5	130797001	130798000	LOC107986449	
5	131168001	131169000	HINT1;LYRM7	Signaling;Transcription
5	131612001	131613000	RAPGEF6	Transcription
5	132861001	132862000	GDF9;UQCRQ	Growth Factors;Metabolism
5	133296001	133297000	FSTL4	Protease; Proteolysis
5	133422001	133423000	FSTL4;MIR1289-2	Protease; Proteolysis
5	134039001	134040000	VDAC1	Transport
5	134144001	134145000	TCF7;SKP1	Transcription;Proteolysis
5	134151001	134152000	TCF7;SKP1	Transcription;Proteolysis
5	134538001	134539000	JADE2;LOC107986451	Transcription
5	134571001	134572000	JADE2	Transcription
5	134745001	134746000	CAMLG	
5	134747001	134748000	CAMLG	
5	134768001	134769000	DDX46	
5	134915001	134916000	PCBD2;MTCYBP18;MTND6P4;MTND5P11	Metabolism
5	135177001	135178000	PITX1-AS1	
5	135749001	135750000	SLC25A48	Transport
5	138009001	138010000	FAM13B	
5	138979001	138980000	SIL1;LOC105379195	
5	139086001	139087000	SIL1	
5	139205001	139206000	SIL1	
5	140099001	140100000	MALINC1	
5	140255001	140256000	PFDN1	Transcription
5	140638001	140639000	TMCO6;CD14;NDUFA2;IK;MIR3655	Receptor;Metabolism;Growth Factors
5	140686001	140687000	HARS1;HARS2	Translation
5	141193001	141194000	PCDHB@;PCDHB16;PCDHB9;PCDHB10;PCDHB11	Cytoskeleton
5	141350001	141351000	PCDHGA1;PCDHG@;PCDHGA2;PCDHGA3;PCDHGB1;PCDHGA4;PCDHGB2	Cytoskeleton
5	141497001	141498000	PCDHGA1;PCDHG@;PCDHGA2;PCDHGA3;PCDHGB1;PCDHGA4;PCDHGB2;PCDHGA5;PCDHGB3;PCDHGA6;PCDHGA7;PCDHGB4;PCDHGA8;PCDHGB5;PCDHGA9;PCDHGB6;PCDHGA10;PCDHGB7;PCDHGA11;PCDHGA12;PCDHGC3;PCDHGC4;PCDHGC5	Cytoskeleton
5	141540001	141541000	DIAPH1	
5	141649001	141650000	RELL2;FCHSD1;ARAP3	Signaling
5	142343001	142344000	SPRY4-AS1	
5	142416001	142417000	SPRY4-AS1	
5	142895001	142896000	ARHGAP26	Signaling
5	143762001	143763000	LOC100420513	
5	146706001	146708000	PPP2R2B;KRT8P48;PPP2R2B;KRT8P48	Signaling;Signaling
5	148461001	148462000	HTR4;LOC107986462	Signaling
5	148576001	148577000	HTR4	Signaling
5	148611001	148612000	HTR4	Signaling
5	151380001	151381000	LOC105378234;LOC100420127;LOC100419720	
5	151763001	151764000	ATOX1;ATOX1-AS1;RPLP1P6;LOC100652758;G3BP1	Metabolism
5	152940001	152941000	LINC01470	
5	153949001	153950000	LOC107986464	
5	154809001	154810000	LARP1;FAXDC2	Metabolism;Metabolism
5	154964001	154965000	MRPL22	Translation
5	155845001	155846000	SGCD;RNA5SP199	Cytoskeleton
5	156649001	156650000	SGCD	Cytoskeleton

5	157424001	157425000	NIPAL4-DT;RPL26P18	
5	157677001	157678000	SOX30;C5orf52	Transcription
5	158384001	158385000	LINC02227	
5	158904001	158905000	EBF1	Transcription
5	160823001	160824000	ATP10B	Transport
5	162770001	162771000	ARL2BPP5	
5	163012001	163013000	LOC105377699	
5	164405001	164406000	LOC105377703	
5	167381001	167382000	TENM2	
5	168262001	168263000	TENM2	
5	168367001	168368000	WWC1	
5	168445001	168446000	WWC1	
5	168923001	168924000	SLIT3;LOC107986472	
5	170562001	170563000	KCNIP1	
5	171245001	171246000	RANBP17;USP12P1	Transport
5	171871001	171872000	FBXW11	
5	172608001	172609000	LOC105377729;LOC107986478	
5	172758001	172759000	LOC101928093;LOC401218;DUSP1	Signaling
5	172787001	172788000	LOC105377730	
5	172840001	172841000	ERGIC1	
5	173115001	173116000	CREBRF	
5	173265001	173266000	LOC105377731	
5	173562001	173564000	LOC105377733;LOC105377733	;
5	173679001	173680000	LINC01942	
5	173697001	173698000	LINC01942;LINC01484	
5	174051001	174052000	NSG2	
5	175908001	175909000	LOC107986485	
5	176251001	176252000	SIMC1	
5	176281001	176282000	SIMC1	
5	176422001	176423000	ARL10;CLTB	Transport
5	176800001	176801000	UNC5A	Receptor
5	177354001	177355000	LMAN2;RPS20P17;RGS14	Transport
5	177704001	177705000	LOC107986489;FAM153A	
5	177808001	177809000	LOC105377752;LOC107986490;LOC107986491	
5	177961001	177963000	LOC100128340;SUDS3P1;LOC100128340;SUDS3P1	;
5	178083001	178085000	LOC105377754;LOC105377754	;
5	178327001	178328000	COL23A1	Extracellular Matrix
5	178524001	178525000	COL23A1	Extracellular Matrix
5	179074001	179075000	ZNF354C	
5	179255001	179256000	ADAMTS2	Protease
5	179348001	179349000	ADAMTS2;LOC105377759	Protease
5	179545001	179546000	LOC105377762;RUFY1	
5	179746001	179747000	MAML1	Transcription
5	179883001	179884000	TBC1D9B	Signaling
5	179898001	179899000	TBC1D9B	Signaling
5	180252001	180253000	MAPK9;LOC100419721	Signaling
5	180279001	180280000	MAPK9	Signaling
5	180535001	180536000	CNOT6;LOC100329129	Translation
5	180619001	180620000	FLT4	Receptor
5	180948001	180949000	BTNL8;RPS29P12	Immune
5	181004001	181005000	BTNL3;RNU6-1036P	Immune
5	181063001	181064000	BTNL9;RPL13P10	Immune
5	181075001	181076000	RPL13P10	
5	181264001	181266000	CTC-338M12.4;TRIM52;TRIM52-AS1;CTC-338M12.4;TRIM52;TRIM52-AS1	Proteolysis;Proteolysis
5	181279001	181281000	TRIM52-AS1;TRIM52-AS1	;
5	181384001	181385000	WBP1LP4	
6	199001	200000	LOC285766	
6	519001	520000	EXOC2	
6	543001	544000	EXOC2	
6	589001	590000	EXOC2	
6	720001	721000	LOC105374873	
6	1068001	1070000	LINC01622;LINC01622	;
6	1615001	1616000	FOXCUT;FOXC1;GMDS	Transcription;Metabolism
6	3005001	3006000	NQO2-AS1;NQO2	Metabolism
6	3087001	3088000	RIPK1;RNA5SP201	Signaling
6	3115001	3116000	RIPK1;LOC107986556;BPHL	Signaling;Metabolism

6	3168001	3169000	LOC105374889;TUBB2BP1	
6	3272001	3273000	PSMG4;SLC22A23	Transcription;Transport
6	3849001	3850000	FAM50B;LOC107986557	
6	3986001	3987000	GLRX3P2	
6	4927001	4928000	CDYL	
6	5073001	5074000	LYRM4-AS1;LYRM4;LOC442155	
6	6275001	6276000	F13A1	Transport
6	7437001	7438000	LOC102724234	
6	7671001	7672000	LOC105374906	
6	7838001	7839000	BMP6	Growth Factors
6	8040001	8042000	BLOC1S5-TXNDC5;EEF1E1-BLOC1S5;BLOC1S5;BLOC1S5-TXNDC5;EEF1E1-BLOC1S5;BLOC1S5	;
6	8667001	8668000	LOC100506207	
6	9883001	9884000	OFCC1	
6	9970001	9971000	OFCC1	
6	10076001	10077000	OFCC1	
6	10149001	10150000	OFCC1;LOC105374918	
6	10361001	10362000	LOC105374921;LOC442161	
6	10806001	10807000	MAK	Signaling
6	11147001	11148000	SMIM13	
6	12163001	12164000	HIVEP1	
6	13338001	13339000	TBC1D7-LOC100130357;TBC1D7	Signaling
6	13366001	13367000	LOC105374936;GFOD1	Metabolism
6	13590001	13591000	SIRT5;LOC105374938	
6	14570001	14571000	LOC101928354	
6	15311001	15312000	JARID2;RNU6-522P	Epigenetic
6	15385001	15386000	JARID2	Epigenetic
6	16251001	16252000	GMPR	Metabolism
6	17020001	17021000	LOC105374951	
6	17641001	17642000	NUP153	Transport
6	17759001	17760000	KIF13A	Cytoskeleton
6	18406001	18407000	RNF144B	Proteolysis
6	18506001	18507000	LOC105374955	
6	19698001	19700000	LOC105374959;LOC105374959	;
6	20672001	20673000	CDKAL1	
6	20801001	20802000	CDKAL1	
6	20907001	20908000	CDKAL1	
6	21152001	21153000	CDKAL1	
6	21231001	21232000	CDKAL1;LOC107986578	
6	21493001	21494000	LINC00581	
6	21803001	21804000	CASC15	
6	21866001	21867000	CASC15	
6	22013001	22014000	CASC15	
6	22131001	22132000	CASC15;NBAT1	
6	22711001	22712000	LOC105374972	
6	23401001	23402000	LOC102724749;LOC105374976	
6	24420001	24421000	MRS2;GPLD1	Translation;Metabolism
6	24816001	24817000	RIPOR2	Signaling
6	25473001	25474000	CARMIL1	
6	25920001	25921000	SLC17A2	Transport
6	26877001	26878000	POM121L6P;GUSBP2	
6	26889001	26890000	POM121L6P;GUSBP2;LOC112267954	
6	27225001	27226000	TRR-ACG2-3;TRL-TAA4-1;TRV-AAC5-1	
6	27525001	27526000	LOC107986584;TRQ-CTG1-2;HNRNPA1P1;TRS-AGA3-1	
6	27582001	27583000	TRK-TTT9-1;TRD-GTC3-1;TRK-TTT4-1;TRX-CAT1-6	
6	27734001	27735000	TRT-AGT4-1;TRV-CAC7-1;GPR89P	
6	27744001	27745000	GPR89P;TRV-AAC1-5	
6	28122001	28123000	ZNF602P;ZSCAN16-AS1;ZSCAN16	Transcription
6	28899001	28900000	ZNF90P2;TRL-CAA1-1;HCG14;TRIM27	Proteolysis
6	28926001	28927000	TRIM27	Proteolysis
6	29799001	29800000	HCG4;HLA-V;HCG4P9;HLA-P;RPL7AP7	
6	30032001	30033000	ZNRD1ASP;ETF1P1	
6	30826001	30827000	LINC00243	
6	31166001	31167000	CCHCR1;TCF19;POU5F1;PSORS1C3	Transcription
6	31196001	31197000	PSORS1C3;HCG27	
6	31306001	31307000	LINC02571;LOC112267902	

6	31454001	31455000	LINC01149;HLA-X;HCP5	
6	31612001	31613000	UQCRHP1;AIF1;PRRC2A	Signaling;Metabolism
6	31645001	31647000	PRRC2A;BAG6;APOM;PRRC2A;BAG6;APOM	Metabolism;Transcription;Binding Proteins;Metabolism
6	31723001	31724000	LY6G6F-LY6G6D;LY6G6E;LY6G6D;LY6G6C;MPIG6B;DDAH2;CLIC1	Receptor;Metabolism;Transport
6	31878001	31879000	SLC44A4;EHMT2;LOC107986588	Transport
6	31913001	31914000	C2	
6	32131001	32132000	ATF6B;FKBPL	Transcription
6	32156001	32157000	PRRT1;LOC100507547;PPT2;PPT2-EGFL8;EGFL8	Golgi;Signaling
6	32928001	32929000	HLA-DMB	Immune
6	33295001	33297000	WDR46;MIR6873;PFDN6;MIR6834;RGL2;TAPBP;WDR46;MIR6873;PFDN6;MIR6834;RGL2;TAPBP	Transcription;Immune;Transcription;Immune
6	33331001	33332000	DAXX;SMIM40;MYL12BP3	
6	33382001	33383000	RPL35AP4;KIFC1	Cytoskeleton
6	33393001	33394000	RPL35AP4;KIFC1;RPL12P1	Cytoskeleton
6	33600001	33601000	LINC00336	
6	34071001	34072000	GRM4	Signaling
6	34083001	34084000	GRM4	Signaling
6	34640001	34641000	ILRUN	
6	34810001	34811000	UHRF1BP1	
6	35374001	35375000	PPARD	Transcription
6	35565001	35566000	LOC101929309;RPS15AP19;FKBP5	Transcription
6	36941001	36942000	PI16	Immune
6	37282001	37283000	TBC1D22B	Signaling
6	37692001	37693000	MDGA1	
6	38010001	38011000	ZFAND3	
6	38054001	38055000	ZFAND3	
6	38162001	38163000	ZFAND3;BTBD9	
6	38767001	38768000	DNAH8;ZRF1PS	Cytoskeleton
6	39584001	39585000	KIF6	Cytoskeleton
6	39676001	39677000	KIF6	Cytoskeleton
6	39724001	39725000	KIF6	Cytoskeleton
6	40511001	40512000	LRFN2	
6	41243001	41244000	TREML4;RNA5SP207;TREML5P	
6	41346001	41347000	NCR2	
6	41454001	41455000	LOC112267957	
6	41831001	41832000	USP49	Protease
6	42329001	42330000	TRERF1	
6	42924001	42925000	PTCRA;CNPY3;CNPY3-GNMT	
6	43014001	43015000	MEA1;PPP2R5D;KLHDC3;RRP36	Signaling
6	43160001	43161000	PTK7	Receptor
6	43381001	43382000	LOC105375065	
6	43738001	43739000	POLR1C	Transcription
6	43961001	43962000	POLR1C	Transcription
6	44044001	44045000	POLR1C;SCIRT	Transcription
6	44177001	44178000	POLR1C;CAPN11;LOC105375072	Transcription;Protease
6	44252001	44253000	POLR1C;HSP90AB1;SLC35B2;MIR4647;NFKBIE	Transcription;Signaling;Transport;Transport
6	44283001	44284000	POLR1C;TMEM151B;TCTE1	Transcription;Cytoskeleton
6	52829001	52830000	GSTA5	Transport
6	53950001	53951000	LOC101927189	
6	55376001	55377000	GFRAL	Receptor
6	56012001	56013000	LOC105375100	
6	56752001	56753000	DST	Cytoskeleton
6	57262001	57263000	LOC100506188	
6	57345001	57346000	PRIM2	Cell Cycle
6	57805001	57806000	LOC105375103;TRI-AAT1-1;LOC105375102	
6	61791001	61792000	KHDRBS2	Translation
6	63229001	63230000	SPTLC1P3;LOC100128610	
6	68700001	68701000	ADGRB3	Signaling
6	68758001	68759000	ADGRB3	Signaling
6	69713001	69714000	LMBRD1;NPM1P37	Transport
6	70187001	70188000	COL19A1	Extracellular Matrix
6	70419001	70420000	FAM135A-AS1;FAM135A	
6	73201001	73202000	KCNQ5;KHDC1P1	Transport
6	73251001	73252000	KHDC1	
6	73470001	73471000	MTO1;RNU6-975P	
6	73476001	73477000	MTO1	

6	75594001	75595000	H3P27;RNU6-1338P;SENP6	Protease
6	77659001	77660000	MEI4	
6	80456001	80457000	BCKDHB	Metabolism
6	84682001	84683000	TBX18-AS1	
6	86077001	86078000	RPL7P27	
6	87303001	87304000	GJB7;HSPD1P10	Cytoskeleton
6	89150001	89151000	PM20D2;LOC101929004	Protease
6	89484001	89485000	ANKRD6;RN7SL11P	
6	89504001	89505000	ANKRD6;LOC105377889	
6	89840001	89841000	CASP8AP2	Signaling
6	90554001	90555000	MAP3K7	Signaling
6	90737001	90738000	LOC107986623	
6	90760001	90761000	LOC107986623	
6	92586001	92587000	LOC105377896	
6	95500001	95501000	CYCSP17	
6	97057001	97058000	KLHL32	
6	97180001	97181000	MMS22L	
6	97226001	97227000	MMS22L	
6	98511001	98512000	LOC105377910	
6	99305001	99306000	FAXC	
6	99405001	99406000	PNISR	
6	99936001	99937000	MCHR2	Signaling
6	100985001	100986000	LOC107984041	
6	101415001	101416000	GRIK2	Receptor
6	102210001	102211000	LOC105377913	
6	104462001	104463000	LOC105377918	
6	106758001	106759000	LINC02532	
6	107226001	107227000	PDSS2;RPS24P12	
6	107498001	107499000	SOBP	
6	107506001	107507000	SOBP	
6	108236001	108237000	SNX3	
6	108401001	108402000	AFG1L;LOC105377932;RNU6-770P	
6	110015001	110016000	LOC107986631	
6	110449001	110450000	SLC22A16;RN7SL617P	Transport
6	110771001	110772000	CDK19	Signaling
6	111306001	111307000	REV3L	Transcription
6	112047001	112048000	CCN6	Growth Factors
6	112054001	112055000	CCN6	Growth Factors
6	112791001	112792000	LOC107986634	
6	112934001	112935000	LOC107986635	
6	114426001	114427000	LOC107986638	
6	114470001	114471000	LOC107986638;LOC105377959	
6	115036001	115037000	LOC105377960	
6	115051001	115052000	LOC105377960	
6	116082001	116083000	FRK	
6	116365001	116366000	DSE;LOC100287467	
6	116817001	116818000	GPRC6A	Signaling
6	116930001	116932000	RFX6;RFX6	Transcription;Transcription
6	119197001	119199000	MAN1A1;MAN1A1	Golgi;Golgi
6	121412001	121413000	HMGN2P29	
6	121948001	121949000	LOC105377979	
6	123473001	123474000	TRDN;TRDN-AS1	
6	129572001	129573000	ARHGAP18	Signaling
6	130721001	130722000	LOC105378002	
6	131407001	131408000	LOC105378005	
6	131859001	131860000	ENPP1	
6	132797001	132798000	SLC18B1;LOC107986644	Transport
6	135280001	135281000	AHI1	
6	136063001	136064000	PDE7B;LOC644135	Signaling
6	138381001	138382000	SMIM28	
6	139620001	139621000	ATP5PBP6	
6	139662001	139663000	LOC100129554	
6	141458001	141460000	LOC105378029;LOC105378029	;
6	142020001	142022000	LOC105378031;LOC105378031	;
6	143306001	143307000	AIG1	
6	143565001	143566000	PHACTR2;PHACTR2-AS1	Signaling

6	144480001	144481000	UTRN	
6	144767001	144768000	UTRN	
6	145522001	145523000	EPM2A	
6	146173001	146174000	GRM1;FUNDC2P3	Signaling
6	147324001	147325000	STXBP5	Transport
6	148894001	148895000	UST	Transport
6	149016001	149017000	UST	Transport
6	149313001	149314000	TAB2;FABP12P1	
6	149514001	149515000	PPIL4;RNU7-3P	Transcription
6	149751001	149752000	NUP43;PCMT1	Epigenetic
6	150055001	150056000	ULBP3	Immune
6	150369001	150371000	IYD;IYD	Metabolism;Metabolism
6	150843001	150844000	PLEKHG1	
6	151086001	151087000	MTHFD1L	
6	151372001	151374000	ZBTB2;ZBTB2	Transcription;Transcription
6	151448001	151449000	RMND1;ARMT1	
6	151924001	151925000	ESR1	
6	152869001	152870000	LINC02840	
6	154263001	154265000	IPCEF1;IPCEF1	;
6	154424001	154425000	CNKSR3	
6	155047001	155049000	TIAM2;TIAM2	;
6	155098001	155099000	TIAM2	
6	157041001	157043000	ARID1B;ARID1B	;
6	157266001	157267000	LOC105378075	
6	157932001	157933000	SNX9;HSPE1P26;RNU6-786P	Cytoskeleton
6	158018001	158019000	SYNJ2	Signaling
6	158182001	158183000	GTF2H5	Transcription
6	158572001	158574000	TMEM181;TMEM181	;
6	158587001	158588000	TMEM181	
6	159048001	159049000	TAGAP-AS1;TAGAP;LOC107986664;LOC112267968	Signaling
6	159078001	159079000	TAGAP-AS1;LOC112267968	
6	159211001	159212000	FNDC1	
6	160613001	160614000	LPA	Protease
6	160618001	160619000	LPA	Protease
6	160640001	160641000	LPA	Protease
6	160645001	160646000	LPA	Protease
6	162055001	162056000	PRKN	Proteolysis
6	162587001	162588000	PRKN	Proteolysis
6	163203001	163204000	PACRG;LOC105378095	
6	163285001	163286000	PACRG	
6	164096001	164097000	LOC105378106	
6	164460001	164461000	LOC107986667	
6	165714001	165715000	PDE10A;RNU6-730P	Signaling
6	165793001	165795000	PDE10A;PDE10A	Signaling;Signaling
6	165952001	165953000	PDE10A;LINC00473	Signaling
6	166638001	166639000	RPS6KA2;LOC107986671	Golgi
6	166647001	166649000	RPS6KA2;LOC107986671;RPS6KA2;LOC107986671	Golgi;Golgi
6	166829001	166830000	RPS6KA2	Golgi
6	167186001	167187000	TCP10L2	Cytoskeleton
6	167270001	167271000	UNC93A	Signaling
6	167277001	167278000	UNC93A	Signaling
6	167422001	167423000	LOC105378126	
6	167454001	167455000	LOC105378127	
6	167996001	167997000	KIF25-AS1;KIF25	Cytoskeleton
6	168270001	168271000	LOC101929420	
6	168301001	168302000	DACT2;LOC105378138	
6	168529001	168530000	SMOC2	Signaling
6	168535001	168536000	SMOC2	Signaling
6	168998001	168999000	LOC105378145	
6	169256001	169257000	THBS2	
6	169591001	169592000	WDR27	
6	169637001	169638000	WDR27	
6	169641001	169642000	WDR27	
6	169986001	169987000	LOC107986675;LOC102724475	
7	10001	11000	LOC102723872	
7	20001	21000	LOC102723872	

7	216001	217000	FAM20C	
7	281001	282000	FOXL3;FOXL3-OT1	Transcription
7	301001	302000	FOXL3;FOXL3-OT1	Transcription
7	552001	553000	PRKAR1B;PRKAR1B-AS2	Signaling
7	641001	642000	PRKAR1B	Signaling
7	643001	644000	PRKAR1B	Signaling
7	661001	662000	PRKAR1B	Signaling
7	1000001	1001000	C7orf50	
7	1033001	1034000	C7orf50;MIR339	
7	1049001	1050000	C7orf50;GPR146;LOC107986755	Signaling
7	1081001	1083000	C7orf50;LOC107986755;LOC105375120;GPER1;C7orf50;LOC107986755;LOC105375120;GPER1	Signaling;Signaling
7	1089001	1090000	C7orf50;LOC105375120;GPER1	Signaling
7	1096001	1097000	C7orf50;GPER1	Signaling
7	1117001	1118000	C7orf50;LOC102723758	
7	1480001	1481000	INTS1	
7	1489001	1490000	INTS1	
7	1523001	1524000	MAFK;LOC100128653	Transcription
7	1642001	1644000	LOC107986757;LOC107986757	;
7	1690001	1691000	ELFN1;LNCRI	Receptor
7	1693001	1694000	ELFN1;LNCRI	Receptor
7	1704001	1706000	ELFN1;LNCRI;LOC105375124;ELFN1;LNCRI;LOC105375124	Receptor;Receptor
7	1713001	1714000	ELFN1;LOC105375124	Receptor
7	1805001	1806000	MAD1L1	
7	1818001	1820000	MAD1L1;LOC105375128;MAD1L1;LOC105375128	;
7	1946001	1947000	MAD1L1	
7	2046001	2047000	MAD1L1	
7	2052001	2053000	MAD1L1	
7	2075001	2076000	MAD1L1	
7	2099001	2100000	MAD1L1;SNORA114	
7	2275001	2277000	SNX8;SNX8	Cytoskeleton;Cytoskeleton
7	2353001	2354000	SNX8;EIF3B	Cytoskeleton;Translation
7	2383001	2384000	EIF3B;KIF19BP;LOC402634	Translation
7	2481001	2482000	GRIFIN;LOC107986758	Extracellular Matrix
7	2591001	2593000	IQCE;IQCE	;
7	2850001	2851000	GNA12	Signaling
7	2966001	2967000	CARD11	
7	2969001	2971000	CARD11;LOC107986761;CARD11;LOC107986761	;
7	3235001	3236000	SDK1-AS1	
7	3941001	3942000	SDK1	
7	4204001	4205000	SDK1	
7	4267001	4268000	SDK1	
7	4716001	4718000	FOXK1;FOXK1	;
7	4898001	4899000	MMD2	Signaling
7	4907001	4908000	MMD2	Signaling
7	4927001	4928000	MMD2	Signaling
7	5139001	5140000	ZNF890P	
7	5285001	5286000	SLC29A4	Transport
7	5308001	5309000	SLC29A4;TNRC18	Transport;Transcription
7	5312001	5313000	SLC29A4;TNRC18	Transport;Transcription
7	5349001	5351000	TNRC18;TNRC18	Transcription;Transcription
7	5405001	5406000	TNRC18	Transcription
7	5534001	5535000	LOC221946;ACTB	Cytoskeleton
7	5869001	5870000	OCM;RN7SL556P	Signaling
7	6505001	6506000	GRID2IP;LOC101927325	
7	6550001	6551000	GRID2IP	
7	6622001	6624000	ZNF853;ZNF853	Transcription;Transcription
7	6744001	6745000	PMS2CL;RSPH10B2	
7	6875001	6876000	OR7E136P;OR7E59P	
7	6988001	6989000	LOC107986763;LOC105375138	
7	7465001	7466000	COL28A1	Extracellular Matrix
7	12660001	12661000	SCIN	Cytoskeleton
7	12996001	12997000	LOC105375158	
7	13591001	13592000	LOC107986770	
7	15776001	15777000	LOC105375167	
7	17537001	17538000	LINC02889	

7	18990001	18991000	HDAC9	
7	20787001	20788000	SP8	Transcription
7	21231001	21232000	ASS1P11	
7	21702001	21703000	DNAH11	Cytoskeleton
7	21899001	21900000	DNAH11;CDCA7L	Cytoskeleton
7	23167001	23168000	KLHL7	Cytoskeleton
7	23218001	23219000	LOC101927890	
7	24323001	24324000	LOC107986777	
7	24743001	24744000	GSDME	
7	25630001	25631000	LOC646588	
7	26269001	26270000	RPL23P7	
7	26317001	26318000	SNX10	
7	26345001	26346000	SNX10	
7	28247001	28248000	JAZF1-AS1	
7	28453001	28454000	CREB5	Transcription
7	29210001	29211000	CHN2;CHN2-AS1;NANOGP4	
7	29556001	29557000	LOC107986700;GTF3C6P3;PRR15	
7	30460001	30461000	NOD1;LOC101928268	Cytoskeleton
7	30482001	30483000	NOD1	Cytoskeleton
7	31099001	31100000	ADCYAP1R1	Receptor
7	31205001	31206000	LOC107986781	
7	31616001	31617000	ITPRID1;PDE1C	Signaling
7	32172001	32173000	PDE1C	Signaling
7	32469001	32470000	SLC25A5P5	
7	32522001	32523000	AVL9	
7	34342001	34343000	NPSR1-AS1	
7	36403001	36404000	ANLN	Cytoskeleton
7	37166001	37167000	ELMO1	Cytoskeleton
7	38023001	38024000	LOC105375236	
7	38915001	38916000	VPS41	Transport
7	39578001	39579000	YAE1;LOC100101126	
7	39793001	39794000	LINC00265;CICP22	
7	40514001	40515000	SUGCT	Transport
7	40625001	40626000	SUGCT	Transport
7	41488001	41489000	LOC107986787	
7	41765001	41766000	INHBA-AS1;LOC107986788	
7	41852001	41853000	LOC107986788	
7	43966001	43967000	UBE2D4;POLR2J4	Proteolysis
7	44822001	44823000	LOC105375260;H2AZ2	Epigenetic
7	45051001	45052000	CCM2	
7	45489001	45490000	LOC107986737	
7	45812001	45813000	GTF2IP13;LOC730234;LOC112267918;LOC112267911;CICP20;SEPTIN14P7	
7	45883001	45884000	IGFBP1	Protease; Proteolysis
7	47417001	47418000	TNS3	Cytoskeleton
7	48535001	48536000	ABCA13	Transport
7	49822001	49823000	VWC2	
7	50597001	50598000	GRB10	Cytoskeleton
7	50782001	50784000	GRB10;GRB10	Cytoskeleton;Cytoskeleton
7	51076001	51077000	COBL	
7	53401001	53402000	LOC105375282	
7	55157001	55158000	EGFR	Receptor
7	55347001	55348000	LOC100129276	
7	55731001	55732000	SUMO2P3;CICP11;LOC107986799	
7	55833001	55834000	SEPTIN14	Cytoskeleton
7	55892001	55893000	ZNF713	Transcription
7	56049001	56050000	PSPH;CCT6A;SNORA22B	Signaling;Translation
7	56068001	56069000	CCT6A;SNORA15;SUMF2	Translation
7	56286001	56287000	LOC441228	
7	56367001	56368000	SEPTIN14P24;CICP8;LOC731631;LOC100419985;LOC100533648;LOC100419986;LOC100533649;LOC107986800	
7	56370001	56371000	SEPTIN14P24;CICP8;LOC731631;LOC100419985;LOC100533648;LOC100419986;LOC100533649;LOC107986800	
7	56373001	56374000	CICP8;LOC731631;LOC100419985;LOC100533648;LOC100419986;LOC100533649;LOC107986800;LOC105375292	
7	56430001	56431000	LOC650226	
7	56482001	56483000	LOC100240728;RBM22P3	

7	56573001	56574000	LOC100130210	
7	56605001	56606000	LOC346296;LOC100289196;LOC105375291	
7	56829001	56831000	LOC100533651;LOC100507393;LOC101928507;LOC112267998;LOC105375294;LOC100533651;LOC100507393;LOC101928507;LOC112267998;LOC105375294	;
7	56832001	56833000	LOC100507393;LOC101928507;LOC112267998;LOC105375294	
7	56986001	56987000	TNRC18P3	
7	57238001	57240000	LOC105375298;LOC105375298	;
7	57404001	57405000	LOC105375299;LOC100653233;MIR3147	
7	57601001	57602000	NCOR1P3	
7	57607001	57608000	NCOR1P3	
7	57868001	57869000	LOC102723842	
7	63154001	63155000	SEPTIN14P1	
7	63243001	63244000	PHKG1P1;SEPTIN7P4	
7	63250001	63251000	SEPTIN7P4	
7	63590001	63591000	TNRC18P2	
7	63750001	63751000	LOC112267999;LOC112267913	
7	65169001	65170000	INTS4P1	
7	65234001	65235000	INTS4P1	
7	65342001	65343000	LOC105375334;RSL24D1P3	
7	66037001	66038000	LOC105375336;LOC100419772	
7	66347001	66348000	TPST1;RNU6-313P	Transport
7	66472001	66473000	LOC107986704	
7	66714001	66715000	RABGEF1	Transcription
7	67038001	67039000	TYW1	Metabolism
7	67073001	67074000	TYW1	Metabolism
7	69400001	69402000	RNU6-229P;RNU6-229P	;
7	69704001	69705000	AUTS2	
7	70040001	70041000	AUTS2	
7	71360001	71361000	GALNT17	Golgi
7	72659001	72660000	TYW1B	Metabolism
7	72963001	72964000	NSUN5P2;TRIM74;LOC100101148	Proteolysis
7	73052001	73053000	SPDYE11	
7	73266001	73267000	GTF2IRD2P1	
7	73540001	73542000	BCL7B;BCL7B	;
7	73695001	73696000	BUD23;STX1A;LOC105375350	Epigenetic;Transcription
7	73820001	73821000	CLDN4	Cell Junction
7	74092001	74093000	LIMK1	
7	74356001	74358000	CLIP2;CLIP2	Transcription;Transcription
7	74397001	74398000	CLIP2	Transcription
7	74529001	74530000	GTF2IRD1;WBSCR23	Transcription
7	74541001	74542000	GTF2IRD1;WBSCR23	Transcription
7	74568001	74569000	GTF2IRD1	Transcription
7	74585001	74586000	GTF2IRD1;MIR10525	Transcription
7	75317001	75318000	SPDYE14;PMS2P10	
7	75421001	75422000	NSUN5P1;POM121C	Transport
7	75733001	75734000	HIP1	Cytoskeleton
7	75786001	75787000	CCL26	Growth Factors
7	76208001	76209000	SRRM3	
7	76361001	76362000	YWHAG	Cytoskeleton
7	77070001	77071000	SPDYE18;LOC100422695	
7	77114001	77115000	FAM185BP;RPL7AP43;CCDC146	Development
7	77823001	77824000	PHTF2	Development
7	78232001	78233000	MAGI2	
7	78808001	78809000	MAGI2	
7	83462001	83463000	SEMA3E;LOC105375378	Signaling
7	85450001	85451000	LINC00972	
7	86934001	86935000	ELAPOR2	
7	87463001	87464000	ABCB4	Transport
7	87509001	87510000	ABCB1	Transport
7	88934001	88935000	ZNF804B	
7	91217001	91218000	CDK14	Signaling
7	92371001	92372000	ANKIB1	Proteolysis
7	92977001	92978000	RN7SL7P	
7	95810001	95811000	DYNC1I1	Cytoskeleton
7	96287001	96288000	SLC25A13;RPL21P74	Transport
7	96289001	96290000	SLC25A13;RPL21P74	Transport

7	96682001	96683000	SEM1	Protease
7	97909001	97910000	ASNS;CZ1P-ASNS;RPS3AP29	Metabolism
7	98109001	98110000	LMTK2	Receptor
7	98173001	98174000	LMTK2	Receptor
7	99065001	99067000	SMURF1;SMURF1	Proteolysis;Proteolysis
7	99466001	99467000	ATP5MF-PTCD1;CPSF4;ATP5MF;TRW-CCA5-1;ZNF789	Translation;Metabolism
7	100045001	100046000	ZKSCAN1;ZSCAN21	Transcription
7	100115001	100116000	AP4M1;TAF6;CNPY4	Transport;Transcription
7	100316001	100317000	SPDYE3;PMS2P1	Transcription
7	100399001	100400000	PILRA;ZCWPW1	Immune
7	100580001	100581000	IRS3P;SAP25;LRCH4;ZASP;FBXO24;PCOLCE-AS1	Epigenetic
7	100640001	100641000	TFR2;ACTL6B	Protease;Cytoskeleton
7	100665001	100666000	ACTL6B;LOC105375429;GNB2	Cytoskeleton;Signaling
7	100812001	100813000	EPHB4;RN7SL750P	Receptor
7	100870001	100871000	SLC12A9;TRIP6;MIR6875;SRRT	Transport;Cytoskeleton
7	100957001	100959000	LOC105375431;MUC3A;LOC105375431;MUC3A	Cytoskeleton;Cytoskeleton
7	101092001	101094000	TRIM56;TRIM56	;
7	101102001	101103000	TRIM56	
7	101127001	101129000	SERPINE1;SERPINE1	Protease; Proteolysis;Protease; Proteolysis
7	101430001	101431000	COL26A1	
7	101504001	101505000	COL26A1	
7	101554001	101555000	COL26A1;LINC01007	
7	102066001	102067000	CUX1	Development
7	102165001	102166000	CUX1	Development
7	102559001	102560000	POLR2J3;SPDYE2	Transcription
7	102612001	102613000	RASA4	Signaling
7	103215001	103216000	LOC105375434;DPY19L2P2	
7	103464001	103465000	LOC101927870;RELN	Extracellular Matrix
7	105107001	105108000	KMT2E;SRPK2	Signaling
7	105385001	105386000	SRPK2	Signaling
7	105661001	105662000	ATXN7L1	
7	106814001	106815000	LINC02577	
7	106830001	106831000	LINC02577	
7	108298001	108299000	NRCAM	
7	110005001	110006000	RPL3P8	
7	110696001	110697000	IMMP2L	Protease
7	114329001	114330000	FOXP2	
7	114929001	114930000	MDFIC	
7	115003001	115004000	MDFIC	
7	116626001	116627000	COMETT	
7	116792001	116794000	MET;MET	Receptor;Receptor
7	116949001	116950000	LOC105375465;RNA5SP239;ST7-AS1;ST7;ST7-OT4	
7	117813001	117814000	CTTNBP2	
7	120274001	120275000	KCND2	Transport
7	120581001	120582000	KCND2	Transport
7	121316001	121318000	WNT16;WNT16	Signaling;Signaling
7	121739001	121740000	RN7SKP277	
7	122671001	122672000	CADPS2;RPS26P31	Transport
7	122837001	122838000	CADPS2	Transport
7	124985001	124986000	POT1-AS1	
7	126505001	126506000	GRM8;LOC101928357;LOC100422325	Signaling
7	126608001	126609000	GRM8	Signaling
7	126615001	126616000	GRM8	Signaling
7	127341001	127342000	ZNF800	
7	127592001	127593000	GCC1;ARF5;LOC105375489;FSCN3	Signaling;Cytoskeleton
7	127728001	127729000	SND1	
7	128066001	128067000	SND1	
7	128256001	128257000	LEP	Hormone
7	129182001	129184000	SMO;SMO	Receptor;Receptor
7	129302001	129303000	AHCYL2	Metabolism
7	129324001	129325000	AHCYL2	Metabolism
7	129396001	129397000	AHCYL2;RNU7-16P	Metabolism
7	130103001	130104000	KLHDC10	
7	130485001	130486000	MESTIT1;MEST	Protease
7	130678001	130679000	COPG2;TSGA13	Transport
7	130682001	130683000	TSGA13	

7	130799001	130800000	LOC105375508;LOC105375509	
7	131156001	131157000	MKLN1	
7	132667001	132668000	FLJ40288	
7	132727001	132728000	FLJ40288	
7	133043001	133044000	CHCHD3;LOC729998;MIR3654	
7	133251001	133252000	EXOC4	Transport
7	133906001	133907000	EXOC4	Transport
7	134456001	134457000	AKR1B1	Metabolism
7	134955001	134956000	CALD1	Cytoskeleton
7	135167001	135168000	CYREN;TMEM140	
7	136972001	136973000	CHRM2;LOC349160	Signaling
7	138083001	138084000	AKR1D1;RN7SKP223	Metabolism
7	138175001	138176000	LOC107986749	
7	138833001	138835000	KIAA1549;KIAA1549	;
7	138953001	138954000	KIAA1549	
7	139077001	139078000	ZC3HAV1	
7	139288001	139289000	UBN2	Cytoskeleton
7	139465001	139467000	KLRG2;KLRG2	Receptor;Receptor
7	139559001	139560000	HIPK2	
7	139620001	139622000	HIPK2;LOC105375530;HIPK2;LOC105375530	;
7	139845001	139846000	TBXAS1	Metabolism
7	140323001	140325000	SLC37A3;SLC37A3	Transport;Transport
7	140436001	140437000	RAB19;LOC642355	
7	140633001	140634000	DENND2A;LOC105375535	
7	141126001	141127000	TMEM178B	
7	142829001	142830000	LOC105375541	
7	142887001	142888000	TRPV6	Transport
7	143310001	143311000	CASP2;HINT1P1;CLCN1	Protease;Transport
7	143386001	143388000	FAM131B;LOC100507507;ZYX;MIR6892;EPHA1;FAM131B;LOC100507507;ZYX;MIR6892;EPHA1	Cytoskeleton;Receptor;Cytoskeleton;Receptor
7	143539001	143540000	RPL26P22;PAICSP5	
7	143788001	143790000	PAICSP6;RPL26P24;PAICSP6;RPL26P24	;
7	144189001	144190000	CTAGE4;ARHGEF35;ARHGEF35-AS1;LOC112267987	Transport
7	144259001	144260000	ARHGEF35-AS1;OR2A1-AS1;OR2A20P;OR2A7;ARHGEF34P;CTAGE8	Transport
7	147093001	147095000	CNTNAP2;CNTNAP2-AS1;CNTNAP2;CNTNAP2-AS1	;
7	148229001	148230000	CNTNAP2	
7	148311001	148313000	CNTNAP2;CNTNAP2	;
7	148331001	148332000	CNTNAP2	
7	148643001	148644000	LOC643438;LOC100301516	
7	148937001	148938000	RNY5	
7	149872001	149873000	ZNF862;ATP6V0E2-AS1;ATP6V0E2;ACTR3C	Metabolism;Cytoskeleton
7	150035001	150036000	ACTR3C;LOC112268011;LOC100134040	Cytoskeleton
7	150371001	150372000	REPIN1-AS1;REPIN1;ZNF775;ZNF775-AS1	Transcription
7	151075001	151076000	SLC4A2;FASTK;TMUB1;AGAP3	Transport
7	151099001	151100000	AGAP3;LOC107986862	
7	151122001	151124000	AGAP3;AGAP3	;
7	151170001	151171000	GBX1;ASB10	Development;Cytoskeleton
7	151842001	151843000	PRKAG2	Signaling
7	151990001	151991000	GALNTL5	Golgi
7	152532001	152533000	LOC105375574	
7	152871001	152873000	ACTR3B;ACTR3B	Cytoskeleton;Cytoskeleton
7	152908001	152909000	ACTR3B	Cytoskeleton
7	153073001	153074000	ACTR3B	Cytoskeleton
7	153401001	153402000	LOC105375577;LINC01287	
7	154017001	154018000	DPP6	Protease
7	154375001	154376000	DPP6	Protease
7	154570001	154571000	DPP6	Protease
7	154830001	154831000	DPP6;LOC105375580	Protease
7	155397001	155398000	LOC100286906	
7	155779001	155780000	RBM33	
7	155819001	155820000	SHH	
7	155963001	155964000	LOC389602	
7	156148001	156149000	LOC105375603;LOC105375602	
7	156933001	156934000	LOC105375604;LOC105375605	
7	156961001	156962000	NOM1	Translation
7	157166001	157167000	UBE3C	Proteolysis

7	157400001	157401000	DNAJB6	
7	157641001	157642000	PTPRN2	Signaling
7	157757001	157758000	PTPRN2	Signaling
7	157771001	157773000	PTPRN2;PTPRN2	Signaling;Signaling
7	157811001	157812000	PTPRN2	Signaling
7	157822001	157823000	PTPRN2	Signaling
7	157881001	157882000	PTPRN2	Signaling
7	157887001	157888000	PTPRN2	Signaling
7	158019001	158020000	PTPRN2	Signaling
7	158058001	158059000	PTPRN2	Signaling
7	158084001	158085000	PTPRN2	Signaling
7	158147001	158148000	PTPRN2	Signaling
7	158149001	158150000	PTPRN2	Signaling
7	158236001	158237000	PTPRN2	Signaling
7	158314001	158315000	PTPRN2	Signaling
7	158381001	158382000	PTPRN2	Signaling
7	158756001	158757000	ESYT2	
7	158782001	158783000	ESYT2;LOC105379752	
7	158916001	158917000	DYNC2I1	
7	158929001	158931000	DYNC2I1;DYNC2I1	;
7	158945001	158946000	DYNC2I1	
8	96001	97000	LOC101927506	
8	899001	900000	DLGAP2;LOC112268020	Cytoskeleton
8	986001	987000	DLGAP2	Cytoskeleton
8	992001	993000	DLGAP2	Cytoskeleton
8	1007001	1008000	DLGAP2	Cytoskeleton
8	1039001	1040000	DLGAP2	Cytoskeleton
8	1049001	1050000	DLGAP2	Cytoskeleton
8	1093001	1094000	DLGAP2	Cytoskeleton
8	1103001	1104000	DLGAP2	Cytoskeleton
8	1250001	1251000	DLGAP2	Cytoskeleton
8	1375001	1376000	DLGAP2;LOC105379585	Cytoskeleton
8	1441001	1442000	DLGAP2;LOC105377775;LOC105379586	Cytoskeleton
8	1471001	1472000	DLGAP2	Cytoskeleton
8	1641001	1642000	DLGAP2	Cytoskeleton
8	1751001	1752000	LOC105377778;CLN8;CLN8-AS1	
8	2119001	2120000	MYOM2	
8	2274001	2275000	LOC105377783	
8	2812001	2813000	LOC105377785	
8	2862001	2863000	LOC105377785	
8	2879001	2880000	LOC105377785	
8	2986001	2987000	LOC105377785;CSMD1	
8	3206001	3207000	CSMD1	
8	4284001	4285000	CSMD1	
8	4364001	4365000	CSMD1	
8	6692001	6693000	MCPH1-AS1	
8	6851001	6852000	GS1-24F4.2	
8	7091001	7092000	LOC107986875	
8	7211001	7212000	LOC101928095	
8	7278001	7280000	FAM90A3P;FAM90A4P;FAM90A13P;FAM90A5P;FAM90A4P;FAM90A13P;FAM90A5P	;
8	7518001	7519000	DEFB107B;LOC105377801;LOC101927997	
8	7575001	7576000	LOC105377800;FAM90A21P;FAM90A22P;FAM90A23P	
8	7714001	7716000	OR7E154P;LOC105379215;FAM90A14P;FAM90A18P;OR7E154P;LOC105379215;FAM90A14P;FAM90A18P	;
8	7737001	7738000	FAM90A16P;FAM90A8P;FAM90A17P	
8	7739001	7740000	FAM90A16P;FAM90A8P;FAM90A17P	
8	7852001	7853000	SPAG11A	
8	7974001	7975000	FAM66E;LOC392187;USP17L8;USP17L3	Protease
8	8154001	8155000	FAM85B;LOC112268014;ENPP7P1	
8	8328001	8329000	PRAG1	Signaling
8	8833001	8834000	MFHAS1	Cytoskeleton
8	10146001	10148000	MSRA;MSRA	Metabolism;Metabolism
8	10480001	10481000	LINCR-0001;PRSS52P;RNU6-729P;LOC112268021	
8	10866001	10867000	LOC101929248;LOC112268022	
8	11109001	11110000	XKR6	
8	11443001	11444000	FAM167A-AS1;FAM167A	

8	11633001	11634000	LOC105379242	
8	11647001	11648000	LOC105379242	
8	11724001	11726000	GATA4;GATA4	Transcription;Transcription
8	11729001	11730000	GATA4	Transcription
8	12377001	12379000	FAM66A;LOC100421094;LOC649352;FAM66A;LOC100421094;LOC649352	;
8	12439001	12440000	FAM86B2;LOC100506990;DEFB109E;ENPP7P6	
8	12450001	12451000	LOC100506990;DEFB109E;ENPP7P6	
8	12612001	12613000	LOC729732	
8	12744001	12745000	LONRF1	Proteolysis
8	12802001	12803000	LOC340357;LINC00681	
8	12806001	12807000	LOC340357;LINC00681	
8	12808001	12809000	LOC340357;LINC00681	
8	12818001	12819000	LOC340357;LINC00681	
8	14222001	14223000	SGCZ	Cytoskeleton
8	14264001	14265000	SGCZ	Cytoskeleton
8	14805001	14807000	SGCZ;SGCZ	Cytoskeleton;Cytoskeleton
8	16179001	16180000	MSR1	Protease
8	17681001	17682000	MTUS1;MIR548V	
8	17884001	17885000	FGL1	Signaling
8	18206001	18207000	NAT1;MTND4LP26	Metabolism
8	18555001	18556000	PSD3	Transcription
8	22200001	22201000	BMP1	Protease
8	22221001	22222000	BMP1;PHYHIP	Protease
8	22256001	22257000	POLR3D;PIWIL2-DT	Transcription
8	22307001	22308000	PIWIL2	Translation
8	22677001	22678000	BIN3;LOC107986924;EGR3	Transcription
8	22738001	22739000	PEBP4;LOC107986925	Protease; Proteolysis
8	22759001	22760000	PEBP4;LOC107986925	Protease; Proteolysis
8	22904001	22905000	PEBP4	Protease; Proteolysis
8	22948001	22949000	PEBP4;LOC107984124	Protease; Proteolysis
8	23294001	23295000	R3HCC1;LOXL2	Metabolism
8	23319001	23320000	LOXL2	Metabolism
8	23484001	23486000	LOC646708;LOC105379327;LOC646708;LOC105379327	;
8	24318001	24319000	ADAM28;LOC101929294	Protease
8	25796001	25797000	LOC107986933	
8	26116001	26117000	LOC105379335	
8	26441001	26442000	DNAJB6P2;LOC105379337	
8	26564001	26565000	DPYSL2	Metabolism
8	27430001	27431000	PTK2B;MIR6842	
8	27528001	27529000	EPHX2	Metabolism
8	27554001	27555000	EPHX2	Metabolism
8	28422001	28424000	RNU6-178P;FBXO16;RNU6-178P;FBXO16	;
8	28484001	28485000	FBXO16;FZD3	Receptor
8	28847001	28848000	INTS9	
8	28934001	28935000	HMBOX1	
8	30552001	30553000	RBPMS;LOC112268024	Translation
8	30671001	30672000	GSR	Metabolism
8	31077001	31078000	WRN	Epigenetic
8	32456001	32457000	NRG1	Growth Factors
8	32531001	32532000	NRG1	Growth Factors
8	32591001	32592000	NRG1	Growth Factors
8	34219001	34220000	LOC105379365	
8	37098001	37099000	SMARCE1P4	
8	38903001	38904000	PLEKHA2	
8	40793001	40794000	ZMAT4	
8	40909001	40910000	LOC105379389	
8	41291001	41292000	SFRP1;RNU6-895P	Receptor
8	41416001	41417000	SNORD65B	
8	41485001	41486000	GOLGA7	
8	43117001	43118000	POMK	
8	43149001	43150000	HGSNAT;RNY5P6	
8	43491001	43492000	LOC105379397	
8	43546001	43547000	CYP4F44P	
8	47143001	47144000	LOC107986885	
8	47310001	47311000	SPIDR	Transcription
8	47421001	47422000	SPIDR	Transcription

8	47727001	47728000	SPIDR;CEBPD	Transcription;Transcription
8	47955001	47956000	PRKDC;MCM4	Signaling;Transcription
8	48072001	48073000	UBE2V2	Proteolysis
8	49976001	49977000	SNTG1	
8	50480001	50481000	SNTG1;LOC100422267	
8	51709001	51710000	PXDNL	Metabolism
8	52306001	52307000	ST18;RPL34P17	Transcription
8	52784001	52785000	LOC105375835	
8	53819001	53820000	ATP6V1H	Metabolism
8	54156001	54157000	MRPL15;RNU6ATAC32P	Translation
8	55689001	55691000	LOC105375845;LOC105375846;LOC105375845;LOC105375846	;
8	55936001	55937000	LYN	
8	56654001	56655000	LOC105375851	
8	57220001	57221000	LINC01606	
8	58423001	58424000	UBXN2B;LOC100421822	Signaling
8	58502001	58503000	CYP7A1;LOC110596867	Metabolism
8	58808001	58809000	TOX;RNU4-50P	
8	59762001	59763000	LOC107986889	
8	61031001	61033000	CLVS1;CLVS1	Transport;Transport
8	62459001	62460000	NKAIN3	
8	63162001	63163000	YTHDF3-DT;YTHDF3	
8	64135001	64136000	LINC01414	
8	65635001	65636000	ARMC1;MTFR1	
8	65669001	65670000	MTFR1	
8	66916001	66917000	MCMDC2;SNHG6;SNORD87	Transcription
8	69157001	69158000	TRE-CTC14-1	
8	69554001	69555000	SULF1	Metabolism
8	69579001	69580000	SULF1	Metabolism
8	70365001	70366000	NCOA2;RNY3P14	Epigenetic
8	70513001	70514000	RPL13P11	
8	73446001	73447000	STAU2-AS1;STAU2	
8	73669001	73670000	STAU2;LOC105375902	
8	74039001	74040000	LY96;RNU6-1300P	Cytoskeleton
8	79594001	79595000	LOC105375916	
8	79721001	79722000	LOC107986893	
8	80106001	80107000	TPD52	
8	80979001	80980000	PAG1	
8	81933001	81934000	LINC02235	
8	84740001	84741000	RALYL	
8	85180001	85181000	E2F5-DT;E2F5	Transcription
8	85494001	85495000	LOC105375937	
8	85724001	85725000	REXO1L12P	
8	85728001	85729000	LOC101929601;REXO1L11P	
8	85734001	85735000	LOC101929601;REXO1L11P;LOC101929627	
8	85760001	85762000	REXO1L9P;REXO1L9P	;
8	85768001	85769000	REXO1L9P;REXO1L2P	
8	85774001	85775000	REXO1L2P	
8	85808001	85809000	REXO1L4P;REXO1L5P	
8	85814001	85815000	REXO1L5P	
8	85817001	85818000	REXO1L5P;REXO1L6P	
8	85827001	85829000	REXO1L6P;REXO1L6P	;
8	86133001	86134000	ATP6V0D2	Metabolism
8	86894001	86895000	CNBD1	
8	87620001	87621000	LOC105375626	
8	89748001	89749000	RIPK2-DT;RIPK2	Signaling
8	90761001	90762000	LOC105375633	
8	90947001	90948000	NECAB1;LOC105375634	
8	91029001	91030000	PIP4P2	
8	93745001	93746000	RBM12B;RBM12B-AS1;RBM12B-DT;TMEM67	Translation
8	94120001	94121000	CDH17	Cytoskeleton
8	94195001	94196000	CDH17;RPL6P23	Cytoskeleton
8	94455001	94456000	RAD54B	
8	95253001	95254000	CFAP418	
8	95301001	95302000	CFAP418-AS1	
8	97841001	97842000	LAPTM4B	Transport
8	98406001	98407000	LOC100131849	

8	99007001	99008000	VPS13B-DT;LOC107986871;VPS13B	Transport
8	99163001	99164000	VPS13B	Transport
8	99614001	99615000	VPS13B;RN7SL350P	Transport
8	99692001	99693000	VPS13B;LETM1P3	Transport
8	100064001	100065000	RGS22	
8	100595001	100596000	SNX31	Cytoskeleton
8	100674001	100675000	LOC105375672;RNU6-1092P	
8	100712001	100713000	PABPC1;MIR7705	
8	100831001	100832000	RNU4-83P	
8	101131001	101132000	LOC105375674;LOC105375675;RN7SKP249	
8	102893001	102894000	MAILR	
8	104211001	104212000	RIMS2	Transport
8	104362001	104363000	DCSTAMP	
8	106769001	106771000	ABRA;ABRA	;
8	106783001	106784000	ABRA	
8	108420001	108421000	LOC105375704	
8	112536001	112537000	CSMD3;RPL30P16	
8	116967001	116968000	SLC30A8	Transport
8	119896001	119897000	DEPTOR;RNA5SP277	Transcription
8	120220001	120221000	COL14A1	Extracellular Matrix
8	123216001	123218000	FAM83A;MIR4663;C8orf76;ZHX1-C8orf76;FAM83A;MIR4663;C8orf76;ZHX1-C8orf76	Development;Development
8	124301001	124302000	LOC112268031;TMEM65	
8	125614001	125615000	LOC105375746	
8	125930001	125931000	LINC00861	
8	127432001	127433000	CASC8	
8	131910001	131911000	EFR3A	
8	132455001	132456000	KCNQ3	Transport
8	132617001	132618000	DNAAF11	
8	133317001	133318000	RPL32P20	
8	136794001	136795000	LINC02055	
8	138316001	138317000	FAM135B	
8	138772001	138773000	COL22A1	Extracellular Matrix
8	138882001	138883000	COL22A1	Extracellular Matrix
8	139751001	139752000	TRAPPC9	
8	140077001	140078000	TRAPPC9	
8	140167001	140168000	TRAPPC9	
8	140195001	140196000	TRAPPC9	
8	140275001	140276000	TRAPPC9;LOC105375779	
8	140655001	140656000	PTK2	
8	140987001	140988000	PTK2	
8	141124001	141125000	DENND3-AS1;DENND3	
8	141193001	141194000	DENND3;LOC105375785	
8	141237001	141238000	SLC45A4	Transport
8	141257001	141258000	SLC45A4;LOC105375787	Transport
8	141414001	141415000	PTP4A3	Signaling
8	141524001	141525000	LOC105375791	
8	142285001	142286000	TSNARE1	Transcription
8	142290001	142291000	TSNARE1	Transcription
8	142346001	142347000	TSNARE1	Transcription
8	142408001	142409000	TSNARE1	Transcription
8	142501001	142502000	ADGRB1	Signaling
8	142837001	142838000	GML	
8	142979001	142980000	LY6E-DT	
8	143263001	143264000	ZFP41;GLI4	Transcription
8	143316001	143317000	TOP1MT	Transcription
8	143423001	143424000	LOC107986984;MAFA-AS1;MAFA	Transcription
8	143493001	143494000	ZC3H3	
8	143852001	143854000	EPPK1;EPPK1	Cytoskeleton;Cytoskeleton
8	144117001	144118000	MAF1;WDR97	Transcription
8	144275001	144276000	BOP1;SCX	Translation;Transcription
8	144277001	144278000	BOP1;SCX	Translation;Transcription
8	144294001	144295000	BOP1;HSF1	Translation;Transcription
8	144307001	144309000	HSF1;DGAT1;MIR6848;HSF1;DGAT1;MIR6848	Transcription;Metabolism;Transcription;Metabolism
8	144325001	144326000	DGAT1;MIR6848;SCRT1	Metabolism;Transcription
8	144373001	144374000	LOC101928902;ADCK5	Transport
8	144475001	144477000	CYHR1;KIFC2;FOXH1;PPP1R16A;KIFC2;FOXH1;PPP1R16A	Cytoskeleton;Signaling;Cytoskeleton;Signaling

8	144513001	144514000	LOC101928953;GPT;MFSD3;RECQL4;LRRC14;LRRC24	Metabolism;Transport;Epigenetic;Receptor
8	144601001	144602000	ARHGAP39	
9	476001	477000	LOC112268042;KANK1;RPL12P25	Cytoskeleton
9	518001	519000	KANK1	Cytoskeleton
9	851001	852000	DMRT1	Transcription
9	869001	870000	DMRT1	Transcription
9	1384001	1385000	LOC102723803	
9	1401001	1402000	LOC102723803	
9	1760001	1761000	LOC105375951	
9	3811001	3812000	LOC105375962	
9	4058001	4059000	GLIS3	Transcription
9	4117001	4118000	GLIS3	Transcription
9	4285001	4286000	GLIS3	Transcription
9	5144001	5145000	INSL6	Hormone
9	5894001	5895000	KIAA2026;MLANA	
9	6474001	6475000	UHRF2	Proteolysis
9	6594001	6595000	GLDC	Metabolism
9	6639001	6640000	GLDC;RPL23AP57	Metabolism
9	7475001	7476000	RPL4P5	
9	7553001	7554000	PPIAP33	
9	8332001	8333000	PTPRD	Signaling
9	10021001	10022000	PTPRD	Signaling
9	10414001	10415000	PTPRD	Signaling
9	10554001	10555000	PTPRD	Signaling
9	13431001	13432000	LINC01235;LOC105375977	
9	15442001	15443000	SNAPC3;RNU6-319P	Transcription
9	15882001	15883000	CCDC171	
9	16821001	16822000	BNC2	Transcription
9	16845001	16846000	BNC2	Transcription
9	18915001	18916000	ADAMTSL1	Protease
9	20308001	20309000	SLC24A2	Transport
9	20337001	20338000	SMNP;MLLT3	Transcription
9	20692001	20693000	FOCAD;FOCAD-AS1	
9	21392001	21393000	IFNA2;IFNA11P	Immune
9	25670001	25671000	TUSC1	
9	29175001	29176000	LINGO2	Receptor
9	33393001	33395000	AQP7;LOC105376020;AQP7;LOC105376020	Transport;Transport
9	34002001	34003000	UBAP2	
9	34037001	34038000	UBAP2	
9	34184001	34185000	UBAP1;LOC114224;RPL35AP2	
9	34357001	34358000	MYORG	Metabolism
9	34937001	34938000	PHF24	Signaling
9	35277001	35278000	UNC13B	
9	35439001	35440000	ATP8B5P;ZFAND6P1	
9	35557001	35558000	RUSC2;FAM166B	
9	35594001	35595000	RPS29P17	
9	35802001	35803000	NPR2;SPAG8;HINT2	Signaling;Signaling
9	35957001	35958000	OR2S2	Receptor
9	36299001	36300000	LOC102724322;HMGB3P24	
9	37653001	37654000	FRMPD1;RN7SKP171	
9	38497001	38498000	LOC105376042;VN1R48P	
9	38842001	38843000	LOC105376043	
9	38962001	38963000	LOC101927042	
9	39041001	39042000	VN2R3P	
9	39470001	39471000	ZNF658B	Transcription
9	40270001	40272000	ANKRD20A2P;ANKRD20A2P	;
9	40929001	40930000	MIR1299	
9	40952001	40953000	LOC107986997	
9	41073001	41074000	PGM5P2;LOC105376058	
9	41570001	41571000	RN7SL565P	
9	42215001	42216000	FAM74A7;LOC112268036	
9	42827001	42828000	MEP1AP4;LOC102724818	
9	42900001	42901000	ANKRD20A7P;LOC112268044	
9	61856001	61857000	FAM27C;FAM27E4;LOC105379807;LOC102723678	
9	62799001	62800000	LINC01410;RNA5SP283	
9	62843001	62844000	LOC100132249;PTGER4P2-CDK2AP2P2;PTGER4P2;CDK2AP2P2	

9	63388001	63389000	LOC100996643;BMS1P10	
9	63823001	63824000	LINC00537;RNA5SP284;DUX4L50;MIR4477B;FRG1JP	
9	65383001	65386000	LOC105379444;LOC105379444;LOC107987069;LOC105379444;LOC107987069	;;
9	65407001	65408000	LOC105379445	
9	67277001	67278000	CNTNAP3P2	
9	67834001	67835000	LOC101928608	
9	70519001	70520000	KLF9-DT;TRPM3	Transport
9	72336001	72337000	LINC01504	
9	74566001	74567000	RORB	Transcription
9	74876001	74877000	TRPM6	Transport
9	76008001	76009000	PCSK5	Protease
9	76175001	76176000	PCSK5	Protease
9	76440001	76441000	GCNT1	Golgi
9	77036001	77037000	LOC105376096;ATP5MFP3	
9	77168001	77169000	VPS13A-AS1;VPS13A	Transport
9	77462001	77463000	GNA14;GNA14-AS1	Signaling
9	77526001	77527000	GNA14;GNA14-AS1	Signaling
9	77625001	77626000	GNA14;RNU6-1303P	Signaling
9	77863001	77864000	GNAQ	Signaling
9	78100001	78101000	LOC107987031	
9	78185001	78186000	ASS1P3;RPL21P84	
9	78308001	78309000	PSAT1	Metabolism
9	81930001	81931000	LOC105376108;SPATA31D4	
9	82063001	82064000	LOC105376107;SPATA31B1P	
9	82717001	82718000	LOC107987087	
9	84832001	84833000	NTRK2	Receptor
9	85424001	85425000	LOC105376121	
9	85688001	85689000	AGTPBP1	Protease
9	85826001	85827000	LOC389765	
9	85850001	85851000	LOC389765	
9	87501001	87502000	DAPK1	Signaling
9	87659001	87660000	DAPK1	Signaling
9	87663001	87664000	DAPK1	Signaling
9	88968001	88969000	PCNPP2	
9	88974001	88975000	PCNPP2	
9	89059001	89060000	SHC3	Cytoskeleton
9	89369001	89371000	SECISBP2;SEMA4D;SECISBP2;SEMA4D	Signaling;Signaling
9	89429001	89430000	SEMA4D	Signaling
9	90842001	90843000	SYK	
9	91711001	91712000	LOC105376147	
9	91918001	91919000	ROR2	Receptor
9	92176001	92177000	BEND3P2;PRSS47	Protease
9	92959001	92960000	FGD3	Transcription
9	93263001	93264000	WNK2	Signaling
9	93677001	93678000	PHF2	
9	93946001	93947000	BARX1;BARX1-DT	Development
9	94262001	94263000	LINC02603;ZNF169	Transcription
9	94556001	94557000	PCAT7;FBP2	Metabolism
9	94735001	94736000	AOPEP	Protease
9	94925001	94926000	AOPEP;LOC101928119	Protease
9	94927001	94928000	AOPEP;LOC101928119	Protease
9	95144001	95145000	AOPEP;FANCC;RNA5SP288	Protease
9	95182001	95183000	FANCC	
9	95238001	95239000	FANCC	
9	96111001	96112000	LOC158435;LOC158434	
9	96267001	96268000	HSD17B3	
9	96467001	96468000	HABP4	Metabolism
9	96606001	96607000	CDC14B;LOC105376163	Signaling
9	97796001	97797000	PTCSC2	
9	97916001	97917000	TRMO;HEMGN	
9	98181001	98182000	CORO2A	Cytoskeleton
9	98447001	98448000	GABBR2	Signaling
9	98736001	98738000	ANKS6;ANKS6	Metabolism;Metabolism
9	99040001	99041000	COL15A1	Extracellular Matrix
9	99239001	99240000	SEC61B	Transport
9	99424001	99425000	LOC107987011	

9	100505001	100506000	MSANTD3-TMEFF1;TMEFF1	
9	100855001	100856000	LOC105376179	
9	101567001	101568000	RNF20;GRIN3A	Receptor
9	104633001	104634000	OR13I1P	
9	104801001	104802000	ABCA1	Transport
9	106683001	106684000	LINC01505;RN7SKP77	
9	108247001	108248000	LOC105376214;LOC105376212	
9	108371001	108372000	LOC105376214	
9	108616001	108618000	RPL36P14;RPL36P14	;
9	109985001	109986000	PALM2AKAP2	
9	110041001	110042000	PALM2AKAP2	
9	110677001	110678000	MUSK	Receptor
9	111403001	111404000	ECPAS;RNA5SP294	Cytoskeleton
9	111520001	111521000	ZNF483	
9	112423001	112424000	HSDL2	Metabolism
9	112481001	112482000	HSDL2;HSDL2-AS1;KIAA1958	Metabolism
9	113542001	113543000	RGS3	
9	114222001	114223000	COL27A1	Extracellular Matrix
9	114360001	114361000	AKNA	
9	115096001	115097000	TNC	Extracellular Matrix
9	115124001	115125000	TNC;LOC101928748	Extracellular Matrix
9	115347001	115348000	DELEC1;LOC105376232	
9	117642001	117643000	LOC101928797	
9	119170001	119171000	BRINP1	
9	119957001	119958000	LOC107987122	
9	120092001	120093000	LOC107987124	
9	120495001	120496000	CDK5RAP2	
9	120746001	120747000	FBXW2	
9	120841001	120842000	PSMD5;CUTALP	Protease
9	121076001	121078000	C5;CNTRL;C5;CNTRL	Protease; Proteolysis;Protease; Proteolysis
9	121493001	121494000	GGTA1;RN7SL187P	
9	122136001	122137000	NDUFA8	Metabolism
9	122158001	122159000	NDUFA8;MORN5	Metabolism
9	122452001	122453000	OR1J2	Receptor
9	122800001	122801000	OR1K1	
9	123331001	123332000	LOC105376265	
9	124088001	124089000	LOC107987037	
9	124438001	124439000	LOC105376270	
9	124465001	124466000	ADGRD2	Signaling
9	124742001	124743000	NR6A1	
9	124773001	124774000	NR6A1;LOC107987126;OLFML2A	Development
9	124885001	124886000	ARPC5L;GOLGA1;RNU4-82P	Cytoskeleton
9	125740001	125741000	LOC51145;PBX3	Development
9	126403001	126404000	MVB12B;NRON	
9	126615001	126616000	LOC105376277;LMX1B	Development
9	126916001	126917000	RALGPS1	Transcription
9	127173001	127175000	RALGPS1;LOC105376278;RALGPS1;LOC105376278	Transcription;Transcription
9	127433001	127434000	ZNF79	Transcription
9	127768001	127769000	SH2D3C;LOC107987132	
9	128251001	128252000	DNM1;MIR199B;MIR3154;GOLGA2	Transport
9	128359001	128360000	SLC27A4	Transport
9	128596001	128597000	SPTAN1	
9	128632001	128633000	SPTAN1;DYNC2I2	
9	128660001	128662000	DYNC2I2;VTI1BP4;LOC105376287;HMGA1P4;DYNC2I2;VTI1BP4;LOC105376287;HMGA1P4	;
9	129004001	129005000	NUP188;LOC101929314;SH3GLB2	
9	129278001	129279000	LOC101929331	
9	129319001	129320000	LOC107987133;LINC02913	
9	129378001	129379000	LOC107987038;RN7SL159P	
9	129446001	129447000	LOC105376291	
9	129576001	129577000	LOC105376292	
9	129647001	129649000	ASB6;ASB6	Cytoskeleton;Cytoskeleton
9	129672001	129673000	PRRX2	
9	130169001	130170000	NCS1	
9	130454001	130455000	ASS1	Metabolism
9	130621001	130622000	FUBP3;MIR6856	Metabolism
9	130877001	130879000	ABL1;ABL1	;

9	131886001	131887000	MED27	Transcription
9	132362001	132363000	SETX	
9	132617001	132618000	DDX31	
9	132872001	132873000	AK8;SPACA9	Signaling
9	133235001	133236000	LCN1P1	Transport
9	133310001	133311000	LCN1P2	
9	133626001	133627000	DBH	Metabolism
9	133751001	133752000	VAV2	
9	133786001	133787000	VAV2	
9	133795001	133796000	VAV2	
9	133860001	133861000	VAV2	
9	133878001	133879000	VAV2	
9	134059001	134060000	BRD3;LOC100130548	
9	134073001	134074000	BRD3;ARF4P1	
9	134389001	134390000	RXRA;MIR4669	Transcription
9	134505001	134506000	LOC100506532	
9	134554001	134555000	LOC100506532	
9	134716001	134717000	COL5A1	Extracellular Matrix
9	134846001	134847000	COL5A1;LOC101448202;MIR3689C;MIR3689A;MIR3689D1;MIR3689B;MIR3689D2;MIR3689E;MIR3689F	Extracellular Matrix
9	134883001	134884000	FCN2	Signaling
9	135188001	135189000	LOC401557	
9	135244001	135245000	LOC107987138	
9	135356001	135357000	LINC02907	
9	135634001	135635000	GLT6D1	Golgi
9	135774001	135775000	KCNT1;LOC107987139	Transport
9	135837001	135838000	CAMSAP1	
9	136042001	136044000	NACC2;NACC2	;
9	136293001	136294000	CCDC187	Cytoskeleton
9	136397001	136398000	SNAPC4;ENTR1	
9	136736001	136737000	SNHG7;SNORA17B;SNORA17A;LCN10;LCN6;LOC100128593;MIR6722	Transport
9	136820001	136821000	RABL6;NCLP1;MIR4292	Signaling
9	136830001	136831000	RABL6;MIR4292	Signaling
9	136900001	136901000	TRAF2	Cytoskeleton
9	136903001	136904000	TRAF2	Cytoskeleton
9	137139001	137140000	GRIN1	Receptor
9	137192001	137193000	ANAPC2;SSNA1;TPRN	
9	137216001	137217000	NDOR1;RNF208;CYSRT1	Metabolism
9	137328001	137329000	EXD3	
9	137430001	137431000	EXD3;NOXA1;ENTPD8	Signaling;Signaling
9	137450001	137451000	ENTPD8;NSMF;MIR7114;PNPLA7	Signaling;Metabolism
9	137499001	137500000	PNPLA7	Metabolism
9	137522001	137525000	PNPLA7;PNPLA7;PNPLA7	Metabolism;Metabolism;Metabolism
9	138119001	138122000	CACNA1B;CACNA1B;CACNA1B	Transport;Transport;Transport
9	138140001	138142000	IL9RP1;TUBBP5;IL9RP1;TUBBP5	;
9	138232001	138233000	FAM157B	
10	284001	285000	DIP2C;RNA5SP298	
10	528001	529000	DIP2C	
10	634001	635000	DIP2C;LOC101930421;MIR5699	
10	830001	831000	LARP4B	Metabolism
10	878001	879000	LARP4B	Metabolism
10	888001	889000	LARP4B	Metabolism
10	1020001	1021000	GTPBP4;IDI2;IDI2-AS1	Metabolism
10	1169001	1170000	LOC105376344;LINC00200;ADARB2	Metabolism
10	1213001	1214000	ADARB2	Metabolism
10	1672001	1673000	ADARB2	Metabolism
10	3254001	3255000	LINC02668;LOC105376353	
10	3315001	3316000	LOC105376356;LOC105376360	
10	3356001	3357000	LOC105376360	
10	3428001	3429000	LOC105376360;LINC02669	
10	3519001	3520000	LOC105376360	
10	3993001	3994000	MIR6078	
10	4158001	4159000	LOC105376368	
10	5169001	5170000	AKR1C8P	
10	6152001	6153000	PFKFB3;RN7SKP78;MIR3155A;MIR3155B	Metabolism
10	6472001	6473000	PRKCQ	Signaling

10	6541001	6542000	PRKCQ	Signaling
10	7378001	7379000	SFMBT2	Epigenetic
10	7976001	7977000	TAF3	
10	10498001	10499000	CELF2	
10	10614001	10615000	CELF2	
10	10926001	10927000	CELF2;LOC105376405;LINC00710	
10	11583001	11584000	USP6NL	Signaling
10	12175001	12177000	SEC61A2;NUDT5;SEC61A2;NUDT5	Transport;Signaling;Transport;Signaling
10	12593001	12594000	CAMK1D	Signaling
10	12967001	12968000	CCDC3	
10	14067001	14068000	FRMD4A;LOC101928453	
10	16703001	16704000	RSU1	Cytoskeleton
10	17698001	17699000	STAM	Cytoskeleton
10	17742001	17743000	TMEM236	
10	18034001	18035000	SLC39A12	Transport
10	18176001	18177000	CACNB2	Transport
10	21094001	21095000	NEBL	
10	21317001	21318000	RNU6-15P	
10	21546001	21547000	MLLT10	Transcription
10	21911001	21912000	DNAJC1	Transcription
10	23148001	23149000	YWHAZP3	
10	23331001	23332000	C10orf67	
10	23550001	23551000	LOC105376454	
10	24270001	24271000	KIAA1217;MIR603	
10	24525001	24527000	KIAA1217;KIAA1217	;
10	24555001	24556000	KIAA1217	
10	24620001	24621000	ARHGAP21	
10	24948001	24949000	PRTFDC1;THNSL1	Golgi
10	25364001	25365000	GPR158	Signaling
10	26498001	26499000	APBB1IP	Cytoskeleton
10	26589001	26590000	LOC101929117;FAM238A	
10	26668001	26669000	SELENOOLP	
10	27238001	27239000	ACBD5;RNU7-12P;LRRC37A6P	Transport
10	28289001	28290000	MPP7;MIR8086	Cytoskeleton
10	28754001	28755000	LINC01517;RNU6-270P	
10	29518001	29519000	SVIL	Cytoskeleton
10	29987001	29988000	LOC107984218	
10	30317001	30318000	MTPAP	Metabolism
10	31033001	31034000	ZNF438;LOC105376481	Transcription
10	31183001	31184000	LINC02664	
10	32449001	32450000	LOC101929431;CCDC7	
10	33000001	33001000	ITGB1-DT	
10	33301001	33302000	NRP1	
10	34264001	34265000	PARD3	
10	34355001	34356000	PARD3	
10	34936001	34937000	RNU6-847P;RNU6-193P	
10	35575001	35576000	CCNY;LOC107984221	
10	35582001	35583000	CCNY;LOC107984221	
10	38443001	38444000	LOC101929540;LINC00999	
10	38483001	38484000	LOC107984177	
10	38593001	38594000	ABCD1P2	
10	42150001	42151000	KSR1P1	
10	43798001	43801000	LOC105378275;HNRNPA3P1;LOC105378275;HNRNPA3P1;LOC105378275;HNRNPA3P1	;;
10	45234001	45235000	LOC105378283;CUBNP2	
10	45725001	45726000	FAM21FP;LOC107984227;WASHC2C	Cytoskeleton
10	46293001	46294000	LOC105378577;ANTXRL	Cytoskeleton
10	46943001	46944000	PTPN20	Signaling
10	47328001	47330000	GDF2;GDF2	Growth Factors;Growth Factors
10	47633001	47634000	ANXA8	Signaling
10	47754001	47755000	ANXA8;LOC102724593;LOC102724488	Signaling
10	48456001	48457000	ARHGAP22	
10	48758001	48759000	WDFY4	
10	49105001	49106000	VSTM4	Immune
10	49658001	49659000	CHAT	Metabolism
10	49741001	49743000	OGDHL;OGDHL	Metabolism;Metabolism
10	49822001	49823000	RPL21P89;PARG	Metabolism

10	50738001	50739000	ASAH2B	
10	51746001	51747000	PRKG1	Signaling
10	52069001	52070000	PRKG1	Signaling
10	53797001	53798000	PCDH15	Cytoskeleton
10	54641001	54642000	PCDH15;LOC105378311	Cytoskeleton
10	55738001	55739000	LOC100419872	
10	58738001	58739000	BICC1	Metabolism
10	59869001	59870000	CCDC6	
10	59882001	59883000	CCDC6	
10	60862001	60863000	RHOBTB1	Signaling
10	61860001	61861000	LINC02625	
10	64839001	64840000	LOC105378336	
10	65762001	65763000	LINC01515;LOC105378338	
10	66418001	66419000	CTNNA3	Cytoskeleton
10	68399001	68401000	RUFY2;RUFY2	;
10	68477001	68478000	DNA2;SLC25A16	Transport
10	68999001	69000000	KIFBP;MED28P1	
10	70071001	70072000	MACROH2A2	Epigenetic
10	70171001	70172000	SAR1A;CALM2P2	
10	71240001	71241000	UNC5B;LOC107987148;LOC112268061	Receptor
10	71688001	71689000	CDH23	Cytoskeleton
10	72023001	72024000	CHST3	Transport
10	72060001	72061000	SPOCK2	Extracellular Matrix
10	72217001	72218000	ASCC1;ANAPC16	
10	72381001	72382000	MICU1;RNU6-805P	Signaling
10	72435001	72436000	MICU1	Signaling
10	73638001	73639000	MYOZ1;SYNPO2L	Cytoskeleton;Cytoskeleton
10	73884001	73885000	CAMK2G	Signaling
10	76076001	76077000	LRMDA	
10	76475001	76476000	LRMDA	
10	77099001	77100000	KCNMA1	Transport
10	77134001	77135000	KCNMA1;KCNMA1-AS2	Transport
10	77138001	77139000	KCNMA1;KCNMA1-AS2	Transport
10	77361001	77362000	KCNMA1;KCNMA1-AS3	Transport
10	77460001	77461000	KCNMA1	Transport
10	77801001	77802000	DLG5	Cytoskeleton
10	77860001	77861000	DLG5	Cytoskeleton
10	78031001	78032000	POLR3A;RPS24	Transcription;Translation
10	79272001	79273000	ZMIZ1	
10	79378001	79379000	ZCCHC24	
10	80425001	80426000	PRXL2A	
10	82669001	82670000	NRG3	Growth Factors
10	82879001	82880000	NRG3	Growth Factors
10	84298001	84299000	LINC00858	
10	84454001	84455000	CCSER2;CACYBPP1	
10	84476001	84477000	CCSER2	
10	85862001	85863000	GRID1	Receptor
10	86121001	86122000	GRID1	Receptor
10	86332001	86333000	GRID1	Receptor
10	86540001	86542000	LOC105378408;LOC105378407;LOC105378408;LOC105378407	;
10	86811001	86812000	BMPR1A	Signaling
10	86902001	86903000	BMPR1A	Signaling
10	87251001	87252000	NUTM2A-AS1	
10	87484001	87485000	LOC112268063	
10	89286001	89287000	LOC105378419	
10	89404001	89405000	IFIT1;LOC107984251;IFIT5	
10	89933001	89935000	LINC01375;LINC01375	;
10	90800001	90801000	HTR7	Signaling
10	90808001	90809000	HTR7	Signaling
10	91168001	91169000	LOC105378429;PCGF5	Epigenetic
10	91222001	91223000	PCGF5	Epigenetic
10	91465001	91466000	HECTD2-AS1;HECTD2	Proteolysis
10	93416001	93417000	MYOF	Transport
10	94797001	94798000	CYP2C19	Metabolism
10	96240001	96241000	BLNK	Cytoskeleton
10	96458001	96459000	TLL2	Protease

10	96557001	96558000	TM9SF3;NPM1P26	Transport
10	96736001	96737000	RNU6-1274P	
10	97900001	97902000	CRTAC1;CRTAC1	;
10	98551001	98552000	HPSE2	Metabolism
10	100081001	100082000	CPN1	Protease
10	100144001	100145000	CYP2C23P;SPCS2P2;ERLIN1	
10	100381001	100382000	OLMALINC	
10	100908001	100909000	SLF2	
10	101047001	101048000	SFXN3	Transport
10	101112001	101113000	TLX1NB	
10	101959001	101960000	ARMH3	
10	103076001	103078000	CNNM2;CNNM2	;
10	103364001	103365000	TAF5	
10	103431001	103432000	PDCD11	Metabolism
10	103473001	103474000	CALHM3;NEURL1-AS1	
10	103620001	103621000	SH3PXD2A	
10	103680001	103681000	SH3PXD2A	
10	104067001	104068000	COL17A1	Extracellular Matrix
10	108723001	108724000	LINC02661	
10	109985001	109986000	ADD3-AS1	
10	110816001	110817000	RBM20	
10	112776001	112777000	VTI1A	Transcription
10	112817001	112819000	VTI1A;LOC105378487;LOC112268058;LOC103344931;VTI1A;LOC112268058;LOC103344931	Transcription;Transcription
10	112843001	112844000	VTI1A	Transcription
10	113546001	113547000	HABP2	Protease
10	114409001	114410000	AFAP1L2	
10	114455001	114456000	ABLIM1	Cytoskeleton
10	115080001	115081000	LOC105378496	
10	115230001	115231000	ATRNL1	Extracellular Matrix
10	115614001	115615000	ATRNL1	Extracellular Matrix
10	116050001	116051000	GFRA1	Receptor
10	116528001	116529000	RNU6-1090P	
10	117085001	117086000	SHTN1	
10	118666001	118667000	CACUL1	
10	118741001	118742000	CACUL1	
10	119050001	119051000	EIF3A;SNORA19	Translation
10	119505001	119506000	RGS10	
10	119520001	119521000	RGS10	
10	119563001	119564000	TIAL1	Translation
10	119721001	119722000	INPP5F	Signaling
10	119855001	119856000	MCMBP	
10	121584001	121585000	FGFR2	Receptor
10	121815001	121816000	ATE1	Translation
10	122318001	122319000	BTBD16	
10	123681001	123682000	GPR26	Signaling
10	123894001	123895000	CPXM2;LOC105378534	Protease
10	124614001	124615000	LHPP;LOC105378539;FAM53B	Signaling
10	125012001	125013000	CTBP2	Transcription
10	125052001	125053000	CTBP2	Transcription
10	125647001	125648000	TEX36	
10	125778001	125779000	MMP21;UROS	Protease;Metabolism
10	125797001	125798000	UROS	Metabolism
10	125977001	125978000	FANK1;FANK1-AS1	
10	126106001	126107000	ADAM12	Protease
10	126637001	126638000	C10orf90	
10	126644001	126645000	C10orf90	
10	127100001	127101000	DOCK1	Transcription
10	127106001	127107000	DOCK1	Transcription
10	127446001	127448000	DOCK1;DOCK1	Transcription;Transcription
10	127886001	127887000	CLRN3	
10	127924001	127925000	PTPRE	Signaling
10	128072001	128073000	PTPRE;AS-PTPRE	Signaling
10	129761001	129763000	MGMT;MGMT	;
10	130232001	130234000	LINC02646;LINC02646	;
10	130422001	130423000	LINC02646	

10	131148001	131149000	TCERG1L	Transcription
10	131176001	131177000	TCERG1L	Transcription
10	131773001	131774000	LINC01164	
10	131916001	131917000	PPP2R2D	Signaling
10	131977001	131978000	PPP2R2D;BNIP3	Signaling
10	131985001	131986000	BNIP3	
10	132221001	132222000	STK32C	Signaling
10	132260001	132261000	STK32C	Signaling
10	132364001	132365000	LRRC27	
10	132573001	132575000	INPP5A;INPP5A	Signaling;Signaling
10	132645001	132646000	INPP5A	Signaling
10	132663001	132665000	INPP5A;INPP5A	Signaling;Signaling
10	132714001	132716000	INPP5A;INPP5A	Signaling;Signaling
10	132789001	132790000	INPP5A;NKX6-2	Signaling;Development
10	132858001	132859000	CFAP46;LOC105378571	
10	132910001	132911000	CFAP46	
10	132989001	132990000	LOC107984283	
10	133010001	133012000	LOC112268067;LOC112268067	;
10	133111001	133112000	ADGRA1	Signaling
10	133175001	133176000	KNDC1	Transcription
10	133179001	133180000	KNDC1	Transcription
10	133208001	133209000	KNDC1;LOC105378573	Transcription
10	133223001	133224000	KNDC1;LOC105378573;UTF1	Transcription
10	133253001	133254000	MIR202HG;MIR202;ADAM8	Protease
10	133481001	133482000	OR7M1P;LOC105378575	
10	133665001	133666000	CLUHP5;DUX4L28;DUX4L25;DUX4L24;DUX4L23	
10	133672001	133673000	CLUHP5;DUX4L28;DUX4L25;DUX4L24;DUX4L23;DUX4L22;DUX4L21	
10	133746001	133748000	DUX4L29;DUX4L10;DUX4L11;DUX4L12;DUX4L13;DUX4L14;DUX4L29;DUX4L10;DUX4L11;DUX4L12;DUX4L13;DUX4L14	;
10	133749001	133750000	DUX4L29;DUX4L10;DUX4L11;DUX4L12;DUX4L13;DUX4L14;DUX4L15	
11	223001	224000	RIC8A;SIRT3	
11	233001	234000	SIRT3;PSMD13;LOC100420679	Protease
11	325001	326000	IFITM1;IFITM3;LOC105376504;LOC105376505	
11	398001	399000	PKP3;SIGIRR	Cytoskeleton;Receptor
11	449001	450000	ANO9;LOC105376506;RN7SL838P;PTDSS2	Transport
11	461001	462000	PTDSS2	Transport
11	592001	593000	PHRF1	
11	751001	752000	LOC105376509;TALDO1	Metabolism
11	999001	1000000	AP2A2	Transport
11	1017001	1019000	AP2A2;MUC6;AP2A2;MUC6	Transport;Extracellular Matrix;Transport;Extracellular M
11	1032001	1033000	MUC6	Extracellular Matrix
11	1055001	1056000	LINC02688	
11	1095001	1096000	MUC2	Extracellular Matrix
11	1236001	1237000	MUC5B;MUC5B-AS1	Extracellular Matrix
11	1255001	1257000	MUC5B;MUC5B-AS1;MIR6744;MUC5B;MUC5B-AS1;MIR6744	Extracellular Matrix;Extracellular Matrix
11	1675001	1676000	FAM99A;FAM99B	
11	1735001	1736000	IFITM10	
11	1892001	1894000	LSP1;LINC01150;LSP1;LINC01150	Cytoskeleton;Cytoskeleton
11	1924001	1925000	TNNT3	Cytoskeleton
11	2125001	2126000	INS-IGF2;IGF2;MIR483	Growth Factors
11	2469001	2471000	KCNQ1;KCNQ1	Transport;Transport
11	2798001	2800000	KCNQ1;KCNQ1	Transport;Transport
11	2926001	2927000	SLC22A18;PHLDA2	Transport
11	3042001	3043000	CARS1;CARS1-AS1;RNU1-91P	Translation
11	3410001	3411000	TSSC2;FAM86GP	
11	3653001	3654000	ART1	Transport
11	3994001	3995000	STIM1;HMGN1P21	
11	4005001	4006000	STIM1	
11	4320001	4322000	SSU72P3;SSU72P3	Signaling;Signaling
11	4736001	4737000	MMP26;OR51F3P	Protease
11	4933001	4934000	MMP26;OR51G1;OR51A3P;OR51A4	Protease;Receptor
11	6404001	6405000	SMPD1;APBB1	Signaling
11	6408001	6409000	APBB1	
11	7076001	7077000	NLRP14	
11	7577001	7578000	PPFIBP2;LOC105376535	
11	7580001	7581000	PPFIBP2;LOC105376535	

11	8149001	8150000	RIC3	
11	8700001	8701000	DENND2B	
11	8730001	8731000	DENND2B	
11	8859001	8860000	DENND2B	
11	8869001	8870000	DENND2B	
11	8995001	8996000	NRIP3;NRIP3-DT	Proteolysis
11	9221001	9222000	DENND5A	
11	9456001	9458000	IPO7;LOC644656;ZNF143;IPO7;LOC644656;ZNF143	Transport;Transcription;Transport;Transcription
11	9583001	9584000	WEE1	Signaling
11	9662001	9663000	RPL21P97;SWAP70	Cytoskeleton
11	9873001	9874000	SBF2;LOC101928008	Signaling
11	12149001	12150000	MICAL2	
11	13465001	13466000	BTBD10	
11	13856001	13857000	LINC02548;LINC02545	
11	14732001	14733000	PDE3B	Signaling
11	16499001	16501000	SOX6;SOX6	;
11	17860001	17861000	SERGEF;LOC107984317	Proteolysis
11	18175001	18176000	MRGPRX4	Signaling
11	18474001	18475000	LDHAL6A;TSG101	Metabolism;Proteolysis
11	18630001	18631000	SPTY2D1;LOC105376578	Epigenetic
11	18762001	18763000	PTPN5	
11	19174001	19175000	ZDHHC13;CSRP3	Cytoskeleton
11	19589001	19590000	NAV2	
11	20120001	20121000	NAV2;LOC107984418	
11	22650001	22651000	GAS2	
11	23382001	23383000	LOC100131557	
11	27522001	27523000	BDNF-AS;MIR8087;RNA5SP339	
11	27581001	27582000	BDNF-AS;RPS25P1	
11	30194001	30195000	ARL14EP-DT	
11	30267001	30268000	ARL14EP-DT	
11	30417001	30418000	MPPED2	Metabolism
11	30545001	30546000	MPPED2	Metabolism
11	31547001	31548000	ELP4	
11	32591001	32592000	EIF3M;HNRNPA3P9	
11	33243001	33244000	RPS24P15	
11	34315001	34316000	ABTB2	Cytoskeleton
11	35757001	35758000	TRIM44	Proteolysis
11	36263001	36264000	COMMD9	
11	36753001	36754000	LOC107984326	
11	45111001	45112000	PRDM11;LOC105376652	Transcription
11	45157001	45158000	PRDM11	Transcription
11	46060001	46061000	PHF21A	
11	46430001	46431000	AMBRA1;RPS10P19	
11	46478001	46479000	AMBRA1	
11	46805001	46806000	CKAP5	Cytoskeleton
11	47165001	47166000	C11orf49;ARFGAP2	Signaling
11	47358001	47359000	MYBPC3;SPI1	Transcription
11	47360001	47362000	MYBPC3;SPI1;MYBPC3;SPI1	Transcription;Transcription
11	47905001	47906000	LOC100287189	
11	47972001	47973000	PTPRJ	Signaling
11	48167001	48169000	PTPRJ;PTPRJ	Signaling;Signaling
11	48412001	48413000	OR4C2P	
11	57104001	57105000	LOC105369309	
11	57437001	57438000	SLC43A3	Transport
11	57824001	57825000	TMX2-CTNND1;CTNND1	Cytoskeleton
11	58054001	58055000	OR9Q1	Receptor
11	58300001	58301000	OR10Q2P	
11	60621001	60622000	LOC105369321;LINC00301	
11	60683001	60684000	LOC105369321;LINC00301	
11	61025001	61026000	CD6;LOC105369325	Protease
11	61387001	61388000	TMEM138;TMEM216	
11	61711001	61712000	DAGLA	Metabolism
11	62992001	62994000	SLC22A6;SLC22A8;SLC22A6;SLC22A8	Transport;Transport
11	63420001	63421000	SLC22A9	Transport
11	63820001	63821000	SPINDOC	
11	63894001	63895000	MARK2;TRR-CCT9-1	Signaling

11	64235001	64236000	TRPT1;NUDT22;DNAJC4;VEGFB;FKBP2;LOC114841035;PPP1R14B;PPP1R14B-AS1	Transport;Signaling;Growth Factors;Signaling
11	64371001	64372000	RPS6KA4;MIR1237	Golgi
11	64720001	64721000	NRXN2;RASGRP2	Transcription
11	64754001	64756000	RASGRP2;PYGM;SF1;RASGRP2;PYGM;SF1	Transcription;Golgi;Translation;Transcription;Golgi;Tran
11	64808001	64809000	MAP4K2;MEN1	
11	65052001	65053000	SAC3D1;NAALADL1;CDCA5	Cytoskeleton;Protease
11	65318001	65319000	LOC105369344;CDC42EP2	
11	65715001	65716000	KAT5;RNASEH2C	Epigenetic
11	66096001	66097000	PACS1	
11	66367001	66369000	LOC102724064;SLC29A2;LOC102724064;SLC29A2	Transport;Transport
11	66393001	66394000	BRD9P1;RNU1-84P	
11	66825001	66826000	C11orf80	
11	67633001	67634000	NUDT8;TBX10;ACY3	Signaling;Transcription
11	67669001	67670000	ALDH3B2	Metabolism
11	67737001	67738000	OR7E11P	
11	67868001	67869000	ENPP7P7	
11	68130001	68131000	CHKA;CHKA-DT	Signaling
11	68265001	68266000	C11orf24;LOC105369363	
11	68912001	68914000	MRPL21;IGHMBP2;MRPL21;IGHMBP2	Translation;Transcription;Translation;Transcription
11	69005001	69006000	MRGPRF;MRGPRF-AS1	Signaling
11	69097001	69098000	TPCN2;LOC107984344	Transport
11	70019001	70021000	ANO1;ANO1	;
11	70026001	70027000	ANO1	
11	70195001	70196000	ANO1;FADD	
11	70371001	70372000	PPFIA1;LOC107984347	
11	70388001	70389000	PPFIA1;CTTN	Cytoskeleton
11	70436001	70437000	CTTN	Cytoskeleton
11	70668001	70669000	SHANK2	
11	71133001	71134000	SHANK2	
11	71396001	71397000	ACTE1P	
11	71398001	71399000	ACTE1P	
11	71482001	71483000	NADSYN1;MIR6754	Metabolism
11	71629001	71630000	OR7E4P;LOC645544	
11	71740001	71741000	ENPP7P8	
11	72662001	72663000	PDE2A;PDE2A-AS1	Signaling
11	73590001	73591000	FAM168A;HMGN2P38	Cytoskeleton
11	74172001	74173000	C2CD3;PPME1	Golgi
11	74845001	74846000	RNF169;XRRA1;RN7SL239P	
11	75192001	75193000	SLCO2B1	Transport
11	75450001	75451000	GDPD5	Signaling
11	76116001	76117000	UVRAG	
11	76194001	76195000	WNT11	Signaling
11	76627001	76628000	LINC02757	
11	76875001	76876000	ACER3	
11	77112001	77113000	CAPN5;OMP	Protease
11	77295001	77297000	GDPD4;GDPD4	Signaling;Signaling
11	78104001	78105000	ALG8	Golgi
11	82404001	82405000	MIR4300HG	
11	83213001	83214000	ANKRD42;CKS1BP4;RPL32P24	
11	84268001	84269000	DLG2	Cytoskeleton
11	84313001	84314000	DLG2	Cytoskeleton
11	85757001	85758000	SYTL2	
11	85952001	85953000	PICALM	Transport
11	87050001	87051000	TMEM135	
11	87061001	87062000	TMEM135	
11	87217001	87218000	TMEM135	
11	87245001	87246000	TMEM135	
11	87495001	87496000	LOC107984361	
11	89141001	89142000	LOC107984363	
11	90403001	90404000	DISC1FP1	
11	92170001	92171000	RPL7AP57	
11	92487001	92488000	FAT3	Cytoskeleton
11	93243001	93244000	SLC36A4	Transport
11	94269001	94270000	LOC105369435	
11	94278001	94279000	LOC105369435	
11	94392001	94393000	GPR83	Signaling

11	95507001	95508000	LOC105369440	
11	99377001	99378000	CNTN5	
11	101991001	101992000	CEP126	Transcription
11	102264001	102265000	LOC105369460	
11	102402001	102403000	TMEM123	
11	102648001	102649000	LOC101928477	
11	106105001	106106000	AASDHPPT;LINC02719	Transport
11	106774001	106775000	GUCY1A2;LOC112268081;LOC100418884	Signaling
11	107362001	107363000	CWF19L2	
11	107952001	107953000	RAB39A	
11	108248001	108249000	ATM	Signaling
11	108273001	108274000	ATM	Signaling
11	111492001	111493000	BTG4	
11	111529001	111530000	HOATZ	
11	111696001	111697000	SIK2;PPP2R1B	Signaling;Signaling
11	111948001	111949000	DIXDC1	Cytoskeleton
11	112030001	112031000	DIXDC1;DLAT;PPIHP1;RNU6-893P	Cytoskeleton;Metabolism
11	112187001	112188000	BCO2;KCTD9P4	Metabolism
11	112746001	112747000	LOC105369496	
11	112990001	112991000	NCAM1	
11	113041001	113042000	NCAM1	
11	113154001	113155000	NCAM1	
11	113350001	113351000	TTC12	
11	114259001	114260000	ZBTB16;NNMT	Transcription;Epigenetic
11	114383001	114384000	LOC101928940;LOC107984391;C11orf71	
11	114479001	114480000	NXPE2	
11	115580001	115581000	RPL12P46	
11	115816001	115817000	LINC02698	
11	115946001	115947000	LINC02703;RPL15P15	
11	116454001	116455000	LOC107987166	
11	116831001	116832000	APOA4;APOC3;APOA1;APOA1-AS	Binding Proteins
11	117228001	117229000	PCSK7;RNF214	Protease
11	117605001	117606000	DSCAML1	Cytoskeleton
11	117908001	117909000	TMPRSS13	Protease
11	118882001	118883000	CXCR5	
11	118988001	118990000	FOXR1;CENATAC-DT;CENATAC;FOXR1;CENATAC-DT;CENATAC	;
11	119064001	119065000	HYOU1;VPS11	Transport
11	119628001	119629000	LOC105369520;NECTIN1	
11	119648001	119649000	LOC105369520;NECTIN1;RNU6-1123P	
11	120550001	120551000	GRIK4	Receptor
11	120785001	120786000	GRIK4	Receptor
11	120817001	120818000	GRIK4;LOC105369532	Receptor
11	121642001	121643000	SORL1	Transport
11	122766001	122767000	UBASH3B	
11	123087001	123088000	CLMP	
11	123338001	123339000	PHBP17	
11	123520001	123521000	GRAMD1B	
11	123610001	123611000	GRAMD1B	
11	124086001	124087000	LOC107984403;LOC105369545;OR10D4P	Receptor
11	124205001	124206000	OR8F1P;OR8G3P	Receptor
11	125089001	125090000	SLC37A2;TMEM218	Transport
11	125194001	125195000	PKNOX2	Development
11	125329001	125330000	PKNOX2	Development
11	125381001	125382000	PKNOX2	Development
11	125882001	125883000	HYLS1	
11	126198001	126199000	RPUSD4	Translation
11	126347001	126348000	DCPS;GSEC;ST3GAL4	Metabolism;Transport
11	126450001	126451000	KIRREL3	
11	126795001	126796000	KIRREL3	
11	126799001	126800000	KIRREL3	
11	127233001	127234000	LOC107984379	
11	127264001	127265000	LINC02712	
11	129388001	129389000	BARX2	Development
11	129848001	129849000	TMEM45B	
11	129920001	129921000	PRDM10	Transcription
11	129952001	129953000	PRDM10	Transcription

11	130134001	130136000	APLP2;APLP2	Protease; Proteolysis;Protease; Proteolysis
11	130465001	130466000	ADAMTS15	Protease
11	130918001	130919000	SNX19	Cytoskeleton
11	131608001	131609000	NTM	Immune
11	131615001	131616000	NTM	Immune
11	132162001	132163000	NTM	Immune
11	132657001	132658000	OPCML	Immune
11	133140001	133142000	OPCML;OPCML	Immune;Immune
11	133464001	133465000	OPCML	Immune
11	133518001	133519000	OPCML	Immune
11	133950001	133951000	IGSF9B	
11	134739001	134740000	LINC02714	
11	134985001	134986000	LOC100507548	
12	10001	11000	DDX11L8;WASH8P	Epigenetic
12	149001	150000	IQSEC3;LOC574538	Transcription
12	511001	512000	B4GALNT3	Golgi
12	550001	552000	B4GALNT3;B4GALNT3	Golgi;Golgi
12	1794001	1795000	ADIPOR2;CACNA2D4;LOC105369602	Signaling;Transport
12	2051001	2052000	CACNA1C;CACNA1C-IT2	Transport
12	2138001	2139000	CACNA1C;LOC107984131	Transport
12	2256001	2257000	CACNA1C	Transport
12	2576001	2577000	CACNA1C	Transport
12	2587001	2589000	CACNA1C;CACNA1C	Transport;Transport
12	2611001	2612000	CACNA1C	Transport
12	2793001	2794000	ITFG2-AS1;CBX3P4;FKBP4	Transcription
12	2863001	2864000	ITFG2;TEX52;FOXM1	Extracellular Matrix
12	2873001	2874000	FOXM1;RHNO1	
12	2892001	2893000	RHNO1;TULP3	
12	2914001	2915000	TULP3	
12	2939001	2940000	TULP3	
12	3006001	3007000	TEAD4	Transcription
12	3275001	3278000	TSPAN9;TSPAN9;TSPAN9	;;
12	3320001	3321000	LINC02827;LOC100128253;LOC100418939	
12	3502001	3503000	PRMT8;THCAT155	Golgi
12	3583001	3584000	PRMT8	Golgi
12	3602001	3603000	PRMT8	Golgi
12	3649001	3650000	CRACR2A	
12	3893001	3894000	PARP11-AS1	
12	4289001	4290000	CCND2	Signaling
12	5055001	5056000	KCNA5	Transport
12	5467001	5468000	NTF3	Growth Factors
12	5491001	5492000	NTF3	Growth Factors
12	5929001	5930000	ANO2	
12	6039001	6040000	VWF;SNORA120	Extracellular Matrix
12	6180001	6182000	LOC105369623;LOC105369622;LOC105369623;LOC105369622	;
12	6629001	6630000	LOC105369631;LPAR5;ACRBP	Signaling
12	6660001	6661000	ING4;ZNF384	Epigenetic;Transcription
12	6784001	6785000	LAG3;RN7SL380P;CD4	Receptor
12	6834001	6835000	GPR162;P3H3;GNB3;CDCA3	Signaling;Extracellular Matrix;Signaling
12	7209001	7210000	PEX5	Transport
12	7235001	7236000	LOC100420983	
12	7641001	7642000	APOBEC1	Translation
12	7755001	7756000	CLEC4C;NANOGNB	Transport
12	8062001	8063000	FOXJ2;C3AR1	Signaling
12	8677001	8678000	LOC101060064;RIMKLB	Translation
12	8980001	8981000	KLRG1	Receptor
12	9127001	9128000	KLRG1;KRT17P8;BTG1P1	Receptor
12	9219001	9220000	KLRG1;A2MP1	Receptor
12	9287001	9288000	LOC642846	
12	9738001	9739000	CLECL1	Receptor
12	9920001	9921000	CLEC2A	
12	10199001	10200000	TMEM52B;LOC107984509	
12	12263001	12264000	LRP6	Binding Proteins
12	12299001	12300000	RNU6-318P	
12	12371001	12372000	BORCS5	
12	15516001	15517000	PTPRO;LOC105369672	Receptor

12	21927001	21929000	ABCC9;ABCC9	Transport;Transport
12	22308001	22309000	ST8SIA1;LOC112268093	Transport
12	24904001	24905000	BCAT1	Metabolism
12	25166001	25167000	DNAI7	
12	25655001	25656000	LMNTD1	
12	26946001	26947000	INTS13;FGFR1OP2	
12	27783001	27784000	MANSC4;KLHL42	
12	29294001	29295000	FAR2;LOC100506606	
12	29409001	29410000	OVCH1-AS1	
12	30500001	30501000	LOC100422352	
12	30518001	30519000	LOC100422352	
12	30641001	30642000	IPO8	Transport
12	31087001	31088000	DDX11	Epigenetic
12	31476001	31477000	DENND5B;MRPL30P2	
12	31554001	31555000	DENND5B	
12	31656001	31657000	ETFBKMT	Epigenetic
12	31748001	31749000	LOC105369724;IFITM3P2	
12	32374001	32375000	BICD1	
12	34206001	34207000	RNA5SP357;DUX4L27	
12	34243001	34244000	AK6P1	
12	38151001	38152000	LOC107984469;RNA5SP358	
12	38156001	38157000	RNA5SP358;RNA5SP359	
12	41493001	41494000	PDZRN4	
12	43563001	43564000	RPL21P101;LOC400026	
12	44619001	44620000	NELL2	Signaling
12	45967001	45968000	SCAF11	
12	46011001	46012000	RPL13AP21	
12	46645001	46646000	LOC100288798	
12	46974001	46975000	LOC107984466	
12	47416001	47417000	LINC02156	
12	48076001	48077000	SENP1;RNU6-1203P;LOC101927180	Protease
12	48428001	48429000	C12orf54	
12	48644001	48645000	KANSL2;SNORA2C;MIR1291	
12	49100001	49101000	DHH;LOC105369759;LMBR1L	Receptor
12	50173001	50174000	CERS5;LIMA1	Cytoskeleton
12	50179001	50180000	LIMA1	Cytoskeleton
12	50335001	50336000	FAM186A	
12	50372001	50373000	FAM186A;RPL26P33	
12	50677001	50678000	DIP2B	
12	50743001	50744000	DIP2B	
12	53120001	53121000	SOAT2	Metabolism
12	53170001	53171000	CSAD;ZNF740	Metabolism;Transcription
12	53377001	53378000	SP1	Transcription
12	53432001	53434000	AMHR2;PRR13;AMHR2;PRR13	Signaling;Signaling
12	54211001	54212000	LOC105369777	
12	54471001	54472000	LOC102724050;GTSF1	
12	56352001	56353000	STAT2;RNU7-40P;APOF	Transcription;Binding Proteins
12	56413001	56414000	TIMELESS	
12	57272001	57273000	R3HDM2	
12	57543001	57544000	DCTN2;KIF5A	Cytoskeleton
12	57573001	57574000	KIF5A	Cytoskeleton
12	57669001	57670000	RPL13AP23	
12	57901001	57902000	LOC283387;LOC101927608	
12	59754001	59755000	SLC16A7	Transport
12	61602001	61603000	DUX4L52	
12	63050001	63051000	RSL24D1P5	
12	63880001	63881000	SRGAP1;RPL36AP41;LOC100419700	Signaling
12	64682001	64683000	RASSF3	Cytoskeleton
12	64699001	64700000	RASSF3	Cytoskeleton
12	65303001	65305000	MSRB3;MSRB3	Metabolism;Metabolism
12	65515001	65516000	LOC100507065	
12	67052001	67053000	GRIP1	
12	67679001	67680000	LOC105369816	
12	69469001	69470000	LINC02373;FRS2	
12	69723001	69724000	LOC101928002	
12	69773001	69774000	RAB3IP	Transcription

12	70806001	70807000	PTPRR	
12	72519001	72520000	TRHDE	Protease
12	73003001	73004000	LOC105369838	
12	75381001	75382000	CAPS2;GLIPR1L2	Signaling;Immune
12	75743001	75744000	LOC105369844	
12	75942001	75943000	LOC105369844	
12	79228001	79229000	SYT1	Transport
12	79452001	79453000	SYT1	Transport
12	80092001	80093000	OTOGL;RPL26P32	Extracellular Matrix
12	80227001	80228000	OTOGL	Extracellular Matrix
12	83071001	83072000	TMTC2	Golgi
12	83142001	83143000	TMTC2;RPL6P25	Golgi
12	88134001	88135000	CEP290;TMTC3	
12	89497001	89498000	POC1B;CENPCP1	
12	90926001	90927000	LINC00615	
12	93609001	93610000	SOCS2	Signaling
12	94002001	94003000	LOC105369912;RN7SKP263	
12	94180001	94181000	PLXNC1	
12	94265001	94266000	PLXNC1;CEP83	
12	94465001	94466000	CEP83;CEP83-DT;LOC102724960	
12	95469001	95471000	RPL29P26;METAP2;RPL29P26;METAP2	Protease;Protease
12	95504001	95505000	METAP2	Protease
12	95531001	95532000	USP44	Protease
12	95778001	95779000	NTN4	Extracellular Matrix
12	95928001	95929000	CCDC38	
12	95956001	95957000	AMDHD1	Protease
12	98501001	98502000	LINC02453;TRD-GTC1-1;TRW-CCA4-1	
12	98710001	98711000	APAF1	
12	98719001	98720000	APAF1;ANKS1B	Cytoskeleton
12	99065001	99067000	ANKS1B;ANKS1B	Cytoskeleton;Cytoskeleton
12	99224001	99225000	ANKS1B	Cytoskeleton
12	99715001	99716000	ANKS1B	Cytoskeleton
12	100273001	100274000	DEPDC4;SCYL2	Cytoskeleton;Signaling
12	100608001	100609000	GAS2L3	
12	101432001	101433000	RPS27P23	
12	101895001	101896000	DRAM1	
12	101907001	101908000	DRAM1	
12	103223001	103224000	C12orf42	
12	103296001	103297000	C12orf42	
12	103835001	103836000	NT5DC3;LOC107984433;TTC41P	Signaling
12	103999001	104000000	GLT8D2	Golgi
12	104089001	104090000	HCFC2	
12	104977001	104979000	KRT18P20;LOC105369952;NOPCHAP1;KRT18P20;LOC105369952;NOPCHAP1	;
12	107376001	107377000	BTBD11	Cytoskeleton
12	107637001	107639000	BTBD11;BTBD11	Cytoskeleton;Cytoskeleton
12	109105001	109106000	UNG;ACACB	Epigenetic
12	109208001	109209000	ACACB	
12	109256001	109257000	ACACB	
12	110088001	110089000	LOC105369976	
12	110340001	110341000	ATP2A2	Transport
12	111280001	111282000	CUX2;LOC105369983;CUX2;LOC105369983	Development;Development
12	111326001	111327000	CUX2;LOC105369983	Development
12	111545001	111546000	ATXN2	Metabolism
12	111853001	111854000	MAPKAPK5-AS1;MAPKAPK5	Signaling
12	111881001	111882000	MAPKAPK5;RPS2P41	Signaling
12	112398001	112399000	RPL6	Translation
12	112480001	112481000	PTPN11	Signaling
12	112975001	112976000	OAS3;OAS2	Metabolism
12	113017001	113018000	OAS2;IMMP1LP2	Metabolism
12	113320001	113322000	SLC8B1;SLC8B1	Transport;Transport
12	113373001	113374000	PLBD2	Metabolism
12	113642001	113643000	LOC105369990	
12	113788001	113789000	DYNLL1P4	
12	113844001	113846000	RBM19;RBM19	Translation;Translation
12	113878001	113879000	RBM19	Translation
12	113919001	113920000	RBM19	Translation

12	115972001	115973000	MED13L	
12	116720001	116721000	SPRING1	
12	116807001	116809000	RNFT2;RNFT2	;
12	117183001	117184000	FBXO21	
12	117281001	117282000	NOS1	Metabolism
12	117921001	117922000	KSR2	Signaling
12	118211001	118212000	TAOK3	Signaling
12	118905001	118906000	LOC105370019	
12	118961001	118962000	LOC105370020	
12	120098001	120100000	BICDL1;RAB35;BICDL1;RAB35	Transport;Transport
12	120876001	120877000	SPPL3	Proteolysis
12	120911001	120912000	SPPL3;XLOC_009911;CLIC1P1;RPL12P33	Proteolysis
12	120999001	121000000	HNF1A;C12orf43	Transcription
12	121138001	121139000	LOC105370032;P2RX7	Ion Channel
12	121217001	121218000	LOC105370032;P2RX4	Ion Channel
12	121621001	121622000	ORAI1	Transport
12	122043001	122044000	BCL7A	
12	122085001	122086000	MLXIP	Transcription
12	122093001	122094000	MLXIP	Transcription
12	122181001	122182000	LRRC43;IL31	Signaling;Cytokine
12	122189001	122190000	LRRC43	Signaling
12	122463001	122464000	ZCCHC8	Metabolism
12	122473001	122474000	ZCCHC8	Metabolism
12	123005001	123007000	PITPNM2;MIR4304;PITPNM2;MIR4304	Transport;Transport
12	123235001	123236000	MPHOSPH9;MTRFR	
12	123256001	123257000	MTRFR;CDK2AP1	Signaling
12	123512001	123513000	RILPL1	
12	123654001	123655000	GTF2H3	Transcription
12	123674001	123675000	TCTN2	
12	123745001	123746000	ATP6V0A2	Metabolism
12	123841001	123842000	DNAH10	Cytoskeleton
12	124181001	124182000	ZNF664-RFLNA	
12	124313001	124315000	ZNF664-RFLNA;RFLNA;ZNF664-RFLNA;RFLNA;NCOR2	;Epigenetic
12	124433001	124434000	NCOR2	Epigenetic
12	124438001	124440000	NCOR2;NCOR2	Epigenetic;Epigenetic
12	124966001	124967000	DHX37	Transcription
12	124984001	124985000	DHX37;BRI3BP	Transcription
12	125000001	125002000	BRI3BP;BRI3BP	;
12	125104001	125106000	AACS;AACS	Metabolism;Metabolism
12	125973001	125974000	LINC00939	
12	126417001	126418000	LINC02825;LINC02350	
12	127035001	127036000	LINC02405;LOC105370063	
12	127868001	127869000	LOC100419932	
12	128432001	128433000	TMEM132C	
12	128473001	128474000	TMEM132C	
12	128803001	128804000	SLC15A4	Transport
12	128856001	128857000	LOC100128276;GLT1D1	
12	128932001	128933000	GLT1D1;AK3P6	
12	130338001	130339000	LOC101927786;PIWIL1	Translation
12	130573001	130574000	RIMBP2	
12	130990001	130991000	ADGRD1;ADGRD1-AS1	Signaling
12	131588001	131589000	LOC105370084	
12	131652001	131653000	LINC02414;LOC105370086;RNA5SP377	
12	131905001	131906000	ULK1	Signaling
12	132099001	132100000	EP400P1	
12	132156001	132159000	NOC4L;LOC105370090;NOC4L;LOC105370090;NOC4L;LOC105370090	Metabolism;Metabolism;Metabolism
12	132203001	132204000	LOC107984456;GALNT9;LOC105370091	Golgi
12	132213001	132214000	GALNT9;LOC105370091	Golgi
12	132243001	132244000	GALNT9	Golgi
12	132441001	132442000	LOC105370092;LOC112268102	
12	132714001	132715000	PXMP2;PGAM5;RNA5SP379	Transport;Metabolism
12	132717001	132718000	PGAM5;RNA5SP379;ANKLE2	Metabolism
12	132802001	132804000	GOLGA3;GOLGA3	Cytoskeleton;Cytoskeleton
12	132817001	132819000	GOLGA3;RPS11P5;GOLGA3;RPS11P5	Cytoskeleton;Cytoskeleton
12	132878001	132879000	CHFR;LOC101928530	Proteolysis
12	133093001	133094000	ZNF140	

12	133221001	133222000	ZNF268;ANHX	Transcription;Development
13	18298001	18299000	KMT5AP1	
13	18904001	18905000	SNX19P2;LINC00408	
13	19028001	19029000	USP24P1;GTF2IP3	
13	19137001	19138000	CENPIP1;RNU6-52P	
13	20336001	20337000	LOC105370102	
13	20398001	20399000	CRYL1	Metabolism
13	20718001	20719000	IL17D;EEF1AKMT1	
13	23360001	23361000	SACS	
13	23814001	23815000	MIPEP	Protease
13	24245001	24246000	SPATA13;SPATA13-AS1	Transcription
13	24284001	24285000	SPATA13	Transcription
13	24291001	24292000	SPATA13	Transcription
13	24559001	24560000	LOC101927375;PSPC1P2	
13	24567001	24568000	LOC101927375;PSPC1P2	
13	24682001	24683000	ATP12A	Transport
13	24699001	24700000	ATP12A;RNY1P7;RPL26P34	Transport
13	24764001	24765000	RNF17;LOC112268115	
13	24868001	24869000	RNF17	
13	25154001	25155000	AMER2	
13	25870001	25871000	ATP8A2	Transport
13	25886001	25887000	ATP8A2	Transport
13	25929001	25930000	ATP8A2	Transport
13	25975001	25976000	ATP8A2;LOC105370122	Transport
13	26548001	26549000	WASF3	Cytoskeleton
13	27672001	27673000	POLR1D;LOC105370128	Transcription
13	28265001	28266000	PAN3	Transcription
13	28925001	28926000	MTUS2	
13	29382001	29383000	MTUS2	
13	29481001	29482000	MTUS2;MTUS2-AS1	
13	30433001	30434000	UBE2L5	Proteolysis
13	30913001	30914000	TEX26-AS1;MEDAG	
13	32290001	32291000	FRY	Cytoskeleton
13	32436001	32437000	N4BP2L1;N4BP2L2	
13	32831001	32832000	LINC00423	
13	33202001	33203000	STARD13	Signaling
13	34380001	34381000	LINC02343	
13	34707001	34708000	LOC107987189	
13	34986001	34987000	NBEA	
13	35009001	35010000	NBEA	
13	35085001	35086000	NBEA	
13	35428001	35429000	NBEA	
13	40796001	40797000	SLC25A15;TPTE2P5	Transport
13	41108001	41109000	MIR3168	
13	42135001	42136000	DGKH	Signaling
13	42427001	42428000	LINC02341	
13	42440001	42441000	LINC02341	
13	43220001	43221000	ENOX1	Metabolism
13	45118001	45119000	LOC101929259;GTF2F2;RN7SKP4	Transcription
13	46103001	46104000	CPB2-AS1;CPB2	Protease
13	46198001	46199000	LOC105370192;LOC107984578	
13	46504001	46505000	FKBP1AP3	
13	48056001	48058000	NUDT15;NUDT15	;
13	48311001	48312000	RB1-DT;RB1;PPP1R26P1	Epigenetic
13	49031001	49032000	FNDC3A	Proteolysis
13	49218001	49219000	FNDC3A;OGFOD1P1;MLNR	Proteolysis;Signaling
13	49497001	49498000	SETDB2-PHF11;SETDB2;SNRPGP14;PHF11	Epigenetic;Transcription
13	49712001	49713000	KPNA3	Transport
13	50166001	50167000	DLEU1;ST13P4	
13	50792001	50793000	DLEU7	
13	51002001	51003000	GUCY1B2	Signaling
13	51170001	51172000	C13orf42;RPL5P31;C13orf42;RPL5P31	;
13	51446001	51447000	INTS6;INTS6-AS1	Transcription
13	52745001	52746000	CNMD	
13	60092001	60093000	DIAPH3	
13	60208001	60209000	LINC00434	

13	60645001	60646000	EIF4A1P6	
13	62399001	62400000	SQSTM1P1	
13	62778001	62779000	LINC00448	
13	64968001	64969000	LGMNP1	
13	72898001	72899000	PIBF1	
13	73084001	73085000	KLF5	Transcription
13	73834001	73835000	KLF12	Transcription
13	75668001	75669000	LMO7	
13	78062001	78064000	LINC00446;OBI1-AS1;LINC00446;OBI1-AS1	;
13	80327001	80328000	SPRY2	Cytoskeleton
13	80716001	80717000	LOC105370278	
13	87612001	87613000	MIR4500HG;MIR4500	
13	91175001	91176000	LINC00379	
13	91542001	91543000	GPC5	
13	93355001	93356000	GPC6	
13	93562001	93563000	GPC6	
13	94266001	94267000	GPC6	
13	94321001	94322000	GPC6	
13	94336001	94337000	GPC6	
13	94380001	94381000	GPC6	
13	94545001	94546000	DCT;TRF-GAA1-5	Metabolism
13	94611001	94612000	GPR180;NDUFA3P5	
13	95737001	95739000	DNAJC3;DNAJC3	;
13	96404001	96405000	HS6ST3	Transport
13	97572001	97573000	LOC105370324	
13	97707001	97709000	LOC105370324;LOC105370324	;
13	98186001	98187000	FARP1;RNF113B	Translation
13	98407001	98408000	FARP1	
13	98463001	98464000	FARP1;STK24	
13	98634001	98635000	CYCSP35;CALM2P4	
13	99276001	99277000	UBAC2	
13	99358001	99359000	UBAC2;FKSG29;MIR623	
13	99530001	99531000	TM9SF2;RNY3P6	Transport
13	99716001	99717000	CLYBL;CLYBL-AS1	Metabolism
13	100298001	100299000	PCCA	Metabolism
13	100419001	100420000	PCCA	Metabolism
13	107818001	107819000	NALF1;NALF1-IT1;LOC107984581	
13	108267001	108268000	TNFSF13B	
13	108609001	108610000	MYO16	
13	109734001	109735000	LINC00676	
13	110489001	110490000	COL4A2	Extracellular Matrix
13	110654001	110655000	CARS2	Translation
13	110678001	110679000	CARS2	Translation
13	110719001	110720000	CARS2;ING1	Translation;Epigenetic
13	111178001	111179000	ARHGEF7;SALL4P4	Transcription
13	111590001	111591000	LINC02337	
13	112028001	112029000	SOX1-OT;LOC105378193	
13	112118001	112119000	SOX1-OT	
13	112358001	112359000	LOC105370373	
13	112745001	112746000	ATP11A;ATP11A-AS1	Transport
13	112898001	112899000	MCF2L	Transcription
13	113431001	113432000	ADPRHL1	Metabolism
13	113496001	113497000	DCUN1D2;TMCO3	
13	113522001	113523000	TMCO3	
13	113530001	113531000	TMCO3	
13	113541001	113543000	TMCO3;TMCO3	;
13	113606001	113607000	TFDP1	Transcription
13	113633001	113634000	TFDP1	Transcription
13	113670001	113671000	GRK1	Signaling
13	113672001	113673000	GRK1	Signaling
13	113737001	113738000	GRK1;LOC105377805	Signaling
13	113777001	113778000	TMEM255B	
13	113876001	113877000	GAS6-DT;LINC00454	
13	113942001	113943000	LOC105377806	
13	114012001	114013000	RASA3	Signaling
13	114017001	114018000	RASA3	Signaling

13	114041001	114042000	RASA3	Signaling
13	114108001	114109000	RASA3;RASA3-IT1	Signaling
13	114231001	114232000	CFAP97D2;CDC16	Proteolysis
13	114354001	114355000	LOC112268113	
14	19242001	19243000	LOC100420097;NBEAP6	
14	19300001	19301000	LINC01297-DUXAP10-NBEAP6;DUXAP10;BMS1P17	
14	19434001	19435000	POTEG;LOC101929572	Transport
14	19483001	19484000	MED15P1;NF1P4	
14	19718001	19719000	OR11H2	Signaling
14	20353001	20354000	PARP2;RPPH1	Transcription
14	20538001	20539000	SETP1	
14	20720001	20721000	RANBP20P;EDDM3A;LOC107984671	
14	21441001	21442000	CHD8	
14	22615001	22616000	ABHD4	
14	23016001	23017000	AJUBA-DT;C14orf93;PSMB5	Protease
14	23059001	23060000	CDH24;LOC105370705;ACIN1	Cytoskeleton
14	23531001	23533000	THTPA;ZFHX2;THTPA;ZFHX2	Signaling;Transcription;Signaling;Transcription
14	23584001	23585000	JPH4	
14	24068001	24069000	CARMIL3;LOC105370412;CPNE6;NRL	Transcription
14	24089001	24090000	NRL;PCK2	Transcription;Signaling
14	26678001	26679000	NOVA1-DT	
14	30959001	30960000	STRN3;HIGD1AP17	
14	33789001	33790000	NPAS3	Transcription
14	34440001	34441000	SPTSSA	Golgi
14	34459001	34460000	SPTSSA	Golgi
14	34560001	34561000	LOC105370449;RNU1-28P;SNX6;RPS19P3	Cytoskeleton
14	35714001	35715000	RALGAPA1	Signaling
14	38912001	38913000	LINC00639;LOC105370457	
14	40340001	40341000	LOC105370463;LOC105370462	
14	43536001	43538000	KRT8P2;KRT8P2	;
14	45069001	45070000	TOGARAM1	Cytoskeleton
14	45223001	45224000	MIS18BP1	
14	46427001	46428000	LINC00871	
14	47106001	47107000	MDGA2	
14	47173001	47174000	MDGA2	
14	47279001	47280000	MDGA2	
14	48404001	48405000	LOC100422641	
14	48779001	48780000	LOC105378178	
14	49178001	49179000	LOC105378178	
14	50292001	50293000	L2HGDH;MIR4504	Metabolism
14	50309001	50310000	L2HGDH;MIR4504;DMAC2L	Metabolism;Metabolism
14	50663001	50664000	SAV1	
14	51030001	51031000	TRIM9	Proteolysis
14	53156001	53157000	DDHD1;DDHD1-DT	Metabolism
14	53323001	53324000	LOC112268134	
14	53986001	53987000	ATP5F1CP1	
14	54096001	54097000	LOC105370507	
14	54789001	54790000	SAMD4A	
14	54982001	54983000	WDHD1	Epigenetic
14	55015001	55016000	WDHD1;RPSAP13	Epigenetic
14	58148001	58149000	ARMH4;UBA52P3	
14	58521001	58522000	KIAA0586;HNRNPCP1	
14	59463001	59464000	GPR135;L3HYPDH	Signaling;Metabolism
14	59729001	59730000	RTN1	
14	61040001	61041000	SLC38A6;LOC101927756	Transport
14	61597001	61598000	FLJ22447	
14	61615001	61616000	FLJ22447	
14	61785001	61786000	SNAPC1	
14	62709001	62710000	KCNH5	Transport
14	65263001	65264000	RPL21P7;RPL36AP2	
14	65404001	65405000	FUT8;FUT8-AS1	Golgi
14	65496001	65497000	FUT8	Golgi
14	67504001	67505000	TMEM229B	
14	67931001	67932000	RAD51B	Transcription
14	68521001	68522000	RAD51B	Transcription
14	68560001	68561000	RAD51B	Transcription

14	68974001	68975000	ACTN1;ACTN1-DT	
14	69349001	69350000	GALNT16	Golgi
14	70012001	70013000	SMOC1	Signaling
14	70555001	70557000	ADAM20;LOC100420563;ADAM20;LOC100420563	Protease;Protease
14	70675001	70676000	TTC9	Transcription
14	71646001	71647000	SIPA1L1	Signaling
14	71684001	71685000	SIPA1L1	Signaling
14	71982001	71983000	RGS6	
14	73112001	73113000	RBM25	
14	73259001	73260000	PAPLN	Protease
14	73415001	73416000	NUMB	Cytoskeleton
14	73622001	73623000	ACOT6;NDUFB8P1	Metabolism
14	73973001	73974000	COQ6;ENTPD5	Metabolism;Signaling
14	74106001	74107000	LIN52;LOC105370563	
14	74224001	74225000	LOC105370564	
14	74292001	74293000	ABCD4	Transport
14	74908001	74909000	DLST;RPS6KL1	Transport;Signaling
14	74935001	74936000	PGF;LOC107984689	Growth Factors
14	75026001	75027000	MLH3;RNU6-689P	Transcription
14	75567001	75568000	LOC107984652;LOC107984653;LOC102724153	
14	76002001	76003000	IFT43	
14	76019001	76020000	IFT43;LOC100506576	
14	77271001	77272000	NGB;MIR1260A;POMT2	Transport;Transport
14	77793001	77794000	ADCK1	Transport
14	77808001	77809000	ADCK1	Transport
14	77909001	77910000	ADCK1	Transport
14	78467001	78468000	NRXN3	
14	78691001	78692000	NRXN3	
14	79345001	79346000	NRXN3	
14	79818001	79819000	NRXN3	
14	79994001	79995000	LOC105370590	
14	80154001	80155000	LOC105370591	
14	80643001	80644000	CEP128	
14	80782001	80783000	CEP128	
14	81131001	81132000	TSHR;LOC101928462	Signaling
14	81143001	81144000	TSHR;LOC101928462	Signaling
14	81405001	81406000	STON2;DYNLL1P2	Transport
14	81664001	81665000	EEF1A1P2	
14	83910001	83911000	LINC02305	
14	84176001	84177000	MTND4P33;MTND6P27;MTCYBP27	
14	88448001	88449000	SPATA7	
14	88631001	88632000	ZC3H14;EML5	
14	89272001	89273000	FOXN3	
14	90099001	90100000	KCNK13;GLRXP2	Transport
14	90276001	90277000	PSMC1;NRDE2	Protease
14	90774001	90775000	TTC7B;RPS18P2	
14	90794001	90795000	TTC7B	
14	90831001	90832000	LINC02321	
14	90951001	90952000	RPS6KA5	Golgi
14	91143001	91144000	DGLUCY	Metabolism
14	91355001	91356000	CCDC88C	Transport
14	91834001	91835000	TC2N	
14	91867001	91868000	TC2N;FBLN5	Extracellular Matrix
14	92088001	92089000	ATXN3	Epigenetic
14	92557001	92558000	RIN3	Transcription
14	92635001	92637000	RIN3;RIN3	Transcription;Transcription
14	93076001	93077000	ITPK1;ITPK1-AS1	Signaling
14	94164001	94165000	LOC105370634;PPP4R4	Signaling
14	94295001	94296000	SERPINA10;SERPINA6	Protease; Proteolysis
14	94590001	94592000	SERPINA5;LOC112268127;SERPINA5;LOC112268127	Protease; Proteolysis;Protease; Proteolysis
14	95221001	95222000	CLMN	
14	96240001	96241000	BDKRB2	Signaling
14	96739001	96740000	LINC02299	
14	97624001	97625000	LINC02291	
14	99147001	99148000	LOC105370659	
14	99413001	99414000	SETD3	Epigenetic

14	99452001	99453000	SETD3;RNU6-91P	Epigenetic
14	99841001	99842000	EML1	
14	99952001	99953000	EML1;LOC102724682;RNU1-47P	
14	100463001	100464000	WDR25	Cytoskeleton
14	100717001	100718000	LOC105370669;DLK1	Signaling
14	101072001	101073000	MIR377;MIR541;MIR409;MIR412;MIR369;MIR410;MIR656;MEG9;LOC105370670	
14	101076001	101077000	MIR656;MEG9;LOC105370670	
14	101352001	101353000	LOC107984697;LOC105370671	
14	101473001	101474000	LOC105370672	
14	101519001	101520000	LOC105370673	
14	101936001	101937000	PPP2R5C	Signaling
14	102022001	102023000	DYNC1H1	Cytoskeleton
14	102261001	102262000	MOK	Signaling
14	102297001	102298000	MOK	Signaling
14	102713001	102714000	RCOR1	
14	102900001	102901000	TRAF3	Cytoskeleton
14	103085001	103086000	LBHD2;EXOC3L4	Transport
14	103116001	103117000	EXOC3L4;LINC00677;TNFAIP2	Transport
14	103297001	103298000	LOC105370686	
14	103336001	103337000	LOC105370687;EIF5;SNORA28	Translation
14	103685001	103686000	KLC1	Cytoskeleton
14	103937001	103938000	TDRD9;RD3L	Transcription
14	104008001	104009000	TDRD9;RN7SL634P	Transcription
14	104725001	104726000	INF2;ADSS1	Metabolism
14	104932001	104933000	PLD4;AHNAK2	Metabolism
14	105142001	105143000	JAG2;MIR6765	
14	105561001	105563000	LOC105370698;LOC105370698	;
14	105577001	105578000	IGH;IGHA2	Immune
14	105685001	105686000	IGH;LOC112268138	
14	105693001	105694000	IGH	
14	105716001	105717000	IGH;IGHA1;IGHEP1	Immune
14	105855001	105856000	IGH;IGHD;IGHM;MIR4539;MIR4507;MIR4538;MIR4537;IGHJ6;IGHJ3P;IGHJ5;IGHJ4;IGHJ3;IGHJ2P;IGHJ2;IGHJ1;IGHD7-27;IGHJ1P	Immune
14	105896001	105897000	IGH;IGHD3-22;IGHD2-21;IGHD1-20;IGHD6-19;IGHD5-18;IGHD4-17;IGHD3-16;IGHD2-15;IGHD1-14;IGHD6-13;IGHD5-12;IGHD4-11;IGHD3-10;IGHD3-9	
14	106401001	106402000	IGH;IGHV3-36;IGHV3-37;IGHV3-38	Immune
14	106479001	106483000	IGH;IGHV3-43;IGHVII-43-1;IGHVIII-44;LINC00221;IGHVIV-44-1;IGH;IGHV3-43;IGHVII-43-1;IGHVIII-44;LINC00221;IGHVIV-44-1;IGH;IGHVII-43-1;IGHVIII-44;LINC00221;IGHVIV-44-1;IGH;IGHVII-43-1;IGHVIII-44;LINC00221;IGHVIV-44-1	Immune;Immune;;
15	20071001	20072000	BCAR1P1	
15	20295001	20296000	CHEK2P2	
15	20432001	20434000	HERC2P3;HERC2P3	Transcription;Transcription
15	20535001	20536000	LOC112268160;GOLGA6L6	Cytoskeleton
15	20552001	20553000	LOC100631265;GOLGA8CP	Transport
15	20741001	20742000	NBEAP1;RNU6-498P	
15	20941001	20942000	NF1P1;MIR5701-1;LINC01193	
15	21157001	21158000	BMS1P16	
15	21262001	21263000	NBEAP4;LOC105370715;LOC105370714	
15	21393001	21394000	GRAMD4P6	
15	22272001	22273000	REREP3	
15	22563001	22564000	HERC2P2	
15	22611001	22612000	LOC101927846;RN7SL495P;GOLGA8IP	Transport
15	22704001	22705000	LOC100133165	
15	22862001	22863000	NIPA2;CYFIP1	Cytoskeleton
15	22900001	22901000	CYFIP1	Cytoskeleton
15	23077001	23078000	ELMO2P1	
15	23130001	23131000	LOC101060118;GOLGA6L1	Cytoskeleton
15	23396001	23397000	LOC105370728	
15	24677001	24678000	NPAP1	Transport
15	24827001	24828000	SNHG14;SNRPN	Translation
15	25189001	25190000	SNHG14;SNORD115@;SNORD115-6;SNORD115-7;SNORD115-8;SNORD115-9;SNORD115-10;SNORD115-11;SNORD115-12;SNORD115-13;SNORD115-14;SNORD115-15;SNORD115-16	
15	26434001	26435000	LINC02248;LOC105370740	
15	27153001	27154000	GABRG3;GABRG3-AS1	Ion Channel
15	28294001	28295000	HERC2	Transcription

15	28317001	28318000	HERC2;RPL41P2	Transcription
15	28320001	28321000	HERC2;RPL41P2	Transcription
15	28470001	28471000	LOC101059997	
15	28560001	28562000	LOC100132202;LOC105369220;LOC100132202;LOC105369220	;
15	28833001	28834000	PDCD6IPP2;LOC100129687;GOLGA6L7	Cytoskeleton
15	28897001	28898000	APBA2	Transport
15	28924001	28925000	APBA2	Transport
15	29369001	29370000	FAM189A1	
15	29384001	29385000	FAM189A1	
15	29556001	29557000	FAM189A1	
15	30495001	30496000	LOC100288203	
15	30748001	30749000	LOC100288637	
15	31088001	31089000	TRPM1	Transport
15	31159001	31160000	TRPM1	Transport
15	32182001	32183000	CHRNA7;LOC102724078	Ion Channel
15	32734001	32735000	GREM1	
15	33159001	33160000	FMN1	
15	34174001	34175000	KATNBL1	
15	34247001	34248000	SLC12A6	Transport
15	34421001	34422000	GOLGA8A	Transport
15	34896001	34897000	AQR	
15	38516001	38517000	RASGRP1;LOC105370774	Transcription
15	40027001	40028000	EIF2AK4;SRP14	Signaling;Metabolism
15	40166001	40167000	BUB1B;LOC107984763	Signaling
15	40398001	40399000	KNSTRN;IVD	Metabolism
15	40875001	40876000	RHOV	Signaling
15	41171001	41172000	CIBAR1P1	
15	41239001	41240000	EXD1;CHP1	
15	41311001	41312000	OIP5-AS1;OIP5	
15	41379001	41380000	NUSAP1;NDUFAF1	
15	41541001	41542000	RPAP1	
15	41552001	41554000	RPAP1;ELOCP2;TYRO3;RPAP1;ELOCP2;TYRO3	Receptor;Receptor
15	41674001	41675000	MGA	Transcription
15	41742001	41743000	MGA	Transcription
15	42037001	42038000	PLA2G4E;LOC105370793	Metabolism
15	42726001	42727000	STARD9;CDAN1	
15	43883001	43884000	FRMD5;PIN4P1	
15	43894001	43895000	FRMD5	
15	43958001	43959000	FRMD5	
15	44824001	44825000	SORD2P	
15	45352001	45353000	RNU6-953P;GATM	Transport
15	45376001	45377000	GATM	Transport
15	45461001	45462000	LOC105376714	
15	48246001	48247000	SLC12A1;LOC107984758	Transport
15	49784001	49785000	RLIMP3	
15	49930001	49931000	ATP8B4	Transport
15	50620001	50621000	TRPM7	Transport
15	51285001	51286000	MIR4713HG;CYP19A1	Metabolism
15	51954001	51955000	LOC112268148;LEO1	Transcription
15	51966001	51967000	LEO1	Transcription
15	52394001	52395000	MYO5A	Cytoskeleton
15	52588001	52589000	FAM214A	
15	55097001	55098000	LOC105370829	
15	56461001	56462000	MNS1	Development
15	57055001	57056000	TCF12	Transcription
15	57305001	57306000	LINC00926	
15	57447001	57448000	CGNL1	
15	57784001	57785000	LOC105370834	
15	58544001	58545000	LIPC	Metabolism
15	59251001	59252000	MYO1E	Cytoskeleton
15	59255001	59256000	MYO1E	Cytoskeleton
15	59543001	59544000	RPL21P117	
15	60432001	60433000	ICE2	
15	60546001	60547000	RORA-AS1;RORA	Transcription
15	60904001	60905000	RORA	Transcription
15	61061001	61062000	RORA;LOC107984805	Transcription

15	62019001	62020000	VPS13C	Transport
15	62761001	62762000	TLN2	
15	64085001	64087000	CIAO2A;SNX1;CIAO2A;SNX1	Cytoskeleton;Cytoskeleton
15	64295001	64296000	CSNK1G1	Signaling
15	64849001	64850000	PLEKHO2	
15	65248001	65249000	LOC112268147;PARP16	
15	66080001	66081000	MEGF11	Extracellular Matrix
15	66189001	66190000	MEGF11	Extracellular Matrix
15	66263001	66264000	MEGF11	Extracellular Matrix
15	66333001	66334000	DIS3L;TIPIN	Transcription;Transcription
15	66808001	66809000	LETM1P1	
15	66922001	66923000	LINC02206	
15	68555001	68556000	CORO2B	Cytoskeleton
15	68989001	68990000	NOX5	Metabolism
15	69462001	69463000	RPLP1;LINC02896	Translation
15	69621001	69622000	PCAT29	
15	69651001	69652000	PCAT29	
15	70193001	70194000	RNU6-745P	
15	70302001	70303000	LOC105370878	
15	71199001	71200000	THSD4;THSD4-AS1	Protease
15	72289001	72290000	LOC105370887;CELF6	
15	72308001	72309000	CELF6	
15	72383001	72384000	HEXA;HEXA-AS1;RPL12P35	Metabolism
15	72616001	72617000	LOC646665;LINC02259	
15	72789001	72790000	ADPGK;ADPGK-AS1	Signaling
15	73223001	73224000	NEO1	
15	73755001	73756000	INSYN1	
15	74766001	74767000	CYP1A2	Metabolism
15	74888001	74889000	MPI	Metabolism
15	74935001	74936000	COX5A	Metabolism
15	74959001	74960000	RPP25;LOC107984731	Translation
15	75409001	75410000	SIN3A	Epigenetic
15	76710001	76711000	SCAPER	
15	76953001	76954000	RCN2	Signaling
15	77526001	77527000	HMG20A;LOC101929457	
15	77618001	77619000	LOC105370906;LINGO1	Receptor
15	77737001	77738000	LINGO1	Receptor
15	78162001	78163000	IDH3A;ACSBG1	Metabolism;Metabolism
15	79024001	79025000	RASGRF1	Transcription
15	79051001	79052000	RASGRF1	Transcription
15	79364001	79365000	TMED3	Transport
15	79555001	79556000	TFDP1P3	
15	79918001	79920000	ST20-MTHFS;ST20;ST20-AS1;ST20-MTHFS;ST20;ST20-AS1	;
15	80084001	80085000	ZFAND6	
15	80410001	80411000	ARNT2	Transcription
15	80905001	80906000	CEMIP;LOC107984749	
15	80959001	80960000	CEMIP;MESD	
15	81325001	81326000	STARD5;TMC3-AS1;TMC3	
15	81424001	81425000	TMC3-AS1	
15	81699001	81700000	LOC105370921	
15	82078001	82079000	LINC01583	
15	82697001	82699000	CPEB1-AS1;AP3B2;LOC100421235;CPEB1-AS1;AP3B2;LOC100421235	Transport;Transport
15	83554001	83555000	SH3GL3	
15	83724001	83725000	ADAMTSL3;RNU6-401P	Protease
15	83864001	83865000	ADAMTSL3	Protease
15	83895001	83896000	ADAMTSL3	Protease
15	84397001	84398000	LOC105376722;LOC100288367;RN7SL417P;CSPG4P5	
15	84605001	84606000	ZSCAN2;LOC105370947	Transcription
15	84766001	84767000	ZNF592	
15	85798001	85799000	KLHL25	
15	86380001	86381000	AGBL1	Protease
15	87360001	87361000	LOC105370956	
15	87881001	87882000	NTRK3	Receptor
15	88212001	88213000	NTRK3	Receptor
15	88243001	88244000	NTRK3;NTRK3-AS1	Receptor
15	88302001	88303000	LOC105370958	

15	88900001	88901000	HAPLN3;MFGE8	Extracellular Matrix;Metabolism
15	88982001	88983000	LOC100129942	
15	89811001	89812000	ANPEP;LOC105370965	Protease
15	90666001	90667000	CRTC3-AS1;LINC01585	
15	90838001	90839000	LOC105370969	
15	90944001	90946000	UNC45A;RCCD1-AS1;RCCD1;UNC45A;RCCD1-AS1;RCCD1	;
15	91025001	91026000	VPS33B;VPS33B-DT;LOC390638	Transport
15	92571001	92572000	LINC00930	
15	92661001	92662000	FAM174B	
15	92711001	92712000	FAM174B;HMGN1P38;RPL31P6	
15	92798001	92799000	LOC101926994;ASB9P1	
15	92873001	92874000	CHASERR	
15	93242001	93243000	LOC105370982	
15	94852001	94853000	LOC440311	
15	95052001	95053000	LOC105370991	
15	95157001	95158000	LOC105370991;LOC105370990	
15	96256001	96257000	NR2F2-AS1	
15	97999001	98000000	LOC105371009;LINC02251	
15	98459001	98460000	FAM169B;LOC100421087	
15	98990001	98991000	PGPEP1L	Protease
15	99145001	99146000	SYNM;TTC23	
15	99253001	99254000	TTC23;LRRC28;HSP90B2P	Cytoskeleton
15	100187001	100188000	ADAMTS17	Protease
15	100441001	100442000	CERS3-AS1;CERS3	
15	100554001	100555000	CERS3;PRKXP1;RNU6-322P;LOC102723335;RNU6-181P	
15	101130001	101132000	LOC105371026;LOC105371026	;
15	101260001	101261000	CHSY1;LOC107984727;SELENOS	Golgi
16	254001	256000	FAM234A;FAM234A	;
16	288001	289000	ARHGDIG;PDIA2;AXIN1	Signaling;Transcription;Cytoskeleton
16	341001	342000	AXIN1	Cytoskeleton
16	367001	369000	MRPL28;PGAP6;MRPL28;PGAP6	Translation;Cytoskeleton;Translation;Cytoskeleton
16	554001	555000	CAPN15;PRR35	Protease
16	640001	641000	WFIKKN1;METTL26;TRG-CCC2-2;LOC100287175;MCRIP2;LOC105371038;WDR90	
16	718001	719000	METRN;ANTKMT;CCDC78;HAGHL	
16	738001	739000	HAGHL;CIAO3	Metabolism
16	812001	813000	PRR25	
16	858001	860000	LMF1;LMF1	;
16	922001	923000	LMF1;LMF1-AS1	
16	938001	939000	LMF1;LMF1-AS1	
16	952001	954000	LMF1;LMF1	;
16	1092001	1093000	C1QTNF8	
16	1186001	1187000	CACNA1H	Transport
16	1196001	1197000	CACNA1H	Transport
16	1345001	1346000	BAIAP3;TSR3;GNPTG	Signaling
16	1367001	1368000	GNPTG;UNKL;TJP1P1	Signaling
16	1405001	1407000	UNKL;LOC101929440;UNKL;LOC101929440	;
16	1738001	1739000	MAPK8IP3;MIR3177	Cytoskeleton
16	1740001	1741000	MAPK8IP3;MIR3177	Cytoskeleton
16	2024001	2025000	NPW;SLC9A3R2	
16	2036001	2037000	SLC9A3R2;NTHL1	Epigenetic
16	2387001	2388000	ABCA17P	
16	2493001	2494000	TBC1D24	Signaling
16	2568001	2569000	PDPK1	Signaling
16	2635001	2636000	LOC652276;PDPK2P;FLJ42627	Signaling
16	2693001	2694000	KCTD5	
16	2734001	2735000	SRRM2-AS1	
16	2766001	2767000	SRRM2;ELOB	Transcription
16	2791001	2792000	PRSS33;SNORA3C;PRSS41	Protease
16	3203001	3204000	OR1F1;TRR-CCT5-1;TRK-CTT14-1	Receptor
16	3293001	3294000	ZNF263;TIGD7	Transcription;Epigenetic
16	3466001	3467000	NAA60	Metabolism
16	3551001	3552000	NLRC3;LOC101929732	Cytoskeleton
16	3648001	3649000	DNASE1;TRAP1	Signaling
16	3664001	3665000	DNASE1;TRAP1	Signaling
16	3667001	3668000	DNASE1;TRAP1	Signaling
16	3751001	3752000	CREBBP	Epigenetic

16	3969001	3970000	ADCY9	
16	3988001	3989000	ADCY9	
16	4462001	4463000	DNAJA3;NMRAL1	Transcription
16	4854001	4855000	GLYR1;UBN1	Metabolism;Cytoskeleton
16	4970001	4971000	SEC14L5	
16	5028001	5029000	SEC14L5;NAGPA;NAGPA-AS1	
16	5618001	5619000	RBFOX1;LINC01570	Translation
16	5653001	5654000	RBFOX1	Translation
16	5730001	5731000	RBFOX1	Translation
16	6118001	6119000	RBFOX1	Translation
16	6759001	6760000	RBFOX1	Translation
16	7158001	7159000	RBFOX1	Translation
16	7361001	7362000	RBFOX1	Translation
16	7486001	7487000	RBFOX1	Translation
16	8562001	8563000	TMEM114	
16	8591001	8592000	TMEM114	
16	8774001	8775000	ABAT	Metabolism
16	8857001	8859000	PMM2;LOC100130283;CARHSP1;PMM2;LOC100130283;CARHSP1	Metabolism;Metabolism;Metabolism;Metabolism
16	8887001	8888000	LITAFD;USP7	Cytoskeleton;Protease
16	9154001	9155000	RPL21P119	
16	9442001	9443000	LOC101927026;LINC01177;LINC01195	
16	9637001	9638000	LOC101927026	
16	9658001	9659000	LOC101927026	
16	9818001	9819000	GRIN2A;LOC105371077	Receptor
16	9892001	9893000	GRIN2A	Receptor
16	10487001	10488000	ATF7IP2;RNU6-633P	Transcription
16	10667001	10668000	TEKT5	Cytoskeleton
16	10685001	10686000	TEKT5;LOC105371079	Cytoskeleton
16	11266001	11267000	LOC105371082;SOCS1;TNP2;PRM3;PRM2	Signaling;Epigenetic;Epigenetic
16	11363001	11364000	LOC105371082;LOC400499	Transport
16	11459001	11460000	LOC400499;LOC101927131	Transport
16	11754001	11755000	ZC3H7A	Metabolism
16	12266001	12267000	SNX29	Cytoskeleton
16	12292001	12293000	SNX29	Cytoskeleton
16	13090001	13091000	SHISA9	
16	13956001	13957000	ERCC4	Transcription
16	14312001	14313000	MIR193BHG;MIR193B;MIR365A	
16	15124001	15125000	PDXDC1;NPIPP1;PKD1P6-NPIPP1;PKD1P6;MIR6511B2	
16	15132001	15133000	PDXDC1;NPIPP1;PKD1P6-NPIPP1;PKD1P6;MIR6511B2	
16	15151001	15152000	PDXDC1;PKD1P6;MIR3180-4	
16	15291001	15293000	LOC105371097;LOC105371097	;
16	15670001	15671000	NDE1	
16	15789001	15790000	MYH11	
16	16035001	16036000	ABCC1	Transport
16	16187001	16188000	ABCC6	Transport
16	16351001	16352000	PKD1P1;PKD1P2	
16	16397001	16398000	NPIPA7;LOC105371098	
16	16478001	16479000	LOC105371105	
16	18492001	18493000	MIR3670-4;LOC105379460;MIR3179-4;NOMO2	
16	20306001	20307000	GP2	Receptor
16	21073001	21074000	DNAH3	Cytoskeleton
16	21679001	21680000	OTOA	Cytoskeleton
16	21853001	21854000	LOC112268174;NPIPB4	
16	22033001	22034000	MOSMO	
16	22340001	22341000	POLR3E;CDR2	Transcription
16	22498001	22499000	SMG1P1;NPIPB5	
16	22540001	22541000	NPIPB5;LOC105371131;OTOAP1	
16	22606001	22607000	LOC112268175	
16	22827001	22828000	HS3ST2	Transport
16	23408001	23409000	COG7;RN7SKP23	
16	23552001	23553000	EARS2;UBFD1	Translation
16	23866001	23867000	PRKCB	Signaling
16	24477001	24478000	LOC105371143	
16	24568001	24569000	RBBP6	Proteolysis
16	24706001	24707000	TNRC6A	Metabolism
16	24834001	24835000	TNRC6A;LOC107984825	Metabolism

16	25065001	25066000	LINC02175;SCML2P2;LOC100421169	
16	26342001	26343000	LOC102723536	
16	27040001	27041000	LOC105371153	
16	27138001	27139000	EEF1A1P38	
16	27344001	27346000	IL4R;IL4R	Receptor;Receptor
16	27533001	27534000	GTF3C1	Transcription
16	27791001	27792000	GSG1L	Cytoskeleton
16	27870001	27871000	GSG1L;RNU6-159P	Cytoskeleton
16	28101001	28102000	XPO6;TPRKBP2	
16	28295001	28296000	SBK1	Signaling
16	28341001	28342000	LOC105369182;NPIPB6	
16	28642001	28643000	NPIPB8	
16	28745001	28747000	EIF3C;NPIPB9;LOC105379464;PAWRP2;NPIPB9;LOC105379464;PAWRP2	Translation;
16	28838001	28839000	ATXN2L;TUFM;MIR4721;SH2B1	Metabolism;Translation;Cytoskeleton
16	29153001	29154000	LOC107984832	
16	29198001	29199000	LOC107984832	
16	29298001	29300000	LOC105371159;LOC107984833;SNX29P2;LOC105371159;LOC107984833;SNX29P2	Cytoskeleton;Cytoskeleton
16	29378001	29379000	NPIPB11	
16	29383001	29384000	NPIPB11	
16	29843001	29844000	MVP	Cytoskeleton
16	30251001	30252000	LOC101929894;NPIPB13	
16	30661001	30663000	PRR14;FBRS;PRR14;FBRS	;
16	30738001	30739000	SRCAP;TMEM265;PHKG2	Signaling
16	30807001	30808000	RNF40	
16	30979001	30980000	SETD1A;HSD3B7;STX1B	Epigenetic;Metabolism;Transcription
16	31007001	31009000	STX1B;STX1B	Transcription;Transcription
16	31040001	31041000	STX4;LOC101928762	Transcription
16	31175001	31176000	NDUFA3P6;FUS	Metabolism
16	31239001	31240000	TRIM72	Proteolysis
16	31322001	31323000	ITGAM	Extracellular Matrix
16	32116001	32118000	LOC101060104;LOC101060104	;
16	32210001	32211000	ABHD17AP8	
16	33105001	33106000	HERC2P8	
16	33125001	33126000	HERC2P8;LOC107987232	
16	33554001	33555000	LOC101929031	
16	33614001	33615000	LOC102724181	
16	34162001	34163000	LINC00273;LOC110262331;RNA5-8SP2;MIR9901	
16	34277001	34278000	BCLAF1P2	
16	35140001	35141000	VN1R69P;LOC105371197	
16	35251001	35252000	LOC105371200;FRG2JP;RARRES2P6;AGGF1P8	
16	46693001	46694000	VPS35;ORC6;MYLK3	Transport;Cell Cycle;Signaling
16	46915001	46916000	GPT2	Metabolism
16	47350001	47351000	ITFG1	
16	47552001	47553000	PHKB;LOC100420642	Signaling
16	48149001	48150000	ABCC12	Transport
16	48241001	48242000	ABCC11;LONP2	Transport;Protease
16	48394001	48396000	SIAH1;SIAH1	Proteolysis;Proteolysis
16	48501001	48502000	MOCS1P1	
16	49288001	49289000	CBLN1;LOC102724859	
16	49489001	49490000	ZNF423	Transcription
16	50117001	50118000	HEATR3	
16	51287001	51288000	UNGP1	
16	52591001	52592000	CASC16;LOC105371265	
16	53733001	53734000	FTO	Metabolism
16	53791001	53792000	FTO	Metabolism
16	54122001	54124000	FTO;FTO	Metabolism;Metabolism
16	54255001	54256000	LINC02169	
16	56040001	56041000	LOC107984815	
16	56340001	56341000	GNAO1	Signaling
16	56575001	56576000	MT4	
16	56588001	56589000	MT3	
16	57246001	57247000	RSPRY1;ARL2BP;PLLP	Transport
16	57282001	57283000	PLLP	Transport
16	57650001	57652000	ADGRG1;ADGRG1	Signaling;Signaling
16	57761001	57762000	KATNB1;KIFC3	Cytoskeleton
16	57795001	57796000	KIFC3	Cytoskeleton

16	58290001	58292000	CCDC113;PRSS54;CCDC113;PRSS54	Protease;Protease
16	58633001	58634000	CNOT1	Translation
16	58669001	58670000	SLC38A7	Transport
16	58766001	58767000	LOC107984867;RN7SL143P;GEMIN8P2	
16	62725001	62726000	LOC102723560	
16	66670001	66671000	CMTM4	Transport
16	66975001	66976000	CES3	Metabolism
16	67211001	67212000	ELMO3;MIR328;LRRC29	Cytoskeleton
16	67398001	67399000	TPPP3;RNU1-123P;ZDHHC1	Cytoskeleton
16	67422001	67423000	ZDHHC1;HSD11B2	Metabolism
16	67601001	67602000	CTCF	Transcription
16	67788001	67789000	RANBP10	Cytoskeleton
16	67816001	67817000	RANBP10;TSNAXIP1	Cytoskeleton
16	68007001	68008000	DPEP2;KARS1P3;DPEP2NB;RNU6-359P	Protease
16	68101001	68102000	NFATC3	Transcription
16	68316001	68317000	SLC7A6OS;PRMT7	Golgi
16	69015001	69016000	TANGO6;RNU6-898P;LOC101060098	
16	69857001	69858000	WWP2	Proteolysis
16	69975001	69976000	CLEC18A;PDXDC2P-NPIPB14P;NPIPB14P	Immune
16	70116001	70117000	PDPR	Metabolism
16	70445001	70446000	ST3GAL2;RPS27P26;RNU6-23P;FCSK	Transport;Signaling
16	70743001	70744000	VAC14	Cytoskeleton
16	70846001	70847000	HYDIN;RNU6ATAC25P	
16	71544001	71545000	CHST4	Transport
16	71855001	71856000	ATXN1L;ZNF821	Transcription
16	72564001	72565000	LINC01572	
16	73205001	73206000	ZFHX3	Transcription
16	73501001	73502000	ZFHX3	Transcription
16	73554001	73555000	ZFHX3	Transcription
16	73563001	73564000	ZFHX3	Transcription
16	73602001	73603000	ZFHX3	Transcription
16	73895001	73896000	ZFHX3	Transcription
16	74361001	74363000	PDPR2P;PDPR2P	;
16	74365001	74366000	PDPR2P;NPIPB15	
16	74444001	74445000	LOC107984827;GLG1	
16	74454001	74455000	GLG1;RNU6-237P	
16	74461001	74462000	GLG1;RNU6-237P	
16	75053001	75054000	ZNRF1	Proteolysis
16	75272001	75273000	BCAR1;LOC105371344;LOC105371345	
16	75383001	75384000	CFDP1	
16	75488001	75489000	CHST6	Transport
16	78386001	78387000	WWOX	Metabolism
16	78520001	78521000	WWOX	Metabolism
16	78567001	78568000	WWOX	Metabolism
16	79082001	79083000	WWOX;LOC107984806	Metabolism
16	79161001	79162000	WWOX	Metabolism
16	79272001	79273000	MAF;RNA5SP431	Transcription
16	79310001	79311000	MAF	Transcription
16	80334001	80335000	DYNLRB2-AS1	
16	80568001	80569000	DYNLRB2-AS1;LINC01227	
16	80696001	80697000	CDYL2	
16	81133001	81134000	PKD1L2	Transport
16	81138001	81139000	PKD1L2	Transport
16	81373001	81374000	GAN	Cytoskeleton
16	81383001	81384000	GAN;MIR4720	Cytoskeleton
16	81646001	81648000	CMIP;CMIP	;
16	81650001	81651000	CMIP	
16	84257001	84258000	RNA5SP433	
16	84326001	84327000	WFDC1	
16	84380001	84382000	ATP2C2;ATP2C2	Transport;Transport
16	84433001	84434000	ATP2C2	Transport
16	84718001	84719000	USP10	Protease
16	84980001	84981000	ZDHHC7	
16	85066001	85067000	KIAA0513	
16	85144001	85145000	LINC02139;LOC105371382	
16	85264001	85265000	GSE1	

16	85503001	85504000	GSE1	
16	85621001	85622000	GSE1	
16	85796001	85797000	EMC8;LOC101928557;COX4I1	Metabolism
16	85826001	85827000	RPL10AP12	
16	85935001	85936000	LINC02132;LOC105371388	
16	86224001	86225000	LINC01081	
16	86742001	86743000	LINC02188	
16	86909001	86910000	LOC105371393	
16	87279001	87280000	LOC101928682	
16	87371001	87372000	FBXO31	Proteolysis
16	87624001	87625000	JPH3	
16	87748001	87749000	KLHDC4	
16	87984001	87985000	BANP	
16	88196001	88197000	LINC02182	
16	88380001	88382000	ZNF469;ZNF469	;
16	88465001	88466000	ZFPM1;MIR5189	Transcription
16	88521001	88522000	ZFPM1;LOC107984890;ZFPM1-AS1	Transcription
16	88827001	88828000	GALNS;LOC107987238	Metabolism
16	89149001	89150000	ACSF3;LINC00304	Metabolism
16	89207001	89208000	SLC22A31;ZNF778	Transport;Transcription
16	89596001	89598000	CPNE7;CPNE7	;
16	89619001	89620000	DPEP1	Protease
16	89859001	89860000	SPIRE2;LOC105371419	Cytoskeleton
16	89862001	89863000	SPIRE2;LOC105371419;LOC112268179	Cytoskeleton
16	89960001	89961000	DEF8;SNORA119;CENPBD1	
16	89980001	89981000	CENPBD1;AFG3L1P	
17	102001	103000	LOC101929823;GTF2IP17;LOC101929828	
17	157001	158000	DOC2B	
17	161001	162000	DOC2B	
17	251001	252000	RPH3AL	
17	306001	307000	RPH3AL	
17	545001	546000	VPS53	Transport
17	579001	580000	VPS53	Transport
17	759001	760000	GEMIN4;DBIL5P;GLOD4	
17	761001	763000	GEMIN4;DBIL5P;GLOD4;GEMIN4;DBIL5P;GLOD4	;
17	795001	796000	GLOD4;MRM3;NXN	Epigenetic;Metabolism
17	800001	801000	MRM3;NXN	Epigenetic;Metabolism
17	1117001	1118000	ABR	Signaling
17	1424001	1425000	CRK	Cytoskeleton
17	1584001	1585000	SLC43A2	
17	1678001	1679000	PRPF8	Translation
17	1849001	1850000	RPA1;LOC642502	Transcription
17	1877001	1878000	RPA1	Transcription
17	2038001	2039000	DPH1;OVCA2	Metabolism
17	2119001	2120000	SMG6;MCUR1P1	Metabolism
17	2127001	2128000	SMG6;MCUR1P1	Metabolism
17	2558001	2559000	RN7SL33P	
17	2689001	2690000	PAFAH1B1;RN7SL608P;LOC105371490;CLUH;MIR6776	Translation
17	2714001	2716000	CLUH;LOC105371592;CCDC92B;CLUH;LOC105371592;CCDC92B	Translation;Translation
17	2760001	2761000	RAP1GAP2	Signaling
17	2920001	2921000	RAP1GAP2	Signaling
17	2954001	2955000	RAP1GAP2;LOC101927911	Signaling
17	3262001	3263000	OR1D3P	
17	3350001	3351000	OR3A2	Receptor
17	3513001	3515000	SPATA22;ASPA;TRPV3;SPATA22;TRPV3	Transport;Transport
17	3633001	3634000	SHPK;CTNS;LOC105371493	Metabolism;Transport
17	3869001	3870000	CAMKK1	Signaling
17	4023001	4024000	ZZEF1	
17	4992001	4994000	CAMTA2;CAMTA2-AS1;INCA1;KIF1C;CAMTA2;CAMTA2-AS1;INCA1;KIF1C	Transcription;Cytoskeleton;Transcription;Cytoskeleton
17	5000001	5001000	CAMTA2-AS1;INCA1;KIF1C	Cytoskeleton
17	5233001	5234000	ZNF594-DT;SCIMP	
17	5431001	5432000	RPAIN;C1QBP;DHX33	Transcription
17	6112001	6113000	WSCD1	
17	6418001	6419000	AIPL1	Transcription
17	6886001	6887000	ALOX12P2	
17	6902001	6903000	ALOX12P2	

17	7033001	7034000	BCL6B;SLC16A13;SLC16A11	Transcription;Transport
17	7085001	7086000	CLEC10A	Transport
17	7192001	7194000	DLG4;DLG4	Cytoskeleton;Cytoskeleton
17	7213001	7214000	DLG4;ACADVL;MIR324	Cytoskeleton;Metabolism
17	7284001	7285000	SLC2A4;YBX2	
17	7596001	7597000	MPDU1;SOX15;FXR2	Development;Translation
17	7804001	7805000	DNAH2	Cytoskeleton
17	8134001	8135000	HES7;TRG-GCC2-6;LOC105376799;TRS-CGA1-1;TRT-AGT5-1;PER1;MIR6883	Transcription;Transcription
17	8511001	8512000	MYH10;LOC102724262;RNU7-43P	
17	8755001	8756000	LOC105371529;SPDYE4;LOC105371530	
17	8811001	8812000	PIK3R6	Signaling
17	8851001	8852000	PIK3R6	Signaling
17	9126001	9127000	NTN1	Extracellular Matrix
17	9246001	9247000	NTN1;LOC105371532;LOC105371531;STX8	Extracellular Matrix;Transcription
17	9397001	9398000	STX8	Transcription
17	9519001	9520000	STX8	Transcription
17	9636001	9637000	CFAP52;USP43;LOC107985011	Protease
17	9724001	9725000	USP43	Protease
17	10052001	10053000	GAS7	Cytoskeleton
17	10062001	10063000	GAS7	Cytoskeleton
17	10126001	10127000	GAS7	Cytoskeleton
17	10142001	10144000	GAS7;GAS7	Cytoskeleton;Cytoskeleton
17	10400001	10401000	MYHAS;MYH8	
17	11151001	11152000	RN7SL601P	
17	11424001	11425000	SHISA6	
17	11605001	11606000	DNAH9	Cytoskeleton
17	11678001	11679000	DNAH9	Cytoskeleton
17	11775001	11776000	DNAH9	Cytoskeleton
17	11798001	11799000	DNAH9	Cytoskeleton
17	12623001	12624000	LINC00670	
17	12971001	12972000	ARHGAP44	
17	13853001	13854000	LOC100506974	
17	14834001	14835000	LINC02096	
17	15572001	15573000	TVP23C-CDRT4;TVP23C;CDRT1	Proteolysis
17	16074001	16075000	NCOR1;RPL22P21	Epigenetic
17	16224001	16225000	NCOR1;PIGL	Epigenetic;Metabolism
17	17158001	17159000	MPRIP	Cytoskeleton
17	17285001	17286000	COPS3	Protease
17	17319001	17320000	NT5M	
17	17722001	17723000	RAI1	Transcription
17	17798001	17799000	RAI1	Transcription
17	18031001	18032000	ATPAF2;GID4	Transcription
17	18050001	18051000	GID4	
17	18642001	18643000	TBC1D28;LOC645139	Signaling
17	18690001	18691000	ZNF286B;FOXO3B;TRIM16L	
17	18713001	18714000	TRIM16L	
17	18729001	18730000	TRIM16L	
17	19149001	19150000	GRAPL;GRAPL-AS1	Cytoskeleton
17	19381001	19382000	B9D1;LOC105371573;MAPK7;MFAP4	Development;Signaling;Signaling
17	19406001	19407000	RNF112	Signaling
17	19817001	19819000	ULK2;ULK2	Signaling;Signaling
17	20320001	20321000	SPECC1;CCDC144CP;RNU6-467P	
17	20437001	20438000	USP32P3;NOS2P3	
17	20988001	20989000	LOC339260	
17	21021001	21022000	USP22	Protease
17	21702001	21704000	KCNJ18;KCNJ18	Transport;Transport
17	22384001	22385000	LOC105377824;LOC107987246;LOC105377822	
17	22397001	22398000	LOC107987246;LOC105377822;FLJ36000	
17	28184001	28185000	NLK	Signaling
17	28224001	28225000	PYY2	
17	28605001	28606000	SPAG5;SPAG5-AS1;RSKR;KIAA0100	Signaling
17	28917001	28918000	PHF12;LOC101927018	Epigenetic
17	29052001	29053000	PIPOX	Metabolism
17	29504001	29505000	TAOK1	Signaling
17	29860001	29861000	SSH2;RPL9P30	Signaling
17	29968001	29969000	EFCAB5	Signaling

17	30026001	30027000	EFCAB5	Signaling
17	30101001	30102000	EFCAB5	Signaling
17	31563001	31564000	MIR193A;RNU6ATAC7P	
17	31880001	31881000	UTP6	Metabolism
17	32159001	32160000	RHOT1;ARGFXP2	Signaling
17	32526001	32527000	MYO1D;LOC105371734;LOC107985012	Cytoskeleton
17	32773001	32774000	MYO1D	Cytoskeleton
17	32789001	32790000	MYO1D	Cytoskeleton
17	32959001	32960000	LOC102724731	
17	33246001	33247000	ASIC2	Transport
17	34184001	34187000	LINC01989;LINC01989;LINC01989	;;
17	35027001	35028000	RFFL;RAD51L3-RFFL	Proteolysis
17	35282001	35283000	SLFN5	
17	35837001	35838000	TAF15	Metabolism
17	35882001	35883000	LOC105371744;CCL5;LOC105371745	Growth Factors
17	36379001	36380000	LOC100420852;TBC1D3H	Signaling
17	36639001	36640000	MRM1	Epigenetic
17	36661001	36663000	LOC105371750;LOC105371751;LOC105371750;LOC105371751	;
17	36754001	36755000	LOC105371750	
17	37052001	37053000	AATF;LOC105371753	Epigenetic
17	37350001	37352000	ACACA;ACACA	;
17	37425001	37426000	TADA2A	Transcription
17	37496001	37497000	DUSP14	
17	38057001	38059000	TBC1D3C;TBC1D3C	Signaling;Signaling
17	38413001	38414000	SOCS7;ARHGAP23	Signaling
17	38539001	38540000	SRCIN1	
17	39243001	39244000	LOC101929578;FBXL20	
17	39299001	39300000	FBXL20;MIR548BC	
17	39488001	39489000	CDK12	Signaling
17	39933001	39934000	ORMDL3;LRRC3C;LOC112268187	Receptor
17	39937001	39938000	ORMDL3;LRRC3C;LOC112268187	Receptor
17	39988001	39990000	PSMD3;PSMD3	Protease;Protease
17	40011001	40012000	CSF3;MED24	Transcription
17	40448001	40449000	RPL23AP75;IGFBP4;LOC107985064	Protease; Proteolysis
17	40512001	40513000	LOC105371773	
17	40974001	40975000	LOC107985072;KRT39;KRT40	
17	41571001	41572000	KRT9	
17	41633001	41634000	KRT17;KRT42P	
17	41760001	41761000	JUP	
17	41949001	41951000	ODAD4;ODAD4	;
17	42245001	42246000	STAT5B	Transcription
17	42373001	42374000	STAT3	Transcription
17	42458001	42459000	ATP6V0A1	Metabolism
17	42570001	42571000	COASY;MLX;PSMC3IP;RETREG3	Signaling;Transcription;Transcription
17	42686001	42687000	PLEKHH3;CCR10;CNTNAP1	
17	42860001	42861000	AOC2;AOC3;AOC4P	Metabolism
17	43001001	43002000	RUNDC1;RPL27;IFI35	Signaling;Translation;Transcription
17	43668001	43669000	MEOX1;LINC02594;LOC107985085	Development
17	43842001	43843000	MPP3;LOC107984979;CD300LG;LOC107985077	Cytoskeleton;Immune
17	43912001	43913000	MPP2;LOC105371787;FAM215A;LOC107985086;LRRC37A10P	Cytoskeleton
17	44125001	44126000	HDAC5;LOC105371789;RNU6-131P	
17	44237001	44238000	SHC1P2	
17	44772001	44773000	ADAM11	Protease
17	44982001	44983000	LOC112268183;LOC107987243	
17	45142001	45143000	PLCD3;ACBD4;HEXIM1	Metabolism;Transport
17	45153001	45154000	ACBD4;HEXIM1;HEXIM2;LOC112268203	Transport
17	45238001	45239000	FMNL1;LOC107985040;MAP3K14-AS1	
17	45454001	45455000	PLEKHM1	
17	45513001	45514000	LOC105369225;LRRC37A4P	
17	46107001	46108000	KANSL1	
17	46216001	46217000	KANSL1	
17	46553001	46554000	ARL17A;LRRC37A2;FAM215B	Signaling;Receptor
17	46622001	46623000	LRRC37A2;NSF	Receptor;Transport
17	47087001	47088000	LOC101927060	
17	49185001	49186000	B4GALNT2;TRQ-TTG1-1	
17	49245001	49246000	FLJ40194	

17	49331001	49332000	ZNF652	Transcription
17	49963001	49965000	RNU6-1313P;DLX4;RNU6-1313P;DLX4	Development;Development
17	50136001	50137000	SAMD14;PPP1R9B	
17	50262001	50263000	LOC105371819;LOC105371822;TMEM92	
17	50529001	50530000	MYCBPAP;EPN3;LOC105371824	
17	50554001	50555000	SPATA20;CACNA1G-AS1;CACNA1G	Transport
17	50682001	50683000	ABCC3	Transport
17	50883001	50884000	LOC105371825	
17	55401001	55402000	MMD	Signaling
17	55800001	55801000	PCTP	
17	56530001	56531000	LOC112268194	
17	56980001	56981000	LOC105371836;SCPEP1	Protease
17	57377001	57378000	MSI2	
17	57500001	57501000	MSI2	
17	58613001	58614000	TEX14	
17	58883001	58884000	PPM1E	Signaling
17	59180001	59181000	PRR11;SPDYE22P	
17	59348001	59349000	YPEL2	
17	59575001	59576000	DHX40	Transcription
17	59605001	59606000	DHX40	Transcription
17	60306001	60307000	USP32;H3P42	Protease
17	61075001	61076000	BCAS3;RPL23AP74	
17	61968001	61969000	MED13	
17	62434001	62435000	METTL2A	Epigenetic
17	62773001	62774000	MARCHF10	Proteolysis
17	63237001	63238000	TANC2	
17	64440001	64441000	MILR1	Immune
17	65005001	65006000	GNA13	Signaling
17	65085001	65086000	LOC105371864	
17	65145001	65146000	RGS9	
17	66185001	66187000	CEP112;CEP112	;
17	66550001	66551000	PRKCA	Signaling
17	66798001	66799000	PRKCA	Signaling
17	67217001	67218000	HELZ;RPL36AP48	
17	67539001	67540000	PITPNC1	Transport
17	68246001	68247000	AMZ2	
17	68654001	68655000	LINC01482	
17	69534001	69535000	MAP2K6	Signaling
17	69832001	69833000	LINC01483	
17	71621001	71622000	MYL6P5	
17	72413001	72414000	LINC00673	
17	72423001	72424000	LINC00673	
17	72731001	72732000	SLC39A11	Transport
17	72864001	72865000	SLC39A11	Transport
17	73093001	73094000	SLC39A11	Transport
17	73294001	73295000	CDC42EP4	
17	73368001	73369000	SDK2	
17	73402001	73403000	SDK2	
17	73776001	73777000	LINC00469;LINC02092	
17	74244001	74245000	TTYH2	Transport
17	74419001	74420000	RNA5SP448	
17	74551001	74552000	LOC107985074;CD300C;CD300H	Immune
17	75075001	75076000	KCTD2;TRIM80P	
17	75116001	75117000	SLC16A5;ARMC7	Transport
17	75195001	75196000	NUP85	Development
17	75280001	75281000	MIF4GD;MIF4GD-DT;SLC25A19	Metabolism;Transport
17	75688001	75689000	SAP30BP	Transcription
17	76402001	76403000	UBE2O	
17	76475001	76476000	AANAT;RHBDF2	Metabolism;Protease
17	77110001	77111000	SEC14L1;LOC105371900	
17	77169001	77170000	SEC14L1	
17	77173001	77174000	SEC14L1	
17	78090001	78091000	TNRC6C	Metabolism
17	78105001	78106000	TNRC6C;TMC6	Metabolism
17	78471001	78472000	DNAH17	Cytoskeleton
17	78721001	78722000	CYTH1	Transcription

17	78825001	78826000	USP36	Protease
17	78878001	78879000	TIMP2	Protease; Proteolysis
17	79001001	79002000	CANT1	Signaling
17	79300001	79301000	RBFOX3	Translation
17	79457001	79459000	RBFOX3;RBFOX3	Translation;Translation
17	79732001	79733000	ENPP7	
17	79986001	79987000	TBC1D16	Signaling
17	80002001	80003000	TBC1D16	Signaling
17	80148001	80149000	EIF4A3	
17	80315001	80316000	RNF213	
17	80570001	80571000	RPTOR;LOC105371922	
17	80621001	80622000	RPTOR	
17	80683001	80685000	RPTOR;RPTOR	;
17	80760001	80761000	RPTOR	
17	80795001	80796000	RPTOR;LOC101928855	
17	80821001	80822000	RPTOR	
17	80836001	80837000	RPTOR	
17	80840001	80841000	RPTOR	
17	81047001	81049000	BAIAP2;BAIAP2	Cytoskeleton;Cytoskeleton
17	81051001	81053000	BAIAP2;BAIAP2	Cytoskeleton;Cytoskeleton
17	81253001	81254000	SLC38A10	Transport
17	81260001	81261000	SLC38A10	Transport
17	81274001	81275000	SLC38A10	Transport
17	81305001	81306000	SLC38A10;LINC00482;TMEM105	Transport
17	81488001	81489000	LINC01971	
17	81793001	81794000	LOC105376789	
17	81997001	81998000	ASPSCR1	
17	82171001	82172000	CCDC57	
17	82237001	82238000	SLC16A3;MIR6787;CSNK1D	Transport;Signaling
17	82570001	82571000	FOXK2;ARL2BPP9;LOC105371942	
17	82801001	82802000	TBCD	Transcription
17	82848001	82849000	TBCD;ZNF750	Transcription
17	82919001	82920000	TBCD	Transcription
17	82938001	82939000	TBCD;B3GNTL1	Transcription;Golgi
18	21001	22000	LINC02564	
18	106001	107000	ROCK1P1;MIR8078	
18	228001	229000	THOC1	Metabolism
18	482001	483000	COLEC12	Extracellular Matrix
18	502001	503000	COLEC12;LINC01925	Extracellular Matrix
18	600001	601000	CLUL1	
18	708001	709000	ENOSF1	Metabolism
18	2132001	2133000	LOC105371956	
18	3197001	3198000	MYOM1	
18	3517001	3518000	DLGAP1	Cytoskeleton
18	3627001	3628000	DLGAP1	Cytoskeleton
18	3741001	3742000	DLGAP1	Cytoskeleton
18	4089001	4090000	DLGAP1	Cytoskeleton
18	4408001	4409000	DLGAP1	Cytoskeleton
18	4430001	4431000	DLGAP1	Cytoskeleton
18	7056001	7057000	LAMA1	Extracellular Matrix
18	7136001	7137000	SLC25A51P2	
18	7331001	7332000	LOC105371975	
18	8058001	8059000	PTPRM	Signaling
18	8104001	8105000	PTPRM	Signaling
18	8219001	8220000	PTPRM	Signaling
18	8622001	8623000	RAB12	
18	8682001	8683000	TOMM20P3	
18	9536001	9537000	RALBP1;PPP4R1	Signaling;Signaling
18	10706001	10708000	PIEZO2;PIEZO2	;
18	12738001	12739000	LINC01882	
18	12881001	12882000	PTPN2	Signaling
18	13308001	13309000	LDLRAD4	
18	13429001	13430000	LDLRAD4;LDLRAD4-AS1	
18	13448001	13451000	LDLRAD4;LDLRAD4;MIR5190;LDLRAD4;MIR5190	;;
18	13823001	13824000	MC5R	Signaling
18	13892001	13893000	MC2R	Signaling

18	14432001	14434000	LOC105372003;LOC105372003	;
18	15303001	15304000	LOC644669	
18	21601001	21602000	ESCO1	Metabolism
18	23236001	23237000	CABLES1;TMEM241	Transport
18	23373001	23374000	TMEM241	Transport
18	23756001	23757000	LAMA3	Extracellular Matrix
18	24166001	24167000	CABYR;OSBPL1A;RNA5SP452	
18	24373001	24374000	OSBPL1A	
18	24633001	24634000	LINC01915	
18	25142001	25143000	ZNF521	Transcription
18	26643001	26644000	KCTD1;LOC102725227	Cytoskeleton
18	32287001	32289000	GAREM1;GAREM1	;
18	32441001	32442000	GAREM1	
18	34998001	34999000	MAPRE2	Cytoskeleton
18	35358001	35359000	ZNF396	Transcription
18	35706001	35707000	GALNT1;LOC105372064	Golgi
18	35715001	35716000	GALNT1;LOC105372064	Golgi
18	35846001	35847000	NRBF2P1	
18	35963001	35964000	LOC105372066;C18orf21	
18	36052001	36053000	RPRD1A	Signaling
18	37414001	37415000	CELF4	
18	39442001	39443000	MIR924HG	
18	40140001	40141000	RPL17P45	
18	41977001	41978000	PIK3C3	Signaling
18	42387001	42388000	LINC00907	
18	42739001	42740000	RIT2	Signaling
18	42878001	42879000	RIT2	Signaling
18	43108001	43109000	RIT2	Signaling
18	44826001	44827000	SETBP1	Epigenetic
18	44937001	44938000	SETBP1	Epigenetic
18	45857001	45858000	EPG5	
18	46170001	46171000	C18orf25	
18	46453001	46454000	RNF165	Proteolysis
18	46698001	46699000	ST8SIA5	Transport
18	47025001	47026000	KATNAL2;ELOA3BP;ELOA3P;ELOA2	Cytoskeleton
18	47034001	47036000	KATNAL2;ELOA3BP;ELOA3P;ELOA2;KATNAL2;ELOA3P;ELOA2	Cytoskeleton;Cytoskeleton
18	48603001	48604000	CTIF	Metabolism
18	48856001	48857000	CTIF	Metabolism
18	49039001	49040000	LOC100129878;DYM;MIR4744	
18	49071001	49072000	DYM	
18	49493001	49494000	C18orf32;RPL17-C18orf32;MIR1539;RPL17;SNORD58C;SNORD58A;SNORD58B;SRP72P1;LINC02837	Translation
18	50157001	50158000	MYO5B	Cytoskeleton
18	50283001	50284000	MBD1;CXXC1	
18	50402001	50404000	SKA1;LOC105372115;SKA1;LOC105372115	;
18	50830001	50831000	MRO;RPL17P46	
18	52792001	52793000	DCC	
18	53349001	53350000	DCC	
18	55156001	55157000	RNA5SP459	
18	55458001	55459000	TCF4;TCF4-AS1	Transcription
18	56639001	56640000	TXNL1	Metabolism
18	57696001	57697000	ATP8B1	Transport
18	58390001	58391000	NEDD4L	Proteolysis
18	59062001	59063000	OACYLP	
18	59064001	59065000	OACYLP	
18	59896001	59897000	PMAIP1	
18	62350001	62351000	TNFRSF11A	Receptor
18	62380001	62381000	TNFRSF11A	Receptor
18	62812001	62813000	PHLPP1	Cytoskeleton
18	62948001	62949000	PHLPP1	Cytoskeleton
18	63103001	63106000	LOC105372161;LOC105372161;LOC105372161	;;
18	63199001	63200000	BCL2	
18	63948001	63949000	HMSD	Protease; Proteolysis
18	63959001	63960000	HMSD;LOC105372165	Protease; Proteolysis
18	67742001	67743000	DSEL-AS1;LOC105372173	
18	68765001	68766000	CCDC102B	

18	69469001	69470000	DOK6;LOC105372179	
18	69674001	69675000	DOK6	
18	69839001	69840000	DOK6	
18	70325001	70326000	SOCS6;LINC01909;LIVAR	Signaling
18	71197001	71198000	LOC105372188	
18	74616001	74618000	ZNF407;ZNF407	Transcription;Transcription
18	75235001	75236000	TSHZ1	Transcription
18	76815001	76816000	ZNF236-DT;ZNF236	Transcription
18	76884001	76885000	ZNF236;LOC100421894	Transcription
18	76967001	76968000	ZNF236	Transcription
18	77258001	77259000	GALR1	Signaling
18	77527001	77528000	LOC107985172	
18	77869001	77870000	RNA5SP461	
18	78638001	78639000	LOC105372221	
18	79073001	79074000	LOC105372225;ATP9B	Transport
18	79482001	79483000	NFATC1;LOC107985162	Transcription
18	79524001	79526000	NFATC1;LOC102723506;NFATC1;LOC102723506	Transcription;Transcription
18	79579001	79580000	LOC284240	
18	79605001	79607000	LOC105372228;LOC105372228	;
18	79633001	79634000	LOC284241	
18	79831001	79832000	KCNG2	Transport
19	302001	304000	MIER2;MIER2	Development;Development
19	309001	310000	MIER2	Development
19	433001	434000	SHC2	Cytoskeleton
19	466001	467000	SHC2;ODF3L2	Cytoskeleton;Development
19	477001	478000	ODF3L2	Development
19	499001	500000	ODF3L2;MADCAM1;MADCAM1-AS1;TPGS1	Development;Cytoskeleton
19	520001	521000	TPGS1	Cytoskeleton
19	586001	587000	BSG;HCN2;LOC100420586	Cytoskeleton;Transport
19	703001	705000	PRSS57;PALM;PRSS57;PALM	Protease;Protease
19	1141001	1142000	SBNO2	
19	1288001	1289000	FAM174C;EFNA2	Signaling
19	1362001	1363000	PWWP3A	Transcription
19	1386001	1387000	PWWP3A;TRF-GAA1-6;TRN-GTT2-6;NDUFS7	Transcription;Metabolism
19	1389001	1390000	TRF-GAA1-6;TRN-GTT2-6;NDUFS7;GAMT	Metabolism;Epigenetic
19	1615001	1616000	UQCR11;TCF3;RNU6-1223P	Metabolism;Transcription
19	1657001	1658000	TCF3	Transcription
19	2030001	2031000	LOC107985278;MKNK2	Signaling
19	2349001	2350000	SPPL2B	Proteolysis
19	2439001	2440000	LMNB2;MIR7108	
19	2669001	2671000	GNG7;GNG7	Signaling;Signaling
19	2932001	2933000	LOC101928631;ZNF77	Transcription
19	2939001	2940000	ZNF77	Transcription
19	3069001	3071000	TLE5;LOC105372242;TLE5;LOC105372242	Transcription;Transcription
19	3127001	3128000	GNA11;LOC390876;GNA15	Signaling
19	3199001	3200000	NCLN	
19	3201001	3202000	NCLN	
19	3254001	3255000	CELF5	
19	3278001	3279000	CELF5	
19	3533001	3534000	FZR1;MFSD12;C19orf71	Proteolysis
19	3546001	3547000	FZR1;MFSD12;C19orf71	Proteolysis
19	3563001	3565000	MFSD12;HMG20B;MFSD12;HMG20B	;
19	3613001	3614000	TBXA2R;CACTIN-AS1;CACTIN	Signaling;Cytoskeleton
19	3649001	3650000	PIP5K1C	Signaling
19	3677001	3679000	PIP5K1C;PIP5K1C	Signaling;Signaling
19	3883001	3884000	ATCAY	
19	3900001	3901000	ATCAY	
19	3979001	3980000	DAPK3;EEF2;SNORD37	Signaling;Translation
19	4087001	4088000	MAP2K2	Signaling
19	4177001	4178000	CREB3L3;SIRT6;ANKRD24	
19	4188001	4189000	SIRT6;ANKRD24	
19	4190001	4191000	SIRT6;ANKRD24	
19	4201001	4202000	ANKRD24	
19	4329001	4330000	FSD1;STAP2	Proteolysis;Cytoskeleton
19	4385001	4386000	SH3GL1	
19	4503001	4504000	HDGFL2;PLIN4	Transcription

19	4511001	4512000	HDGFL2;PLIN4	Transcription
19	4729001	4730000	DPP9;LOC105372250;TRG-TCC1-1;TRV-CAC3-1	Protease
19	4784001	4785000	FEM1A	
19	4789001	4790000	FEM1A	
19	4930001	4931000	UHRF1;MIR4747	Proteolysis
19	4946001	4947000	UHRF1	Proteolysis
19	4999001	5000000	KDM4B	Epigenetic
19	5056001	5057000	KDM4B	Epigenetic
19	5109001	5110000	KDM4B	Epigenetic
19	5119001	5120000	KDM4B	Epigenetic
19	5271001	5272000	PTPRS;RPL32P34	Signaling
19	5594001	5595000	SAFB2	
19	5916001	5918000	VMAC;CAPS;RANBP3;VMAC;CAPS;RANBP3	Signaling;Cytoskeleton;Signaling;Cytoskeleton
19	6299001	6300000	ACER1	
19	6532001	6533000	TNFSF9	
19	6657001	6658000	TNFSF14	
19	6708001	6709000	C3	Protease; Proteolysis
19	7031001	7032000	MBD3L2B;MBD3L5;MBD3L4	
19	7096001	7097000	ZNF557	Transcription
19	7142001	7143000	INSR	Receptor
19	7450001	7451000	ARHGEF18	
19	7467001	7468000	ARHGEF18;PEX11G	
19	7558001	7559000	PNPLA6	Metabolism
19	7645001	7647000	PCP2;STXBP2;PCP2;STXBP2	Transport;Transport
19	7683001	7684000	MCEMP1;TRAPPC5;FCER2	Transport
19	7828001	7829000	EVI5L	Signaling
19	7843001	7844000	EVI5L	Signaling
19	8025001	8026000	LOC105372266	
19	8052001	8053000	CCL25	Growth Factors
19	8124001	8125000	FBN3	Extracellular Matrix
19	8315001	8316000	CD320;NDUFA7;RPS28;KANK3	Binding Proteins;Metabolism;Translation;Cytoskeleton
19	8826001	8827000	ZNF558;MBD3L1	Transcription
19	9137001	9138000	ZNF317	
19	9182001	9183000	OR7D2;ELOCP29;OR7E16P	Receptor
19	9288001	9289000	OR7H1P;ZNF699	
19	9395001	9396000	LOC112268250	
19	9675001	9676000	ZNF562;RPS4XP22	Transcription
19	9700001	9701000	ZNF812P;LOC105372270	
19	9751001	9752000	ZNF846;LOC100419831	Transcription
19	9770001	9772000	ZNF846;UBE2L4;ZNF846;UBE2L4	Transcription;Transcription
19	9917001	9918000	OLFM2	Development
19	10096001	10097000	SHFL;ANGPTL6;PPAN;PPAN-P2RY11	Signaling;Metabolism
19	10126001	10127000	EIF3G;DNMT1	Translation;Epigenetic
19	10361001	10362000	TYK2	
19	10589001	10590000	AP1M2;LOC100820734	Transport
19	10807001	10808000	DNM2;MIR199A1	Transport
19	10851001	10852000	C19orf38;HIKESHIP2	
19	11008001	11009000	SMARCA4;RN7SL192P	Epigenetic
19	11067001	11068000	SMARCA4	Epigenetic
19	11135001	11136000	LDLR;SPC24	Binding Proteins
19	11159001	11160000	SPC24;KANK2	Cytoskeleton
19	11183001	11184000	KANK2	Cytoskeleton
19	11340001	11341000	RAB3D;TMEM205;CCDC159	
19	11590001	11591000	ZNF627;GAPDHP76	Transcription
19	11815001	11816000	ZNF491;ZNF440	Transcription
19	11927001	11928000	ZNF69;ZNF700	Transcription
19	12001001	12002000	ZNF433-AS1;RNA5SP464	
19	12155001	12156000	LOC105372275;ZNF625-ZNF20;ZNF625;ZNF136	Transcription
19	12752001	12753000	GET3;BEST2	Transport;Transport
19	12768001	12769000	BEST2;HOOK2	Transport;Transport
19	12781001	12782000	HOOK2;MIR5684;JUNB	Transport;Transcription
19	12808001	12809000	PRDX2;THSD8;RNASEH2A	Metabolism;Translation
19	12911001	12912000	SYCE2;MIR5695	
19	12972001	12974000	DAND5;DAND5	;
19	13318001	13319000	CACNA1A	Transport
19	13762001	13763000	YJU2B;MRI1	Metabolism

19	13801001	13802000	ZSWIM4;LOC107985334	
19	13825001	13826000	ZSWIM4;LOC107985334;RN7SL619P;MIR23AHG	
19	13894001	13896000	BRME1;BRME1	;
19	13937001	13938000	CC2D1A;PODNL1	
19	13960001	13961000	PODNL1;DCAF15;RFX1	Transcription
19	14177001	14178000	ADGRL1-AS1;ADGRL1	Signaling
19	14183001	14184000	ADGRL1	Signaling
19	14378001	14379000	ADGRE5	Signaling
19	14549001	14550000	TECR;LOC112268253	Metabolism
19	14676001	14677000	ADGRE3;LOC107985308	Signaling
19	14841001	14842000	OR7C1;OR7A5;OR7A10	Receptor
19	15672001	15673000	CYP4F3;CYP4F10P;CYP4F12	Metabolism
19	15819001	15820000	OR10H1;ZNF861P;LINC01764;UCA1	Signaling
19	16015001	16016000	LOC105372291;LINC00661	
19	16069001	16070000	LINC01855;TPM4	Cytoskeleton
19	16172001	16173000	CIB3	
19	16193001	16194000	FAM32A;AP1M1	Transport
19	16357001	16358000	EPS15L1	Transport
19	16378001	16379000	EPS15L1	Transport
19	16821001	16823000	NWD1;SIN3B;NWD1;SIN3B	Epigenetic;Epigenetic
19	16875001	16876000	SIN3B	Epigenetic
19	16977001	16978000	CPAMD8	Protease; Proteolysis
19	17086001	17087000	MYO9B;SNORA118	
19	17089001	17090000	MYO9B;SNORA118	
19	17364001	17365000	PLVAP	
19	17628001	17629000	UNC13A	
19	17761001	17762000	FCHO1	Cytoskeleton
19	17808001	17809000	B3GNT3;INSL3	Golgi
19	17841001	17842000	JAK3	
19	18283001	18284000	IQCN;JUND;MIR3188;RPL39P38	Transcription
19	18767001	18768000	CRTC1	Transcription
19	18911001	18913000	COPE;DDX49;COPE;DDX49	Transport;Transport
19	18993001	18994000	SUGP2	Translation
19	19036001	19037000	SUGP2;ARMC6	Translation
19	19285001	19286000	SUGP1	Translation
19	19453001	19454000	GATAD2A	Transcription
19	19502001	19503000	GATAD2A	Transcription
19	19574001	19575000	PBX4	Development
19	19582001	19583000	PBX4;PHF5CP	Development
19	19610001	19611000	PBX4	Development
19	20092001	20093000	ZNF90;BNIP3P14	Transcription
19	20531001	20532000	LOC105372316;ZNF737	Transcription
19	22235001	22236000	LOC105372328	
19	22642001	22643000	ZNF492	Transcription
19	22672001	22673000	ZNF492	Transcription
19	22833001	22834000	ZNF723;BNIP3P35	Transcription
19	22925001	22926000	LOC105372332	
19	23878001	23879000	RPSAP58	
19	27788001	27789000	LINC00662;LOC101927151	
19	29697001	29698000	C19orf12	
19	32254001	32255000	RNA5SP472	
19	32445001	32446000	DPY19L3	
19	32451001	32452000	DPY19L3	
19	32596001	32597000	PDCD5;ANKRD27	Signaling
19	32638001	32639000	ANKRD27;RPS12P31	
19	32721001	32722000	NUDT19;LOC105372368;TDRD12	Cytoskeleton
19	33201001	33202000	LRP3;SLC7A10	Binding Proteins;Transport
19	33233001	33234000	SLC7A10	Transport
19	33404001	33406000	PEPD;PEPD	Protease;Protease
19	33458001	33459000	PEPD	Protease
19	34133001	34134000	CHCHD2P3	
19	34167001	34168000	LSM14A	Metabolism
19	34183001	34184000	LSM14A	Metabolism
19	34256001	34257000	GARRE1	
19	34350001	34351000	GARRE1;GPI	Metabolism
19	34598001	34599000	ZNF807P;SCGB1B2P;SCGB2B2	

19	34929001	34930000	ZNF30-AS1;ZNF30	
19	34998001	34999000	GRAMD1A	
19	35002001	35003000	GRAMD1A	
19	35307001	35308000	MAG	Immune
19	35521001	35522000	DMKN;SBSN	
19	35719001	35720000	ZBTB32;KMT2B	Transcription;Epigenetic
19	35780001	35781000	PROSER3;ARHGAP33;LINC01529	Signaling
19	35873001	35874000	KIRREL2;LOC107985317;APLP1;RN7SL402P	Protease; Proteolysis
19	35944001	35945000	LRFN3;LOC105372383	
19	36121001	36122000	WDR62;OVOL3;POLR2I;TBCB;LOC105372385	Transcription;Transcription
19	36305001	36306000	LOC100134317;LINC00665	
19	36308001	36309000	LOC100134317;LINC00665	
19	36356001	36357000	ZFP14	
19	36492001	36493000	ZNF566;ZNF566-AS1;CTBP2P7	Transcription
19	36666001	36667000	ZNF461;ZNF567	Transcription
19	36945001	36946000	ZNF568	
19	37234001	37235000	ZNF383	Transcription
19	37271001	37272000	LINC01535;LOC284412	
19	38176001	38177000	SIPA1L3	Signaling
19	38183001	38184000	SIPA1L3	Signaling
19	38408001	38409000	SPRED3;FAM98C;RASGRP4	Cytoskeleton;Translation;Transcription
19	38417001	38418000	FAM98C;RASGRP4	Translation;Transcription
19	38572001	38573000	RYR1	Ion Channel
19	38597001	38598000	RYR1;MAP4K1;MAP4K1-AS1	Ion Channel
19	38738001	38739000	ACTN4;LOC107985291;CAPN12	Protease
19	39174001	39175000	PAK4	Signaling
19	39308001	39309000	IFNL1;LRFN1	Cytokine
19	39392001	39393000	SAMD4B;PAF1;MED29	Transcription
19	39659001	39660000	LGALS16	Extracellular Matrix
19	39669001	39670000	LGALS16;LGALS17A	Extracellular Matrix
19	39878001	39879000	FCGBP	Extracellular Matrix
19	40118001	40119000	VN1R96P	
19	40224001	40225000	MAP3K10;TTC9B;CCNP;AKT2	Signaling;Transcription;Signaling
19	40280001	40281000	AKT2;MIR641	Signaling
19	40402001	40403000	PRX	
19	40762001	40763000	SNRPA	Translation
19	41055001	41056000	CYP2G2P	
19	41186001	41187000	RN7SL718P;CYP2S1	Metabolism
19	41324001	41325000	CCDC97;TGFB1	Growth Factors
19	41747001	41748000	CEACAM6;LOC112268252	
19	42070001	42072000	GRIK5;ZNF574;GRIK5;ZNF574	Receptor;Transcription;Receptor;Transcription
19	42369001	42371000	MEGF8;MEGF8	Extracellular Matrix;Extracellular Matrix
19	43221001	43222000	LOC284344;CEACAMP10	
19	43246001	43248000	LOC284344;PSG9;LOC284344;PSG9	;
19	43407001	43409000	TEX101;LOC110467528;TEX101;LOC110467528	;
19	44866001	44867000	NECTIN2	
19	45141001	45142000	PPP1R37;NKPD1	
19	45183001	45184000	TRAPPC6A;BLOC1S3	
19	45225001	45226000	EXOC3L2;LOC100420902	Transport
19	45239001	45240000	EXOC3L2;LOC100420902	Transport
19	45360001	45361000	KLC3;ERCC2	Cytoskeleton;Epigenetic
19	45377001	45378000	ERCC2;PPP1R13L	Epigenetic
19	45616001	45617000	EML2	
19	45881001	45882000	FOXA3;IRF2BP1;MYPOP	Transcription;Transcription;Transcription
19	46240001	46242000	IGFL1;IGFL1	Growth Factors;Growth Factors
19	46334001	46335000	HIF3A	Transcription
19	46596001	46597000	PPP5D1P;CALM3	Signaling
19	46820001	46821000	HNRNPMP2;LOC107985273;SNAR-E	
19	46831001	46832000	LOC107985273;SNAR-E;AP2S1	Transport
19	46978001	46980000	ARHGAP35;ARHGAP35	Signaling;Signaling
19	47192001	47193000	SAE1	Proteolysis
19	47700001	47701000	BICRA	
19	47742001	47743000	EHD2;NOP53	Transport
19	47755001	47756000	NOP53;SNORD23;NOP53-AS1	
19	47837001	47838000	CRX	Development
19	47893001	47894000	SULT2A1	Transport

19	47916001	47917000	SNAR-A12;SNAR-C5;SNAR-A1;SNAR-A3	
19	47920001	47921000	SNAR-C5;SNAR-A1;SNAR-A3;SNAR-C2	
19	47938001	47940000	SNAR-C2;SNAR-A2;SNAR-C4;SNAR-A13;SNAR-A2;SNAR-C4;SNAR-A13	;
19	48109001	48110000	PLA2G4C;LIG1	Metabolism;Methylation
19	48342001	48343000	TMEM143	
19	48359001	48360000	TMEM143;SYNGR4	Transport
19	48374001	48375000	SYNGR4;KDELR1	Transport
19	48378001	48379000	SYNGR4;KDELR1	Transport
19	48600001	48601000	SULT2B1;FAM83E;SPACA4	Transport
19	48604001	48605000	SULT2B1;FAM83E;SPACA4	Transport
19	48935001	48936000	DHDH	Metabolism
19	49238001	49239000	LOC107985340	
19	49243001	49244000	LOC107985340;SLC6A21P	
19	49518001	49519000	FCGRT;RCN3	Immune;Signaling
19	50086001	50087000	SNAR-A4	
19	50127001	50128000	SNAR-A9;SNAR-A10;SNAR-A11;SNAR-B1;LOC105372436	
19	50132001	50133000	SNAR-A10;SNAR-A11;SNAR-B1;LOC105372436;SNAR-B2;SNAR-D	
19	50244001	50245000	MYH14	
19	50246001	50247000	MYH14	
19	50435001	50436000	SPIB;MYBPC2	Transcription
19	50475001	50476000	MYBPC2;FAM71E1;EMC10	Epigenetic
19	50518001	50519000	JOSD2;ASPDH;LRRC4B	Protease;Metabolism
19	50549001	50550000	LRRC4B	
19	50658001	50659000	C19orf81;SHANK1	
19	50874001	50875000	KLK2;KLKP1	Protease
19	51278001	51279000	LOC107985327;SIGLECL1;LINC01872;SIGLEC24P;LOC107985326;LOC100420904	Immune
19	51595001	51596000	ZNF175;LINC01530;LOC339352	
19	52183001	52184000	PPP2R1A	Signaling
19	52307001	52308000	ZNF480	
19	52677001	52678000	ZNF83	Transcription
19	52684001	52685000	ZNF83	Transcription
19	52818001	52819000	ZNF28;PABPN1P2	
19	52853001	52854000	ZNF468;ZNF320	
19	53280001	53281000	VN1R4;FAM90A27P;BIRC8	Receptor;Protease; Proteolysis
19	53469001	53470000	VN1R102P;ZNF813;TPM3P6	
19	53597001	53598000	LOC284379;DPRX;LOC100422610	Development
19	53974001	53975000	CACNG8;MIR935	Transport
19	54036001	54037000	VSTM1	Immune
19	54337001	54338000	LILRA4	Immune
19	54497001	54498000	LAIR2	Immune
19	54512001	54513000	LAIR2;LOC105372460	Immune
19	54675001	54676000	LILRB4;LOC102724290	Immune
19	54821001	54822000	KIR2DL4;KIR3DL1	Immune
19	55039001	55040000	GP6-AS1;GP6;RDH13	Immune;Golgi
19	55052001	55054000	GP6-AS1;RDH13;RDH13	Golgi;Golgi
19	55122001	55123000	PPP1R12C;MIR7975;TNNT1	Signaling;Cytoskeleton
19	55210001	55211000	PTPRH	Signaling
19	55366001	55367000	FAM71E2;IL11;TMEM190	Cytokine
19	55401001	55402000	RPL28;UBE2S;SNORD157;LOC105372462	Translation;Proteolysis
19	55508001	55509000	SSC5D	Protease
19	55745001	55746000	NLRP9;RN7SKP109;LOC105372465	
19	55785001	55786000	RFPL4AP1;NLRP11	
19	56197001	56198000	ZSCAN5B;ZSCAN5C	Transcription
19	56266001	56267000	ZSCAN5A;EDDM13	Transcription
19	56287001	56288000	ZSCAN5A;EDDM13;LOC105372470	Transcription
19	56347001	56348000	ZSCAN5A;ZSCAN5A-AS1;VN2R18P;LOC646709	Transcription
19	56400001	56401000	ZNF582;ZNF582-DT;ZNF583	Transcription
19	56609001	56610000	ZNF71-SMIM17;ZNF71	Transcription
19	56742001	56743000	LOC105372472	
19	57143001	57144000	ZIM3	
19	57415001	57417000	ZNF17;ZNF17	;
19	57856001	57857000	ZNF587B;ZNF587	Transcription
19	57910001	57911000	ZNF417	
19	58421001	58422000	ZNF584	
19	58498001	58499000	ZNF446;RN7SL693P;SLC27A5;LOC105372485	Transcription;Transport
20	306001	307000	ZCCHC3	Epigenetic

20	453001	454000	TBC1D20	Signaling
20	1781001	1782000	LOC100289473;LOC107984104	
20	1832001	1833000	LOC107984104;LOC105372501	
20	2472001	2473000	SNRPB;SNORD119;ZNF343	Translation;Transcription
20	2531001	2532000	ZNF343;LOC105372504;TMC2	Transcription
20	2725001	2726000	EBF4	Transcription
20	2782001	2783000	RPL19P1	
20	2898001	2899000	PTPRA	Signaling
20	3073001	3074000	LOC101929098;OXT;AVP	Signaling
20	3105001	3106000	UBOX5-AS1;UBOX5	
20	3232001	3233000	ITPA;SLC4A11	Signaling;Transport
20	3396001	3397000	DNAAF9	
20	3757001	3758000	HSPA12B;C20orf27	
20	3850001	3851000	MAVS	
20	3881001	3882000	MAVS;PANK2-AS1;PANK2	Signaling
20	4178001	4179000	SMOX	Metabolism
20	4183001	4184000	SMOX;LINC01433	Metabolism
20	4987001	4988000	SLC23A2	Transport
20	5758001	5759000	SHLD1	
20	8826001	8827000	PLCB1;RNU105B	Metabolism
20	9598001	9599000	PAK5;LOC105372523	Signaling
20	10044001	10045000	SNAP25-AS1;ANKEF1	
20	14129001	14130000	MACROD2;AIMP1P1	
20	14466001	14467000	MACROD2	
20	15518001	15520000	MACROD2;MACROD2	;
20	16352001	16353000	KIF16B	Cytoskeleton
20	17294001	17296000	PCSK2;LOC105372546;PCSK2;LOC105372546	Protease;Protease
20	17624001	17625000	RRBP1	Cytoskeleton
20	17653001	17654000	RRBP1	Cytoskeleton
20	17807001	17808000	LOC107985440	
20	17888001	17889000	LOC105372548	
20	18612001	18613000	DTD1;RNU6ATAC34P	Metabolism
20	18817001	18818000	SCP2D1-AS1;SCP2D1	Transport
20	19724001	19725000	SLC24A3	Transport
20	19755001	19756000	LOC107987276;RIN2	Transcription
20	19818001	19819000	RIN2;LOC100421663;RPL12P12	Transcription
20	19903001	19904000	RIN2	Transcription
20	21405001	21406000	NKX2-4;LOC105372558	Development
20	22445001	22446000	LOC105372562	
20	22467001	22468000	LOC105372562;LOC105372563	
20	23340001	23341000	NXT1-AS1;LOC105372571;NXT1	
20	23423001	23424000	NAPB	Transport
20	24349001	24350000	LOC105372577	
20	24521001	24522000	SYNDIG1	
20	25406001	25407000	LOC105372581;GINS1	Transcription
20	25535001	25536000	NINL	
20	25591001	25592000	NINL	
20	29095001	29096000	FRG1DP	
20	29298001	29299000	LOC110467521;LOC110467538;LOC107987277	
20	29326001	29328000	DUX4L32;PCMTD1P7;DUX4L33;DUX4L32;PCMTD1P7;DUX4L33	;
20	29427001	29428000	RARRES2P11;AGGF1P10	
20	29875001	29876000	RNA5SP532;DUX4L37	
20	30295001	30296000	LINC01597;LOC107985433;LOC105372586	
20	30490001	30491000	LOC110467523;LOC102723618;LOC110467535;CDC27P4	
20	30505001	30506000	LOC110467526;LOC105379481	
20	30676001	30677000	ANKRD20A21P	
20	30909001	30910000	DUX4L39;CDC27P5	
20	31315001	31316000	DEFB116;RPL31P3	
20	31737001	31738000	TPX2	Cytoskeleton
20	32008001	32009000	XKR7;RNA5SP481;CCM2L	
20	33147001	33148000	LOC105372714	
20	33548001	33549000	CBFA2T2	Transcription
20	33722001	33723000	PXMP4;ZNF341	Transcription
20	33725001	33726000	PXMP4;ZNF341	Transcription
20	34298001	34299000	AHCY	Metabolism
20	34602001	34603000	PIGU	

20	34725001	34726000	NCOA6	Epigenetic
20	35160001	35161000	MMP24-AS1-EDEM2	
20	35367001	35368000	UQCC1	Transcription
20	35761001	35762000	RPF2P1	
20	35891001	35893000	PHF20;RNU4-40P;PHF20;RNU4-40P	;
20	35933001	35934000	PHF20;RNU6-937P	
20	36208001	36209000	EPB41L1;LOC105372602	
20	36217001	36218000	EPB41L1;LOC105372602	
20	36491001	36492000	DLGAP4	Cytoskeleton
20	36535001	36536000	DLGAP4;DLGAP4-AS1;MYL9	Cytoskeleton;Cytoskeleton
20	37314001	37315000	MANBAL	
20	37384001	37385000	SRC	
20	37572001	37573000	LINC01746	
20	37932001	37933000	VSTM2L	
20	37938001	37939000	VSTM2L	
20	38410001	38411000	SNHG17	
20	38512001	38513000	RALGAPB;MIR548O2	
20	38541001	38543000	RALGAPB;RPS3P2;RALGAPB;RPS3P2	;
20	38651001	38652000	ARHGAP40	Signaling
20	38971001	38972000	DHX35;NPM1P19	Transcription
20	40638001	40639000	LOC102724968	
20	41412001	41413000	CHD6	
20	42075001	42076000	LOC101927182;PTPRT	Signaling
20	42697001	42698000	PTPRT;LOC101927159	Signaling
20	43129001	43130000	PTPRT	Signaling
20	44185001	44187000	JPH2;OSER1;JPH2;OSER1	;
20	44408001	44409000	HNF4A;MIR3646	Transcription
20	45102001	45103000	KCNS1;WFDC5	Transport;Protease; Proteolysis
20	45910001	45911000	PLTP	
20	46595001	46596000	SLC13A3	Transport
20	46976001	46977000	EYA2	
20	47190001	47191000	EYA2	
20	47413001	47414000	LINC01754	
20	47698001	47699000	SULF2	Metabolism
20	47844001	47845000	LOC105372636	
20	47855001	47856000	SRMP1	
20	48124001	48126000	LOC105372640;LOC105372640	;
20	48202001	48203000	LOC105372641	
20	48473001	48474000	LOC105372645;LOC107985439	
20	48629001	48631000	PREX1;PREX1	Transcription;Transcription
20	48651001	48652000	PREX1	Transcription
20	48722001	48723000	PREX1	Transcription
20	49458001	49459000	KCNB1	Transport
20	49702001	49703000	B4GALT5	Golgi
20	49958001	49959000	RNF114;KRT18P4	
20	49994001	49995000	SNAI1	Transcription
20	50179001	50180000	LINC01273;CEBPB-AS1	
20	50264001	50265000	LOC105372656;PELATON	
20	50817001	50818000	BCAS4	
20	51433001	51434000	NFATC2	Transcription
20	51653001	51654000	ATP9A	Transport
20	52153001	52154000	ZFP64	Transcription
20	53610001	53611000	ZNF217;LOC105372672	Transcription
20	53630001	53631000	LOC105372672	
20	55616001	55617000	LOC105372676	
20	55819001	55820000	LOC105372677	
20	57723001	57724000	LOC105372692	
20	58276001	58277000	PPP4R1L	Signaling
20	58681001	58682000	STX16-NPEPL1;STX16;NPEPL1	Transcription;Protease
20	58755001	58756000	PIEZO1P2	
20	59121001	59122000	MRPS16P2;ZNF831	
20	59307001	59308000	EDN3	Hormone
20	59772001	59773000	PHACTR3	Signaling
20	59774001	59775000	PHACTR3	Signaling
20	60148001	60149000	MIR646HG	
20	61087001	61088000	LINC01718	

20	61375001	61376000	CDH4	Cytoskeleton
20	61672001	61673000	CDH4	Cytoskeleton
20	61809001	61810000	CDH4	Cytoskeleton
20	61839001	61840000	CDH4	Cytoskeleton
20	61850001	61851000	CDH4	Cytoskeleton
20	61981001	61982000	TAF4	Transcription
20	62152001	62153000	PSMA7;SS18L1	Protease;Transcription
20	62291001	62292000	OSBPL2	
20	62322001	62323000	LAMA5;MIR4758	Extracellular Matrix
20	62328001	62329000	LAMA5;MIR4758	Extracellular Matrix
20	62431001	62432000	RBBP8NL;LOC105372710	Transcription
20	62694001	62695000	SLCO4A1	Transport
20	62732001	62733000	NTSR1	Signaling
20	62877001	62878000	LOC105372717;ARF4P2;DIDO1;SNORA117	Transcription
20	62879001	62880000	LOC105372717;ARF4P2;DIDO1;SNORA117	Transcription
20	62943001	62944000	DIDO1;GID8;SLC17A9	Transcription;Cytoskeleton;Transport
20	63044001	63045000	LINC01749;LINC00029;LINC01056	
20	63288001	63289000	ARFGAP1;MIR4326;COL20A1	Signaling;Extracellular Matrix
20	63396001	63398000	KCNQ2;KCNQ2	Transport;Transport
20	63576001	63577000	HELZ2	
20	63578001	63579000	HELZ2;GMEB2	
20	63688001	63689000	RTEL1-TNFRSF6B;RTEL1;TNFRSF6B;ARFRP1	Epigenetic;Receptor
20	63690001	63691000	RTEL1-TNFRSF6B;RTEL1;TNFRSF6B;ARFRP1	Epigenetic;Receptor
20	63801001	63802000	ZBTB46;ZBTB46-AS1	Cytoskeleton
20	63925001	63926000	DNAJC5;MIR941-1;MIR941-2;MIR941-3;MIR941-4;MIR941-5	Transcription
20	64059001	64060000	TCEA2;SOX18	Transcription;Development
20	64096001	64097000	OPRL1;NPBWR2	Signaling
20	64235001	64236000	MYT1	Transcription
21	5032001	5033000	LOC102723996	
21	5037001	5038000	LOC102723996	
21	6666001	6667000	LOC102724701;LOC102724677	
21	6802001	6803000	LOC102724726	
21	7052001	7053000	LOC105379501;LOC102724843	
21	8202001	8204000	MIR6724-1;RNA45SN2;MIR3648-1;RNA18SN2;RNA5-8SN2;MIR6724-1;RNA45SN2;MIR3648-1;RNA18SN2;RNA5-8SN2;RNA28SN2	;
21	8219001	8220000	RNA45SN2;RNA18SN2;RNA5-8SN2;RNA28SN2;LOC105379506;LOC107987293	
21	8221001	8223000	RNA45SN2;RNA18SN2;RNA5-8SN2;RNA28SN2;LOC105379506;LOC107987293;RNA45SN2;RNA5-8SN2;RNA28SN2;LOC105379506;LOC107987293	;
21	8224001	8225000	RNA45SN2;RNA28SN2;LOC105379506;LOC107987293	
21	8227001	8228000	RNA45SN2;RNA28SN2;LOC105379506;LOC107987293	
21	8231001	8234000	LOC105379506;LOC107987293;CDC27P9;LOC105379506;LOC107987293;CDC27P9;LOC105379506;LOC107987293;CDC27P9	;;
21	8235001	8236000	LOC107987293;CDC27P9	
21	8240001	8241000	CDC27P9;MIR6724-2	
21	8243001	8246000	CDC27P9;MIR6724-2;RNA18SP5;CDC27P9;MIR6724-2;RNA18SP5;CDC27P9;MIR6724-2;RNA18SP5	;;
21	8247001	8249000	CDC27P9;MIR6724-2;RNA18SP5;RNA5-8SP10;CDC27P9;MIR6724-2;RNA18SP5;RNA5-8SP10;LOC110467529	;
21	8385001	8386000	MIR6724-3;RNA45SN3;RNA18SN3;RNA5-8SN3	
21	8387001	8388000	MIR6724-3;RNA45SN3;RNA18SN3;RNA5-8SN3;RNA28SN3	
21	8404001	8408000	RNA45SN3;RNA18SN3;RNA5-8SN3;RNA28SN3;LOC105379508;RNA45SN3;RNA5-8SN3;RNA28SN3;LOC105379508;RNA45SN3;RNA28SN3;LOC105379508;RNA45SN3;RNA28SN3;LOC105379508	;;;
21	8409001	8411000	RNA45SN3;RNA28SN3;LOC105379508;RNA45SN3;RNA28SN3;LOC105379508	;
21	8413001	8414000	LOC105379508;CDC27P10	
21	8415001	8416000	LOC105379508;CDC27P10	
21	8419001	8420000	CDC27P10	
21	8421001	8424000	CDC27P10;CDC27P10;MIR6724-4;CDC27P10;MIR6724-4;RNA45SN1	;;
21	8425001	8426000	CDC27P10;MIR6724-4;RNA45SN1	
21	8428001	8429000	CDC27P10;MIR6724-4;RNA45SN1;MIR10396B;RNA18SN1	
21	8431001	8432000	CDC27P10;MIR6724-4;RNA45SN1;MIR10396B;RNA18SN1;RNA5-8SN1;RNA28SN1	
21	8448001	8450000	RNA45SN1;RNA18SN1;RNA5-8SN1;RNA28SN1;LOC105379507;LOC107987294;RNA45SN1;RNA5-8SN1;RNA28SN1;LOC105379507;LOC107987294	;
21	8451001	8453000	RNA45SN1;RNA28SN1;LOC105379507;LOC107987294;RNA45SN1;RNA28SN1;LOC105379507;LOC107987294	;

21	8454001	8455000	RNA45SN1;RNA28SN1;LOC105379507;LOC107987294	
21	8459001	8460000	LOC105379507;LOC107987294;CDC27P11	
21	8462001	8463000	LOC107987294;CDC27P11	
21	8464001	8466000	LOC107987294;CDC27P11;CDC27P11	;
21	8467001	8468000	CDC27P11	
21	8472001	8473000	CDC27P11	
21	8757001	8758000	LINC01666	
21	8806001	8807000	LINC01666;SNX18P10	
21	8809001	8810000	LINC01666;SNX18P10;LOC107985475	
21	8811001	8812000	LINC01666;SNX18P10;LOC107985475	
21	9082001	9083000	TEKT4P2;SOWAHCP2;SNX18P11	
21	9129001	9130000	TEKT4P2	
21	9544001	9545000	NCOR1P4	
21	9801001	9802000	LINC01667	
21	9818001	9820000	LINC01667;LINC01667	;
21	10106001	10107000	LOC105372733	
21	10330001	10331000	EIF3FP1	
21	10427001	10428000	BAGE2	
21	10468001	10469000	BAGE2	
21	10477001	10478000	BAGE2	
21	10652001	10653000	IGHV1OR21-1	Immune
21	10654001	10657000	IGHV1OR21-1;IGHV1OR21-1;IGHV1OR21-1	Immune;Immune;Immune
21	13065001	13066000	ANKRD30BP2	
21	14083001	14084000	ERLEC1P1	
21	15262001	15263000	LOC105369292	
21	16481001	16482000	MIR99AHG	
21	17751001	17752000	LOC105372741	
21	18551001	18552000	MIR548XHG	
21	18724001	18725000	MIR548XHG	
21	19121001	19122000	LOC107985490	
21	26992001	26993000	LOC112268279	
21	28140001	28141000	LINC01697;LINC01695	
21	28162001	28163000	LINC01695	
21	28642001	28643000	N6AMT1	Epigenetic
21	29093001	29094000	MAP3K7CL	
21	31252001	31253000	TIAM1;LOC105372777	
21	31517001	31518000	TIAM1	
21	32584001	32585000	CFAP298-TCP10L;TCP10L;LOC105372782	Cytoskeleton
21	32587001	32588000	CFAP298-TCP10L;TCP10L;LOC105372782	Cytoskeleton
21	33348001	33349000	IFNAR1	Receptor
21	33363001	33364000	IFNAR1	Receptor
21	33717001	33718000	ITSN1	Transport
21	34029001	34030000	LOC105372790;LOC105372789	
21	34134001	34135000	MRPS6;RPS5P2	Translation
21	34144001	34146000	MRPS6;RPS5P2;MRPS6;RPS5P2	Translation;Translation
21	34747001	34748000	LINC00160;LOC107985515;LINC01426	
21	34822001	34823000	RUNX1	Transcription
21	35565001	35566000	LOC100506403	
21	36064001	36065000	SETD4;SETD4-AS1;RNU6-992P;LOC100133286;CBR1	Epigenetic;Metabolism
21	36237001	36238000	DOP1B	
21	36276001	36277000	DOP1B	
21	36295001	36296000	DOP1B;SRSF9P1;RPS26P1	
21	36419001	36420000	CHAF1B	Epigenetic
21	36463001	36464000	LOC105369301;CLDN14	Cell Junction
21	36763001	36764000	HLCS	Metabolism
21	37043001	37044000	RNU6-696P	
21	37744001	37745000	KCNJ6;KCNJ6-AS1;LOC101928368	Transport
21	37753001	37754000	KCNJ6;LOC101928368	Transport
21	38361001	38362000	LOC107985513;ERG	Transcription
21	38423001	38424000	ERG	Transcription
21	38469001	38470000	ERG	Transcription
21	38733001	38734000	LINC00114	
21	38914001	38915000	LOC400867	
21	39372001	39373000	LOC105372804;RNF6P1;GET1-SH3BGR;GET1	
21	40033001	40034000	DSCAM	Cytoskeleton
21	40481001	40482000	DSCAM	Cytoskeleton

21	40791001	40792000	DSCAM	Cytoskeleton
21	41150001	41151000	LINC00323;LOC107985495	
21	41431001	41432000	MX1	Transport
21	41687001	41688000	LINC00111	
21	41722001	41724000	LINC00479;LINC00112;LINC00479;LINC00112	;
21	41772001	41773000	RIPK4;LOC102724800	Signaling
21	41830001	41831000	PRDM15	Transcription
21	41983001	41984000	ZBTB21	Transcription
21	42305001	42306000	ABCG1;TFF3	Transport;Signaling
21	42548001	42549000	SLC37A1	Transport
21	42564001	42565000	SLC37A1;LOC101928212	Transport
21	42666001	42667000	PDE9A;LOC105372816	Signaling
21	42861001	42862000	WDR4	Translation
21	42918001	42920000	NDUFV3;ERVH48-1;NDUFV3;ERVH48-1	Metabolism;Metabolism
21	43353001	43354000	LINC01679	
21	43743001	43744000	PDXK;LOC105372824	Signaling
21	43805001	43806000	RRP1;AATBC	
21	43846001	43848000	MYL6P1;MYL6P1	;
21	43920001	43921000	AGPAT3;RNU6-859P	Metabolism
21	44395001	44396000	TRPM2	Transport
21	44427001	44428000	TRPM2;TRPM2-AS	Transport
21	44642001	44643000	TSPEAR;KRTAP10-10;KRTAP10-11	Signaling
21	44698001	44699000	TSPEAR;KRTAP10-12;KRTAP10-13P	Signaling
21	45530001	45531000	SLC19A1;LOC107987304	Transport
21	45874001	45875000	PCBP3	Metabolism
21	45893001	45894000	PCBP3	Metabolism
21	45941001	45943000	PCBP3;PCBP3	Metabolism;Metabolism
21	46077001	46078000	PSMA6P3	
21	46091001	46092000	COL6A2	Extracellular Matrix
21	46331001	46332000	C21orf58;PCNT	
21	46363001	46364000	PCNT	
22	11700001	11701000	LOC107987320	
22	11820001	11821000	LOC107984037;LOC107984030	
22	11956001	11957000	LOC102723769	
22	12172001	12173000	LOC105379516;LOC107987322	
22	12175001	12177000	LOC105379516;LOC105379516	;
22	12699001	12700000	DUX4L44	
22	15543001	15544000	ARHGAP42P3	
22	15689001	15690000	POTEH	Transport
22	15761001	15762000	PSLNR;LOC107987324	
22	16584001	16585000	LOC100422375;KCNMB3P1;CCT8L2;FABP5P11	Translation
22	16614001	16615000	TPTEP1	
22	16742001	16743000	LINC01665	
22	17179001	17180000	ADA2	Metabolism
22	17342001	17343000	RN7SL843P	
22	17450001	17451000	CECR2	
22	17487001	17488000	CECR2	
22	17656001	17657000	BCL2L13	
22	18140001	18141000	TUBA8;USP18	Cytoskeleton;Protease
22	18220001	18221000	LOC105372858;LOC101060852	
22	18377001	18378000	FAM230J	
22	18493001	18494000	FAM230A	
22	18595001	18596000	RIMBP3;RN7SKP131	
22	18786001	18787000	GGT3P	Protease
22	19226001	19227000	CLTCL1	Transport
22	19252001	19253000	CLTCL1;DVL1P1;KRT18P62	Transport;Cytoskeleton
22	19567001	19568000	LINC00895	
22	19727001	19728000	SEPTIN5;SEPT5-GP1BB;GP1BB;LOC105372861	Cytoskeleton
22	19765001	19766000	TBX1	Transcription
22	19768001	19769000	TBX1	Transcription
22	19870001	19871000	TXNRD2	Metabolism
22	20002001	20003000	ARVCF	Cytoskeleton
22	20551001	20552000	MED15	
22	20716001	20717000	PI4KA	Signaling
22	20876001	20877000	SNAP29	Transcription
22	21060001	21061000	LRRC74B;TUBA3GP	

22	21228001	21229000	GGT2	Protease
22	21246001	21247000	GGT2	Protease
22	21558001	21559000	RIMBP3C;UBE2L3	Proteolysis
22	21645001	21646000	CCDC116;SDF2L1;LOC107985532;MIR301B;MIR130B	Transport
22	21752001	21753000	MAPK1	Signaling
22	21911001	21912000	PPM1F	Signaling
22	21925001	21926000	PPM1F	Signaling
22	21949001	21950000	PPM1F;PPM1F-AS1;TOP3B	Signaling;Transcription
22	22581001	22582000	IGL;IGLV2-34;IGLV2-33	Immune
22	23377001	23378000	CES5AP1	
22	23436001	23437000	LINC01659	
22	23578001	23579000	LOC105372957;IGLL1;DRICH1	Immune
22	23759001	23760000	ZNF70;VPREB3;C22orf15;CHCHD10;LOC107985577	Transcription;Immune
22	24133001	24134000	CABIN1	Signaling
22	24263001	24264000	POM121L9P;BCRP1;RPS10P29;SPECC1L-ADORA2A;SPECC1L	
22	24577001	24578000	SNRPD3;GGT1;LRRC75B	Translation;Protease
22	24646001	24647000	BCRP3;POM121L10P	
22	24799001	24800000	SGSM1	Signaling
22	24956001	24957000	TMEM211;KIAA1671	
22	25075001	25076000	KIAA1671	
22	25118001	25119000	KIAA1671;KIAA1671-AS1	
22	25845001	25846000	MYO18B	
22	26600001	26601000	TPST2;CRYBB1;CRYBA4	Transport
22	26742001	26744000	MIATNB;MIATNB	;
22	26910001	26911000	LINC01422	
22	26914001	26915000	LINC01422	
22	27743001	27745000	MN1;MN1	;
22	27786001	27787000	MN1	
22	27922001	27923000	PITPNB;TTC28-AS1;MIR3199-1;MIR3199-2	Transport
22	28540001	28541000	TTC28;LOC101929594	Cytoskeleton
22	28688001	28689000	TTC28;CHEK2	Cytoskeleton;Signaling
22	29548001	29549000	THOC5;NIPSNAP1	
22	29710001	29711000	CABP7	
22	29940001	29941000	MTMR3	Signaling
22	29984001	29985000	MTMR3	Signaling
22	30452001	30453000	SEC14L3;LOC105372991	
22	30696001	30697000	OSBP2	
22	30868001	30869000	OSBP2;LOC105372994	
22	31184001	31185000	RNF185	Proteolysis
22	31277001	31278000	LIMK2;PPP1R14BP1;PIK3IP1	Protease
22	31556001	31557000	SFI1;RPS18P14	
22	31711001	31712000	PRR14L	
22	31823001	31824000	DEPDC5	Signaling
22	32209001	32210000	RFPL2;SLC5A4-AS1;SLC5A4	Transport
22	33437001	33438000	LARGE1;MIR4764	Golgi
22	33458001	33459000	LARGE1	Golgi
22	33502001	33503000	LARGE1	Golgi
22	33527001	33528000	LARGE1	Golgi
22	33827001	33828000	LARGE1	Golgi
22	35232001	35233000	LINC01399;LOC105373017	
22	35553001	35554000	RASD2	
22	35586001	35587000	LOC107985590	
22	35633001	35634000	MB;LOC284912	
22	36598001	36599000	CACNG2	Transport
22	36606001	36607000	CACNG2	Transport
22	37422001	37423000	ELFN2	Receptor
22	37482001	37483000	MFNG;CARD10	Golgi
22	37851001	37852000	ANKRD54;MIR658;MIR659;EIF3L	Translation
22	37892001	37893000	EIF3L;RNU6-900P	Translation
22	38114001	38115000	BAIAP2L2;PLA2G6	Cytoskeleton;Metabolism
22	38620001	38621000	FAM227A	
22	38679001	38680000	CBY1;TOMM22;JOSD1	Transport;Protease
22	38875001	38876000	CBX6	
22	39035001	39036000	APOBEC3F;APOBEC3D	Translation
22	39572001	39573000	CACNA1I	Transport
22	39649001	39650000	CACNA1I	Transport

22	39824001	39825000	ENTHD1	
22	40274001	40275000	TNRC6B	Metabolism
22	40286001	40287000	TNRC6B	Metabolism
22	40684001	40685000	GAPDHP37;MCHR1	Signaling
22	40957001	40958000	RBX1	Proteolysis
22	40983001	40984000	RBX1	Proteolysis
22	41228001	41229000	L3MBTL2;CHADL	Epigenetic;Receptor
22	41250001	41251000	CHADL;RANGAP1;MIR6889	Receptor;Signaling
22	41252001	41253000	RANGAP1;MIR6889	Signaling
22	41486001	41487000	ACO2	Metabolism
22	41529001	41530000	ACO2;POLR3H	Metabolism;Transcription
22	41593001	41594000	PMM1;DESI1	Metabolism;Protease
22	41632001	41633000	XRCC6	Epigenetic
22	41936001	41937000	TNFRSF13C;CENPM	Receptor
22	42845001	42846000	ARFGAP3;LOC692246	Signaling
22	42968001	42969000	PACSIN2	Cytoskeleton
22	43068001	43069000	TTLL1	Cytoskeleton
22	43075001	43076000	TTLL1	Cytoskeleton
22	43217001	43219000	SCUBE1;SCUBE1-AS2;SCUBE1;SCUBE1-AS2	Extracellular Matrix;Extracellular Matrix
22	43275001	43276000	SCUBE1;SCUBE1-AS1	Extracellular Matrix
22	43353001	43354000	SCUBE1	Extracellular Matrix
22	43605001	43606000	EFCAB6	Signaling
22	43775001	43776000	EFCAB6	Signaling
22	43972001	43973000	SAMM50	
22	44323001	44324000	SHISAL1;LOC101927499	Cytoskeleton
22	44335001	44336000	SHISAL1	Cytoskeleton
22	44738001	44739000	PRR5;PRR5-ARHGAP8	
22	44740001	44741000	PRR5;PRR5-ARHGAP8	
22	44987001	44988000	PHF21B	
22	45205001	45206000	KIAA0930;MIR1249	
22	45590001	45591000	FBLN1	
22	45890001	45891000	LOC107985535;LOC105373071	
22	45932001	45933000	WNT7B	Signaling
22	46215001	46216000	PPARA	Transcription
22	46219001	46220000	PPARA	Transcription
22	46240001	46241000	PPARA;CDPF1	Transcription
22	46566001	46568000	GRAMD4;GRAMD4	;
22	46889001	46891000	TBC1D22A;TBC1D22A	Signaling;Signaling
22	46910001	46911000	TBC1D22A;TBC1D22A-AS1	Signaling
22	47210001	47211000	TBC1D22A	Signaling
22	48337001	48339000	LOC105373080;LOC105373080	;
22	48509001	48510000	TAFA5	Growth Factors
22	48566001	48567000	TAFA5;LOC105373083	Growth Factors
22	48584001	48585000	TAFA5	Growth Factors
22	48670001	48671000	TAFA5	Growth Factors
22	49527001	49528000	MIR3667HG;LOC105373087	
22	49569001	49570000	MIR3667HG	
22	49615001	49616000	MIR3667HG	
22	49823001	49824000	BRD1;LOC100420885;LOC105377205	Transcription
22	49872001	49873000	ZBED4;ALG12	Golgi
22	49901001	49902000	ALG12	Golgi
22	50029001	50030000	TTLL8	
22	50380001	50381000	PPP6R2;RN7SL500P	Signaling
22	50548001	50549000	LOC102724608;KLHDC7B-DT;KLHDC7B;SYCE3	
22	50700001	50701000	SHANK3;RNU6-409P	
22	50733001	50734000	SHANK3;LOC105373100;ACR	Protease
22	50807001	50809000	RPL23AP82;RPL23AP82	;
X	395001	396000	PPP2R3B	Signaling
X	457001	459000	LOC102724521;LOC102724521	;
X	471001	472000	LOC102724521	
X	1233001	1235000	LOC107985697;LOC107985697	;
X	1262001	1263000	LOC107985697;CSF2RA	Receptor
X	1386001	1387000	IL3RA;LOC101928055;SLC25A6;LOC105373102	Receptor;Transport
X	1395001	1397000	SLC25A6;LOC105373102;LINC00106;ASMTL-AS1;ASMTL;SLC25A6;LOC105373102;LINC00106;ASMTL-AS1;ASMTL	Transport;Transport
X	1447001	1449000	ASMTL;ASMTL	;

X	1585001	1586000	AKAP17A;ASMT	Epigenetic
X	2274001	2275000	DHRSX	Metabolism
X	2489001	2490000	DHRSX;ZBED1	Metabolism;Transcription
X	2949001	2950000	ARSL	Metabolism
X	6534001	6535000	VCX3A	
X	6973001	6974000	PUDP	Signaling
X	8033001	8034000	LOC107985675	
X	8219001	8220000	LOC107985675	
X	9399001	9400000	LOC442443;LOC107985634	
X	9612001	9613000	TBL1X	
X	9760001	9761000	GPR143	Signaling
X	10567001	10568000	MID1	Proteolysis
X	10602001	10603000	MID1	Proteolysis
X	12538001	12539000	FRMPD4	
X	12848001	12849000	MRPL35P4	
X	13279001	13280000	LINC02154	
X	13303001	13304000	LINC02154;LOC107985701;GS1-600G8.3	
X	14769001	14770000	FANCB	
X	16806001	16807000	TXLNG;RPL12P49	Transport
X	17061001	17062000	REPS2	Transport
X	19357001	19358000	PDHA1;MAP3K15	Metabolism;Signaling
X	23283001	23284000	PTCHD1-AS	
X	23737001	23738000	ACOT9	Metabolism
X	24461001	24462000	PDK3	Signaling
X	24563001	24565000	PCYT1B;PCYT1B	Transport;Transport
X	26557001	26558000	VENTXP8;VENTXP1	
X	27284001	27285000	LOC105373150	
X	28072001	28073000	LOC105373152	
X	28504001	28506000	MIR6134;MIR6134	;
X	29077001	29078000	IL1RAPL1	Receptor
X	29746001	29747000	IL1RAPL1	Receptor
X	30571001	30572000	TASL	
X	31241001	31242000	DMD	
X	31934001	31935000	DMD	
X	32305001	32306000	DMD	
X	33834001	33835000	LOC105373153	
X	36175001	36176000	CFAP47	Development
X	37460001	37461000	PRRG1	Protease
X	37631001	37632000	LANCL3	
X	38653001	38654000	TSPAN7	
X	40147001	40148000	BCOR	
X	40589001	40590000	ATP6AP2	Receptor
X	41612001	41613000	CASK	Cytoskeleton
X	41915001	41916000	CASK;RNU6-202P	Cytoskeleton
X	44514001	44515000	FUNDC1	
X	44972001	44973000	KDM6A	Epigenetic
X	45138001	45139000	DIPK2B	
X	45141001	45142000	DIPK2B	
X	45156001	45157000	DIPK2B	
X	45171001	45172000	DIPK2B	
X	46655001	46656000	SLC9A7;PGAM1P7	Transport
X	48130001	48131000	SSX6P;SPACA5B	
X	49047001	49048000	TFE3;CCDC120	
X	49327001	49328000	GAGE10;GAGE12J;GAGE13	
X	49339001	49340000	GAGE12J;GAGE13;GAGE12B	
X	49549001	49550000	GAGE12C;GAGE12D;GAGE12E	
X	49589001	49590000	GAGE12H;GAGE2A;GAGE1	
X	50440001	50441000	DGKK	Signaling
X	51815001	51816000	MAGED1	Cytoskeleton
X	52204001	52205000	LOC105377209	
X	54357001	54358000	WNK3	Signaling
X	56765001	56766000	NBDY	
X	56995001	56996000	SPIN3	
X	62845001	62846000	MTND1P31;MTND2P25	
X	63497001	63498000	LINC01278	
X	63634001	63635000	ARHGEF9	Transcription

X	63735001	63736000	ARHGEF9	Transcription
X	64848001	64849000	LOC105373239	
X	64999001	65000000	ZC4H2	
X	65131001	65132000	ZC3H12B	Translation
X	65598001	65599000	MSN;LOC107985679	Cytoskeleton
X	66200001	66201000	HEPH	Metabolism
X	68653001	68654000	COX6CP12;STARD8	Signaling
X	69215001	69216000	LINC00269	
X	70151001	70152000	IGBP1;LOC107985683	Transcription
X	70716001	70717000	TEX11	
X	70965001	70966000	RPS23P8	
X	71199001	71200000	LOC107985688	
X	71324001	71325000	ZCRB1P1	
X	71408001	71409000	TAF1	Transcription
X	71710001	71711000	LINC00891;CXorf49	
X	71788001	71789000	LOC100132741	
X	71872001	71873000	LOC100289206	
X	72173001	72174000	PIN4	
X	72325001	72326000	HDAC8	
X	72729001	72730000	CAPZA1P3	
X	72782001	72783000	DMRTC1B;FAM226B;FAM236B	Transcription
X	73832001	73833000	TSIX;XIST	
X	74248001	74249000	FTX;RN7SL648P	
X	74764001	74765000	NEXMIF	
X	77541001	77542000	ATRX	Transcription
X	77553001	77554000	ATRX	Transcription
X	78100001	78101000	PGK1	Metabolism
X	84221001	84222000	MIR548I4	
X	89911001	89912000	TGIF2LX	Development
X	92215001	92216000	PCDH11X	Cytoskeleton
X	96627001	96629000	SKP2P1;SKP2P1	;
X	96708001	96709000	DIAPH2	
X	96739001	96740000	DIAPH2	
X	97056001	97057000	DIAPH2	
X	97373001	97374000	DIAPH2	
X	97612001	97613000	DIAPH2	
X	100985001	100986000	ARL13A;TRMT2B	Epigenetic
X	101056001	101057000	TRMT2B	Epigenetic
X	101321001	101322000	RNU6-934P	
X	101328001	101329000	RNU6-934P;NANOGNBP3	
X	101382001	101383000	BTK;RPL36A-HNRNPH2;RPL36A	Translation
X	101600001	101601000	ARMCX7P	
X	103375001	103377000	BEX3;BEX3	;
X	104094001	104095000	SLC25A53	
X	104185001	104186000	FAM199X	
X	106469001	106470000	LOC105373306;NAP1L4P2	
X	107507001	107508000	FRMPD3;FRMPD3-AS1	
X	107581001	107582000	FRMPD3	
X	107857001	107858000	MID2	Proteolysis
X	107996001	107997000	LOC112267910	
X	108598001	108599000	COL4A5	Extracellular Matrix
X	112394001	112395000	RTL4	
X	112434001	112435000	RTL4	
X	114603001	114605000	HTR2C;HTR2C	Signaling;Signaling
X	115012001	115013000	IL13RA2	Receptor
X	115661001	115662000	LOC107985682;PHF5FP;ASS1P5	
X	115851001	115852000	DANT2;DANT1	
X	116722001	116723000	LOC105373320	
X	119069001	119070000	LOC727838;KIAA1210	
X	119235001	119236000	NUDT19P6;PGRMC1	Receptor
X	119253001	119254000	PGRMC1;AKAP17BP	Receptor
X	119341001	119342000	LOC101928336	
X	119587001	119588000	UBE2A;NKRF	Transcription
X	119614001	119615000	NKRF;SEPTIN6	Transcription;Cytoskeleton
X	119676001	119677000	SEPTIN6	Cytoskeleton
X	120740001	120741000	GLRX5P1	

X	121044001	121045000	LOC105373328;GLUD2	Metabolism
X	123602001	123603000	THOC2;FERP1	Transcription
X	128537001	128538000	LOC107985698	
X	128782001	128783000	LOC107985699	
X	130065001	130066000	BCORL1;ELF4	Transcription
X	130165001	130166000	RAB33A;AIFM1	Metabolism
X	130250001	130251000	ZNF280C	Transcription
X	132132001	132133000	FRMD7;AGKP2	
X	132911001	132912000	HS6ST2;NAA20P1	Transport
X	133419001	133420000	GPC4	
X	134548001	134550000	MIR450B;MIR450A1;MIR450A2;MIR542;MIR503HG;MIR503;MIR424;LINC00629;MIR450B;MIR450A1;MIR450A2;MIR542;MIR503HG;MIR503;MIR424;LINC00629	;
X	135159001	135160000	CT55	
X	135832001	135833000	CT45A7	Transcription
X	138674001	138675000	FGF13;MIR504	Growth Factors
X	139857001	139858000	ATP11C	Transport
X	139871001	139872000	ATP11C	Transport
X	141002001	141003000	SPANXB1	
X	141529001	141530000	SPANXA2-OT1	
X	142175001	142176000	LOC392555	
X	143504001	143505000	SPANXN3	
X	148792001	148793000	AFF2	Transcription
X	148916001	148917000	AFF2	Transcription
X	149535001	149536000	IDS2;IDSP1;LINC00893;EOLA1	
X	149711001	149712000	MAGEA11	Cytoskeleton
X	150364001	150365000	MAMLD1	
X	150398001	150399000	MAMLD1	
X	150846001	150847000	CD99L2	
X	150870001	150871000	CD99L2	
X	152103001	152104000	LOC105373369	
X	152292001	152293000	GABRA3	Ion Channel
X	152582001	152583000	LOC105373370	
X	152727001	152728000	MAGEA2B;CSAG1;MAGEA12;CSAG4	Cytoskeleton
X	153446001	153447000	TREX2;HAUS7	
X	153713001	153714000	BCAP31	Transport
X	153814001	153815000	PDZD4	
X	153858001	153860000	L1CAM;L1CAM	;
X	153926001	153927000	ARHGAP4;NAA10;RENBP	Signaling;Metabolism;Metabolism
X	154409001	154411000	RPL10;SNORA70;DNASE1L1;TAFAZZIN;RPL10;SNORA70;DNASE1L1;TAFAZZIN	Translation;Translation
X	154883001	154884000	F8;H2AB1;F8A1;MIR1184-1	Metabolism;Epigenetic
X	154887001	154888000	F8;H2AB1;F8A1;MIR1184-1	Metabolism;Epigenetic
Y	11221001	11222000	LOC105379273	
Y	11296001	11298000	DUX4L16;DUX4L16	;
Y	11301001	11302000	DUX4L16	
Y	11306001	11307000	DUX4L16;DUX4L17	
Y	11308001	11309000	DUX4L16;DUX4L17	
Y	11317001	11318000	DUX4L16;DUX4L17;DUX4L18	
Y	11329001	11330000	DUX4L18;DUX4L19;PABPC1P5	
Y	11331001	11332000	DUX4L18;DUX4L19;PABPC1P5;SLC9B1P1	
Y	26639001	26640000	PARP4P1	
Y	26644001	26645000	PARP4P1	
